# Child anthropometry data quality from Demographic and Health Surveys, Multiple Indicator Cluster Surveys, and National Nutrition Surveys in the West Central Africa region: are we comparing apples and oranges?

**DOI:** 10.1080/16549716.2017.1328185

**Published:** 2017-06-22

**Authors:** Daniel J. Corsi, Jessica M. Perkins, S. V. Subramanian

**Affiliations:** ^a^ OMNI Research Group, Ottawa Hospital Research Institute, Ottawa, ON, Canada; ^b^ Center for Population & Development Studies, Harvard School of Public Health, Cambridge, MA, USA; ^c^ MGH Global Health, Massachusetts General Hospital, Boston, MA, USA; ^d^ Department of Social and Behavioral Sciences, Harvard T.H. Chan School of Public Health, Boston, MA, USA

**Keywords:** Undernutrition, child health, data quality, anthropometry, DHS, MICS, NNS, height, weight

## Abstract

**Background**: There has been limited work comparing survey characteristics and assessing the quality of child anthropometric data from population-based surveys.

**Objective**: To investigate survey characteristics and indicators of quality of anthropometric data in children aged 0–59 months from 23 countries in the West Central Africa region.

**Methods**: Using established methodologies and criteria to examine child age, sex, height, and weight, we conducted a comprehensive assessment and scoring of the quality of anthropometric data collected in 100 national surveys.

**Results**: The Multiple Indicator Cluster Surveys (MICS) and Demographic and Health Surveys (DHS) collected data from a greater number of younger children than older children while the opposite was found for the National Nutrition Surveys (NNS). Missing or implausible height/weight data proportions were 12% and 8% in MICS and DHS compared to 3% in NNS. Average data quality scores were 14 in NNS, 33 in DHS, and 41 in MICS.

**Conclusions**: Although our metric of data quality suggests that data from the NNS appear more consistent and robust, it is equally important to consider its disadvantages related to access and lack of broader socioeconomic information. In comparison, the DHS and MICS are publicly-accessable for research and provide socioeconomic context essential for assessing and addressing the burden of undernutrition within and between countries. The strengths and weaknesses of data from these three sources should be carefully considered when seeking to determine the burden of child undernutrition and its variation within countries.

## Background

Population-based surveys such as the Demographic and Health Surveys (DHS), National Nutrition Surveys (NNS), and Multiple Indicator Cluster Surveys (MICS) are important sources of information on child health and nutritional status in low-income countries [1,2]. Anthropometric measurement in large-scale surveys is a complex and difficult undertaking. Inaccuracies and other deficiencies in the quality of the anthropometric data collected by these surveys arise frequently and may have important implications for understanding the burden of malnutrition at the population level in low-income settings. Potential threats to high data quality may occur across various research stages, from survey and questionnaire development, to training, fieldwork, and data entry, to data cleaning and analysis. First, variability inherently exists in the precision and validity of anthropometric measurement tools (e.g. measuring tapes/boards, scales, calipers) [3]. In addition, errors may arise when using measurement instruments including when reading and recording measurements. These can be identified using on-site digital data input or in-field checks [4]. Second, fieldworker variation has the potential to dramatically affect the output of analyses using the recorded data  although this source of data error may be less apparent than error due to variation in instrument use [5]. Moreover, fieldworkers tend to have a subtle directional bias in their measurements, which may go unnoticed without undertaking specialized analyses (for example digit preference) and can often be exacerbated when there are multiple fieldworkers collecting data [6,7]. Finally, the study population may not be representative of the base population being studied [8].

Measures to address data error and low data quality vary. Some studies may alter data recording protocols throughout the collection phase. Alternatively, researchers may test for evidence of systematic differences in data trends based on recording practices after data collection is finished [4]. Previous studies have implemented repeatability and test–retest measures to look at both measurement performance within fieldworkers on separate occasions and measurement comparison between different fieldworkers on the same subject [9]. Such reliability studies serve to give fieldworkers an opportunity to self-correct and, if necessary, allow data to be adjusted post-collection based on trends among individual fieldworkers. The World Health Organization (WHO) has compiled a database of expected anthropometric measurements that can serve as a standardization tool for analysis [2]. Several individual papers also present standardization corrections for analysis given expected error ranges [6].

The Emergency Nutrition Assessment (ENA) and Standardized Monitoring and Assessment of Relief and Transitions (SMART) provide a basic, integrated survey method for assessing nutritional status in emergency and surveillance situations [10]. Methodologies used in SMART and built into the ENA software package, which can be used in the field, incorporate a quality assessment for nutrition data focusing on several issues: terminal digit preference, prevalence of implausible or missing values for anthropometric and demographic data (especially for age), and implausible age and sex ratios (which may indicate a non-representative sample) [2,10–12]. A composite quality score based on these parameters can indicate overall data quality as well as specific issues around missing data, heaping/rounding of values, and implausible age or sex distributions and can be disseminated to researchers [2].

The use of a standard overall data quality score facilitates comparisons of anthropometric data quality across surveys and survey sources. Preliminary analyses suggest that prevalence estimates of child wasting (low weight-for-height) in DHS/MICS may be biased by 3–5% at a national level due to variation in criteria for excluding implausible values for height and/or weight [13]. The current study assesses multiple characteristics of three survey sources  (DHS, NNS, and MICS) and the quality of anthropometric data produced through surveys conducted between 1990 and 2012 in the UNICEF-designated West Central Africa region. This region is the only one where the three survey programs currently operate. Three data quality issues were assessed: (1) incorrect measurement of child age (which is important for determining whether a child is stunted or underweight), (2) incorrect measurement of height, and (3) incorrect measurement of weight.

## Methods

### Data sources

A total of 100 surveys providing information on anthropometric measures (i.e. child age, sex, height – or length if under 2 years, and weight) in children aged 0–59 months were available from 23 countries in the United Nations Children’s Fund (UNICEF)-designated West Central Africa region. There were 45 DHS covering 19 countries, 27 NNS covering 13 countries, and 28 MICS covering 16 countries. Each of the survey programs partners with local nodal organizations and agencies to collect data with technical support and financial assistance from the United States Agency for International Development (USAID; DHS), UNICEF (MICS and NNS), and other national and international sources. Supplemental Table 1 indicates the specific countries included in this study. The data collection procedures were approved by the relevant institutional review board in each country. Oral or written informed consent for the survey was obtained from respondents by interviewers. This analysis was reviewed by the Harvard School of Public Health Institutional Review Board and was considered exempt from full review because the study was based on a de-identified and anonymous data-set available for secondary analyses.

DHS are large, standardized, household surveys produced by the Demographic and Health Surveys Program [14]. The targeted sample is based on nationally representative sampling plans. The surveys emphasize data collection on standardized measures of fertility and child mortality, and indicators of access to maternal and child health interventions, illness, treatment, and nutritional status [1]. These surveys also collect an extensive set of standardized socioeconomic indicators and other such information. These data-sets are fully open-access. Based on DHS measurement protocols, all children of selected mothers who are of appropriate age based on the three- or five-year reference window are potentially eligible for measurement. In some surveys, one-third, one-half, or two-thirds subsamples of children are selected for anthropometric measurements. In addition, in the more recent DHS surveys, all children of appropriate age in selected households are eligible for measurement regardless of whether their mother participated in the women’s questionnaire. There were 304,858 children eligible for measurements across the individual DHS included in this study (mean n across surveys = 7001 and SD = 4194). Age and sex were assessed by self-reports, and completion of reproductive calendars during household visits (used to determine age at first pregnancy and duration between pregnancies). Standard protocols in DHS instructed field investigators to weigh each child using a solar-powered digital scale (Seca 878) with a precision of ±100 g. Standing height was measured for children older than two years and lying length obtained in children less than two years old using an adjustable measuring board which is theoretically accurate to 1 millimeter [15]. In some early (phase 2–3) DHS, the age of eligibility ended at 35 months (n = 7 among the surveys included here).

MICS are large, standardized, multi-topic household surveys produced by UNICEF [16]. They tend to focus on reproductive health, maternal and child health interventions, child nutritional status, and early childhood development, and use similar methodology and measurement protocols to DHS. MICS also collect a standardized set of socioeconomic characteristics of individuals and households. Data-sets can be accessed in the public domain. The survey employs a two-stage cluster design and households are randomly selected without replacement from a listing of households within primary sample units (PSU). Children eligible for measurement are aged 0–59 months with some surveys restricted to 0-36 months. There were 232,124 children eligible for measurements in the MICS included in this study (mean n across surveys = 6775 and SD = 4439). Age and sex were assessed by self-reports and confirmed during field interviews. Similar to DHS, MICS used Seca digital scales to measure weight and standing/lying height/length was obtained via Shorr measuring boards.

NNS are rapid surveys conducted on a by-country basis (typically every two years, annually or bi-annually) and are not part of a standardized data collection program. They focus on assessing child and maternal nutrition indicators using SMART methodology, crude under-five mortality rates, and selected interventions (e.g. vitamin A supplementation and measles vaccination). As the NNS are not standardized across countries, they typically collect less extensive data on socioeconomic status and other characteristics. The information included may vary from country to country. Special permission is required from country governments to obtain access to these data, which were provided to the study authors via UNICEF West and Central Africa Regional Office (WCARO). Children aged less than 5 years are selected from a random/systematic sample of households without replacement within clusters. Many NNS do not have children aged 0–5 months in their samples, which are restricted to ≥6 months of age. There were 189,029 children eligible for measurements in the NNS included in this study (mean n across surveys = 8290 and SD = 5291). Age and sex were assessed by self-reports and verified by interviewers during household visits. The NNS height and weight protocol was similar to that of DHS and MICS, and used Seca 878 digital scales to measure weight and Shorr measuring boards (or equivalent locally made boards) to measure length (in children < 2 years or < 87 cm)/standing height.

This study also used information from six waves of the National Health and Nutrition Examination Survey (NHANES) as a comparison data-set. The chosen NHANES represents a stratified, multistage probability sample of the civilian, non-institutionalized US population from 2003–2010 [17,18]. There were 8890 eligible children aged 0–59 months in NHANES (mean n across surveys = 1482 and SD = 127). Age and sex were assessed by self-reports and verified by field interviewers. NHANES protocol instructed trained health technicians to collect data on weight to the nearest 0.1 kilogram, and stature, length, and circumference measurements to the nearest millimeter. NHANES includes two measurements. A third measurement is also triggered if there is observed deviation between the first two measurements that is beyond an acceptable range [2].

### Data quality indicators

Child age ratios were calculated across survey populations. The age ratio was defined as the number of children aged 6–29 months over the number of children aged 30–59 months. (Age ratios were not calculated for some DHS as the age of eligibility ended at 35 months.) If the sample has good coverage of all ages in the 6–59 month range, then the ratio should be close to 1.0. However, some variability may arise due to age recording error, lack of knowledge of the child’s birthday, and/or demographic changes and changes in mortality rates over time. In addition, information on the age distribution of children covering 0–59 months (in six-month intervals) was also calculated to examine departures from the expected distribution for under-five populations. The statistics for age ratios are based on original data quality score reports (according to SMART methods). It should be noted that there has been some disagreement in the literature about what the ideal age ratio should show given differences in the month intervals between 6–29 and 30–59 and changes in fertility and other variations which may arise. Therefore, formal statistical tests of these intervals were not conducted.

Child sex ratios were calculated, defined as the number of males to females in a population. In theory, this ratio should be 1.0. True ratios may deviate somewhat from this ideal, and some variability may arise due to sampling variation or recording error.

We created three categories of children according to the presence and validity of their height and weight data. The first category represents children who were targeted for measurement, but who could not be located for measurement or their mothers refused measurement. We refer to these children as having ‘missing’ data. The second category represents children with standardized anthropometric z-scores (based on age, sex, height, and weight) that were biologically implausible, which was defined as values that were five standard deviations above or below (for weight-for-height – WHZ), six standard deviations above or below (for height-for-age – HAZ), and five standard deviations above or six standard deviations below (for weight-for-age – WAZ) the mean (i.e. greater than or less than WHO 2006 defined norms) [19,20]. Children who had both height and weight data that were neither missing nor implausible were considered to have ‘valid’ anthropometric data. It is anticipated that these indicators of missing and implausible data will be lower in the NNS due to field use of laptops and ENA software that provides daily feedback to interviewers regarding missing and implausible values.

We chose to assess terminal digit preference as an indicator of interviewer performance because demographic methods have been developed that are able to parse random vs. non-random recoding of digits as an indication of data quality and interviewer performance. Thus, we calculated terminal digit preference scores (DPSs) for height and weight data using methods proposed by SMART and the WHO Monica blood pressure study, which involves a chi-square test of homogeneity of DPS [10,12]. As the *p*-values from a chi-square test of departure from a uniform distribution are generally very small when large sample sizes are used, such as in this study, the absolute values of DPSs were the present focus. The DPS varies between 0 and 100. Scores are low in instances of high agreement with the ideal of nonpreference of the terminal digits, whereas scores rise as deviations from a uniform distribution across the terminal digits 0 through 9 increase. Scores above 20 are indicative of a statistically significant preference detected for the terminal digit. We calculated the percentage of surveys within a survey source that had a DPS above 20 for height and weight, separately.

Using age, sex, height, and weight data, and the WHO Child Growth Standards [21], we transformed the height and weight data into standardized z-scores representing HAZ, WAZ, and WHZ. For each survey, we calculated the mean and standard deviation as well as estimates of skewness and kurtosis for HAZ, WAZ, and WHZ for children. Skewness is a measure of distribution symmetry or asymmetry. In general, a positive value for skewness indicates that the probability density function for a particular variable is longer and/or fatter on the right side; negative values indicate a distribution is longer and/or fatter on the left side. Kurtosis is an indication of peakedness and/or tail weight of a distribution. A perfectly symmetrical normal distribution would have a skewness of 0 and kurtosis of 3. These indicators were derived from an existing score card for nutrition data quality that we used as a basis for comparison. Although the distributions of z-scores may have a certain level of skew in situations with high prevalence of undernutrition (and this phenomenon has been observed among adults across countries of similar socioeconomic conditions), data quality issues may be observed if certain distributions are dramatically skewed beyond what would be expected.

Finally, a total data quality score was created using the aforementioned indicators according with weighting applied following the SMART criteria, which is one method available to review and assess anthropometric data quality [10–12]. The score represents a weighted combination of the level of missing and/or implausible data, overall sex ratio, overall age ratio, digit preference score for weight, digit preference score for height, standard deviation of WHZ, skewness of WHS, and kurtosis of WHZ. Weights and points for each measure were used to calculate a total data quality score with a maximum possible score of 90. Lower scores indicate higher-quality data.

### Analyses

We examined the number of children in each of the age categories against the expected values from a chi-squared distribution. A graphical assessment was also performed on the DHS data-set using all children irrespective of survivor status to examine the distribution of ages of children within sampled households. In the other surveys, information on deceased children was not available. The main analyses provided summary statistics (across surveys, but within survey source) using the various data quality indicators described previously (e.g. average and distribution of age and sex ratios, prevalence of valid, missing, and implausible data, and spread of DPSs, anthropometric z-scores, and data quality scores). Supplementary analyses provided similar information at the individual survey level, which is the national level for a given year.

## Results

Table 1 presents the mean age and sex ratios across the surveys from each survey source. The range of age ratios in MICS varied from 0.69 in Chad in 2010 to 1.11 in Gambia in 2005. The range of age ratios in the DHS varied from 0.82 in both Burkina Faso in 1993 and Cameroon in 1991 to 1.15 in Sierra Leone in 2008. The NNS age ratios varied from 0.84 in Cameroon in 2011 to 1.13 in Mauritania in 2009. Each survey source had several surveys where the age ratio differed from 1.0 though the direction of the ratio differed. The average age ratio for MICS and DHS was less than 1.0 with more than half of the surveys from each source exhibiting an age ratio of less than 0.95, indicating that fewer younger children (6–29 months) were measured as compared to children aged 35–59 months. The opposite was true for the NNS where 30% of surveys exhibited ratios greater than 1.05. The age ratio in NHANES was 1.27, indicating greater numbers of younger children surveyed. Plots of the age distributions are provided in Supplemental Figure 1. In several surveys, there was clear evidence of age heaping around or just after 12, 24, and 36 months of age.

The sex ratio among the MICS varied from 0.93 in Sao Tome et Principe in 2000 to 1.06 in Niger in 2000. The sex ratios among the DHS ranged from 0.94 in Senegal in 1992 to 1.11 in Guinea in 1999. Finally, the sex ratios varied from 0.99 in Sierra Leone in 2010 to 1.13 in Mauritania in 2008 in the NNS. Among the NHANES, the sex ratios varied from 1.02 to 1.18. Age and sex ratios for each individual survey within each survey source are presented in Supplemental Tables 2–4.

**Table 1.** Summary statistics for age and sex ratios from children aged 0–59 months across MICS, DHS, and NNS surveys in West Central African countries and NHANES in the US.

		Age ratio					Sex ratio				
		Mean ratio across surveys			Surveys with ratio < 0.95	Surveys with ratio > 1.05	Mean ratio across surveys			Surveys with ratio < 0.95	Surveys with ratio > 1.05
	N of surveys	Mean	SD	Range	n (and %)	N (and %)	Mean	SD	Range	n (and %)	n (and %)
MICS	28	0.90	0.10	0.69 to 1.11	20 (71%)	2 (7%)	1.01	0.03	0.93 to 1.06	1 (4%)	2 (7%)
DHS	38^a^	0.93	0.07	0.82 to 1.15	25 (67%)	2 (5%)	1.03	0.03	0.94 to 1.11	1 (2%)	11 (24%)
NNS	26^b^	1.01	0.08	0.84 to 1.13	4 (15%)	8 (31%)	1.04	0.03	0.99 to 1.13	0 (0%)	6 (22%)
NHANES	6	1.3	0.10	1.2 to 1.4	0 (0%)	6 (100%)	1.08	0.06	1.02 to 1.18	0 (0%)	2 (33%)

Notes: ^a^7 out of 45 DHS did not include children older than 35 months so the age ratio was not calculated for these surveys. ^b^1 out of 27 surveys did not include children older than 39 months.

DHS = Demographic and Health Surveys; MICS = Multiple Indicator Cluster Surveys; NNS = National Nutrition Surveys; NHANES = National Health and Nutrition Examination Survey. Age ratio was defined as the number of children aged 6–29 months over the number of children aged 30–59 months. Sex ratio was defined as the number of males to females in a population.

Table 2 presents basic statistics about the percentages of children with valid, missing, and implausible height and weight data across the surveys conducted by each survey source. The average percentage of children with valid data was 88% in MICS, 92% in DHS, 97% in NNS, and 93% in NHANES. The presence of valid data varied from 69% to 96% in the MICS, from 76% to 96% in the DHS, and from 88% to 100% in the NNS. Out of 28 MICS surveys, 46% of the surveys had 10% or more missing or implausible anthropometric data (among children who were targeted for measurement). Likewise, 20% of the DHS had 10% or more missing or implausible data. The NNS only had 2 out of 28 surveys with 10% or more missing or implausible data. The percentages of children who fall into these categories in each survey within each source are presented in Supplemental Tables 5–7.

**Table 2.** The percentage of children (aged 0–59 months) with valid, missing, or implausible height and weight data across surveys within the MICS, DHS, and NNS programs in West and Central African countries, and the NHANES program in the US.

	Percentage with valid data			Percentage with implausible data			Percentage with missing data			Percentage with implausible or missing data		
	Mean across surveys	SD	Range	Mean across surveys	SD	Range	Mean across surveys	SD	Range	Mean across surveys	SD	Range
MICS	87.6%	8.0 %-points	68.5% to 96.3%	4.5%	3.2 %- points	0.4% to 12.1%	7.9%	5.7 %-points	0.6% to 21.5%	12.4%	8.0 %-points	3.7% to 31.5%
DHS	92.0%	4.6 %-points	76.3% to 95.6%	4.1%	3.2 %-points	2.1% to 23.7%	3.0%	2.2 %-points	0.6% to 8.7%	8.0%	4.6 %-points	2.1% to 23.7%
NNS	96.8%	3.2 %-points	88.3% to 100%	0.5%	0.8 %-points	0% to 4.1%	2.7%	3.2 %-points	0% to 11.1%	3.2%	3.2 %-points	0% to 11.7%
NHANES	92.9%	6.9 %-points	78.9% to 96.1%	0.4%	0.2 %-points	0.2% to 0.6%	6.7%	7.0 %-points	3.3% to 20.9%	7.1%	6.9 %-points	3.9% to 21.1%

Notes: DHS = Demographic and Health Surveys; MICS = Multiple Indicator Cluster Surveys; NNS = National Nutrition Surveys; NHANES = National Health and Nutrition Examination Survey. There was a total of 232,124 eligible children across 28 MICS (mean n across surveys = 6775 and SD = 4439); a total of 304,858 eligible children across 45 DHS (mean n across surveys = 7001 and SD = 4194); a total of 189,029 eligible children across 27 NNS (mean n across surveys = 8290 and SD = 5291); and a total of 8890 eligible children across 6 waves of NHANES (mean n across surveys = 1482 and SD = 127).

Summary statistics regarding terminal DPSs for height and weight data are presented in Table 3. The average height DPS was 5.9 in NNS, 20.6 in DHS, and 31.6 in MICS while the average weight DPS was 1.7 in NNS, 5.4 in DHS, and 9.8 in MICS. In comparison, the DPSs for height and weight in NHANES were 2.6 and 2.9, respectively. None of the NNS surveys had height DPSs above 20 whereas 44% and 61% of surveys in DHS and MICS, respectively, had height DPSs above 20. Similarly, 7% and 14% of surveys in DHS and MICS, respectively, had weight DPSs above 20 while no NNS surveys did. Information on the DPSs within each survey is presented in Supplemental Tables 8–10.

**Table 3.** Summary statistics for terminal digit preference score (DPS) for height and weight data from children aged 0–59 months in MICS, DHS, and NNS surveys in West and Central African countries, and from the NHANES program in the US.

	Mean height DPS across surveys			Surveys with meanheight DPS above 20	Mean weight DPS across surveys			Surveys with meanweight DPS above 20
	Mean	SD	Range	n and %	Mean	SD	Range	n and %
MICS	31.6	23.6	4.2 to 92.4	17 (61%)	9.8	15.4	1.2 to 71.2	4 (14%)
DHS	20.6	14.8	6.7 to 91.8	20 (44%)	5.4	12.1	20.7 to 80.3	3 (7%)
NNS	5.9	4.2	2.0 to 19.7	0 (0%)	1.7	0.7	0.6 to 3.2	0 (0%)
NHANES	2.6	1.1	1.6 to 4.0	0 (0%)	2.9	0.5	2.1 to 3.5	0 (0%)

Notes: DHS = Demographic and Health Surveys; MICS = Multiple Indicator Cluster Surveys; NNS = National Nutrition Surveys; NHANES = National Health and Nutrition Examination Survey. There were 28 MICS covering 16 countries, 45 DHS covering 19 countries, and 27 NNS covering 13 countries. Scores above 20 are indicative of a statistically significant preference detected for the terminal digit.

Means and standard deviations (SDs) of the mean z-score, SD, skewness, and kurtosis for HAZ, WAZ, and WHZ are presented across surveys by survey source (Table 4). The average SDs of the mean HAZ, WAZ, and WHZ scores across the NNS surveys were lower than the mean SDs for these indicators across surveys in the DHS and MICS and closer to the means for NHANES of 1.12 (HAZ), 1.07 (WAZ), and 1.06 (WHZ). The average skewness for HAZ across surveys within each source was positive while the average skewness was slightly negative for WAZ and WHZ across survey sources. Supplemental Tables 11–13 present the mean z-score and SD for the HAZ, WAZ, and WHZ indicators for each survey within each survey source. Skewness and kurtosis statistics are also provided.

**Table 4.** Summary statistics across surveys within the MICS, DHS, and NNS survey programs, separately, for the average height-for-age (HAZ), weight-for-age (WAZ), and weight-for-height (WHZ) z-scores (based on the WHO 2006 reference standard across surveys) from children aged 0–59 months in West Central African countries, and from US children in the NHANES program.

	Mean of mean z-scores across surveys			Standard deviation of mean z-scores across surveys		
	HAZ	WAZ	WHZ	HAZ	WAZ	WHZ
MICS	−1.46	−1.06	−0.26	0.21	0.25	0.34
DHS	−1.40	−1.08	−0.35	0.24	0.33	0.33
NNS	−1.35	−1.14	−0.54	0.22	0.18	0.26
NHANES	0.06	0.44	0.57	0.05	0.04	0.04
	Mean standard deviation of z-scores across surveys			Standard deviation of mean standard deviation of z-scores across surveys		
	HAZ	WAZ	WHZ	HAZ	WAZ	WHZ
MICS	1.82	1.40	1.45	0.24	0.16	0.22
DHS	1.80	1.39	1.44	0.20	0.14	0.19
NNS	1.36	1.13	1.11	0.13	0.10	0.06
NHANES	1.12	1.07	1.06	0.04	0.03	0.03
	Mean z-score skewness across surveys			Standard deviation of z-score skewness across surveys		
	HAZ	WAZ	WHZ	HAZ	WAZ	WHZ
MICS	0.39	−0.04	−0.07	0.13	0.10	0.09
DHS	0.37	−0.01	−0.03	0.13	0.14	0.14
NNS	0.23	−0.11	−0.04	0.16	0.14	0.12
NHANES	0.08	0.11	0.25	0.03	0.03	0.08
	Mean z-score kurtosis across surveys			Mean standard deviation of z-score kurtosis across surveys		
	HAZ	WAZ	WHZ	HAZ	WAZ	WHZ
MICS	3.78	3.79	3.68	0.34	0.38	0.33
DHS	3.79	3.80	3.83	0.36	0.37	0.40
NNS	3.93	3.64	3.69	0.57	0.39	0.32
NHANES	3.85	3.85	3.71	0.52	0.31	0.18

Notes: DHS = Demographic and Health Surveys; MICS = Multiple Indicator Cluster Surveys; NNS = National Nutrition Surveys; NHANES = National Health and Nutrition Examination Survey. There were 28 MICS covering 16 countries, 45 DHS covering 19 countries, and 27 NNS covering 13 countries.

Information on the overall data quality scores is presented in Table 5. The MICS had a mean data quality score of 40.9 with a range from 18 in Central African Republic in 2010 to 61 in Central African Republic in 2000. The average data quality score across the DHS was 33, with a range from 11 in Congo in 2012 to 70 in Benin in 2011. The average data quality score in the NNS was 14.0, with a range from a low score of 4 in Mauritania in July 2011 to a score of 35 in Cameroon in 2011. One third of the NNS had a data quality score below 10 (n = 9), with 14/27 (52%) falling between scores of 10 and 19. For comparison, the average data quality score in NHANES was 24.3 with a range from 16 to 38, and 4/45 (9%) falling in the 10–19 range. Supplemental Tables 14–16 present equivalent data by regions within countries for each of the surveys within each of the three survey sources. Appendices A–L present all of the major analyses at the sub-national region (domain) level within countries.

**Table 5.** Summary statistics of mean data quality scores (lower = worse) for anthropometric data from children aged 0–59 months across MICS, DHS, and NNS surveys in West Central African countries, and NHANES in the US.

	Mean data quality score across surveys			Surveys with a data quality score of less than 10	Surveys with a data quality score from 10–19
	Mean	SD	Range	n (%)	n (%)
MICS	40.9	13.5	18 to 61	0 (0%)	1 (4%)
DHS	33.0	12.7	11 to 70	0 (0%)	4 (9%)
NNS	14.0	8.0	4 to 35	9 (33%)	14 (52%)
NHANES	24.3	7.5	16 to 38	0 (0%)	1 (17%)

Notes: DHS = Demographic and Health Surveys; MICS = Multiple Indicator Cluster Surveys; NNS = National Nutrition Surveys; NHANES = National Health and Nutrition Examination Survey. Lower scores indicate higher quality of data. The potential range of scores is from 0 to 90.

## Discussion

This study presents a comprehensive assessment of the quality of child anthropometric data in 100 surveys conducted in the West Central Africa region. The most salient findings suggest that (1) there is an unequal distribution in the age of children being recorded and measured, (2) there is a substantial amount of missing or implausible anthropometric data across surveys in the DHS and MICS, (3) there is definite evidence of terminal digit preference for height data, and (4) anthropometric data quality was highly variable both between and within survey sources and over time, on average.

The variation in age ratios and age distribution as well as in levels of missing and implausible data may be due to variation in child eligibility, interviewer training across the surveys, and data collection protocols. In addition, the prevalence of missing and implausible data may be lower in the NNS because the NNS is a narrow-topic survey and teams are dedicated to anthropometric indicators. Moreover, the NNS use specialized software to collect and flag data while interviewers are in the field, which could then permit implausible values to be flagged and corrected. It has been suggested, however, that this process itself may generate data which are ‘in range’ but of poor quality if data are modified on the fly in order to satisfy software range checks without remeasurement of the child. In the multi-topic surveys (DHS, MICS), the level of missing data among anthropometric variables exceeded that of other types of variables (e.g. socioeconomic status), illustrating the increased complexity in gathering such data [7]. Critically, the NNS does not record a complete household listing of all members in all surveys. In the DHS and MICS, all children are recorded, making it possible to view who is measured and who is not measured. In the NNS, however, there is no household roster . Information on children who were not part of the measurement sample is not captured. Therefore, it is not clear if the complete denominator of eligible children for whom anthropometric data could have been collected was recorded. Although efforts are made to revisit households to ensure complete follow-up of those children, the omission of any children with missing anthropometric data from the NNS data-set may artificially lower the proportion of missing data.

Increased terminal digit preference for recorded heights is likely related to the larger number of possible values for height. The interval of 50 to 110 cm (including nearly all accepted height values) covers 600 unique values compared to 170 unique values across the interval of 3.0–19.9 kg for weight. Separately, although there was sizeable variation in the distribution of the anthropometric z-scores, in particular as indicated by the SD of WHZ, it is not clear what the ideal parameters may be and whether the variability may decrease as the nutritional status of children in the population improves. Moreover, many of the NNS were conducted from 2010 and onward, whereas some of the DHS and MICS surveys are much older. It is likely that nutritional improvements were made during the time gap. For example, the mean WHZ for Ghana was −0.56 in 1993. That score improved to −0.31 by 2008, although the SD remained relatively stable, decreasing from 1.422 to 1.416.

Finally, although the data quality score is a useful starting point for comparison across multiple surveys, issues remain with some of the score components. First, the score gives relatively large weight to missing and/or implausible values. If that component of the data quality score is removed, then the NNS has an overall mean score of 10.1 across surveys (versus 33.0), the DHS has a mean score of 22.6 (versus 33) across surveys, and the MICS has a mean score of 27.1 (versus 40.9) across surveys. Second, there is lack of consensus around the ‘true’ population parameter for the age ratio given that a comparison is being made between groups spanning an unequal number of months (6–29 months versus 30–59 months). Age ratios may be sensitive within countries to demographic changes from changing fertility rates and rates of infant and child mortality. In addition, many NNS surveys do not cover children in the range of 0–5 months (ages which may be more difficult to assess). It is not clear if this ratio should be 1.0 or some other value and if the true value may vary between countries and over time. Although derivation of the data quality score is subjective as different weight values are applied to each parameter making up the overall score are not empirically derived, the data quality score is based on key parameters which literature has shown to be important for the assessment of anthropometric data quality. Further work is needed to explore the measurement properties of this score along with its performance across nutrition data-sets of various types and sample sizes. Critically, the statistics for data quality assessment are still in their infancy. More work is needed to improve the ability to assess and determine the presence of poor data quality given complexities and differences across survey platforms.

There are several limitations to this study. First, it was not possible to assess the impact of non-normality and age misclassification on the prevalence of stunting, underweight, and wasting. Future research should consider conducting a simulation analysis where different values for mean, SD, skewness, and kurtosis are used to simulate the prevalence of stunting, underweight, and wasting under different data quality assumptions. This exercise would help determine the extent to which inaccuracies in each parameter (e.g. skewness) result in the greatest change in the estimates of undernutrition. Doing so would inform future iterations of the data quality score where the weight values can be adjusted to represent the most important parameters. For instance, our preliminary simulation work conducted with a sample of DHS and MICS data-sets to induce heaping/digit preference in age distributions found that inaccuracies in age could result in a 4.5% over-estimation in the prevalence of stunting and a 4.2% overestimation in the prevalence of underweight, while inaccuracies in weight at the level of 0.1 kg could result in a 2% over-estimation of prevalence of underweight or wasting. Second, further work is needed to ensure that robust data quality scores can be derived at lower levels of aggregation despite large variations in sample sizes. Third, although we did not explicitly model variation across interviewers, we addressed this by examining terminal digit preference as one proxy for interviewer-caused variation. In addition, our supplemental analyses by sub-national regions within countries are implicitly related to interviewer performance because field teams are assigned to specific clusters and regions within countries during fieldwork. The literature suggests that interviewer variation is present in many DHS, particularly for anthropometric data [7,22]. Future analyses could incorporate this issue into the overall data quality score. Examination of sub-national estimates may also identify regions with poor data quality which may need to be excluded from certain analyses or prevalence calculations.

Finally, this study did not attempt to make direct comparisons between survey programs as many methodological and sampling differences exist between them, which may indirectly impact the quality of the nutrition data. Moreover, survey performance may be related to implementing agency, funding, or a country's political situation, which would not be uniform across a survey program. For example, anthropometric data for Benin 2011 (DHS) was identified as low quality in the final DHS survey report [23]. Indeed, this particular survey was one of the worst-performing surveys according to our metrics, suggesting that surveys with similar quality scores (> 70) should be flagged prior to analyses. Regional estimates, however, may be valid if data quality scores are improved at the sub-national level. In addition, countries such as Congo, Cameroon, Togo, and Central Africa Republic seem to have some consistency in lower overall quality scores regardless of program, perhaps due to instability or other challenges in operating a large survey program within the country. Moreover, for countries with multiple surveys, there were no patterns over time in data quality scores. Instead, data quality varied from year to year. Such variability may be due to the difficulty inherent in collecting anthropometric measurements, that the data quality assessment itself is not perfect, or that nutritional status in the population may be changing, and making it a moving target. Thus, continuous training in, and monitoring of, data collection are needed to ensure the highest possible quality of anthropometric data given these inherent challenges.

Despite these limitations, this study has several strengths. First, we included 100 surveys for data assessment and provided a detailed explanation of how data were collected by the associated survey program, obtained by the authors, and analysed. Second, we conducted many sub-national analyses and applied data quality scores to all available regions within countries. These analyses will enable researchers using these data to be aware of potential data quality issues at the national and regional levels. Third, we combined several methods from leading contributors in the fields of data quality and anthropometric assessment to create a balanced and comprehensive data quality score. The score itself is not a panacea but can be a starting point for comparisons between surveys and to further emphasize the need for continued monitoring, training, and improvements in anthropometric data collection in field surveys in low-income settings.

Our recommendations for improvement in data quality include continuous fieldworker training and monitoring, streamlining and monitoring of data processing, use of high-quality measurement equipment designed for survey settings, and overall simplification of data collection processes to reduce both interviewer and respondent  burden [7,22]. Another suggestion is to reduce the number of values recorded for height, by measuring to the nearest fifth or half of a centimeter, without substantially impacting data quality [7]. In addition, range checks and calculation of z-scores could be implemented during fieldwork and before leaving a particular village by team supervisors who could monitor the need for child reassessment. Such procedures would allow for early identification of problematic data, thus helping data collection teams revisit households where problems exist. Finally, implementing SMART/ENA methods to review data quality during the collection process and during analyses might help to improve both collection and interpretation of anthropometric data in resource-limited settings.

## Conclusions

These survey programs provide key information on the nutritional status of young children across countries. Stunting and underweight indicators are commonly used in comparative analyses and in the assessment of progress toward international development goals. Therefore, a reasonable concern is that their anthropometric data be of the highest possible quality. In addition, pairing socioeconomic information with anthropometric data is also critically important to show inequality in outcomes and understand which sub-populations are most affected by the burden of child malnutrition [24–31]. Moreover, comparing the distribution of malnutrition between countries requires that surveys in each country collect the same data. At present, however, it is not possible to examine nutritional status along many socioeconomic factors or other dimensions in many of the NNS, although stratification by region is possible in all survey sources. Further, comparison of anthropometric data between countries according to non-anthropometric information is not possible when using NNS. In contexts where chronic nutritional deprivation is a key burden, the benefits and richness of MICS and DHS outweigh any perceived weakness in terms of greater variability in data quality. Thus, when identifying a data-set and survey source from which to obtain child anthropometric data, users should weigh which survey characteristics are relevant for their work, including whether data are publicly available, the extent of standardized socioeconomic and other information collected, the population sample, and the data quality. Overall, this study suggests that errors in anthropometric measures are both commonplace and difficult to isolate. Thus, assessment of data quality before, during, and after data collection is needed.

## Supplementary Material

Appendices A-L

Supplementary Material

## Paper context

The measurement of anthropometric (height and weight) data in young children is routinely done in large population surveys to assess and monitor undernutrition in low-resource settings. Important differences often arise in the results of these surveys within countries, across survey agencies, and over time. Recent efforts have been made to develop quality standards and assessments of anthropometric data in these contexts. This study describes and compares survey characteristics and indicators of quality in 100 surveys from 23 countries in West Central Africa.
