# Child anthropometry data quality from Demographic and Health Surveys, Multiple Indicator Cluster Surveys, and National Nutrition Surveys in the West Central Africa region: are we comparing apples and oranges?

**DOI:** 10.1080/16549716.2018.1444115

**Published:** 2018-03-06

**Authors:** Daniel J. Corsi, Jessica M. Perkins, S. V. Subramanian

**Affiliations:** a OMNI Research Group, Ottawa Hospital Research Institute, Ottawa, ON, Canada; b Center for Population & Development Studies, Harvard School of Public Health, Cambridge, MA, USA; c MGH Global Health, Massachusetts General Hospital, Boston, MA, USA; d Department of Social and Behavioral Sciences, Harvard T.H. Chan School of Public Health, Boston, MA, USA

*Global Health Action*, 10(1)

https://doi.org/10.1080/16549716.2017.1328185

On page 6 of the published article, there is an error in the heading for Table 5. The words “(lower = worse)” should not be present and the correct heading is displayed below:

Table 5. Summary statistics of mean data quality scores for anthropometric data from children aged 0–59 months across MICS, DHS, and NNS surveys in West Central African countries, and NHANES in the US.

