# Supplementary material for: Child anthropometry data quality from Demographic and Health Surveys, Multiple Indicator Cluster Surveys, and National Nutrition Surveys in the West Central Africa region: are we comparing apples and oranges?
Source: Glob Health Action. 2017 Jun 22;10(1):1328185. doi: 10.1080/16549716.2017.1328185 (PMC5496063; doi:10.1080/16549716.2017.1328185)
Supplement: Appendices A-L [file zgha_a_1328185_sm3411.pdf]

Appendix A

Region (domain-level) sample sizes by country and survey year, DHS

Benin 2001	No.
atacora	700
atlantique	1008
borgou	836
mono	635
oueme	718
zou	832
Total	4729

Benin 2006	No.
alibori	1224
atacora	1385
atlantique	1626
borgou	1510
collines	1002
couffo	1233
donga	773
littoral	975
mono	973
plateau	735
quémé	1725
zou	1521
Total	14682

Benin 2011	No.
Alibori	935
Atacora	1318
Atlantique	1484
Borgou	1082
Collines	856
Couffo	961
Donga	701
Littoral	1036
Mono	750
oueme	1490
Plateau	804
Zou	1262
Total	12679

Burkina Faso 1993	No.
central/south	1150
east	941
north	784
ouagadougou	925

west	1275
Total	5075

Burkina Faso 1998	No.
central/south	1280
east	1407
north	694
ouagadougou	499
west	1192
Total	5072

Burkina Faso 2003	No.
boucle de mouhoun	711
cascades	621
centre (sans ouagadougou)	288
centre-est	630
centre-nord	778
centre-ouest	928
centre-sud	603
est	678
hauts bassins	776
nord	713
ouagadougou	276
plateau central	858
sahel	624
sud-ouest	868
Total	9352

Burkina Faso 2010	No.
boucle de mouhoun	1244
cascades	855
centre	839
centre-est	1090
centre-nord	1047
centre-ouest	1222
centre-sud	876
est	1357
hauts basins	1143
nord	1085
plateau central	938
sahel	1085
sud-ouest	935
Total	13716

Cameroon 1991	No.
adam/nord/ext-nord	910
centre/sud/est	496
nord-ouest/sud-ouest	406
ouest/littoral	449
yaoundé/douala	777
Total	3038

Cameroon 1998	No.
central, south, & east	620
north/ extreme north/ ad	699
northwest & southwest	334
west & littoral	433
Total	2086

Cameroon 2011	No.
adamaoua	960
centre	949
douala	745
est	766
extrême-nord	1547
littoral	540
nord	1373
nord-ouest	938
ouest	992
sud	624
sud-ouest	622
yaoundé	678
Total	10734

Central African Rep 1994	No.
bangui	527
rs i	415
rs ii	443
rs iii	484
rs iv	351
rs v	337
Total	2557

Chad 1996	No.
b.e.t.	56
batha	252
biltine	148
chari-baguirmi	688
guéra	236
kanem	234
lac	225
logone occidental	422
logone oriental	415
mayo-kebbi	656
moyen chari	689
n'djaména	1071
ouaddaï	569
salamat	225
tandjilé	452
Total	6338

Chad 2004	No.

b. e. t.	513
bar azoum	440
centre est	424
chari baguirmi	504
logone occidental	608
mayo kebbi	559
moyen chari	465
n'djaména	1004
ouaddai est	409
Total	4926

**Congo 2005**	**No.**
brazzaville	1081
nord	1038
pointe noire	901
sud	1415
Total	4435

**Congo 2012**	**No.**
bouenza	794
brazzaville	684
cuvette	666
cuvette - ouest	587
kouilou	851
lekoumou	639
likouala	912
niari	788
plateaux	681
pointe-noire	802
pool	891
sangha	562
Total	8857

**Congo DR 2007**	**No.**
bandundu	734
bas-congo	590
equateur	789
kasaï occident	726
kasaï oriental	826
katanga	766
kinshasa	852
maniema	736
nord-kivu	691
orientale	567
sud-kivu	710
Total	7987

**Côte d'Ivoire 1994**	**No.**
center	341
center east	147
center north	331

center west	598
north	254
north east	192
north west	216
south	968
south west	251
west	360
Total	3658

**Côte d'Ivoire 1998**	**No.**
capital (abidjan)	476
countryside	691
small city	536
Total	1703

**Côte d'Ivoire 2011**	**No.**
Centre	582
Centre-Est	559
Centre-Nord	724
Centre-Ouest	679
Nord	671
Nord-Ouest	956
Nord-est	572
Ouest	672
Sud sans Abidjan	486
Sud-ouest	604
Ville d'Abidjan	588
Total	7093

**Gabon 2000**	**No.**
east (haut-ogooué & ogooué-lolo)	939
libreville,port-gentil	1007
north (ogooué-ivindo & woleu-ntem)	721
south (ngounié, nyanga)	770
west (estuaire, moyen-ogooué & ogooué-maritime)	663
Total	4100

**Gabon 2012**	**No.**
estuaire	506
haut-ogooué	622
libreville-port-gentil	797
moyen-ogooué	441
ngounié	663
nyanga	532
ogooué maritime	373
ogooué-ivindo	876
ogooué-lolo	534
woleu-ntem	403
Total	5747

**Ghana 1993**	**No.**

ashanti	379
brong-ahafo	200
central	213
eastern	222
greater accra	188
northern	227
upper east	143
upper west	71
volta	226
western	187
Total	2056

**Ghana 1998**	**No.**
ashanti region	420
brong ahafo region	202
central region	271
eastern region	347
greater accra region	267
northern region	304
upper east region	343
upper west region	261
volta region	267
western region	333
Total	3015

**Ghana 2003**	**No.**
ashanti	529
brong ahafo	424
central	221
eastern	294
greater accra	317
northern	569
upper east	279
upper west	330
volta	248
western	319
Total	3530

**Ghana 2008**	**No.**
ashanti	412
brong ahafo	254
central	208
eastern	245
greater accra	264
northern	432
upper east	217
upper west	264
volta	238
western	260
Total	2794

Guinea 1999	No.
central guinea	860
conakry	704
forest guinea	1337
lower guinea	1049
upper guinea	1016
Total	4966

Guinea 2005	No.
boké	724
conakry	460
faranah	743
kankan	892
kindia	833
labé	578
mamou	554
n'zérékoré	832
Total	5616

Guinea 2012	No.
Bok?	631
Conakry	728
Faranah	995
Kankan	1052
Kindia	747
Lab?	749
Mamou	688
N'Z?r?kor?	834
Total	6424

Liberia 2007	No.
monrovia	893
north central	1089
north western	671
south central	844
south eastern a	780
south eastern b	1028
Total	5305

Mali 1995	No.
bamako	532
gao	274
kayes	801
koulikoro	956
mopti	578
sikasso	954
ségou	861
timbuktu	262
Total	5218

Mali 2001	No.

bamako	1199
gao	433
kayes	1619
kidal	159
koulikoro	1768
mopti	1424
segou	1527
sikasso	2097
tombouctou	393
Total	10619

**Mali 2006**	**No.**
bamako	1318
gao	862
kayes	1562
kidal	286
koulikoro	1638
mopti	1951
segou	1491
sikasso	2456
tombouctou	873
Total	12437

**Niger 1992**	**No.**
agadez	213
diffa	139
dosso	647
maradi	969
niamey	989
tahoua	925
tillabéri	753
zinder	917
Total	5552

**Niger 1998**	**No.**
dosso	609
maradi	1023
niamey	413
tahoua/agadez	712
tillaberi	765
zinda/diffa	704
Total	4226

**Niger 2006**	**No.**
agadez	683
diffa	723
dosso	1284
maradi	1268
niamey	873
tahoua	1296
tillabéri	1120

zinder	962
Total	8209

Niger 2012	No.
Agadez	579
Diffa	1035
Dosso	1580
Maradi	2372
Niamey	959
Tahoua	1899
Tillaberi	1485
Zinder	1693
Total	11602

Nigeria 1990	No.
northeast	1654
northwest	1542
southeast	1780
southwest	1811
Total	6787

Nigeria 2003	No.
north central	875
north east	1159
north west	1475
south east	458
south south	471
south west	570
Total	5008

Nigeria 2008	No.
north central	4542
north east	5737
north west	6899
south east	2173
south south	2997
south west	3098
Total	25446

Sao Tome et Principe 2008	No.
região centro	572
região do principe	294
região norte	513
região sul	472
Total	1851

Senegal 1992	No.
central	1903
north east	868
south	610
west	1687

Total	5068

Senegal 2005	No.
dakar	727
diourbel	1072
fatick	886
kaolack	1192
kolda	1002
louga	920
matam	882
saint-louis	791
tambacounda	950
thiès	1031
zuguinchor	573
Total	10026

Senegal 2010	No.
dakar	759
diourbel	1008
fatick	854
kaffrine	894
kaolack	1120
kedougou	397
kolda	964
louga	843
matam	782
saint-louis	768
sedhiou	884
tambacounda	912
thiès	879
ziguinchor	569
Total	11633

Sierra Leone 2008	No.
eastern	1352
northern	1668
southern	1212
western	811
Total	5043

Togo 1998	No.
centrale	608
kara	609
lomé	322
marities	517
plateaux	803
savanes	1004
Total	3863

Region (domain-level) sample sizes by country and survey year, NNS

Benin 2008	No.
alibori	403
atacora	368
atlantique	309
borgou	334
collines	261
couffo	398
donga	262
littoral	223
mono	246
oueme	271
plateau	235
zou	242
Total	3,552

Burkina Faso 2012	No.
bales	775
bam	841
banwa	623
banwa ganzourgou	856
bazega	598
boulgou	689
cascades	644
centre ouest	582
est	922
houet	491
kadiogo	441
kenedougou	753
kossi	756
koulpelogo	834
kouritenga	708
kourweogo	660
mouhoun	641
nahouri	498
namentenga	881
nayala	680
nord	731
oubritenga	670
sahel	851
sanmentenga	862
sourou	660
sud ouest	513
tuy	565
zoundweogo	629
Total	19,354

Cameroon 2011	No.
extrême-nord	720
nord	761
Total	1,481

Central African Rep 2012	No.
bamingui bangoran	918
bangui	812
basse kotto	946
haut kotto	974
haut mbomou	950
kemo	933
lobaye	1,091
mambere kadei	1,299
mbomou	983
nana grebizi	1,073
nana mambere	657
ombella mpoko	891
ouaka	824
ouham	998
ouham pende	799
sangha mbarere	1,083
vakaga	510
Total	15,741

Chad June 2012	No.
Barh El Ghazal	750
Batha	748
Guéra	785
Hadjer Lamis	633
Kanem	599
Lac	570
N'Djamena	800
Ouaddai	602
Salamat	778
Sila	768
Wadi Fira	744
Total	7,777

Chad (7 regions) Dec/Jan 2012-13	No.
Logone Occidental	830
Logone Oriental	835
Mandoul	813
Mayo-Kebbi Est	1,023
Mayo-Kebbi Ouest	673
Moyen-Chari	720
Tandjilé	905
Total	5,799

The Gambia 2012	No.

banjul	745
basse	1,332
brikama	855
janjanburay	1,013
kanifing	764
kerewan	1,114
kuntaur	1,112
mansakonko	844
Total	7,779

Guinea-Bissau 2008	No.
Capitale	445
Est (Bafata e Gabu)	836
Nord (Biombo, Cacheu e Oio)	752
Sud (Bolama, Quinara e Tombali)	685
Total	2,718

Guinée Conakay 2012	No.
boke nord	891
boke sud	1,108
conakry	855
faranah	1,083
kankan	887
kindia	1,132
labe	700
mamou	844
nzerekore	709
Total	8,209

Liberia 2010	No.
bomi	334
bong	477
gbarpolu	369
grand bassa	405
grand cape mount	500
grand gedeh	443
grand kru	450
lofa	481
margibi	385
maryland	373
montserrado	350
nimba	441
river gee	422
rivercess	468
rural montserrado	421
sinoe	487
Total	6,806

Liberia 2011	No.
North Central	269
North Western	93

South Central	1,002
South Eastern	120
Total	1,484

**Mali 2011**	**No.**
bamako	1,273
gao	1,133
kayes	1,085
kidal	233
koulikoro	899
mopti	1,020
segou	676
sikasso	1,232
tombouctou	680
Total	8,231

**Mauritania 2006**	**No.**
Centre	417
Fleuve	849
Nord	230
Nouakchott	1,857
SudEst	528
Total	3,881

**Mauritania March 2008**	**No.**
Centre	953
Fleuve Nord	1,330
Fleuve Sud	1,012
Nord	493
Nouakchott	1,375
SudEst	1,222
Total	6,385

**Mauritania Dec 2008**	**No.**
Centre	959
Nord	710
Nouakchott A	804
Nouakchott B	927
Sud	1,072
SudEst	989
Trarza	881
Total	6,342

**Mauritania 2009**	**No.**
Centre	638
Nord	688
Nouakchott	679
Sud	965
Sud-est	699
Trarza	709
Total	4,378

Mauritania July 2010	No.
adrar/inchiri tiris	441
assaba	755
brakna	576
gorgol	723
guidimakha	632
hodh chargui	485
hodh gharbi	531
nouadhibou	505
nouakchott	505
tagant	646
trarza	584
Total	6,383

Mauritania Dec 2010	No.
adrar/inchiri tiris	408
assaba	556
brakna	567
gorgol	553
guidimakha	600
hodh chargui	471
hodh gharbi	551
nouadhibou	563
nouakchott	517
tagant	589
trarza	476
Total	5,851

Mauritania July 2011	No.
adrar/inchiri tiris	678
assaba	706
brakna	743
gorgol	680
guidimakha	769
hodh chargui	647
hodh gharbi	777
nouadhibou	674
nouakchott	645
tagant	742
trarza	719
Total	7,780

Mauritania Dec 2011	No.
adrar/inchiri tiris	690
assaba	763
brakna	701
gorgol	791
guidimakha	855
hodh chargui	628
hodh gharbi	803

nouadhibou	716
nouakchott	711
tagant	789
trarza	726
Total	8,173

Mauritania July 2012	No.
adrar/inchiri tiris	451
assaba	640
brakna	573
gorgol	610
guidimakha	856
hodh chargui	670
hodh gharbi	742
nouadhibou	474
nouakchott	590
tagant	755
trarza	456
Total	6,817

Niger 2012	No.
agadez	1,223
diffa	1,311
dosso	1,123
maradi	1,093
niamey	1,173
tahoua	1,469
tillabéri	895
zinder	939
Total	9,226

Nigeria (Northern States) 2011	No.
jigawa	954
kano	917
katsina	1,003
kebbi	1,021
sokoto	945
yobe	1,074
zamfara	995
Total	6,909

Senegal 2012	No.
dakar	487
kolda	552
matam	1,948
myf	726
sedhiou	2,212
tambacounda	3,114
velingara	676
Total	9,715

Sierra Leone 2010	No.
eastern	2,706
northern	5,043
southern	3,987
western	2,281
Total	14,017

Togo June 2012	No.
Centrale	417
Kara	612
Lomé	335
Maritime	479
Plateaux	372
Savanes	565
Total	2,780

Togo Dec 2012	No.
Kara	649
Savanes	802
Total	1,451

Region (domain-level) sample sizes by country and survey year, MICS

Burkina Faso 2006	No.
Boucle du Mouhoun	415
Cascade	627
Centre	519
Centre-Est	333
Centre-Nord	432
Centre-Ouest	531
Centre-Sud	366
Est	481
Hauts-Bassins	336
Nord	484
Plateau-Central	456
Sahel	381
Sud-Ouest	316
Total	5677

Cameroon 2006	No.
Adamaoua	649
Centre	560
Douala	385
Est	674
Extreme Nord	747
Littoral	426
Nord	815
Nord Ouest	383
Ouest	547
Sud	458
Sud Ouest	382
Yaounde	469
Total	6495

Central African Rep 2000	No.
Bamingui-Bangoran	465
Bangui	1620
Basse-Kotto	1099
Haut-Mbomou	378
Haute-Kotto	790
Kémo	1031
Lobaye	1122
Mambéré-Kadeï	755
Mbomou	899
Nana-Grébizi	1027
Nana-Mambéré	536
Ombella-M'poko	1094
Ouaka	987

Ouham	624
Ouham-Pendé	556
Sangha-Mbaéré	704
Vakaga	613
Total	####

Central African Rep 2006	No.
“Baminigui Bangoran”	577
“Bangui”	754
“Basse Kotto”	484
“Haut Mbomou”	364
“Haute Kotto”	1077
“Lobaye”	657
“Mambere Kadei”	528
“Mbomou”	556
“Nana Mambere”	1271
“Ombella Mpoko”	644
“Ouaka”	675
“Ouham Pende”	629
“Ouham”	688
“Sangha Mbaere”	916
Total	9820

Central African Rep 2010	No.
Baminigui Bangoran	427
Bangui	717
Basse Kotto	698
Haut Mbomou	373
Haute-Kotto	662
Kémo	846
Lobaye	890
Mambere Kadei	749
Mbomou	612
Nana Grebizi	696
Nana Mambéré	605
Ombella Mpoko	784
Ouaka	571
Ouham	685
Ouham Pende	710
Sangha Mbaere	821
Vakaga	58
Total	####

Chad 2000	No.
Autres villes	2000
N'Djaména	846
Rural	2538
Total	5384

Chad 2010	No.
Barh El Gazal	753

Bhata	948
Chari Baguirmi	795
Guéra	1462
Hadjer Lamis	837
Kanem	710
Lac	716
Logone Occidental	747
Logone Oriental	934
Mandoul	880
Mayo Kebbi Est	1219
Mayo Kebbi Ouest	910
Moyen Chari	736
Ndjaména	983
Ouaddai	922
Salamat	864
Sila	933
Tandjilé	1054
Wad Fira	732
bet	578
Total	####

Congo DR 2001	No.
Bandundu	1198
Bas-congo	542
Equateur	1160
Kasai Occidental	958
Kasai Oriental	1114
Katanga	1149
Kinshasa	1473
Maniema	262
Nord-Kivu	740
Orientale	1041
Sud-Kivu	617
Total	####

Congo DR 2010	No.
Bandundu	977
Bas congo	769
Equateur	1056
Kasai Occidental	1083
Kasai Oriental	1048
Katanga	1257
Kinshasa	843
Maniema	1017
Nord Kivu	1169
Province Orientale	894
Sud Kivu	1132
Total	####

Côte d’Ivoire 2006	No.
Centre	664

Centre Est	544
Centre Nord	761
Centre Ouest	799
Nord	653
Nord Est	555
Nord Ouest	927
Ouest	706
Sud (sans ville d' Abidjan)	889
Sud Ouest	1127
Ville Abidjan	979
Total	8604

Equatorial Guinea 2000	No.
Annobon	14
Bioko Norte	609
Bioko Sur	35
Centro Sur	267
Kie Ntem	506
Litoral	715
Wele Nzas	311
Total	2457

Gambia 2000	No.
Banjul	479
Basse	462
Brikama	451
Janjabureh	365
Kanifing	484
Kerewan	455
Kuntaur	574
Mansakonko	362
Total	3632

Gambia 2005	No.
Banjul	167
Basse	1161
Brikama	1390
Janjanburay	754
Kanifing	1461
Kerewan	863
Kuntaur	441
Mansakonko	404
Total	6641

Ghana 2006	No.
Ashanti	426
Brong Ahafo	245
Central	263
Eastern	346
Greater Accra	330
Northern	595

Upper East	399
Upper West	377
Volta	245
Western	319
Total	3545

Ghana 2011	No.
Asante	476
Brong Ahafo	410
Central	1009
Eastern	346
Greater Accra	400
Northern	2008
Upper East	997
Upper West	1157
Volta	402
Western	421
Total	7626

Guinea Bissau 2000	No.
Bafatá	971
Biombo	301
Bolama/Bijagós	172
Cacheu	773
Gabú	772
Oio	1131
Quinará	239
Tombali	319
sab	1173
Total	5851

Guinea Bissau 2006	No.
EST (Bafata e Gabu)	1519
NORD (Biombo, Cacheu e Oio)	2725
SAB Capital	1069
SUD (Bolama, Quinara e Tombali)	1257
Total	6570

Mauritania 2007	No.
Adrar	335
Assaba	940
Brakna	884
Gorgol	936
Guidimagha	884
Hodh ECharghi	784
Hodh ELGharbi	736
Inchiri	130
Nouadhibou	600
Nouakchott	1507
Tagant	311
Tiris Zemmour	308

Trarza	626
Total	8981

Niger 2000	No.
Diffa	68
Agadez	420
Dosso	618
Maradi	1207
Niamey	731
Tahoua	732
Tillaberi	644
Zinder	660
Total	5080

Nigeria 2007	No.
Abia	340
Abuja FCT	445
Adamawa	411
Akwa-Ibom	487
Anambra	347
Bauchi	789
Bayelsa	510
Benue	526
Borno	473
Cross-Rivers	369
Delta	333
Ebonyi	469
Edo	391
Ekiti	276
Enugu	341
Gombe	434
Imo	270
Jigawa	821
Kaduna	681
Kano	598
Katsina	546
Kebbi	509
Kogi	326
Kwara	330
Lagos	351
Nasarawa	544
Niger	608
Ogun	299
Ondo	348
Osun	250
Oyo	358
Plataeu	463
Rivers	316
Sokoto	447
Taraba	525
Yobe	733

Zamfara	829
Total	####

Nigeria 2011	No.
Abia	465
Adamawa	875
Akwa ibom	564
Anambra	562
Bauchi	1001
Bayelsa	552
Benue	633
Borno	796
Cross River	593
Delta	552
Ebonyi	685
Edo	519
Ekiti	402
Enugu	352
FCT (Abuja)	646
Gombe	971
Imo	414
Jigawa	1063
Kaduna	962
Kano	956
Katsina	1024
Kebbi	947
Kogi	430
Kwara	564
Lagos	545
Nasarawa	891
Niger	957
Ogun	588
Ondo	427
Osun	456
Oyo	592
Plateau	687
Rivers	467
Sokoto	1020
Taraba	792
Yobe	1015
Zamfara	1053
Total	####

Sao Tome et Principe 2000	No.
Centro	1557
Norte	442
Principe	104
Sul	105
Total	2208

Senegal 2000	No.

Dakar	1077
Diourbel	759
Fatick	908
Kaolack	968
Kolda	668
Louga	923
Saint louis	893
Tambacounda	897
Thies	1180
Ziguinchor	760
Total	9033

Sierra Leone 2000	No.
East	701
North	1048
South	357
West	598
Total	2704

Sierra Leone 2005	No.
East	1478
North	2273
South	1638
West	515
Total	5904

Sierra Leone 2010	No.
East	1942
North	3310
South	2410
West	1136
Total	8798

Togo 2006	No.
Centrale	601
Kara	601
Lomé commune	549
Maritime (sans Lomé commune)	831
Plateaux	569
Savanes	1003
Total	4154

Togo 2010	No.
Centrale	778
Kara	863
Lomé	506
Maritime	825
Plateaux	816
Savanes	1120
Total	4908

Appendix B

Proportion of children with values for height or weight that were in the acceptable range, flagged as implausible, had missing data, or for whom measurement was not applicable, by region DHS

Benin 2001, regions	In range	Flagged	Missing	Flagged/missing	NA	Total
	%	%	%	%	%	No.
atacora	97.1	2.5	0.4	2.9	2.9	700
atlantique	97.8	1.4	0.8	2.2	4.9	1008
borgou	96.7	2.8	0.5	3.3	5.9	836
mono	98.2	1.5	0.3	1.8	3.3	635
oueme	97.8	1.3	0.9	2.2	3.9	718
zou	98.1	1.5	0.4	1.9	3.6	832

Benin 2006, regions	In range	Flagged	Missing	Flagged/missing	NA	Total
	%	%	%	%	%	No.
alibori	80.7	16.4	2.9	19.3	6.0	1224
atacora	94.0	4.1	1.8	6.0	5.7	1385
atlantique	95.6	2.9	1.6	4.4	3.1	1626
borgou	90.7	6.4	2.9	9.3	5.4	1510
collines	90.8	7.5	1.7	9.2	4.7	1002
couffo	78.8	1.2	20.0	21.2	3.2	1233
donga	88.4	7.1	4.5	11.6	5.0	773
littoral	94.2	3.2	2.6	5.8	6.9	975
mono	86.8	2.0	11.2	13.2	4.2	973
plateau	90.4	6.5	3.2	9.6	5.4	735
quémé	90.0	5.5	4.5	10.0	5.2	1725
zou	86.6	10.5	2.9	13.4	2.6	1521

Benin 2011, regions	In range	Flagged	Missing	Flagged/missing	NA	Total
	%	%	%	%	%	No.
Alibori	77.8	14.4	7.7	22.2	10.3	935
Atacora	82.4	11.6	6.0	17.6	7.7	1318
Atlantique	67.3	27.5	5.3	32.7	5.3	1484
Borgou	66.9	22.6	10.5	33.1	6.4	1082
Collines	77.4	14.8	7.8	22.6	4.4	856
Couffo	94.9	4.1	1.0	5.1	4.0	961
Donga	84.0	10.8	5.2	16.0	4.7	701
Littoral	61.0	23.6	15.4	39.0	7.4	1036
Mono	87.3	9.9	2.8	12.7	4.1	750
Ou?m?	80.1	14.0	5.9	19.9	4.2	1490
Plateau	66.0	29.7	4.3	34.0	3.7	804
Zou	76.3	18.1	5.6	23.7	4.9	1262

Burkina Faso 1993, regions	In range	Flagged	Missing	Flagged/missing	NA	Total
	%	%	%	%	%	No.
central/south	90.9	4.4	4.6	9.1	4.1	1150
east	87.1	3.6	9.3	12.9	3.7	941
north	92.3	2.1	5.6	7.7	3.8	784
ouagadougou	93.6	1.1	5.2	6.4	5.2	925
west	91.5	2.1	6.3	8.5	4.6	1275

Burkina Faso 1998, regions	In range	Flagged	Missing	Flagged/missing	NA	Total
	%	%	%	%	%	No.
central/south	94.2	2.7	3.2	5.8	3.5	1280
east	94.5	3.8	1.7	5.5	3.8	1407
north	91.8	4.6	3.7	8.2	5.6	694
ouagadougou	95.9	1.5	2.6	4.1	7.6	499
west	95.9	2.8	1.3	4.1	3.9	1192

Burkina Faso 2003, regions	In range	Flagged	Missing	Flagged/missing	NA	Total
	%	%	%	%	%	No.
boucle de mouhoun	92.6	6.4	1.0	7.4	2.7	711
cascades	89.3	8.6	2.0	10.7	4.8	621
centre (sans ouagadougou)	88.2	5.7	6.1	11.8	2.8	288
centre-est	89.3	9.5	1.1	10.7	1.9	630
centre-nord	93.6	5.6	0.8	6.4	3.7	778
centre-ouest	87.7	3.2	9.2	12.3	4.7	928
centre-sud	85.7	13.3	1.0	14.3	1.7	603
est	84.0	14.0	2.1	16.0	0.7	678
hauts bassins	94.5	2.0	3.5	5.5	4.6	776
nord	94.5	4.9	0.6	5.5	5.6	713
ouagadougou	93.3	4.0	2.8	6.7	8.3	276
plateau central	88.7	9.8	1.5	11.3	2.0	858
sahel	91.0	5.6	3.4	9.0	5.8	624
sud-ouest	92.5	3.6	3.9	7.5	3.0	868

Burkina Faso 2010, regions	In range	Flagged	Missing	Flagged/missing	NA	Total
	%	%	%	%	%	No.
boucle de mouhoun	96.8	1.8	1.4	3.2	49.5	1244
cascades	95.5	3.6	1.0	4.5	51.1	855
centre	91.1	3.0	5.9	8.9	51.8	839
centre-est	93.1	4.0	2.9	6.9	49.4	1090
centre-nord	98.1	1.7	0.2	1.9	48.4	1047
centre-ouest	97.0	0.8	2.1	3.0	50.5	1222
centre-sud	95.8	2.6	1.6	4.2	50.8	876
est	92.3	4.6	3.1	7.7	52.1	1357
hauts basins	98.8	0.5	0.7	1.2	49.0	1143
nord	97.7	1.4	0.9	2.3	48.3	1085
plateau central	96.4	2.3	1.3	3.6	49.9	938
sahel	95.8	1.7	2.5	4.2	51.7	1085
sud-ouest	98.5	1.5	0.0	1.5	50.8	935

Cameroon 1991, regions	In range	Flagged	Missing	Flagged/missing	NA	Total
	%	%	%	%	%	No.
adam/nord/ext-nord	92.6	1.7	5.7	7.4	3.2	910
centre/sud/est	93.4	0.9	5.7	6.6	8.1	496
nord-ouest/sud-ouest	95.5	0.5	4.0	4.5	7.1	406
ouest/littoral	91.2	1.4	7.4	8.8	3.6	449
yaoundé/douala	89.1	0.3	10.6	10.9	6.4	777

Cameroon 1998, regions	In range	Flagged	Missing	Flagged/missing	NA	Total
	%	%	%	%	%	No.
central, south, & east	95.5	0.7	3.8	4.5	7.4	620
north/ extreme north/ ad	94.7	2.7	2.7	5.3	3.7	699
northwest & southwest	90.7	5.0	4.3	9.3	3.3	334

west & littoral	85.5	2.0	12.5	14.5	7.9	433

Cameroon 2011, regions	In range	Flagged	Missing	Flagged/missing	NA	Total
	%	%	%	%	%	No.
adamaoua	97.5	1.5	1.1	2.5	50.4	960
centre	98.0	0.7	1.2	2.0	56.9	949
douala	98.4	0.8	0.8	1.6	50.9	745
est	96.6	2.3	1.0	3.4	49.6	766
extrême-nord	94.2	3.5	2.3	5.8	50.0	1547
littoral	97.1	1.1	1.8	2.9	48.7	540
nord	95.3	2.8	1.9	4.7	49.9	1373
nord-ouest	94.3	2.8	3.0	5.7	46.2	938
ouest	95.0	1.9	3.1	5.0	51.4	992
sud	98.2	1.4	0.4	1.8	54.6	624
sud-ouest	98.4	1.0	0.6	1.6	49.8	622
yaoundé	95.5	1.9	2.5	4.5	53.7	678

Central African Rep 1994, regions	In range	Flagged	Missing	Flagged/missing	NA	Total
	%	%	%	%	%	No.
bangui	94.6	2.4	3.0	5.4	4.7	527
rs i	93.8	2.2	4.0	6.2	2.9	415
rs ii	95.1	2.6	2.3	4.9	3.6	443
rs iii	95.0	2.5	2.5	5.0	0.4	484
rs iv	95.6	3.5	0.9	4.4	2.0	351
rs v	94.2	3.1	2.8	5.8	3.0	337

Chad 1996, regions	In range	Flagged	Missing	Flagged/missing	NA	Total
	%	%	%	%	%	No.
b.e.t.	94.3	5.7	0.0	5.7	5.4	56
batha	97.6	0.8	1.6	2.4	2.4	252
biltine	93.6	0.7	5.7	6.4	4.7	148
chari-baguirmi	92.6	2.5	4.9	7.4	5.8	688
guéra	92.1	2.6	5.3	7.9	3.8	236
kanem	95.5	1.8	2.7	4.5	5.6	234
lac	89.9	8.3	1.8	10.1	3.1	225
logone occidental	94.9	2.7	2.5	5.1	3.3	422
logone oriental	92.4	2.0	5.5	7.6	4.3	415
mayo-kebbi	93.9	2.8	3.3	6.1	1.8	656
moyen chari	95.3	2.4	2.3	4.7	4.5	689
n'djaména	90.5	2.9	6.6	9.5	5.5	1071
ouaddaï	95.1	2.2	2.6	4.9	6.2	569
salamat	98.6	0.9	0.5	1.4	3.6	225
tandjilé	94.5	2.5	3.0	5.5	3.8	452

Chad 2004, regions	In range	Flagged	Missing	Flagged/missing	NA	Total
	%	%	%	%	%	No.
b. e. t.	91.7	6.5	1.8	8.3	3.9	513
bar azoum	92.0	6.4	1.6	8.0	0.7	440
centre est	92.9	4.9	2.2	7.1	3.1	424
chari baguirmi	90.1	3.9	6.0	9.9	4.0	504
logone occidental	90.5	4.1	5.4	9.5	2.6	608
mayo kebbi	93.6	3.1	3.3	6.4	2.9	559
moyen chari	97.6	1.1	1.3	2.4	1.5	465
n'djaména	89.5	5.4	5.0	10.5	2.9	1004
ouaddai est	92.8	5.5	1.7	7.2	1.7	409

Congo 2005, regions	In range	Flagged	Missing	Flagged/missing	NA	Total
	%	%	%	%	%	No.
brazzaville	94.2	2.9	2.9	5.8	7.0	1081
nord	94.5	3.3	2.2	5.5	6.4	1038
pointe noire	93.6	2.5	3.9	6.4	8.0	901
sud	96.1	3.0	0.9	3.9	6.1	1415

Congo 2012, regions	In range	Flagged	Missing	Flagged/missing	NA	Total
	%	%	%	%	%	No.
bouenza	99.3	0.7	0.0	0.7	45.1	794
brazzaville	95.6	1.6	2.8	4.4	53.7	684
cuvette	98.8	0.6	0.6	1.2	49.4	666
cuvette - ouest	94.0	1.4	4.6	6.0	51.4	587
kouilou	98.9	0.0	1.1	1.1	45.9	851
lekoumou	98.2	0.3	1.5	1.8	47.7	639
likouala	99.1	0.7	0.2	0.9	51.2	912
niari	99.0	0.7	0.2	1.0	47.2	788
plateaux	95.9	3.8	0.3	4.1	49.6	681
pointe-noire	98.6	0.2	1.1	1.4	45.5	802
pool	98.1	0.2	1.7	1.9	46.0	891
sangha	96.9	2.1	1.0	3.1	48.4	562

Congo DR 2007, regions	In range	Flagged	Missing	Flagged/missing	NA	Total
	%	%	%	%	%	No.
bandundu	89.2	7.0	3.8	10.8	49.6	734
bas-congo	80.6	5.2	14.2	19.4	51.0	590
equateur	81.2	9.1	9.7	18.8	54.2	789
kasaï occident	85.8	6.3	8.0	14.2	51.7	726
kasaï oriental	76.8	9.5	13.7	23.2	51.5	826
katanga	85.4	7.7	6.9	14.6	50.7	766
kinshasa	86.9	2.5	10.6	13.1	52.5	852
maniema	83.8	8.8	7.4	16.2	50.4	736
nord-kivu	85.0	8.4	6.7	15.0	48.0	691
orientale	86.0	6.8	7.2	14.0	48.5	567
sud-kivu	84.4	7.9	7.6	15.6	52.1	710

Côte d'Ivoire 1994, regions	In range	Flagged	Missing	Flagged/missing	NA	Total
	%	%	%	%	%	No.
center	98.2	1.2	0.6	1.8	4.4	341
center east	95.9	3.4	0.7	4.1	1.4	147
center north	97.5	1.6	0.9	2.5	3.9	331
center west	96.0	2.6	1.4	4.0	3.7	598
north	97.6	2.0	0.4	2.4	1.2	254
north east	96.3	2.1	1.6	3.7	2.6	192
north west	97.7	2.3	0.0	2.3	0.0	216
south	99.0	0.4	0.5	1.0	5.4	968
south west	98.4	1.6	0.0	1.6	2.0	251
west	96.2	3.2	0.6	3.8	4.4	360

Côte d'Ivoire 1998, regions	In range	Flagged	Missing	Flagged/missing	NA	Total
	%	%	%	%	%	No.
capital (abidjan)	97.3	1.6	1.1	2.7	8.0	476
countryside	97.6	2.0	0.5	2.4	4.3	691
small city	94.1	2.7	3.1	5.9	4.7	536

Côte d'Ivoire 2011, regions	In range	Flagged	Missing	Flagged/missing	NA	Total
	%	%	%	%	%	No.
Centre	95.7	2.5	1.8	4.3	51.5	582
Centre-Est	94.8	1.5	3.7	5.2	51.9	559
Centre-Nord	97.0	1.1	1.9	3.0	49.3	724
Centre-Ouest	87.3	1.5	11.2	12.7	50.2	679
Nord	96.7	1.8	1.5	3.3	50.7	671
Nord-Ouest	93.8	1.8	4.4	6.2	52.9	956
Nord-est	93.1	1.7	5.2	6.9	49.7	572
Ouest	96.7	1.6	1.6	3.3	45.7	672
Sud sans Abidjan	91.7	3.9	4.4	8.3	58.0	486
Sud-ouest	88.9	4.9	6.3	11.1	52.5	604
Ville d'Abidjan	87.2	4.9	7.9	12.8	54.9	588

Gabon 2000, regions	In range	Flagged	Missing	Flagged/missing	NA	Total
	%	%	%	%	%	No.
east (haut-ogooué & ogooué-lolo)	96.7	1.0	2.3	3.3	6.5	939
libreville,port-gentil	93.7	2.6	3.7	6.3	14.7	1007
north (ogooué-ivindo & woleu-ntem)	98.4	1.3	0.3	1.6	13.2	721
south (ngounié, nyanga)	99.0	0.7	0.3	1.0	11.3	770
west (estuaire, moyen-ogooué & ogooué-maritime)	97.6	0.7	1.7	2.4	11.9	663

Gabon 2012, regions	In range	Flagged	Missing	Flagged/missing	NA	Total
	%	%	%	%	%	No.
estuaire	94.3	1.7	4.0	5.7	41.1	506
haut-ogooué	93.0	5.6	1.4	7.0	33.4	622
libreville-port-gentil	92.0	2.4	5.6	8.0	37.5	797
moyen-ogooué	95.6	2.6	1.8	4.4	38.5	441
ngounié	96.7	1.4	1.9	3.3	37.0	663
nyanga	95.8	2.3	1.9	4.2	41.9	532
ogooué maritime	91.5	6.9	1.6	8.5	34.0	373
ogooué-ivindo	96.5	1.5	2.0	3.5	37.9	876
ogooué-lolo	84.5	4.7	10.8	15.5	36.0	534
woleu-ntem	95.5	2.9	1.6	4.5	39.5	403

Ghana 1993, regions	In range	Flagged	Missing	Flagged/missing	NA	Total
	%	%	%	%	%	No.
ashanti	90.5	2.9	6.6	9.5	0.0	379
brong-ahafo	94.5	1.5	4.0	5.5	0.0	200
central	94.4	2.8	2.8	5.6	0.0	213
eastern	91.0	3.2	5.9	9.0	0.0	222
greater accra	96.3	0.5	3.2	3.7	0.0	188
northern	89.4	5.7	4.8	10.6	0.0	227
upper east	94.4	1.4	4.2	5.6	0.0	143
upper west	93.0	7.0	0.0	7.0	0.0	71
volta	90.7	3.5	5.8	9.3	0.0	226
western	92.0	1.1	7.0	8.0	0.0	187

Ghana 1998, regions	In range	Flagged	Missing	Flagged/missing	NA	Total
	%	%	%	%	%	No.
ashanti region	95.8	2.0	2.2	4.2	4.0	420
brong ahafo region	94.4	2.0	3.6	5.6	3.0	202
central region	94.6	2.3	3.1	5.4	4.8	271
eastern region	98.5	0.6	0.9	1.5	4.6	347

greater accra region	96.1	2.0	2.0	3.9	4.9	267
northern region	95.9	2.4	1.7	4.1	3.6	304
upper east region	97.9	1.2	0.9	2.1	0.9	343
upper west region	89.3	4.9	5.7	10.7	6.5	261
volta region	95.3	1.9	2.7	4.7	3.4	267
western region	96.2	1.9	1.9	3.8	4.8	333

Ghana 2003, regions	In range	Flagged	Missing	Flagged/missing	NA	Total
	%	%	%	%	%	No.
ashanti	96.8	0.8	2.4	3.2	6.8	529
brong ahafo	95.4	2.8	1.8	4.6	7.8	424
central	96.3	2.3	1.4	3.7	2.7	221
eastern	95.4	3.2	1.4	4.6	4.1	294
greater accra	92.5	4.8	2.7	7.5	7.9	317
northern	93.4	2.6	4.0	6.6	6.9	569
upper east	91.4	2.7	5.9	8.6	21.1	279
upper west	92.6	3.8	3.5	7.4	5.5	330
volta	92.9	6.3	0.8	7.1	4.0	248
western	97.3	2.0	0.7	2.7	6.0	319

Ghana 2008, regions	In range	Flagged	Missing	Flagged/missing	NA	Total
	%	%	%	%	%	No.
ashanti	94.9	2.6	2.6	5.1	5.1	412
brong ahafo	96.4	0.4	3.2	3.6	2.4	254
central	84.5	7.2	8.2	15.5	6.7	208
eastern	85.7	5.2	9.1	14.3	6.1	245
greater accra	83.3	5.4	11.3	16.7	2.7	264
northern	89.4	3.1	7.4	10.6	3.5	432
upper east	82.5	4.7	12.7	17.5	2.3	217
upper west	89.4	2.4	8.2	10.6	3.4	264
volta	94.7	0.9	4.4	5.3	5.5	238
western	87.7	5.3	7.0	12.3	6.2	260

Guinea 1999, regions	In range	Flagged	Missing	Flagged/missing	NA	Total
	%	%	%	%	%	No.
central guinea	89.6	4.7	5.7	10.4	4.3	860
conakry	94.2	4.8	1.0	5.8	5.3	704
forest guinea	92.4	4.4	3.1	7.6	4.1	1337
lower guinea	95.1	3.0	1.8	4.9	6.1	1049
upper guinea	90.7	5.9	3.4	9.3	3.4	1016

Guinea 2005, regions	In range	Flagged	Missing	Flagged/missing	NA	Total
	%	%	%	%	%	No.
boké	95.3	2.6	2.1	4.7	52.9	724
conakry	83.8	9.3	6.9	16.2	53.0	460
faranah	96.6	2.6	0.8	3.4	48.3	743
kankan	95.8	2.6	1.6	4.2	44.2	892
kindia	96.0	2.7	1.2	4.0	51.7	833
labé	95.1	2.3	2.6	4.9	54.0	578
mamou	96.0	2.9	1.1	4.0	50.4	554
n'zérékoré	97.5	2.5	0.0	2.5	51.1	832

Guinea 2012, regions	In range	Flagged	Missing	Flagged/missing	NA	Total
	%	%	%	%	%	No.
Bok?	96.8	2.4	0.9	3.2	46.3	631

Conakry	93.3	2.2	4.4	6.7	56.7	728
Faranah	95.8	1.9	2.3	4.2	51.7	995
Kankan	95.2	4.0	0.9	4.8	44.9	1052
Kindia	95.2	3.8	1.0	4.8	47.4	747
Lab?	93.3	4.0	2.7	6.7	50.1	749
Mamou	95.7	2.8	1.4	4.3	49.0	688
N'Z?r?kor?	95.5	2.6	1.9	4.5	49.2	834

**Liberia 2007, regions**	**In range**	**Flagged**	**Missing**	**Flagged/missing**	**NA**	**Total**
	**%**	**%**	**%**	**%**	**%**	**No.**
monrovia	90.0	4.3	5.7	10.0	11.4	893
north central	91.8	3.2	5.1	8.2	9.6	1089
north western	94.2	2.1	3.6	5.8	9.4	671
south central	93.7	2.9	3.3	6.3	11.4	844
south eastern a	91.4	5.1	3.5	8.6	12.4	780
south eastern b	91.1	4.6	4.4	8.9	10.6	1028

**Mali 1995, regions**	**In range**	**Flagged**	**Missing**	**Flagged/missing**	**NA**	**Total**
	**%**	**%**	**%**	**%**	**%**	**No.**
bamako	87.5	7.7	4.8	12.5	2.6	532
gao	96.6	2.3	1.1	3.4	4.4	274
kayes	89.4	2.2	8.4	10.6	3.0	801
koulikoro	94.6	3.9	1.5	5.4	1.8	956
mopti	91.5	7.8	0.7	8.5	2.4	578
sikasso	94.0	5.5	0.5	6.0	0.5	954
ségou	92.4	6.7	0.8	7.6	1.6	861
timbuktu	91.1	6.2	2.7	8.9	1.5	262

**Mali 2001, regions**	**In range**	**Flagged**	**Missing**	**Flagged/missing**	**NA**	**Total**
	**%**	**%**	**%**	**%**	**%**	**No.**
bamako	94.4	3.2	2.4	5.6	10.4	1199
gao	96.3	2.3	1.5	3.8	7.6	433
kayes	95.3	3.2	1.5	4.7	5.7	1619
kidal	95.9	2.1	2.1	4.1	8.8	159
koulikoro	95.2	3.2	1.6	4.8	4.6	1768
mopti	94.0	4.6	1.5	6.0	8.1	1424
segou	90.1	7.6	2.2	9.9	5.0	1527
sikasso	96.2	2.9	0.8	3.8	4.0	2097
tombouctou	87.7	9.4	2.9	12.3	5.1	393

**Mali 2006, regions**	**In range**	**Flagged**	**Missing**	**Flagged/missing**	**NA**	**Total**
	**%**	**%**	**%**	**%**	**%**	**No.**
bamako	91.3	4.7	4.0	8.7	7.3	1318
gao	85.6	9.9	4.5	14.4	4.3	862
kayes	94.6	3.6	1.8	5.4	4.4	1562
kidal	91.2	6.6	2.2	8.8	4.9	286
koulikoro	94.5	3.9	1.6	5.5	3.3	1638
mopti	87.8	8.2	4.0	12.2	4.6	1951
segou	93.4	4.0	2.6	6.6	4.2	1491
sikasso	93.4	4.4	2.2	6.6	3.3	2456
tombouctou	92.1	4.8	3.1	7.9	4.2	873

**Niger 1992, regions**	**In range**	**Flagged**	**Missing**	**Flagged/missing**	**NA**	**Total**
	**%**	**%**	**%**	**%**	**%**	**No.**
agadez	92.6	6.4	1.0	7.4	4.7	213

diffa	89.8	5.5	4.7	10.2	8.6	139
dosso	87.7	1.3	11.0	12.3	4.8	647
maradi	86.4	4.3	9.4	13.6	5.5	969
niamey	94.4	1.6	4.0	5.6	6.3	989
tahoua	86.5	3.5	10.0	13.5	4.1	925
tillabéri	94.1	1.4	4.5	5.9	6.0	753
zinder	91.6	4.6	3.8	8.4	8.2	917

Niger 1998, regions	In range	Flagged	Missing	Flagged/missing	NA	Total
	%	%	%	%	%	No.
dosso	96.8	2.3	0.8	3.2	1.6	609
maradi	94.2	3.1	2.6	5.8	3.1	1023
niamey	93.5	2.2	4.2	6.5	2.4	413
tahoua/agadez	94.4	2.3	3.3	5.6	1.8	712
tillaberi	96.8	2.0	1.2	3.2	2.2	765
zinda/diffa	93.8	3.8	2.4	6.2	3.7	704

Niger 2006, regions	In range	Flagged	Missing	Flagged/missing	NA	Total
	%	%	%	%	%	No.
agadez	85.6	4.0	10.4	14.4	56.2	683
diffa	96.0	2.7	1.2	4.0	54.5	723
dosso	98.0	1.1	0.9	2.0	50.3	1284
maradi	93.2	4.8	2.1	6.8	50.2	1268
niamey	94.0	1.6	4.4	6.0	50.3	873
tahoua	94.4	2.7	2.9	5.6	49.3	1296
tillabéri	91.5	5.5	3.1	8.5	50.9	1120
zinder	90.5	5.4	4.1	9.5	54.0	962

Niger 2012, regions	In range	Flagged	Missing	Flagged/missing	NA	Total
	%	%	%	%	%	No.
Agadez	83.4	6.3	10.3	16.6	48.0	579
Diffa	67.8	13.3	18.9	32.2	52.9	1035
Dosso	97.0	0.9	2.1	3.0	51.3	1580
Maradi	87.9	6.3	5.8	12.1	50.4	2372
Niamey	86.4	4.6	9.0	13.6	54.6	959
Tahoua	86.9	7.5	5.6	13.1	54.2	1899
Tillaberi	96.2	1.4	2.4	3.8	52.7	1485
Zinder	87.1	3.8	9.2	12.9	57.5	1693

Nigeria 1990, regions	In range	Flagged	Missing	Flagged/missing	NA	Total
	%	%	%	%	%	No.
northeast	85.2	6.4	8.5	14.8	5.1	1654
northwest	89.7	3.4	6.9	10.3	4.4	1542
southeast	95.9	2.9	1.2	4.1	5.8	1780
southwest	94.5	2.1	3.4	5.5	6.5	1811

Nigeria 2003, regions	In range	Flagged	Missing	Flagged/missing	NA	Total
	%	%	%	%	%	No.
north central	95.8	3.6	0.6	4.2	4.1	875
north east	95.5	3.9	0.6	4.5	4.9	1159
north west	85.7	12.3	2.0	14.3	3.1	1475
south east	91.4	6.3	2.3	8.6	3.7	458
south south	91.4	7.5	1.1	8.6	6.4	471
south west	95.5	3.3	1.3	4.5	3.3	570

Nigeria 2008, regions	In range	Flagged	Missing	Flagged/missing	NA	Total
	%	%	%	%	%	No.
north central	80.6	12.5	6.9	19.4	5.5	4542
north east	79.1	13.8	7.1	20.9	4.1	5737
north west	73.1	16.5	10.4	26.9	4.8	6899
south east	82.9	9.9	7.2	17.1	6.4	2173
south south	82.8	9.3	7.9	17.2	6.3	2997
south west	88.6	7.6	3.8	11.4	5.2	3098

Sao Tome et Principe 2008, regions	In range	Flagged	Missing	Flagged/missing	NA	Total
	%	%	%	%	%	No.
região centro	70.0	18.7	11.2	30.0	6.6	572
região do principe	94.1	4.5	1.4	5.9	2.0	294
região norte	87.7	9.1	3.2	12.3	3.1	513
região sul	85.0	11.1	3.9	15.0	2.8	472

Senegal 1992, regions	In range	Flagged	Missing	Flagged/missing	NA	Total
	%	%	%	%	%	No.
central	93.7	2.5	3.8	6.3	4.6	1903
north east	91.1	5.0	3.8	8.9	3.8	868
south	93.3	2.6	4.0	6.7	6.9	610
west	94.3	1.8	3.9	5.7	5.2	1687

Senegal 2005, regions	In range	Flagged	Missing	Flagged/missing	NA	Total
	%	%	%	%	%	No.
dakar	90.0	3.0	7.0	10.0	72.4	727
diourbel	93.6	1.2	5.2	6.4	69.3	1072
fatick	94.7	2.0	3.3	5.3	72.5	886
kaolack	94.9	1.1	4.0	5.1	70.3	1192
kolda	98.8	0.6	0.6	1.2	66.7	1002
louga	87.3	1.4	11.3	12.7	69.1	920
matam	84.6	0.4	15.0	15.4	69.8	882
saint-louis	85.8	1.2	13.0	14.2	67.9	791
tambacounda	93.7	3.0	3.3	6.3	68.2	950
thiès	94.7	1.4	3.9	5.3	65.5	1031
zuguinchor	96.3	0.5	3.2	3.7	67.0	573

Senegal 2010, regions	In range	Flagged	Missing	Flagged/missing	NA	Total
	%	%	%	%	%	No.
dakar	91.4	3.3	5.2	8.6	64.6	759
diourbel	81.4	4.4	14.1	18.6	64.2	1008
fatick	92.3	2.4	5.3	7.7	60.4	854
kaffrine	95.8	2.7	1.5	4.2	62.4	894
kaolack	88.6	2.8	8.6	11.4	61.6	1120
kedougou	75.7	4.1	20.3	24.3	62.7	397
kolda	93.4	3.4	3.1	6.6	63.6	964
louga	86.3	7.2	6.5	13.7	61.9	843
matam	80.8	4.8	14.4	19.2	62.8	782
saint-louis	81.1	4.6	14.2	18.9	63.4	768
sedhiou	92.2	3.7	4.1	7.8	66.6	884
tambacounda	80.2	3.1	16.7	19.8	64.6	912
thiès	88.5	5.3	6.2	11.5	61.4	879
ziguinchor	96.7	2.2	1.1	3.3	68.4	569

Sierra Leone 2008, regions	In range	Flagged	Missing	Flagged/missing	NA	Total
	%	%	%	%	%	No.
eastern	85.7	9.9	4.4	14.3	55.0	1352
northern	87.5	6.2	6.2	12.5	49.9	1668
southern	82.8	12.9	4.3	17.2	52.1	1212
western	86.4	5.7	7.9	13.6	54.7	811

Togo 1998, regions	In range	Flagged	Missing	Flagged/missing	NA	Total
	%	%	%	%	%	No.
centrale	95.4	0.7	4.0	4.6	0.8	608
kara	96.7	1.8	1.5	3.3	0.2	609
lomé	94.1	2.3	3.6	5.9	4.7	322
marities	96.3	2.4	1.4	3.7	1.4	517
plateaux	94.7	4.1	1.1	5.3	0.7	803
savanes	95.8	3.1	1.1	4.2	0.2	1004

Proportion of children with values for height or weight that were in the acceptable range, flagged as implausible, or had missing data, by region NNS

Benin 2008, regions	In range	Flagged	Missing	Flagged/missing	Total
	%	%	%	%	No.
alibori	91.1	0	8.9	8.9	403
atacora	93.5	0.5	6	6.5	368
atlantique	98.7	0	1.3	1.3	309
borgou	95.5	1.2	3.3	4.5	334
collines	94.6	0	5.4	5.4	261
couffo	98.5	0	1.5	1.5	398
donga	96.2	1.9	1.9	3.8	262
littoral	98.2	0.4	1.3	1.8	223
mono	96.7	0.8	2.4	3.3	246
oueme	92.6	0.4	7	7.4	271
plateau	94.9	0.9	4.3	5.1	235
zou	96.3	0.4	3.3	3.7	242

Burkina Faso 2012, regions	In range	Flagged	Missing	Flagged/missing	Total
	%	%	%	%	No.
bales	99.7	0	0.3	0.3	775
bam	97.7	0	2.3	2.3	841
banwa	98.1	0.5	1.4	1.9	623
banwa ganzourgou	97.2	0.4	2.5	2.8	856
bazega	99	0	1	1	598
boulgou	99.1	0	0.9	0.9	689
cascades	99.2	0.2	0.6	0.8	644
centre ouest	99.7	0	0.3	0.3	582
est	99.7	0.1	0.2	0.3	922
houet	96.5	0	3.5	3.5	491
kadiogo	96.8	0	3.2	3.2	441
kenedougou	99.9	0	0.1	0.1	753
kossi	98.1	0.3	1.6	1.9	756
koulpelogo	98.8	0.1	1.1	1.2	834
kouritenga	99.7	0	0.3	0.3	708
kourweogo	99.1	0	0.9	0.9	660
mouhoun	99.4	0	0.6	0.6	641
nahouri	99.8	0	0.2	0.2	498
namentenga	99	0.3	0.7	1	881
nayala	94.3	0	5.7	5.7	680
nord	98.1	0	1.9	1.9	731
oubritenga	97.3	0	2.7	2.7	670
sahel	98	0.1	1.9	2	851
sanmentenga	99.4	0	0.6	0.6	862
sourou	99.5	0	0.5	0.5	660
sud ouest	96.9	0	3.1	3.1	513
tuy	99.1	0	0.9	0.9	565
zoundweogo	96.5	0.2	3.3	3.5	629

Cameroon 2011, regions	In range	Flagged	Missing	Flagged/missing	Total
	%	%	%	%	No.
extrême-nord	89	0.6	10.4	11	720

nord	87.5	0.8	11.7	12.5	761

Central African Rep 2012, regions	In range	Flagged	Missing	Flagged/missing	Total
	%	%	%	%	No.
bamingui bangoran	95.9	0.4	3.7	4.1	918
bangui	99.4	0	0.6	0.6	812
basse kotto	97.3	1.1	1.7	2.7	946
haut kotto	99.7	0.3	0	0.3	974
haut mbomou	99.5	0	0.5	0.5	950
kemo	99.1	0.3	0.5	0.9	933
lobaye	99.4	0.5	0.2	0.6	1091
mambere kadei	97.3	1.2	1.5	2.7	1299
mbomou	99.6	0.2	0.2	0.4	983
nana grebizi	99.5	0.4	0.1	0.5	1073
nana mambere	98	1.1	0.9	2	657
ombella mpoko	98.1	0.6	1.3	1.9	891
ouaka	96.6	0.7	2.7	3.4	824
ouham	97.4	1.1	1.5	2.6	998
ouham pende	97	0.3	2.8	3	799
sangha mbarere	99.4	0.5	0.2	0.6	1083
vakaga	98.4	0	1.6	1.6	510

Chad June 2012, regions	In range	Flagged	Missing	Flagged/missing	Total
	%	%	%	%	No.
Barh El Ghazal	95.5	0.3	4.3	4.5	750
Batha	91.8	0.4	7.8	8.2	748
Guéra	93	0.4	6.6	7	785
Hadjer Lamis	95.4	0.2	4.4	4.6	633
Kanem	93.5	0	6.5	6.5	599
Lac	92.5	0.5	7	7.5	570
N'Djamena	92	0.5	7.5	8	800
Ouaddai	97.8	0	2.2	2.2	602
Salamat	91.8	0.8	7.5	8.2	778
Sila	88.8	0.1	11.1	11.2	768
Wadi Fira	96.5	0.1	3.4	3.5	744

Chad (7 regions) Dec/Jan 2012-13, regions	In range	Flagged	Missing	Flagged/missing	Total
	%	%	%	%	No.
Logone Occidental	95.4	0	4.6	4.6	830
Logone Oriental	93.5	0.4	6.1	6.5	835
Mandoul	98.6	0.4	1	1.4	813
Mayo-Kebbi Est	95.6	0.5	3.9	4.4	1023
Mayo-Kebbi Ouest	91.4	0.6	8	8.6	673
Moyen-Chari	96.4	0.4	3.2	3.6	720
Tandjilé	98.7	0.2	1.1	1.3	905

The Gambia 2012, regions	In range	Flagged	Missing	Flagged/missing	Total
	%	%	%	%	No.
banjul	95.7	0.4	3.9	4.3	745
basse	98.6	0.1	1.4	1.4	1332
brikama	96.7	0.4	2.9	3.3	855
janjanburay	98.8	0.5	0.7	1.2	1013
kanifing	97.6	0.1	2.2	2.4	764
kerewan	98.7	0.4	0.8	1.3	1114
kuntaur	96.6	0.1	3.3	3.4	1112

mansakonko	98.5	0	1.5	1.5	844

Guinea-Bissau 2008, regions	In range	Flagged	Missing	Flagged/missing	Total
	%	%	%	%	No.
Capitale	98.7	0	1.3	1.3	445
Est (Bafata e Gabu)	99.2	0.2	0.6	0.8	836
Nord (Biombo, Cacheu e Oio)	97.2	0.9	1.9	2.8	752
Sud (Bolama, Quinara e Tombali)	98.1	1.5	0.4	1.9	685

Guinée Conakay 2012, regions	In range	Flagged	Missing	Flagged/missing	Total
	%	%	%	%	No.
boke nord	99.6	0.1	0.2	0.4	855
boke sud	99	0.3	0.7	1	709
conakry	98.7	0.2	1.1	1.3	891
faranah	99.3	0.3	0.4	0.7	700
kankan	98.9	0.2	0.8	1.1	843
kindia	99	0.2	0.8	1	887
labe	99.1	0.4	0.6	0.9	1083
mamou	98.7	0.4	1	1.3	1132
nzerekore	99.1	0.2	0.7	0.9	1108

Liberia 2010, regions	In range	Flagged	Missing	Flagged/missing	Total
	%	%	%	%	No.
bomi	97	0.3	2.7	3	334
bong	96.4	0.6	2.9	3.6	477
gbarpolu	94.9	1.1	4.1	5.1	369
grand bassa	98	1	1	2	405
grand cape mount	98.6	0.6	0.8	1.4	500
grand gedeh	98.4	0.5	1.1	1.6	443
grand kru	97.3	1.1	1.6	2.7	450
lofa	98.3	0.4	1.2	1.7	481
margibi	99	0.3	0.8	1	385
maryland	95.7	1.9	2.4	4.3	373
montserrado	98.9	0	1.1	1.1	350
nimba	99.1	0	0.9	0.9	441
river gee	94.3	1.9	3.8	5.7	422
rivercess	98.3	0.4	1.3	1.7	468
rural montserrado	97.4	0.5	2.1	2.6	421
sinoe	97.7	0.2	2.1	2.3	487

Liberia 2011, regions	In range	Flagged	Missing	Flagged/missing	Total
	%	%	%	%	No.
North Central	97.8	0.7	1.5	2.2	269
North Western	96.8	1.1	2.2	3.2	93
South Central	99.4	0.6	0	0.6	1002
South Eastern	96.7	2.5	0.8	3.3	120

Mali 2011, regions	In range	Flagged	Missing	Flagged/missing	Total
	%	%	%	%	No.
bamako	94.2	0.2	5.7	5.8	1273
gao	99.6	0.3	0.2	0.4	1133
kayes	94.3	0.1	5.6	5.7	1085
kidal	99.1	0.9	0	0.9	233
koulikoro	99	0.2	0.8	1	899
mopti	98.2	0.2	1.6	1.8	1020

segou	98.4	0	1.6	1.6	676
sikasso	95.5	0.2	4.3	4.5	1232
tombouctou	99.9	0	0.1	0.1	680

Mauritania 2006, regions	In range	Flagged	Missing	Flagged/missing	Total
	%	%	%	%	No.
Centre	95.7	2.4	1.9	4.3	417
Fleuve	93.5	3.8	2.7	6.5	849
Nord	96.5	1.7	1.7	3.5	230
Nouakchott	94.1	5.1	0.9	5.9	1857
SudEst	94.7	3.8	1.5	5.3	528

Mauritania March 2008, regions	In range	Flagged	Missing	Flagged/missing	Total
	%	%	%	%	No.
Centre	98.8	1.2	0	1.2	953
Fleuve Nord	98.9	1	0.1	1.1	1330
Fleuve Sud	99.1	0.7	0.2	0.9	1012
Nord	99.6	0.4	0	0.4	493
Nouakchott	98.5	1.5	0	1.5	1375
SudEst	98.4	1.3	0.3	1.6	1222

Mauritania Dec 2008, regions	In range	Flagged	Missing	Flagged/missing	Total
	%	%	%	%	No.
Centre	99.7	0.1	0.2	0.3	959
Nord	99.9	0.1	0	0.1	710
Nouakchott A	99.6	0.2	0.1	0.4	804
Nouakchott B	99.9	0	0.1	0.1	927
Sud	99.4	0.3	0.3	0.6	1072
SudEst	99.7	0.2	0.1	0.3	989
Trarza	100	0	0	0	881

Mauritania 2009, regions	In range	Flagged	Missing	Flagged/missing	Total
	%	%	%	%	No.
Centre	100	0	0	0	638
Nord	99.9	0.1	0	0.1	688
Nouakchott	99.9	0	0.1	0.1	679
Sud	100	0	0	0	965
Sud-est	100	0	0	0	699
Trarza	100	0	0	0	709

Mauritania July 2010, regions	In range	Flagged	Missing	Flagged/missing	Total
	%	%	%	%	No.
adrar/inchiri tiris	100	0	0	0	441
assaba	100	0	0	0	755
brakna	99.8	0.2	0	0.2	576
gorgol	100	0	0	0	723
guidimakha	99.7	0.3	0	0.3	632
hodh chargui	99.6	0.4	0	0.4	485
hodh gharbi	100	0	0	0	531
nouadhibou	100	0	0	0	505
nouakchott	99.2	0.8	0	0.8	505
tagant	99.4	0.6	0	0.6	646
trarza	100	0	0	0	584

Mauritania Dec 2010, regions	In range	Flagged	Missing	Flagged/missing	Total
	%	%	%	%	No.
adrar/inchiri tiris	98.8	0	1.2	1.2	408
assaba	100	0	0	0	556
brakna	100	0	0	0	567
gorgol	99.8	0.2	0	0.2	553
guidimakha	99.5	0.3	0.2	0.5	600
hodh chargui	100	0	0	0	471
hodh gharbi	99.8	0.2	0	0.2	551
nouadhibou	99.6	0.4	0	0.4	563
nouakchott	99.4	0.4	0.2	0.6	517
tagant	99.8	0	0.2	0.2	589
trarza	99.2	0.2	0.6	0.8	476

Mauritania July 2011, regions	In range	Flagged	Missing	Flagged/missing	Total
	%	%	%	%	No.
adrar/inchiri tiris	98.5	0.7	0.7	1.5	678
assaba	100	0	0	0	706
brakna	99.3	0.1	0.5	0.7	743
gorgol	100	0	0	0	680
guidimakha	98.6	1	0.4	1.4	769
hodh chargui	87.6	0.8	11.6	12.4	647
hodh gharbi	99	0.8	0.3	1	777
nouadhibou	100	0	0	0	674
nouakchott	99.7	0.3	0	0.3	645
tagant	99.5	0.3	0.3	0.5	742
trarza	97.9	1.5	0.6	2.1	719

Mauritania Dec 2011, regions	In range	Flagged	Missing	Flagged/missing	Total
	%	%	%	%	No.
adrar/inchiri tiris	99.7	0.3	0	0.3	690
assaba	100	0	0	0	763
brakna	99.4	0	0.6	0.6	701
gorgol	99.5	0.1	0.4	0.5	791
guidimakha	99.5	0.5	0	0.5	855
hodh chargui	99.8	0.2	0	0.2	628
hodh gharbi	99.6	0.1	0.2	0.4	803
nouadhibou	99.7	0.3	0	0.3	716
nouakchott	99.4	0.4	0.1	0.6	711
tagant	100	0	0	0	789
trarza	99.4	0.6	0	0.6	726

Mauritania July 2012, regions	In range	Flagged	Missing	Flagged/missing	Total
	%	%	%	%	No.
adrar/inchiri tiris	99.6	0.2	0.2	0.4	451
assaba	99.8	0	0.2	0.2	640
brakna	100	0	0	0	573
gorgol	100	0	0	0	610
guidimakha	99.6	0.2	0.1	0.4	856
hodh chargui	99.7	0.3	0	0.3	670
hodh gharbi	100	0	0	0	742
nouadhibou	100	0	0	0	474
nouakchott	98.8	0.5	0.7	1.2	590
tagant	100	0	0	0	755
trarza	99.6	0.4	0	0.4	456

Niger 2012, regions	In range	Flagged	Missing	Flagged/missing	Total
	%	%	%	%	No.
agadez	99.7	0	0.3	0.3	1223
diffa	98.1	0.3	1.6	1.9	1311
dosso	98.4	0.4	1.2	1.6	1123
maradi	99.4	0.1	0.5	0.6	1093
niamey	98.5	0.1	1.4	1.5	1173
tahoua	98.9	0.7	0.3	1.1	1469
tillabéri	99.3	0.3	0.3	0.7	895
zinder	98.2	0.2	1.6	1.8	939

Nigeria (Northern States) 2011, regions	In range	Flagged	Missing	Flagged/missing	Total
	%	%	%	%	No.
jigawa	91.4	0.5	8.1	8.6	954
kano	84.5	0.8	14.7	15.5	917
katsina	91.5	0.2	8.3	8.5	1003
kebbi	96.1	0.3	3.6	3.9	1021
sokoto	92.2	0.2	7.6	7.8	945
yobe	93.9	0.4	5.8	6.1	1074
zamfara	93.2	0.6	6.2	6.8	995

Senegal 2012, regions	In range	Flagged	Missing	Flagged/missing	Total
	%	%	%	%	No.
dakar	80.3	0	19.7	19.7	487
kolda	99.6	0.2	0.2	0.4	552
matam	99.9	0	0.1	0.1	1948
myf	98.9	0.1	1	1.1	726
sedhiou	93	0	6.9	7	2212
tambacounda	94.8	0	5.2	5.2	3114
velingara	89.6	0.1	10.2	10.4	676

Sierra Leone 2010, regions	In range	Flagged	Missing	Flagged/missing	Total
	%	%	%	%	No.
eastern	98.9	0.1	1	1.1	2706
northern	99.2	0.2	0.6	0.8	5043
southern	99.1	0.4	0.5	0.9	3987
western	98.4	0.6	1.1	1.6	2281

Togo June 2012, regions	In range	Flagged	Missing	Flagged/missing	Total
	%	%	%	%	No.
Centrale	96.6	0	3.4	3.4	417
Kara	95.4	0.2	4.4	4.6	612
Lomé	94.3	0	5.7	5.7	335
Maritime	94.8	0.6	4.6	5.2	479
Plateaux	96.2	0.3	3.5	3.8	372
Savanes	95.6	0	4.4	4.4	565

Togo Dec 2012, regions	In range	Flagged	Missing	Flagged/missing	Total
	%	%	%	%	No.
Kara	94	0.2	5.9	6	649
Savanes	98.1	0.1	1.7	1.9	802

Proportion of children with values for height or weight that were in the acceptable range, flagged as implausible, or had missing data, by region MICS

Burkina Faso 2006, regions	In range	Flagged	Missing	Flagged/missing	Total
	%	%	%	%	No.
Boucle du Mouhoun	89.4	3.4	7.2	10.6	415
Cascade	78.5	12.9	8.6	21.5	627
Centre	82.7	2.5	14.8	17.3	519
Centre-Est	84.1	9.9	6.0	15.9	333
Centre-Nord	77.8	8.3	13.9	22.2	432
Centre-Ouest	85.7	3.2	11.1	14.3	531
Centre-Sud	90.4	3.6	6.0	9.6	366
Est	73.4	9.6	17.0	26.6	481
Hauts-Bassins	87.8	4.5	7.7	12.2	336
Nord	81.8	8.9	9.3	18.2	484
Plateau-Central	83.8	3.3	12.9	16.2	456
Sahel	76.9	17.3	5.8	23.1	381
Sud-Ouest	83.2	5.7	11.1	16.8	316

Cameroon 2006, regions	In range	Flagged	Missing	Flagged/missing	Total
	%	%	%	%	No.
Adamaoua	94.8	1.2	4.0	5.2	649
Centre	90.0	2.5	7.5	10.0	560
Douala	87.8	4.2	8.1	12.2	385
Est	93.5	2.2	4.3	6.5	674
Extreme Nord	93.6	1.7	4.7	6.4	747
Littoral	96.2	1.2	2.6	3.8	426
Nord	85.0	6.6	8.3	15.0	815
Nord Ouest	83.3	7.3	9.4	16.7	383
Ouest	93.4	2.2	4.4	6.6	547
Sud	86.0	4.1	9.8	14.0	458
Sud Ouest	94.0	3.7	2.4	6.0	382
Yaounde	82.1	2.8	15.1	17.9	469

Central African Rep 2000, regions	In range	Flagged	Missing	Flagged/missing	Total
	%	%	%	%	No.
Bamingui-Bangoran	92.0	4.1	3.9	8.0	465
Bangui	87.6	5.2	7.2	12.4	1620
Basse-Kotto	83.4	6.8	9.7	16.6	1099
Haut-Mbomou	86.8	5.3	7.9	13.2	378
Haute-Kotto	92.3	2.9	4.8	7.7	790
Kémo	88.0	5.8	6.2	12.0	1031
Lobaye	86.6	5.9	7.5	13.4	1122
Mambéré-Kadeï	91.0	5.6	3.4	9.0	755
Mbomou	79.3	4.8	15.9	20.7	899
Nana-Grébizi	94.2	2.5	3.3	5.8	1027
Nana-Mambéré	89.7	5.6	4.7	10.3	536
Ombella-M'poko	89.5	4.8	5.7	10.5	1094
Ouaka	88.9	4.4	6.8	11.1	987
Ouham	91.7	4.8	3.5	8.3	624
Ouham-Pendé	85.3	11.3	3.4	14.7	556
Sangha-Mbaéré	92.8	4.1	3.1	7.2	704

Vakaga	89.1	5.7	5.2	10.9	613

**Central African Rep 2006, regions**	**In range**	**Flagged**	**Missing**	**Flagged/missing**	**Total**
	**%**	**%**	**%**	**%**	**No.**
“Baminigui Bangoran”	83.5	4.2	12.3	16.5	577
“Bangui”	77.2	6.0	16.8	22.8	754
“Basse Kotto”	84.9	7.0	8.1	15.1	484
“Haut Mbomou”	78.8	6.0	15.1	21.2	364
“Haute Kotto”	91.9	4.8	3.2	8.1	1077
“Lobaye”	94.1	5.2	0.8	5.9	657
“Mambere Kadei”	90.3	4.2	5.5	9.7	528
“Mbomou”	87.4	7.7	4.9	12.6	556
“Nana Mambere”	82.9	9.4	7.6	17.1	1271
“Ombella Mpoko”	81.1	7.6	11.3	18.9	644
“Ouaka”	88.3	5.6	6.1	11.7	675
“Ouham Pende”	84.6	5.2	10.2	15.4	629
“Ouham”	92.4	4.2	3.3	7.6	688
“Sangha Mbaere”	93.3	3.6	3.1	6.7	916

**Central African Rep 2010, regions**	**In range**	**Flagged**	**Missing**	**Flagged/missing**	**Total**
	**%**	**%**	**%**	**%**	**No.**
Baminigui Bangoran	88.3	0.0	11.7	11.7	427
Bangui	88.1	0.7	11.2	11.9	717
Basse Kotto	93.8	0.4	5.7	6.2	698
Haut Mbomou	90.9	0.5	8.6	9.1	373
Haute-Kotto	95.8	0.3	3.9	4.2	662
Kémo	97.3	0.7	2.0	2.7	846
Lobaye	92.2	1.0	6.7	7.8	890
Mambere Kadei	93.2	0.8	6.0	6.8	749
Mbomou	91.7	0.7	7.7	8.3	612
Nana Grebizi	93.1	0.7	6.2	6.9	696
Nana Mambéré	96.5	1.0	2.5	3.5	605
Ombella Mpoko	97.1	0.6	2.3	2.9	784
Ouaka	95.6	0.4	4.0	4.4	571
Ouham	94.5	0.9	4.7	5.5	685
Ouham Pende	94.4	0.3	5.4	5.6	710
Sangha Mbaere	93.8	0.6	5.6	6.2	821
Vakaga	82.8	0.0	17.2	17.2	58

**Chad 2000, regions**	**In range**	**Flagged**	**Missing**	**Flagged/missing**	**Total**
	**%**	**%**	**%**	**%**	**No.**
Autres villes	95.3	2.8	2.0	4.7	2000
N'Djaména	98.0	0.6	1.4	2.0	846
Rural	96.3	1.9	1.9	3.7	2538

**Chad 2010, regions**	**In range**	**Flagged**	**Missing**	**Flagged/missing**	**Total**
	**%**	**%**	**%**	**%**	**No.**
Barh El Gazal	83.3	4.0	12.7	16.7	753
Bhata	84.0	2.7	13.3	16.0	948
Chari Baguirmi	86.7	2.8	10.6	13.3	795
Guéra	85.0	2.5	12.4	15.0	1462
Hadjer Lamis	82.0	5.3	12.8	18.0	837
Kanem	76.3	8.5	15.2	23.7	710
Lac	82.4	5.0	12.6	17.6	716
Logone Occidental	82.3	2.0	15.7	17.7	747

Logone Oriental	89.2	3.6	7.2	10.8	934
Mandoul	90.0	1.0	9.0	10.0	880
Mayo Kebbi Est	83.4	5.0	11.6	16.6	1219
Mayo Kebbi Ouest	86.8	1.3	11.9	13.2	910
Moyen Chari	89.3	2.0	8.7	10.7	736
Ndjaména	79.6	2.7	17.7	20.4	983
Ouaddai	81.9	5.1	13.0	18.1	922
Salamat	78.2	8.2	13.5	21.8	864
Sila	79.4	5.7	14.9	20.6	933
Tandjilé	79.2	5.5	15.3	20.8	1054
Wad Fira	83.3	3.7	13.0	16.7	732
bet	74.7	2.2	23.0	25.3	578

Congo DR 2001, regions	In range	Flagged	Missing	Flagged/missing	Total
	%	%	%	%	No.
Bandundu	89.1	7.1	3.8	10.9	1198
Bas-congo	93.2	4.4	2.4	6.8	542
Equateur	91.6	6.8	1.6	8.4	1160
Kasai Occidental	93.2	5.5	1.3	6.8	958
Kasai Oriental	86.5	6.6	6.8	13.5	1114
Katanga	90.8	6.0	3.2	9.2	1149
Kinshasa	91.8	4.1	4.1	8.2	1473
Maniema	85.5	11.8	2.7	14.5	262
Nord-Kivu	90.0	5.0	5.0	10.0	740
Orientale	89.4	4.6	6.0	10.6	1041
Sud-Kivu	88.2	6.8	5.0	11.8	617

Congo DR 2010, regions	In range	Flagged	Missing	Flagged/missing	Total
	%	%	%	%	No.
Bandundu	93.6	3.8	2.7	6.4	977
Bas congo	95.7	2.2	2.1	4.3	769
Equateur	91.6	3.2	5.2	8.4	1056
Kasai Occidental	96.7	1.5	1.8	3.3	1083
Kasai Oriental	92.4	3.4	4.2	7.6	1048
Katanga	94.6	3.3	2.1	5.4	1257
Kinshasa	94.4	0.8	4.7	5.6	843
Maniema	91.9	2.3	5.8	8.1	1017
Nord Kivu	90.2	2.9	6.8	9.8	1169
Province Orientale	92.5	2.2	5.3	7.5	894
Sud Kivu	93.6	2.2	4.2	6.4	1132

Côte d’Ivoire 2006, regions	In range	Flagged	Missing	Flagged/missing	Total
	%	%	%	%	No.
Centre	97.4	1.8	0.8	2.6	664
Centre Est	99.4	0.6	0.0	0.6	544
Centre Nord	97.4	2.0	0.7	2.6	761
Centre Ouest	97.0	2.8	0.3	3.0	799
Nord	97.4	2.5	0.2	2.6	653
Nord Est	94.2	5.4	0.4	5.8	555
Nord Ouest	90.0	8.6	1.4	10.0	927
Ouest	94.3	5.1	0.6	5.7	706
Sud (sans ville d' Abidjan)	95.8	2.9	1.2	4.2	889
Sud Ouest	95.3	4.0	0.7	4.7	1127
Ville Abidjan	96.2	3.6	0.2	3.8	979

Equatorial Guinea 2000, regions	In range	Flagged	Missing	Flagged/missing	Total
	%	%	%	%	No.
Annobon	78.6	21.4	0.0	21.4	14
Bioko Norte	71.9	16.9	11.2	28.1	609
Bioko Sur	88.6	2.9	8.6	11.4	35
Centro Sur	78.7	15.7	5.6	21.3	267
Kie Ntem	85.6	8.5	5.9	14.4	506
Litoral	82.2	10.2	7.6	17.8	715
Wele Nzas	86.8	8.4	4.8	13.2	311

Gambia 2000, regions	In range	Flagged	Missing	Flagged/missing	Total
	%	%	%	%	No.
Banjul	69.7	11.1	19.2	30.3	479
Basse	77.9	9.1	13.0	22.1	462
Brikama	74.5	5.8	19.7	25.5	451
Janjabureh	77.8	12.1	10.1	22.2	365
Kanifing	65.5	10.5	24.0	34.5	484
Kerewan	87.3	5.3	7.5	12.7	455
Kuntaur	67.1	11.7	21.3	32.9	574
Mansakonko	83.1	6.4	10.5	16.9	362

Gambia 2005, regions	In range	Flagged	Missing	Flagged/missing	Total
	%	%	%	%	No.
Banjul	95.8	0.0	4.2	4.2	167
Basse	90.4	0.2	9.4	9.6	1161
Brikama	97.6	0.6	1.8	2.4	1390
Janjanburay	99.6	0.3	0.1	0.4	754
Kanifing	96.7	0.1	3.2	3.3	1461
Kerewan	97.1	1.7	1.2	2.9	863
Kuntaur	89.3	0.2	10.4	10.7	441
Mansakonko	97.8	0.5	1.7	2.2	404

Ghana 2006, regions	In range	Flagged	Missing	Flagged/missing	Total
	%	%	%	%	No.
Ashanti	93.0	2.3	4.7	7.0	426
Brong Ahafo	97.1	1.2	1.6	2.9	245
Central	96.2	1.1	2.7	3.8	263
Eastern	94.5	1.7	3.8	5.5	346
Greater Accra	90.9	1.5	7.6	9.1	330
Northern	94.1	1.5	4.4	5.9	595
Upper East	89.7	3.8	6.5	10.3	399
Upper West	94.7	1.6	3.7	5.3	377
Volta	89.8	1.2	9.0	10.2	245
Western	97.2	1.6	1.3	2.8	319

Ghana 2011, regions	In range	Flagged	Missing	Flagged/missing	Total
	%	%	%	%	No.
Asante	96.0	0.8	3.2	4.0	476
Brong Ahafo	96.8	1.0	2.2	3.2	410
Central	96.9	0.6	2.5	3.1	1009
Eastern	97.7	0.0	2.3	2.3	346
Greater Accra	96.0	0.3	3.8	4.0	400
Northern	97.2	1.0	1.8	2.8	2008
Upper East	96.5	1.9	1.6	3.5	997
Upper West	94.4	2.2	3.5	5.6	1157

Volta	98.5	0.7	0.7	1.5	402
Western	92.2	1.4	6.4	7.8	421

Guinea Bissau 2000, regions	In range	Flagged	Missing	Flagged/missing	Total
	%	%	%	%	No.
Bafatá	88.6	5.4	6.1	11.4	971
Biombo	97.0	2.0	1.0	3.0	301
Bolama/Bijagós	93.6	1.7	4.7	6.4	172
Cacheu	96.4	2.6	1.0	3.6	773
Gabú	85.2	9.2	5.6	14.8	772
Oio	95.1	3.7	1.1	4.9	1131
Quinará	91.2	8.4	0.4	8.8	239
Tombali	91.5	6.3	2.2	8.5	319
sab	90.0	3.0	7.0	10.0	1173

Guinea Bissau 2006, regions	In range	Flagged	Missing	Flagged/missing	Total
	%	%	%	%	No.
EST (Bafata e Gabu)	72.0	11.7	16.4	28.0	1519
NORD (Biombo, Cacheu e Oio)	75.9	9.5	14.6	24.1	2725
SAB Capital	66.2	8.0	25.8	33.8	1069
SUD (Bolama, Quinara e Tombali)	54.4	21.6	23.9	45.6	1257

Mauritania 2007, regions	In range	Flagged	Missing	Flagged/missing	Total
	%	%	%	%	No.
Adrar	85.7	2.4	11.9	14.3	335
Assaba	88.1	2.8	9.1	11.9	940
Brakna	88.1	3.2	8.7	11.9	884
Gorgol	86.8	3.1	10.1	13.2	936
Guidimagha	86.1	3.7	10.2	13.9	884
Hodh ECharghi	85.3	3.6	11.1	14.7	784
Hodh ELGharbi	91.2	1.5	7.3	8.8	736
Inchiri	90.8	4.6	4.6	9.2	130
Nouadhibou	92.0	2.8	5.2	8.0	600
Nouakchott	83.7	3.0	13.3	16.3	1507
Tagant	89.1	3.5	7.4	10.9	311
Tiris Zemmour	93.2	1.9	4.9	6.8	308
Trarza	92.2	1.8	6.1	7.8	626

Niger 2000, regions	In range	Flagged	Missing	Flagged/missing	Total
	%	%	%	%	No.
Diffa	92.6	2.9	4.4	7.4	68
Agadez	96.7	1.2	2.1	3.3	420
Dosso	92.6	3.2	4.2	7.4	618
Maradi	96.0	1.9	2.1	4.0	1207
Niamey	94.8	0.4	4.8	5.2	731
Tahoua	89.6	3.7	6.7	10.4	732
Tillaberi	95.8	1.4	2.8	4.2	644
Zinder	90.6	4.2	5.2	9.4	660

Nigeria 2007, regions	In range	Flagged	Missing	Flagged/missing	Total
	%	%	%	%	No.
Abia	91.8	5.3	2.9	8.2	340
Abuja FCT	85.4	9.7	4.9	14.6	445
Adamawa	77.4	16.3	6.3	22.6	411
Akwa-Ibom	89.7	5.3	4.9	10.3	487

Anambra	79.5	6.9	13.5	20.5	347
Bauchi	79.0	12.9	8.1	21.0	789
Bayelsa	89.2	4.1	6.7	10.8	510
Benue	87.3	5.1	7.6	12.7	526
Borno	77.8	16.7	5.5	22.2	473
Cross-Rivers	89.4	3.3	7.3	10.6	369
Delta	94.6	3.0	2.4	5.4	333
Ebonyi	81.7	9.6	8.7	18.3	469
Edo	90.8	3.3	5.9	9.2	391
Ekiti	92.0	5.4	2.5	8.0	276
Enugu	89.1	5.9	5.0	10.9	341
Gombe	73.5	17.7	8.8	26.5	434
Imo	76.3	18.1	5.6	23.7	270
Jigawa	83.7	14.9	1.5	16.3	821
Kaduna	88.3	10.1	1.6	11.7	681
Kano	75.4	22.2	2.3	24.6	598
Katsina	87.7	10.6	1.6	12.3	546
Kebbi	75.2	22.4	2.4	24.8	509
Kogi	90.5	5.2	4.3	9.5	326
Kwara	89.1	7.6	3.3	10.9	330
Lagos	81.5	11.4	7.1	18.5	351
Nasarawa	76.8	7.4	15.8	23.2	544
Niger	89.6	6.3	4.1	10.4	608
Ogun	88.3	6.0	5.7	11.7	299
Ondo	85.9	7.8	6.3	14.1	348
Osun	88.8	2.4	8.8	11.2	250
Oyo	88.8	5.3	5.9	11.2	358
Plataeu	85.7	10.6	3.7	14.3	463
Rivers	81.6	8.2	10.1	18.4	316
Sokoto	74.3	20.6	5.1	25.7	447
Taraba	78.9	13.3	7.8	21.1	525
Yobe	78.6	15.7	5.7	21.4	733
Zamfara	78.3	19.9	1.8	21.7	829

Nigeria 2011, regions	In range	Flagged	Missing	Flagged/missing	Total
	%	%	%	%	No.
Abia	98.3	0.4	1.3	1.7	465
Adamawa	88.7	0.6	10.7	11.3	875
Akwa ibom	96.5	0.4	3.2	3.5	564
Anambra	96.6	0.7	2.7	3.4	562
Bauchi	91.6	1.2	7.2	8.4	1001
Bayelsa	93.5	0.5	6.0	6.5	552
Benue	94.0	1.3	4.7	6.0	633
Borno	83.2	1.1	15.7	16.8	796
Cross River	92.7	0.7	6.6	7.3	593
Delta	94.7	0.5	4.7	5.3	552
Ebonyi	94.2	0.1	5.7	5.8	685
Edo	96.7	0.8	2.5	3.3	519
Ekiti	92.5	0.7	6.7	7.5	402
Enugu	97.7	0.3	2.0	2.3	352
FCT (Abuja)	92.7	0.3	7.0	7.3	646
Gombe	91.8	0.7	7.5	8.2	971
Imo	97.8	0.2	1.9	2.2	414
Jigawa	92.5	1.7	5.8	7.5	1063
Kaduna	93.7	2.5	3.8	6.3	962

Kano	92.6	1.5	6.0	7.4	956
Katsina	92.0	2.4	5.6	8.0	1024
Kebbi	89.8	1.6	8.7	10.2	947
Kogi	97.7	0.7	1.6	2.3	430
Kwara	96.1	0.5	3.4	3.9	564
Lagos	93.6	0.6	5.9	6.4	545
Nasarawa	83.2	0.2	16.6	16.8	891
Niger	92.3	1.8	6.0	7.7	957
Ogun	90.5	0.2	9.4	9.5	588
Ondo	93.2	0.9	5.9	6.8	427
Osun	97.1	0.0	2.9	2.9	456
Oyo	89.7	1.4	9.0	10.3	592
Plateau	92.0	0.6	7.4	8.0	687
Rivers	94.2	1.3	4.5	5.8	467
Sokoto	96.5	2.1	1.5	3.5	1020
Taraba	82.6	0.9	16.5	17.4	792
Yobe	90.5	1.4	8.1	9.5	1015
Zamfara	90.2	1.5	8.3	9.8	1053

**Sao Tome et Principe 2000, regions**	**In range**	**Flagged**	**Missing**	**Flagged/missing**	**Total**
	**%**	**%**	**%**	**%**	**No.**
Centro	74.9	3.0	22.2	25.1	1557
Norte	77.1	2.9	19.9	22.9	442
Principe	82.7	3.8	13.5	17.3	104
Sul	76.2	5.7	18.1	23.8	105

**Senegal 2000, regions**	**In range**	**Flagged**	**Missing**	**Flagged/missing**	**Total**
	**%**	**%**	**%**	**%**	**No.**
Dakar	88.4	2.3	9.3	11.6	1077
Diourbel	94.2	2.5	3.3	5.8	759
Fatick	94.1	1.7	4.3	5.9	908
Kaolack	93.2	3.5	3.3	6.8	968
Kolda	92.8	2.4	4.8	7.2	668
Louga	94.1	1.1	4.8	5.9	923
Saint louis	94.5	1.3	4.1	5.5	893
Tambacounda	91.0	2.9	6.1	9.0	897
Thies	95.4	1.1	3.5	4.6	1180
Ziguinchor	92.4	2.4	5.3	7.6	760

**Sierra Leone 2000, regions**	**In range**	**Flagged**	**Missing**	**Flagged/missing**	**Total**
	**%**	**%**	**%**	**%**	**No.**
East	77.9	8.7	13.4	22.1	701
North	77.3	5.6	17.1	22.7	1048
South	83.8	12.3	3.9	16.2	357
West	94.1	3.7	2.2	5.9	598

**Sierra Leone 2005, regions**	**In range**	**Flagged**	**Missing**	**Flagged/missing**	**Total**
	**%**	**%**	**%**	**%**	**No.**
East	69.1	5.6	25.3	30.9	1478
North	82.1	7.1	10.8	17.9	2273
South	84.7	4.6	10.7	15.3	1638
West	94.0	4.3	1.7	6.0	515

**Sierra Leone 2010, regions**	**In range**	**Flagged**	**Missing**	**Flagged/missing**	**Total**
	**%**	**%**	**%**	**%**	**No.**

East	85.5	6.0	8.4	14.5	1942
North	90.4	5.0	4.5	9.6	3310
South	87.9	6.0	6.1	12.1	2410
West	81.9	8.1	10.0	18.1	1136

Togo 2006, regions	In range	Flagged	Missing	Flagged/missing	Total
	%	%	%	%	No.
Centrale	96.3	2.3	1.3	3.7	601
Kara	90.5	6.3	3.2	9.5	601
Lomé commune	90.2	2.6	7.3	9.8	549
Maritime (sans Lomé commune)	93.5	3.5	3.0	6.5	831
Plateaux	89.3	3.2	7.6	10.7	569
Savanes	91.6	4.9	3.5	8.4	1003

Togo 2010, regions	In range	Flagged	Missing	Flagged/missing	Total
	%	%	%	%	No.
Centrale	94.7	0.5	4.8	5.3	778
Kara	95.0	0.5	4.5	5.0	863
Lomé	91.9	0.2	7.9	8.1	506
Maritime	93.3	0.2	6.4	6.7	825
Plateaux	96.2	0.5	3.3	3.8	816
Savanes	97.6	0.4	2.1	2.4	1120

Appendix C

Digit preference score for terminal digit of height, by region DHS

Benin 2001	N	Mean	Mean	Mean
	hgtdps	hgtdps	c2dhreg	pvdhreg
atacora	678	20.98	268.67	0.00
atlantique	954	28.61	702.56	0.00
borgou	785	28.28	564.92	0.00
mono	612	7.08	27.58	0.00
oueme	687	26.94	448.82	0.00
zou	801	26.91	522.21	0.00
Total	4517	23.93	451.49	0.00

Benin 2006	N	Mean	Mean	Mean
	hgtdps	hgtdps	c2dhreg	pvdhreg
alibori	1138	52.46	2818.27	0.00
atacora	1287	17.07	337.64	0.00
atlantique	1555	37.30	1947.32	0.00
borgou	1403	45.99	2670.76	0.00
collines	945	44.26	1666.08	0.00
couffo	954	55.58	2652.77	0.00
donga	704	46.76	1385.43	0.00
littoral	887	25.69	526.83	0.00
mono	831	31.56	744.99	0.00
plateau	676	38.33	893.88	0.00
quémé	1573	32.39	1485.25	0.00
zou	1459	23.85	746.78	0.00
Total	13412	36.73	1536.98	0.00

Benin 2011	N	Mean	Mean	Mean
	hgtdps	hgtdps	c2dhreg	pvdhreg
Alibori	793	85.92	5269.18	0.00
Atacora	1152	60.72	3822.64	0.00
Atlantique	1348	52.28	3315.53	0.00
Borgou	935	58.58	2887.71	0.00
Collines	749	58.32	2292.46	0.00
Couffo	910	31.49	811.89	0.00
Donga	636	76.88	3383.43	0.00
Littoral	835	63.87	3065.18	0.00
Mono	701	34.16	736.28	0.00
Ou?m?	1350	62.31	4717.38	0.00
Plateau	740	58.54	2282.11	0.00
Zou	1142	51.60	2736.93	0.00
Total	11291	57.41	3065.94	0.00

Burkina Faso 1993	N	Mean	Mean	Mean
	hgtdps	hgtdps	c2dhreg	pvdhreg
central/south	1055	9.90	93.05	0.00
east	826	21.34	338.65	0.00
north	715	14.85	141.85	0.00
ouagadougou	837	24.57	454.84	0.00

west	1141	14.44	214.21	0.00
Total	4574	16.56	241.46	0.00

Burkina Faso 1998	N	Mean	Mean	Mean
	hgtdps	hgtdps	c2dhreg	pvdhreg
central/south	1202	10.09	110.15	0.00
east	1338	15.06	273.02	0.00
north	630	11.36	73.17	0.00
ouagadougou	450	24.21	237.29	0.00
west	1134	19.47	386.86	0.00
Total	4754	15.23	229.13	0.00

Burkina Faso 2003	N	Mean	Mean	Mean
	hgtdps	hgtdps	c2dhreg	pvdhreg
boucle de mouhoun	684	20.87	268.13	0.00
cascades	580	43.38	982.45	0.00
centre (sans ouagadougou)	266	42.84	439.41	0.00
centre-est	612	23.37	300.71	0.00
centre-nord	742	27.60	508.73	0.00
centre-ouest	805	33.18	797.40	0.00
centre-sud	589	14.37	109.42	0.00
est	662	55.83	1857.03	0.00
hauts bassins	715	20.00	257.43	0.00
nord	668	31.09	581.28	0.00
ouagadougou	246	41.89	388.47	0.00
plateau central	830	14.36	154.14	0.00
sahel	565	16.19	133.35	0.00
sud-ouest	811	27.31	544.38	0.00
Total	8775	28.07	533.34	0.00

Burkina Faso 2010	N	Mean	Mean	Mean
	hgtdps	hgtdps	c2dhreg	pvdhreg
boucle de mouhoun	618	10.00	55.66	0.00
cascades	414	20.61	158.22	0.00
centre	380	14.44	71.26	0.00
centre-est	537	19.16	177.43	0.00
centre-nord	540	16.49	132.22	0.00
centre-ouest	592	8.43	37.83	0.00
centre-sud	426	10.53	42.54	0.00
est	631	15.00	127.75	0.00
hauts basins	577	24.34	307.70	0.00
nord	556	9.21	42.45	0.00
plateau central	464	15.20	96.47	0.00
sahel	514	11.26	58.65	0.00
sud-ouest	460	15.51	99.57	0.00
Total	6709	14.53	109.80	0.00

Cameroon 1991	N	Mean	Mean	Mean
	hgtdps	hgtdps	c2dhreg	pvdhreg
adam/nord/ext-nord	831	8.75	57.22	0.00
centre/sud/est	431	14.55	82.16	0.00
nord-ouest/sud-ouest	366	6.36	13.34	0.15
ouest/littoral	400	7.47	20.10	0.02
yaoundé/douala	652	18.93	210.33	0.00
Total	2680	11.64	86.95	0.02

Cameroon 1998	N	Mean	Mean	Mean
	hgtdps	hgtdps	c2dhreg	pvdhreg
central, south, & east	553	23.30	270.09	0.00
north/ extreme north/ ad	657	24.81	364.05	0.00
northwest & southwest	310	17.49	85.35	0.00
west & littoral	349	26.07	213.44	0.00
Total	1869	23.38	261.90	0.00

Cameroon 2011	N	Mean	Mean	Mean
	hgtdps	hgtdps	c2dhreg	pvdhreg
adamaoua	474	7.83	26.13	0.00
centre	404	4.49	7.34	0.60
douala	362	10.32	34.69	0.00
est	383	10.57	38.49	0.00
extrême-nord	755	7.90	42.44	0.00
littoral	272	14.16	49.10	0.00
nord	670	10.27	63.58	0.00
nord-ouest	489	9.09	36.38	0.00
ouest	467	12.53	65.96	0.00
sud	281	10.31	26.86	0.00
sud-ouest	307	8.43	19.61	0.02
yaoundé	305	10.60	30.84	0.00
Total	5169	9.48	39.12	0.05

Central African Rep 1994	N	Mean	Mean	Mean
	hgtdps	hgtdps	c2dhreg	pvdhreg
bangui	489	21.44	202.31	0.00
rs i	392	17.25	105.04	0.00
rs ii	419	14.83	82.89	0.00
rs iii	473	26.12	290.49	0.00
rs iv	341	27.27	228.18	0.00
rs v	319	19.33	107.30	0.00
Total	2433	21.08	174.38	0.00

Chad 1996	N	Mean	Mean	Mean
	hgtdps	hgtdps	c2dhreg	pvdhreg
b.e.t.	53	16.65	13.23	0.15
batha	243	16.73	61.24	0.00
biltine	133	32.52	126.62	0.00
chari-baguirmi	617	17.12	162.69	0.00
guéra	214	14.06	38.06	0.00
kanem	215	13.85	37.14	0.00
lac	216	35.45	244.28	0.00
logone occidental	397	21.65	167.41	0.00
logone oriental	376	28.09	266.98	0.00
mayo-kebbi	621	18.10	183.11	0.00
moyen chari	643	20.28	238.04	0.00
n'djaména	950	29.86	762.32	0.00
ouaddaï	520	25.11	295.00	0.00
salamat	216	22.95	102.43	0.00
tandjilé	423	33.20	419.62	0.00
Total	5837	23.53	293.39	0.00

Chad 2004	N	Mean	Mean	Mean

	hgtdps	hgtdps	c2dhreg	pvdhreg
b. e. t.	485	22.90	229.00	0.00
bar azoum	430	27.41	290.74	0.00
centre est	401	41.26	614.34	0.00
chari baguirmi	453	18.17	134.62	0.00
logone occidental	560	17.03	146.11	0.00
mayo kebbi	524	22.61	241.00	0.00
moyen chari	453	15.63	99.56	0.00
n'djaména	923	29.44	720.22	0.00
ouaddai est	395	36.08	462.90	0.00
Total	4624	25.42	355.59	0.00

Congo 2005	N	Mean	Mean	Mean
	hgtdps	hgtdps	c2dhreg	pvdhreg
brazzaville	974	46.56	1900.02	0.00
nord	950	27.96	668.17	0.00
pointe noire	796	55.11	2175.53	0.00
sud	1317	47.82	2710.83	0.00
Total	4037	44.28	1928.97	0.00

Congo 2012	N	Mean	Mean	Mean
	hgtdps	hgtdps	c2dhreg	pvdhreg
bouenza	436	11.05	47.90	0.00
brazzaville	306	20.60	116.88	0.00
cuvette	335	9.14	25.21	0.00
cuvette - ouest	272	12.22	36.53	0.00
kouilou	455	10.17	42.34	0.00
lekoumou	329	20.73	127.26	0.00
likouala	445	8.28	27.43	0.00
niari	412	15.85	93.10	0.00
plateaux	341	9.58	28.18	0.00
pointe-noire	432	4.77	8.83	0.45
pool	475	11.16	53.27	0.00
sangha	287	12.39	39.66	0.00
Total	4525	11.79	52.34	0.04

Congo DR 2007	N	Mean	Mean	Mean
	hgtdps	hgtdps	c2dhreg	pvdhreg
bandundu	361	30.89	310.11	0.00
bas-congo	248	34.01	258.13	0.00
equateur	336	46.27	647.51	0.00
kasaï occident	329	45.40	610.36	0.00
kasaï oriental	357	40.94	538.43	0.00
katanga	357	42.05	568.13	0.00
kinshasa	363	28.31	261.77	0.00
maniema	346	24.94	193.65	0.00
nord-kivu	343	29.35	265.83	0.00
orientale	274	37.60	348.70	0.00
sud-kivu	317	41.26	485.81	0.00
Total	3631	36.37	410.94	0.00

Côte d'Ivoire 1994	N	Mean	Mean	Mean
	hgtdps	hgtdps	c2dhreg	pvdhreg
center	325	13.24	51.28	0.00
center east	145	8.68	9.83	0.36

center north	317	20.87	124.29	0.00
center west	569	17.60	158.64	0.00
north	250	12.41	34.64	0.00
north east	184	37.30	230.35	0.00
north west	216	10.83	22.80	0.01
south	912	26.56	579.10	0.00
south west	246	26.21	152.13	0.00
west	343	33.85	353.76	0.00
Total	3507	21.89	253.96	0.02

**Côte d'Ivoire 1998**	**N**	**Mean**	**Mean**	**Mean**
	**hgtdps**	**hgtdps**	**c2dhreg**	**pvdhreg**
capital (abidjan)	433	25.88	261.06	0.00
countryside	659	16.43	160.20	0.00
small city	497	40.25	724.51	0.00
Total	1589	26.46	364.19	0.00

**Côte d'Ivoire 2011**	**N**	**Mean**	**Mean**	**Mean**
	**hgtdps**	**hgtdps**	**c2dhreg**	**pvdhreg**
Centre	277	8.59	18.42	0.03
Centre-Est	258	9.68	21.77	0.01
Centre-Nord	360	8.26	22.11	0.01
Centre-Ouest	300	42.30	483.20	0.00
Nord	326	10.87	34.67	0.00
Nord-Ouest	428	10.78	44.80	0.00
Nord-est	274	20.99	108.63	0.00
Ouest	359	16.49	87.82	0.00
Sud sans Abidjan	194	22.63	89.40	0.00
Sud-ouest	268	11.99	34.69	0.00
Ville d'Abidjan	245	24.87	136.35	0.00
Total	3289	16.44	95.91	0.00

**Gabon 2000**	**N**	**Mean**	**Mean**	**Mean**
	**hgtdps**	**hgtdps**	**c2dhreg**	**pvdhreg**
east (haut-ogooué & ogooué-lolo)	859	11.70	105.88	0.00
libreville,port-gentil	820	18.32	247.59	0.00
north (ogooué-ivindo & woleu-ntem)	624	12.89	93.24	0.00
south (ngounié, nyanga)	681	13.84	117.43	0.00
west (estuaire, moyen-ogooué & ogooué-maritime)	574	17.85	164.68	0.00
Total	3558	14.84	148.02	0.00

**Gabon 2012**	**N**	**Mean**	**Mean**	**Mean**
	**hgtdps**	**hgtdps**	**c2dhreg**	**pvdhreg**
estuaire	286	14.00	50.43	0.00
haut-ogooué	407	16.14	95.38	0.00
libreville-port-gentil	470	17.38	127.74	0.00
moyen-ogooué	267	10.71	27.57	0.00
ngounié	413	10.48	40.83	0.00
nyanga	303	13.45	49.31	0.00
ogooué maritime	244	10.69	25.10	0.00
ogooué-ivindo	534	12.98	80.98	0.00
ogooué-lolo	307	18.64	96.03	0.00
woleu-ntem	239	16.76	60.46	0.00
Total	3470	14.20	70.82	0.00

Ghana 1993	N hgtdps	Mean hgtdps	Mean c2dhreg	Mean pvdhreg
ashanti	355	19.49	121.42	0.00
brong-ahafo	192	17.27	51.54	0.00
central	207	19.24	68.99	0.00
eastern	211	8.81	14.73	0.10
greater accra	182	11.40	21.30	0.01
northern	217	10.28	20.65	0.01
upper east	140	17.27	37.57	0.00
upper west	71	25.23	40.69	0.00
volta	214	14.89	42.73	0.00
western	175	17.00	45.51	0.00
Total	1964	15.66	52.96	0.01

Ghana 1998	N hgtdps	Mean hgtdps	Mean c2dhreg	Mean pvdhreg
ashanti region	395	10.25	37.38	0.00
brong ahafo region	189	15.32	39.94	0.00
central region	253	9.95	22.53	0.01
eastern region	329	17.91	94.98	0.00
greater accra region	246	21.51	102.46	0.00
northern region	289	13.26	45.71	0.00
upper east region	334	8.73	22.89	0.01
upper west region	230	16.76	58.17	0.00
volta region	252	22.34	113.16	0.00
western region	311	14.92	62.34	0.00
Total	2828	14.68	58.91	0.00

Ghana 2003	N hgtdps	Mean hgtdps	Mean c2dhreg	Mean pvdhreg
ashanti	482	10.08	44.06	0.00
brong ahafo	385	13.98	67.70	0.00
central	213	20.38	79.63	0.00
eastern	277	15.54	60.22	0.00
greater accra	286	12.54	40.50	0.00
northern	512	13.12	79.29	0.00
upper east	208	12.45	29.02	0.00
upper west	301	8.02	17.44	0.04
volta	236	12.20	31.63	0.00
western	296	18.61	92.24	0.00
Total	3196	13.32	56.06	0.00

Ghana 2008	N hgtdps	Mean hgtdps	Mean c2dhreg	Mean pvdhreg
ashanti	383	15.62	84.08	0.00
brong ahafo	241	13.44	39.21	0.00
central	181	10.43	17.73	0.04
eastern	216	16.94	55.76	0.00
greater accra	231	17.65	64.80	0.00
northern	389	13.88	67.43	0.00
upper east	187	22.05	81.82	0.00
upper west	236	12.24	31.80	0.00
volta	216	14.25	39.46	0.00
western	229	11.69	28.16	0.00
Total	2509	14.75	54.16	0.00

Guinea 1999	N hgtdps	Mean hgtdps	Mean c2dhreg	Mean pvdhreg
central guinea	777	10.06	70.79	0.00
conakry	657	9.19	49.96	0.00
forest guinea	1244	23.13	598.94	0.00
lower guinea	971	9.56	79.84	0.00
upper guinea	946	21.03	376.52	0.00
Total	4595	15.63	275.65	0.00

Guinea 2005	N hgtdps	Mean hgtdps	Mean c2dhreg	Mean pvdhreg
boké	333	22.77	155.38	0.00
conakry	200	27.76	138.70	0.00
faranah	382	28.67	282.55	0.00
kankan	491	12.31	66.98	0.00
kindia	395	16.61	98.09	0.00
labé	260	23.13	125.23	0.00
mamou	272	25.88	163.96	0.00
n'zérékoré	406	27.03	267.00	0.00
Total	2739	22.17	162.33	0.00

Guinea 2012	N hgtdps	Mean hgtdps	Mean c2dhreg	Mean pvdhreg
Bok?	335	9.33	26.22	0.00
Conakry	302	15.60	66.15	0.00
Faranah	469	10.32	44.92	0.00
Kankan	573	9.53	46.88	0.00
Kindia	388	14.29	71.33	0.00
Lab?	364	12.74	53.20	0.00
Mamou	347	30.67	293.84	0.00
N'Z?r?kor?	417	9.03	30.60	0.00
Total	3195	13.37	74.63	0.00

Liberia 2007	N hgtdps	Mean hgtdps	Mean c2dhreg	Mean pvdhreg
monrovia	753	10.62	76.44	0.00
north central	937	9.85	81.88	0.00
north western	587	9.12	43.92	0.00
south central	729	10.78	76.23	0.00
south eastern a	659	11.77	82.23	0.00
south eastern b	882	14.14	158.61	0.00
Total	4547	11.14	90.11	0.00

Mali 1995	N hgtdps	Mean hgtdps	Mean c2dhreg	Mean pvdhreg
bamako	496	20.46	186.86	0.00
gao	259	15.31	54.63	0.00
kayes	714	14.49	134.85	0.00
koulikoro	929	13.72	157.38	0.00
mopti	563	38.71	759.33	0.00
sikasso	946	17.10	248.95	0.00
ségou	843	21.48	350.06	0.00
timbuktu	251	11.57	30.24	0.00
Total	5001	19.23	262.95	0.00

Mali 2001	N hgtdps	Mean hgtdps	Mean c2dhreg	Mean pvdhreg
bamako	1049	16.95	271.31	0.00
gao	395	19.87	140.32	0.00
kayes	1513	15.83	341.18	0.00
kidal	142	20.81	55.32	0.00
koulikoro	1662	9.53	135.80	0.00
mopti	1297	13.47	211.78	0.00
segou	1424	7.63	74.58	0.00
sikasso	1998	16.70	501.28	0.00
tombouctou	366	11.61	44.38	0.00
Total	9846	13.64	252.74	0.00

Mali 2006	N hgtdps	Mean hgtdps	Mean c2dhreg	Mean pvdhreg
bamako	1166	22.97	553.55	0.00
gao	794	18.27	238.42	0.00
kayes	1462	20.63	560.13	0.00
kidal	266	24.99	149.56	0.00
koulikoro	1560	19.58	538.35	0.00
mopti	1793	28.56	1316.42	0.00
segou	1392	24.32	740.97	0.00
sikasso	2330	26.29	1448.94	0.00
tombouctou	814	41.36	1252.95	0.00
Total	11577	24.93	891.51	0.00

Niger 1992	N hgtdps	Mean hgtdps	Mean c2dhreg	Mean pvdhreg
agadez	201	20.37	75.07	0.00
diffa	121	18.58	37.60	0.00
dosso	548	6.29	19.52	0.02
maradi	830	6.86	35.18	0.00
niamey	891	15.48	192.15	0.00
tahoua	802	11.72	99.12	0.00
tillabéri	675	9.64	56.45	0.00
zinder	808	10.17	75.27	0.00
Total	4876	10.95	83.91	0.00

Niger 1998	N hgtdps	Mean hgtdps	Mean c2dhreg	Mean pvdhreg
dosso	595	8.52	38.83	0.00
maradi	969	11.56	116.56	0.00
niamey	388	12.94	58.49	0.00
tahoua/agadez	678	11.24	77.10	0.00
tillaberi	739	10.52	73.60	0.00
zinda/diffa	665	14.07	118.44	0.00
Total	4034	11.41	85.32	0.00

Niger 2006	N hgtdps	Mean hgtdps	Mean c2dhreg	Mean pvdhreg
agadez	270	36.04	315.63	0.00
diffa	327	23.97	169.06	0.00
dosso	633	37.63	806.76	0.00
maradi	620	30.46	517.71	0.00

niamey	414	34.30	438.27	0.00
tahoua	640	29.42	498.47	0.00
tillabéri	533	14.08	95.12	0.00
zinder	427	19.11	140.33	0.00
Total	3864	28.20	409.74	0.00

Niger 2012	N	Mean	Mean	Mean
	hgtdps	hgtdps	c2dhreg	pvdhreg
Agadez	275	57.65	822.71	0.00
Diffa	426	30.29	351.75	0.00
Dosso	752	14.47	141.64	0.00
Maradi	1115	37.16	1385.60	0.00
Niamey	397	36.02	463.63	0.00
Tahoua	830	26.19	512.29	0.00
Tillaberi	687	10.37	66.44	0.00
Zinder	654	47.44	1324.56	0.00
Total	5136	30.23	690.95	0.00

Nigeria 1990	N	Mean	Mean	Mean
	hgtdps	hgtdps	c2dhreg	pvdhreg
northeast	1460	13.14	226.74	0.00
northwest	1380	13.94	241.33	0.00
southeast	1659	10.41	161.84	0.00
southwest	1644	12.62	235.56	0.00
Total	6143	12.44	214.85	0.00

Nigeria 2003	N	Mean	Mean	Mean
	hgtdps	hgtdps	c2dhreg	pvdhreg
north central	834	20.25	307.85	0.00
north east	1096	19.00	356.17	0.00
north west	1416	27.24	945.69	0.00
south east	433	19.88	154.04	0.00
south south	439	20.10	159.70	0.00
south west	549	25.24	314.77	0.00
Total	4767	22.57	481.61	0.00

Nigeria 2008	N	Mean	Mean	Mean
	hgtdps	hgtdps	c2dhreg	pvdhreg
north central	4092	35.97	4764.66	0.00
north east	5209	21.77	2222.68	0.00
north west	6093	29.03	4620.52	0.00
south east	1910	30.06	1553.11	0.00
south south	2662	26.20	1644.77	0.00
south west	2850	22.05	1247.45	0.00
Total	22816	27.50	3073.62	0.00

Sao Tome et Principe 2008	N	Mean	Mean	Mean
	hgtdps	hgtdps	c2dhreg	pvdhreg
região centro	476	55.96	1341.71	0.00
região do principe	284	42.24	456.05	0.00
região norte	487	78.84	2724.29	0.00
região sul	452	82.52	2769.92	0.00
Total	1699	67.29	1969.93	0.00

Senegal 1992	N hgtdps	Mean hgtdps	Mean c2dhreg	Mean pvdhreg
central	1753	11.07	193.19	0.00
north east	809	11.95	103.97	0.00
south	547	6.75	22.45	0.01
west	1543	8.23	94.12	0.00
Total	4652	9.77	124.74	0.00

Senegal 2005	N hgtdps	Mean hgtdps	Mean c2dhreg	Mean pvdhreg
dakar	187	17.17	49.63	0.00
diourbel	313	12.47	43.77	0.00
fatick	237	12.15	31.48	0.00
kaolack	341	10.46	33.57	0.00
kolda	332	19.26	110.89	0.00
louga	257	13.55	42.49	0.00
matam	226	9.17	17.10	0.05
saint-louis	222	9.20	16.92	0.05
tambacounda	293	12.56	41.57	0.00
thiès	340	6.58	13.24	0.15
zuguinchor	184	20.52	69.70	0.00
Total	2932	12.70	43.23	0.03

Senegal 2010	N hgtdps	Mean hgtdps	Mean c2dhreg	Mean pvdhreg
dakar	255	19.68	88.88	0.00
diourbel	310	27.21	206.52	0.00
fatick	321	32.75	309.87	0.00
kaffrine	331	30.17	271.21	0.00
kaolack	395	26.51	249.89	0.00
kedougou	118	15.31	24.88	0.00
kolda	340	22.61	156.41	0.00
louga	301	22.97	142.89	0.00
matam	249	21.29	101.57	0.00
saint-louis	244	33.33	243.95	0.00
sedhiou	286	26.21	176.87	0.00
tambacounda	269	13.76	45.83	0.00
thiès	322	14.65	62.16	0.00
ziguinchor	178	26.19	109.87	0.00
Total	3919	24.20	168.68	0.00

Sierra Leone 2008	N hgtdps	Mean hgtdps	Mean c2dhreg	Mean pvdhreg
eastern	583	11.68	71.56	0.00
northern	788	20.11	286.92	0.00
southern	559	24.40	299.44	0.00
western	340	12.42	47.24	0.00
Total	2270	17.85	198.80	0.00

Togo 1998	N hgtdps	Mean hgtdps	Mean c2dhreg	Mean pvdhreg
centrale	582	9.53	47.59	0.00
kara	601	13.59	99.88	0.00
lomé	299	30.55	251.13	0.00
marities	504	9.38	39.93	0.00

plateaux	789	13.96	138.39	0.00
savanes	995	8.17	59.70	0.00
Total	3770	12.39	93.24	0.00

Digit preference score for terminal digit of height, by region NNS

Benin 2008	N	Mean	Mean	Mean
	hgtdps	hgtdps	c2dhreg	pvdhreg
alibori	368	10.75	38.3	0
atacora	346	21.6	145.27	0
atlantique	305	7.34	14.77	0.1
borgou	323	8.9	23.04	0.01
collines	247	28.93	186.08	0
couffo	392	6.8	16.32	0.06
donga	258	21.03	102.7	0
littoral	218	13.1	33.65	0
mono	242	6.18	8.33	0.5
oueme	264	10.23	24.86	0
plateau	225	7.79	12.29	0.2
zou	235	10.94	25.3	0
Total	3423	12.61	52.52	0.07

Burkina Faso 2012	N	Mean	Mean	Mean
	hgtdps	hgtdps	c2dhreg	pvdhreg
bales	773	1	0.7	1
bam	822	3.01	6.69	0.67
banwa	614	1.65	1.5	1
banwa ganzourgou	835	3.17	7.57	0.58
bazega	592	3.09	5.09	0.83
boulgou	683	4.28	11.25	0.26
cascades	640	2	2.31	0.99
centre ouest	580	3.08	4.97	0.84
est	920	1.99	3.28	0.95
houet	474	2.85	3.47	0.94
kadiogo	427	4.59	8.11	0.52
kenedougou	752	3.04	6.24	0.72
kossi	745	4.56	13.94	0.12
koulpelogo	825	1.94	2.79	0.97
kouritenga	706	5.3	17.85	0.04
kourweogo	653	2.54	3.8	0.92
mouhoun	637	3.21	5.9	0.75
nahouri	497	1.5	1.01	1
namentenga	876	4.57	16.49	0.06
nayala	642	4.84	13.51	0.14
nord	716	2.48	3.97	0.91
oubritenga	653	3.57	7.51	0.58
sahel	835	1.6	1.92	0.99
sanmentenga	857	3.4	8.92	0.45
sourou	657	5.54	18.18	0.03
sud ouest	496	2.21	2.19	0.99
tuy	560	0.84	0.36	1
zoundweogo	608	0.59	0.19	1
Total	19075	2.96	6.64	0.67

Cameroon 2011	N	Mean	Mean	Mean
	hgtdps	hgtdps	c2dhreg	pvdhreg
extrême-nord	645	6.19	22.24	0.01

nord	672	12.4	92.97	0
Total	1317	9.36	58.33	0

**Central African Rep 2012**	**N**	**Mean**	**Mean**	**Mean**
	**hgtdps**	**hgtdps**	**c2dhreg**	**pvdhreg**
bamingui bangoran	884	10.8	92.86	0
bangui	807	5.99	26.05	0
basse kotto	930	3.53	10.45	0.32
haut kotto	974	7.88	54.46	0
haut mbomou	945	2.86	6.97	0.64
kemo	928	4.53	17.13	0.05
lobaye	1091	8.02	63.23	0
mambere kadei	1284	5.23	31.62	0
mbomou	982	8.67	66.37	0
nana grebizi	1072	5.08	24.92	0
nana mambere	651	6.28	23.12	0.01
ombella mpoko	882	9.16	66.55	0
ouaka	804	8.08	47.24	0
ouham	985	9.27	76.21	0
ouham pende	779	4.33	13.16	0.16
sangha mbarere	1083	8.25	66.39	0
vakaga	501	6.92	21.57	0.01
Total	15582	6.76	42.97	0.07

**Chad June 2012**	**N**	**Mean**	**Mean**	**Mean**
	**hgtdps**	**hgtdps**	**c2dhreg**	**pvdhreg**
Barh El Ghazal	719	2.86	5.3	0.81
Batha	690	4.3	11.51	0.24
Guéra	733	2.24	3.3	0.95
Hadjer Lamis	606	5.22	14.86	0.09
Kanem	562	7.46	28.14	0
Lac	530	3.02	4.34	0.89
N’Djamena	744	8.23	45.41	0
Ouaddai	590	2.76	4.03	0.91
Salamat	722	8.59	48	0
Sila	686	3.5	7.56	0.58
Wadi Fira	721	7.44	35.88	0
Total	7303	5.11	19.6	0.4

**Chad (7 regions) Dec/Jan 2012-13**	**N**	**Mean**	**Mean**	**Mean**
	**hgtdps**	**hgtdps**	**c2dhreg**	**pvdhreg**
Logone Occidental	793	3.79	10.27	0.33
Logone Oriental	784	5.98	25.21	0
Mandoul	807	10.08	73.73	0
Mayo-Kebbi Est	983	5.28	24.68	0
Mayo-Kebbi Ouest	619	6.85	26.15	0
Moyen-Chari	699	5.24	17.27	0.04
Tandjilé	898	8.44	57.57	0
Total	5583	6.54	34.32	0.05

**The Gambia 2012**	**N**	**Mean**	**Mean**	**Mean**
	**hgtdps**	**hgtdps**	**c2dhreg**	**pvdhreg**
banjul	717	6.67	28.68	0
basse	1314	4.94	28.91	0
brikama	831	4.94	18.28	0.03

janjanburay	1007	5.97	32.26	0
kanifing	746	6.6	29.28	0
kerewan	1105	5.14	26.25	0
kuntaur	1075	8.85	75.86	0
mansakonko	831	7.44	41.36	0
Total	7626	6.25	35.8	0

**Guinea-Bissau 2008**	**N**	**Mean**	**Mean**	**Mean**
	**hgtdps**	**hgtdps**	**c2dhreg**	**pvdhreg**
Capitale	443	8.02	25.65	0
Est (Bafata e Gabu)	836	8.22	50.79	0
Nord (Biombo, Cacheu e Oio)	752	7.51	38.13	0
Sud (Bolama, Quinara e Tombali)	685	5.42	18.11	0.03
Total	2716	7.28	34.94	0.01

**Guinée Conakay 2012**	**N**	**Mean**	**Mean**	**Mean**
	**hgtdps**	**hgtdps**	**c2dhreg**	**pvdhreg**
boke nord	855	8.76	59.02	0
boke sud	706	4.4	12.3	0.2
conakry	881	10.36	85.03	0
faranah	699	5.4	18.32	0.03
kankan	839	5.72	24.68	0
kindia	880	6.29	31.36	0
labe	1080	3.85	14.44	0.11
mamou	1122	8.2	67.95	0
nzerekore	1100	7.62	57.42	0
Total	8162	6.81	42.9	0.03

**Liberia 2010**	**N**	**Mean**	**Mean**	**Mean**
	**hgtdps**	**hgtdps**	**c2dhreg**	**pvdhreg**
bomi	334	10.08	30.55	0
bong	476	12.17	63.5	0
gbarpolu	369	12.14	48.91	0
grand bassa	404	7.55	20.75	0.01
grand cape mount	500	11.92	63.96	0
grand gedeh	442	6.49	16.78	0.05
grand kru	449	7.22	21.09	0.01
lofa	480	6.7	19.42	0.02
margibi	385	15.55	83.75	0
maryland	373	10.77	38.93	0
montserrado	350	12.93	52.69	0
nimba	441	10.52	43.92	0
river gee	418	8.49	27.12	0
rivercess	467	5.5	12.72	0.18
rural montserrado	421	9.73	35.84	0
sinoe	487	16.19	114.95	0
Total	6796	10.19	43.84	0.02

**Liberia 2011**	**N**	**Mean**	**Mean**	**Mean**
	**hgtdps**	**hgtdps**	**c2dhreg**	**pvdhreg**
North Central	266	12.33	36.41	0
North Western	92	20.23	33.87	0
South Central	1002	10.12	92.29	0
South Eastern	119	12.11	15.71	0.07
Total	1479	11.3	72.44	0.01

Mali 2011	N hgtdps	Mean hgtdps	Mean c2dhreg	Mean pvdhreg
bamako	1201	8.4	76.21	0
gao	1129	5.27	28.23	0
kayes	1023	3.7	12.63	0.18
kidal	233	4.7	4.64	0.86
koulikoro	891	4.22	14.26	0.11
mopti	1011	10.24	95.4	0
segou	664	3.51	7.36	0.6
sikasso	1179	5.57	32.88	0
tombouctou	680	8.62	45.5	0
Total	8011	6.21	40.09	0.11

Mauritania 2006	N hgtdps	Mean hgtdps	Mean c2dhreg	Mean pvdhreg
Centre	410	18.88	131.51	0
Fleuve	830	17.39	225.88	0
Nord	229	14.19	41.52	0
Nouakchott	1850	19.58	638.53	0
SudEst	524	17.19	139.28	0
Total	3843	18.39	391.67	0

Mauritania March 2008	N hgtdps	Mean hgtdps	Mean c2dhreg	Mean pvdhreg
Centre	947	39.57	1334.62	0
Fleuve Nord	1327	14.38	246.89	0
Fleuve Sud	1011	26.59	643.17	0
Nord	490	14.58	93.76	0
Nouakchott	1375	8.65	92.69	0
SudEst	1221	29.84	978.58	0
Total	6371	21.8	566.63	0

Mauritania Dec 2008	N hgtdps	Mean hgtdps	Mean c2dhreg	Mean pvdhreg
Centre	956	9.24	73.52	0
Nord	710	7.75	38.39	0
Nouakchott A	802	3.92	11.12	0.27
Nouakchott B	926	5.29	23.33	0.01
Sud	1072	5.42	28.32	0
SudEst	988	4.37	16.98	0.05
Trarza	881	5.02	19.96	0.02
Total	6335	5.83	30.43	0.05

Mauritania 2009	N hgtdps	Mean hgtdps	Mean c2dhreg	Mean pvdhreg
Centre	638	7.15	29.37	0
Nord	688	5.69	20.05	0.02
Nouakchott	679	4.06	10.09	0.34
Sud	965	6.24	33.79	0
Sud-est	699	8.13	41.56	0
Trarza	709	5.52	19.48	0.02
Total	4378	6.13	26.23	0.06

Mauritania July 2010	N hgtdps	Mean hgtdps	Mean c2dhreg	Mean pvdhreg
adrar/inchiri tiris	441	10.09	40.43	0
assaba	755	6.7	30.47	0
brakna	576	10.92	61.81	0
gorgol	723	6.17	24.73	0
guidimakha	632	13.99	111.32	0
hodh chargui	485	10.98	52.59	0
hodh gharbi	531	7.93	30.04	0
nouadhibou	505	6.2	17.48	0.04
nouakchott	505	6.28	17.95	0.04
tagant	646	5.74	19.17	0.02
trarza	584	8.8	40.69	0
Total	6383	8.43	40.76	0.01

Mauritania Dec 2010	N hgtdps	Mean hgtdps	Mean c2dhreg	Mean pvdhreg
adrar/inchiri tiris	408	5.96	13.03	0.16
assaba	556	11.97	71.7	0
brakna	567	7.05	25.36	0
gorgol	553	6.89	23.65	0
guidimakha	600	3.4	6.23	0.72
hodh chargui	471	8.76	32.55	0
hodh gharbi	551	17.21	146.95	0
nouadhibou	562	4.23	9.07	0.43
nouakchott	517	5.53	14.24	0.11
tagant	589	11.12	65.52	0
trarza	476	5.86	14.71	0.1
Total	5850	8.06	39.44	0.15

Mauritania July 2011	N hgtdps	Mean hgtdps	Mean c2dhreg	Mean pvdhreg
adrar/inchiri tiris	677	22.81	316.99	0
assaba	706	13.57	117	0
brakna	743	7.2	34.62	0
gorgol	680	5.37	17.65	0.04
guidimakha	767	17.59	213.61	0
hodh chargui	647	15.54	140.62	0
hodh gharbi	775	6.07	25.74	0
nouadhibou	674	6.33	24.31	0
nouakchott	645	5.65	18.55	0.03
tagant	741	3.54	8.38	0.5
trarza	718	5.26	17.85	0.04
Total	7773	9.85	84.53	0.06

Mauritania Dec 2011	N hgtdps	Mean hgtdps	Mean c2dhreg	Mean pvdhreg
adrar/inchiri tiris	690	8.23	42.06	0
assaba	763	5.39	19.98	0.02
brakna	701	7.18	32.48	0
gorgol	791	14.09	141.33	0
guidimakha	855	11.37	99.47	0
hodh chargui	628	7.47	31.55	0
hodh gharbi	803	2.84	5.83	0.76
nouadhibou	716	1.2	0.93	1

nouakchott	710	8.34	44.39	0
tagant	789	6.2	27.26	0
trarza	726	15.73	161.69	0
Total	8172	8.05	56.22	0.16

**Mauritania July 2012**	**N hgtdps**	**Mean hgtdps**	**Mean c2dhreg**	**Mean pvdhreg**
adrar/inchiri tiris	450	7.82	24.76	0
assaba	640	11.86	81	0
brakna	573	10.54	57.28	0
gorgol	610	9.11	45.61	0
guidimakha	856	10.18	79.77	0
hodh chargui	670	7.28	32	0
hodh gharbi	742	7.89	41.56	0
nouadhibou	474	9.98	42.5	0
nouakchott	590	8.5	38.37	0
tagant	755	5.03	17.23	0.05
trarza	456	4.5	8.3	0.5
Total	6816	8.47	44.56	0.04

**Niger 2012**	**N hgtdps**	**Mean hgtdps**	**Mean c2dhreg**	**Mean pvdhreg**
agadez	1221	7.37	59.71	0
diffa	1294	6.58	50.45	0
dosso	1110	4.96	24.59	0
maradi	1087	5.84	33.4	0
niamey	1156	3.04	9.62	0.38
tahoua	1465	4.07	21.86	0.01
tillabéri	895	5.72	26.35	0
zinder	924	4.13	14.18	0.12
Total	9152	5.22	30.77	0.06

**Nigeria (Northern States) 2011**	**N hgtdps**	**Mean hgtdps**	**Mean c2dhreg**	**Mean pvdhreg**
jigawa	903	6.19	31.12	0
kano	841	6.03	27.5	0
katsina	954	4.95	21.05	0.01
kebbi	993	5.68	28.84	0
sokoto	908	5.45	24.29	0
yobe	1048	5.36	27.08	0
zamfara	956	4.81	19.88	0.02
Total	6603	5.48	25.65	0.01

**Senegal 2012**	**N hgtdps**	**Mean hgtdps**	**Mean c2dhreg**	**Mean pvdhreg**
dakar	390	7.16	18	0.04
kolda	549	10.75	57.07	0
matam	1942	5.02	44	0
myf	719	4.84	15.14	0.09
sedhiou	2059	3.78	26.48	0
tambacounda	2950	3.96	41.6	0
velingara	607	8.35	38.06	0
Total	9216	5.04	36.35	0.01

**Sierra Leone 2010**	**N**	**Mean**	**Mean**	**Mean**

	hgtdps	hgtdps	c2dhreg	pvdhreg
eastern	2685	5.07	62.19	0
northern	5024	0.72	2.32	0.99
southern	3967	2	14.31	0.11
western	2264	5.28	56.76	0
Total	13940	2.66	26.11	0.39

Togo June 2012	N	Mean	Mean	Mean
	hgtdps	hgtdps	c2dhreg	pvdhreg
Centrale	403	5.06	9.28	0.41
Kara	587	2.79	4.12	0.9
Lomé	316	6.14	10.71	0.3
Maritime	458	5.63	13.05	0.16
Plateaux	359	6.83	15.07	0.09
Savanes	540	3.36	5.48	0.79
Total	2663	4.68	8.97	0.5

Togo Dec 2012	N	Mean	Mean	Mean
	hgtdps	hgtdps	c2dhreg	pvdhreg
Kara	612	5.14	14.57	0.1
Savanes	788	4.74	15.96	0.07
Total	1400	4.92	15.35	0.08

Digit preference score for terminal digit of height, by region MICS

Burkina Faso 2006	N	Mean	Mean	Mean
	hgtdps	hgtdps	c2dhreg	pvdhreg
Boucle du Mouhoun	386	16.6	95.71	0.00
Cascade	575	17.07	150.79	0.00
Centre	443	23.63	222.71	0.00
Centre-Est	314	48.04	652.18	0.00
Centre-Nord	371	39.45	519.54	0.00
Centre-Ouest	472	39.53	663.93	0.00
Centre-Sud	345	42.34	556.71	0.00
Est	401	37.95	519.72	0.00
Hauts-Bassins	311	20.55	118.16	0.00
Nord	440	47.32	886.59	0.00
Plateau-Central	398	35.07	440.54	0.00
Sahel	362	54.27	959.44	0.00
Sud-Ouest	283	29.28	218.31	0.00
Total	5101	34.11	459.71	0.00

Cameroon 2006	N	Mean	Mean	Mean
	hgtdps	hgtdps	c2dhreg	pvdhreg
Adamaoua	626	9.88	54.99	0.00
Centre	522	37.41	657.62	0.00
Douala	353	33.79	362.84	0.00
Est	646	16.49	158.09	0.00
Extreme Nord	713	12.37	98.21	0.00
Littoral	416	12.56	59.1	0.00
Nord	751	26.39	470.66	0.00
Nord Ouest	361	13.16	56.26	0.00
Ouest	525	13.29	83.44	0.00
Sud	412	29.49	322.42	0.00
Sud Ouest	378	24.98	212.21	0.00
Yaounde	396	38.01	514.91	0.00
Total	6099	21.4	252.02	0.00

Central African Rep 2000	N	Mean	Mean	Mean
	hgtdps	hgtdps	c2dhreg	pvdhreg
Bamingui-Bangoran	454	71.39	2082.52	0.00
Bangui	1525	83.82	9641.93	0.00
Basse-Kotto	1054	82.8	6503.09	0.00
Haut-Mbomou	352	65.78	1370.61	0.00
Haute-Kotto	757	86.93	5148.09	0.00
Kémo	979	92.22	7494.1	0.00
Lobaye	1055	82.06	6394.01	0.00
Mambéré-Kadeï	733	63.84	2688.25	0.00
Mbomou	787	91.47	5926.46	0.00
Nana-Grébizi	998	85.36	6544.88	0.00
Nana-Mambéré	528	45	962.23	0.00
Ombella-M'poko	1048	87.87	7282.07	0.00
Ouaka	928	78.18	5104.91	0.00
Ouham	604	73.65	2948.44	0.00
Ouham-Pendé	536	70.88	2423.48	0.00
Sangha-Mbaéré	687	88.43	4835.48	0.00

Vakaga	586	78.4	3242.09	0.00
Total	13611	80.44	5533.08	0.00

Central African Rep 2006	N hgtdps	Mean hgtdps	Mean c2dhreg	Mean pvdhreg
“Baminigui Bangoran”	508	42.36	820.43	0.00
“Bangui”	641	55.63	1785.1	0.00
“Basse Kotto”	445	34.39	473.76	0.00
“Haut Mbomou”	320	29.28	246.88	0.00
“Haute Kotto”	1046	38.05	1363.16	0.00
“Lobaye”	654	83.92	4144.96	0.00
“Mambere Kadei”	510	57.25	1504.31	0.00
“Mbomou”	534	76.59	2818.96	0.00
“Nana Mambere”	1210	63.67	4414.1	0.00
“Ombella Mpoko”	578	53.03	1462.73	0.00
“Ouaka”	651	50.75	1508.95	0.00
“Ouham Pende”	592	62.8	2101.01	0.00
“Ouham”	668	55.71	1866.04	0.00
“Sangha Mbaere”	899	80.57	5252.94	0.00
Total	9256	57.71	2446.15	0.00

Central African Rep 2010	N hgtdps	Mean hgtdps	Mean c2dhreg	Mean pvdhreg
Baminigui Bangoran	377	10.12	34.75	0.00
Bangui	636	26.3	395.82	0.00
Basse Kotto	658	4.63	12.67	0.18
Haut Mbomou	343	7.27	16.33	0.06
Haute-Kotto	633	10.67	64.87	0.00
Kémo	829	7.8	45.37	0.00
Lobaye	828	5.97	26.54	0.00
Mambere Kadei	706	4.12	10.77	0.29
Mbomou	566	4.88	12.13	0.21
Nana Grebizi	652	8.13	38.83	0.00
Nana Mambéré	591	6.17	20.25	0.02
Ombella Mpoko	766	7.28	36.51	0.00
Ouaka	549	5.2	13.39	0.15
Ouham	653	11.3	75.1	0.00
Ouham Pende	669	8.79	46.53	0.00
Sangha Mbaere	779	7.1	35.34	0.00
Vakaga	48	18.32	14.5	0.11
Total	10283	8.46	55.91	0.05

Chad 2000	N hgtdps	Mean hgtdps	Mean c2dhreg	Mean pvdhreg
Autres villes	1954	12.96	295.55	0.00
N'Djaména	829	13.76	141.19	0.00
Rural	2483	12.72	361.31	0.00
Total	5266	12.97	302.26	0.00

Chad 2010	N hgtdps	Mean hgtdps	Mean c2dhreg	Mean pvdhreg
Barh El Gazal	657	22.11	288.98	0.00
Bhata	820	10.65	83.68	0.00
Chari Baguirmi	708	22.83	332.14	0.00
Guéra	1280	23.16	618.14	0.00

Hadjer Lamis	719	18.41	219.26	0.00
Kanem	605	18.13	179.02	0.00
Lac	625	16.96	161.77	0.00
Logone Occidental	629	28.88	472.19	0.00
Logone Oriental	870	12.59	124.14	0.00
Mandoul	801	11.81	100.56	0.00
Mayo Kebbi Est	1075	8.37	67.74	0.00
Mayo Kebbi Ouest	803	39.93	1152.38	0.00
Moyen Chari	673	23.88	345.47	0.00
Ndjaména	806	26.07	492.93	0.00
Ouaddai	804	25.09	455.45	0.00
Salamat	741	55.97	2088.92	0.00
Sila	775	14.12	139	0.00
Tandjilé	894	43.75	1539.94	0.00
Wad Fira	632	10.14	58.44	0.00
bet	444	10.85	47.04	0.00
Total	15361	22.36	470.14	0.00

Congo DR 2001	N hgtdps	Mean hgtdps	Mean c2dhreg	Mean pvdhreg
Bandundu	1172	37.93	1517.51	0.00
Bas-congo	532	42.47	863.75	0.00
Equateur	1148	37.25	1433.5	0.00
Kasai Occidental	949	39	1298.87	0.00
Kasai Oriental	1062	54.25	2813.18	0.00
Katanga	1132	35.05	1251.87	0.00
Kinshasa	1415	30.39	1175.78	0.00
Maniema	257	45.43	477.36	0.00
Nord-Kivu	712	17.13	188.03	0.00
Orientale	1005	46.39	1946.19	0.00
Sud-Kivu	591	48.23	1237.44	0.00
Total	9975	38.71	1416.4	0.00

Congo DR 2010	N hgtdps	Mean hgtdps	Mean c2dhreg	Mean pvdhreg
Bandundu	949	10.51	94.34	0.00
Bas congo	753	25.4	437.27	0.00
Equateur	1002	29.24	771.21	0.00
Kasai Occidental	1066	14.44	200.02	0.00
Kasai Oriental	1005	14.43	188.44	0.00
Katanga	1235	21.33	505.46	0.00
Kinshasa	802	20.29	297.28	0.00
Maniema	962	14.06	171.04	0.00
Nord Kivu	1093	17.95	316.78	0.00
Province Orientale	845	14.16	152.57	0.00
Sud Kivu	1085	18.26	325.55	0.00
Total	10797	18.14	319.51	0.00

Côte d'Ivoire 2006	N hgtdps	Mean hgtdps	Mean c2dhreg	Mean pvdhreg
Centre	660	69.98	2908.94	0.00
Centre Est	544	62.88	1935.93	0.00
Centre Nord	756	10.7	77.94	0.00
Centre Ouest	796	39.23	1102.44	0.00
Nord	651	18.33	196.76	0.00

Nord Est	553	70.89	2501.2	0.00
Nord Ouest	913	27.53	622.54	0.00
Ouest	703	26.56	446.17	0.00
Sud (sans ville d' Abidjan)	880	63.63	3206.73	0.00
Sud Ouest	1125	49.45	2475.51	0.00
Ville Abidjan	977	57.43	2899.88	0.00
Total	8558	44.68	1722.7	0.00

Equatorial Guinea 2000	N	Mean	Mean	Mean
	hgtdps	hgtdps	c2dhreg	pvdhreg
Annobon	14	17.82	4	0.68
Bioko Norte	558	18.66	174.94	0.00
Bioko Sur	34	24.88	18.94	0.02
Centro Sur	262	31.19	229.37	0.00
Kie Ntem	492	33	482.07	0.00
Litoral	680	22.16	300.56	0.00
Wele Nzas	299	17.17	79.36	0.00
Total	2339	23.99	266.65	0.00

Gambia 2000	N	Mean	Mean	Mean
	hgtdps	hgtdps	c2dhreg	pvdhreg
Banjul	389	33.8	399.92	0.00
Basse	404	10.57	40.65	0.00
Brikama	364	22.94	172.37	0.00
Janjabureh	333	31.24	292.5	0.00
Kanifing	371	32.81	359.49	0.00
Kerewan	427	19.25	142.39	0.00
Kuntaur	454	12.54	64.28	0.00
Mansakonko	325	44.67	583.71	0.00
Total	3067	25.03	242.97	0.00

Gambia 2005	N	Mean	Mean	Mean
	hgtdps	hgtdps	c2dhreg	pvdhreg
Banjul	160	14.37	29.75	0.00
Basse	1053	4.87	22.49	0.01
Brikama	1365	25.7	811.58	0.00
Janjanburay	752	6.02	24.54	0.00
Kanifing	1413	15.89	321.09	0.00
Kerewan	853	59.17	2687.57	0.00
Kuntaur	395	8.46	25.43	0.00
Mansakonko	397	11.27	45.34	0.00
Total	6388	20	615.05	0.00

Ghana 2006	N	Mean	Mean	Mean
	hgtdps	hgtdps	c2dhreg	pvdhreg
Ashanti	409	23.87	209.66	0.00
Brong Ahafo	241	13.81	41.37	0.00
Central	258	8.95	18.59	0.03
Eastern	333	10.87	35.44	0.00
Greater Accra	304	7.36	14.82	0.10
Northern	573	10.82	60.39	0.00
Upper East	374	9.54	30.65	0.00
Upper West	365	5.51	9.99	0.35
Volta	224	15.12	46.09	0.00
Western	314	7.34	15.24	0.08

Total	3395	11.41	53.5	0.06

Ghana 2011	N	Mean	Mean	Mean
	hgtdps	hgtdps	c2dhreg	pvdhreg
Asante	461	17	119.98	0.00
Brong Ahafo	402	14.94	80.74	0.00
Central	987	13.85	170.4	0.00
Eastern	338	16.52	83.07	0.00
Greater Accra	387	29.26	298.25	0.00
Northern	1978	21.32	808.96	0.00
Upper East	984	10.11	90.51	0.00
Upper West	1120	19.09	367.32	0.00
Volta	399	64.58	1497.47	0.00
Western	396	8.52	25.87	0.00
Total	7452	19.73	417.04	0.00

Guinea Bissau 2000	N	Mean	Mean	Mean
	hgtdps	hgtdps	c2dhreg	pvdhreg
Bafatá	926	57.49	2754.05	0.00
Biombo	299	57.4	886.59	0.00
Bolama/Bijagós	164	65.61	635.39	0.00
Cacheu	769	58.07	2333.61	0.00
Gabú	739	88.85	5250.17	0.00
Oio	1122	82.1	6806.32	0.00
Quinará	239	55.3	657.7	0.00
Tombali	315	41.33	484.33	0.00
sab	1095	28.87	821.5	0.00
Total	5668	60.24	3076.92	0.00

Guinea Bissau 2006	N	Mean	Mean	Mean
	hgtdps	hgtdps	c2dhreg	pvdhreg
EST (Bafata e Gabu)	1316	77.71	7151.51	0.00
NORD (Biombo, Cacheu e Oio)	2352	63.54	8546.18	0.00
SAB Capital	821	74.87	4141.55	0.00
SUD (Bolama, Quinara e Tombali)	1050	72.9	5022.06	0.00
Total	5539	70.36	6893.91	0.00

Mauritania 2007	N	Mean	Mean	Mean
	hgtdps	hgtdps	c2dhreg	pvdhreg
Adrar	306	35.98	356.55	0.00
Assaba	856	32.8	829	0.00
Brakna	812	63.21	2919.53	0.00
Gorgol	850	59.16	2677.36	0.00
Guidimagha	797	17.47	219	0.00
Hodh ECharghi	705	48.03	1463.64	0.00
Hodh ELGharbi	685	17.73	193.76	0.00
Inchiri	124	36.92	152.1	0.00
Nouadhibou	573	44.38	1015.71	0.00
Nouakchott	1331	36.27	1575.89	0.00
Tagant	290	44.08	507.17	0.00
Tiris Zemmour	295	76.88	1569.1	0.00
Trarza	587	18.81	186.85	0.00
Total	8211	39.63	1244.95	0.00

Niger 2000	N	Mean	Mean	Mean

	hgtdps	hgtdps	c2dhreg	pvdhreg
Diffa	65	15.43	13.92	0.13
Agadez	411	10.93	44.16	0.00
Dosso	594	7.51	30.18	0.00
Maradi	1192	11.3	136.93	0.00
Niamey	706	9.15	53.18	0.00
Tahoua	690	7.07	31.01	0.00
Tillaberi	626	8.27	38.5	0.00
Zinder	637	6.46	23.89	0.00
Total	4921	8.95	60.65	0.00

Nigeria 2007	N	Mean	Mean	Mean
	hgtdps	hgtdps	c2dhreg	pvdhreg
Abia	332	41.67	518.72	0.00
Abuja FCT	416	37.92	538.33	0.00
Adamawa	386	64.28	1435.5	0.00
Akwa-Ibom	463	53.07	1173.83	0.00
Anambra	308	56.21	875.77	0.00
Bauchi	723	53.96	1894.33	0.00
Bayelsa	482	56.9	1404.43	0.00
Benue	487	61.88	1678.15	0.00
Borno	446	45.61	834.94	0.00
Cross-Rivers	346	29.35	268.28	0.00
Delta	326	69.69	1424.82	0.00
Ebonyi	427	22.18	189	0.00
Edo	370	68.34	1555.35	0.00
Ekiti	269	48.23	563.23	0.00
Enugu	323	32.51	307.25	0.00
Gombe	397	51.63	952.55	0.00
Imo	259	66.4	1027.6	0.00
Jigawa	811	55.92	2282.4	0.00
Kaduna	671	67.37	2740.94	0.00
Kano	586	47.36	1183.18	0.00
Katsina	538	48.61	1144.01	0.00
Kebbi	498	69.1	2140.15	0.00
Kogi	311	24.42	166.91	0.00
Kwara	320	28.29	230.44	0.00
Lagos	319	78.22	1756.71	0.00
Nasarawa	458	17.79	130.43	0.00
Niger	579	44.36	1025.51	0.00
Ogun	282	38.96	385.23	0.00
Ondo	323	15.17	66.88	0.00
Osun	228	16.54	56.12	0.00
Oyo	337	11.8	42.26	0.00
Plataeu	442	45.95	840.04	0.00
Rivers	285	47.74	584.65	0.00
Sokoto	439	76.57	2316.37	0.00
Taraba	489	50.64	1128.48	0.00
Yobe	688	50.65	1588.63	0.00
Zamfara	818	71.07	3718.97	0.00
Total	16182	49.76	1292.27	0.00

Nigeria 2011	N	Mean	Mean	Mean
	hgtdps	hgtdps	c2dhreg	pvdhreg
Abia	458	12.3	62.35	0.00

Adamawa	782	5.48	21.15	0.01
Akwa ibom	545	26.05	332.96	0.00
Anambra	544	11.51	64.86	0.00
Bauchi	931	18.9	299.26	0.00
Bayelsa	517	7.4	25.5	0.00
Benue	604	52.52	1499.24	0.00
Borno	671	5.31	17.03	0.05
Cross River	553	8.46	35.63	0.00
Delta	526	9.84	45.86	0.00
Ebonyi	647	9.27	50.08	0.00
Edo	506	18.4	154.2	0.00
Ekiti	374	12.67	54.02	0.00
Enugu	343	32.98	335.69	0.00
FCT (Abuja)	599	5.93	18.95	0.03
Gombe	898	8.13	53.47	0.00
Imo	406	15.51	87.89	0.00
Jigawa	1002	12.2	134.23	0.00
Kaduna	924	6.34	33.4	0.00
Kano	898	10.88	95.74	0.00
Katsina	975	11.75	121.11	0.00
Kebbi	865	11.4	101.09	0.00
Kogi	422	36.02	492.64	0.00
Kwara	544	6.14	18.46	0.03
Lagos	510	8.59	33.88	0.00
Nasarawa	743	9.85	64.87	0.00
Niger	900	34.43	960.44	0.00
Ogun	533	13.47	87.09	0.00
Ondo	402	40.4	590.59	0.00
Osun	442	15.21	92.03	0.00
Oyo	539	21.15	216.97	0.00
Plateau	633	12.41	87.71	0.00
Rivers	446	12.71	64.85	0.00
Sokoto	1000	12.34	137.08	0.00
Taraba	659	8.64	44.29	0.00
Yobe	933	13.56	154.34	0.00
Zamfara	968	15.5	209.27	0.00
Total	24242	14.71	185.57	0.00

**Sao Tome et Principe 2000**	**N**	**Mean**	**Mean**	**Mean**
	**hgtdps**	**hgtdps**	**c2dhreg**	**pvdhreg**
Centro	1380	84.27	8820.56	0.00
Norte	379	79.32	2145.94	0.00
Principe	93	55.25	255.52	0.00
Sul	97	46.85	191.62	0.00
Total	1949	80.06	6684.47	0.00

**Senegal 2000**	**N**	**Mean**	**Mean**	**Mean**
	**hgtdps**	**hgtdps**	**c2dhreg**	**pvdhreg**
Dakar	982	21.69	415.94	0.00
Diourbel	734	18.23	219.62	0.00
Fatick	869	23.95	448.59	0.00
Kaolack	938	22.62	431.98	0.00
Kolda	637	37.58	809.48	0.00
Louga	878	6.02	28.63	0.00
Saint louis	849	5.82	25.92	0.00

Tambacounda	844	18.17	250.88	0.00
Thies	1140	15.72	253.42	0.00
Ziguinchor	722	33.14	713.68	0.00
Total	8593	19.56	342.53	0.00

**Sierra Leone 2000**	**N**	**Mean**	**Mean**	**Mean**
	**hgtdps**	**hgtdps**	**c2dhreg**	**pvdhreg**
East	632	24.68	346.32	0.00
North	887	19.1	291.25	0.00
South	347	18.49	106.75	0.00
West	592	33.54	599.42	0.00
Total	2458	23.93	353.59	0.00

**Sierra Leone 2005**	**N**	**Mean**	**Mean**	**Mean**
	**hgtdps**	**hgtdps**	**c2dhreg**	**pvdhreg**
East	1125	46.71	2209.37	0.00
North	2078	26.64	1327.24	0.00
South	1473	13.17	230.09	0.00
West	507	22.46	230.14	0.00
Total	5183	26.76	1099.58	0.00

**Sierra Leone 2010**	**N**	**Mean**	**Mean**	**Mean**
	**hgtdps**	**hgtdps**	**c2dhreg**	**pvdhreg**
East	1813	28.28	1305.37	0.00
North	3200	18.78	1015.55	0.00
South	2278	26.49	1438.59	0.00
West	1044	28.72	775.02	0.00
Total	8335	24.2	1164.08	0.00

**Togo 2006**	**N**	**Mean**	**Mean**	**Mean**
	**hgtdps**	**hgtdps**	**c2dhreg**	**pvdhreg**
Centrale	594	28.93	447.31	0.00
Kara	581	53.93	1520.74	0.00
Lomé commune	511	18.66	160.14	0.00
Maritime (sans Lomé commune)	806	36.83	984.15	0.00
Plateaux	527	38.62	707.48	0.00
Savanes	968	30.54	812.79	0.00
Total	3987	34.53	798.58	0.00

**Togo 2010**	**N**	**Mean**	**Mean**	**Mean**
	**hgtdps**	**hgtdps**	**c2dhreg**	**pvdhreg**
Centrale	743	4.28	12.28	0.20
Kara	823	5.44	21.92	0.01
Lomé	466	8.87	32.97	0.00
Maritime	770	4.35	13.09	0.16
Plateaux	792	8.2	47.87	0.00
Savanes	1097	3.77	14.04	0.12
Total	4691	5.49	22.58	0.09

Appendix D

Digit preference score for terminal digit of weight, by region DHS

Benin 2001	N	Mean	Mean	Mean
	wgtdps	wgtdps	c2dwreg	pvdwreg
atacora	680	5.01	15.38	0.08
atlantique	954	3.59	11.07	0.27
borgou	786	6.08	26.16	0.00
mono	614	4.83	12.87	0.17
oueme	689	3.02	5.64	0.77
zou	800	2.11	3.20	0.96
Total	4523	4.06	12.37	0.38

Benin 2006	N	Mean	Mean	Mean
	wgtdps	wgtdps	c2dwreg	pvdwreg
alibori	1143	12.65	164.67	0.00
atacora	1291	3.30	12.68	0.18
atlantique	1555	5.75	46.34	0.00
borgou	1407	22.74	654.77	0.00
collines	949	5.51	25.91	0.00
couffo	953	15.11	195.89	0.00
donga	708	8.78	49.15	0.00
littoral	890	2.23	4.00	0.91
mono	834	11.82	104.92	0.00
plateau	676	6.88	28.76	0.00
quémé	1580	5.18	38.22	0.00
zou	1464	11.34	169.36	0.00
Total	13450	9.43	138.50	0.08

Benin 2011	N	Mean	Mean	Mean
	wgtdps	wgtdps	c2dwreg	pvdwreg
Alibori	824	48.94	1776.49	0.00
Atacora	1173	22.68	543.26	0.00
Atlantique	1381	28.67	1021.57	0.00
Borgou	951	16.58	235.40	0.00
Collines	791	13.69	133.36	0.00
Couffo	918	7.46	45.94	0.00
Donga	657	29.98	531.48	0.00
Littoral	891	35.37	1003.29	0.00
Mono	707	16.60	175.31	0.00
Ou?m?	1389	13.22	218.47	0.00
Plateau	765	25.73	455.69	0.00
Zou	1180	22.69	546.93	0.00
Total	11627	23.03	563.16	0.00

Burkina Faso 1993	N	Mean	Mean	Mean
	wgtdps	wgtdps	c2dwreg	pvdwreg
central/south	1083	3.92	14.98	0.09
east	847	5.85	26.07	0.00
north	743	6.44	27.73	0.00
ouagadougou	870	6.03	28.46	0.00
west	1188	3.11	10.37	0.32

Total	4731	4.85	20.29	0.10

Burkina Faso 1998	N	Mean	Mean	Mean
	wgtdps	wgtdps	c2dwreg	pvdwreg
central/south	1216	4.11	18.49	0.03
east	1348	2.26	6.18	0.72
north	649	3.44	6.92	0.65
ouagadougou	458	4.69	9.07	0.43
west	1143	2.48	6.34	0.71
Total	4814	3.17	9.70	0.51

Burkina Faso 2003	N	Mean	Mean	Mean
	wgtdps	wgtdps	c2dwreg	pvdwreg
boucle de mouhoun	688	34.03	717.23	0.00
cascades	587	79.61	3348.55	0.00
centre (sans ouagadougou)	271	5.73	8.00	0.53
centre-est	614	44.64	1101.34	0.00
centre-nord	743	10.59	74.97	0.00
centre-ouest	818	2.48	4.52	0.87
centre-sud	591	6.47	22.25	0.01
est	662	53.80	1724.53	0.00
hauts bassins	725	4.11	11.01	0.27
nord	671	3.52	7.46	0.59
ouagadougou	249	4.08	3.73	0.93
plateau central	832	40.36	1220.04	0.00
sahel	575	6.33	20.70	0.01
sud-ouest	820	3.50	9.02	0.44
Total	8846	22.03	610.44	0.23

Burkina Faso 2010	N	Mean	Mean	Mean
	wgtdps	wgtdps	c2dwreg	pvdwreg
boucle de mouhoun	625	2.58	3.75	0.93
cascades	414	7.58	21.41	0.01
centre	391	7.92	22.07	0.01
centre-est	538	5.62	15.27	0.08
centre-nord	540	10.81	56.81	0.00
centre-ouest	595	1.63	1.42	1.00
centre-sud	427	7.70	22.81	0.01
est	640	4.72	12.81	0.17
hauts basins	580	4.79	11.97	0.22
nord	557	4.50	10.16	0.34
plateau central	464	5.99	14.97	0.09
sahel	514	6.50	19.54	0.02
sud-ouest	460	3.69	5.65	0.77
Total	6745	5.50	16.27	0.31

Cameroon 1991	N	Mean	Mean	Mean
	wgtdps	wgtdps	c2dwreg	pvdwreg
adam/nord/ext-nord	834	3.97	11.85	0.22
centre/sud/est	433	6.49	16.40	0.06
nord-ouest/sud-ouest	364	6.69	14.68	0.10
ouest/littoral	402	7.08	18.15	0.03
yaoundé/douala	652	3.86	8.74	0.46
Total	2685	5.19	13.15	0.21

Cameroon 1998	N	Mean	Mean	Mean
	wgtdps	wgtdps	c2dwreg	pvdwreg
central, south, & east	563	7.45	28.10	0.00
north/ extreme north/ ad	670	3.11	5.82	0.76
northwest & southwest	322	4.74	6.51	0.69
west & littoral	383	6.57	14.89	0.09
Total	1938	5.32	14.20	0.40

Cameroon 2011	N	Mean	Mean	Mean
	wgtdps	wgtdps	c2dwreg	pvdwreg
adamaoua	474	2.27	2.20	0.99
centre	405	4.59	7.67	0.57
douala	363	4.67	7.11	0.63
est	383	5.76	11.44	0.25
extrême-nord	755	2.24	3.40	0.95
littoral	272	6.10	9.10	0.43
nord	679	5.02	15.42	0.08
nord-ouest	500	5.18	12.08	0.21
ouest	467	5.14	11.09	0.27
sud	282	8.25	17.29	0.04
sud-ouest	311	6.01	10.13	0.34
yaoundé	308	7.57	15.90	0.07
Total	5199	4.83	9.76	0.44

Central African Rep 1994	N	Mean	Mean	Mean
	wgtdps	wgtdps	c2dwreg	pvdwreg
bangui	495	5.92	15.61	0.08
rs i	398	8.31	24.71	0.00
rs ii	423	4.68	8.32	0.50
rs iii	477	6.26	16.82	0.05
rs iv	343	3.61	4.03	0.91
rs v	323	4.69	6.38	0.70
Total	2459	5.67	13.24	0.33

Chad 1996	N	Mean	Mean	Mean
	wgtdps	wgtdps	c2dwreg	pvdwreg
b.e.t.	53	14.90	10.58	0.31
batha	244	7.00	10.75	0.29
biltine	136	11.14	15.18	0.09
chari-baguirmi	633	2.27	2.94	0.97
guéra	223	6.38	8.17	0.52
kanem	218	5.33	5.58	0.78
lac	217	8.24	13.28	0.15
logone occidental	406	3.43	4.30	0.89
logone oriental	387	6.18	13.28	0.15
mayo-kebbi	638	4.26	10.40	0.32
moyen chari	653	4.30	10.84	0.29
n'djaména	988	2.88	7.38	0.60
ouaddaï	524	4.67	10.27	0.33
salamat	216	7.91	12.15	0.21
tandjilé	425	4.63	8.20	0.51
Total	5961	4.71	8.79	0.48

Chad 2004	N	Mean	Mean	Mean
	wgtdps	wgtdps	c2dwreg	pvdwreg

b. e. t.	488	6.98	21.39	0.01
bar azoum	433	4.10	6.56	0.68
centre est	406	6.31	14.54	0.10
chari baguirmi	465	3.89	6.33	0.71
logone occidental	575	4.21	9.16	0.42
mayo kebbi	529	5.21	12.91	0.17
moyen chari	456	4.24	7.38	0.60
n'djaména	943	3.69	11.54	0.24
ouaddai est	396	8.44	25.41	0.00
Total	4691	5.01	12.48	0.32

**Congo 2005**	**N**	**Mean**	**Mean**	**Mean**
	**wgtdps**	**wgtdps**	**c2dwreg**	**pvdwreg**
brazzaville	984	4.44	17.42	0.04
nord	968	3.52	10.82	0.29
pointe noire	815	3.68	9.92	0.36
sud	1324	3.82	17.34	0.04
Total	4091	3.87	14.34	0.16

**Congo 2012**	**N**	**Mean**	**Mean**	**Mean**
	**wgtdps**	**wgtdps**	**c2dwreg**	**pvdwreg**
bouenza	436	5.47	11.75	0.23
brazzaville	309	3.62	3.65	0.93
cuvette	337	3.07	2.85	0.97
cuvette - ouest	272	6.02	8.88	0.45
kouilou	455	5.39	11.88	0.22
lekoumou	329	6.62	12.98	0.16
likouala	445	5.09	10.39	0.32
niari	415	5.26	10.33	0.32
plateaux	342	4.33	5.78	0.76
pointe-noire	433	6.22	15.06	0.09
pool	477	5.69	13.92	0.13
sangha	287	6.83	12.06	0.21
Total	4537	5.31	10.32	0.37

**Congo DR 2007**	**N**	**Mean**	**Mean**	**Mean**
	**wgtdps**	**wgtdps**	**c2dwreg**	**pvdwreg**
bandundu	366	5.39	9.57	0.39
bas-congo	254	4.92	5.53	0.79
equateur	340	8.85	23.94	0.00
kasaï occident	337	3.91	4.63	0.87
kasaï oriental	366	5.38	9.52	0.39
katanga	360	5.71	10.56	0.31
kinshasa	375	4.66	7.32	0.60
maniema	356	7.69	18.94	0.03
nord-kivu	345	8.44	22.10	0.01
orientale	277	7.87	15.45	0.08
sud-kivu	330	7.95	18.79	0.03
Total	3706	6.41	13.35	0.31

**Côte d'Ivoire 1994**	**N**	**Mean**	**Mean**	**Mean**
	**wgtdps**	**wgtdps**	**c2dwreg**	**pvdwreg**
center	325	5.79	9.80	0.37
center east	145	9.21	11.07	0.27
center north	317	5.09	7.38	0.60

center west	574	5.05	13.18	0.15
north	250	5.99	8.08	0.53
north east	187	5.53	5.14	0.82
north west	216	6.80	9.00	0.44
south	916	2.26	4.20	0.90
south west	246	5.18	5.95	0.74
west	344	5.78	10.36	0.32
Total	3520	4.85	8.09	0.55

**Côte d'Ivoire 1998**	**N**	**Mean**	**Mean**	**Mean**
	**wgtdps**	**wgtdps**	**c2dwreg**	**pvdwreg**
capital (abidjan)	435	4.45	7.74	0.56
countryside	660	4.23	10.61	0.30
small city	511	7.85	28.32	0.00
Total	1606	5.44	15.46	0.28

**Côte d'Ivoire 2011**	**N**	**Mean**	**Mean**	**Mean**
	**wgtdps**	**wgtdps**	**c2dwreg**	**pvdwreg**
Centre	276	8.88	19.58	0.02
Centre-Est	259	4.40	4.51	0.87
Centre-Nord	360	3.97	5.11	0.82
Centre-Ouest	306	5.97	9.82	0.37
Nord	327	6.84	13.76	0.13
Nord-Ouest	435	3.53	4.89	0.84
Nord-est	274	6.76	11.26	0.26
Ouest	359	5.86	11.11	0.27
Sud sans Abidjan	196	4.58	3.69	0.93
Sud-ouest	269	9.13	20.18	0.02
Ville d'Abidjan	250	6.14	8.48	0.49
Total	3311	5.88	10.08	0.46

**Gabon 2000**	**N**	**Mean**	**Mean**	**Mean**
	**wgtdps**	**wgtdps**	**c2dwreg**	**pvdwreg**
east (haut-ogooué & ogooué-lolo)	861	3.17	7.77	0.56
libreville,port-gentil	831	1.90	2.71	0.97
north (ogooué-ivindo & woleu-ntem)	625	3.91	8.62	0.47
south (ngounié, nyanga)	681	4.30	11.32	0.25
west (estuaire, moyen-ogooué & ogooué-maritime)	574	3.28	5.55	0.78
Total	3572	3.24	7.06	0.62

**Gabon 2012**	**N**	**Mean**	**Mean**	**Mean**
	**wgtdps**	**wgtdps**	**c2dwreg**	**pvdwreg**
estuaire	289	7.43	14.36	0.11
haut-ogooué	411	6.54	15.84	0.07
libreville-port-gentil	477	5.49	12.92	0.17
moyen-ogooué	269	6.59	10.52	0.31
ngounié	417	4.03	6.09	0.73
nyanga	308	4.40	5.38	0.80
ogooué maritime	245	6.62	9.65	0.38
ogooué-ivindo	538	3.22	5.01	0.83
ogooué-lolo	314	7.39	15.43	0.08
woleu-ntem	241	6.56	9.33	0.41
Total	3509	5.56	10.26	0.41

**Ghana 1993**	**N**	**Mean**	**Mean**	**Mean**

	wgtdps	wgtdps	c2dwreg	pvdwreg
ashanti	360	4.76	7.33	0.60
brong-ahafo	194	8.21	11.77	0.23
central	209	8.18	12.58	0.18
eastern	218	10.50	21.63	0.01
greater accra	182	8.02	10.53	0.31
northern	217	5.14	5.17	0.82
upper east	140	9.34	11.00	0.28
upper west	71	13.52	11.68	0.23
volta	223	4.82	4.67	0.86
western	176	7.59	9.11	0.43
Total	1990	7.32	10.21	0.43

Ghana 1998	N	Mean	Mean	Mean
	wgtdps	wgtdps	c2dwreg	pvdwreg
ashanti region	401	4.50	7.30	0.61
brong ahafo region	196	8.27	12.06	0.21
central region	255	9.10	19.00	0.03
eastern region	330	5.50	8.97	0.44
greater accra region	253	7.00	11.15	0.27
northern region	290	5.63	8.28	0.51
upper east region	340	6.41	12.59	0.18
upper west region	241	8.45	15.47	0.08
volta region	255	7.12	11.63	0.24
western region	312	5.61	8.83	0.45
Total	2873	6.52	11.16	0.32

Ghana 2003	N	Mean	Mean	Mean
	wgtdps	wgtdps	c2dwreg	pvdwreg
ashanti	486	7.24	22.93	0.01
brong ahafo	390	8.12	23.13	0.01
central	213	10.78	22.26	0.01
eastern	278	4.35	4.73	0.86
greater accra	289	5.71	8.47	0.49
northern	526	5.33	13.43	0.14
upper east	258	12.54	36.50	0.00
upper west	301	11.17	33.78	0.00
volta	238	10.55	23.85	0.00
western	298	8.28	18.38	0.03
Total	3277	8.00	20.28	0.14

Ghana 2008	N	Mean	Mean	Mean
	wgtdps	wgtdps	c2dwreg	pvdwreg
ashanti	389	5.34	10.00	0.35
brong ahafo	242	6.74	9.90	0.36
central	182	5.90	5.69	0.77
eastern	221	7.25	10.45	0.32
greater accra	237	6.83	9.96	0.35
northern	402	5.31	10.19	0.34
upper east	191	10.01	17.22	0.05
upper west	238	6.16	8.13	0.52
volta	221	4.39	3.84	0.92
western	240	2.71	1.58	1.00
Total	2563	5.91	8.79	0.48

Guinea 1999	N wgtdps	Mean wgtdps	Mean c2dwreg	Mean pvdwreg
central guinea	796	3.66	9.58	0.39
conakry	668	5.35	17.21	0.05
forest guinea	1273	2.40	6.60	0.68
lower guinea	981	1.92	3.27	0.95
upper guinea	977	4.84	20.62	0.01
Total	4695	3.44	10.84	0.46

Guinea 2005	N wgtdps	Mean wgtdps	Mean c2dwreg	Mean pvdwreg
boké	338	5.64	9.69	0.38
conakry	210	7.78	11.43	0.25
faranah	383	3.50	4.23	0.90
kankan	491	6.54	18.92	0.03
kindia	398	3.72	4.96	0.84
labé	261	6.32	9.38	0.40
mamou	274	4.99	6.15	0.73
n'zérékoré	407	6.87	17.30	0.04
Total	2762	5.57	10.77	0.43

Guinea 2012	N wgtdps	Mean wgtdps	Mean c2dwreg	Mean pvdwreg
Bok?	339	4.30	5.63	0.78
Conakry	306	7.23	14.39	0.11
Faranah	471	2.40	2.44	0.98
Kankan	578	6.74	23.66	0.00
Kindia	391	5.21	9.54	0.39
Lab?	369	5.35	9.51	0.39
Mamou	347	5.81	10.55	0.31
N'Z?r?kor?	419	4.55	7.80	0.55
Total	3220	5.16	10.96	0.43

Liberia 2007	N wgtdps	Mean wgtdps	Mean c2dwreg	Mean pvdwreg
monrovia	758	5.50	20.63	0.01
north central	957	4.70	19.06	0.02
north western	596	4.36	10.21	0.33
south central	739	2.69	4.80	0.85
south eastern a	671	4.66	13.13	0.16
south eastern b	883	3.68	10.74	0.29
Total	4604	4.26	13.42	0.27

Mali 1995	N wgtdps	Mean wgtdps	Mean c2dwreg	Mean pvdwreg
bamako	505	4.27	8.29	0.51
gao	260	8.29	16.08	0.07
kayes	727	5.16	17.42	0.04
koulikoro	929	5.64	26.62	0.00
mopti	563	9.20	42.88	0.00
sikasso	947	4.81	19.73	0.02
ségou	845	6.84	35.53	0.00
timbuktu	255	6.80	10.61	0.30
Total	5031	6.07	24.11	0.08

Mali 2001	N wgtdps	Mean wgtdps	Mean c2dwreg	Mean pvdwreg
bamako	1061	3.39	10.94	0.28
gao	398	4.71	7.93	0.54
kayes	1511	4.92	32.94	0.00
kidal	144	10.97	15.58	0.08
koulikoro	1673	1.93	5.62	0.78
mopti	1302	2.18	5.59	0.78
segou	1444	5.11	33.98	0.00
sikasso	2009	1.77	5.64	0.78
tombouctou	369	5.34	9.46	0.40
Total	9911	3.38	14.87	0.46

Mali 2006	N wgtdps	Mean wgtdps	Mean c2dwreg	Mean pvdwreg
bamako	1192	5.31	30.27	0.00
gao	801	5.71	23.51	0.01
kayes	1477	4.14	22.82	0.01
kidal	268	8.11	15.88	0.07
koulikoro	1565	5.00	35.19	0.00
mopti	1782	6.26	62.77	0.00
segou	1387	3.86	18.65	0.03
sikasso	2347	2.56	13.89	0.13
tombouctou	820	11.00	89.27	0.00
Total	11639	5.03	33.63	0.03

Niger 1992	N wgtdps	Mean wgtdps	Mean c2dwreg	Mean pvdwreg
agadez	202	8.31	12.55	0.18
diffa	124	7.13	5.68	0.77
dosso	574	4.01	8.30	0.50
maradi	885	6.34	31.98	0.00
niamey	905	4.41	15.85	0.07
tahoua	836	3.37	8.52	0.48
tillabéri	691	3.40	7.19	0.62
zinder	817	2.93	6.32	0.71
Total	5034	4.37	13.49	0.38

Niger 1998	N wgtdps	Mean wgtdps	Mean c2dwreg	Mean pvdwreg
dosso	596	4.77	12.22	0.20
maradi	979	2.67	6.30	0.71
niamey	400	6.56	15.50	0.08
tahoua/agadez	693	3.27	6.67	0.67
tillaberi	743	3.75	9.40	0.40
zinda/diffa	665	5.01	15.05	0.09
Total	4076	4.04	10.12	0.41

Niger 2006	N wgtdps	Mean wgtdps	Mean c2dwreg	Mean pvdwreg
agadez	272	5.02	6.16	0.72
diffa	328	6.14	11.15	0.27
dosso	634	3.08	5.40	0.80
maradi	621	5.52	17.02	0.05
niamey	419	4.42	7.37	0.60

tahoua	644	2.80	4.54	0.87
tillabéri	534	4.80	11.06	0.27
zinder	432	5.03	9.85	0.36
Total	3884	4.42	9.14	0.50

**Niger 2012**	**N**	**Mean**	**Mean**	**Mean**
	**wgtdps**	**wgtdps**	**c2dwreg**	**pvdwreg**
Agadez	279	12.33	38.17	0.00
Diffa	431	30.38	357.98	0.00
Dosso	754	3.53	8.47	0.49
Maradi	1119	4.55	20.85	0.01
Niamey	400	9.19	30.40	0.00
Tahoua	836	14.24	152.54	0.00
Tillaberi	689	2.98	5.50	0.79
Zinder	661	11.13	73.72	0.00
Total	5169	9.53	74.84	0.18

**Nigeria 1990**	**N**	**Mean**	**Mean**	**Mean**
	**wgtdps**	**wgtdps**	**c2dwreg**	**pvdwreg**
northeast	1463	10.41	142.67	0.00
northwest	1379	7.89	77.27	0.00
southeast	1661	7.77	90.30	0.00
southwest	1649	5.70	48.18	0.00
Total	6152	7.87	88.54	0.00

**Nigeria 2003**	**N**	**Mean**	**Mean**	**Mean**
	**wgtdps**	**wgtdps**	**c2dwreg**	**pvdwreg**
north central	839	3.49	9.19	0.42
north east	1102	2.02	4.04	0.91
north west	1429	5.42	37.82	0.00
south east	441	5.38	11.49	0.24
south south	441	8.51	28.77	0.00
south west	551	5.47	14.83	0.10
Total	4803	4.59	19.18	0.32

**Nigeria 2008**	**N**	**Mean**	**Mean**	**Mean**
	**wgtdps**	**wgtdps**	**c2dwreg**	**pvdwreg**
north central	4201	6.62	165.76	0.00
north east	5278	6.31	188.95	0.00
north west	6268	9.98	561.71	0.00
south east	1977	4.76	40.36	0.00
south south	2769	3.59	32.20	0.00
south west	2899	4.20	45.92	0.00
Total	23392	6.63	235.83	0.00

**Sao Tome et Principe 2008**	**N**	**Mean**	**Mean**	**Mean**
	**wgtdps**	**wgtdps**	**c2dwreg**	**pvdwreg**
região centro	423	76.72	2240.76	0.00
região do principe	288	71.06	1308.67	0.00
região norte	490	81.40	2921.71	0.00
região sul	456	81.20	2705.80	0.00
Total	1657	78.35	2408.10	0.00

**Senegal 1992**	**N**	**Mean**	**Mean**	**Mean**

	wgtdps	wgtdps	c2dwreg	pvdwreg
central	1789	2.94	13.93	0.12
north east	824	2.51	4.67	0.86
south	558	4.61	10.67	0.30
west	1576	3.22	14.67	0.10
Total	4747	3.15	12.19	0.27

Senegal 2005	N	Mean	Mean	Mean
	wgtdps	wgtdps	c2dwreg	pvdwreg
dakar	190	5.71	5.58	0.78
diourbel	316	6.95	13.75	0.13
fatick	237	4.36	4.05	0.91
kaolack	341	6.20	11.82	0.22
kolda	333	6.33	12.02	0.21
louga	259	4.80	5.36	0.80
matam	229	6.52	8.77	0.46
saint-louis	222	5.32	5.66	0.77
tambacounda	294	4.40	5.12	0.82
thiès	344	5.52	9.43	0.40
zuguinchor	185	7.44	9.22	0.42
Total	2950	5.77	8.64	0.51

Senegal 2010	N	Mean	Mean	Mean
	wgtdps	wgtdps	c2dwreg	pvdwreg
dakar	256	3.60	2.98	0.96
diourbel	312	9.68	26.33	0.00
fatick	322	5.53	8.87	0.45
kaffrine	331	4.21	5.28	0.81
kaolack	399	6.95	17.37	0.04
kedougou	124	9.25	9.55	0.39
kolda	341	4.40	5.95	0.74
louga	303	4.15	4.69	0.86
matam	251	4.62	4.82	0.85
saint-louis	248	7.36	12.08	0.21
sedhiou	286	3.54	3.23	0.95
tambacounda	271	5.99	8.74	0.46
thiès	324	9.44	26.00	0.00
ziguinchor	178	7.19	8.29	0.50
Total	3946	6.02	10.78	0.51

Sierra Leone 2008	N	Mean	Mean	Mean
	wgtdps	wgtdps	c2dwreg	pvdwreg
eastern	583	6.42	21.61	0.01
northern	793	4.20	12.56	0.18
southern	565	7.65	29.74	0.00
western	341	7.01	15.10	0.09
Total	2282	6.04	19.51	0.08

Togo 1998	N	Mean	Mean	Mean
	wgtdps	wgtdps	c2dwreg	pvdwreg
centrale	600	4.09	9.03	0.43
kara	608	3.82	7.99	0.54
lomé	303	5.32	7.73	0.56
marities	509	6.30	18.17	0.03
plateaux	794	2.76	5.45	0.79

savanes	1002	2.47	5.49	0.79
Total	3816	3.74	8.30	0.58

Digit preference score for terminal digit of weight, by region NNS

Benin 2008	N	Mean	Mean	Mean
	wgtdps	wgtdps	c2dwreg	pvdwreg
alibori	369	4.84	7.78	0.56
atacora	348	5.12	8.21	0.51
atlantique	306	6.26	10.8	0.29
borgou	326	6.29	11.61	0.24
collines	247	6.53	9.48	0.39
couffo	393	5.2	9.57	0.39
donga	258	6.68	10.37	0.32
littoral	222	12.41	30.79	0
mono	243	7.34	11.77	0.23
oueme	264	7.1	11.98	0.21
plateau	225	5.23	5.53	0.79
zou	236	6.51	9	0.44
Total	3437	6.41	11	0.37

Burkina Faso 2012	N	Mean	Mean	Mean
	wgtdps	wgtdps	c2dwreg	pvdwreg
bales	774	1.64	1.87	0.99
bam	823	1.37	1.39	1
banwa	615	1.61	1.44	1
banwa ganzourgou	836	2.51	4.74	0.86
bazega	592	1.37	1.01	1
boulgou	683	2.74	4.6	0.87
cascades	640	2.42	3.38	0.95
centre ouest	580	2.56	3.41	0.95
est	921	1.5	1.88	0.99
houet	475	1.23	0.64	1
kadiogo	428	4.13	6.58	0.68
kenedougou	753	2.83	5.42	0.8
kossi	745	1.38	1.27	1
koulpelogo	827	3.38	8.51	0.48
kouritenga	706	3.17	6.38	0.7
kourweogo	654	2.27	3.03	0.96
mouhoun	637	4.49	11.56	0.24
nahouri	497	1.64	1.21	1
namentenga	876	1.88	2.79	0.97
nayala	644	3.82	8.45	0.49
nord	717	2.12	2.9	0.97
oubritenga	655	1.74	1.78	0.99
sahel	835	0.93	0.65	1
sanmentenga	859	1.65	2.11	0.99
sourou	658	3.53	7.38	0.6
sud ouest	497	3.38	5.11	0.82
tuy	563	0.75	0.29	1
zoundweogo	609	0.45	0.11	1
Total	19099	2.2	3.55	0.87

Cameroon 2011	N	Mean	Mean	Mean
	wgtdps	wgtdps	c2dwreg	pvdwreg
extrême-nord	645	3.49	7.08	0.63

nord	672	4.13	10.29	0.33
Total	1317	3.81	8.72	0.48

**Central African Rep 2012**	**N**	**Mean**	**Mean**	**Mean**
	**wgtdps**	**wgtdps**	**c2dwreg**	**pvdwreg**
bamingui bangoran	893	2.67	5.73	0.77
bangui	807	3.58	9.32	0.41
basse kotto	938	2.39	4.84	0.85
haut kotto	974	3.37	9.98	0.35
haut mbomou	947	2.52	5.43	0.8
kemo	928	2.82	6.66	0.67
lobaye	1091	3.62	12.84	0.17
mambere kadei	1295	3.4	13.44	0.14
mbomou	983	2.57	5.86	0.75
nana grebizi	1073	2.18	4.59	0.87
nana mambere	654	5.82	19.91	0.02
ombella mpoko	883	3.08	7.52	0.58
ouaka	817	3.72	10.18	0.34
ouham	997	3.59	11.56	0.24
ouham pende	785	3.76	9.97	0.35
sangha mbarere	1083	3.24	10.21	0.33
vakaga	505	5.09	11.77	0.23
Total	15653	3.27	9.24	0.47

**Chad June 2012**	**N**	**Mean**	**Mean**	**Mean**
	**wgtdps**	**wgtdps**	**c2dwreg**	**pvdwreg**
Barh El Ghazal	719	3.09	6.16	0.72
Batha	691	1.89	2.21	0.99
Guéra	735	1.92	2.43	0.98
Hadjer Lamis	609	2.53	3.5	0.94
Kanem	567	6.06	18.77	0.03
Lac	533	4.06	7.92	0.54
N’Djamena	750	2.39	3.84	0.92
Ouaddai	591	4.13	9.05	0.43
Salamat	734	2.6	4.47	0.88
Sila	686	2.79	4.82	0.85
Wadi Fira	723	3.73	9.05	0.43
Total	7338	3.11	6.28	0.72

**Chad (7 regions) Dec/Jan 2012-13**	**N**	**Mean**	**Mean**	**Mean**
	**wgtdps**	**wgtdps**	**c2dwreg**	**pvdwreg**
Logone Occidental	810	3.3	7.93	0.54
Logone Oriental	809	2.27	3.74	0.93
Mandoul	808	2.07	3.11	0.96
Mayo-Kebbi Est	994	3.54	11.19	0.26
Mayo-Kebbi Ouest	637	3.71	7.88	0.55
Moyen-Chari	711	3.45	7.61	0.57
Tandjilé	902	3.59	10.44	0.32
Total	5671	3.13	7.57	0.58

**The Gambia 2012**	**N**	**Mean**	**Mean**	**Mean**
	**wgtdps**	**wgtdps**	**c2dwreg**	**pvdwreg**
banjul	719	3.48	7.83	0.55
basse	1316	2.16	5.52	0.79
brikama	832	4.65	16.22	0.06

janjanburay	1008	3.5	11.09	0.27
kanifing	748	2.6	4.54	0.87
kerewan	1106	2.09	4.34	0.89
kuntaur	1076	2.84	7.81	0.55
mansakonko	831	4.12	12.72	0.18
Total	7636	3.07	8.48	0.54

**Guinea-Bissau 2008**	**N**	**Mean**	**Mean**	**Mean**
	**wgtdps**	**wgtdps**	**c2dwreg**	**pvdwreg**
Capitale	444	3.74	5.59	0.78
Est (Bafata e Gabu)	836	3.37	8.52	0.48
Nord (Biombo, Cacheu e Oio)	752	4.29	12.47	0.19
Sud (Bolama, Quinara e Tombali)	685	4.63	13.23	0.15
Total	2717	4	10.32	0.37

**Guinée Conakay 2012**	**N**	**Mean**	**Mean**	**Mean**
	**wgtdps**	**wgtdps**	**c2dwreg**	**pvdwreg**
boke nord	807	9.01	58.99	0
boke sud	624	4.29	10.33	0.32
conakry	771	10.3	73.66	0
faranah	654	5.45	17.5	0.04
kankan	772	5.25	19.17	0.02
kindia	804	6.71	32.62	0
labe	1022	3.9	13.97	0.12
mamou	1049	8.33	65.46	0
nzerekore	1007	7	44.45	0
Total	7510	6.75	38.75	0.05

**Liberia 2010**	**N**	**Mean**	**Mean**	**Mean**
	**wgtdps**	**wgtdps**	**c2dwreg**	**pvdwreg**
bomi	334	4.19	5.28	0.81
bong	477	6.85	20.13	0.02
gbarpolu	369	9.95	32.87	0
grand bassa	405	5.62	11.52	0.24
grand cape mount	500	7.79	27.28	0
grand gedeh	443	3.81	5.78	0.76
grand kru	450	6.33	16.22	0.06
lofa	481	2.83	3.47	0.94
margibi	385	14.42	72.01	0
maryland	373	6.85	15.77	0.07
montserrado	350	4.97	7.77	0.56
nimba	441	5.31	11.18	0.26
river gee	419	7.43	20.83	0.01
rivercess	467	3.26	4.46	0.88
rural montserrado	421	7.9	23.63	0
sinoe	487	5.14	11.58	0.24
Total	6802	6.33	17.79	0.3

**Liberia 2011**	**N**	**Mean**	**Mean**	**Mean**
	**wgtdps**	**wgtdps**	**c2dwreg**	**pvdwreg**
North Central	266	5.64	7.61	0.57
North Western	91	10.92	9.77	0.37
South Central	1002	3.12	8.78	0.46
South Eastern	119	5.08	2.76	0.97
Total	1478	4.21	8.14	0.51

Mali 2011	N wgtdps	Mean wgtdps	Mean c2dwreg	Mean pvdwreg
bamako	1205	4.35	20.55	0.01
gao	1133	2.85	8.3	0.5
kayes	1025	4.55	19.07	0.02
kidal	233	5.47	6.27	0.71
koulikoro	893	3.62	10.55	0.31
mopti	1014	3.53	11.38	0.25
segou	665	3.06	5.6	0.78
sikasso	1184	4.57	22.27	0.01
tombouctou	680	4.08	10.18	0.34
Total	8032	3.92	14.09	0.26

Mauritania 2006	N wgtdps	Mean wgtdps	Mean c2dwreg	Mean pvdwreg
Centre	417	8.33	26.05	0
Fleuve	849	4.18	13.32	0.15
Nord	230	6.18	7.91	0.54
Nouakchott	1857	3.24	17.56	0.04
SudEst	528	4.98	11.77	0.23
Total	3881	4.4	16.18	0.12

Mauritania March 2008	N wgtdps	Mean wgtdps	Mean c2dwreg	Mean pvdwreg
Centre	953	4.62	18.28	0.03
Fleuve Nord	1330	2.92	10.24	0.33
Fleuve Sud	1012	3.53	11.32	0.25
Nord	493	7.33	23.86	0
Nouakchott	1375	4.86	29.21	0
SudEst	1222	4.55	22.75	0.01
Total	6385	4.34	19.14	0.12

Mauritania Dec 2008	N wgtdps	Mean wgtdps	Mean c2dwreg	Mean pvdwreg
Centre	959	1.75	2.64	0.98
Nord	710	5.65	20.37	0.02
Nouakchott A	804	4.1	12.14	0.21
Nouakchott B	927	3.15	8.29	0.51
Sud	1071	2.12	4.32	0.89
SudEst	989	4.04	14.53	0.1
Trarza	881	4.61	16.83	0.05
Total	6341	3.5	10.77	0.42

Mauritania 2009	N wgtdps	Mean wgtdps	Mean c2dwreg	Mean pvdwreg
Centre	638	4.38	11.03	0.27
Nord	688	7.7	36.71	0
Nouakchott	679	4.6	12.91	0.17
Sud	965	2.9	7.28	0.61
Sud-est	699	5.28	17.55	0.04
Trarza	709	4.75	14.37	0.11
Total	4378	4.81	16.11	0.22

Mauritania July 2010	N wgtdps	Mean wgtdps	Mean c2dwreg	Mean pvdwreg
adrar/inchiri tiris	441	3.6	5.15	0.82
assaba	755	6.05	24.91	0
brakna	576	4.09	8.65	0.47
gorgol	723	3.34	7.25	0.61
guidimakha	632	2.69	4.11	0.9
hodh chargui	485	6.58	18.9	0.03
hodh gharbi	531	3.85	7.1	0.63
nouadhibou	505	4.61	9.67	0.38
nouakchott	505	4.81	10.5	0.31
tagant	646	2.38	3.29	0.95
trarza	584	3.56	6.65	0.67
Total	6383	4.11	9.87	0.52

Mauritania Dec 2010	N wgtdps	Mean wgtdps	Mean c2dwreg	Mean pvdwreg
adrar/inchiri tiris	408	3.79	5.28	0.81
assaba	556	4.4	9.68	0.38
brakna	567	4.09	8.54	0.48
gorgol	553	4.28	9.12	0.43
guidimakha	600	3.57	6.9	0.65
hodh chargui	471	4.24	7.62	0.57
hodh gharbi	551	4.32	9.27	0.41
nouadhibou	563	3.88	7.64	0.57
nouakchott	517	6.25	18.18	0.03
tagant	589	4.34	10	0.35
trarza	476	3.29	4.63	0.87
Total	5851	4.23	8.9	0.49

Mauritania July 2011	N wgtdps	Mean wgtdps	Mean c2dwreg	Mean pvdwreg
adrar/inchiri tiris	677	5.03	15.39	0.08
assaba	706	3.71	8.73	0.46
brakna	743	3.13	6.57	0.68
gorgol	680	3.36	6.91	0.65
guidimakha	769	5.84	23.6	0
hodh chargui	647	4.65	12.58	0.18
hodh gharbi	776	5.76	23.15	0.01
nouadhibou	674	7.89	37.78	0
nouakchott	645	3.4	6.71	0.67
tagant	742	5.41	19.51	0.02
trarza	719	4.35	12.22	0.2
Total	7778	4.79	15.87	0.26

Mauritania Dec 2011	N wgtdps	Mean wgtdps	Mean c2dwreg	Mean pvdwreg
adrar/inchiri tiris	690	2.87	5.13	0.82
assaba	763	2.69	4.98	0.84
brakna	701	2.04	2.64	0.98
gorgol	791	4.87	16.88	0.05
guidimakha	855	2.79	5.97	0.74
hodh chargui	628	3.91	8.62	0.47
hodh gharbi	803	4.2	12.75	0.17
nouadhibou	716	3.13	6.32	0.71

nouakchott	711	5.38	18.49	0.03
tagant	789	3.18	7.16	0.62
trarza	726	5.22	17.77	0.04
Total	8173	3.66	9.73	0.5

Mauritania July 2012	N	Mean	Mean	Mean
	wgtdps	wgtdps	c2dwreg	pvdwreg
adrar/inchiri tiris	451	4.86	9.6	0.38
assaba	640	3.65	7.66	0.57
brakna	573	4.71	11.45	0.25
gorgol	610	4.71	12.2	0.2
guidimakha	856	4.15	13.25	0.15
hodh chargui	670	4.43	11.85	0.22
hodh gharbi	742	3.52	8.3	0.5
nouadhibou	474	5.69	13.81	0.13
nouakchott	590	3.1	5.12	0.82
tagant	755	2.77	5.23	0.81
trarza	456	4.35	7.77	0.56
Total	6817	4.08	9.64	0.42

Niger 2012	N	Mean	Mean	Mean
	wgtdps	wgtdps	c2dwreg	pvdwreg
agadez	1222	1.83	3.7	0.93
diffa	1299	2.55	7.58	0.58
dosso	1110	3.39	11.46	0.25
maradi	1087	3.1	9.42	0.4
niamey	1161	3.24	10.95	0.28
tahoua	1468	4.78	30.22	0
tillabéri	895	5.05	20.56	0.01
zinder	926	2.41	4.84	0.85
Total	9168	3.3	12.79	0.41

Nigeria (Northern States) 2011	N	Mean	Mean	Mean
	wgtdps	wgtdps	c2dwreg	pvdwreg
jigawa	906	4.5	16.52	0.06
kano	846	3.99	12.11	0.21
katsina	959	3.42	10.1	0.34
kebbi	996	2.53	5.73	0.77
sokoto	910	2.83	6.57	0.68
yobe	1049	2.41	5.48	0.79
zamfara	961	4.42	16.91	0.05
Total	6627	3.41	10.35	0.43

Senegal 2012	N	Mean	Mean	Mean
	wgtdps	wgtdps	c2dwreg	pvdwreg
dakar	391	4.58	7.39	0.6
kolda	551	3.27	5.32	0.81
matam	1948	2.79	13.65	0.14
myf	719	1.38	1.24	1
sedhiou	2059	1.92	6.81	0.66
tambacounda	2954	2	10.65	0.3
velingara	607	3.74	7.65	0.57
Total	9229	2.4	9.04	0.46

Sierra Leone 2010	N	Mean	Mean	Mean

	wgtdps	wgtdps	c2dwreg	pvdwreg
eastern	2688	1.06	2.73	0.97
northern	5025	0.44	0.89	1
southern	3967	1.06	4.01	0.91
western	2265	2.57	13.49	0.14
Total	13945	1.08	4.18	0.83

Togo June 2012	N	Mean	Mean	Mean
	wgtdps	wgtdps	c2dwreg	pvdwreg
Centrale	404	3.73	5.06	0.83
Kara	587	5.68	17.04	0.05
Lomé	317	5.62	9.03	0.43
Maritime	460	3.37	4.7	0.86
Plateaux	360	3.04	3	0.96
Savanes	541	3.17	4.9	0.84
Total	2669	4.12	7.79	0.64

Togo Dec 2012	N	Mean	Mean	Mean
	wgtdps	wgtdps	c2dwreg	pvdwreg
Kara	612	2.97	4.86	0.85
Savanes	788	2.95	6.16	0.72
Total	1400	2.96	5.59	0.78

Digit preference score for terminal digit of weight, by region MICS

Burkina Faso 2006	N wgtdps	Mean wgtdps	Mean c2dwreg	Mean pvdwreg
Boucle du Mouhoun	387	41.67	604.65	0.00
Cascade	579	66.02	2271.09	0.00
Centre	454	71.48	2087.89	0.00
Centre-Est	315	58.19	959.89	0.00
Centre-Nord	373	81.76	2244.08	0.00
Centre-Ouest	472	34.13	494.78	0.00
Centre-Sud	349	64.94	1324.42	0.00
Est	409	65.42	1575.28	0.00
Hauts-Bassins	312	42.85	515.56	0.00
Nord	441	70.88	1993.75	0.00
Plateau-Central	398	68.61	1686.27	0.00
Sahel	362	88.74	2565.44	0.00
Sud-Ouest	296	58.61	915.07	0.00
Total	5147	62.67	1532.37	0.00

Cameroon 2006	N wgtdps	Mean wgtdps	Mean c2dwreg	Mean pvdwreg
Adamaoua	627	4.33	10.56	0.31
Centre	524	3.88	7.11	0.63
Douala	364	9.94	32.37	0.00
Est	648	3.58	7.46	0.59
Extreme Nord	715	3.82	9.41	0.40
Littoral	417	4.41	7.29	0.61
Nord	755	11.72	93.41	0.00
Nord Ouest	357	6.89	15.24	0.08
Ouest	527	6.34	19.09	0.02
Sud	415	7.42	20.59	0.01
Sud Ouest	381	3.62	4.49	0.88
Yaounde	412	6.15	14.02	0.12
Total	6142	6.01	22.6	0.31

Central African Rep 2000	N wgtdps	Mean wgtdps	Mean c2dwreg	Mean pvdwreg
Bamingui-Bangoran	451	15.86	102.15	0.00
Bangui	1529	22.42	691.79	0.00
Basse-Kotto	1007	23.39	495.81	0.00
Haut-Mbomou	352	23.83	179.93	0.00
Haute-Kotto	766	24.67	419.67	0.00
Kémo	990	16.83	252.42	0.00
Lobaye	1046	16.91	269.09	0.00
Mambéré-Kadeï	735	17.83	210.21	0.00
Mbomou	764	29.49	597.83	0.00
Nana-Grébizi	1000	19.48	341.4	0.00
Nana-Mambéré	516	27.34	347.02	0.00
Ombella-M'poko	1047	21.8	447.95	0.00
Ouaka	937	29.65	741.26	0.00
Ouham	607	18.11	179.11	0.00
Ouham-Pendé	543	26.39	340.26	0.00
Sangha-Mbaéré	688	19.65	239.01	0.00

Vakaga	588	17.06	153.97	0.00
Total	13566	21.77	395.33	0.00

Central African Rep 2006	N	Mean	Mean	Mean
	wgtdps	wgtdps	c2dwreg	pvdwreg
“Baminigui Bangoran”	514	7.73	27.63	0.00
“Bangui”	658	4.3	10.94	0.28
“Basse Kotto”	453	3.91	6.23	0.72
“Haut Mbomou”	320	8.37	20.19	0.02
“Haute Kotto”	1048	4.33	17.65	0.04
“Lobaye”	655	9.54	53.66	0.00
“Mambere Kadei”	513	4.7	10.22	0.33
“Mbomou”	537	7.87	29.91	0.00
“Nana Mambere”	1212	7.51	61.47	0.00
“Ombella Mpoko”	593	4.74	12.01	0.21
“Ouaka”	654	3.63	7.74	0.56
“Ouham Pende”	595	13.51	97.76	0.00
“Ouham”	669	3.32	6.65	0.67
“Sangha Mbaere”	901	2.81	6.4	0.70
Total	9322	5.98	27.96	0.25

Central African Rep 2010	N	Mean	Mean	Mean
	wgtdps	wgtdps	c2dwreg	pvdwreg
Baminigui Bangoran	379	5.51	10.37	0.32
Bangui	637	8.82	44.62	0.00
Basse Kotto	658	3.77	8.41	0.49
Haut Mbomou	343	5.83	10.5	0.31
Haute-Kotto	636	3.25	6.04	0.74
Kémo	830	2.74	5.61	0.78
Lobaye	835	4.68	16.46	0.06
Mambere Kadei	707	6	22.94	0.01
Mbomou	567	5.18	13.69	0.13
Nana Grebizi	653	3.22	6.1	0.73
Nana Mambéré	591	5.55	16.39	0.06
Ombella Mpoko	766	3.73	9.61	0.38
Ouaka	549	5.02	12.44	0.19
Ouham	654	2.8	4.62	0.87
Ouham Pende	672	6.22	23.39	0.01
Sangha Mbaere	779	4.11	11.82	0.22
Vakaga	48	15.77	10.75	0.29
Total	10304	4.72	13.98	0.34

Chad 2000	N	Mean	Mean	Mean
	wgtdps	wgtdps	c2dwreg	pvdwreg
Autres villes	2000	1.48	3.93	0.92
N'Djaména	846	4.11	12.84	0.17
Rural	2538	2.9	19.17	0.02
Total	5384	2.56	12.51	0.38

Chad 2010	N	Mean	Mean	Mean
	wgtdps	wgtdps	c2dwreg	pvdwreg
Barh El Gazal	662	3.75	8.36	0.50
Bhata	830	3.68	10.12	0.34
Chari Baguirmi	716	5.28	17.94	0.04
Guéra	1298	6.28	46.02	0.00

Hadjer Lamis	740	4.34	12.54	0.18
Kanem	614	2.98	4.89	0.84
Lac	629	4.93	13.75	0.13
Logone Occidental	639	6.76	26.31	0.00
Logone Oriental	872	7.77	47.4	0.00
Mandoul	805	1.73	2.17	0.99
Mayo Kebbi Est	1099	3.83	14.53	0.10
Mayo Kebbi Ouest	808	3.74	10.17	0.34
Moyen Chari	679	8.38	42.96	0.00
Ndjaména	829	5.43	22.01	0.01
Ouaddai	807	10.85	85.43	0.00
Salamat	759	9.31	59.25	0.00
Sila	809	7.74	43.65	0.00
Tandjilé	904	4.07	13.48	0.14
Wad Fira	642	4.46	11.49	0.24
bet	459	5.42	12.13	0.21
Total	15600	5.55	26.49	0.19

**Congo DR 2001**	**N**	**Mean**	**Mean**	**Mean**
	**wgtdps**	**wgtdps**	**c2dwreg**	**pvdwreg**
Bandundu	1181	5.35	30.39	0.00
Bas-congo	539	2.17	2.28	0.99
Equateur	1158	6.48	43.8	0.00
Kasai Occidental	954	4.35	16.23	0.06
Kasai Oriental	1078	4.06	16.01	0.07
Katanga	1138	4.18	17.92	0.04
Kinshasa	1446	2.74	9.8	0.37
Maniema	256	8.95	18.45	0.03
Nord-Kivu	718	6.09	24.01	0.00
Orientale	1014	7.68	53.83	0.00
Sud-Kivu	602	13.98	105.84	0.00
Total	10084	5.47	29.29	0.12

**Congo DR 2010**	**N**	**Mean**	**Mean**	**Mean**
	**wgtdps**	**wgtdps**	**c2dwreg**	**pvdwreg**
Bandundu	962	4.15	14.9	0.09
Bas congo	754	3.67	9.16	0.42
Equateur	1014	5.81	30.75	0.00
Kasai Occidental	1070	4.31	17.85	0.04
Kasai Oriental	1015	2.81	7.24	0.61
Katanga	1242	2.76	8.5	0.48
Kinshasa	804	5.04	18.39	0.03
Maniema	977	1.42	1.78	0.99
Nord Kivu	1118	4.49	20.32	0.02
Province Orientale	859	2.54	4.99	0.83
Sud Kivu	1088	3.04	9.02	0.44
Total	10903	3.62	13.09	0.36

**Côte d'Ivoire 2006**	**N**	**Mean**	**Mean**	**Mean**
	**wgtdps**	**wgtdps**	**c2dwreg**	**pvdwreg**
Centre	663	3.56	7.54	0.58
Centre Est	544	5.2	13.24	0.15
Centre Nord	760	4.97	16.87	0.05
Centre Ouest	798	5.18	19.24	0.02
Nord	652	3.9	8.92	0.44

Nord Est	553	13.81	94.94	0.00
Nord Ouest	925	6.69	37.26	0.00
Ouest	706	4.77	14.48	0.11
Sud (sans ville d' Abidjan)	882	5.6	24.89	0.00
Sud Ouest	1126	3.88	15.24	0.08
Ville Abidjan	979	9.41	77.95	0.00
Total	8588	5.98	30.14	0.11

Equatorial Guinea 2000	N	Mean	Mean	Mean
	wgtdps	wgtdps	c2dwreg	pvdwreg
Annobon	14	12.6	2	0.96
Bioko Norte	570	7.15	26.25	0.00
Bioko Sur	32	17.31	8.63	0.47
Centro Sur	265	12.56	37.6	0.00
Kie Ntem	493	5.5	13.43	0.14
Litoral	707	10.57	71.15	0.00
Wele Nzas	306	4.28	5.05	0.83
Total	2387	8.22	35.06	0.15

Gambia 2000	N	Mean	Mean	Mean
	wgtdps	wgtdps	c2dwreg	pvdwreg
Banjul	479	14.16	86.41	0.00
Basse	462	6.75	18.95	0.03
Brikama	451	15.51	97.58	0.00
Janjabureh	364	14.35	67.43	0.00
Kanifing	480	18.2	143.04	0.00
Kerewan	454	9.53	37.15	0.00
Kuntaur	574	5.22	14.08	0.12
Mansakonko	360	25.82	216.06	0.00
Total	3624	13.1	80.05	0.02

Gambia 2005	N	Mean	Mean	Mean
	wgtdps	wgtdps	c2dwreg	pvdwreg
Banjul	160	9.99	14.38	0.11
Basse	1068	2.71	7.07	0.63
Brikama	1367	3.59	15.83	0.07
Janjanburay	753	2.57	4.46	0.88
Kanifing	1416	5.75	42.11	0.00
Kerewan	853	5.02	19.32	0.02
Kuntaur	410	5	9.22	0.42
Mansakonko	398	3.35	4.01	0.91
Total	6425	4.22	18.11	0.31

Ghana 2006	N	Mean	Mean	Mean
	wgtdps	wgtdps	c2dwreg	pvdwreg
Ashanti	411	3.33	4.11	0.90
Brong Ahafo	241	6.73	9.83	0.36
Central	258	5.86	7.97	0.54
Eastern	333	4.43	5.89	0.75
Greater Accra	305	6.17	10.44	0.32
Northern	572	3.68	6.99	0.64
Upper East	377	5.42	9.98	0.35
Upper West	366	3.99	5.26	0.81
Volta	226	10.33	21.7	0.01
Western	315	3.7	3.89	0.92

Total	3404	4.99	7.95	0.60

**Ghana 2011**	**N**	**Mean**	**Mean**	**Mean**
	**wgtdps**	**wgtdps**	**c2dwreg**	**pvdwreg**
Asante	462	3.95	6.48	0.69
Brong Ahafo	403	4.71	8.04	0.53
Central	988	2.46	5.38	0.80
Eastern	339	5.23	8.35	0.50
Greater Accra	386	4.93	8.46	0.49
Northern	1976	2	7.13	0.62
Upper East	985	3.71	12.21	0.20
Upper West	1127	2.37	5.72	0.77
Volta	399	9.2	30.4	0.00
Western	397	5.89	12.4	0.19
Total	7462	3.5	9.01	0.54

**Guinea Bissau 2000**	**N**	**Mean**	**Mean**	**Mean**
	**wgtdps**	**wgtdps**	**c2dwreg**	**pvdwreg**
Bafatá	928	23.92	477.69	0.00
Biombo	300	13.96	52.6	0.00
Bolama/Bijagós	167	7.09	7.55	0.58
Cacheu	771	6.8	32.1	0.00
Gabú	748	30.29	617.59	0.00
Oio	1129	4.86	24.03	0.00
Quinará	239	3.61	2.8	0.97
Tombali	318	5.13	7.53	0.58
sab	1134	3.72	14.1	0.12
Total	5734	11.8	173.22	0.11

**Guinea Bissau 2006**	**N**	**Mean**	**Mean**	**Mean**
	**wgtdps**	**wgtdps**	**c2dwreg**	**pvdwreg**
EST (Bafata e Gabu)	1331	12.68	192.49	0.00
NORD (Biombo, Cacheu e Oio)	2419	12.73	352.74	0.00
SAB Capital	815	47.33	1642.88	0.00
SUD (Bolama, Quinara e Tombali)	1090	20.96	431.14	0.00
Total	5655	19.29	516.07	0.00

**Mauritania 2007**	**N**	**Mean**	**Mean**	**Mean**
	**wgtdps**	**wgtdps**	**c2dwreg**	**pvdwreg**
Adrar	300	6.5	11.4	0.25
Assaba	885	3.25	8.44	0.49
Brakna	849	5.37	22.06	0.01
Gorgol	863	4.77	17.68	0.04
Guidimagha	816	3.16	7.33	0.60
Hodh ECharghi	707	3.28	6.85	0.65
Hodh ELGharbi	698	3.77	8.91	0.45
Inchiri	124	10.56	12.45	0.19
Nouadhibou	584	2.92	4.49	0.88
Nouakchott	1360	3.21	12.65	0.18
Tagant	292	8.13	17.38	0.04
Tiris Zemmour	298	5.61	8.44	0.49
Trarza	593	4.18	9.34	0.41
Total	8369	4.18	11.52	0.36

**Niger 2000**	**N**	**Mean**	**Mean**	**Mean**

	wgtdps	wgtdps	c2dwreg	pvdwreg
Diffa	68	12.75	9.94	0.36
Agadez	420	5.32	10.71	0.30
Dosso	618	3.15	5.53	0.79
Maradi	1207	2.67	7.72	0.56
Niamey	731	4.49	13.28	0.15
Tahoua	732	4.94	16.09	0.07
Tillaberi	644	3.77	8.24	0.51
Zinder	660	4.58	12.48	0.19
Total	5080	4.06	10.42	0.38

Nigeria 2007	N	Mean	Mean	Mean
	wgtdps	wgtdps	c2dwreg	pvdwreg
Abia	332	10.96	35.89	0.00
Abuja FCT	429	14.09	76.66	0.00
Adamawa	411	28.79	306.69	0.00
Akwa-Ibom	470	6.63	18.6	0.03
Anambra	311	15.51	67.3	0.00
Bauchi	786	19.86	279.01	0.00
Bayelsa	482	4.99	10.82	0.29
Benue	487	5.26	12.12	0.21
Borno	457	29.06	347.27	0.00
Cross-Rivers	342	4.4	5.95	0.74
Delta	329	7.87	18.33	0.03
Ebonyi	448	7.15	20.62	0.01
Edo	372	5.65	10.69	0.30
Ekiti	271	8.51	17.67	0.04
Enugu	332	6.35	12.04	0.21
Gombe	400	56.31	1141.6	0.00
Imo	260	11.09	28.77	0.00
Jigawa	819	6.56	31.73	0.00
Kaduna	679	29.96	548.7	0.00
Kano	596	18.75	188.66	0.00
Katsina	546	18.64	170.7	0.00
Kebbi	505	31.32	445.87	0.00
Kogi	312	6.77	12.87	0.17
Kwara	320	7.74	17.25	0.04
Lagos	341	8.52	22.26	0.01
Nasarawa	466	9.97	41.73	0.00
Niger	585	18.53	180.79	0.00
Ogun	283	6.23	9.9	0.36
Ondo	329	9.22	25.19	0.00
Osun	230	5.1	5.39	0.80
Oyo	338	7.97	19.34	0.02
Plataeu	449	9.51	36.55	0.00
Rivers	286	29.57	225.05	0.00
Sokoto	447	23.45	221.17	0.00
Taraba	501	17.99	145.97	0.00
Yobe	714	22.83	334.99	0.00
Zamfara	828	24.51	447.77	0.00
Total	16493	16.12	178.08	0.07

Nigeria 2011	N	Mean	Mean	Mean
	wgtdps	wgtdps	c2dwreg	pvdwreg
Abia	459	7.29	21.98	0.01

Adamawa	784	3.2	7.22	0.61
Akwa ibom	546	5.29	13.74	0.13
Anambra	547	5.71	16.05	0.07
Bauchi	932	6.92	40.15	0.00
Bayelsa	521	4.64	10.07	0.34
Benue	605	5.72	17.79	0.04
Borno	677	5.16	16.22	0.06
Cross River	556	4.45	9.9	0.36
Delta	527	4.61	10.06	0.35
Ebonyi	647	2.53	3.74	0.93
Edo	506	4.48	9.14	0.42
Ekiti	375	5.45	10.04	0.35
Enugu	348	4.53	6.43	0.70
FCT (Abuja)	603	6.29	21.46	0.01
Gombe	907	4.76	18.48	0.03
Imo	406	3.16	3.66	0.93
Jigawa	1006	3.62	11.85	0.22
Kaduna	925	5.1	21.69	0.01
Kano	899	2.66	5.73	0.77
Katsina	972	4.89	20.92	0.01
Kebbi	874	5.9	27.37	0.00
Kogi	423	5.17	10.17	0.34
Kwara	546	5.76	16.31	0.06
Lagos	513	6.14	17.39	0.04
Nasarawa	744	2.9	5.65	0.77
Niger	900	6.2	31.16	0.00
Ogun	536	4.7	10.64	0.30
Ondo	403	11.66	49.28	0.00
Osun	443	4.35	7.54	0.58
Oyo	551	3.76	7.02	0.63
Plateau	638	5.32	16.23	0.06
Rivers	452	4.8	9.37	0.40
Sokoto	1007	6.55	38.93	0.00
Taraba	669	4.18	10.51	0.31
Yobe	933	4.43	16.49	0.06
Zamfara	972	6.42	36.11	0.00
Total	24352	5.05	17.71	0.24

**Sao Tome et Principe 2000**	**N**	**Mean**	**Mean**	**Mean**
	**wgtdps**	**wgtdps**	**c2dwreg**	**pvdwreg**
Centro	1557	47.09	3106.77	0.00
Norte	442	46.87	874.02	0.00
Principe	103	38.5	137.38	0.00
Sul	105	42.38	169.76	0.00
Total	2207	46.42	2381.3	0.00

**Senegal 2000**	**N**	**Mean**	**Mean**	**Mean**
	**wgtdps**	**wgtdps**	**c2dwreg**	**pvdwreg**
Dakar	984	18.94	317.71	0.00
Diourbel	737	21.41	304.04	0.00
Fatick	875	9.18	66.34	0.00
Kaolack	941	19.67	327.72	0.00
Kolda	640	4.62	12.28	0.20
Louga	889	2.96	7.03	0.63
Saint louis	868	3.58	10	0.35

Tambacounda	852	4.91	18.49	0.03
Thies	1148	6.51	43.72	0.00
Ziguinchor	726	4.34	12.32	0.20
Total	8660	9.75	115.57	0.13

Sierra Leone 2000	N	Mean	Mean	Mean
	wgtdps	wgtdps	c2dwreg	pvdwreg
East	624	5.51	17.06	0.05
North	891	5.34	22.84	0.01
South	355	5.75	10.55	0.31
West	592	8.93	42.49	0.00
Total	2462	6.3	24.33	0.06

Sierra Leone 2005	N	Mean	Mean	Mean
	wgtdps	wgtdps	c2dwreg	pvdwreg
East	1133	6.3	40.46	0.00
North	2078	4.21	33.15	0.00
South	1483	2.33	7.23	0.61
West	513	3.66	6.2	0.72
Total	5207	4.07	24.71	0.25

Sierra Leone 2010	N	Mean	Mean	Mean
	wgtdps	wgtdps	c2dwreg	pvdwreg
East	1832	3.2	16.84	0.05
North	3207	4.92	69.76	0.00
South	2296	4.14	35.39	0.00
West	1050	10.11	96.59	0.00
Total	8385	4.98	52.15	0.01

Togo 2006	N	Mean	Mean	Mean
	wgtdps	wgtdps	c2dwreg	pvdwreg
Centrale	594	13.51	97.62	0.00
Kara	584	57.63	1745.69	0.00
Lomé commune	515	5.84	15.82	0.07
Maritime (sans Lomé commune)	810	2.89	6.07	0.73
Plateaux	528	11.38	61.58	0.00
Savanes	973	45.56	1817.33	0.00
Total	4004	24.32	722.11	0.16

Togo 2010	N	Mean	Mean	Mean
	wgtdps	wgtdps	c2dwreg	pvdwreg
Centrale	743	2.26	3.42	0.95
Kara	825	2.76	5.65	0.77
Lomé	466	4.11	7.09	0.63
Maritime	772	4.13	11.83	0.22
Plateaux	792	3.02	6.51	0.69
Savanes	1097	2.87	8.13	0.52
Total	4695	3.11	7.18	0.62

Appendix E Digit preference score for terminal digit of mid-upper arm circumference, NNS

Country, survey year	N	DPS	Chi-sq	p-value
Benin 2008	3552	7.08	154.99	0
Burkina Faso 2012	19354	1.07	17.51	0.04
Cameroon 2011	1481	5.34	34.46	0
Central African Rep 2012	15741	4.13	210.32	0
Chad June 2012	7777	1.63	17.05	0.05
Chad (7 regions) Dec/Jan 2012-13	5799	2.15	21.19	0.01
The Gambia 2012	7779	2.69	45.13	0
Guinea-Bissau 2008	2718	3.82	35.63	0
Guinée Conakay 2012	8208	8.12	426.56	0
Liberia 2010	6806	3.17	61.32	0
Liberia 2011	1484	6.71	59.9	0
Mali 2011	8231	3.35	75.82	0
Mauritania 2006	0			
Mauritania March 2008	0			
Mauritania Dec 2008	6342	4.3	105.26	0
Mauritania 2009	0			
Mauritania July 2010	0			
Mauritania Dec 2010	5851	3.13	51.55	0
Mauritania July 2011	7780	3.82	101.97	0
Mauritania Dec 2011	8173	4.2	129.59	0
Mauritania July 2012	6817	5	152.22	0
Niger 2012	0			
Nigeria (Northern States) 2011	6909	4.36	113.49	0
Senegal 2012	9715	2.79	59.7	0
Sierra Leone 2010	14027	1.32	21.79	0.01
Togo June 2012	2780	1.66	5.96	0.74
Togo Dec 2012	1451	2.85	9.26	0.41
Total	158775	3.33	95.39	0.03

Appendix F

Digit preference score for terminal digit of mid-upper arm circumference, by region NNS

Benin 2008	N	Mean	Mean	Mean
	muacdps	muacdps	c2dmreg	pvdmreg
alibori	368	5.8	11.13	0.27
atacora	348	11.01	37.98	0
atlantique	306	11.37	35.63	0
borgou	326	5.98	10.5	0.31
collines	247	16.52	60.65	0
couffo	393	11.95	50.54	0
donga	259	12.02	33.7	0
littoral	220	11.74	27.27	0
mono	243	7.95	13.83	0.13
oueme	264	15.88	59.94	0
plateau	225	12.8	33.18	0
zou	236	12.75	34.51	0
Total	3435	11.03	33.77	0.07

Burkina Faso 2012	N	Mean	Mean	Mean
	muacdps	muacdps	c2dmreg	pvdmreg
bales	681	1.52	1.42	1
bam	734	1.74	1.99	0.99
banwa	561	2.05	2.12	0.99
banwa ganzourgou	746	2.84	5.42	0.8
bazega	524	3.2	4.82	0.85
boulgou	604	4.97	13.45	0.14
cascades	554	1.73	1.49	1
centre ouest	523	3.47	5.66	0.77
est	788	3.02	6.47	0.69
houet	424	3.67	5.15	0.82
kadiogo	385	5.07	8.9	0.45
kenedougou	660	2.29	3.12	0.96
kossi	663	4.4	11.56	0.24
koulpelogo	744	2.84	5.41	0.8
kouritenga	635	5.22	15.57	0.08
kourweogo	587	2.99	4.74	0.86
mouhoun	571	4.41	10	0.35
nahouri	435	2.62	2.68	0.98
namentenga	764	1.59	1.73	1
nayala	573	4.36	9.81	0.37
nord	644	2.12	2.61	0.98
oubritenga	592	2	2.12	0.99
sahel	733	1.2	0.96	1
sanmentenga	781	1.66	1.93	0.99
sourou	595	4.85	12.58	0.18
sud ouest	445	3.25	4.24	0.9
tuy	505	1.65	1.24	1
zoundweogo	546	0.83	0.34	1
Total	16997	2.84	5.2	0.76

Cameroon 2011	N muacdps	Mean muacdps	Mean c2dmreg	Mean pvdmreg
extrême-nord	663	7.29	31.71	0
nord	680	6.75	27.91	0
Total	1343	7.02	29.78	0

Central African Rep 2012	N muacdps	Mean muacdps	Mean c2dmreg	Mean pvdmreg
bamingui bangoran	799	9.04	58.71	0
bangui	697	5.62	19.8	0.02
basse kotto	819	2.95	6.43	0.7
haut kotto	831	7.97	47.54	0
haut mbomou	828	11.3	95.21	0
kemo	803	5.43	21.3	0.01
lobaye	948	4.77	19.41	0.02
mambere kadei	1143	7.45	57.13	0
mbomou	828	6.31	29.66	0
nana grebizi	916	4.12	14.02	0.12
nana mambere	575	7.92	32.46	0
ombella mpoko	786	6.8	32.75	0
ouaka	740	3.75	9.38	0.4
ouham	886	4.37	15.24	0.08
ouham pende	678	5.39	17.72	0.04
sangha mbarere	951	6.45	35.57	0
vakaga	448	13.7	75.71	0
Total	13676	6.47	34.16	0.08

Chad June 2012	N muacdps	Mean muacdps	Mean c2dmreg	Mean pvdmreg
Barh El Ghazal	719	4.19	11.33	0.25
Batha	681	2.57	4.04	0.91
Guéra	729	3.93	10.11	0.34
Hadjer Lamis	607	3.6	7.09	0.63
Kanem	559	5.51	15.26	0.08
Lac	528	4.27	8.67	0.47
N'Djamena	722	5.39	18.86	0.03
Ouaddai	557	4.57	10.49	0.31
Salamat	696	3.99	9.98	0.35
Sila	677	3.46	7.3	0.61
Wadi Fira	697	5.06	16.07	0.07
Total	7172	4.21	10.91	0.37

Chad (7 regions) Dec/Jan 2012-13	N muacdps	Mean muacdps	Mean c2dmreg	Mean pvdmreg
Logone Occidental	724	5.21	17.69	0.04
Logone Oriental	722	4.24	11.68	0.23
Mandoul	709	3.89	9.63	0.38
Mayo-Kebbi Est	890	3.12	7.78	0.56
Mayo-Kebbi Ouest	565	4.76	11.51	0.24
Moyen-Chari	660	4.26	10.76	0.29
Tandjilé	801	7.26	38.04	0
Total	5071	4.67	15.59	0.25

The Gambia 2012	N muacdps	Mean muacdps	Mean c2dmreg	Mean pvdmreg

banjul	669	4.75	13.59	0.14
basse	1194	4.26	19.48	0.02
brikama	759	4.42	13.35	0.15
janjanburay	905	4.63	17.44	0.04
kanifing	674	4.95	14.84	0.1
kerewan	975	3.69	11.94	0.22
kuntaur	1000	6.67	40	0
mansakonko	746	6.81	31.16	0
Total	6922	4.98	20.68	0.08

**Guinea-Bissau 2008**	**N**	**Mean**	**Mean**	**Mean**
	**muacdps**	**muacdps**	**c2dmreg**	**pvdmreg**
Capitale	445	9.84	38.75	0
Est (Bafata e Gabu)	834	6.22	29	0
Nord (Biombo, Cacheu e Oio)	752	5.5	20.47	0.02
Sud (Bolama, Quinara e Tombali)	684	4.5	12.49	0.19
Total	2715	6.18	24.08	0.05

**Guinée Conakay 2012**	**N**	**Mean**	**Mean**	**Mean**
	**muacdps**	**muacdps**	**c2dmreg**	**pvdmreg**
boke nord	764	5.22	18.75	0.03
boke sud	628	14.39	117.06	0
conakry	767	11.56	92.28	0
faranah	617	7	27.2	0
kankan	728	9.39	57.74	0
kindia	770	23.3	376.36	0
labe	938	6.67	37.59	0
mamou	991	5.06	22.81	0.01
nzerekore	981	18.88	314.77	0
Total	7184	11.25	121.64	0

**Liberia 2010**	**N**	**Mean**	**Mean**	**Mean**
	**muacdps**	**muacdps**	**c2dmreg**	**pvdmreg**
bomi	334	7.48	16.84	0.05
bong	477	8.83	33.5	0
gbarpolu	369	10.49	36.56	0
grand bassa	405	8.22	24.6	0
grand cape mount	498	12.64	71.6	0
grand gedeh	443	12.28	60.14	0
grand kru	448	11.27	51.2	0
lofa	481	6.97	21.02	0.01
margibi	385	14.62	74.09	0
maryland	372	6.27	13.16	0.16
montserrado	350	7.04	15.6	0.08
nimba	441	12.04	57.57	0
river gee	420	5.88	13.05	0.16
rivercess	467	9.13	35.03	0
rural montserrado	421	5.57	11.76	0.23
sinoe	487	11.13	54.33	0
Total	6798	9.46	37.94	0.04

**Liberia 2011**	**N**	**Mean**	**Mean**	**Mean**
	**muacdps**	**muacdps**	**c2dmreg**	**pvdmreg**
North Central	266	4.42	4.68	0.86
North Western	91	13.55	15.04	0.09

South Central	1002	7.09	45.37	0
South Eastern	119	19.1	39.07	0
Total	1478	7.98	35.67	0.16

Mali 2011	N	Mean	Mean	Mean
	muacdps	muacdps	c2dmreg	pvdmreg
bamako	1151	10.1	105.69	0
gao	1085	4.06	16.06	0.07
kayes	988	5.92	31.17	0
kidal	215	4.66	4.21	0.9
koulikoro	833	8.21	50.49	0
mopti	908	8.87	64.29	0
segou	620	7.16	28.65	0
sikasso	1083	3.98	15.4	0.08
tombouctou	631	7.52	32.09	0
Total	7514	6.82	43.37	0.05

Mauritania 2006	N	Mean	Mean	Mean
	muacdps	muacdps	c2dmreg	pvdmreg
Centre	958	5.31	24.34	0
Nord	698	5.58	19.59	0.02
Nouakchott A	802	6.79	33.31	0
Nouakchott B	922	4.26	15.03	0.09
Sud	1072	4	15.41	0.08
SudEst	989	4.74	19.97	0.02
Trarza	880	11.66	107.7	0
Total	6321	5.95	33	0.03

Mauritania March 2008	N	Mean	Mean	Mean
	muacdps	muacdps	c2dmreg	pvdmreg
adrar/inchiri tiris	408	7.49	20.63	0.01
assaba	556	7.85	30.8	0
brakna	566	7.37	27.64	0
gorgol	553	6.8	23.04	0.01
guidimakha	599	5.87	18.58	0.03
hodh chargui	471	4.42	8.3	0.5
hodh gharbi	551	11.99	71.34	0
nouadhibou	563	6.03	18.4	0.03
nouakchott	516	10.07	47.1	0
tagant	589	10.18	54.96	0
trarza	475	11.26	54.24	0
Total	5847	8.12	34.39	0.05

Mauritania Dec 2008	N	Mean	Mean	Mean
	muacdps	muacdps	c2dmreg	pvdmreg
adrar/inchiri tiris	677	15.71	150.46	0
assaba	706	8.66	47.65	0
brakna	743	4.92	16.21	0.06
gorgol	680	4.08	10.18	0.34
guidimakha	768	12.69	111.32	0
hodh chargui	646	11.94	82.85	0
hodh gharbi	776	5.17	18.66	0.03
nouadhibou	674	4.57	12.65	0.18
nouakchott	645	6.22	22.49	0.01
tagant	742	5.72	21.88	0.01

trarza	719	4.54	13.36	0.15
Total	7776	7.62	45.89	0.07

**Mauritania 2009**	**N**	**Mean**	**Mean**	**Mean**
	**muacdps**	**muacdps**	**c2dmreg**	**pvdmreg**
adrar/inchiri tiris	689	5.01	15.54	0.08
assaba	763	9.19	57.96	0
brakna	700	4.55	13.03	0.16
gorgol	791	7.92	44.61	0
guidimakha	855	16.86	218.86	0
hodh chargui	626	4.56	11.7	0.23
hodh gharbi	802	4.49	14.58	0.1
nouadhibou	716	3.22	6.68	0.67
nouakchott	711	5.22	17.42	0.04
tagant	788	6.64	31.29	0
trarza	725	9.56	59.62	0
Total	8166	7.22	47.82	0.11

**Mauritania July 2010**	**N**	**Mean**	**Mean**	**Mean**
	**muacdps**	**muacdps**	**c2dmreg**	**pvdmreg**
adrar/inchiri tiris	445	11.38	51.83	0
assaba	634	8.19	38.24	0
brakna	567	11.87	71.89	0
gorgol	610	9.05	44.95	0
guidimakha	854	5.54	23.59	0.01
hodh chargui	666	6.24	23.34	0.01
hodh gharbi	729	7.49	36.8	0
nouadhibou	472	10.6	47.75	0
nouakchott	588	6.8	24.48	0
tagant	755	7.4	37.23	0
trarza	448	3.72	5.57	0.78
Total	6768	7.85	36.28	0.05

**Mauritania Dec 2010**	**N**	**Mean**	**Mean**	**Mean**
	**muacdps**	**muacdps**	**c2dmreg**	**pvdmreg**
jigawa	904	4.92	19.67	0.02
kano	843	7.21	39.41	0
katsina	958	5.65	27.55	0
kebbi	996	4.24	16.11	0.06
sokoto	910	5.87	28.26	0
yobe	1048	4.93	22.95	0.01
zamfara	961	5.09	22.44	0.01
Total	6620	5.37	24.89	0.01

**Mauritania July 2011**	**N**	**Mean**	**Mean**	**Mean**
	**muacdps**	**muacdps**	**c2dmreg**	**pvdmreg**
dakar	349	4.61	6.67	0.67
kolda	506	8.75	34.83	0
matam	1946	2.87	14.44	0.11
myf	655	4.05	9.69	0.38
sedhiou	1806	3.5	19.95	0.02
tambacounda	2701	3.46	29.04	0
velingara	555	8.91	39.61	0
Total	8518	4.1	22.41	0.09

Mauritania Dec 2011	N	Mean	Mean	Mean
	muacdps	muacdps	c2dmreg	pvdmreg
eastern	2685	3.83	35.51	0
northern	5024	0.55	1.35	1
southern	3966	0.94	3.15	0.96
western	2266	2.77	15.63	0.07
Total	13941	1.65	10.76	0.64

Mauritania July 2012	N	Mean	Mean	Mean
	muacdps	muacdps	c2dmreg	pvdmreg
Centrale	364	4.24	5.89	0.75
Kara	528	4.79	10.9	0.28
Lomé	285	3.71	3.53	0.94
Maritime	416	3.86	5.59	0.78
Plateaux	318	4.29	5.27	0.81
Savanes	494	4.95	10.9	0.28
Total	2405	4.39	7.6	0.59

Niger 2012	N	Mean	Mean	Mean
	muacdps	muacdps	c2dmreg	pvdmreg
Kara	558	3.39	5.76	0.76
Savanes	713	5.12	16.8	0.05
Total	1271	4.36	11.96	0.36

Appendix G Mean, standard deviation (SD), skewness, and kurtosis for height-for-age (HAZ), weight-for-age (WTZ), and weight-for-height (WHZ) based on the WHO 2006 reference standard, NHANES

	N	Mean	SD	Skewness	Kurtosis
HAZ	5755	0.03	1.08	0.03	3.64
WAZ	5933	0.4	1.06	0.13	3.9
WHZ	5726	0.54	1.03	0.31	3.75

Data drawn from four waves of NHANES from 2003-2010

Appendix H

Mean and standard deviation (SD) for height-for-age (HAZ), weight-for-age (WTZ), and weight-for-height (WHZ), by region DHS

Benin 2001	Mean	Mean	Mean	Sd	Sd	Sd
	who htz	who wtz	who wfl	who htz	who wtz	who wfl
atacora	-1.618	-1.337	-0.497	1.737	1.292	1.368
atlantique	-1.355	-0.885	-0.119	1.54	1.218	1.261
borgou	-1.615	-1.3	-0.473	1.891	1.362	1.458
mono	-1.649	-1.074	-0.133	1.547	1.092	1.232
oueme	-1.377	-1.048	-0.346	1.542	1.178	1.295
zou	-1.502	-1.07	-0.25	1.585	1.226	1.317
Total	-1.509	-1.108	-0.296	1.648	1.245	1.332

Benin 2006	Mean	Mean	Mean	Sd	Sd	Sd
	who htz	who wtz	who wfl	who htz	who wtz	who wfl
alibori	-2.557	-1.591	0.528	2.133	1.439	1.936
atacora	-1.465	-1.279	-0.59	1.88	1.385	1.482
atlantique	-1.561	-1.055	-0.269	1.736	1.217	1.403
borgou	-1.678	-1.018	-0.037	2.027	1.385	1.657
collines	-1.755	-0.905	0.191	1.794	1.238	1.525
couffo	-1.588	-0.939	0.004	1.357	1.131	1.178
donga	-1.346	-1.039	-0.294	2.164	1.462	1.716
littoral	-1.123	-0.639	0.056	1.554	1.244	1.395
mono	-1.878	-0.89	0.29	1.669	1.183	1.489
plateau	-1.662	-1.036	-0.011	1.909	1.375	1.579
quémé	-1.513	-0.891	0.027	1.747	1.249	1.504
zou	-2.315	-0.77	0.981	1.825	1.214	1.799
Total	-1.711	-1.009	0.077	1.86	1.313	1.623

Benin 2011	Mean	Mean	Mean	Sd	Sd	Sd
	who htz	who wtz	who wfl	who htz	who wtz	who wfl
Alibori	-2.099	-1.255	0.001	2.411	1.649	2.163
Atacora	-1.784	-0.99	0.087	2.52	1.734	2.103
Atlantique	-2.03	-0.387	0.249	2.576	2.023	2.288
Borgou	-1.721	-1.238	-0.172	2.563	2.013	2.221
Collines	-1.739	-0.987	0.177	2.546	1.586	2.344
Couffo	-1.148	-0.8	-0.17	2.158	1.485	1.727
Donga	-2.065	-1.04	0.335	2.343	1.486	2.142
Littoral	-1.741	-0.284	0.413	2.48	2.086	2.115
Mono	-1.731	-1.12	-0.033	2.255	1.528	1.793
Ou?m?	-1.455	-0.493	0.335	2.396	1.716	2.048
Plateau	-2.75	-0.845	0.495	2.387	2.511	2.535
Zou	-1.509	-0.942	-0.122	2.539	1.816	2.272
Total	-1.773	-0.826	0.121	2.47	1.857	2.154

Burkina Faso 1993	Mean	Mean	Mean	Sd	Sd	Sd
	who htz	who wtz	who wfl	who htz	who wtz	who wfl
central/south	-1.536	-1.423	-0.732	1.746	1.47	1.486
east	-1.662	-1.307	-0.448	1.69	1.465	1.509
north	-1.43	-1.283	-0.605	1.729	1.362	1.349
ouagadougou	-0.938	-0.851	-0.456	1.561	1.252	1.295

west	-1.4	-1.29	-0.663	1.646	1.34	1.337
Total	-1.398	-1.241	-0.592	1.691	1.394	1.402

**Burkina Faso 1998**	**Mean**	**Mean**	**Mean**	**Sd**	**Sd**	**Sd**
	**who htz**	**who wtz**	**who wfl**	**who htz**	**who wtz**	**who wfl**
central/south	-1.73	-1.461	-0.663	1.74	1.341	1.381
east	-1.894	-1.695	-0.719	1.752	1.399	1.356
north	-1.706	-1.65	-0.847	2.038	1.514	1.417
ouagadougou	-0.88	-0.881	-0.473	1.569	1.23	1.219
west	-1.759	-1.49	-0.585	1.826	1.36	1.324
Total	-1.697	-1.504	-0.666	1.811	1.394	1.354

**Burkina Faso 2003**	**Mean**	**Mean**	**Mean**	**Sd**	**Sd**	**Sd**
	**who htz**	**who wtz**	**who wfl**	**who htz**	**who wtz**	**who wfl**
boucle de mouhoun	-1.448	-1.722	-1.104	1.792	1.504	1.577
cascades	-1.733	-1.827	-0.996	1.919	1.687	1.85
centre (sans ouagadougou)	-1.501	-1.215	-0.588	1.854	1.456	1.467
centre-est	-1.608	-1.686	-0.881	1.862	1.6	1.755
centre-nord	-1.659	-1.226	-0.355	1.983	1.334	1.806
centre-ouest	-1.539	-1.558	-0.905	1.835	1.32	1.448
centre-sud	-1.367	-1.535	-0.809	2.105	1.811	1.805
est	-2.409	-1.441	0.356	2.138	1.558	2.193
hauts bassins	-1.249	-1.16	-0.56	1.836	1.29	1.406
nord	-1.424	-1.558	-0.945	1.942	1.515	1.509
ouagadougou	-0.845	-0.878	-0.476	1.494	1.274	1.388
plateau central	-1.54	-1.933	-1.251	1.989	1.611	1.789
sahel	-1.985	-1.835	-0.871	1.888	1.624	1.591
sud-ouest	-1.604	-1.705	-1.035	1.954	1.5	1.54
Total	-1.59	-1.563	-0.771	1.945	1.537	1.723

**Burkina Faso 2010**	**Mean**	**Mean**	**Mean**	**Sd**	**Sd**	**Sd**
	**who htz**	**who wtz**	**who wfl**	**who htz**	**who wtz**	**who wfl**
boucle de mouhoun	-1.296	-1.135	-0.587	1.497	1.118	1.251
cascades	-1.454	-1.043	-0.283	1.847	1.395	1.541
centre	-0.934	-1.008	-0.512	1.599	1.156	1.583
centre-est	-1.313	-1.472	-0.882	1.756	1.259	1.467
centre-nord	-0.98	-1.195	-0.869	1.803	1.156	1.645
centre-ouest	-1.484	-1.319	-0.638	1.497	1.133	1.259
centre-sud	-1.174	-1.012	-0.546	1.8	1.256	1.695
est	-1.586	-1.504	-0.744	1.742	1.54	1.46
hauts basins	-1.217	-1.052	-0.498	1.673	1.139	1.25
nord	-1.425	-1.361	-0.784	1.441	1.189	1.349
plateau central	-1.512	-1.235	-0.531	1.517	1.257	1.373
sahel	-1.732	-1.631	-0.873	1.51	1.326	1.319
sud-ouest	-1.558	-1.196	-0.449	1.497	1.241	1.299
Total	-1.368	-1.257	-0.644	1.644	1.263	1.426

**Cameroon 1991**	**Mean**	**Mean**	**Mean**	**Sd**	**Sd**	**Sd**
	**who htz**	**who wtz**	**who wfl**	**who htz**	**who wtz**	**who wfl**
adam/nord/ext-nord	-2.02	-1.513	-0.464	1.767	1.404	1.222
centre/sud/est	-1.284	-0.677	0.097	1.538	1.232	1.092
nord-ouest/sud-ouest	-1.516	-0.506	0.503	1.407	1.126	1.073
ouest/littoral	-1.229	-0.263	0.606	1.534	1.154	1.079
yaoundé/douala	-0.742	-0.047	0.556	1.28	1.083	1.051
Total	-1.402	-0.698	0.167	1.611	1.361	1.207

Cameroon 1998	Mean	Mean	Mean	Sd	Sd	Sd
	who htz	who wtz	who wfl	who htz	who wtz	who wfl
central, south, & east	-1.133	-0.503	0.131	1.656	1.296	1.296
north/ extreme north/ ad	-1.578	-1.224	-0.478	1.732	1.444	1.402
northwest & southwest	-1.094	-0.318	0.422	2.114	1.39	1.708
west & littoral	-1.147	-0.161	0.541	1.716	1.322	1.387
Total	-1.286	-0.653	0.042	1.787	1.434	1.48

Cameroon 2011	Mean	Mean	Mean	Sd	Sd	Sd
	who htz	who wtz	who wfl	who htz	who wtz	who wfl
adamaoua	-1.568	-0.92	-0.065	1.589	1.275	1.312
centre	-0.682	-0.221	0.221	1.708	1.247	1.149
douala	-0.598	0.03	0.517	1.376	0.991	1.223
est	-1.392	-0.849	0.013	1.833	1.305	1.341
extrême-nord	-1.661	-1.344	-0.493	1.935	1.43	1.383
littoral	-0.99	-0.007	0.756	1.556	1.153	1.142
nord	-1.472	-1.225	-0.505	1.75	1.296	1.267
nord-ouest	-1.408	-0.286	0.697	1.701	1.221	1.257
ouest	-1.313	-0.176	0.785	1.491	1.081	1.092
sud	-1.187	-0.598	0.108	1.653	1.248	1.268
sud-ouest	-1.063	-0.411	0.262	1.517	1.195	1.129
yaoundé	-0.513	0.073	0.505	1.398	1.11	1.147
Total	-1.235	-0.613	0.139	1.707	1.339	1.331

Central African Rep 1994	Mean	Mean	Mean	Sd	Sd	Sd
	who htz	who wtz	who wfl	who htz	who wtz	who wfl
bangui	-1.239	-0.943	-0.295	1.586	1.246	1.341
rs i	-1.612	-1.121	-0.266	1.727	1.299	1.394
rs ii	-1.735	-1.139	-0.137	1.673	1.383	1.337
rs iii	-1.539	-1.071	-0.269	1.724	1.349	1.361
rs iv	-1.623	-1.174	-0.431	1.619	1.311	1.29
rs v	-1.519	-1.27	-0.524	1.917	1.396	1.395
Total	-1.534	-1.105	-0.307	1.707	1.33	1.357

Chad 1996	Mean	Mean	Mean	Sd	Sd	Sd
	who htz	who wtz	who wfl	who htz	who wtz	who wfl
b.e.t.	-1.701	-2.216	-1.543	1.766	1.537	1.604
batha	-1.55	-1.786	-1.282	1.77	1.3	1.262
biltine	-2.022	-1.935	-1.066	1.892	1.33	1.176
chari-baguirmi	-1.652	-1.652	-0.949	1.9	1.403	1.366
guéra	-1.599	-1.801	-1.218	2.069	1.48	1.438
kanem	-2.605	-2.445	-1.284	1.66	1.239	1.21
lac	-2.183	-2.156	-0.943	2.123	1.389	1.538
logone occidental	-1.739	-1.282	-0.382	2.138	1.49	1.405
logone oriental	-1.283	-1.174	-0.57	1.914	1.437	1.423
mayo-kebbi	-1.675	-1.106	-0.227	1.77	1.273	1.359
moyen chari	-1.247	-0.937	-0.293	1.949	1.469	1.472
n'djaména	-1.384	-1.153	-0.475	1.813	1.344	1.339
ouaddaï	-1.848	-1.775	-0.98	1.8	1.338	1.235
salamat	-1.712	-1.928	-1.304	1.982	1.475	1.429
tandjilé	-1.436	-1.116	-0.35	1.884	1.402	1.333
Total	-1.609	-1.429	-0.677	1.911	1.442	1.416

Chad 2004	Mean	Mean	Mean	Sd	Sd	Sd

	who htz	who wtz	who wfl	who htz	who wtz	who wfl
b. e. t.	-2.168	-2.084	-0.985	2.131	1.483	1.521
bar azoum	-2.011	-1.899	-0.972	2.088	1.638	1.57
centre est	-1.607	-1.649	-0.949	2.178	1.614	1.491
chari baguirmi	-1.677	-1.616	-0.793	2.049	1.428	1.325
logone occidental	-1.432	-0.967	-0.188	2.204	1.555	1.572
mayo kebbi	-1.331	-1.245	-0.663	2.027	1.384	1.554
moyen chari	-1.019	-0.861	-0.387	1.778	1.451	1.457
n'djaména	-1.069	-1.108	-0.631	2.026	1.501	1.617
ouaddai est	-2.158	-1.763	-0.612	2.005	1.394	1.489
Total	-1.538	-1.409	-0.667	2.096	1.549	1.545

Congo 2005	Mean	Mean	Mean	Sd	Sd	Sd
	who htz	who wtz	who wfl	who htz	who wtz	who wfl
brazzaville	-1.047	-0.426	0.21	1.896	1.199	1.482
nord	-1.247	-0.519	0.219	1.975	1.276	1.434
pointe noire	-0.823	-0.359	0.127	1.798	1.107	1.499
sud	-1.047	-0.828	-0.279	1.817	1.255	1.474
Total	-1.049	-0.565	0.036	1.875	1.233	1.488

Congo 2012	Mean	Mean	Mean	Sd	Sd	Sd
	who htz	who wtz	who wfl	who htz	who wtz	who wfl
bouenza	-0.843	-0.856	-0.529	1.62	1.085	1.203
brazzaville	-0.668	-0.402	-0.058	1.461	1.098	1.253
cuvette	-1.059	-0.622	-0.04	1.411	1.046	1.002
cuvette - ouest	-1.223	-0.933	-0.279	1.554	1.121	1.116
kouilou	-1.185	-1.019	-0.495	1.391	1.043	1.111
lekoumou	-1.602	-1.006	-0.122	1.562	1.186	1.263
likouala	-1.211	-0.728	-0.011	1.47	1.202	1.141
niari	-1.093	-0.693	-0.105	1.585	1.117	1.148
plateaux	-1.224	-1.022	-0.288	1.591	1.203	1.181
pointe-noire	-0.913	-0.645	-0.172	1.38	1.062	1.095
pool	-1.219	-0.819	-0.194	1.48	1.151	1.274
sangha	-1.555	-0.721	0.2	1.446	1.102	1.271
Total	-1.138	-0.792	-0.19	1.513	1.131	1.188

Congo DR 2007	Mean	Mean	Mean	Sd	Sd	Sd
	who htz	who wtz	who wfl	who htz	who wtz	who wfl
bandundu	-1.791	-1.214	-0.246	1.921	1.525	1.53
bas-congo	-1.7	-1.269	-0.293	2.027	1.418	1.643
equateur	-1.514	-1.052	-0.396	2.138	1.755	1.648
kasaï occident	-1.752	-1.446	-0.418	2.014	1.449	1.553
kasaï oriental	-1.693	-1.252	-0.309	2.094	1.576	1.65
katanga	-1.624	-0.93	0.085	2.189	1.447	1.617
kinshasa	-0.845	-0.803	-0.479	1.781	1.243	1.325
maniema	-1.384	-0.921	-0.002	2.318	1.445	1.784
nord-kivu	-1.963	-0.882	0.406	2.052	1.574	1.636
orientale	-1.54	-0.835	0.059	1.959	1.383	1.493
sud-kivu	-1.894	-1.089	0.012	2.168	1.654	1.693
Total	-1.599	-1.06	-0.146	2.082	1.516	1.618

Côte d'Ivoire 1994	Mean	Mean	Mean	Sd	Sd	Sd
	who htz	who wtz	who wfl	who htz	who wtz	who wfl
center	-1.029	-0.943	-0.505	1.447	1.249	1.216
center east	-1.333	-1.013	-0.254	1.492	1.343	1.417

center north	-1.091	-0.875	-0.287	1.64	1.354	1.27
center west	-1.22	-0.971	-0.408	1.782	1.436	1.367
north	-1.455	-1.443	-0.859	1.705	1.487	1.423
north east	-1.533	-1.148	-0.33	1.621	1.308	1.437
north west	-1.563	-1.181	-0.391	1.504	1.386	1.298
south	-1.07	-0.854	-0.368	1.497	1.218	1.243
south west	-1.356	-1.082	-0.422	1.537	1.274	1.153
west	-1.688	-1.142	-0.282	1.541	1.437	1.257
Total	-1.265	-1.011	-0.405	1.597	1.345	1.3

Côte d'Ivoire 1998	Mean	Mean	Mean	Sd	Sd	Sd
	who htz	who wtz	who wfl	who htz	who wtz	who wfl
capital (abidjan)	-0.659	-0.443	-0.091	1.346	1.136	1.298
countryside	-1.438	-1.048	-0.279	1.594	1.236	1.246
small city	-1.405	-0.734	0.102	1.632	1.268	1.502
Total	-1.214	-0.784	-0.109	1.579	1.244	1.353

Côte d'Ivoire 2011	Mean	Mean	Mean	Sd	Sd	Sd
	who htz	who wtz	who wfl	who htz	who wtz	who wfl
Centre	-1.316	-1.033	-0.321	1.589	1.248	1.196
Centre-Est	-1.146	-0.852	-0.277	1.355	1.076	1.065
Centre-Nord	-1.214	-0.726	-0.007	1.522	1.123	1.201
Centre-Ouest	-1.246	-0.852	-0.226	1.459	1.107	1.297
Nord	-1.513	-1.151	-0.346	1.626	1.151	1.205
Nord-Ouest	-1.402	-0.914	-0.139	1.549	1.153	1.199
Nord-est	-1.406	-1.18	-0.442	1.491	1.178	1.308
Ouest	-1.329	-0.798	0.05	1.596	1.237	1.188
Sud sans Abidjan	-1.155	-0.862	-0.215	1.736	1.315	1.406
Sud-ouest	-1.051	-0.641	-0.151	1.627	1.388	1.312
Ville d'Abidjan	-0.778	-0.654	-0.202	1.674	1.316	1.315
Total	-1.254	-0.881	-0.194	1.571	1.212	1.244

Gabon 2000	Mean	Mean	Mean	Sd	Sd	Sd
	who htz	who wtz	who wfl	who htz	who wtz	who wfl
east (haut-ogooué & ogooué-lolo)	-1.398	-0.733	0.08	1.576	1.176	1.228
libreville,port-gentil	-0.656	-0.261	0.171	1.674	1.279	1.284
north (ogooué-ivindo & woleu-ntem)	-1.558	-0.718	0.249	1.608	1.261	1.141
south (ngounié, nyanga)	-1.414	-0.809	-0.012	1.499	1.166	1.172
west (estuaire, moyen-ogooué & ogooué-maritime)	-0.991	-0.436	0.135	1.474	1.148	1.167
Total	-1.192	-0.587	0.122	1.61	1.228	1.209

Gabon 2012	Mean	Mean	Mean	Sd	Sd	Sd
	who htz	who wtz	who wfl	who htz	who wtz	who wfl
estuaire	-0.826	-0.273	0.245	1.409	1.092	1.168
haut-ogooué	-1.087	-0.413	0.139	1.568	1.324	1.463
libreville-port-gentil	-0.424	-0.016	0.272	1.411	1.215	1.278
moyen-ogooué	-1.243	-0.548	0.19	1.552	1.243	1.181
ngounié	-1.225	-0.633	0.025	1.484	1.102	1.179
nyanga	-0.971	-0.686	-0.145	1.509	1.237	1.125
ogooué maritime	-1.043	-0.311	0.268	1.809	1.32	1.495
ogooué-ivindo	-1.383	-0.671	0.167	1.715	1.212	1.321
ogooué-lolo	-1.038	-0.491	0.108	1.837	1.314	1.24
woleu-ntem	-0.678	-0.024	0.396	1.456	1.259	1.339
Total	-1.004	-0.421	0.16	1.605	1.251	1.29

Ghana 1993	Mean	Mean	Mean	Sd	Sd	Sd
	who htz	who wtz	who wfl	who htz	who wtz	who wfl
ashanti	-1.318	-1.11	-0.433	1.645	1.319	1.374
brong-ahafo	-1.244	-1.335	-0.827	1.681	1.386	1.444
central	-1.135	-1.032	-0.508	1.695	1.35	1.518
eastern	-1.302	-0.58	0.112	1.617	1.505	1.487
greater accra	-0.896	-0.806	-0.387	1.431	1.236	1.096
northern	-1.646	-1.744	-0.863	2.025	1.73	1.613
upper east	-1.312	-1.532	-1.067	1.592	1.485	1.279
upper west	-1.674	-1.501	-0.959	1.426	1.163	1.135
volta	-1.11	-0.99	-0.594	1.572	1.42	1.325
western	-1.594	-1.342	-0.622	1.472	1.354	1.332
Total	-1.3	-1.158	-0.563	1.656	1.45	1.422

Ghana 1998	Mean	Mean	Mean	Sd	Sd	Sd
	who htz	who wtz	who wfl	who htz	who wtz	who wfl
ashanti region	-1.455	-1.095	-0.39	1.521	1.255	1.301
brong ahafo region	-1.166	-1.088	-0.479	1.342	1.154	1.239
central region	-1.485	-1.129	-0.427	1.6	1.298	1.261
eastern region	-1.113	-0.965	-0.43	1.59	1.125	1.166
greater accra region	-0.727	-0.566	-0.247	1.443	1.105	1.215
northern region	-1.781	-1.46	-0.587	1.725	1.308	1.263
upper east region	-1.509	-1.33	-0.633	1.806	1.26	1.215
upper west region	-1.601	-1.262	-0.436	1.816	1.381	1.267
volta region	-1.262	-1.206	-0.595	1.486	1.135	1.338
western region	-1.333	-1.063	-0.468	1.672	1.148	1.417
Total	-1.355	-1.121	-0.471	1.635	1.239	1.273

Ghana 2003	Mean	Mean	Mean	Sd	Sd	Sd
	who htz	who wtz	who wfl	who htz	who wtz	who wfl
ashanti	-1.447	-0.979	-0.239	1.549	1.14	1.286
brong ahafo	-1.453	-0.975	-0.22	1.547	1.216	1.207
central	-1.522	-0.816	0.131	1.446	1.113	1.231
eastern	-1.312	-0.863	-0.125	1.491	1.182	1.298
greater accra	-0.715	-0.554	-0.205	1.62	1.235	1.451
northern	-2.122	-1.389	-0.138	1.525	1.256	1.363
upper east	-1.307	-1.465	-0.764	1.576	1.222	1.38
upper west	-1.457	-0.866	-0.187	1.659	1.586	1.691
volta	-1.157	-1.188	-0.616	1.535	1.459	1.531
western	-1.329	-0.826	-0.184	1.519	1.177	1.359
Total	-1.443	-1.015	-0.233	1.588	1.284	1.388

Ghana 2008	Mean	Mean	Mean	Sd	Sd	Sd
	who htz	who wtz	who wfl	who htz	who wtz	who wfl
ashanti	-0.958	-0.698	-0.321	1.701	1.255	1.332
brong ahafo	-1.036	-0.821	-0.299	1.47	0.992	1.079
central	-1.316	-0.898	-0.177	1.817	1.583	1.664
eastern	-1.397	-0.587	0.26	1.721	1.382	1.583
greater accra	-0.683	-0.258	-0.127	1.548	1.446	1.332
northern	-1.137	-1.012	-0.548	1.843	1.274	1.366
upper east	-1.346	-1.092	-0.536	1.936	1.45	1.431
upper west	-0.984	-0.987	-0.686	1.565	1.184	1.442
volta	-1.028	-0.711	-0.226	1.595	1.268	1.421
western	-1.018	-0.631	-0.101	1.765	1.313	1.42
Total	-1.073	-0.774	-0.302	1.71	1.325	1.416

Guinea 1999	Mean	Mean	Mean	Sd	Sd	Sd
	who htz	who wtz	who wfl	who htz	who wtz	who wfl
central guinea	-1.292	-1.056	-0.397	1.915	1.42	1.508
conakry	-0.836	-0.863	-0.44	1.593	1.396	1.363
forest guinea	-1.425	-0.875	-0.032	1.939	1.471	1.352
lower guinea	-1.227	-0.924	-0.289	1.708	1.368	1.364
upper guinea	-1.165	-1.045	-0.45	2.054	1.583	1.469
Total	-1.222	-0.95	-0.292	1.875	1.457	1.417

Guinea 2005	Mean	Mean	Mean	Sd	Sd	Sd
	who htz	who wtz	who wfl	who htz	who wtz	who wfl
boké	-1.054	-0.728	-0.163	1.787	1.27	1.325
conakry	-0.911	-0.572	-0.184	1.908	1.604	1.542
faranah	-1.377	-1.246	-0.574	1.759	1.225	1.422
kankan	-1.734	-1.275	-0.322	1.808	1.282	1.506
kindia	-1.246	-0.955	-0.257	1.968	1.517	1.383
labé	-1.363	-0.922	-0.218	2.063	1.467	1.679
mamou	-1.428	-0.821	-0.006	1.745	1.282	1.352
n'zérékoré	-1.807	-1.196	-0.215	1.784	1.423	1.526
Total	-1.415	-1.015	-0.261	1.865	1.392	1.471

Guinea 2012	Mean	Mean	Mean	Sd	Sd	Sd
	who htz	who wtz	who wfl	who htz	who wtz	who wfl
Bok?	-0.92	-0.653	-0.182	1.884	1.399	1.406
Conakry	-0.272	-0.488	-0.448	1.968	1.328	1.312
Faranah	-1.188	-0.844	-0.228	1.706	1.334	1.285
Kankan	-1.224	-1.268	-0.74	1.825	1.453	1.534
Kindia	-0.9	-0.586	-0.113	1.984	1.453	1.437
Lab?	-1.037	-0.868	-0.366	2.059	1.338	1.381
Mamou	-1.412	-1.062	-0.402	1.85	1.249	1.391
N'Z?r?kor?	-1.269	-0.924	-0.262	1.691	1.223	1.332
Total	-1.062	-0.871	-0.36	1.885	1.376	1.407

Liberia 2007	Mean	Mean	Mean	Sd	Sd	Sd
	who htz	who wtz	who wfl	who htz	who wtz	who wfl
monrovia	-1.119	-0.835	-0.335	1.782	1.374	1.464
north central	-1.578	-0.976	-0.029	1.818	1.362	1.274
north western	-1.491	-0.897	-0.006	1.795	1.286	1.194
south central	-1.378	-0.888	-0.092	1.657	1.209	1.243
south eastern a	-1.6	-0.979	-0.118	1.856	1.398	1.285
south eastern b	-1.663	-1.112	-0.125	1.98	1.428	1.496
Total	-1.478	-0.955	-0.118	1.831	1.352	1.342

Mali 1995	Mean	Mean	Mean	Sd	Sd	Sd
	who htz	who wtz	who wfl	who htz	who wtz	who wfl
bamako	-0.73	-1.416	-1.216	1.649	1.606	1.631
gao	-1.387	-1.502	-0.96	1.47	1.458	1.532
kayes	-1.521	-1.577	-0.98	1.8	1.394	1.327
koulikoro	-1.433	-1.674	-1.105	1.78	1.48	1.563
mopti	-1.031	-1.754	-1.414	2.18	1.743	1.63
sikasso	-1.586	-1.775	-1.119	1.796	1.478	1.536
ségou	-1.486	-1.671	-1.059	1.982	1.569	1.652
timbuktu	-1.367	-2.01	-1.488	1.688	1.646	1.522
Total	-1.363	-1.67	-1.137	1.856	1.54	1.558

Mali 2001	Mean	Mean	Mean	Sd	Sd	Sd
	who htz	who wtz	who wfl	who htz	who wtz	who wfl
bamako	-0.722	-0.625	-0.321	1.598	1.247	1.232
gao	-1.269	-1.426	-0.975	1.9	1.484	1.329
kayes	-1.694	-1.361	-0.509	1.872	1.498	1.392
kidal	-1.238	-1.088	-0.503	1.473	1.305	1.168
koulikoro	-1.647	-1.385	-0.532	1.741	1.368	1.294
mopti	-1.718	-1.493	-0.575	1.869	1.471	1.337
segou	-1.535	-1.381	-0.63	2.16	1.542	1.603
sikasso	-2.02	-1.534	-0.463	1.709	1.368	1.291
tombouctou	-1.612	-1.39	-0.576	2.286	1.574	1.599
Total	-1.603	-1.341	-0.53	1.881	1.451	1.372

Mali 2006	Mean	Mean	Mean	Sd	Sd	Sd
	who htz	who wtz	who wfl	who htz	who wtz	who wfl
bamako	-0.739	-0.825	-0.578	1.883	1.423	1.499
gao	-1.553	-1.283	-0.634	2.185	1.834	1.712
kayes	-1.12	-1.081	-0.598	2.003	1.574	1.618
kidal	-0.803	-1.05	-0.805	2.178	1.6	1.816
koulikoro	-1.448	-1.326	-0.621	1.862	1.388	1.581
mopti	-1.471	-1.304	-0.541	2.19	1.58	1.558
segou	-1.464	-1.304	-0.566	1.856	1.404	1.477
sikasso	-1.667	-1.391	-0.551	1.881	1.474	1.528
tombouctou	-1.724	-1.36	-0.466	2.1	1.545	1.72
Total	-1.396	-1.244	-0.575	2.007	1.524	1.577

Niger 1992	Mean	Mean	Mean	Sd	Sd	Sd
	who htz	who wtz	who wfl	who htz	who wtz	who wfl
agadez	-1.489	-1.506	-0.679	1.775	1.343	1.534
diffa	-1.937	-1.549	-0.697	1.936	1.475	1.675
dosso	-1.623	-1.519	-0.826	1.654	1.28	1.167
maradi	-2.173	-2.048	-1.027	1.765	1.488	1.408
niamey	-1.075	-1.127	-0.708	1.547	1.223	1.318
tahoua	-1.838	-1.645	-0.74	1.797	1.411	1.335
tillabéri	-1.614	-1.602	-0.906	1.747	1.309	1.25
zinder	-1.907	-1.71	-0.821	2.017	1.526	1.498
Total	-1.699	-1.604	-0.826	1.801	1.411	1.365

Niger 1998	Mean	Mean	Mean	Sd	Sd	Sd
	who htz	who wtz	who wfl	who htz	who wtz	who wfl
dosso	-1.736	-1.8	-1.102	1.517	1.257	1.217
maradi	-2.164	-2.038	-1.11	1.645	1.418	1.371
niamey	-1.224	-1.238	-0.759	1.594	1.305	1.361
tahoua/agadez	-1.659	-1.789	-1.153	1.628	1.394	1.275
tillaberi	-1.626	-1.771	-1.201	1.735	1.379	1.312
zinda/diffa	-1.779	-1.903	-1.2	2	1.437	1.458
Total	-1.763	-1.812	-1.114	1.72	1.393	1.341

Niger 2006	Mean	Mean	Mean	Sd	Sd	Sd
	who htz	who wtz	who wfl	who htz	who wtz	who wfl
agadez	-1.459	-1.192	-0.403	1.899	1.313	1.425
diffa	-1.79	-1.745	-0.9	1.936	1.358	1.324
dosso	-1.87	-1.492	-0.559	1.596	1.312	1.296
maradi	-2.524	-1.887	-0.512	1.773	1.415	1.408

niamey	-0.984	-0.767	-0.326	1.727	1.313	1.312
tahoua	-1.898	-1.557	-0.618	1.904	1.366	1.403
tillabéri	-2.029	-1.495	-0.457	1.609	1.362	1.399
zinder	-2.377	-1.86	-0.476	1.954	1.371	1.595
Total	-1.923	-1.529	-0.531	1.837	1.393	1.4

**Niger 2012**	**Mean**	**Mean**	**Mean**	**Sd**	**Sd**	**Sd**
	**who htz**	**who wtz**	**who wfl**	**who htz**	**who wtz**	**who wfl**
Agadez	-1.701	-1.097	-0.175	1.91	1.407	1.841
Diffa	-2.133	-2.397	-1.31	1.821	1.437	1.859
Dosso	-1.516	-1.439	-0.806	1.483	1.257	1.206
Maradi	-1.842	-1.733	-0.766	1.991	1.367	1.597
Niamey	-0.916	-0.893	-0.397	1.622	1.248	1.341
Tahoua	-1.382	-1.699	-1.026	1.809	1.428	1.517
Tillaberi	-1.57	-1.503	-0.856	1.42	1.154	1.152
Zinder	-1.874	-1.743	-0.889	1.855	1.403	1.39
Total	-1.626	-1.61	-0.822	1.779	1.382	1.485

**Nigeria 1990**	**Mean**	**Mean**	**Mean**	**Sd**	**Sd**	**Sd**
	**who htz**	**who wtz**	**who wfl**	**who htz**	**who wtz**	**who wfl**
northeast	-2.21	-1.737	-0.472	2.012	1.597	1.491
northwest	-2.039	-1.548	-0.43	1.957	1.486	1.479
southeast	-1.632	-1.235	-0.344	1.722	1.333	1.202
southwest	-1.514	-1.088	-0.253	1.626	1.303	1.188
Total	-1.824	-1.384	-0.369	1.844	1.449	1.338

**Nigeria 2003**	**Mean**	**Mean**	**Mean**	**Sd**	**Sd**	**Sd**
	**who htz**	**who wtz**	**who wfl**	**who htz**	**who wtz**	**who wfl**
north central	-1.262	-0.975	-0.258	1.789	1.337	1.341
north east	-1.769	-1.303	-0.353	1.932	1.382	1.458
north west	-2.288	-1.675	-0.25	2.057	1.58	1.772
south east	-0.935	-0.532	0.061	1.91	1.472	1.375
south south	-0.907	-0.763	-0.399	1.834	1.494	1.508
south west	-1.173	-0.924	-0.289	1.689	1.384	1.409
Total	-1.597	-1.192	-0.265	1.974	1.503	1.532

**Nigeria 2008**	**Mean**	**Mean**	**Mean**	**Sd**	**Sd**	**Sd**
	**who htz**	**who wtz**	**who wfl**	**who htz**	**who wtz**	**who wfl**
north central	-1.623	-0.925	0.176	2.158	1.67	1.768
north east	-1.658	-1.492	-0.348	2.329	1.685	2.013
north west	-1.805	-1.622	-0.429	2.394	1.824	2.006
south east	-0.828	-0.492	-0.032	1.967	1.485	1.583
south south	-1.173	-0.516	0.156	1.964	1.493	1.617
south west	-1.145	-0.673	-0.031	1.985	1.4	1.614
Total	-1.493	-1.121	-0.147	2.225	1.711	1.857

**Sao Tome et Principe 2008**	**Mean**	**Mean**	**Mean**	**Sd**	**Sd**	**Sd**
	**who htz**	**who wtz**	**who wfl**	**who htz**	**who wtz**	**who wfl**
região centro	-1.268	-0.83	-0.052	1.786	1.323	1.705
região do principe	-0.182	-0.408	-0.469	1.721	1.485	1.519
região norte	-1.28	-0.525	0.262	1.861	1.463	1.794
região sul	-1.166	-0.87	-0.228	2.097	1.351	1.999
Total	-1.056	-0.678	-0.082	1.923	1.413	1.801

Senegal 1992	Mean<br>who htz	Mean<br>who wtz	Mean<br>who wfl	Sd<br>who htz	Sd<br>who wtz	Sd<br>who wfl
central	-1.559	-1.177	-0.34	1.695	1.375	1.366
north east	-1.529	-1.346	-0.556	1.671	1.411	1.406
south	-1.457	-1.146	-0.399	1.673	1.408	1.347
west	-1.084	-0.759	-0.204	1.493	1.235	1.241
Total	-1.384	-1.063	-0.339	1.637	1.359	1.336

Senegal 2005	Mean<br>who htz	Mean<br>who wtz	Mean<br>who wfl	Sd<br>who htz	Sd<br>who wtz	Sd<br>who wfl
dakar	-0.491	-0.256	0.003	1.525	1.225	1.157
diourbel	-0.749	-0.754	-0.458	1.438	1.139	1.212
fatick	-0.917	-0.85	-0.46	1.336	0.991	1.139
kaolack	-0.802	-0.761	-0.431	1.454	1.13	1.142
kolda	-1.398	-1.176	-0.55	1.576	1.327	1.207
louga	-0.915	-0.933	-0.61	1.487	1.136	1.289
matam	-0.9	-1.06	-0.748	1.57	1.248	1.299
saint-louis	-1.095	-1.105	-0.631	1.545	1.202	1.199
tambacounda	-1.251	-1.107	-0.46	1.604	1.183	1.393
thiès	-0.611	-0.615	-0.39	1.285	1.141	1.155
zuguinchor	-0.796	-0.578	-0.224	1.557	1.121	1.171
Total	-0.915	-0.852	-0.463	1.505	1.197	1.228

Senegal 2010	Mean<br>who htz	Mean<br>who wtz	Mean<br>who wfl	Sd<br>who htz	Sd<br>who wtz	Sd<br>who wfl
dakar	-0.803	-0.703	-0.371	1.563	1.212	1.201
diourbel	-1.303	-1.055	-0.475	1.577	1.295	1.242
fatick	-0.984	-0.798	-0.351	1.568	1.174	1.214
kaffrine	-1.572	-1.249	-0.423	1.675	1.257	1.38
kaolack	-1.204	-1.023	-0.557	1.449	1.208	1.214
kedougou	-1.484	-1.132	-0.347	1.71	1.321	1.127
kolda	-1.622	-1.208	-0.368	1.56	1.304	1.169
louga	-1.278	-1.291	-0.737	1.728	1.377	1.452
matam	-1.23	-1.3	-0.742	1.675	1.316	1.403
saint-louis	-0.948	-1.108	-0.835	1.609	1.484	1.199
sedhiou	-1.7	-1.172	-0.234	1.581	1.258	1.267
tambacounda	-1.053	-1.167	-0.783	1.919	1.294	1.257
thiès	-1.04	-0.949	-0.527	1.88	1.303	1.389
ziguinchor	-1.178	-0.869	-0.23	1.348	1.193	1.169
Total	-1.247	-1.076	-0.505	1.655	1.294	1.286

Sierra Leone 2008	Mean<br>who htz	Mean<br>who wtz	Mean<br>who wfl	Sd<br>who htz	Sd<br>who wtz	Sd<br>who wfl
eastern	-1.197	-0.415	0.222	2.077	1.802	1.824
northern	-1.361	-0.939	-0.201	2.105	1.553	1.512
southern	-1.397	-0.759	-0.142	2.525	1.842	2.021
western	-0.852	-0.567	-0.144	2.018	1.507	1.689
Total	-1.249	-0.705	-0.071	2.199	1.699	1.758

Togo 1998	Mean<br>who htz	Mean<br>who wtz	Mean<br>who wfl	Sd<br>who htz	Sd<br>who wtz	Sd<br>who wfl
centrale	-1.18	-1.018	-0.464	1.7	1.287	1.364
kara	-1.052	-1.163	-0.783	1.744	1.28	1.329
lomé	-0.759	-0.712	-0.374	1.475	1.226	1.353
marities	-1.164	-1.101	-0.574	1.554	1.301	1.37

plateaux	-1.261	-1.065	-0.445	1.587	1.385	1.344
savanes	-1.535	-1.51	-0.91	1.758	1.359	1.374
Total	-1.235	-1.167	-0.636	1.677	1.342	1.371

Mean and standard deviation (SD) for height-for-age (HAZ), weight-for-age (WTZ), weight-for-height (WHZ), and mid-upper arm circumference, by region NNS

Benin 2008	Mean	Mean	Mean	Mean	Sd	Sd	Sd	Sd
	who htz	who wtz	who wfl	muac	who htz	who wtz	who wfl	muac
alibori	-1.639	-0.985	-0.096	14.523	1.201	0.978	1.092	1.271
atacora	-1.723	-1.266	-0.376	14.583	1.408	1.095	1.157	1.326
atlantique	-1.553	-1.113	-0.33	15.282	1.402	1.117	1.028	1.275
borgou	-1.412	-0.906	-0.127	14.881	1.52	1.124	1.164	1.337
collines	-1.412	-0.829	-0.052	15.263	1.378	1.069	1.14	1.24
couffo	-1.753	-1.058	-0.095	14.819	1.358	1.062	1.074	1.279
donga	-1.462	-0.899	-0.099	14.992	1.318	1.213	1.171	1.389
littoral	-1.106	-0.587	0.032	15.331	1.236	1.103	1.152	1.432
mono	-1.558	-1.206	-0.411	15.359	1.31	1.057	0.953	1.229
oueme	-1.374	-1.068	-0.393	15.085	1.231	1.023	1.031	1.217
plateau	-1.628	-1.279	-0.481	15.327	1.342	1.126	0.951	1.216
zou	-1.586	-1.076	-0.204	15.061	1.401	1.092	0.955	1.398
Total	-1.538	-1.03	-0.215	14.997	1.356	1.099	1.09	1.33

Burkina Faso 2012	Mean	Mean	Mean	Mean	Sd	Sd	Sd	Sd
	who htz	who wtz	who wfl	muac	who htz	who wtz	who wfl	muac
bales	-1.089	-1.05	-0.616	14.315	1.156	0.986	1.039	1.128
bam	-1.42	-1.379	-0.831	14.231	1.291	1.13	1.146	1.283
banwa	-1.576	-1.354	-0.669	14.442	1.219	1.065	1.087	1.272
banwa ganzourgou	-1.488	-1.31	-0.668	14.366	1.158	0.992	0.976	1.21
bazega	-1.478	-1.334	-0.705	14.579	1.155	1.043	1.039	1.123
boulgou	-1.43	-1.271	-0.63	14.411	1.108	1.014	1.087	1.195
cascades	-1.62	-1.123	-0.26	14.749	1.233	1.043	1.087	1.224
centre ouest	-1.466	-1.374	-0.792	14.468	1.188	1.057	1.03	1.153
est	-1.813	-1.585	-0.753	14.205	1.193	1.041	1.04	1.199
houet	-1.408	-1.096	-0.422	14.683	1.241	1.098	1.116	1.208
kadiogo	-0.959	-1.013	-0.669	14.866	1.21	1.054	1.067	1.353
kenedougou	-1.655	-1.215	-0.395	14.453	1.212	1.097	1.066	1.24
kossi	-1.665	-1.458	-0.705	14.218	1.27	1.113	1.136	1.295
koulpelogo	-1.763	-1.502	-0.696	14.349	1.139	1.01	1.036	1.139
kouritenga	-1.545	-1.397	-0.752	14.293	1.224	1.043	1.004	1.129
kourweogo	-1.675	-1.598	-0.915	14.247	1.217	1.085	1.051	1.232
mouhoun	-1.402	-1.238	-0.639	14.49	1.172	1.032	1.052	1.322
nahouri	-1.454	-1.235	-0.57	14.705	1.133	0.939	0.986	1.063
namentenga	-1.618	-1.444	-0.727	14.202	1.195	1.084	1.114	1.227
nayala	-1.449	-1.392	-0.791	14.139	1.239	1.028	1.097	1.193
nord	-1.364	-1.361	-0.846	14.509	1.26	1.053	1.012	1.156
oubritenga	-1.477	-1.414	-0.833	14.525	1.157	1.075	1.043	1.198
sahel	-1.614	-1.32	-0.573	14.098	1.366	0.987	1.138	1.157
sanmentenga	-1.495	-1.446	-0.866	14.279	1.172	1.042	1.037	1.168
sourou	-1.434	-1.249	-0.619	14.466	1.153	1.043	1.032	1.14
sud ouest	-1.519	-1.248	-0.532	14.254	1.23	1.085	1.102	1.182
tuy	-1.461	-1.203	-0.527	14.421	1.113	1.04	1.079	1.236
zoundweogo	-1.507	-1.324	-0.652	14.824	1.173	1	1.024	1.212
Total	-1.509	-1.334	-0.675	14.396	1.213	1.055	1.072	1.216

Cameroon 2011	Mean	Mean	Mean	Mean	Sd	Sd	Sd	Sd
	who htz	who wtz	who wfl	muac	who htz	who wtz	who wfl	muac
extrême-nord	-1.645	-1.472	-0.764	14.276	1.457	1.181	1.055	1.494
nord	-1.805	-1.266	-0.369	14.285	1.589	1.312	1.29	1.454
Total	-1.726	-1.367	-0.563	14.281	1.527	1.253	1.197	1.473

Central African Rep 2012	Mean	Mean	Mean	Mean	Sd	Sd	Sd	Sd

	who htz	who wtz	who wfl	muac	who htz	who wtz	who wfl	muac
bamingui bangoran	-1.082	-0.992	-0.551	14.655	1.452	1.226	1.126	1.327
bangui	-1.098	-1.021	-0.556	14.278	1.508	1.198	1.075	1.108
basse kotto	-1.847	-1.411	-0.43	14.165	1.583	1.249	1.178	1.249
haut kotto	-1.18	-0.694	-0.017	14.656	1.292	1.08	1.098	1.265
haut mbomou	-1.304	-0.902	-0.203	13.96	1.471	1.169	1.157	1.258
kemo	-1.661	-1.349	-0.544	14.019	1.27	1.203	1.225	1.397
lobaye	-1.991	-1.512	-0.486	14.12	1.384	1.174	1.079	1.216
mambere kadei	-2.145	-1.558	-0.383	14.378	1.488	1.281	1.123	1.309
mbomou	-1.538	-1.043	-0.213	14.28	1.472	1.208	1.213	1.319
nana grebizi	-1.595	-1.263	-0.505	13.894	1.329	1.158	1.143	1.304
nana mambere	-1.818	-1.309	-0.328	14.318	1.489	1.286	1.165	1.322
ombella mpoko	-1.479	-1.199	-0.463	14.403	1.418	1.211	1.109	1.264
ouaka	-1.441	-1.193	-0.485	14.243	1.537	1.253	1.159	1.273
ouham	-1.67	-1.135	-0.212	14.544	1.579	1.225	1.187	1.268
ouham pende	-1.601	-1.291	-0.485	14.09	1.492	1.259	1.19	1.214
sangha mbarere	-1.886	-1.392	-0.401	14.357	1.383	1.209	1.121	1.31
vakaga	-1.081	-0.979	-0.494	14.412	1.514	1.231	1.079	1.233
Total	-1.588	-1.207	-0.391	14.278	1.482	1.233	1.153	1.295

Chad June 2012	Mean	Mean	Mean	Mean	Sd	Sd	Sd	Sd
	who htz	who wtz	who wfl	muac	who htz	who wtz	who wfl	muac
Barh El Ghazal	-1.167	-1.524	-1.26	14.193	1.282	1.071	1.071	1.321
Batha	-1.178	-1.589	-1.335	13.79	1.425	1.07	1.009	1.232
Guéra	-1.343	-1.434	-0.921	13.817	1.278	1.104	1.042	1.382
Hadjer Lamis	-1.298	-1.502	-1.1	14.132	1.315	1.17	1.112	1.421
Kanem	-2.064	-1.97	-1.162	13.723	1.349	1.1	1.031	1.273
Lac	-1.93	-1.773	-0.926	13.78	1.189	1.001	1.103	1.364
N’Djamena	-0.66	-0.978	-0.849	14.559	1.519	1.201	1.106	1.382
Ouaddai	-1.508	-1.583	-1.05	14.166	1.465	1.096	1.037	1.161
Salamat	-1.465	-1.604	-1.134	14.012	1.572	1.217	1.136	1.309
Sila	-1.405	-1.421	-0.896	13.989	1.515	1.162	1.096	1.318
Wadi Fira	-1.532	-1.71	-1.227	14.056	1.322	1.034	1.044	1.198
Total	-1.385	-1.538	-1.079	14.029	1.437	1.141	1.083	1.329

Chad (7 regions) Dec/Jan 2012-13	Mean	Mean	Mean	Mean	Sd	Sd	Sd	Sd
	who htz	who wtz	who wfl	muac	who htz	who wtz	who wfl	muac
Logone Occidental	-1.542	-0.934	-0.1	14.669	1.512	1.241	1.193	1.447
Logone Oriental	-1.465	-0.814	-0.021	14.604	1.409	1.183	1.169	1.468
Mandoul	-1.108	-0.499	0.182	14.634	1.364	1.114	1.114	1.238
Mayo-Kebbi Est	-1.652	-1.058	-0.155	14.543	1.361	1.165	1.093	1.258
Mayo-Kebbi Ouest	-0.923	-0.545	-0.005	14.701	1.532	1.215	1.152	1.303
Moyen-Chari	-1.777	-1.105	-0.148	14.525	1.297	1.123	1.125	1.392
Tandjilé	-1.313	-0.694	0.06	14.594	1.363	1.164	1.116	1.281
Total	-1.412	-0.816	-0.028	14.606	1.427	1.192	1.141	1.342

The Gambia 2012	Mean	Mean	Mean	Mean	Sd	Sd	Sd	Sd
	who htz	who wtz	who wfl	muac	who htz	who wtz	who wfl	muac
banjul	-0.812	-0.927	-0.671	15.062	1.15	1.07	1.09	1.215
basse	-1.277	-1.223	-0.721	14.501	1.158	1.046	1.07	1.21
brikama	-0.999	-0.967	-0.582	15.095	1.197	1.064	1.03	1.151
janjanburay	-1.32	-1.25	-0.738	14.417	1.278	1.136	1.103	1.205
kanifing	-0.77	-0.867	-0.624	14.984	1.201	1.103	1.098	1.243
kerewan	-1.292	-1.202	-0.68	14.786	1.125	1.043	1.075	1.238
kuntaur	-1.392	-1.385	-0.854	14.325	1.267	1.054	1.045	1.247
mansakonko	-1.288	-1.147	-0.614	14.789	1.164	1.04	1.079	1.181
Total	-1.179	-1.148	-0.695	14.702	1.213	1.08	1.076	1.244

Guinea-Bissau 2008	Mean	Mean	Mean	Mean	Sd	Sd	Sd	Sd

	who htz	who wtz	who wfl	muac	who htz	who wtz	who wfl	muac
Capitale	-0.858	-0.507	-0.055	15.274	1.306	1.144	1.077	1.404
Est (Bafata e Gabu)	-1.488	-1.209	-0.532	14.432	1.282	1.093	1.045	1.386
Nord (Biombo, Cacheu e Oio)	-1.337	-0.986	-0.343	14.574	1.443	1.275	1.085	1.5
Sud (Bolama, Quinara e Tombali)	-1.364	-0.87	-0.176	14.709	1.269	1.132	1.021	1.33
Total	-1.312	-0.947	-0.312	14.679	1.345	1.186	1.069	1.435

Guinée Conakay 2012	Mean	Mean	Mean	Mean	Sd	Sd	Sd	Sd
	who htz	who wtz	who wfl	muac	who htz	who wtz	who wfl	muac
boke nord	-1.716	-1.02	-0.033	14.364	1.356	1.066	1.218	1.185
boke sud	-1.182	-0.773	-0.113	15.212	1.393	1.198	1.111	1.366
conakry	-0.947	-0.786	-0.354	15.215	1.372	1.131	1.169	1.357
faranah	-1.713	-0.894	0.117	14.912	1.331	1.101	1.175	1.27
kankan	-1.654	-1.042	-0.135	14.526	1.316	1.109	1.108	1.295
kindia	-1.506	-0.963	-0.138	14.861	1.321	1.151	1.193	1.422
labe	-1.679	-1.226	-0.364	14.461	1.41	1.191	1.159	1.293
mamou	-1.662	-1.142	-0.272	14.568	1.269	1.125	1.089	1.362
nzerekore	-1.656	-0.836	0.188	14.965	1.204	1.073	1.079	1.35
Total	-1.537	-0.978	-0.131	14.769	1.35	1.137	1.157	1.358

Liberia 2010	Mean	Mean	Mean	Mean	Sd	Sd	Sd	Sd
	who htz	who wtz	who wfl	muac	who htz	who wtz	who wfl	muac
bomi	-1.76	-0.932	0.101	14.902	1.54	1.173	1.091	1.312
bong	-1.509	-0.902	-0.112	15.061	1.475	1.187	1.085	1.361
gbarpolu	-1.658	-0.884	0.098	15.032	1.405	1.125	1.185	1.273
grand bassa	-1.899	-1.02	0.097	14.786	1.297	1.092	1.096	1.324
grand cape mount	-1.413	-0.846	-0.075	15.132	1.644	1.321	1.222	1.412
grand gedeh	-1.748	-1.053	-0.078	14.972	1.347	1.115	1.13	1.347
grand kru	-1.687	-0.9	0.078	14.797	1.489	1.091	1.025	1.25
lofa	-1.581	-0.912	-0.069	15.225	1.438	1.133	1.078	1.368
margibi	-2.202	-1.01	0.342	15.064	1.226	1.019	1.117	1.425
maryland	-1.64	-0.889	0.039	14.75	1.626	1.301	1.26	1.383
montserrado	-1.31	-0.872	-0.197	14.999	1.468	1.2	1.055	1.372
nimba	-1.819	-1.129	-0.151	15.052	1.13	0.939	1.016	1.079
river gee	-1.407	-0.608	0.211	15.055	1.628	1.206	1.089	1.395
rivercess	-1.711	-1.023	-0.068	14.725	1.479	1.188	1.116	1.368
rural montserrado	-1.573	-0.78	0.163	15.131	1.579	1.171	1.069	1.256
sinoe	-1.873	-1.087	-0.067	14.822	1.231	1.072	1.184	1.414
Total	-1.672	-0.931	0.013	14.972	1.457	1.155	1.123	1.344

Liberia 2011	Mean	Mean	Mean	Mean	Sd	Sd	Sd	Sd
	who htz	who wtz	who wfl	muac	who htz	who wtz	who wfl	muac
North Central	-1.505	-1.011	-0.239	14.469	1.431	1.208	1.087	1.276
North Western	-1.638	-1.282	-0.55	14.364	1.38	1.235	1.191	1.265
South Central	-1.466	-1.202	-0.587	14.1	1.449	1.31	1.272	1.404
South Eastern	-1.622	-1.102	-0.31	14.523	1.396	1.355	1.191	1.389
Total	-1.496	-1.165	-0.5	14.217	1.437	1.293	1.237	1.382

Mali 2011	Mean	Mean	Mean	Mean	Sd	Sd	Sd	Sd
	who htz	who wtz	who wfl	muac	who htz	who wtz	who wfl	muac
bamako	-0.52	-0.749	-0.654	14.71	1.447	1.154	1.068	1.365
gao	-0.913	-1.082	-0.824	14.159	1.374	1.133	1.131	1.217
kayes	-1.004	-1.129	-0.799	14.261	1.296	1.089	1.126	1.317
kidal	-0.545	-0.508	-0.276	14.683	1.269	0.941	1.044	1.277
koulikoro	-1.216	-1.204	-0.746	14.364	1.368	1.134	1.111	1.289
mopti	-1.402	-1.213	-0.602	14.36	1.395	1.097	1.112	1.269
segou	-1.148	-1.032	-0.55	14.401	1.318	0.953	1.027	1.231
sikasso	-1.648	-1.283	-0.471	14.639	1.408	1.116	1.071	1.373
tombouctou	-1.156	-1.359	-1.017	14.186	1.411	1.115	1.071	1.373

Total	-1.098	-1.1	-0.685	14.41	1.422	1.122	1.104	1.322

Mauritania 2006	Mean	Mean	Mean	Mean	Sd	Sd	Sd	Sd
	who htz	who wtz	who wfl	muac	who htz	who wtz	who wfl	muac
Centre	-1.334	-1.108	-0.582		1.662	1.311	1.114	
Fleuve	-1.168	-1.082	-0.639		1.664	1.372	1.171	
Nord	-1.376	-1.129	-0.477		1.613	1.31	1.124	
Nouakchott	-1.379	-1.091	-0.413		1.745	1.453	1.186	
SudEst	-1.402	-1.031	-0.411		1.691	1.389	1.152	
Total	-1.331	-1.085	-0.483		1.705	1.403	1.17	

Mauritania March 2008	Mean	Mean	Mean	Mean	Sd	Sd	Sd	Sd
	who htz	who wtz	who wfl	muac	who htz	who wtz	who wfl	muac
Centre	-1.495	-1.444	-0.825		1.618	1.193	1.151	
Fleuve Nord	-1.382	-1.32	-0.767		1.508	1.188	1.22	
Fleuve Sud	-1.142	-1.21	-0.801		1.612	1.216	1.195	
Nord	-0.88	-0.529	-0.07		1.732	1.273	1.293	
Nouakchott	-0.941	-0.726	-0.274		1.726	1.296	1.286	
SudEst	-1.699	-1.463	-0.732		1.501	1.259	1.244	
Total	-1.287	-1.159	-0.614		1.631	1.279	1.258	

Mauritania Dec 2008	Mean	Mean	Mean	Mean	Sd	Sd	Sd	Sd
	who htz	who wtz	who wfl	muac	who htz	who wtz	who wfl	muac
Centre	-1.108	-1.202	-0.823	14.155	1.299	1.011	1.012	1.257
Nord	-1.21	-0.813	-0.17	14.347	1.348	1.019	0.962	1.473
Nouakchott A	-0.681	-0.585	-0.282	14.669	1.203	1.032	1.069	1.436
Nouakchott B	-0.886	-0.735	-0.334	14.582	1.204	0.983	0.989	1.473
Sud	-1.161	-1.256	-0.85	14.091	1.282	1.029	1.074	1.301
SudEst	-1.241	-1.084	-0.562	14.261	1.199	1.039	1.05	1.361
Trarza	-0.998	-0.597	-0.058	14.761	1.103	0.945	1.008	1.537
Total	-1.047	-0.918	-0.467	14.394	1.247	1.043	1.067	1.422

Mauritania 2009	Mean	Mean	Mean	Mean	Sd	Sd	Sd	Sd
	who htz	who wtz	who wfl	muac	who htz	who wtz	who wfl	muac
Centre	-1.162	-1.384	-1.043		1.21	0.988	1.102	
Nord	-1.031	-0.972	-0.553		1.375	0.964	1.024	
Nouakchott	-0.74	-0.726	-0.458		1.164	0.961	1.089	
Sud	-1.226	-1.381	-0.983		1.193	1.034	1.07	
Sud-est	-1.142	-1.252	-0.872		1.281	0.944	1.105	
Trarza	-1.016	-0.919	-0.503		1.329	0.984	1.108	
Total	-1.063	-1.12	-0.747		1.267	1.013	1.107	

Mauritania July 2010	Mean	Mean	Mean	Mean	Sd	Sd	Sd	Sd
	who htz	who wtz	who wfl	muac	who htz	who wtz	who wfl	muac
adrar/inchiri tiris	-1.684	-1.221	-0.405		1.249	0.983	0.964	
assaba	-1.15	-1.189	-0.809		0.997	0.894	0.876	
brakna	-0.998	-1.295	-1.066		1.29	1.019	1.031	
gorgol	-1.112	-1.437	-1.175		1.249	0.969	0.953	
guidimakha	-1.271	-1.487	-1.125		1.49	1.161	1.058	
hodh chargui	-1.42	-1.411	-0.895		1.186	1.002	0.996	
hodh gharbi	-0.995	-1.239	-0.994		1.431	0.973	0.93	
nouadhibou	-1.156	-0.742	-0.158		1.34	1.036	1.05	
nouakchott	-0.932	-0.773	-0.391		1.454	1.146	1.244	
tagant	-1.246	-1.356	-0.959		1.255	0.923	0.972	
trarza	-1.037	-1.03	-0.665		1.287	0.959	1.022	
Total	-1.171	-1.214	-0.816		1.305	1.031	1.053	

Mauritania Dec 2010	Mean	Mean	Mean	Mean	Sd	Sd	Sd	Sd
	who htz	who wtz	who wfl	muac	who htz	who wtz	who wfl	muac

adrar/inchiri tiris	-1.03	-0.903	-0.461	14.733	1.378	1.086	1.066	1.267
assaba	-1.189	-0.994	-0.497	14.51	1.29	1.012	0.999	1.151
brakna	-0.899	-1.014	-0.744	14.397	1.296	1.033	1.039	1.188
gorgol	-1.075	-1.098	-0.732	14.35	1.238	0.948	0.963	1.112
guidimakha	-1.181	-1.305	-0.932	14.125	1.358	1.069	0.966	1.063
hodh chargui	-1.564	-1.31	-0.633	14.48	1.131	1.026	1.049	1.222
hodh gharbi	-0.938	-0.786	-0.378	15.102	1.357	1.033	0.993	1.251
nouadhibou	-0.744	-0.53	-0.173	15.429	1.208	0.91	0.945	1.433
nouakchott	-0.778	-0.598	-0.238	14.8	1.337	1.058	1.071	1.276
tagant	-1.358	-1.103	-0.522	14.502	1.196	0.938	1.055	1.198
trarza	-1.004	-0.818	-0.393	14.991	1.268	0.957	0.976	1.089
Total	-1.068	-0.954	-0.525	14.666	1.299	1.035	1.034	1.262

Mauritania July 2011	Mean	Mean	Mean	Mean	Sd	Sd	Sd	Sd
	who htz	who wtz	who wfl	muac	who htz	who wtz	who wfl	muac
adrar/inchiri tiris	-1.11	-1.03	-0.584	14.708	1.367	1.057	1.048	1.216
assaba	-1.321	-1.467	-1.062	14.386	1.282	0.992	0.995	1.076
brakna	-1.189	-1.424	-1.1	14.095	1.217	0.971	1.034	1.113
gorgol	-0.951	-1.237	-1.034	13.964	1.094	0.881	0.917	1.069
guidimakha	-1.383	-1.517	-1.036	13.719	1.466	1.13	1.003	1.116
hodh chargui	-1.606	-1.522	-0.918	14.256	1.178	1.035	0.987	1.163
hodh gharbi	-1.181	-1.27	-0.886	14.479	1.156	0.814	1.004	1.174
nouadhibou	-0.699	-0.432	-0.088	15.452	1.026	0.968	1.077	1.296
nouakchott	-0.684	-0.632	-0.35	14.859	1.177	0.932	0.939	1.109
tagant	-1.085	-1.119	-0.749	14.298	1.196	0.956	0.978	1.085
trarza	-0.822	-0.702	-0.358	14.701	1.246	1.054	1.084	1.107
Total	-1.093	-1.128	-0.751	14.434	1.255	1.047	1.059	1.225

Mauritania Dec 2011	Mean	Mean	Mean	Mean	Sd	Sd	Sd	Sd
	who htz	who wtz	who wfl	muac	who htz	who wtz	who wfl	muac
adrar/inchiri tiris	-1.526	-1.121	-0.383	14.172	1.226	0.963	0.954	1.244
assaba	-1.329	-1.205	-0.681	14.622	1.248	1.023	0.956	1.137
brakna	-1.148	-1.186	-0.805	14.344	1.239	1.008	1.022	1.079
gorgol	-1.319	-1.257	-0.745	14.021	1.472	1.072	1.096	1.034
guidimakha	-1.667	-1.435	-0.708	13.703	1.205	1.054	0.995	1.135
hodh chargui	-1.654	-1.229	-0.443	14.5	1.128	1.015	1.031	1.146
hodh gharbi	-1.076	-1.018	-0.617	14.514	1.119	0.867	0.902	1.084
nouadhibou	-1.055	-0.586	-0.018	14.897	1.223	0.984	1.059	1.33
nouakchott	-0.874	-0.67	-0.255	14.969	1.219	0.978	1.021	1.275
tagant	-1.075	-1.05	-0.672	14.432	1.207	1.032	1.014	1.143
trarza	-0.968	-0.678	-0.193	14.633	1.001	0.892	0.986	1.156
Total	-1.245	-1.046	-0.512	14.423	1.242	1.027	1.033	1.215

Mauritania July 2012	Mean	Mean	Mean	Mean	Sd	Sd	Sd	Sd
	who htz	who wtz	who wfl	muac	who htz	who wtz	who wfl	muac
adrar/inchiri tiris	-1.175	-1.176	-0.764	14.147	1.325	1.08	1.067	1.35
assaba	-0.979	-1.177	-0.896	13.981	1.671	1.224	1.09	1.174
brakna	-1.251	-1.331	-0.918	14.282	1.403	1.118	1.156	1.117
gorgol	-0.827	-0.99	-0.773	14.457	1.141	1.034	1.085	1.177
guidimakha	-1.338	-1.45	-1.027	13.716	1.232	0.962	0.968	1.199
hodh chargui	-1.501	-1.562	-1.017	13.824	1.183	1.034	1.038	1.219
hodh gharbi	-1.234	-1.346	-0.941	13.857	1.289	1.016	1.024	1.23
nouadhibou	-0.462	-0.25	-0.008	14.937	1.243	1.007	1.085	1.354
nouakchott	-0.501	-0.519	-0.358	14.794	1.333	1.123	1.116	1.428
tagant	-1.025	-1.361	-1.142	13.983	1.285	1.046	1.007	1.237
trarza	-0.693	-0.755	-0.534	14.493	1.436	1.091	1.113	1.253
Total	-1.034	-1.135	-0.806	14.169	1.36	1.129	1.104	1.301

Niger 2012	Mean	Mean	Mean	Mean	Sd	Sd	Sd	Sd

	who htz	who wtz	who wfl	muac	who htz	who wtz	who wfl	muac
agadez	-1.299	-1.333	-0.858		1.389	1.082	0.994	
diffa	-1.771	-1.683	-0.962		1.332	1.132	1.077	
dosso	-1.522	-1.407	-0.779		1.322	1.154	1.059	
maradi	-1.857	-1.63	-0.789		1.42	1.166	1.134	
niamey	-0.9	-0.993	-0.695		1.223	1.048	1.064	
tahoua	-1.733	-1.469	-0.669		1.534	1.299	1.207	
tillabéri	-1.488	-1.47	-0.881		1.23	1.153	1.1	
zinder	-1.926	-1.738	-0.927		1.429	1.227	1.113	
Total	-1.56	-1.46	-0.813		1.406	1.183	1.102	

Nigeria (Northern States) 2011	Mean	Mean	Mean	Mean	Sd	Sd	Sd	Sd
	who htz	who wtz	who wfl	muac	who htz	who wtz	who wfl	muac
jigawa	-2.087	-1.635	-0.598	13.97	1.441	1.2	1.251	1.564
kano	-1.905	-1.487	-0.552	14.208	1.435	1.179	1.194	1.462
katsina	-1.812	-1.474	-0.631	14.026	1.384	1.118	1.218	1.442
kebbi	-1.677	-1.371	-0.586	13.814	1.261	1.104	1.176	1.497
sokoto	-1.628	-1.379	-0.628	13.92	1.269	1.083	1.151	1.448
yobe	-1.597	-1.345	-0.589	14.105	1.38	0.993	1.094	1.34
zamfara	-1.588	-1.316	-0.581	13.84	1.442	1.148	1.226	1.571
Total	-1.748	-1.425	-0.595	13.98	1.383	1.12	1.186	1.48

Senegal 2012	Mean	Mean	Mean	Mean	Sd	Sd	Sd	Sd
	who htz	who wtz	who wfl	muac	who htz	who wtz	who wfl	muac
dakar	-0.31	-0.436	-0.383	15.27	1.154	1.075	1.076	1.227
kolda	-1.178	-1.148	-0.698	14.666	1.506	1.161	1.021	1.258
matam	-0.843	-1.208	-1.08	14.475	1.189	1.029	0.981	1.147
myf	-1.363	-1.19	-0.596	14.687	1.378	1.133	1.031	1.191
sedhiou	-1.335	-1.248	-0.696	14.75	1.295	1.066	0.978	1.124
tambacounda	-1.042	-1.221	-0.919	14.422	1.235	1.054	1.031	1.183
velingara	-1.177	-1.204	-0.776	14.683	1.375	1.134	1.039	1.089
Total	-1.077	-1.183	-0.833	14.59	1.297	1.082	1.027	1.18

Sierra Leone 2010	Mean	Mean	Mean	Mean	Sd	Sd	Sd	Sd
	who htz	who wtz	who wfl	muac	who htz	who wtz	who wfl	muac
eastern	-1.486	-1.095	-0.329	14.385	1.288	1.132	1.174	1.58
northern	-1.471	-1.039	-0.279	14.488	1.187	1.022	1.051	1.407
southern	-1.623	-1.205	-0.385	14.373	1.28	1.114	1.107	1.565
western	-1.016	-0.981	-0.535	14.426	1.528	1.263	1.208	1.615
Total	-1.444	-1.088	-0.361	14.425	1.309	1.114	1.121	1.522

Togo June 2012	Mean	Mean	Mean	Mean	Sd	Sd	Sd	Sd
	who htz	who wtz	who wfl	muac	who htz	who wtz	who wfl	muac
Centrale	-1.272	-0.818	-0.139	15.179	1.245	1.105	1.104	1.33
Kara	-1.396	-1.039	-0.34	14.768	1.259	1.113	1.066	1.32
Lomé	-0.82	-0.732	-0.392	15.27	1.114	1.015	1.006	1.205
Maritime	-1.323	-1.026	-0.353	15.173	1.262	1.094	1.037	1.301
Plateaux	-1.179	-0.826	-0.207	15.042	1.228	1.101	1.02	1.413
Savanes	-1.593	-1.35	-0.638	14.366	1.198	1.073	1.033	1.115
Total	-1.307	-1.001	-0.361	14.914	1.243	1.106	1.059	1.318

Togo Dec 2012	Mean	Mean	Mean	Mean	Sd	Sd	Sd	Sd
	who htz	who wtz	who wfl	muac	who htz	who wtz	who wfl	muac
Kara	-1.505	-1.148	-0.429	14.571	1.231	1.004	1.039	1.312
Savanes	-1.542	-1.372	-0.694	14.285	1.163	1.015	1.005	1.229
Total	-1.526	-1.274	-0.578	14.411	1.193	1.016	1.028	1.273

Mean and standard deviation (SD) for height-for-age (HAZ), weight-for-age (WTZ), and weight-for-height (WHZ), by region MICS

Burkina Faso 2006	Mean	Mean	Mean	Sd	Sd	Sd
	who htz	who wtz	who wfl	who htz	who wtz	who wfl
Boucle du Mouhoun	-1.637	-1.415	-0.598	1.62	1.502	1.824
Cascade	-1.448	-1.743	-0.831	2.11	1.835	1.925
Centre	-1.082	-1.13	-0.625	1.60	1.477	1.781
Centre-Est	-2.059	-1.414	-0.155	1.83	1.617	2.01
Centre-Nord	-1.638	-1.259	-0.393	1.83	1.708	1.877
Centre-Ouest	-1.763	-1.967	-1.313	1.57	1.359	1.504
Centre-Sud	-1.793	-1.473	-0.524	1.80	1.431	1.694
Est	-1.684	-1.81	-0.883	2.33	1.737	1.986
Hauts-Bassins	-1.398	-1.128	-0.411	1.85	1.614	1.792
Nord	-1.598	-1.862	-0.999	2.04	1.842	2.016
Plateau-Central	-1.757	-1.384	-0.44	1.77	1.506	1.894
Sahel	-2.271	-2.263	-0.795	1.90	1.834	2.125
Sud-Ouest	-1.243	-1.236	-0.613	1.91	1.615	1.844
Total	-1.629	-1.568	-0.692	1.89	1.665	1.888

Cameroon 2006	Mean	Mean	Mean	Sd	Sd	Sd
	who htz	who wtz	who wfl	who htz	who wtz	who wfl
Adamaoua	-1.395	-0.756	0.088	1.76	1.28	1.264
Centre	-1.254	-0.42	0.42	1.65	1.269	1.367
Douala	-0.605	0.187	0.682	1.82	1.257	1.508
Est	-1.538	-0.827	0.057	1.71	1.348	1.353
Extreme Nord	-1.631	-1.435	-0.669	1.57	1.224	1.276
Littoral	-1.218	-0.246	0.603	1.56	1.218	1.389
Nord	-1.871	-1.235	-0.18	1.86	1.631	1.842
Nord Ouest	-1.527	-0.432	0.661	1.93	1.478	1.569
Ouest	-1.263	-0.176	0.739	1.84	1.273	1.197
Sud	-1.23	-0.478	0.397	1.79	1.397	1.414
Sud Ouest	-1.72	-0.805	0.246	1.65	1.304	1.341
Yaounde	-0.683	0.051	0.618	1.70	1.182	1.367
Total	-1.383	-0.644	0.214	1.77	1.421	1.485

Central African Rep 2000	Mean	Mean	Mean	Sd	Sd	Sd
	who htz	who wtz	who wfl	who htz	who wtz	who wfl
Bamingui-Bangoran	-1.891	-0.992	0.248	1.88	1.394	1.512
Bangui	-1.677	-0.852	0.218	1.86	1.321	1.727
Basse-Kotto	-1.715	-1.132	-0.067	2.08	1.581	1.96
Haut-Mbomou	-1.485	-0.736	0.216	2.01	1.22	1.62
Haute-Kotto	-1.362	-0.602	0.253	1.74	1.281	1.449
Kémo	-1.325	-0.805	0.049	2.07	1.43	1.661
Lobaye	-2.131	-1.045	0.367	2.01	1.356	1.718
Mambéré-Kadeï	-2.118	-1.089	0.335	1.74	1.384	1.37
Mbomou	-1.746	-0.878	0.266	1.93	1.569	1.766
Nana-Grébizi	-1.506	-0.963	-0.145	1.68	1.372	1.338
Nana-Mambéré	-2.025	-1.065	0.33	1.95	1.373	1.759
Ombella-M'poko	-1.389	-0.986	-0.223	1.94	1.496	1.618
Ouaka	-1.401	-1.209	-0.492	2.09	1.388	1.806
Ouham	-1.252	-0.818	-0.057	2.09	1.43	1.546
Ouham-Pendé	-1.357	-1.031	-0.035	2.43	1.502	1.691
Sangha-Mbaéré	-1.91	-1.111	0.156	1.81	1.322	1.444
Vakaga	-1.568	-1.153	-0.243	2.12	1.471	1.614
Total	-1.636	-0.969	0.057	1.98	1.419	1.661

Central African Rep 2006	Mean	Mean	Mean	Sd	Sd	Sd
	who htz	who wtz	who wfl	who htz	who wtz	who wfl
“Baminigui Bangoran”	-1.12	-0.889	-0.349	2.21	1.7	1.798
“Bangui”	-1.066	-0.95	-0.342	2.01	1.495	1.617
“Basse Kotto”	-1.666	-1.052	-0.032	2.25	1.539	1.78
“Haut Mbomou”	-1.071	-0.441	0.139	2.00	1.511	1.625
“Haute Kotto”	-1.185	-0.826	-0.186	1.93	1.506	1.663
“Lobaye”	-1.508	-0.96	-0.076	2.07	1.606	1.603
“Mambere Kadei”	-1.767	-1.306	-0.297	2.03	1.549	1.568
“Mbomou”	-2.178	-0.945	0.58	1.97	1.545	1.623
“Nana Mambere”	-1.743	-1.055	0.085	2.23	1.679	1.774
“Ombella Mpoko”	-1.132	-1.064	-0.451	2.15	1.694	1.539
“Ouaka”	-1.817	-1.195	-0.159	1.89	1.433	1.605
“Ouham Pende”	-1.554	-1.261	-0.498	2.24	1.588	1.571
“Ouham”	-1.859	-1.097	0.091	1.96	1.4	1.502
“Sangha Mbaere”	-2.016	-1.472	-0.3	1.85	1.47	1.327
Total	-1.57	-1.058	-0.133	2.08	1.569	1.636

Central African Rep 2010	Mean	Mean	Mean	Sd	Sd	Sd
	who htz	who wtz	who wfl	who htz	who wtz	who wfl
Baminigui Bangoran	-1.083	-0.855	-0.317	1.48	1.196	1.124
Bangui	-1.268	-1.122	-0.554	1.54	1.261	1.215
Basse Kotto	-1.571	-1.069	-0.199	1.39	1.087	1.02
Haut Mbomou	-0.897	-0.626	-0.163	1.38	1.119	1.026
Haute-Kotto	-1.007	-0.764	-0.242	1.52	1.177	1.057
Kémo	-1.699	-1.237	-0.362	1.62	1.282	1.059
Lobaye	-1.937	-1.516	-0.528	1.55	1.316	1.251
Mambere Kadei	-1.892	-1.339	-0.318	1.59	1.289	1.155
Mbomou	-1.514	-1.012	-0.186	1.41	1.178	1.014
Nana Grebizi	-1.381	-1.027	-0.304	1.56	1.23	1.219
Nana Mambéré	-2.164	-1.457	-0.222	1.42	1.226	1.166
Ombella Mpoko	-1.714	-1.174	-0.236	1.43	1.174	1.147
Ouaka	-1.561	-1.157	-0.361	1.32	1.035	1.005
Ouham	-1.52	-1.173	-0.4	1.60	1.293	1.144
Ouham Pende	-1.462	-0.879	-0.014	1.76	1.225	1.148
Sangha Mbaere	-2.036	-1.459	-0.393	1.56	1.26	1.146
Vakaga	-0.827	-1.105	-0.846	1.41	1.183	1.018
Total	-1.589	-1.152	-0.311	1.56	1.242	1.135

Chad 2000	Mean	Mean	Mean	Sd	Sd	Sd
	who htz	who wtz	who wfl	who htz	who wtz	who wfl
Autres villes	-1.406	-1.214	-0.557	1.74	1.468	1.406
N'Djaména	-1.212	-1.112	-0.582	1.71	1.338	1.242
Rural	-1.56	-1.318	-0.58	1.84	1.476	1.361
Total	-1.448	-1.247	-0.572	1.79	1.454	1.359

Chad 2010	Mean	Mean	Mean	Sd	Sd	Sd
	who htz	who wtz	who wfl	who htz	who wtz	who wfl
Barh El Gazal	-2.002	-2.035	-1.12	1.98	1.472	1.423
Bhata	-1.32	-1.462	-0.973	2.11	1.491	1.363
Chari Baguirmi	-1.632	-1.636	-0.972	1.95	1.419	1.42
Guéra	-1.569	-1.585	-0.91	2.00	1.419	1.274
Hadjer Lamis	-1.363	-1.799	-1.273	2.22	1.357	1.494
Kanem	-1.83	-2.179	-1.086	2.41	1.465	1.334
Lac	-2.159	-2.033	-0.923	2.17	1.502	1.254
Logone Occidental	-1.208	-1.125	-0.567	1.93	1.464	1.428
Logone Oriental	-1.057	-0.728	-0.142	1.92	1.465	1.497
Mandoul	-1.086	-0.682	-0.09	1.78	1.393	1.285
Mayo Kebbi Est	-1.497	-1.361	-0.459	2.05	1.435	1.377

Mayo Kebbi Ouest	-1.822	-1.363	-0.396	1.79	1.363	1.328
Moyen Chari	-0.961	-0.665	-0.163	1.91	1.349	1.251
Ndjaména	-0.793	-0.78	-0.426	1.90	1.378	1.331
Ouaddai	-1.786	-1.705	-0.772	2.00	1.453	1.433
Salamat	-1.175	-1.517	-0.91	2.30	1.709	1.45
Sila	-1.076	-1.547	-1.179	2.41	1.494	1.704
Tandjilé	-1.337	-1.316	-0.507	2.15	1.52	1.475
Wad Fira	-1.002	-1.483	-1.173	2.19	1.446	1.272
bet	-1.688	-1.855	-1.165	1.98	1.423	1.41
Total	-1.406	-1.417	-0.732	2.09	1.513	1.44

Congo DR 2001	Mean	Mean	Mean	Sd	Sd	Sd
	who htz	who wtz	who wfl	who htz	who wtz	who wfl
Bandundu	-1.642	-1.496	-0.646	1.97	1.436	1.629
Bas-congo	-1.886	-1.517	-0.473	1.98	1.414	1.432
Equateur	-1.632	-1.4	-0.46	2.21	1.454	1.694
Kasai Occidental	-1.567	-1.385	-0.579	2.16	1.495	1.678
Kasai Oriental	-1.416	-1.104	-0.331	2.17	1.489	1.755
Katanga	-1.629	-1.418	-0.532	2.05	1.506	1.62
Kinshasa	-0.684	-0.839	-0.565	1.93	1.328	1.523
Maniema	-1.93	-1.446	-0.331	1.91	1.625	1.856
Nord-Kivu	-2.082	-1.39	-0.049	1.84	1.436	1.594
Orientale	-1.724	-1.209	-0.121	1.95	1.396	1.54
Sud-Kivu	-2.043	-1.573	-0.361	2.01	1.476	1.696
Total	-1.554	-1.297	-0.431	2.07	1.462	1.638

Congo DR 2010	Mean	Mean	Mean	Sd	Sd	Sd
	who htz	who wtz	who wfl	who htz	who wtz	who wfl
Bandundu	-1.269	-1.058	-0.541	1.86	1.429	1.442
Bas congo	-1.592	-1.316	-0.551	1.67	1.255	1.189
Equateur	-1.472	-1.083	-0.32	1.85	1.359	1.344
Kasai Occidental	-1.944	-1.363	-0.318	1.69	1.322	1.208
Kasai Oriental	-1.804	-1.172	-0.28	1.82	1.56	1.316
Katanga	-1.579	-1.015	-0.075	2.08	1.456	1.524
Kinshasa	-0.96	-0.81	-0.35	1.61	1.146	1.192
Maniema	-1.397	-0.811	-0.075	2.01	1.474	1.484
Nord Kivu	-2.049	-1.087	0.182	1.90	1.409	1.403
Province Orientale	-1.445	-1.051	-0.237	1.90	1.361	1.392
Sud Kivu	-1.968	-1.164	-0.035	1.76	1.365	1.223
Total	-1.613	-1.086	-0.218	1.87	1.397	1.365

Côte d'Ivoire 2006	Mean	Mean	Mean	Sd	Sd	Sd
	who htz	who wtz	who wfl	who htz	who wtz	who wfl
Centre	-1.531	-0.87	0.072	1.62	1.303	1.368
Centre Est	-1.466	-0.861	-0.057	1.51	1.071	1.263
Centre Nord	-1.263	-0.921	-0.237	1.88	1.372	1.629
Centre Ouest	-1.415	-0.81	0.015	1.94	1.219	1.501
Nord	-1.615	-1.261	-0.429	1.71	1.221	1.48
Nord Est	-2.086	-1.248	0.113	1.71	1.366	1.555
Nord Ouest	-1.464	-0.92	-0.259	2.19	1.629	1.717
Ouest	-1.434	-0.906	-0.07	1.98	1.311	1.499
Sud (sans ville d' Abidjan)	-1.623	-0.764	0.218	1.70	1.25	1.491
Sud Ouest	-1.953	-0.785	0.502	1.81	1.318	1.474
Ville Abidjan	-1.273	-0.571	0.271	1.68	1.16	1.442
Total	-1.555	-0.875	0.043	1.83	1.32	1.526

Equatorial Guinea 2000	Mean	Mean	Mean	Sd	Sd	Sd
	who htz	who wtz	who wfl	who htz	who wtz	who wfl
Annobon	-0.732	-0.668	-0.261	2.86	1.107	1.763

Bioko Norte	-1.378	-0.388	0.517	2.21	1.469	1.71
Bioko Sur	-1.789	-0.942	0.461	1.88	1.035	1.089
Centro Sur	-1.709	-0.521	0.507	1.99	1.666	1.75
Kie Ntem	-1.646	-0.802	0.29	1.91	1.56	1.54
Litoral	-1.505	-0.624	0.348	2.14	1.437	1.827
Wele Nzas	-1.763	-0.853	0.385	2.03	1.586	1.52
Total	-1.564	-0.627	0.392	2.08	1.518	1.684

**Gambia 2000**	**Mean**	**Mean**	**Mean**	**Sd**	**Sd**	**Sd**
	**who htz**	**who wtz**	**who wfl**	**who htz**	**who wtz**	**who wfl**
Banjul	-0.664	-0.268	0.263	1.77	1.23	1.32
Basse	-1.045	-1.163	-0.681	1.78	1.212	1.163
Brikama	-0.947	-0.688	-0.184	1.47	1.147	1.129
Janjabureh	-1.398	-1.174	-0.549	2.14	1.536	1.585
Kanifing	-0.825	-0.562	-0.043	1.60	1.237	1.304
Kerewan	-1.206	-0.991	-0.342	1.78	1.351	1.367
Kuntaur	-1.145	-1.194	-0.671	1.81	1.202	1.218
Mansakonko	-1.049	-0.883	-0.41	1.74	1.329	1.38
Total	-1.036	-0.872	-0.333	1.78	1.317	1.342

**Gambia 2005**	**Mean**	**Mean**	**Mean**	**Sd**	**Sd**	**Sd**
	**who htz**	**who wtz**	**who wfl**	**who htz**	**who wtz**	**who wfl**
Banjul	-0.972	-0.796	-0.382	1.46	1.116	0.992
Basse	-1.341	-1.046	-0.381	1.46	1.184	1.109
Brikama	-0.979	-0.752	-0.251	1.55	1.211	1.317
Janjanburay	-1.534	-1.09	-0.31	1.34	1.114	1.042
Kanifing	-0.7	-0.578	-0.273	1.55	1.139	1.163
Kerewan	-1.576	-1.006	-0.128	1.62	1.269	1.392
Kuntaur	-1.399	-1.254	-0.642	1.56	1.205	1.302
Mansakonko	-1.441	-1.118	-0.46	1.51	1.206	1.192
Total	-1.176	-0.892	-0.309	1.55	1.204	1.221

**Ghana 2006**	**Mean**	**Mean**	**Mean**	**Sd**	**Sd**	**Sd**
	**who htz**	**who wtz**	**who wfl**	**who htz**	**who wtz**	**who wfl**
Ashanti	-1.137	-0.809	-0.23	1.59	1.229	1.228
Brong Ahafo	-1.353	-0.742	0.039	1.38	1.048	1.126
Central	-1.422	-0.923	-0.083	1.33	1.035	1.018
Eastern	-1.307	-0.947	-0.265	1.49	1.129	1.131
Greater Accra	-0.692	-0.399	0.009	1.31	1.07	1.141
Northern	-1.483	-1.19	-0.446	1.59	1.204	1.222
Upper East	-1.458	-1.157	-0.553	1.67	1.353	1.354
Upper West	-1.301	-0.97	-0.222	1.42	1.159	1.203
Volta	-1.175	-0.971	-0.416	1.60	1.188	1.163
Western	-1.093	-0.719	-0.188	1.50	1.008	1.122
Total	-1.26	-0.912	-0.261	1.52	1.181	1.198

**Ghana 2011**	**Mean**	**Mean**	**Mean**	**Sd**	**Sd**	**Sd**
	**who htz**	**who wtz**	**who wfl**	**who htz**	**who wtz**	**who wfl**
Asante	-1.02	-0.725	-0.2	1.42	1.155	1.243
Brong Ahafo	-1.051	-0.747	-0.149	1.23	1.012	1.054
Central	-1.135	-0.886	-0.352	1.36	1.058	1.091
Eastern	-0.986	-0.722	-0.229	1.31	1.149	1.176
Greater Accra	-0.712	-0.564	-0.245	1.26	1.047	1.128
Northern	-1.642	-1.27	-0.462	1.29	1.143	1.137
Upper East	-1.44	-1.241	-0.565	1.28	1.053	1.101
Upper West	-1.158	-0.985	-0.502	1.41	1.106	1.166
Volta	-0.89	-0.811	-0.438	1.46	1.025	1.175
Western	-1.141	-0.819	-0.281	1.43	1.178	1.214
Total	-1.26	-1	-0.401	1.37	1.123	1.147

Guinea Bissau 2000	Mean	Mean	Mean	Sd	Sd	Sd
	who htz	who wtz	who wfl	who htz	who wtz	who wfl
Bafatá	-1.592	-1.136	-0.274	1.85	1.391	1.439
Biombo	-1.375	-0.879	-0.025	1.65	1.184	1.574
Bolama/Bijagós	-1.202	-0.784	-0.16	1.60	1.28	1.301
Cacheu	-1.364	-0.99	-0.246	1.69	1.283	1.445
Gabú	-1.796	-1.377	-0.38	2.06	1.658	1.584
Oio	-1.496	-1.319	-0.61	1.78	1.462	1.517
Quinará	-1.058	-1.035	-0.521	1.78	1.331	1.386
Tombali	-1.124	-1.011	-0.402	1.82	1.275	1.402
sab	-0.827	-0.572	-0.198	1.57	1.249	1.356
Total	-1.347	-1.036	-0.336	1.79	1.407	1.467

Guinea Bissau 2006	Mean	Mean	Mean	Sd	Sd	Sd
	who htz	who wtz	who wfl	who htz	who wtz	who wfl
EST (Bafata e Gabu)	-2.05	-1.138	0.257	2.10	1.388	1.844
NORD (Biombo, Cacheu e Oio)	-1.693	-0.929	0.253	2.05	1.267	1.773
SAB Capital	-1.581	-0.51	0.654	1.78	1.486	1.643
SUD (Bolama, Quinara e Tombali)	-1.945	-0.721	0.583	2.20	1.385	2.016
Total	-1.8	-0.881	0.365	2.05	1.366	1.818

Mauritania 2007	Mean	Mean	Mean	Sd	Sd	Sd
	who htz	who wtz	who wfl	who htz	who wtz	who wfl
Adrar	-1.376	-1.388	-0.79	1.61	1.269	1.193
Assaba	-1.237	-1.353	-0.855	1.69	1.302	1.273
Brakna	-0.96	-1.196	-0.92	1.79	1.367	1.324
Gorgol	-1.073	-1.43	-1.145	1.82	1.293	1.306
Guidimagha	-1.316	-1.44	-0.982	1.93	1.426	1.278
Hodh ECharghi	-1.193	-1.228	-0.751	1.78	1.286	1.343
Hodh ELGharbi	-1.133	-1.184	-0.728	1.76	1.261	1.225
Inchiri	-0.581	-0.96	-0.832	1.98	1.24	1.227
Nouadhibou	-0.941	-0.639	-0.11	1.53	1.294	1.227
Nouakchott	-0.756	-0.693	-0.318	1.71	1.187	1.255
Tagant	-1.443	-1.475	-0.827	1.82	1.349	1.458
Tiris Zemmour	-1.483	-1.082	-0.266	1.72	1.229	1.235
Trarza	-0.867	-0.919	-0.609	1.62	1.194	1.188
Total	-1.074	-1.132	-0.703	1.76	1.318	1.309

Niger 2000	Mean	Mean	Mean	Sd	Sd	Sd
	who htz	who wtz	who wfl	who htz	who wtz	who wfl
Diffa	-1.883	-1.844	-0.979	1.60	1.362	1.371
Agadez	-1.491	-1.462	-0.861	1.42	1.108	1.049
Dosso	-1.872	-1.608	-0.705	1.54	1.399	1.335
Maradi	-2.336	-1.948	-0.793	1.54	1.299	1.267
Niamey	-1.077	-1.11	-0.721	1.49	1.208	1.18
Tahoua	-2.112	-1.649	-0.525	1.65	1.309	1.408
Tillaberi	-1.712	-1.684	-0.939	1.48	1.247	1.281
Zinder	-2.364	-2.198	-1.06	1.74	1.385	1.37
Total	-1.911	-1.7	-0.796	1.62	1.33	1.292

Nigeria 2007	Mean	Mean	Mean	Sd	Sd	Sd
	who htz	who wtz	who wfl	who htz	who wtz	who wfl
Abia	-1.177	-0.784	-0.078	1.82	1.258	1.47
Abuja FCT	-0.511	-0.228	-0.255	2.51	1.887	1.647
Adamawa	-1.067	-0.177	0.786	2.71	2.349	2.075
Akwa-Ibom	-1.315	-1.128	-0.399	1.98	1.442	1.44
Anambra	-0.538	-0.434	-0.209	2.11	1.74	1.499
Bauchi	-1.613	-0.787	0.407	2.79	2.445	2.068

Bayelsa	-0.982	-0.71	-0.073	1.82	1.439	1.432
Benue	-1.031	-0.735	-0.062	2.03	1.431	1.461
Borno	-1.234	-1.049	-0.161	2.74	2.45	2.112
Cross-Rivers	-0.837	-0.801	-0.325	1.94	1.272	1.304
Delta	-1.147	-0.838	-0.175	1.86	1.352	1.434
Ebonyi	-1.234	-0.723	-0.127	2.08	1.453	1.667
Edo	-1.046	-0.709	-0.044	1.89	1.307	1.451
Ekiti	-1.305	-0.741	0.075	2.02	1.372	1.585
Enugu	-0.751	-0.485	0.021	1.96	1.544	1.44
Gombe	-1.077	-1.087	-0.368	2.74	2.256	2.454
Imo	-1.048	-0.621	0.306	2.40	1.7	1.799
Jigawa	-2.47	-1.945	-0.402	2.18	1.657	1.901
Kaduna	-2.022	-1.033	0.172	2.20	1.809	2.069
Kano	-2.932	-1.459	0.044	2.26	2.144	2.061
Katsina	-1.973	-1.408	-0.298	2.67	1.678	2.033
Kebbi	-2.18	-1.185	-0.439	2.60	2.254	2.141
Kogi	-1.171	-0.865	-0.189	2.22	1.498	1.538
Kwara	-1.396	-1.103	-0.32	2.15	1.631	1.683
Lagos	-0.593	-0.673	-0.053	2.60	1.588	1.76
Nasarawa	-0.992	-0.519	0.05	2.21	1.79	1.956
Niger	-1.42	-1.018	-0.242	2.26	1.55	1.95
Ogun	-1.178	-0.775	-0.092	2.07	1.623	1.73
Ondo	-1.267	-0.495	0.18	2.17	1.716	1.704
Osun	-1.306	-0.822	-0.075	1.70	1.214	1.403
Oyo	-1.609	-0.993	-0.074	1.86	1.361	1.566
Plataeu	-0.757	-0.701	-0.191	2.58	1.696	1.89
Rivers	-1.309	-0.828	0.014	1.77	1.64	1.699
Sokoto	-2.841	-1.427	0.391	2.32	1.896	2.073
Taraba	-1.304	-0.924	-0.026	2.50	2.149	1.946
Yobe	-1.618	-1.112	-0.135	2.64	2.395	2.102
Zamfara	-2.026	-1.59	-0.49	2.31	2.194	1.797
Total	-1.459	-0.961	-0.092	2.37	1.876	1.84

**Nigeria 2011**	**Mean**	**Mean**	**Mean**	**Sd**	**Sd**	**Sd**
	**who htz**	**who wtz**	**who wfl**	**who htz**	**who wtz**	**who wfl**
Abia	-0.546	-0.692	-0.533	1.50	1.178	1.193
Adamawa	-1.858	-1.281	-0.283	1.76	1.345	1.131
Akwa ibom	-1.1	-0.896	-0.364	1.58	1.182	1.089
Anambra	-0.481	-0.415	-0.221	1.47	1.082	1.087
Bauchi	-2.197	-1.673	-0.509	1.76	1.308	1.245
Bayelsa	-0.744	-0.687	-0.376	1.56	1.164	1.105
Benue	-1.111	-0.618	0.024	1.77	1.375	1.286
Borno	-1.563	-1.398	-0.693	1.94	1.429	1.36
Cross River	-1.212	-0.839	-0.231	1.61	1.276	1.154
Delta	-0.758	-0.777	-0.504	1.70	1.289	1.26
Ebonyi	-1.044	-0.967	-0.512	1.63	1.134	1.101
Edo	-0.784	-0.606	-0.23	1.50	1.143	1.125
Ekiti	-0.755	-0.498	-0.066	1.44	1.111	1.163
Enugu	-0.554	-0.347	-0.065	1.41	1.139	1.187
FCT (Abuja)	-1.175	-0.862	-0.24	1.57	1.243	1.153
Gombe	-2.113	-1.578	-0.474	1.84	1.362	1.353
Imo	-0.654	-0.578	-0.3	1.43	1.178	1.061
Jigawa	-2.345	-1.733	-0.506	1.94	1.402	1.467
Kaduna	-1.773	-1.282	-0.287	1.93	1.382	1.317
Kano	-2.158	-1.557	-0.401	1.92	1.386	1.318
Katsina	-2.399	-1.778	-0.51	1.80	1.361	1.459
Kebbi	-1.942	-1.638	-0.742	2.14	1.625	1.395
Kogi	-0.906	-0.583	-0.036	1.81	1.348	1.354
Kwara	-1.181	-1.07	-0.528	1.82	1.333	1.245
Lagos	-0.529	-0.746	-0.607	1.37	1.179	1.204

Nasarawa	-1.128	-0.795	-0.207	1.75	1.258	1.229
Niger	-1.666	-1.27	-0.41	2.08	1.551	1.507
Ogun	-0.899	-0.894	-0.537	1.60	1.181	1.156
Ondo	-1.55	-0.663	0.366	1.81	1.234	1.417
Osun	-1.087	-0.835	-0.296	1.30	1.055	1.031
Oyo	-1.122	-0.961	-0.419	1.58	1.191	1.327
Plateau	-1.452	-0.951	-0.152	1.73	1.363	1.173
Rivers	-0.383	-0.485	-0.363	1.87	1.529	1.347
Sokoto	-1.8	-1.501	-0.642	1.90	1.37	1.533
Taraba	-1.37	-0.906	-0.144	1.93	1.327	1.238
Yobe	-2.477	-2.001	-0.761	1.90	1.426	1.322
Zamfara	-2.315	-1.847	-0.705	1.96	1.469	1.477
Total	-1.494	-1.16	-0.403	1.88	1.405	1.31

**Sao Tome et Principe 2000**	**Mean**	**Mean**	**Mean**	**Sd**	**Sd**	**Sd**
	**who htz**	**who wtz**	**who wfl**	**who htz**	**who wtz**	**who wfl**
Centro	-1.459	-0.632	0.306	1.63	1.213	1.376
Norte	-1.541	-0.674	0.304	1.61	1.236	1.402
Principe	-1.238	-0.366	0.555	1.46	1.16	0.991
Sul	-0.967	-0.554	0.231	1.37	1.091	1.192
Total	-1.442	-0.624	0.315	1.61	1.21	1.356

**Senegal 2000**	**Mean**	**Mean**	**Mean**	**Sd**	**Sd**	**Sd**
	**who htz**	**who wtz**	**who wfl**	**who htz**	**who wtz**	**who wfl**
Dakar	-0.932	-0.602	-0.108	1.47	1.35	1.411
Diourbel	-1.264	-1.068	-0.458	1.67	1.369	1.315
Fatick	-1.324	-1.078	-0.422	1.75	1.288	1.215
Kaolack	-1.508	-1.491	-0.828	1.76	1.367	1.302
Kolda	-1.721	-1.346	-0.486	1.95	1.426	1.167
Louga	-1.129	-0.917	-0.382	1.45	1.243	1.171
Saint louis	-0.883	-0.994	-0.696	1.58	1.275	1.169
Tambacounda	-1.276	-1.299	-0.697	1.94	1.418	1.302
Thies	-1.083	-0.774	-0.203	1.53	1.22	1.235
Ziguinchor	-1.114	-0.738	-0.13	1.70	1.266	1.17
Total	-1.204	-1.015	-0.435	1.69	1.346	1.275

**Sierra Leone 2000**	**Mean**	**Mean**	**Mean**	**Sd**	**Sd**	**Sd**
	**who htz**	**who wtz**	**who wfl**	**who htz**	**who wtz**	**who wfl**
East	-1.399	-1.264	-0.452	2.30	1.711	1.407
North	-1.383	-1.149	-0.406	2.02	1.462	1.411
South	-1.615	-0.874	0.011	2.33	1.688	1.77
West	-0.89	-0.791	-0.331	1.79	1.311	1.401
Total	-1.296	-1.051	-0.342	2.10	1.54	1.468

**Sierra Leone 2005**	**Mean**	**Mean**	**Mean**	**Sd**	**Sd**	**Sd**
	**who htz**	**who wtz**	**who wfl**	**who htz**	**who wtz**	**who wfl**
East	-1.551	-1.229	-0.353	2.07	1.53	1.478
North	-2.005	-1.4	-0.2	1.96	1.534	1.468
South	-1.724	-1.158	-0.103	1.93	1.424	1.527
West	-1.329	-0.77	0.07	1.80	1.382	1.468
Total	-1.759	-1.231	-0.178	1.97	1.499	1.492

**Sierra Leone 2010**	**Mean**	**Mean**	**Mean**	**Sd**	**Sd**	**Sd**
	**who htz**	**who wtz**	**who wfl**	**who htz**	**who wtz**	**who wfl**
East	-1.672	-1.092	0.032	1.79	1.393	1.51
North	-1.85	-1.097	0.051	1.88	1.392	1.543
South	-1.711	-0.856	0.196	1.91	1.418	1.578
West	-1.508	-0.773	0.316	1.96	1.527	1.763
Total	-1.731	-0.99	0.119	1.88	1.423	1.576

Togo 2006	Mean	Mean	Mean	Sd	Sd	Sd
	who htz	who wtz	who wfl	who htz	who wtz	who wfl
Centrale	-1.394	-0.942	-0.162	1.55	1.332	1.425
Kara	-1.615	-1.491	-0.63	2.16	1.546	1.873
Lomé commune	-0.84	-0.656	-0.246	1.56	1.26	1.284
Maritime (sans Lomé commune)	-1.092	-1.065	-0.581	1.80	1.433	1.443
Plateaux	-1.316	-0.908	-0.115	1.77	1.287	1.532
Savanes	-1.63	-2.092	-1.559	1.69	1.413	1.43
Total	-1.341	-1.284	-0.654	1.78	1.481	1.595

Togo 2010	Mean	Mean	Mean	Sd	Sd	Sd
	who htz	who wtz	who wfl	who htz	who wtz	who wfl
Centrale	-1.539	-1.047	-0.227	1.25	1.096	1.039
Kara	-1.546	-1.082	-0.29	1.42	1.139	1.077
Lomé	-0.81	-0.631	-0.255	1.24	1.062	1.057
Maritime	-1.269	-0.895	-0.224	1.31	1.118	1.016
Plateaux	-1.329	-0.916	-0.214	1.30	1.06	1.006
Savanes	-1.836	-1.547	-0.688	1.28	1.115	1
Total	-1.457	-1.082	-0.346	1.34	1.138	1.046

Appendix I

Skewness and Kurtosis for height-for-age (HAZ), weight-for-age (WTZ), and weight-for-height (WHZ), by region DHS

Benin 2001	Skewness	Skewness	Skewness	Kurtosis	Kurtosis	Kurtosis
	who htz	who wtz	who wfl	who htz	who wtz	who wfl
atacora	0.389	-0.136	-0.239	3.863	3.832	3.865
atlantique	0.248	-0.182	-0.203	3.347	3.636	4.24
borgou	0.451	0.077	0.026	3.417	4.011	3.857
mono	0.833	-0.323	-0.497	5.835	3.553	5.303
oueme	0.467	0.017	-0.247	4.211	4.305	4.849
zou	0.424	-0.216	-0.375	4.288	3.778	3.811
Total	0.425	-0.138	-0.254	4.011	3.881	4.194

Benin 2006	Skewness	Skewness	Skewness	Kurtosis	Kurtosis	Kurtosis
	who htz	who wtz	who wfl	who htz	who wtz	who wfl
alibori	0.486	0.082	-0.231	3.009	3.787	2.67
atacora	0.467	0.361	0.077	3.675	4.1	3.894
atlantique	0.551	0.247	-0.019	4.448	4.612	3.616
borgou	0.454	0.282	-0.157	3.302	4.716	3.437
collines	0.471	-0.015	0.192	4.278	4.334	3.906
couffo	0.225	0.104	0.067	3.793	3.55	4.222
donga	0.604	0.104	-0.05	3.386	4.396	3.116
littoral	0.048	0.242	0.137	4.546	4.665	3.735
mono	0.178	-0.085	0.03	3.441	3.503	3.049
plateau	0.809	0.266	-0.098	4.276	4.937	3.368
quémé	0.538	0.099	0.138	4.35	4.221	4.208
zou	0.206	-0.002	0.227	3.151	3.889	2.539
Total	0.377	0.093	0.128	3.805	4.259	3.513

Benin 2011	Skewness	Skewness	Skewness	Kurtosis	Kurtosis	Kurtosis
	who htz	who wtz	who wfl	who htz	who wtz	who wfl
Alibori	0.809	0.2	0.047	3.277	3.144	2.323
Atacora	0.563	0.332	0.124	2.824	3.728	2.481
Atlantique	0.608	0.202	0.036	3.067	2.765	2.333
Borgou	0.437	0.225	-0.087	2.704	3.335	2.49
Collines	0.565	0.392	-0.278	2.924	3.597	2.453
Couffo	0.455	0.206	-0.007	3.374	3.459	2.911
Donga	0.588	0.056	-0.145	2.945	3.698	2.295
Littoral	0.492	0.224	-0.085	2.983	3.022	2.666
Mono	0.201	0.3	0.136	2.939	3.748	3.026
Ou?m?	0.533	0.35	-0.016	3.174	3.964	2.681
Plateau	0.869	0.008	-0.259	3.503	2.428	2.231
Zou	0.459	0.272	0.094	2.962	3.723	2.487
Total	0.518	0.261	-0.013	2.983	3.473	2.541

Burkina Faso 1993	Skewness	Skewness	Skewness	Kurtosis	Kurtosis	Kurtosis
	who htz	who wtz	who wfl	who htz	who wtz	who wfl
central/south	0.523	0.025	-0.178	4.137	3.685	3.549
east	0.328	0.127	0.195	3.076	3.793	3.705
north	0.355	-0.17	0.004	3.161	3.6	4.464
ouagadougou	0.263	-0.065	-0.072	3.874	3.769	4.003
west	0.036	-0.128	-0.262	2.958	3.755	3.398
Total	0.273	-0.066	-0.075	3.41	3.721	3.831

Burkina Faso 1998	Skewness	Skewness	Skewness	Kurtosis	Kurtosis	Kurtosis
	who htz	who wtz	who wfl	who htz	who wtz	who wfl
central/south	0.43	0.013	-0.078	3.884	3.656	3.384
east	0.313	-0.186	-0.17	3.469	3.175	3.747
north	0.711	-0.197	-0.092	3.714	3.256	4.082
ouagadougou	0.391	-0.284	-0.124	4.187	3.323	3.412
west	0.313	-0.174	-0.322	3.492	3.049	3.423
Total	0.391	-0.172	-0.178	3.659	3.291	3.614

Burkina Faso 2003	Skewness	Skewness	Skewness	Kurtosis	Kurtosis	Kurtosis
	who htz	who wtz	who wfl	who htz	who wtz	who wfl
boucle de mouhoun	0.496	-0.108	-0.034	4.004	3.194	2.76
cascades	0.502	0.181	-0.037	3.908	3.083	2.686

centre (sans ouagadougou)	0.412	0.382	-0.316	3.933	5.004	3.401
centre-est	0.705	0.075	0.036	3.89	3.693	3.097
centre-nord	0.506	-0.064	0.247	3.613	3.629	3.299
centre-ouest	0.404	0.042	0.034	3.651	3.826	3.679
centre-sud	0.322	0.398	0.005	3.061	3.866	3.061
est	0.853	0.207	-0.179	3.904	3.748	2.614
hauts bassins	0.296	-0.399	-0.197	3.627	3.7	3.82
nord	0.449	-0.123	-0.012	3.399	3.1	3.291
ouagadougou	0.473	-0.238	0.019	4.027	3.266	3.7
plateau central	0.416	0.176	0.15	3.272	2.997	2.92
sahel	0.464	0.12	-0.225	3.19	3.17	2.89
sud-ouest	0.45	-0.028	-0.142	3.266	3.326	3.145
Total	0.435	0.017	0.093	3.48	3.495	3.28

**Burkina Faso 2010**	**Skewness**	**Skewness**	**Skewness**	**Kurtosis**	**Kurtosis**	**Kurtosis**
	**who htz**	**who wtz**	**who wfl**	**who htz**	**who wtz**	**who wfl**
boucle de mouhoun	-0.04	-0.246	-0.217	3.736	3.551	3.851
cascades	0.6	-0.056	-0.109	4.51	3.32	3.547
centre	0.386	-0.39	0.075	5.053	3.307	4.028
centre-est	0.423	-0.28	-0.17	3.93	3.608	3.49
centre-nord	0.07	-0.077	0.231	3.115	3.299	3.461
centre-ouest	0.185	-0.365	-0.248	3.832	3.563	3.537
centre-sud	0.424	0.555	0.053	3.875	4.01	3.683
est	0.427	0.26	0.036	3.496	3.969	3.829
hauts basins	0.498	-0.032	-0.253	4.336	3.523	3.385
nord	0.263	-0.168	-0.259	3.13	3.02	3.934
plateau central	0.497	0.167	0.071	4.636	4.034	3.458
sahel	0.218	-0.146	-0.061	3.89	4.053	3.277
sud-ouest	0.461	0.314	0.003	4.183	5.046	3.681
Total	0.364	-0.049	-0.047	3.989	3.908	3.768

**Cameroon 1991**	**Skewness**	**Skewness**	**Skewness**	**Kurtosis**	**Kurtosis**	**Kurtosis**
	**who htz**	**who wtz**	**who wfl**	**who htz**	**who wtz**	**who wfl**
adam/nord/ext-nord	0.114	-0.08	-0.156	3.093	2.978	3.732
centre/sud/est	0.318	-0.062	-0.16	4.137	3.61	3.454
nord-ouest/sud-ouest	0.459	0.053	-0.168	5.027	4.373	3.73
ouest/littoral	0.184	-0.12	-0.332	4.026	4.407	4.698
yaoundé/douala	0.21	-0.212	-0.121	4.171	5.062	3.94
Total	-0.003	-0.325	-0.287	3.719	3.595	3.718

**Cameroon 1998**	**Skewness**	**Skewness**	**Skewness**	**Kurtosis**	**Kurtosis**	**Kurtosis**
	**who htz**	**who wtz**	**who wfl**	**who htz**	**who wtz**	**who wfl**
central, south, & east	0.329	0.044	-0.218	3.139	2.987	3.663
north/ extreme north/ ad	0.196	0.22	0.218	3.419	3.851	3.808
northwest & southwest	0.437	-0.198	-0.519	3.374	3.165	3.649
west & littoral	0.389	-0.034	-0.097	4.571	3.448	3.807
Total	0.338	-0.02	-0.085	3.685	3.266	3.499

**Cameroon 2011**	**Skewness**	**Skewness**	**Skewness**	**Kurtosis**	**Kurtosis**	**Kurtosis**
	**who htz**	**who wtz**	**who wfl**	**who htz**	**who wtz**	**who wfl**
adamaoua	0.154	-0.008	0.208	3.182	3.089	3.254
centre	0.688	0.011	-0.331	4.45	4.135	3.83
douala	0.426	0.031	0.131	5.029	3.437	4.283
est	0.564	-0.253	-0.314	3.946	3.207	3.475
extrême-nord	0.596	0.282	0.18	3.916	3.6	3.904
littoral	0.57	-0.232	0.077	4.446	3.4	3.636
nord	0.76	-0.095	-0.221	4.58	4.247	3.541
nord-ouest	0.588	-0.2	-0.735	4.584	3.98	5.39
ouest	0.506	-0.085	-0.011	4.521	3.25	3.789
sud	-0.172	-0.31	-0.459	3.509	3.697	4.349
sud-ouest	0.768	-0.155	-0.226	5.027	4.871	3.569
yaoundé	0.356	-0.131	-0.05	4.608	5.052	3.965
Total	0.427	-0.202	-0.206	4.136	3.542	3.639

**Central African Rep 1994**	**Skewness**	**Skewness**	**Skewness**	**Kurtosis**	**Kurtosis**	**Kurtosis**
	**who htz**	**who wtz**	**who wfl**	**who htz**	**who wtz**	**who wfl**
bangui	-0.046	-0.27	0.221	3.067	3.392	4.129
rs i	0.296	-0.181	-0.102	3.775	3.382	3.999
rs ii	0.356	-0.195	-0.101	3.791	3.404	3.997
rs iii	0.16	-0.278	0.024	3.053	3.926	3.829
rs iv	0.037	-0.392	-0.429	3.17	3.463	3.585

rs v	0.701	0.066	-0.098	4.407	2.911	3.338
Total	0.256	-0.221	-0.053	3.586	3.435	3.861

**Chad 1996**	**Skewness who htz**	**Skewness who wtz**	**Skewness who wfl**	**Kurtosis who htz**	**Kurtosis who wtz**	**Kurtosis who wfl**
b.e.t.	0.449	0.216	0.7	3.166	2.307	3.722
batha	0.317	0.332	0.536	3.416	3.254	5.198
biltine	0.581	0.296	0.373	3.432	3.285	3.954
chari-baguirmi	0.38	0.096	0.001	3.581	3.351	3.498
guéra	0.274	0.071	0.04	2.946	3.73	3.69
kanem	0.516	0.326	0.058	3.036	2.866	3.447
lac	0.868	-0.027	0.066	3.987	2.903	3.407
logone occidental	0.652	0.078	-0.487	3.677	3.834	3.919
logone oriental	0.409	-0.14	-0.137	3.26	3.07	3.755
mayo-kebbi	0.523	0.35	-0.269	3.963	3.653	3.655
moyen chari	0.436	0.07	-0.102	3.288	3.739	3.861
n'djaména	0.09	-0.098	-0.072	3.144	3.668	3.948
ouaddaï	0.483	-0.134	-0.388	3.83	3.317	3.339
salamat	0.359	0.086	0.265	3.049	3.001	4.249
tandjilé	0.333	-0.37	-0.306	3.147	3.267	3.728
Total	0.402	0.022	-0.069	3.397	3.347	3.565

**Chad 2004**	**Skewness who htz**	**Skewness who wtz**	**Skewness who wfl**	**Kurtosis who htz**	**Kurtosis who wtz**	**Kurtosis who wfl**
b. e. t.	0.692	0.082	0.151	3.52	2.784	3.666
bar azoum	0.551	0.157	0.201	3.268	3.412	3.523
centre est	0.413	0.113	0.333	3.073	3.394	4.226
chari baguirmi	0.504	-0.011	-0.016	3.444	3.224	3.411
logone occidental	0.353	0.16	-0.015	2.98	3.55	3.419
mayo kebbi	0.627	-0.08	-0.442	3.663	3.363	3.393
moyen chari	0.237	-0.109	-0.237	3.304	3.756	3.446
n'djaména	0.381	-0.005	0.082	3.49	3.99	3.593
ouaddai est	0.565	0.178	0.464	3.746	3.836	3.997
Total	0.422	0.034	0.05	3.275	3.443	3.554

**Congo 2005**	**Skewness who htz**	**Skewness who wtz**	**Skewness who wfl**	**Kurtosis who htz**	**Kurtosis who wtz**	**Kurtosis who wfl**
brazzaville	0.161	-0.103	-0.153	3.692	4.931	3.588
nord	0.526	0.071	-0.018	3.874	3.688	3.527
pointe noire	0.565	-0.154	-0.242	4.227	4.165	3.718
sud	0.393	-0.166	-0.211	3.543	4.13	4.102
Total	0.386	-0.114	-0.161	3.792	4.196	3.765

**Congo 2012**	**Skewness who htz**	**Skewness who wtz**	**Skewness who wfl**	**Kurtosis who htz**	**Kurtosis who wtz**	**Kurtosis who wfl**
bouenza	0.248	0.03	-0.134	3.645	3.264	3.858
brazzaville	0.314	0.244	0.191	4.918	4.316	4.578
cuvette	0.231	-0.172	-0.183	3.757	4.03	4.345
cuvette - ouest	0.659	0.128	0.059	4.861	3.413	3.318
kouilou	0.331	-0.215	-0.404	3.931	3.652	4.161
lekoumou	0.315	0.436	0.24	3.913	4.784	3.832
likouala	-0.153	0.077	0.106	3.464	3.989	5.52
niari	0.539	-0.237	-0.287	4.529	3.192	4.525
plateaux	0.541	-0.246	-0.073	4.661	3.25	3.77
pointe-noire	0.005	0.106	-0.01	3.658	3.511	3.716
pool	0.504	0.099	0.061	4.814	3.912	3.62
sangha	-0.111	0.301	0.674	3.703	4.791	4.433
Total	0.285	0.027	0.03	4.15	3.832	4.268

**Congo DR 2007**	**Skewness who htz**	**Skewness who wtz**	**Skewness who wfl**	**Kurtosis who htz**	**Kurtosis who wtz**	**Kurtosis who wfl**
bandundu	0.518	0.36	0.436	3.529	4.318	4.462
bas-congo	0.884	0.223	0.326	4.338	3.478	3.825
equateur	0.426	0.015	-0.186	3.284	3.677	3.611
kasaï occident	0.709	-0.147	0.247	3.792	3.237	3.982
kasaï oriental	0.418	-0.034	-0.036	3.408	3.368	4.026
katanga	0.876	0.118	0.151	4.246	3.862	3.735
kinshasa	0.592	0.318	0.001	4.837	5.9	4.605
maniema	0.752	0.278	0.156	3.704	3.761	3.225
nord-kivu	0.564	0.144	-0.027	3.786	3.674	3.267
orientale	0.552	0.632	0.218	3.638	4.235	3.965

sud-kivu	0.644	0.489	0.03	3.361	3.661	3.711
Total	0.605	0.174	0.138	3.765	3.934	3.802

Côte d'Ivoire 1994	Skewness who htz	Skewness who wtz	Skewness who wfl	Kurtosis who htz	Kurtosis who wtz	Kurtosis who wfl
center	0.227	-0.158	0.057	3.359	3.332	3.337
center east	0.148	-0.44	0.14	3.338	3.215	3.327
center north	0.103	-0.407	-0.111	3.853	3.753	4.376
center west	0.388	-0.202	-0.156	3.727	3.735	3.337
north	0.126	0.029	-0.027	3.153	3.652	2.881
north east	0.027	-0.438	-0.191	2.782	3.268	3.531
north west	0.078	-0.024	-0.128	3.113	3.533	3.456
south	0.273	-0.113	-0.224	3.832	3.551	3.414
south west	-0.033	-0.15	0.113	3.205	4.024	2.735
west	0.432	-0.412	-0.765	4.156	3.988	4.014
Total	0.219	-0.243	-0.178	3.642	3.713	3.467

Côte d'Ivoire 1998	Skewness who htz	Skewness who wtz	Skewness who wfl	Kurtosis who htz	Kurtosis who wtz	Kurtosis who wfl
capital (abidjan)	0.375	-0.141	0.07	4.07	4.752	4.117
countryside	0.212	-0.146	-0.005	3.842	3.267	3.759
small city	0.15	-0.057	-0.168	4.227	4.202	4.01
Total	0.126	-0.137	0.001	3.982	3.866	4.005

Côte d'Ivoire 2011	Skewness who htz	Skewness who wtz	Skewness who wfl	Kurtosis who htz	Kurtosis who wtz	Kurtosis who wfl
Centre	0.342	-0.483	-0.168	3.856	4.684	3.685
Centre-Est	0.124	-0.022	0.231	3.234	3.163	3.568
Centre-Nord	0.569	-0.021	0.447	5.676	4.669	4.907
Centre-Ouest	0.14	-0.087	-0.331	3.96	3.702	3.644
Nord	0.513	-0.422	0.127	4.334	4.11	4.351
Nord-Ouest	0.324	-0.437	-0.236	3.757	3.954	4.149
Nord-est	0.45	-0.64	-0.446	4.046	4.448	4.018
Ouest	0.206	-0.223	-0.086	3.457	3.415	3.451
Sud sans Abidjan	0.642	0.484	0.436	4.605	4.597	3.737
Sud-ouest	0.214	0.127	-0.425	3.523	4.947	3.712
Ville d'Abidjan	0.346	-0.425	0.19	5.026	3.956	4.187
Total	0.364	-0.177	-0.052	4.192	4.338	4.048

Gabon 2000	Skewness who htz	Skewness who wtz	Skewness who wfl	Kurtosis who htz	Kurtosis who wtz	Kurtosis who wfl
east (haut-ogooué & ogooué-lolo)	0.192	-0.016	-0.149	4.021	4.122	4.168
libreville,port-gentil	0.559	0.132	-0.146	4.61	4.955	4.022
north (ogooué-ivindo & woleu-ntem)	0.57	-0.079	-0.43	5.298	4.646	4.582
south (ngounié, nyanga)	0.619	-0.12	-0.347	5.431	3.985	4.248
west (estuaire, moyen-ogooué & ogooué-maritime)	0.728	0.224	-0.634	5.295	4.654	4.665
Total	0.502	0.052	-0.293	4.778	4.539	4.279

Gabon 2012	Skewness who htz	Skewness who wtz	Skewness who wfl	Kurtosis who htz	Kurtosis who wtz	Kurtosis who wfl
estuaire	0.473	-0.144	-0.263	5.411	4.794	3.993
haut-ogooué	0.401	0.469	0.36	4.964	4.404	4.273
libreville-port-gentil	-0.034	0.011	-0.246	4.919	4.672	4.547
moyen-ogooué	0.58	-0.294	0.058	5.495	4.046	4.049
ngounié	0.275	-0.162	-0.403	4.606	3.776	4.749
nyanga	0.737	-0.489	-0.733	6.148	5.493	4.418
ogooué maritime	-0.137	-0.041	0.38	3.884	4.075	4.679
ogooué-ivindo	0.545	-0.092	-0.318	4.48	4.005	4.798
ogooué-lolo	0.89	0.32	-0.479	4.967	4.903	4.007
woleu-ntem	-0.039	0.586	0.181	4.62	5.388	3.636
Total	0.337	0.052	-0.077	4.704	4.573	4.563

Ghana 1993	Skewness who htz	Skewness who wtz	Skewness who wfl	Kurtosis who htz	Kurtosis who wtz	Kurtosis who wfl
ashanti	0.523	-0.49	-0.541	3.598	3.852	3.795
brong-ahafo	0.177	-0.352	-0.101	3.471	3.534	3.832
central	0.39	-0.333	-0.434	4.261	3.388	2.92
eastern	0.19	0.106	0.451	3.328	3.527	3.036
greater accra	-0.571	-1.024	-0.387	3.565	4.919	2.852
northern	0.818	0.062	0.077	4.918	2.702	3.221
upper east	-0.322	-0.487	-0.454	2.824	3.586	3.912

upper west	-0.136	1.148	-0.078	2.901	5.788	2.627
volta	0.084	0.271	-0.218	3.796	4.739	4.273
western	-0.03	-0.129	-0.185	3.283	2.861	2.625
Total	0.247	-0.216	-0.15	3.875	3.669	3.57

Ghana 1998	Skewness	Skewness	Skewness	Kurtosis	Kurtosis	Kurtosis
	who htz	who wtz	who wfl	who htz	who wtz	who wfl
ashanti region	0.378	-0.121	-0.096	3.606	4.305	4.303
brong ahafo region	0.184	-0.06	0.342	3.218	2.811	4.734
central region	-0.169	0.224	0.175	2.95	4.34	4.418
eastern region	0.485	0.115	0.225	4.214	3.397	4.053
greater accra region	0.059	0.245	0.436	2.936	3.202	5.261
northern region	0.383	0.257	-0.011	3.672	4.199	4.251
upper east region	0.647	0.346	-0.394	3.995	4.779	5.216
upper west region	0.525	0.08	-0.324	4.334	4.356	4.569
volta region	0.168	0.077	0.336	3.48	4.509	4.326
western region	0.183	0.129	0.343	3.353	3.244	4.202
Total	0.278	0.081	0.091	3.673	4.037	4.54

Ghana 2003	Skewness	Skewness	Skewness	Kurtosis	Kurtosis	Kurtosis
	who htz	who wtz	who wfl	who htz	who wtz	who wfl
ashanti	0.246	0.046	-0.125	3.893	3.763	3.978
brong ahafo	-0.148	-0.213	0.077	3.16	4.425	4.988
central	0.075	0.272	0.419	3.306	4.848	4.18
eastern	0.663	-0.603	-0.106	5.378	5.052	3.601
greater accra	0.386	-0.317	-0.294	4.431	6.232	4.078
northern	0.616	-0.179	-0.209	4.874	3.179	3.89
upper east	0.613	0.264	-0.005	3.858	4.503	3.974
upper west	0.562	0.48	0.075	4.098	3.859	2.853
volta	0.442	-0.218	-0.434	3.63	3.909	4.108
western	0.354	0.318	0.441	3.412	4.238	4.513
Total	0.361	-0.007	-0.075	3.943	4.29	4.04

Ghana 2008	Skewness	Skewness	Skewness	Kurtosis	Kurtosis	Kurtosis
	who htz	who wtz	who wfl	who htz	who wtz	who wfl
ashanti	0.296	0.31	0.007	3.295	4.143	3.945
brong ahafo	0.393	0.096	0.281	3.448	2.941	2.913
central	0.116	0.027	0.143	3.759	4.503	3.328
eastern	0.27	0.167	0.048	3.6	5.278	3.437
greater accra	0.235	0.716	0.524	4.984	4.038	4.824
northern	0.469	0.355	-0.007	3.54	4.159	4.018
upper east	0.922	0.708	-0.056	5.354	4.444	4.664
upper west	0.5	0.992	0.096	3.871	7.229	4.841
volta	0.493	0.518	0.305	4.178	3.698	4.914
western	0.487	0.463	-0.286	4.793	4.64	4.119
Total	0.393	0.436	0.112	4.043	4.657	4.208

Guinea 1999	Skewness	Skewness	Skewness	Kurtosis	Kurtosis	Kurtosis
	who htz	who wtz	who wfl	who htz	who wtz	who wfl
central guinea	0.538	0.234	0.087	3.942	4.233	4.146
conakry	0.454	-0.387	-0.058	4.411	4.211	4.375
forest guinea	0.479	0.132	-0.22	3.488	3.689	4.039
lower guinea	0.456	-0.176	-0.381	4.055	3.9	3.749
upper guinea	0.574	0.198	0.113	3.613	4.044	4.066
Total	0.48	0.039	-0.106	3.811	3.979	4.005

Guinea 2005	Skewness	Skewness	Skewness	Kurtosis	Kurtosis	Kurtosis
	who htz	who wtz	who wfl	who htz	who wtz	who wfl
boké	0.399	0.194	0.126	3.608	3.792	4.591
conakry	0.841	0.502	-0.108	4.485	4.096	3.698
faranah	0.444	-0.156	-0.21	3.796	2.878	3.998
kankan	0.588	0.11	0.167	3.862	3.784	3.755
kindia	0.806	0.137	-0.329	4.409	3.808	3.96
labé	0.46	0.218	0.291	3.538	3.855	3.854
mamou	0.355	0.059	0.525	3.567	3.679	4.944
n'zérékoré	0.323	-0.319	-0.337	3.468	3.281	3.609
Total	0.537	0.119	-0.01	3.928	3.875	4.041

Guinea 2012	Skewness	Skewness	Skewness	Kurtosis	Kurtosis	Kurtosis
	who htz	who wtz	who wfl	who htz	who wtz	who wfl
Bok?	0.576	0.736	0.307	3.819	4.63	3.862

Conakry	0.392	0.235	-0.118	3.689	3.975	4.568
Faranah	0.26	-0.186	-0.094	3.549	4.12	4.065
Kankan	0.316	0.13	-0.116	3.606	4.143	3.659
Kindia	0.596	0.145	-0.26	4.037	3.564	4.119
Lab?	0.294	0.05	-0.417	3.114	3.262	4.448
Mamou	0.407	0.541	-0.314	3.412	4.447	3.698
N'Z?r?kor?	0.396	-0.237	-0.589	3.554	3.349	4.208
Total	0.428	0.148	-0.224	3.68	3.995	4.036

**Liberia 2007**	**Skewness who htz**	**Skewness who wtz**	**Skewness who wfl**	**Kurtosis who htz**	**Kurtosis who wtz**	**Kurtosis who wfl**
monrovia	0.485	0.074	-0.168	4.181	3.949	3.941
north central	0.317	-0.214	-0.381	3.427	3.892	4.558
north western	0.632	0.158	0.201	4.202	3.583	4.395
south central	0.293	-0.41	-0.375	3.651	3.218	3.928
south eastern a	0.505	0.152	-0.323	3.696	3.963	4.315
south eastern b	0.648	-0.191	-0.305	3.935	3.924	3.712
Total	0.465	-0.095	-0.283	3.828	3.876	4.174

**Mali 1995**	**Skewness who htz**	**Skewness who wtz**	**Skewness who wfl**	**Kurtosis who htz**	**Kurtosis who wtz**	**Kurtosis who wfl**
bamako	0.334	-0.108	0.323	3.408	3.563	3.452
gao	0.138	-0.093	0.286	3.135	2.848	3.522
kayes	0.042	-0.261	-0.212	2.871	2.899	3.304
koulikoro	0.213	-0.25	-0.052	3.24	2.783	3.042
mopti	0.264	0.024	0.134	2.952	2.931	2.92
sikasso	0.147	-0.207	0.228	3.111	2.758	3.45
ségou	0.163	0	0.004	2.839	3.381	2.975
timbuktu	0.164	0.105	-0.151	3.962	3.202	2.422
Total	0.189	-0.118	0.057	3.193	3.091	3.182

**Mali 2001**	**Skewness who htz**	**Skewness who wtz**	**Skewness who wfl**	**Kurtosis who htz**	**Kurtosis who wtz**	**Kurtosis who wfl**
bamako	0.245	-0.162	-0.234	4.412	4.53	4.444
gao	0.338	0.001	-0.081	3.315	3.436	3.823
kayes	0.362	0	-0.01	3.464	3.18	3.969
kidal	0.251	-0.298	-0.535	3.755	3.431	3.279
koulikoro	0.411	-0.111	-0.1	3.746	3.627	3.632
mopti	0.365	-0.023	-0.105	3.44	3.312	3.855
segou	0.367	0.24	0.055	3.124	4.034	3.429
sikasso	0.629	0.063	-0.047	4.441	3.647	3.778
tombouctou	0.664	-0.131	0.273	3.644	3.071	3.719
Total	0.402	-0.011	-0.058	3.618	3.552	3.846

**Mali 2006**	**Skewness who htz**	**Skewness who wtz**	**Skewness who wfl**	**Kurtosis who htz**	**Kurtosis who wtz**	**Kurtosis who wfl**
bamako	0.344	0.15	0.252	4.039	4.106	4.195
gao	0.427	0.357	0.295	3.454	3.669	3.651
kayes	0.363	0.135	0.329	3.588	3.647	3.805
kidal	0.153	0.29	0.088	3.093	3.83	2.828
koulikoro	0.499	0.093	-0.076	3.859	3.73	3.706
mopti	0.447	0.059	0.068	3.124	3.533	3.811
segou	0.468	-0.111	0.097	3.773	3.311	3.877
sikasso	0.5	0.165	0.019	3.688	3.94	3.864
tombouctou	0.395	0.054	0.379	3.059	3	3.468
Total	0.417	0.125	0.14	3.512	3.727	3.8

**Niger 1992**	**Skewness who htz**	**Skewness who wtz**	**Skewness who wfl**	**Kurtosis who htz**	**Kurtosis who wtz**	**Kurtosis who wfl**
agadez	0.789	-0.032	0.191	5.534	3.502	3.852
diffa	0.223	0.792	0.679	3.298	4.281	4.207
dosso	0.425	0.223	0.075	4.231	4.59	5.201
maradi	0.373	0.178	-0.096	3.464	4.007	3.283
niamey	0.35	0.097	0.196	4.009	3.763	4.161
tahoua	0.621	0.186	0.107	3.918	3.938	4.091
tillabéri	0.467	-0.013	-0.193	3.836	3.678	3.315
zinder	0.534	0.148	-0.012	3.421	3.36	3.413
Total	0.398	0.074	0.055	3.725	3.8	3.922

**Niger 1998**	**Skewness who htz**	**Skewness who wtz**	**Skewness who wfl**	**Kurtosis who htz**	**Kurtosis who wtz**	**Kurtosis who wfl**

dosso	0.178	0.055	0.123	3.457	3.486	3.504
maradi	0.542	0.047	0.129	4.108	3.556	3.701
niamey	0.377	-0.129	0.145	3.93	3.935	3.648
tahoua/agadez	0.358	-0.008	-0.047	3.55	3.182	3.135
tillaberi	0.293	0.195	-0.005	3.667	3.594	3.524
zinda/diffa	0.378	0.151	0.074	3.144	3.174	3.026
Total	0.36	0.051	0.069	3.616	3.415	3.465

Niger 2006	Skewness	Skewness	Skewness	Kurtosis	Kurtosis	Kurtosis
	who htz	who wtz	who wfl	who htz	who wtz	who wfl
agadez	0.882	0.404	0.383	4.774	4.959	4.849
diffa	0.597	0.202	0.162	3.797	3.127	4.67
dosso	0.228	-0.213	-0.304	2.91	3.348	4.377
maradi	0.447	0.15	-0.106	3.027	3.072	3.683
niamey	0.059	-0.039	0.305	3.149	4.328	4.738
tahoua	0.439	0.094	0.077	3.67	3.487	4.376
tillabéri	0.223	0.068	0.375	3.303	3.591	4.393
zinder	0.976	0.113	0.351	4.884	3.731	3.505
Total	0.446	0.054	0.135	3.594	3.478	4.259

Niger 2012	Skewness	Skewness	Skewness	Kurtosis	Kurtosis	Kurtosis
	who htz	who wtz	who wfl	who htz	who wtz	who wfl
Agadez	-0.12	0.145	0.261	2.643	3.559	3.18
Diffa	0.583	0.702	0.375	3.961	3.834	3.196
Dosso	0.188	0.003	0.165	3.701	4.017	4.318
Maradi	0.768	0.145	0.223	4.244	3.251	3.772
Niamey	0.172	-0.212	0.269	5.132	5.043	4.865
Tahoua	0.577	0.039	0.097	4.264	3.532	4.011
Tillaberi	0.218	0.274	0.126	3.641	5.106	3.777
Zinder	0.507	0.063	-0.137	3.569	3.836	3.478
Total	0.433	0.075	0.171	4.002	3.615	4.04

Nigeria 1990	Skewness	Skewness	Skewness	Kurtosis	Kurtosis	Kurtosis
	who htz	who wtz	who wfl	who htz	who wtz	who wfl
northeast	0.6	-0.001	-0.277	3.698	3.133	3.553
northwest	0.544	0.241	-0.231	3.535	3.921	3.731
southeast	0.346	-0.221	-0.077	3.617	3.471	4.267
southwest	0.35	-0.068	-0.101	3.913	3.72	4.406
Total	0.387	-0.089	-0.231	3.607	3.525	4.077

Nigeria 2003	Skewness	Skewness	Skewness	Kurtosis	Kurtosis	Kurtosis
	who htz	who wtz	who wfl	who htz	who wtz	who wfl
north central	0.439	-0.2	-0.38	3.975	4.052	4.353
north east	0.711	0.128	0.047	4.286	3.698	4
north west	0.753	0.049	0.003	4.001	3.414	3.276
south east	0.242	0.017	-0.008	3.588	4.359	4.049
south south	0.285	0.148	-0.017	4.204	4.009	3.923
south west	0.073	0.08	-0.008	3.171	4.447	3.428
Total	0.421	-0.016	-0.04	3.634	3.753	3.797

Nigeria 2008	Skewness	Skewness	Skewness	Kurtosis	Kurtosis	Kurtosis
	who htz	who wtz	who wfl	who htz	who wtz	who wfl
north central	0.513	0.038	-0.237	3.504	3.8	3.603
north east	0.603	0.208	0.005	3.339	3.265	2.872
north west	0.721	0.228	0.008	3.349	3.288	2.819
south east	0.296	-0.021	0.071	3.705	4.325	3.993
south south	0.336	0.172	0.101	3.725	4.327	3.81
south west	0.541	0.114	-0.141	3.912	4.236	3.942
Total	0.509	0.032	-0.099	3.392	3.473	3.242

Sao Tome et Principe 2008	Skewness	Skewness	Skewness	Kurtosis	Kurtosis	Kurtosis
	who htz	who wtz	who wfl	who htz	who wtz	who wfl
região centro	0.842	-0.144	0.022	5.385	3.925	3.358
região do principe	0.689	0.2	0.26	4.475	4.334	3.805
região norte	0.651	-0.216	-0.24	4.499	4.262	3.237
região sul	0.702	-0.085	0.107	4.111	3.471	3.079
Total	0.656	-0.043	0.018	4.409	4.05	3.242

Senegal 1992	Skewness	Skewness	Skewness	Kurtosis	Kurtosis	Kurtosis
	who htz	who wtz	who wfl	who htz	who wtz	who wfl
central	0.123	-0.065	0.064	3.383	3.732	4.024

north east	0.186	-0.109	0.06	3.475	3.87	4.175
south	0.307	-0.068	-0.311	3.597	3.816	3.824
west	0.111	-0.038	-0.087	3.682	3.789	4.118
Total	0.111	-0.114	-0.046	3.494	3.796	4.064

**Senegal 2005**	**Skewness**	**Skewness**	**Skewness**	**Kurtosis**	**Kurtosis**	**Kurtosis**
	**who htz**	**who wtz**	**who wfl**	**who htz**	**who wtz**	**who wfl**
dakar	-0.177	0.116	-0.323	4.751	4.073	3.179
diourbel	0.06	0.167	0.101	3.834	2.982	3.626
fatick	0.459	0.063	-0.245	4.287	2.916	3.084
kaolack	0.141	-0.242	-0.215	3.944	4.852	3.812
kolda	0.276	-0.063	-0.145	4.408	3.648	4.128
louga	0.085	-0.14	0.203	4.494	4.129	4.511
matam	0.462	0.036	0.086	4.762	2.599	3.271
saint-louis	0.316	-0.088	-0.123	3.648	3.513	3.332
tambacounda	0.13	-0.353	0.655	4.16	3.444	5.093
thiès	0.375	-0.333	0.043	4.179	4.828	4.749
zuguinchor	0.714	0.323	-0.085	4.699	4.536	4.072
Total	0.191	-0.101	0.036	4.239	3.845	4.042

**Senegal 2010**	**Skewness**	**Skewness**	**Skewness**	**Kurtosis**	**Kurtosis**	**Kurtosis**
	**who htz**	**who wtz**	**who wfl**	**who htz**	**who wtz**	**who wfl**
dakar	0.117	0.669	0.855	4.277	5.125	4.66
diourbel	0.105	0.688	0.91	4.074	5.913	5.748
fatick	0.369	-0.133	-0.189	5.144	5.469	4.616
kaffrine	0.078	-0.245	0.726	3.19	3.324	4.94
kaolack	-0.022	0.565	0.289	3.651	4.972	5.702
kedougou	0.75	0.134	0.141	5.404	5.111	5.391
kolda	0.386	0.118	-0.029	3.924	4.361	3.679
louga	0.463	0.29	0.606	4.71	4.336	4.804
matam	0.151	-0.001	0.548	4.002	3.621	4.617
saint-louis	0.579	0.682	-0.207	5.085	5.473	3.23
sedhiou	0.225	-0.234	0.626	3.64	3.126	4.323
tambacounda	0.572	-0.111	0.122	3.906	3.777	3.975
thiès	0.32	0.079	-0.18	4.011	3.589	3.901
ziguinchor	-0.009	-0.641	0.152	4.768	5.039	4.891
Total	0.302	0.151	0.309	4.244	4.507	4.639

**Sierra Leone 2008**	**Skewness**	**Skewness**	**Skewness**	**Kurtosis**	**Kurtosis**	**Kurtosis**
	**who htz**	**who wtz**	**who wfl**	**who htz**	**who wtz**	**who wfl**
eastern	0.469	0.24	0.171	3.621	3.059	3.446
northern	0.46	0.104	0.144	3.315	3.374	4.196
southern	0.581	0.19	0.246	3.216	3.441	2.935
western	0.438	0.47	0.313	3.352	4.528	3.696
Total	0.478	0.238	0.235	3.406	3.523	3.579

**Togo 1998**	**Skewness**	**Skewness**	**Skewness**	**Kurtosis**	**Kurtosis**	**Kurtosis**
	**who htz**	**who wtz**	**who wfl**	**who htz**	**who wtz**	**who wfl**
centrale	0.236	-0.154	-0.105	3.401	3.396	3.266
kara	0.427	-0.191	0.025	3.976	3.841	3.206
lomé	0.661	-0.495	-0.163	5.245	4.427	4.385
marities	0.376	-0.069	0.345	4.254	4.042	4.502
plateaux	0.132	-0.175	-0.15	3.122	4.013	3.739
savanes	0.458	0.309	0.293	4.157	4.366	3.566
Total	0.326	-0.064	0.065	3.883	3.841	3.576

Skewness and Kurtosis for height-for-age (HAZ), weight-for-age (WTZ), weight-for-height (WHZ), and MUAC by region NNS

Benin 2008	Skewness	Skewness	Skewness	Skewness	Kurtosis	Kurtosis	Kurtosis	Kurtosis
	who htz	who wtz	who wfl	muac	who htz	who wtz	who wfl	muac
alibori	0.234	-0.301	-0.27	-0.067	3.798	3.117	3.994	2.79
atacora	0.196	-0.182	0.101	0.149	4.46	3.711	3.787	3.037
atlantique	-0.057	-0.12	-0.137	-0.009	3.684	3.283	2.819	3.34
borgou	0.379	-0.328	-0.62	-0.127	4.92	4.882	3.987	3.455
collines	0.02	-0.493	-0.45	0.133	3.191	3.536	4.854	2.877
couffo	0.011	-0.389	-0.201	-0.012	3.732	4.044	3.942	3.833
donga	0.191	-0.188	-0.215	-0.35	3.68	5.57	5.933	3.673
littoral	0.062	0.104	0.394	-0.184	3.231	3.539	4.369	4.861
mono	0.64	-0.572	-0.176	-0.021	6.355	3.869	3.687	3.359
oueme	0.359	0.386	0.25	0.375	3.849	3.333	3.647	3.281
plateau	0.035	-0.214	-0.198	0.007	3.618	3.736	3.517	2.785
zou	0.448	-0.102	0.021	-0.188	4.396	3.99	3.351	3.647
Total	0.194	-0.198	-0.112	-0.046	4.182	4.002	4.085	3.434

Burkina Faso 2012	Skewness	Skewness	Skewness	Skewness	Kurtosis	Kurtosis	Kurtosis	Kurtosis
	who htz	who wtz	who wfl	muac	who htz	who wtz	who wfl	muac
bales	-0.017	-0.073	-0.052	0.049	2.86	3.197	2.973	3.035
bam	0.299	-0.087	-0.003	0.037	4.102	3.3	3.209	3.219
banwa	-0.182	-0.411	-0.161	-0.444	3.355	3.64	3.663	4.347
banwa ganzourgou	0.218	-0.136	-0.065	-0.747	4.501	3.723	3.767	8.135
bazega	-0.033	-0.199	-0.298	-0.199	2.971	3.202	3.327	3.321
boulgou	0.016	-0.031	0.015	0.127	3.475	3.62	3.172	3.192
cascades	0.103	-0.314	-0.132	0.05	2.623	3.07	2.843	2.786
centre ouest	0.056	-0.275	-0.491	-0.108	3.693	3.5	3.657	3.058
est	0.035	-0.128	-0.068	-0.018	2.703	2.817	2.767	3.428
houet	0.108	-0.267	-0.131	-0.19	4.093	3.78	3.307	2.754
kadiogo	0.188	-0.134	0.066	0.193	4.438	3.142	2.761	2.736
kenedougou	-0.032	-0.164	-0.206	-0.014	3.26	3.377	3.1	2.879
kossi	-0.116	-0.383	-0.171	0.056	3.126	2.949	3.084	2.837
koulpelogo	0.131	-0.057	0.111	-0.048	3.367	3.077	3.926	3.007
kouritenga	-0.144	-0.055	0.108	0.022	3.203	3.34	3.89	2.937
kourweogo	0.234	0.076	-0.004	0.005	3.9	3.033	2.934	3.402
mouhoun	-0.095	-0.294	-0.108	-0.038	2.952	3.357	2.944	3.485
nahouri	0.09	-0.033	-0.164	-0.079	2.911	2.7	2.976	2.704
namentenga	0.187	-0.271	-0.074	0.053	3.63	3.21	3.411	3.433
nayala	0.164	-0.062	-0.066	0.04	4.077	3.206	2.932	3.016
nord	0.384	0.123	-0.067	0.102	3.981	3.243	3.08	2.876
oubritenga	-0.234	-0.385	-0.246	-0.253	3.011	3.618	3.452	3.644
sahel	0.511	-0.293	-0.16	-0.186	4.059	3.691	3.036	2.807
sanmentenga	0.114	-0.118	-0.115	-0.016	3.371	2.924	2.839	3.185
sourou	0.042	-0.229	-0.032	0.106	3.809	3.476	3.136	3.126
sud ouest	0.29	0.001	-0.159	0.004	3.41	3.114	3.017	3.075
tuy	-0.054	-0.248	-0.091	0.131	3.096	3.475	3.175	3.379
zoundweogo	-0.11	-0.339	-0.017	0.3	3.166	4.097	3.883	4.068
Total	0.092	-0.174	-0.086	-0.035	3.518	3.306	3.219	3.454

Cameroon 2011	Skewness	Skewness	Skewness	Skewness	Kurtosis	Kurtosis	Kurtosis	Kurtosis
	who htz	who wtz	who wfl	muac	who htz	who wtz	who wfl	muac
extrême-nord	0.355	-0.138	-0.391	-0.656	3.57	3.453	3.502	3.829
nord	0.385	-0.05	-0.042	-0.631	3.246	3.111	3.754	4.321
Total	0.359	-0.061	-0.063	-0.644	3.387	3.284	3.845	4.068

Central African Rep 2012	Skewness	Skewness	Skewness	Skewness	Kurtosis	Kurtosis	Kurtosis	Kurtosis
	who htz	who wtz	who wfl	muac	who htz	who wtz	who wfl	muac
bamingui bangoran	0.095	-0.394	-0.071	-0.255	3.866	3.791	3.74	3.17
bangui	0.121	-0.151	0.247	0.03	3.793	3.307	4.084	2.749
basse kotto	0.448	-0.239	-0.121	-0.222	4.468	3.43	4.213	3.788
haut kotto	0.063	-0.125	0.011	0.075	4.007	3.515	3.701	3.196
haut mbomou	0.542	0.008	0.055	0.087	4.378	3.222	4.235	2.861
kemo	0.065	-0.388	-0.269	-0.489	3.723	3.563	3.726	3.725
lobaye	0.249	0.106	0.397	0.091	4.165	3.347	4.788	3.014
mambere kadei	0.264	-0.114	-0.169	-0.183	3.772	3.509	3.63	3.928
mbomou	0.38	-0.317	-0.13	-0.309	4.941	3.724	3.745	4.452
nana grebizi	0.169	-0.31	-0.171	-0.184	3.732	3.462	3.382	3.568
nana mambere	0.199	-0.32	-0.117	-0.171	3.296	3.305	3.78	3.502

ombella mpoko	-0.028	-0.214	-0.039	-0.05	3.188	3.252	2.98	3.304
ouaka	0.406	-0.151	0.11	0.327	3.973	3.238	3.854	4.702
ouham	0.578	-0.079	-0.052	0.079	4.263	3.725	4.08	3.006
ouham pende	0.581	-0.154	0.243	-0.112	5.488	3.56	4.019	3.337
sangha mbarere	0.189	-0.113	-0.151	-0.148	3.445	3.42	3.879	3.808
vakaga	0.201	0.043	-0.28	0.258	3.615	3.271	3.586	3.419
Total	0.267	-0.187	-0.03	-0.093	3.985	3.441	3.843	3.58

**Chad June 2012**	**Skewness**	**Skewness**	**Skewness**	**Skewness**	**Kurtosis**	**Kurtosis**	**Kurtosis**	**Kurtosis**
	**who htz**	**who wtz**	**who wfl**	**muac**	**who htz**	**who wtz**	**who wfl**	**muac**
Barh El Ghazal	-0.274	-0.145	-0.172	-0.106	3.983	4.601	3.409	3.502
Batha	-0.125	-0.149	0.422	-0.345	3.515	3.889	4.953	3.417
Guéra	-0.012	-0.274	0.168	-0.291	3.833	3.724	4.186	3.415
Hadjer Lamis	-0.094	-0.302	-0.063	-0.396	3.469	3.6	3.371	3.355
Kanem	0.034	-0.02	0.283	0.067	3.209	2.778	3.122	3.474
Lac	-0.18	-0.305	0.241	-0.52	3.383	3.851	3.961	4.507
N’Djamena	0.142	-0.135	0.069	-0.397	3.775	3.331	3.277	3.653
Ouaddai	0.222	-0.151	-0.049	-0.091	3.9	3.698	3.641	3.48
Salamat	0.272	-0.17	0.218	-0.194	3.461	3.066	4.434	4.198
Sila	0.338	-0.027	0.089	-0.424	3.57	3.349	3.769	3.714
Wadi Fira	-0.04	-0.175	0.036	0.182	3.063	3.684	3.249	3.111
Total	0.107	-0.108	0.117	-0.225	3.609	3.546	3.748	3.629

**Chad (7 regions) Dec/Jan 2012-13**	**Skewness**	**Skewness**	**Skewness**	**Skewness**	**Kurtosis**	**Kurtosis**	**Kurtosis**	**Kurtosis**
	**who htz**	**who wtz**	**who wfl**	**muac**	**who htz**	**who wtz**	**who wfl**	**muac**
Logone Occidental	0.039	-0.284	-0.425	-0.149	3.171	3.082	3.356	3.218
Logone Oriental	0.069	-0.569	-0.33	-0.152	3.23	4.076	3.871	3.278
Mandoul	0.145	-0.662	-0.518	-0.131	4.317	4.739	4.317	3.412
Mayo-Kebbi Est	-0.153	-0.18	-0.242	-0.028	2.907	4.175	3.95	3.434
Mayo-Kebbi Ouest	0.15	-0.224	-0.399	-0.202	3.895	3.894	4.644	4.107
Moyen-Chari	-0.009	-0.392	-0.261	-0.222	3.23	4.149	4.693	3.147
Tandjilé	-0.125	-0.41	-0.396	-0.126	3.188	3.999	4.247	4.016
Total	0.043	-0.361	-0.363	-0.141	3.471	3.862	4.062	3.513

**The Gambia 2012**	**Skewness**	**Skewness**	**Skewness**	**Skewness**	**Kurtosis**	**Kurtosis**	**Kurtosis**	**Kurtosis**
	**who htz**	**who wtz**	**who wfl**	**muac**	**who htz**	**who wtz**	**who wfl**	**muac**
banjul	0.24	-0.191	0.13	0.003	3.932	3.441	3.989	3.09
basse	-0.07	-0.163	0.095	0.073	3.56	3.283	3.538	4.076
brikama	0.389	0.148	0.187	-0.155	4.771	3.968	3.728	3.505
janjanburay	-0.009	-0.077	-0.12	-0.17	3.468	3.258	4.339	3.28
kanifing	0.407	-0.151	0.106	-0.466	4.173	4.123	3.921	7.092
kerewan	0.002	-0.008	0.036	-0.231	3.849	3.268	4.149	4.194
kuntaur	-0.027	-0.169	-0.259	-0.258	3.437	3.317	3.439	3.535
mansakonko	0.246	0.037	0.043	-0.177	4.728	4.155	4.014	3.544
Total	0.1	-0.069	0.018	-0.164	3.964	3.548	3.918	3.92

**Guinea-Bissau 2008**	**Skewness**	**Skewness**	**Skewness**	**Skewness**	**Kurtosis**	**Kurtosis**	**Kurtosis**	**Kurtosis**
	**who htz**	**who wtz**	**who wfl**	**muac**	**who htz**	**who wtz**	**who wfl**	**muac**
Capitale	0.032	-0.031	0.118	-0.212	4.574	4.049	4.668	5.909
Est (Bafata e Gabu)	0.187	-0.166	-0.108	-0.621	4.14	4.029	3.588	4.458
Nord (Biombo, Cacheu e Oio)	0.452	0.179	-0.027	-0.626	5.023	4.322	3.765	4.158
Sud (Bolama, Quinara e Tombali)	0.762	0.196	0.225	-0.221	6.165	4.932	4.489	4.423
Total	0.377	0.074	0.032	-0.442	4.858	4.326	4.056	4.61

**Guinée Conakay 2012**	**Skewness**	**Skewness**	**Skewness**	**Skewness**	**Kurtosis**	**Kurtosis**	**Kurtosis**	**Kurtosis**
	**who htz**	**who wtz**	**who wfl**	**muac**	**who htz**	**who wtz**	**who wfl**	**muac**
boke nord	0.325	-0.054	0.41	-0.125	3.802	3.646	3.85	3.292
boke sud	0.374	-0.119	0.115	-0.104	4.846	4.08	4.018	3.404
conakry	0.279	0.017	0.132	-0.039	4.464	3.204	4.67	3.77
faranah	0.146	0.175	0.007	-0.026	3.696	4.098	4.675	3.204
kankan	0.214	-0.199	-0.184	0.004	4.044	4.594	4.453	3.12
kindia	-0.109	-0.333	-0.117	-0.054	3.436	3.547	4.294	2.86
labe	0.119	-0.313	-0.197	-0.418	4.066	4.148	4.18	6.21
mamou	0.119	-0.246	0.029	-0.379	4.224	4.599	3.863	6.081
nzerekore	0.179	-0.394	-0.174	-0.179	4.241	4.266	3.923	3.338
Total	0.2	-0.19	-0.003	-0.118	4.114	4.068	4.17	4.026

**Liberia 2010**	**Skewness**	**Skewness**	**Skewness**	**Skewness**	**Kurtosis**	**Kurtosis**	**Kurtosis**	**Kurtosis**
	**who htz**	**who wtz**	**who wfl**	**muac**	**who htz**	**who wtz**	**who wfl**	**muac**
bomi	0.814	-0.373	0.011	-0.448	4.767	3.348	3.65	3.497
bong	0.557	-0.018	-0.3	-0.242	4.655	3.754	3.385	3.042

gbarpolu	0.456	-0.241	-0.457	-0.107	4.174	3.87	4.325	3.157
grand bassa	0.176	-0.431	-0.127	-0.844	4.037	4.393	3.659	5.365
grand cape mount	0.621	-0.28	-0.273	-0.084	4.262	3.405	5.044	5.755
grand gedeh	0.797	-0.147	-0.371	-0.281	6.722	4.277	5.094	3.395
grand kru	0.275	-0.349	-0.217	-0.253	4.543	4.056	4.527	3.985
lofa	0.867	-0.026	-0.117	-0.183	5.519	3.221	4.888	3.507
margibi	0.043	-0.265	0.045	0.849	3.687	3.625	3.76	9.371
maryland	0.42	-0.105	-0.355	-0.22	3.591	3.151	3.972	3.392
montserrado	0.193	0.268	0.155	-0.205	3.763	3.596	5.089	2.803
nimba	0.239	0.009	-0.14	-0.079	4.434	3.108	4.144	3.337
river gee	0.479	-0.175	0.226	0.189	3.64	3.865	3.641	3.636
rivercess	0.21	-0.527	-0.363	-0.379	3.651	3.766	4.517	3.847
rural montserrado	1.105	0.158	-0.379	-0.139	5.727	3.718	4.275	2.467
sinoe	0.027	-0.353	-0.33	-0.253	3.33	3.887	3.732	3.309
Total	0.557	-0.146	-0.206	-0.157	4.648	3.76	4.297	4.221

**Liberia 2011**	**Skewness who htz**	**Skewness who wtz**	**Skewness who wfl**	**Skewness muac**	**Kurtosis who htz**	**Kurtosis who wtz**	**Kurtosis who wfl**	**Kurtosis muac**
North Central	1.138	0.06	-0.288	-0.185	6.871	3.675	3.639	3.581
North Western	-0.398	-0.948	-1.124	-0.507	3.187	4.633	4.88	3.981
South Central	0.388	0.043	0.083	-0.137	4.75	3.667	3.421	3.048
South Eastern	0.67	0.365	-0.391	0.479	4.891	6.008	2.998	4.678
Total	0.501	0.016	-0.098	-0.13	5.06	3.977	3.459	3.341

**Mali 2011**	**Skewness who htz**	**Skewness who wtz**	**Skewness who wfl**	**Skewness muac**	**Kurtosis who htz**	**Kurtosis who wtz**	**Kurtosis who wfl**	**Kurtosis muac**
bamako	-0.141	-0.024	0.302	1.06	3.206	3.268	3.862	9.486
gao	0.059	-0.267	0.009	0.048	2.901	3.166	3.481	3.241
kayes	0.111	-0.216	-0.067	0.053	3.366	3.762	3.122	3.026
kidal	0.171	-0.057	-0.001	0.99	2.681	3.167	2.608	7.186
koulikoro	0.043	-0.151	-0.187	0.082	3.191	3.042	3.374	6.339
mopti	0.078	-0.168	-0.138	0.036	2.989	2.842	3.303	3.258
segou	-0.018	-0.154	-0.347	0.102	3.247	3.244	3.436	2.898
sikasso	0.269	-0.348	-0.193	-0.137	3.873	3.659	3.889	3.437
tombouctou	0.126	-0.081	0.096	1.787	2.835	2.878	3.771	14.267
Total	0.064	-0.17	-0.06	0.406	3.122	3.31	3.45	5.859

**Mauritania 2006**	**Skewness who htz**	**Skewness who wtz**	**Skewness who wfl**	**Skewness muac**	**Kurtosis who htz**	**Kurtosis who wtz**	**Kurtosis who wfl**	**Kurtosis muac**
Centre	0.718	0.47	-0.316		5.471	5.396	4.113	
Fleuve	0.571	0.31	-0.167		4.715	4.873	4.016	
Nord	0.746	0.169	-0.242		5.675	4.179	3.907	
Nouakchott	0.619	0.104	-0.032		4.742	4.878	4.214	
SudEst	0.447	0.188	-0.253		4.677	4.494	4.25	
Total	0.597	0.192	-0.123		4.842	4.865	4.159	

**Mauritania March 2008**	**Skewness who htz**	**Skewness who wtz**	**Skewness who wfl**	**Skewness muac**	**Kurtosis who htz**	**Kurtosis who wtz**	**Kurtosis who wfl**	**Kurtosis muac**
Centre	0.639	-0.12	0.104		4.395	3.834	4.044	
Fleuve Nord	0.468	-0.127	0.055		4.537	3.262	3.754	
Fleuve Sud	0.459	-0.052	0.048		4.679	3.634	3.407	
Nord	0.402	-0.109	-0.387		3.384	2.811	3.962	
Nouakchott	0.1	-0.048	0.189		3.629	3.776	4.04	
SudEst	0.351	-0.078	-0.049		3.88	3.374	3.509	
Total	0.406	-0.029	0.078		4.022	3.478	3.725	

**Mauritania Dec 2008**	**Skewness who htz**	**Skewness who wtz**	**Skewness who wfl**	**Skewness muac**	**Kurtosis who htz**	**Kurtosis who wtz**	**Kurtosis who wfl**	**Kurtosis muac**
Centre	0.081	-0.096	-0.054	-0.452	2.648	3.043	3.517	4.099
Nord	0.012	-0.473	-0.166	-0.226	3.188	4.011	3.393	4.184
Nouakchott A	-0.14	-0.233	-0.264	-0.287	3.326	3.293	3.19	4.01
Nouakchott B	0.03	-0.086	-0.12	-0.452	2.75	2.775	3.068	4.55
Sud	0.083	-0.123	0.284	-0.269	3.4	3.551	3.768	3.641
SudEst	0.128	-0.03	-0.11	-0.266	2.803	3.031	3.428	3.812
Trarza	0.085	-0.077	-0.092	-0.457	3.087	2.932	2.827	3.765
Total	0.026	-0.158	-0.076	-0.275	3.027	3.175	3.188	3.953

**Mauritania 2009**	**Skewness who htz**	**Skewness who wtz**	**Skewness who wfl**	**Skewness muac**	**Kurtosis who htz**	**Kurtosis who wtz**	**Kurtosis who wfl**	**Kurtosis muac**
Centre	0.232	0.027	0.094		2.785	3.126	3.017	
Nord	0.012	-0.078	-0.146		2.72	2.873	3.511	

Nouakchott	-0.064	-0.128	-0.168		2.866	3.041	2.984	
Sud	0.138	-0.029	-0.152		2.823	2.876	3.687	
Sud-est	0.196	-0.261	0.142		2.876	3.233	3.405	
Trarza	0.063	-0.056	-0.252		2.884	2.918	3.442	
Total	0.093	-0.092	-0.083		2.82	2.981	3.257	

Mauritania July 2010	Skewness who htz	Skewness who wtz	Skewness who wfl	Skewness muac	Kurtosis who htz	Kurtosis who wtz	Kurtosis who wfl	Kurtosis muac
adrar/inchiri tiris	0.637	-0.036	-0.02		5.431	3.34	4.087	
assaba	-0.306	-0.624	-0.353		4.412	3.899	2.988	
brakna	0.124	-0.146	0.08		4.463	3.313	3.17	
gorgol	0.227	-0.069	0.059		3.634	3.205	3.117	
guidimakha	0.207	0.118	-0.093		3.512	3.294	3.449	
hodh chargui	0.321	0.063	0.105		5.133	3.832	3.783	
hodh gharbi	0.761	0.42	0.063		4.684	3.679	3.051	
nouadhibou	0.063	-0.263	-0.003		3.404	3.762	3.73	
nouakchott	0.318	-0.196	0.041		5.376	3.739	3.613	
tagant	0.375	0.545	0.53		3.247	6.045	4.493	
trarza	0.027	0.007	0.094		3.733	3.518	2.974	
Total	0.281	0.005	0.123		4.303	3.654	3.536	

Mauritania Dec 2010	Skewness who htz	Skewness who wtz	Skewness who wfl	Skewness muac	Kurtosis who htz	Kurtosis who wtz	Kurtosis who wfl	Kurtosis muac
adrar/inchiri tiris	0.234	0.077	0.031	0.219	4.346	2.811	2.714	3.378
assaba	0.216	-0.292	-0.056	0.276	4.892	3.626	3.642	2.694
brakna	0.247	-0.21	0.159	0.008	4.444	4.107	3.229	3.252
gorgol	-0.267	-0.188	0.136	-0.004	3.918	3.491	3.31	3.001
guidimakha	0.797	0.215	-0.17	0.097	5.806	4.114	4.539	3.469
hodh chargui	0.007	-0.095	0.001	-0.046	3.381	3.261	3.089	3.024
hodh gharbi	0.103	-0.331	0.042	-0.04	3.821	3.856	3.43	2.849
nouadhibou	0.29	0.012	0.17	0.206	4.76	4.908	3.205	2.882
nouakchott	0.168	-0.008	-0.154	0.307	3.599	3.454	3.85	4.007
tagant	0.404	0.068	0.107	0.15	5.293	3.534	3.291	3.444
trarza	0.247	0.35	0.117	-0.23	4.065	4.422	3.463	3.726
Total	0.259	-0.055	0.032	0.214	4.391	3.692	3.413	3.342

Mauritania July 2011	Skewness who htz	Skewness who wtz	Skewness who wfl	Skewness muac	Kurtosis who htz	Kurtosis who wtz	Kurtosis who wfl	Kurtosis muac
adrar/inchiri tiris	0.062	-0.344	-0.209	0.039	3.359	3.475	3.429	3.423
assaba	0.1	-0.14	0.005	0.174	3.389	2.905	2.953	3.118
brakna	-0.063	0.035	0.072	0.112	3.028	3.258	3.088	3.457
gorgol	0.167	-0.074	0.108	0.176	2.663	2.535	2.757	3.41
guidimakha	0.742	0.082	-0.025	-0.37	4.945	3.611	3.535	6.342
hodh chargui	0.48	0.32	0.052	0.157	5.547	5.727	3.215	3.369
hodh gharbi	0.319	-0.031	0.303	0.043	5.468	3.904	4.489	4.035
nouadhibou	0.03	-0.045	-0.136	0.084	2.764	3.432	3.433	2.932
nouakchott	0.311	-0.086	0.035	0.081	3.901	3.524	2.878	3.334
tagant	0.555	0.255	0.261	-0.09	4.151	3.303	3.879	3.001
trarza	0.185	0.18	0.316	0.216	5.119	4.844	4.254	3.204
Total	0.221	0.022	0.121	0.147	4.056	3.634	3.442	3.646

Mauritania Dec 2011	Skewness who htz	Skewness who wtz	Skewness who wfl	Skewness muac	Kurtosis who htz	Kurtosis who wtz	Kurtosis who wfl	Kurtosis muac
adrar/inchiri tiris	0.456	-0.144	-0.244	-0.089	5.259	4.108	3.863	3.295
assaba	-0.008	0.086	0.157	0.207	2.568	2.893	2.986	3.379
brakna	0.241	0.068	-0.085	0.236	3.402	2.715	3.128	2.93
gorgol	0.042	-0.289	-0.173	-0.199	3.027	2.885	3.792	3.823
guidimakha	-0.055	-0.154	0.091	-0.116	3.243	3.716	3.95	3.849
hodh chargui	0.036	-0.159	-0.182	-0.032	2.951	3.582	3.2	3.509
hodh gharbi	-0.167	-0.443	-0.082	0.054	3.021	3.537	3.172	4.11
nouadhibou	0.01	-0.043	-0.129	0.035	2.983	3.186	3.37	2.78
nouakchott	0.288	0.123	0.125	-0.011	3.472	3.362	3.666	3.303
tagant	0.11	-0.246	-0.075	0.013	2.985	3.404	3.58	3.453
trarza	0.138	-0.549	-0.041	0.835	5.223	7.268	4.178	11.989
Total	0.064	-0.176	-0.039	0.125	3.404	3.524	3.529	4.128

Mauritania July 2012	Skewness who htz	Skewness who wtz	Skewness who wfl	Skewness muac	Kurtosis who htz	Kurtosis who wtz	Kurtosis who wfl	Kurtosis muac
adrar/inchiri tiris	0.616	0.164	-0.037	-0.056	3.434	2.859	2.97	3.763
assaba	0.505	0.16	0.082	-0.54	4.199	3.398	2.923	5.457
brakna	0.005	-0.007	0.011	0.737	2.877	2.825	3.367	5.811

gorgol	0.007	0.038	0.186	0.265	3.358	2.914	3.023	3.267
guidimakha	0.384	0.208	-0.249	-0.136	3.981	3.675	3.43	3.411
hodh chargui	0.315	0.193	-0.027	0.36	3.432	4.21	3.235	3.47
hodh gharbi	0.522	0.255	0.222	-0.209	4.395	3.248	3.178	3.339
nouadhibou	0.411	-0.054	-0.138	-0.521	3.38	3.161	3.321	3.841
nouakchott	-0.003	0.102	0.114	-0.156	3.683	3.979	3.812	3.644
tagant	0.046	0.144	0.025	-0.32	2.525	2.838	3.013	2.793
trarza	0.124	0.021	0.089	0.151	4.478	3.635	3.432	3.525
Total	0.295	0.164	0.097	0.013	3.734	3.25	3.261	3.658

Niger 2012	Skewness who htz	Skewness who wtz	Skewness who wfl	Skewness muac	Kurtosis who htz	Kurtosis who wtz	Kurtosis who wfl	Kurtosis muac
agadez	0.365	0.018	-0.044		3.714	2.876	3.009	
diffa	0.315	0.012	0.024		4.272	3.249	3.553	
dosso	0.031	-0.278	-0.135		3.262	3.548	3.426	
maradi	0.274	0.013	0.001		3.545	3.437	3.209	
niamey	0.033	0.111	0.574		3.81	3.734	5.667	
tahoua	0.44	-0.02	0.204		4.277	3.561	4.19	
tillabéri	0.297	-0.078	-0.014		4.161	3.559	3.366	
zinder	0.289	-0.208	-0.271		3.792	3.4	3.426	
Total	0.217	-0.084	0.077		3.776	3.473	3.891	

Nigeria (Northern States) 2011	Skewness who htz	Skewness who wtz	Skewness who wfl	Skewness muac	Kurtosis who htz	Kurtosis who wtz	Kurtosis who wfl	Kurtosis muac
jigawa	0.51	-0.086	0.057	-0.252	4.56	3.645	3.897	3.455
kano	0.369	-0.017	0.168	-0.251	4.128	4.024	4.358	3.447
katsina	0.28	-0.297	-0.188	-0.346	3.888	3.328	3.633	3.474
kebbi	-0.097	-0.788	-0.408	-0.61	3.341	4.357	3.483	3.975
sokoto	-0.08	-0.596	-0.311	-0.389	3.552	3.905	3.704	3.595
yobe	0.084	-0.24	0.143	-0.246	3.745	4.139	3.55	3.938
zamfara	0.143	-0.624	-0.559	-0.554	3.596	5.178	3.734	3.323
Total	0.168	-0.395	-0.169	-0.403	3.79	4.064	3.796	3.665

Senegal 2012	Skewness who htz	Skewness who wtz	Skewness who wfl	Skewness muac	Kurtosis who htz	Kurtosis who wtz	Kurtosis who wfl	Kurtosis muac
dakar	0.217	-0.19	0.153	0.25	4.824	3.81	3.378	3.178
kolda	0.645	0.13	-0.296	-0.811	5.424	4.086	3.29	8.891
matam	0.208	-0.107	-0.03	0.006	3.627	3.198	3.387	3.076
myf	0.274	0.026	0.066	-0.031	3.527	3.255	3.048	3.593
sedhiou	0.02	-0.217	-0.05	-0.083	3.999	3.501	3.425	3.321
tambacounda	0.228	-0.204	-0.199	-0.082	4.085	3.384	3.254	3.362
velingara	0.125	-0.107	0.055	-0.088	3.576	3.496	3.413	3.387
Total	0.161	-0.113	-0.068	-0.086	4.05	3.458	3.339	3.752

Sierra Leone 2010	Skewness who htz	Skewness who wtz	Skewness who wfl	Skewness muac	Kurtosis who htz	Kurtosis who wtz	Kurtosis who wfl	Kurtosis muac
eastern	0.578	-0.144	-0.308	-0.342	4.602	3.754	3.655	3.423
northern	0.2	-0.205	-0.189	-0.198	4.074	3.861	3.168	3.271
southern	0.225	-0.166	-0.171	-0.334	3.712	3.397	3.144	3.181
western	0.56	-0.143	-0.194	-0.153	4.833	3.846	4.079	3.27
Total	0.436	-0.162	-0.231	-0.272	4.566	3.78	3.515	3.328

Togo June 2012	Skewness who htz	Skewness who wtz	Skewness who wfl	Skewness muac	Kurtosis who htz	Kurtosis who wtz	Kurtosis who wfl	Kurtosis muac
Centrale	0.174	-0.185	-0.035	-0.289	4.468	3.906	3.631	2.925
Kara	0.232	-0.099	0.006	0.057	3.97	3.399	3.973	2.813
Lomé	-0.061	-0.34	-0.115	0.015	3.767	3.975	3.447	3.936
Maritime	0.296	0.054	0.309	-0.023	3.695	3.699	4.488	4.545
Plateaux	0.225	-0.032	0.01	-0.954	3.661	3.393	3.364	9.593
Savanes	0.138	-0.062	0.058	0.226	3.695	3.257	3.673	3.138
Total	0.166	-0.095	0.056	-0.11	3.798	3.485	3.773	4.295

Togo Dec 2012	Skewness who htz	Skewness who wtz	Skewness who wfl	Skewness muac	Kurtosis who htz	Kurtosis who wtz	Kurtosis who wfl	Kurtosis muac
Kara	0.027	-0.228	-0.325	-0.221	3.687	3.21	3.807	3.956
Savanes	0.071	-0.352	-0.075	-0.194	3.234	3.524	3.886	3.372
Total	0.053	-0.297	-0.172	-0.182	3.468	3.409	3.759	3.651

Skewness and Kurtosis for height-for-age (HAZ), weight-for-age (WTZ), and weight-for-height (WHZ), by region MICS

Burkina Faso 2006	Skewness who htz	Skewness who wtz	Skewness who wfl	Kurtosis who htz	Kurtosis who wtz	Kurtosis who wfl
Boucle du Mouhoun	0.15	-0.081	0.023	3.48	2.748	2.586
Cascade	0.296	0.009	0.028	3.07	2.953	2.549
Centre	0.417	-0.165	-0.025	4.02	2.992	2.992
Centre-Est	0.352	-0.095	-0.171	3.39	2.714	2.857
Centre-Nord	0.533	-0.049	-0.225	3.84	3.189	2.618
Centre-Ouest	0.394	-0.005	0.053	3.91	3.366	3.015
Centre-Sud	0.437	-0.204	-0.088	3.28	3.723	2.841
Est	0.656	0.335	0.173	3.23	2.808	2.712
Hauts-Bassins	0.061	0.281	0.175	2.90	3.45	2.947
Nord	0.499	0.124	0.08	3.19	2.586	2.612
Plateau-Central	0.479	0.055	0.023	3.47	3.122	2.748
Sahel	0.559	0.302	0.221	3.53	2.922	2.723
Sud-Ouest	0.49	-0.234	-0.16	3.67	2.959	2.512
Total	0.417	-0.004	0.039	3.48	3.001	2.749

Cameroon 2006	Skewness who htz	Skewness who wtz	Skewness who wfl	Kurtosis who htz	Kurtosis who wtz	Kurtosis who wfl
Adamaoua	0.659	0.022	-0.252	4.61	3.734	3.974
Centre	0.447	0.126	0.077	3.60	3.797	3.816
Douala	0.344	-0.038	-0.336	3.61	3.596	3.809
Est	0.321	-0.209	-0.435	3.50	4.025	3.798
Extreme Nord	0.219	-0.154	-0.104	3.61	3.476	4.033
Littoral	0.347	-0.17	-0.288	3.89	3.55	4.409
Nord	0.488	0.202	0.274	3.67	3.108	3.018
Nord Ouest	0.904	-0.497	-0.497	4.75	4.018	3.597
Ouest	0.505	-0.005	0.107	3.87	3.915	4.082
Sud	0.399	-0.061	-0.079	3.55	4.576	3.66
Sud Ouest	0.42	-0.201	-0.015	3.87	3.483	3.368
Yaounde	0.506	-0.441	-0.238	4.26	4.526	4.154
Total	0.45	-0.138	-0.121	3.88	3.551	3.597

Central African Rep 2000	Skewness who htz	Skewness who wtz	Skewness who wfl	Kurtosis who htz	Kurtosis who wtz	Kurtosis who wfl
Bamingui-Bangoran	0.556	0.193	0.109	3.87	3.793	3.315
Bangui	0.404	-0.082	-0.109	3.67	4.099	3.363
Basse-Kotto	0.643	-0.044	-0.047	3.71	3.135	2.869
Haut-Mbomou	0.415	0.101	-0.131	3.46	4.129	3.487
Haute-Kotto	0.387	-0.126	-0.189	3.68	3.786	4.303
Kémo	0.528	-0.173	-0.241	3.67	4.005	3.646
Lobaye	0.564	0.048	-0.168	3.55	3.527	3.038
Mambéré-Kadeï	0.428	-0.091	0.015	3.45	3.292	3.789
Mbomou	0.548	0.125	-0.152	3.91	3.785	3.155
Nana-Grébizi	0.543	0.012	-0.379	4.11	4.232	3.802
Nana-Mambéré	0.604	0.067	-0.096	3.84	3.634	3.16
Ombella-M'poko	0.555	0.036	-0.165	3.87	3.685	3.693
Ouaka	0.691	-0.179	-0.221	3.79	3.702	2.968
Ouham	0.619	-0.071	-0.01	3.64	3.693	3.621
Ouham-Pendé	0.762	-0.116	-0.468	3.37	3.921	3.431
Sangha-Mbaéré	0.532	-0.055	0.005	3.73	4.005	4.185
Vakaga	0.665	0.161	0.116	3.74	3.565	3.659
Total	0.576	-0.037	-0.148	3.81	3.764	3.488

Central African Rep 2006	Skewness who htz	Skewness who wtz	Skewness who wfl	Kurtosis who htz	Kurtosis who wtz	Kurtosis who wfl
“Baminigui Bangoran”	0.283	0.234	-0.004	3.03	3.869	3.088
“Bangui”	0.539	-0.074	0.059	4.11	3.943	3.762
“Basse Kotto”	0.661	-0.027	-0.148	3.40	3.101	3.332
“Haut Mbomou”	0.444	0.328	-0.072	3.64	3.849	4.017
“Haute Kotto”	0.312	0.013	0.105	3.53	3.492	3.391
“Lobaye”	0.354	-0.089	-0.223	3.29	3.247	3.452
“Mambere Kadei”	0.757	0.327	-0.175	4.20	4.153	4.113
“Mbomou”	0.64	0.101	-0.389	3.71	4.125	3.969
“Nana Mambere”	0.658	0.222	-0.03	3.62	3.649	3.222
“Ombella Mpoko”	0.442	0.311	0.274	3.15	3.601	4.444
“Ouaka”	0.684	0.233	0.087	4.07	3.694	3.682

“Ouham Pende”	0.562	0.199	-0.063	3.41	3.425	3.226
“Ouham”	0.377	-0.164	0.188	3.35	2.833	3.847
“Sangha Mbaere”	0.519	0.015	-0.208	3.54	3.505	3.747
Total	0.514	0.125	-0.007	3.52	3.625	3.561

Central African Rep 2010	Skewness who htz	Skewness who wtz	Skewness who wfl	Kurtosis who htz	Kurtosis who wtz	Kurtosis who wfl
Baminigui Bangoran	0.163	-0.227	-0.016	3.96	4.344	3.691
Bangui	0.391	-0.075	0.014	4.12	3.581	3.918
Basse Kotto	0.252	-0.236	-0.165	3.22	3.801	3.687
Haut Mbomou	0.637	0.006	-0.161	4.26	3.626	3.037
Haute-Kotto	0.32	-0.103	0.154	3.40	3.057	3.646
Kémo	0.648	0.305	-0.058	4.30	3.892	3.582
Lobaye	0.292	-0.155	-0.076	3.30	3.339	3.863
Mambere Kadei	0.45	-0.168	-0.406	3.59	3.329	3.661
Mbomou	0.217	-0.268	-0.198	3.81	4.111	3.55
Nana Grebizi	0.3	-0.208	-0.06	3.93	3.812	3.562
Nana Mambéré	0.031	-0.396	-0.176	3.14	3.301	3.541
Ombella Mpoko	0.326	-0.051	-0.12	3.55	3.639	3.455
Ouaka	0.149	-0.549	-0.249	3.69	4.234	3.392
Ouham	0.241	-0.283	-0.3	3.17	3.073	3.096
Ouham Pende	0.255	-0.328	-0.219	2.70	3.142	3.436
Sangha Mbaere	0.247	0.08	0.021	3.04	3.327	3.435
Vakaga	0.096	-0.412	-0.388	2.75	3.715	3.261
Total	0.296	-0.165	-0.142	3.52	3.526	3.61

Chad 2000	Skewness who htz	Skewness who wtz	Skewness who wfl	Kurtosis who htz	Kurtosis who wtz	Kurtosis who wfl
Autres villes	0.242	-0.035	-0.026	3.37	3.607	3.726
N'Djaména	0.136	-0.309	-0.265	3.24	3.346	3.781
Rural	0.303	-0.066	-0.067	3.43	3.323	3.515
Total	0.246	-0.092	-0.073	3.37	3.438	3.656

Chad 2010	Skewness who htz	Skewness who wtz	Skewness who wfl	Kurtosis who htz	Kurtosis who wtz	Kurtosis who wfl
Barh El Gazal	0.571	0.093	0.058	3.38	3.015	3.389
Bhata	0.588	0.294	0.082	3.56	3.842	3.522
Chari Baguirmi	0.408	0.147	0.017	3.19	3.552	3.483
Guéra	0.561	0.112	-0.249	3.56	3.294	3.567
Hadjer Lamis	0.483	0.001	-0.232	3.08	3.401	2.956
Kanem	0.9	0.259	0.011	3.79	3.187	4.043
Lac	0.894	0.326	-0.168	3.97	3.451	3.575
Logone Occidental	0.124	-0.287	-0.089	2.97	3.561	3.9
Logone Oriental	0.251	-0.102	-0.518	3.80	3.44	3.246
Mandoul	0.36	-0.465	-0.36	3.74	3.741	3.784
Mayo Kebbi Est	0.736	-0.088	-0.465	4.15	3.518	3.386
Mayo Kebbi Ouest	0.438	-0.229	-0.528	3.54	2.994	3.407
Moyen Chari	0.554	-0.035	-0.579	3.98	4.039	3.683
Ndjaména	0.226	0.138	0.051	3.51	3.962	3.988
Ouaddai	0.482	0.179	-0.002	3.39	3.434	3.634
Salamat	0.635	0.502	0.064	3.35	4.03	3.72
Sila	0.357	0.378	0.113	2.63	3.72	3.303
Tandjilé	0.567	-0.033	-0.367	3.53	3.599	3.804
Wad Fira	0.449	0.266	0.061	3.27	3.774	4.091
bet	0.402	0.487	0.334	3.57	4.299	3.474
Total	0.502	0.079	-0.162	3.46	3.376	3.387

Congo DR 2001	Skewness who htz	Skewness who wtz	Skewness who wfl	Kurtosis who htz	Kurtosis who wtz	Kurtosis who wfl
Bandundu	0.443	-0.11	0.214	3.65	3.643	3.443
Bas-congo	0.483	-0.144	-0.158	3.57	3.491	3.723
Equateur	0.695	-0.202	-0.052	3.60	3.625	3.203
Kasai Occidental	0.505	0.057	0.052	3.13	3.305	2.962
Kasai Oriental	0.491	-0.074	0.14	3.55	3.699	3.265
Katanga	0.439	-0.008	0.046	3.35	3.613	3.214
Kinshasa	0.3	-0.293	0.089	3.50	3.719	3.768
Maniema	0.382	0.189	0.002	2.93	3.318	3.138
Nord-Kivu	0.806	0.044	-0.093	4.83	3.54	3.211
Orientale	0.579	0.018	0.084	3.88	3.875	3.387
Sud-Kivu	0.677	0	-0.185	3.84	3.215	3.129
Total	0.502	-0.087	0.041	3.50	3.535	3.315

Congo DR 2010	Skewness who htz	Skewness who wtz	Skewness who wfl	Kurtosis who htz	Kurtosis who wtz	Kurtosis who wfl
Bandundu	0.581	0.661	0.253	3.99	4.659	4.194
Bas congo	0.493	0.148	-0.269	3.84	3.8	3.367
Equateur	0.488	-0.127	-0.124	3.61	3.394	3.846
Kasai Occidental	0.609	-0.059	-0.119	4.15	3.22	3.851
Kasai Oriental	0.321	0.093	-0.289	3.55	3.799	4.418
Katanga	0.792	0.376	0.093	4.20	3.95	4.045
Kinshasa	0.361	-0.35	-0.161	4.34	3.848	4.079
Maniema	0.582	0.284	0.131	3.66	3.679	3.789
Nord Kivu	0.915	0.168	-0.204	4.77	3.931	4.052
Province Orientale	0.544	0.074	-0.114	4.01	3.619	3.683
Sud Kivu	0.715	-0.021	-0.13	4.82	3.503	3.811
Total	0.596	0.154	-0.021	4.05	3.859	4.01

Côte d’Ivoire 2006	Skewness who htz	Skewness who wtz	Skewness who wfl	Kurtosis who htz	Kurtosis who wtz	Kurtosis who wfl
Centre	0.624	0.166	0.004	5.05	4.702	3.927
Centre Est	0.371	-0.102	-0.388	3.47	3.35	4.016
Centre Nord	0.25	0.004	-0.034	3.55	3.442	2.923
Centre Ouest	0.69	-0.127	-0.236	4.06	3.817	3.593
Nord	0.314	-0.393	-0.226	3.51	3.649	3.414
Nord Est	0.333	-0.081	0.131	3.40	3.99	3.451
Nord Ouest	0.29	0.414	0.288	3.09	3.88	3.472
Ouest	0.449	-0.006	-0.009	3.69	4.161	3.716
Sud (sans ville d' Abidjan)	0.634	0.289	-0.056	4.64	4.277	3.618
Sud Ouest	0.656	0.08	-0.336	4.24	4.801	4.061
Ville Abidjan	0.262	-0.388	0.009	4.30	5.063	3.911
Total	0.452	0.033	-0.077	3.92	4.274	3.583

Equatorial Guinea 2000	Skewness who htz	Skewness who wtz	Skewness who wfl	Kurtosis who htz	Kurtosis who wtz	Kurtosis who wfl
Annobon	1.261	0.255	-1.22	3.42	2.281	3.307
Bioko Norte	0.291	-0.016	-0.385	3.33	3.802	3.794
Bioko Sur	0.662	-0.319	-0.179	3.28	2.202	2.246
Centro Sur	0.489	0.175	-0.129	3.77	4.135	2.991
Kie Ntem	0.54	0.206	-0.353	3.59	4.511	3.567
Litoral	0.472	0.014	-0.25	3.62	3.644	3.246
Wele Nzas	0.689	0.176	-0.1	3.88	4.083	3.992
Total	0.497	0.089	-0.271	3.63	4.024	3.544

Gambia 2000	Skewness who htz	Skewness who wtz	Skewness who wfl	Kurtosis who htz	Kurtosis who wtz	Kurtosis who wfl
Banjul	0.376	-0.386	0.156	4.38	4.637	3.88
Basse	0.772	-0.087	-0.155	5.04	3.831	3.403
Brikama	-0.011	-0.252	-0.201	4.28	3.556	3.231
Janjabureh	0.473	-0.093	0.136	3.72	3.438	3.166
Kanifing	0.457	-0.45	-0.344	5.67	4.857	4.193
Kerewan	0.364	-0.301	-0.158	3.72	4.447	4.115
Kuntaur	1.252	0.206	-0.213	6.51	4.806	3.544
Mansakonko	0.012	0.29	0.154	3.43	4.169	3.896
Total	0.477	-0.152	-0.043	4.62	4.036	3.756

Gambia 2005	Skewness who htz	Skewness who wtz	Skewness who wfl	Kurtosis who htz	Kurtosis who wtz	Kurtosis who wfl
Banjul	-0.49	-0.355	0.058	3.35	3.195	3.396
Basse	-0.014	-0.276	-0.016	3.31	3.877	4.196
Brikama	0.352	0.006	0.067	4.01	4.093	3.917
Janjanburay	-0.027	-0.118	-0.027	3.33	3.182	3.622
Kanifing	0.386	0.035	-0.064	4.51	3.717	3.295
Kerewan	0.08	0.124	0.093	3.59	4.356	3.862
Kuntaur	-0.184	-0.185	-0.19	3.39	3.821	3.598
Mansakonko	0.261	0.18	-0.103	3.56	4.292	4.227
Total	0.184	-0.046	0.021	3.92	3.918	3.96

Ghana 2006	Skewness who htz	Skewness who wtz	Skewness who wfl	Kurtosis who htz	Kurtosis who wtz	Kurtosis who wfl
Ashanti	0.151	-0.165	0.046	3.18	5.098	4.315
Brong Ahafo	-0.048	-0.51	-0.081	3.82	4.77	3.337
Central	0.571	-0.198	-0.085	4.30	4.123	3.243

Eastern	0.182	-0.052	0.039	4.11	3.854	5.964
Greater Accra	0.203	-0.609	-0.433	4.17	4.79	4.498
Northern	0.344	-0.273	-0.229	3.97	4.066	3.996
Upper East	0.148	0.356	-0.114	4.15	5.147	3.857
Upper West	0.666	-0.32	-0.263	4.50	3.899	3.645
Volta	0.42	-0.197	-0.45	4.24	4.043	3.418
Western	0.725	0.313	-0.075	4.96	3.887	4.116
Total	0.292	-0.177	-0.2	4.09	4.41	4.125

Ghana 2011	Skewness	Skewness	Skewness	Kurtosis	Kurtosis	Kurtosis
	who htz	who wtz	who wfl	who htz	who wtz	who wfl
Asante	0.283	-0.243	0.087	4.09	3.361	3.768
Brong Ahafo	-0.283	-0.198	-0.007	3.74	3.764	3.66
Central	0.224	-0.065	-0.052	4.74	3.747	4.063
Eastern	0.155	0.026	0.092	3.44	3.368	4.209
Greater Accra	-0.144	0.337	0.727	4.15	3.698	5.02
Northern	0.107	-0.113	-0.187	4.25	4.077	3.558
Upper East	0.137	-0.692	0.062	4.58	4.893	5.544
Upper West	0.223	0.366	0.072	4.10	4.867	4.301
Volta	0.115	-0.101	0.179	3.77	4.312	4.325
Western	0.074	0.143	0.146	4.57	3.904	4.281
Total	0.15	-0.079	0.036	4.17	4.161	4.209

Guinea Bissau 2000	Skewness	Skewness	Skewness	Kurtosis	Kurtosis	Kurtosis
	who htz	who wtz	who wfl	who htz	who wtz	who wfl
Bafatá	0.338	-0.12	0.091	3.82	3.841	3.88
Biombo	0.524	0.144	0.053	5.11	4.042	3.39
Bolama/Bijagós	0.48	0.543	-0.355	3.18	4.542	3.76
Cacheu	0.611	0.01	-0.146	4.48	3.934	3.866
Gabú	0.434	0.244	0.02	3.37	3.664	3.165
Oio	0.643	0.275	-0.167	4.43	4.496	3.465
Quinará	0.55	0.082	0.055	4.69	4.542	3.424
Tombali	0.5	-0.418	-0.206	3.97	4.781	3.911
sab	0.31	0.241	-0.087	4.14	3.877	3.577
Total	0.389	0.037	-0.078	4.00	4.084	3.616

Guinea Bissau 2006	Skewness	Skewness	Skewness	Kurtosis	Kurtosis	Kurtosis
	who htz	who wtz	who wfl	who htz	who wtz	who wfl
EST (Bafata e Gabu)	0.673	0.1	0.085	3.78	4.582	3.111
NORD (Biombo, Cacheu e Oio)	0.625	-0.296	-0.101	4.12	4.327	3.516
SAB Capital	0.648	-0.382	-0.433	4.62	4.609	4.213
SUD (Bolama, Quinara e Tombali)	0.715	-0.157	-0.259	3.95	4.187	3.101
Total	0.632	-0.151	-0.12	4.05	4.341	3.377

Mauritania 2007	Skewness	Skewness	Skewness	Kurtosis	Kurtosis	Kurtosis
	who htz	who wtz	who wfl	who htz	who wtz	who wfl
Adrar	0.525	0.102	0.244	3.44	3.718	4.229
Assaba	0.491	0.185	0.38	4.27	4.135	4.426
Brakna	0.363	0.152	0.053	3.36	4.261	3.946
Gorgol	0.354	0.158	0.164	3.19	3.359	4.141
Guidimagha	0.283	0.448	0.294	2.88	4.27	4.456
Hodh ECharghi	0.337	0.099	0.002	3.28	3.081	4.463
Hodh ELGharbi	0.501	-0.083	0.077	4.45	3.188	4.671
Inchiri	0.259	-0.053	-0.462	2.80	2.78	3.908
Nouadhibou	0.153	-0.326	-0.13	3.52	4.37	3.724
Nouakchott	0.436	-0.061	0.195	4.24	3.48	4.441
Tagant	0.411	0.016	0.573	3.52	3.31	4.894
Tiris Zemmour	0.467	-0.169	0.288	3.96	3.777	3.775
Trarza	0.35	-0.054	0.207	3.49	3.407	3.972
Total	0.362	0.034	0.14	3.61	3.589	4.141

Niger 2000	Skewness	Skewness	Skewness	Kurtosis	Kurtosis	Kurtosis
	who htz	who wtz	who wfl	who htz	who wtz	who wfl
Diffa	-0.058	-0.302	0.162	2.17	2.599	2.925
Agadez	0.279	-0.244	0.266	3.73	2.874	4.791
Dosso	0.214	-0.022	-0.413	3.55	3.552	3.886
Maradi	0.217	-0.245	-0.225	3.12	2.906	3.685
Niamey	0.149	0.009	-0.147	4.05	4.659	3.578
Tahoua	0.236	-0.323	0.148	3.26	3.274	4.366
Tillaberi	0.304	-0.342	-0.149	3.70	3.347	4.216
Zinder	0.87	0.117	-0.134	5.23	3.843	3.384

Total	0.254	-0.182	-0.111	3.56	3.443	3.974

Nigeria 2007	Skewness who htz	Skewness who wtz	Skewness who wfl	Kurtosis who htz	Kurtosis who wtz	Kurtosis who wfl
Abia	0.25	-0.019	0.081	3.50	3.593	3.992
Abuja FCT	0.413	0.352	-0.07	2.94	3.028	3.901
Adamawa	0.242	-0.366	-0.587	2.26	2.346	2.508
Akwa-Ibom	0.518	0.122	-0.005	4.11	4.191	4.171
Anambra	-0.085	-0.303	-0.159	3.05	4.346	4.713
Bauchi	0.605	0.149	-0.484	2.45	2.069	2.525
Bayelsa	0.239	-0.533	0.029	3.62	4.206	4.7
Benue	0.253	-0.132	-0.095	3.36	4.086	4.48
Borno	0.623	0.053	-0.095	2.95	2.36	2.659
Cross-Rivers	0.463	-0.014	-0.19	3.70	3.716	3.307
Delta	0.277	0.036	0.054	3.96	3.55	3.993
Ebonyi	0.548	0.099	0.049	3.80	3.41	3.626
Edo	0.462	-0.055	-0.009	3.43	4.102	4.579
Ekiti	0.569	0.024	-0.368	4.28	4.157	3.72
Enugu	0.132	0.026	0.193	3.66	4.361	4.673
Gombe	0.306	0.206	-0.225	2.41	2.579	2.033
Imo	0.295	-0.454	-0.191	2.99	3.771	3.389
Jigawa	0.829	0.494	-0.031	3.90	3.967	2.981
Kaduna	0.817	0.333	-0.174	4.04	3.7	2.797
Kano	1.093	0.506	-0.187	4.25	3.109	2.876
Katsina	0.773	0.28	0.175	2.71	3.673	2.778
Kebbi	0.793	0.434	-0.005	3.18	3.008	2.555
Kogi	0.302	-0.192	-0.059	2.79	3.707	3.598
Kwara	0.704	-0.082	0.028	3.89	3.629	3.607
Lagos	0.573	0.053	-0.178	3.34	4.362	3.736
Nasarawa	0.378	0.058	-0.19	3.25	3.315	2.934
Niger	0.412	-0.225	-0.075	3.29	3.606	2.744
Ogun	0.317	0.027	0.001	3.30	3.935	3.337
Ondo	0.479	0.245	0.026	3.51	3.645	4.22
Osun	0.9	0.296	0.108	5.09	5.421	4.262
Oyo	0.238	-0.122	0.127	3.79	3.561	3.541
Plataeu	0.358	0.199	-0.307	2.76	4.073	2.834
Rivers	0.056	-0.152	-0.082	3.39	3.425	3.933
Sokoto	1.136	0.344	-0.375	4.38	3.752	2.879
Taraba	0.712	0.204	-0.326	3.43	2.965	2.88
Yobe	0.569	0.248	-0.15	2.74	2.352	2.298
Zamfara	0.934	0.63	-0.033	4.33	3.72	3.385
Total	0.495	0.134	-0.118	3.17	3.349	3.155

Nigeria 2011	Skewness who htz	Skewness who wtz	Skewness who wfl	Kurtosis who htz	Kurtosis who wtz	Kurtosis who wfl
Abia	0.261	0.02	-0.129	4.25	4.263	3.54
Adamawa	0.392	-0.052	-0.204	4.02	3.615	3.572
Akwa ibom	0.342	0.262	0.286	3.79	4.109	4.423
Anambra	-0.247	-0.562	-0.212	3.86	4.588	4.266
Bauchi	0.411	0.12	-0.262	3.42	3.449	3.347
Bayelsa	-0.046	-0.127	-0.014	4.46	3.609	3.531
Benue	0.389	0.058	0.05	4.27	4.302	4.61
Borno	0.606	0.2	-0.099	4.05	3.972	3.821
Cross River	0.409	0.382	0.251	4.81	5.492	4.387
Delta	0.014	0.055	-0.048	3.17	4.08	3.757
Ebonyi	0.179	0.055	-0.238	3.58	3.723	4.617
Edo	0.097	-0.109	-0.019	3.48	3.709	4.234
Ekiti	0.341	-0.286	-0.131	4.38	4.036	3.693
Enugu	0.113	0.15	-0.034	3.84	2.956	3.908
FCT (Abuja)	-0.148	-0.387	-0.116	3.32	3.396	3.712
Gombe	0.672	0.186	-0.227	4.05	3.207	3.7
Imo	-0.134	-0.135	-0.2	4.19	3.988	3.496
Jigawa	0.851	0.503	-0.081	4.48	4.575	3.662
Kaduna	0.425	-0.078	-0.381	3.24	3.752	3.75
Kano	0.703	0.462	-0.056	4.02	3.935	3.594
Katsina	0.662	0.233	0.034	3.88	3.584	3.683
Kebbi	0.798	0.535	-0.002	3.87	3.887	3.538
Kogi	0.171	-0.103	0.042	3.76	3.414	4.062
Kwara	0.36	0.163	-0.041	3.77	4.562	3.906
Lagos	0.27	0.128	0.347	4.48	5.216	5.065
Nasarawa	0.055	-0.089	0.175	3.53	4.03	3.783

Niger	0.568	0.195	-0.1	3.51	3.815	3.409
Ogun	0.354	0.021	-0.187	4.77	3.62	4.13
Ondo	0.283	0.065	0.002	3.46	5.291	3.85
Osun	0.166	0.094	-0.372	4.00	4.358	4.241
Oyo	0.578	0.35	0.334	4.55	4.162	4.467
Plateau	0.329	-0.099	-0.167	4.03	3.812	3.135
Rivers	0.294	0.23	0.291	4.06	4.49	4.338
Sokoto	0.503	-0.063	-0.139	3.60	3.203	2.93
Taraba	0.585	-0.031	-0.374	3.49	4.087	4.333
Yobe	0.712	0.362	-0.193	4.16	3.657	3.674
Zamfara	0.803	0.151	-0.193	3.92	3.358	2.951
Total	0.275	0.007	-0.116	3.45	3.644	3.843

Sao Tome et Principe 2000	Skewness	Skewness	Skewness	Kurtosis	Kurtosis	Kurtosis
	who htz	who wtz	who wfl	who htz	who wtz	who wfl
Centro	0.331	-0.051	-0.117	4.23	4.849	4.032
Norte	0.076	0.093	0.282	3.86	4.025	3.871
Principe	0.067	-1.073	-0.264	2.93	7.041	3.948
Sul	1.403	-1.11	-0.408	8.88	5.515	4.929
Total	0.285	-0.107	-0.052	4.26	4.73	4.073

Senegal 2000	Skewness	Skewness	Skewness	Kurtosis	Kurtosis	Kurtosis
	who htz	who wtz	who wfl	who htz	who wtz	who wfl
Dakar	0.396	-0.165	0.071	4.05	3.961	3.958
Diourbel	0.195	-0.119	0.191	3.65	3.623	3.562
Fatick	0.416	-0.057	-0.16	3.64	3.907	3.984
Kaolack	0.205	-0.035	-0.257	3.34	3.731	3.523
Kolda	0.404	0.209	-0.325	3.57	3.616	3.609
Louga	0.187	-0.018	0.047	4.00	4.284	4.443
Saint louis	0.744	0.078	-0.142	4.86	4.604	3.3
Tambacounda	0.404	-0.01	-0.042	3.37	3.253	3.934
Thies	0.223	-0.247	-0.009	4.35	3.555	4.286
Ziguinchor	0.226	0.188	0.056	3.64	3.467	4.923
Total	0.275	-0.068	-0.036	3.86	3.722	4.004

Sierra Leone 2000	Skewness	Skewness	Skewness	Kurtosis	Kurtosis	Kurtosis
	who htz	who wtz	who wfl	who htz	who wtz	who wfl
East	0.69	0.114	-0.175	3.59	3.328	3.728
North	0.558	0.195	-0.036	3.53	4.041	4.165
South	0.377	0.072	-0.131	2.79	3.543	3.424
West	0.39	-0.086	-0.174	3.78	3.29	3.965
Total	0.482	0.059	-0.065	3.47	3.662	3.939

Sierra Leone 2005	Skewness	Skewness	Skewness	Kurtosis	Kurtosis	Kurtosis
	who htz	who wtz	who wfl	who htz	who wtz	who wfl
East	0.367	-0.11	-0.155	2.99	3.406	3.673
North	0.616	0.224	-0.184	3.88	4.024	3.928
South	0.54	-0.17	-0.131	3.74	3.836	3.733
West	0.401	0.045	-0.003	3.83	3.625	3.648
Total	0.504	0.01	-0.14	3.56	3.748	3.79

Sierra Leone 2010	Skewness	Skewness	Skewness	Kurtosis	Kurtosis	Kurtosis
	who htz	who wtz	who wfl	who htz	who wtz	who wfl
East	0.538	-0.243	-0.014	4.37	4.014	4.29
North	0.486	0.053	-0.004	3.70	3.833	3.551
South	0.346	0.033	0.122	3.44	4.271	3.467
West	0.331	-0.326	0.155	3.59	3.764	3.16
Total	0.434	-0.057	0.072	3.73	3.961	3.643

Togo 2006	Skewness	Skewness	Skewness	Kurtosis	Kurtosis	Kurtosis
	who htz	who wtz	who wfl	who htz	who wtz	who wfl
Centrale	0.377	0.135	-0.198	4.37	4.091	4.298
Kara	0.265	0.212	0.066	2.96	3.888	2.781
Lomé commune	-0.39	-0.501	-0.115	4.51	3.894	4.487
Maritime (sans Lomé commune)	0.243	-0.05	-0.088	3.78	4.009	3.79
Plateaux	0.246	-0.217	-0.058	3.38	3.909	3.622
Savanes	0.493	0.164	-0.06	4.04	3.585	2.969
Total	0.219	-0.074	-0.072	3.66	3.52	3.369

Togo 2010	Skewness	Skewness	Skewness	Kurtosis	Kurtosis	Kurtosis
	who htz	who wtz	who wfl	who htz	who wtz	who wfl

Centrale	0.349	0.066	-0.152	4.45	4.02	3.38
Kara	0.433	-0.424	-0.282	5.08	4.057	3.825
Lomé	0.578	0.137	-0.046	5.29	3.581	3.554
Maritime	0.028	-0.138	-0.115	4.24	4.54	4.083
Plateaux	0.178	-0.313	-0.114	4.21	4.439	3.281
Savanes	0.132	-0.16	-0.163	3.72	3.674	3.719
Total	0.237	-0.18	-0.144	4.36	3.969	3.619

Appendix I

Skewness and Kurtosis for height-for-age (HAZ), weight-for-age (WTZ), and weight-for-height (WHZ), by region DHS

Benin 2001	Skewness	Skewness	Skewness	Kurtosis	Kurtosis	Kurtosis
	who htz	who wtz	who wfl	who htz	who wtz	who wfl
atacora	0.389	-0.136	-0.239	3.863	3.832	3.865
atlantique	0.248	-0.182	-0.203	3.347	3.636	4.24
borgou	0.451	0.077	0.026	3.417	4.011	3.857
mono	0.833	-0.323	-0.497	5.835	3.553	5.303
oueme	0.467	0.017	-0.247	4.211	4.305	4.849
zou	0.424	-0.216	-0.375	4.288	3.778	3.811
Total	0.425	-0.138	-0.254	4.011	3.881	4.194

Benin 2006	Skewness	Skewness	Skewness	Kurtosis	Kurtosis	Kurtosis
	who htz	who wtz	who wfl	who htz	who wtz	who wfl
alibori	0.486	0.082	-0.231	3.009	3.787	2.67
atacora	0.467	0.361	0.077	3.675	4.1	3.894
atlantique	0.551	0.247	-0.019	4.448	4.612	3.616
borgou	0.454	0.282	-0.157	3.302	4.716	3.437
collines	0.471	-0.015	0.192	4.278	4.334	3.906
couffo	0.225	0.104	0.067	3.793	3.55	4.222
donga	0.604	0.104	-0.05	3.386	4.396	3.116
littoral	0.048	0.242	0.137	4.546	4.665	3.735
mono	0.178	-0.085	0.03	3.441	3.503	3.049
plateau	0.809	0.266	-0.098	4.276	4.937	3.368
quémé	0.538	0.099	0.138	4.35	4.221	4.208
zou	0.206	-0.002	0.227	3.151	3.889	2.539
Total	0.377	0.093	0.128	3.805	4.259	3.513

Benin 2011	Skewness	Skewness	Skewness	Kurtosis	Kurtosis	Kurtosis
	who htz	who wtz	who wfl	who htz	who wtz	who wfl
Alibori	0.809	0.2	0.047	3.277	3.144	2.323
Atacora	0.563	0.332	0.124	2.824	3.728	2.481
Atlantique	0.608	0.202	0.036	3.067	2.765	2.333
Borgou	0.437	0.225	-0.087	2.704	3.335	2.49
Collines	0.565	0.392	-0.278	2.924	3.597	2.453
Couffo	0.455	0.206	-0.007	3.374	3.459	2.911
Donga	0.588	0.056	-0.145	2.945	3.698	2.295
Littoral	0.492	0.224	-0.085	2.983	3.022	2.666
Mono	0.201	0.3	0.136	2.939	3.748	3.026
Ou?m?	0.533	0.35	-0.016	3.174	3.964	2.681
Plateau	0.869	0.008	-0.259	3.503	2.428	2.231
Zou	0.459	0.272	0.094	2.962	3.723	2.487
Total	0.518	0.261	-0.013	2.983	3.473	2.541

Burkina Faso 1993	Skewness	Skewness	Skewness	Kurtosis	Kurtosis	Kurtosis
	who htz	who wtz	who wfl	who htz	who wtz	who wfl
central/south	0.523	0.025	-0.178	4.137	3.685	3.549
east	0.328	0.127	0.195	3.076	3.793	3.705
north	0.355	-0.17	0.004	3.161	3.6	4.464
ouagadougou	0.263	-0.065	-0.072	3.874	3.769	4.003
west	0.036	-0.128	-0.262	2.958	3.755	3.398
Total	0.273	-0.066	-0.075	3.41	3.721	3.831

Burkina Faso 1998	Skewness	Skewness	Skewness	Kurtosis	Kurtosis	Kurtosis
	who htz	who wtz	who wfl	who htz	who wtz	who wfl
central/south	0.43	0.013	-0.078	3.884	3.656	3.384
east	0.313	-0.186	-0.17	3.469	3.175	3.747
north	0.711	-0.197	-0.092	3.714	3.256	4.082
ouagadougou	0.391	-0.284	-0.124	4.187	3.323	3.412
west	0.313	-0.174	-0.322	3.492	3.049	3.423
Total	0.391	-0.172	-0.178	3.659	3.291	3.614

Burkina Faso 2003	Skewness	Skewness	Skewness	Kurtosis	Kurtosis	Kurtosis
	who htz	who wtz	who wfl	who htz	who wtz	who wfl
boucle de mouhoun	0.496	-0.108	-0.034	4.004	3.194	2.76
cascades	0.502	0.181	-0.037	3.908	3.083	2.686

centre (sans ouagadougou)	0.412	0.382	-0.316	3.933	5.004	3.401
centre-est	0.705	0.075	0.036	3.89	3.693	3.097
centre-nord	0.506	-0.064	0.247	3.613	3.629	3.299
centre-ouest	0.404	0.042	0.034	3.651	3.826	3.679
centre-sud	0.322	0.398	0.005	3.061	3.866	3.061
est	0.853	0.207	-0.179	3.904	3.748	2.614
hauts bassins	0.296	-0.399	-0.197	3.627	3.7	3.82
nord	0.449	-0.123	-0.012	3.399	3.1	3.291
ouagadougou	0.473	-0.238	0.019	4.027	3.266	3.7
plateau central	0.416	0.176	0.15	3.272	2.997	2.92
sahel	0.464	0.12	-0.225	3.19	3.17	2.89
sud-ouest	0.45	-0.028	-0.142	3.266	3.326	3.145
Total	0.435	0.017	0.093	3.48	3.495	3.28

**Burkina Faso 2010**	**Skewness**	**Skewness**	**Skewness**	**Kurtosis**	**Kurtosis**	**Kurtosis**
	**who htz**	**who wtz**	**who wfl**	**who htz**	**who wtz**	**who wfl**
boucle de mouhoun	-0.04	-0.246	-0.217	3.736	3.551	3.851
cascades	0.6	-0.056	-0.109	4.51	3.32	3.547
centre	0.386	-0.39	0.075	5.053	3.307	4.028
centre-est	0.423	-0.28	-0.17	3.93	3.608	3.49
centre-nord	0.07	-0.077	0.231	3.115	3.299	3.461
centre-ouest	0.185	-0.365	-0.248	3.832	3.563	3.537
centre-sud	0.424	0.555	0.053	3.875	4.01	3.683
est	0.427	0.26	0.036	3.496	3.969	3.829
hauts basins	0.498	-0.032	-0.253	4.336	3.523	3.385
nord	0.263	-0.168	-0.259	3.13	3.02	3.934
plateau central	0.497	0.167	0.071	4.636	4.034	3.458
sahel	0.218	-0.146	-0.061	3.89	4.053	3.277
sud-ouest	0.461	0.314	0.003	4.183	5.046	3.681
Total	0.364	-0.049	-0.047	3.989	3.908	3.768

**Cameroon 1991**	**Skewness**	**Skewness**	**Skewness**	**Kurtosis**	**Kurtosis**	**Kurtosis**
	**who htz**	**who wtz**	**who wfl**	**who htz**	**who wtz**	**who wfl**
adam/nord/ext-nord	0.114	-0.08	-0.156	3.093	2.978	3.732
centre/sud/est	0.318	-0.062	-0.16	4.137	3.61	3.454
nord-ouest/sud-ouest	0.459	0.053	-0.168	5.027	4.373	3.73
ouest/littoral	0.184	-0.12	-0.332	4.026	4.407	4.698
yaoundé/douala	0.21	-0.212	-0.121	4.171	5.062	3.94
Total	-0.003	-0.325	-0.287	3.719	3.595	3.718

**Cameroon 1998**	**Skewness**	**Skewness**	**Skewness**	**Kurtosis**	**Kurtosis**	**Kurtosis**
	**who htz**	**who wtz**	**who wfl**	**who htz**	**who wtz**	**who wfl**
central, south, & east	0.329	0.044	-0.218	3.139	2.987	3.663
north/ extreme north/ ad	0.196	0.22	0.218	3.419	3.851	3.808
northwest & southwest	0.437	-0.198	-0.519	3.374	3.165	3.649
west & littoral	0.389	-0.034	-0.097	4.571	3.448	3.807
Total	0.338	-0.02	-0.085	3.685	3.266	3.499

**Cameroon 2011**	**Skewness**	**Skewness**	**Skewness**	**Kurtosis**	**Kurtosis**	**Kurtosis**
	**who htz**	**who wtz**	**who wfl**	**who htz**	**who wtz**	**who wfl**
adamaoua	0.154	-0.008	0.208	3.182	3.089	3.254
centre	0.688	0.011	-0.331	4.45	4.135	3.83
douala	0.426	0.031	0.131	5.029	3.437	4.283
est	0.564	-0.253	-0.314	3.946	3.207	3.475
extrême-nord	0.596	0.282	0.18	3.916	3.6	3.904
littoral	0.57	-0.232	0.077	4.446	3.4	3.636
nord	0.76	-0.095	-0.221	4.58	4.247	3.541
nord-ouest	0.588	-0.2	-0.735	4.584	3.98	5.39
ouest	0.506	-0.085	-0.011	4.521	3.25	3.789
sud	-0.172	-0.31	-0.459	3.509	3.697	4.349
sud-ouest	0.768	-0.155	-0.226	5.027	4.871	3.569
yaoundé	0.356	-0.131	-0.05	4.608	5.052	3.965
Total	0.427	-0.202	-0.206	4.136	3.542	3.639

**Central African Rep 1994**	**Skewness**	**Skewness**	**Skewness**	**Kurtosis**	**Kurtosis**	**Kurtosis**
	**who htz**	**who wtz**	**who wfl**	**who htz**	**who wtz**	**who wfl**
bangui	-0.046	-0.27	0.221	3.067	3.392	4.129
rs i	0.296	-0.181	-0.102	3.775	3.382	3.999
rs ii	0.356	-0.195	-0.101	3.791	3.404	3.997
rs iii	0.16	-0.278	0.024	3.053	3.926	3.829
rs iv	0.037	-0.392	-0.429	3.17	3.463	3.585

rs v	0.701	0.066	-0.098	4.407	2.911	3.338
Total	0.256	-0.221	-0.053	3.586	3.435	3.861

Chad 1996	Skewness who htz	Skewness who wtz	Skewness who wfl	Kurtosis who htz	Kurtosis who wtz	Kurtosis who wfl
b.e.t.	0.449	0.216	0.7	3.166	2.307	3.722
batha	0.317	0.332	0.536	3.416	3.254	5.198
biltine	0.581	0.296	0.373	3.432	3.285	3.954
chari-baguirmi	0.38	0.096	0.001	3.581	3.351	3.498
guéra	0.274	0.071	0.04	2.946	3.73	3.69
kanem	0.516	0.326	0.058	3.036	2.866	3.447
lac	0.868	-0.027	0.066	3.987	2.903	3.407
logone occidental	0.652	0.078	-0.487	3.677	3.834	3.919
logone oriental	0.409	-0.14	-0.137	3.26	3.07	3.755
mayo-kebbi	0.523	0.35	-0.269	3.963	3.653	3.655
moyen chari	0.436	0.07	-0.102	3.288	3.739	3.861
n'djaména	0.09	-0.098	-0.072	3.144	3.668	3.948
ouaddaï	0.483	-0.134	-0.388	3.83	3.317	3.339
salamat	0.359	0.086	0.265	3.049	3.001	4.249
tandjilé	0.333	-0.37	-0.306	3.147	3.267	3.728
Total	0.402	0.022	-0.069	3.397	3.347	3.565

Chad 2004	Skewness who htz	Skewness who wtz	Skewness who wfl	Kurtosis who htz	Kurtosis who wtz	Kurtosis who wfl
b. e. t.	0.692	0.082	0.151	3.52	2.784	3.666
bar azoum	0.551	0.157	0.201	3.268	3.412	3.523
centre est	0.413	0.113	0.333	3.073	3.394	4.226
chari baguirmi	0.504	-0.011	-0.016	3.444	3.224	3.411
logone occidental	0.353	0.16	-0.015	2.98	3.55	3.419
mayo kebbi	0.627	-0.08	-0.442	3.663	3.363	3.393
moyen chari	0.237	-0.109	-0.237	3.304	3.756	3.446
n'djaména	0.381	-0.005	0.082	3.49	3.99	3.593
ouaddai est	0.565	0.178	0.464	3.746	3.836	3.997
Total	0.422	0.034	0.05	3.275	3.443	3.554

Congo 2005	Skewness who htz	Skewness who wtz	Skewness who wfl	Kurtosis who htz	Kurtosis who wtz	Kurtosis who wfl
brazzaville	0.161	-0.103	-0.153	3.692	4.931	3.588
nord	0.526	0.071	-0.018	3.874	3.688	3.527
pointe noire	0.565	-0.154	-0.242	4.227	4.165	3.718
sud	0.393	-0.166	-0.211	3.543	4.13	4.102
Total	0.386	-0.114	-0.161	3.792	4.196	3.765

Congo 2012	Skewness who htz	Skewness who wtz	Skewness who wfl	Kurtosis who htz	Kurtosis who wtz	Kurtosis who wfl
bouenza	0.248	0.03	-0.134	3.645	3.264	3.858
brazzaville	0.314	0.244	0.191	4.918	4.316	4.578
cuvette	0.231	-0.172	-0.183	3.757	4.03	4.345
cuvette - ouest	0.659	0.128	0.059	4.861	3.413	3.318
kouilou	0.331	-0.215	-0.404	3.931	3.652	4.161
lekoumou	0.315	0.436	0.24	3.913	4.784	3.832
likouala	-0.153	0.077	0.106	3.464	3.989	5.52
niari	0.539	-0.237	-0.287	4.529	3.192	4.525
plateaux	0.541	-0.246	-0.073	4.661	3.25	3.77
pointe-noire	0.005	0.106	-0.01	3.658	3.511	3.716
pool	0.504	0.099	0.061	4.814	3.912	3.62
sangha	-0.111	0.301	0.674	3.703	4.791	4.433
Total	0.285	0.027	0.03	4.15	3.832	4.268

Congo DR 2007	Skewness who htz	Skewness who wtz	Skewness who wfl	Kurtosis who htz	Kurtosis who wtz	Kurtosis who wfl
bandundu	0.518	0.36	0.436	3.529	4.318	4.462
bas-congo	0.884	0.223	0.326	4.338	3.478	3.825
equateur	0.426	0.015	-0.186	3.284	3.677	3.611
kasaï occident	0.709	-0.147	0.247	3.792	3.237	3.982
kasaï oriental	0.418	-0.034	-0.036	3.408	3.368	4.026
katanga	0.876	0.118	0.151	4.246	3.862	3.735
kinshasa	0.592	0.318	0.001	4.837	5.9	4.605
maniema	0.752	0.278	0.156	3.704	3.761	3.225
nord-kivu	0.564	0.144	-0.027	3.786	3.674	3.267
orientale	0.552	0.632	0.218	3.638	4.235	3.965

	Skewness	Skewness	Skewness	Kurtosis	Kurtosis	Kurtosis
sud-kivu	0.644	0.489	0.03	3.361	3.661	3.711
Total	0.605	0.174	0.138	3.765	3.934	3.802

Côte d'Ivoire 1994	Skewness	Skewness	Skewness	Kurtosis	Kurtosis	Kurtosis
	who htz	who wtz	who wfl	who htz	who wtz	who wfl
center	0.227	-0.158	0.057	3.359	3.332	3.337
center east	0.148	-0.44	0.14	3.338	3.215	3.327
center north	0.103	-0.407	-0.111	3.853	3.753	4.376
center west	0.388	-0.202	-0.156	3.727	3.735	3.337
north	0.126	0.029	-0.027	3.153	3.652	2.881
north east	0.027	-0.438	-0.191	2.782	3.268	3.531
north west	0.078	-0.024	-0.128	3.113	3.533	3.456
south	0.273	-0.113	-0.224	3.832	3.551	3.414
south west	-0.033	-0.15	0.113	3.205	4.024	2.735
west	0.432	-0.412	-0.765	4.156	3.988	4.014
Total	0.219	-0.243	-0.178	3.642	3.713	3.467

Côte d'Ivoire 1998	Skewness	Skewness	Skewness	Kurtosis	Kurtosis	Kurtosis
	who htz	who wtz	who wfl	who htz	who wtz	who wfl
capital (abidjan)	0.375	-0.141	0.07	4.07	4.752	4.117
countryside	0.212	-0.146	-0.005	3.842	3.267	3.759
small city	0.15	-0.057	-0.168	4.227	4.202	4.01
Total	0.126	-0.137	0.001	3.982	3.866	4.005

Côte d'Ivoire 2011	Skewness	Skewness	Skewness	Kurtosis	Kurtosis	Kurtosis
	who htz	who wtz	who wfl	who htz	who wtz	who wfl
Centre	0.342	-0.483	-0.168	3.856	4.684	3.685
Centre-Est	0.124	-0.022	0.231	3.234	3.163	3.568
Centre-Nord	0.569	-0.021	0.447	5.676	4.669	4.907
Centre-Ouest	0.14	-0.087	-0.331	3.96	3.702	3.644
Nord	0.513	-0.422	0.127	4.334	4.11	4.351
Nord-Ouest	0.324	-0.437	-0.236	3.757	3.954	4.149
Nord-est	0.45	-0.64	-0.446	4.046	4.448	4.018
Ouest	0.206	-0.223	-0.086	3.457	3.415	3.451
Sud sans Abidjan	0.642	0.484	0.436	4.605	4.597	3.737
Sud-ouest	0.214	0.127	-0.425	3.523	4.947	3.712
Ville d'Abidjan	0.346	-0.425	0.19	5.026	3.956	4.187
Total	0.364	-0.177	-0.052	4.192	4.338	4.048

Gabon 2000	Skewness	Skewness	Skewness	Kurtosis	Kurtosis	Kurtosis
	who htz	who wtz	who wfl	who htz	who wtz	who wfl
east (haut-ogooué & ogooué-lolo)	0.192	-0.016	-0.149	4.021	4.122	4.168
libreville,port-gentil	0.559	0.132	-0.146	4.61	4.955	4.022
north (ogooué-ivindo & woleu-ntem)	0.57	-0.079	-0.43	5.298	4.646	4.582
south (ngounié, nyanga)	0.619	-0.12	-0.347	5.431	3.985	4.248
west (estuaire, moyen-ogooué & ogooué-maritime)	0.728	0.224	-0.634	5.295	4.654	4.665
Total	0.502	0.052	-0.293	4.778	4.539	4.279

Gabon 2012	Skewness	Skewness	Skewness	Kurtosis	Kurtosis	Kurtosis
	who htz	who wtz	who wfl	who htz	who wtz	who wfl
estuaire	0.473	-0.144	-0.263	5.411	4.794	3.993
haut-ogooué	0.401	0.469	0.36	4.964	4.404	4.273
libreville-port-gentil	-0.034	0.011	-0.246	4.919	4.672	4.547
moyen-ogooué	0.58	-0.294	0.058	5.495	4.046	4.049
ngounié	0.275	-0.162	-0.403	4.606	3.776	4.749
nyanga	0.737	-0.489	-0.733	6.148	5.493	4.418
ogooué maritime	-0.137	-0.041	0.38	3.884	4.075	4.679
ogooué-ivindo	0.545	-0.092	-0.318	4.48	4.005	4.798
ogooué-lolo	0.89	0.32	-0.479	4.967	4.903	4.007
woleu-ntem	-0.039	0.586	0.181	4.62	5.388	3.636
Total	0.337	0.052	-0.077	4.704	4.573	4.563

Ghana 1993	Skewness	Skewness	Skewness	Kurtosis	Kurtosis	Kurtosis
	who htz	who wtz	who wfl	who htz	who wtz	who wfl
ashanti	0.523	-0.49	-0.541	3.598	3.852	3.795
brong-ahafo	0.177	-0.352	-0.101	3.471	3.534	3.832
central	0.39	-0.333	-0.434	4.261	3.388	2.92
eastern	0.19	0.106	0.451	3.328	3.527	3.036
greater accra	-0.571	-1.024	-0.387	3.565	4.919	2.852
northern	0.818	0.062	0.077	4.918	2.702	3.221
upper east	-0.322	-0.487	-0.454	2.824	3.586	3.912

upper west	-0.136	1.148	-0.078	2.901	5.788	2.627
volta	0.084	0.271	-0.218	3.796	4.739	4.273
western	-0.03	-0.129	-0.185	3.283	2.861	2.625
Total	0.247	-0.216	-0.15	3.875	3.669	3.57

Ghana 1998	Skewness	Skewness	Skewness	Kurtosis	Kurtosis	Kurtosis
	who htz	who wtz	who wfl	who htz	who wtz	who wfl
ashanti region	0.378	-0.121	-0.096	3.606	4.305	4.303
brong ahafo region	0.184	-0.06	0.342	3.218	2.811	4.734
central region	-0.169	0.224	0.175	2.95	4.34	4.418
eastern region	0.485	0.115	0.225	4.214	3.397	4.053
greater accra region	0.059	0.245	0.436	2.936	3.202	5.261
northern region	0.383	0.257	-0.011	3.672	4.199	4.251
upper east region	0.647	0.346	-0.394	3.995	4.779	5.216
upper west region	0.525	0.08	-0.324	4.334	4.356	4.569
volta region	0.168	0.077	0.336	3.48	4.509	4.326
western region	0.183	0.129	0.343	3.353	3.244	4.202
Total	0.278	0.081	0.091	3.673	4.037	4.54

Ghana 2003	Skewness	Skewness	Skewness	Kurtosis	Kurtosis	Kurtosis
	who htz	who wtz	who wfl	who htz	who wtz	who wfl
ashanti	0.246	0.046	-0.125	3.893	3.763	3.978
brong ahafo	-0.148	-0.213	0.077	3.16	4.425	4.988
central	0.075	0.272	0.419	3.306	4.848	4.18
eastern	0.663	-0.603	-0.106	5.378	5.052	3.601
greater accra	0.386	-0.317	-0.294	4.431	6.232	4.078
northern	0.616	-0.179	-0.209	4.874	3.179	3.89
upper east	0.613	0.264	-0.005	3.858	4.503	3.974
upper west	0.562	0.48	0.075	4.098	3.859	2.853
volta	0.442	-0.218	-0.434	3.63	3.909	4.108
western	0.354	0.318	0.441	3.412	4.238	4.513
Total	0.361	-0.007	-0.075	3.943	4.29	4.04

Ghana 2008	Skewness	Skewness	Skewness	Kurtosis	Kurtosis	Kurtosis
	who htz	who wtz	who wfl	who htz	who wtz	who wfl
ashanti	0.296	0.31	0.007	3.295	4.143	3.945
brong ahafo	0.393	0.096	0.281	3.448	2.941	2.913
central	0.116	0.027	0.143	3.759	4.503	3.328
eastern	0.27	0.167	0.048	3.6	5.278	3.437
greater accra	0.235	0.716	0.524	4.984	4.038	4.824
northern	0.469	0.355	-0.007	3.54	4.159	4.018
upper east	0.922	0.708	-0.056	5.354	4.444	4.664
upper west	0.5	0.992	0.096	3.871	7.229	4.841
volta	0.493	0.518	0.305	4.178	3.698	4.914
western	0.487	0.463	-0.286	4.793	4.64	4.119
Total	0.393	0.436	0.112	4.043	4.657	4.208

Guinea 1999	Skewness	Skewness	Skewness	Kurtosis	Kurtosis	Kurtosis
	who htz	who wtz	who wfl	who htz	who wtz	who wfl
central guinea	0.538	0.234	0.087	3.942	4.233	4.146
conakry	0.454	-0.387	-0.058	4.411	4.211	4.375
forest guinea	0.479	0.132	-0.22	3.488	3.689	4.039
lower guinea	0.456	-0.176	-0.381	4.055	3.9	3.749
upper guinea	0.574	0.198	0.113	3.613	4.044	4.066
Total	0.48	0.039	-0.106	3.811	3.979	4.005

Guinea 2005	Skewness	Skewness	Skewness	Kurtosis	Kurtosis	Kurtosis
	who htz	who wtz	who wfl	who htz	who wtz	who wfl
boké	0.399	0.194	0.126	3.608	3.792	4.591
conakry	0.841	0.502	-0.108	4.485	4.096	3.698
faranah	0.444	-0.156	-0.21	3.796	2.878	3.998
kankan	0.588	0.11	0.167	3.862	3.784	3.755
kindia	0.806	0.137	-0.329	4.409	3.808	3.96
labé	0.46	0.218	0.291	3.538	3.855	3.854
mamou	0.355	0.059	0.525	3.567	3.679	4.944
n'zérékoré	0.323	-0.319	-0.337	3.468	3.281	3.609
Total	0.537	0.119	-0.01	3.928	3.875	4.041

Guinea 2012	Skewness	Skewness	Skewness	Kurtosis	Kurtosis	Kurtosis
	who htz	who wtz	who wfl	who htz	who wtz	who wfl
Bok?	0.576	0.736	0.307	3.819	4.63	3.862

Conakry	0.392	0.235	-0.118	3.689	3.975	4.568
Faranah	0.26	-0.186	-0.094	3.549	4.12	4.065
Kankan	0.316	0.13	-0.116	3.606	4.143	3.659
Kindia	0.596	0.145	-0.26	4.037	3.564	4.119
Lab?	0.294	0.05	-0.417	3.114	3.262	4.448
Mamou	0.407	0.541	-0.314	3.412	4.447	3.698
N'Z?r?kor?	0.396	-0.237	-0.589	3.554	3.349	4.208
Total	0.428	0.148	-0.224	3.68	3.995	4.036

Liberia 2007	Skewness	Skewness	Skewness	Kurtosis	Kurtosis	Kurtosis
	who htz	who wtz	who wfl	who htz	who wtz	who wfl
monrovia	0.485	0.074	-0.168	4.181	3.949	3.941
north central	0.317	-0.214	-0.381	3.427	3.892	4.558
north western	0.632	0.158	0.201	4.202	3.583	4.395
south central	0.293	-0.41	-0.375	3.651	3.218	3.928
south eastern a	0.505	0.152	-0.323	3.696	3.963	4.315
south eastern b	0.648	-0.191	-0.305	3.935	3.924	3.712
Total	0.465	-0.095	-0.283	3.828	3.876	4.174

Mali 1995	Skewness	Skewness	Skewness	Kurtosis	Kurtosis	Kurtosis
	who htz	who wtz	who wfl	who htz	who wtz	who wfl
bamako	0.334	-0.108	0.323	3.408	3.563	3.452
gao	0.138	-0.093	0.286	3.135	2.848	3.522
kayes	0.042	-0.261	-0.212	2.871	2.899	3.304
koulikoro	0.213	-0.25	-0.052	3.24	2.783	3.042
mopti	0.264	0.024	0.134	2.952	2.931	2.92
sikasso	0.147	-0.207	0.228	3.111	2.758	3.45
ségou	0.163	0	0.004	2.839	3.381	2.975
timbuktu	0.164	0.105	-0.151	3.962	3.202	2.422
Total	0.189	-0.118	0.057	3.193	3.091	3.182

Mali 2001	Skewness	Skewness	Skewness	Kurtosis	Kurtosis	Kurtosis
	who htz	who wtz	who wfl	who htz	who wtz	who wfl
bamako	0.245	-0.162	-0.234	4.412	4.53	4.444
gao	0.338	0.001	-0.081	3.315	3.436	3.823
kayes	0.362	0	-0.01	3.464	3.18	3.969
kidal	0.251	-0.298	-0.535	3.755	3.431	3.279
koulikoro	0.411	-0.111	-0.1	3.746	3.627	3.632
mopti	0.365	-0.023	-0.105	3.44	3.312	3.855
segou	0.367	0.24	0.055	3.124	4.034	3.429
sikasso	0.629	0.063	-0.047	4.441	3.647	3.778
tombouctou	0.664	-0.131	0.273	3.644	3.071	3.719
Total	0.402	-0.011	-0.058	3.618	3.552	3.846

Mali 2006	Skewness	Skewness	Skewness	Kurtosis	Kurtosis	Kurtosis
	who htz	who wtz	who wfl	who htz	who wtz	who wfl
bamako	0.344	0.15	0.252	4.039	4.106	4.195
gao	0.427	0.357	0.295	3.454	3.669	3.651
kayes	0.363	0.135	0.329	3.588	3.647	3.805
kidal	0.153	0.29	0.088	3.093	3.83	2.828
koulikoro	0.499	0.093	-0.076	3.859	3.73	3.706
mopti	0.447	0.059	0.068	3.124	3.533	3.811
segou	0.468	-0.111	0.097	3.773	3.311	3.877
sikasso	0.5	0.165	0.019	3.688	3.94	3.864
tombouctou	0.395	0.054	0.379	3.059	3	3.468
Total	0.417	0.125	0.14	3.512	3.727	3.8

Niger 1992	Skewness	Skewness	Skewness	Kurtosis	Kurtosis	Kurtosis
	who htz	who wtz	who wfl	who htz	who wtz	who wfl
agadez	0.789	-0.032	0.191	5.534	3.502	3.852
diffa	0.223	0.792	0.679	3.298	4.281	4.207
dosso	0.425	0.223	0.075	4.231	4.59	5.201
maradi	0.373	0.178	-0.096	3.464	4.007	3.283
niamey	0.35	0.097	0.196	4.009	3.763	4.161
tahoua	0.621	0.186	0.107	3.918	3.938	4.091
tillabéri	0.467	-0.013	-0.193	3.836	3.678	3.315
zinder	0.534	0.148	-0.012	3.421	3.36	3.413
Total	0.398	0.074	0.055	3.725	3.8	3.922

Niger 1998	Skewness	Skewness	Skewness	Kurtosis	Kurtosis	Kurtosis
	who htz	who wtz	who wfl	who htz	who wtz	who wfl

dosso	0.178	0.055	0.123	3.457	3.486	3.504
maradi	0.542	0.047	0.129	4.108	3.556	3.701
niamey	0.377	-0.129	0.145	3.93	3.935	3.648
tahoua/agadez	0.358	-0.008	-0.047	3.55	3.182	3.135
tillaberi	0.293	0.195	-0.005	3.667	3.594	3.524
zinda/diffa	0.378	0.151	0.074	3.144	3.174	3.026
Total	0.36	0.051	0.069	3.616	3.415	3.465

Niger 2006	Skewness	Skewness	Skewness	Kurtosis	Kurtosis	Kurtosis
	who htz	who wtz	who wfl	who htz	who wtz	who wfl
agadez	0.882	0.404	0.383	4.774	4.959	4.849
diffa	0.597	0.202	0.162	3.797	3.127	4.67
dosso	0.228	-0.213	-0.304	2.91	3.348	4.377
maradi	0.447	0.15	-0.106	3.027	3.072	3.683
niamey	0.059	-0.039	0.305	3.149	4.328	4.738
tahoua	0.439	0.094	0.077	3.67	3.487	4.376
tillabéri	0.223	0.068	0.375	3.303	3.591	4.393
zinder	0.976	0.113	0.351	4.884	3.731	3.505
Total	0.446	0.054	0.135	3.594	3.478	4.259

Niger 2012	Skewness	Skewness	Skewness	Kurtosis	Kurtosis	Kurtosis
	who htz	who wtz	who wfl	who htz	who wtz	who wfl
Agadez	-0.12	0.145	0.261	2.643	3.559	3.18
Diffa	0.583	0.702	0.375	3.961	3.834	3.196
Dosso	0.188	0.003	0.165	3.701	4.017	4.318
Maradi	0.768	0.145	0.223	4.244	3.251	3.772
Niamey	0.172	-0.212	0.269	5.132	5.043	4.865
Tahoua	0.577	0.039	0.097	4.264	3.532	4.011
Tillaberi	0.218	0.274	0.126	3.641	5.106	3.777
Zinder	0.507	0.063	-0.137	3.569	3.836	3.478
Total	0.433	0.075	0.171	4.002	3.615	4.04

Nigeria 1990	Skewness	Skewness	Skewness	Kurtosis	Kurtosis	Kurtosis
	who htz	who wtz	who wfl	who htz	who wtz	who wfl
northeast	0.6	-0.001	-0.277	3.698	3.133	3.553
northwest	0.544	0.241	-0.231	3.535	3.921	3.731
southeast	0.346	-0.221	-0.077	3.617	3.471	4.267
southwest	0.35	-0.068	-0.101	3.913	3.72	4.406
Total	0.387	-0.089	-0.231	3.607	3.525	4.077

Nigeria 2003	Skewness	Skewness	Skewness	Kurtosis	Kurtosis	Kurtosis
	who htz	who wtz	who wfl	who htz	who wtz	who wfl
north central	0.439	-0.2	-0.38	3.975	4.052	4.353
north east	0.711	0.128	0.047	4.286	3.698	4
north west	0.753	0.049	0.003	4.001	3.414	3.276
south east	0.242	0.017	-0.008	3.588	4.359	4.049
south south	0.285	0.148	-0.017	4.204	4.009	3.923
south west	0.073	0.08	-0.008	3.171	4.447	3.428
Total	0.421	-0.016	-0.04	3.634	3.753	3.797

Nigeria 2008	Skewness	Skewness	Skewness	Kurtosis	Kurtosis	Kurtosis
	who htz	who wtz	who wfl	who htz	who wtz	who wfl
north central	0.513	0.038	-0.237	3.504	3.8	3.603
north east	0.603	0.208	0.005	3.339	3.265	2.872
north west	0.721	0.228	0.008	3.349	3.288	2.819
south east	0.296	-0.021	0.071	3.705	4.325	3.993
south south	0.336	0.172	0.101	3.725	4.327	3.81
south west	0.541	0.114	-0.141	3.912	4.236	3.942
Total	0.509	0.032	-0.099	3.392	3.473	3.242

Sao Tome et Principe 2008	Skewness	Skewness	Skewness	Kurtosis	Kurtosis	Kurtosis
	who htz	who wtz	who wfl	who htz	who wtz	who wfl
região centro	0.842	-0.144	0.022	5.385	3.925	3.358
região do principe	0.689	0.2	0.26	4.475	4.334	3.805
região norte	0.651	-0.216	-0.24	4.499	4.262	3.237
região sul	0.702	-0.085	0.107	4.111	3.471	3.079
Total	0.656	-0.043	0.018	4.409	4.05	3.242

Senegal 1992	Skewness	Skewness	Skewness	Kurtosis	Kurtosis	Kurtosis
	who htz	who wtz	who wfl	who htz	who wtz	who wfl
central	0.123	-0.065	0.064	3.383	3.732	4.024

north east	0.186	-0.109	0.06	3.475	3.87	4.175
south	0.307	-0.068	-0.311	3.597	3.816	3.824
west	0.111	-0.038	-0.087	3.682	3.789	4.118
Total	0.111	-0.114	-0.046	3.494	3.796	4.064

**Senegal 2005**	**Skewness**	**Skewness**	**Skewness**	**Kurtosis**	**Kurtosis**	**Kurtosis**
	**who htz**	**who wtz**	**who wfl**	**who htz**	**who wtz**	**who wfl**
dakar	-0.177	0.116	-0.323	4.751	4.073	3.179
diourbel	0.06	0.167	0.101	3.834	2.982	3.626
fatick	0.459	0.063	-0.245	4.287	2.916	3.084
kaolack	0.141	-0.242	-0.215	3.944	4.852	3.812
kolda	0.276	-0.063	-0.145	4.408	3.648	4.128
louga	0.085	-0.14	0.203	4.494	4.129	4.511
matam	0.462	0.036	0.086	4.762	2.599	3.271
saint-louis	0.316	-0.088	-0.123	3.648	3.513	3.332
tambacounda	0.13	-0.353	0.655	4.16	3.444	5.093
thiès	0.375	-0.333	0.043	4.179	4.828	4.749
zuguinchor	0.714	0.323	-0.085	4.699	4.536	4.072
Total	0.191	-0.101	0.036	4.239	3.845	4.042

**Senegal 2010**	**Skewness**	**Skewness**	**Skewness**	**Kurtosis**	**Kurtosis**	**Kurtosis**
	**who htz**	**who wtz**	**who wfl**	**who htz**	**who wtz**	**who wfl**
dakar	0.117	0.669	0.855	4.277	5.125	4.66
diourbel	0.105	0.688	0.91	4.074	5.913	5.748
fatick	0.369	-0.133	-0.189	5.144	5.469	4.616
kaffrine	0.078	-0.245	0.726	3.19	3.324	4.94
kaolack	-0.022	0.565	0.289	3.651	4.972	5.702
kedougou	0.75	0.134	0.141	5.404	5.111	5.391
kolda	0.386	0.118	-0.029	3.924	4.361	3.679
louga	0.463	0.29	0.606	4.71	4.336	4.804
matam	0.151	-0.001	0.548	4.002	3.621	4.617
saint-louis	0.579	0.682	-0.207	5.085	5.473	3.23
sedhiou	0.225	-0.234	0.626	3.64	3.126	4.323
tambacounda	0.572	-0.111	0.122	3.906	3.777	3.975
thiès	0.32	0.079	-0.18	4.011	3.589	3.901
ziguinchor	-0.009	-0.641	0.152	4.768	5.039	4.891
Total	0.302	0.151	0.309	4.244	4.507	4.639

**Sierra Leone 2008**	**Skewness**	**Skewness**	**Skewness**	**Kurtosis**	**Kurtosis**	**Kurtosis**
	**who htz**	**who wtz**	**who wfl**	**who htz**	**who wtz**	**who wfl**
eastern	0.469	0.24	0.171	3.621	3.059	3.446
northern	0.46	0.104	0.144	3.315	3.374	4.196
southern	0.581	0.19	0.246	3.216	3.441	2.935
western	0.438	0.47	0.313	3.352	4.528	3.696
Total	0.478	0.238	0.235	3.406	3.523	3.579

**Togo 1998**	**Skewness**	**Skewness**	**Skewness**	**Kurtosis**	**Kurtosis**	**Kurtosis**
	**who htz**	**who wtz**	**who wfl**	**who htz**	**who wtz**	**who wfl**
centrale	0.236	-0.154	-0.105	3.401	3.396	3.266
kara	0.427	-0.191	0.025	3.976	3.841	3.206
lomé	0.661	-0.495	-0.163	5.245	4.427	4.385
marities	0.376	-0.069	0.345	4.254	4.042	4.502
plateaux	0.132	-0.175	-0.15	3.122	4.013	3.739
savanes	0.458	0.309	0.293	4.157	4.366	3.566
Total	0.326	-0.064	0.065	3.883	3.841	3.576

Sample size, under-5 sex ratio (males per female), chi square value, and p-value, by region NNS

Benin 2008	N	Mean	Mean	Mean
	srstate	srstate	c2srstat	pvsrstat
alibori	403	1.08	0.56	0.4549
atacora	368	0.98	0.04	0.8348
atlantique	308	1.2	2.55	0.1106
borgou	333	0.91	0.68	0.4111
collines	260	1.1	0.55	0.4568
couffo	398	1.14	1.7	0.1925
donga	261	0.99	0	0.9506
littoral	223	1.23	2.37	0.1235
mono	245	0.9	0.69	0.4062
oueme	270	1.31	4.8	0.0285
plateau	235	1.18	1.54	0.2152
zou	242	0.86	1.34	0.2472
Total	3546	1.07	1.34	0.3808

Burkina Faso 2012	N	Mean	Mean	Mean
	srstate	srstate	c2srstat	pvsrstat
bales	775	1.06	0.68	0.4087
bam	841	0.96	0.43	0.5124
banwa	623	1.11	1.75	0.1861
banwa ganzourgou	854	0.96	0.3	0.584
bazega	598	1.15	2.95	0.0859
boulgou	689	0.97	0.18	0.6752
cascades	644	1.01	0.01	0.9372
centre ouest	582	1.05	0.34	0.5617
est	922	1.04	0.28	0.5982
houet	491	1.04	0.16	0.6846
kadiogo	441	1.16	2.47	0.1161
kenedougou	753	1.16	4.31	0.0378
kossi	756	1.04	0.34	0.5606
koulpelogo	834	1.01	0.04	0.8354
kouritenga	708	1.15	3.53	0.0602
kourweogo	660	1.12	1.96	0.1611
mouhoun	641	0.94	0.69	0.4068
nahouri	498	0.98	0.07	0.788
namentenga	881	1.12	2.95	0.0858
nayala	679	1.08	1.07	0.3001
nord	731	1.08	1.15	0.2834
oubritenga	670	1.06	0.6	0.4397
sahel	851	1.21	7.71	0.0055
sanmentenga	862	1.07	1.04	0.3069
sourou	660	1.02	0.05	0.8153
sud ouest	513	0.99	0.02	0.8946
tuy	565	1.24	6.59	0.0103
zoundweogo	629	0.88	2.67	0.1021
Total	19351	1.06	1.6	0.404

Cameroon 2011	N	Mean	Mean	Mean
	srstate	srstate	c2srstat	pvsrstat
extrême-nord	663	1	0	0.969
nord	680	1.01	0.02	0.8781
Total	1343	1.01	0.01	0.923

Central African Rep 2012	N	Mean	Mean	Mean
	srstate	srstate	c2srstat	pvsrstat
bamingui bangoran	918	1.01	0.04	0.843
bangui	809	0.99	0.01	0.916
basse kotto	946	0.98	0.11	0.7451
haut kotto	974	1.09	1.64	0.2
haut mbomou	950	1.06	0.71	0.3989
kemo	933	0.9	2.57	0.1087
lobaye	1091	0.98	0.15	0.6939
mambere kadei	1299	1.01	0.02	0.8897
mbomou	983	1.09	1.71	0.191
nana grebizi	1073	0.96	0.41	0.5215
nana mambere	657	0.9	1.86	0.1721

ombella mpoko	891	1.1	1.89	0.1696
ouaka	824	1.1	1.75	0.1856
ouham	998	0.95	0.58	0.4474
ouham pende	799	1.09	1.36	0.243
sangha mbarere	1083	1.03	0.21	0.6485
vakaga	510	0.98	0.03	0.8594
Total	15738	1.01	0.85	0.4927

Chad June 2012	N srstate	Mean srstate	Mean c2srstat	Mean pvsrstat
Barh El Ghazal	749	1.01	0.01	0.9127
Batha	746	1.11	1.94	0.1641
Guéra	785	0.87	3.85	0.0496
Hadjer Lamis	633	1.01	0.01	0.9051
Kanem	599	0.98	0.08	0.7749
Lac	570	1.04	0.25	0.6152
N'Djamena	799	1	0	0.9718
Ouaddai	602	0.91	1.3	0.2538
Salamat	778	1.16	4.32	0.0376
Sila	768	1.04	0.33	0.5637
Wadi Fira	744	1.12	2.37	0.1236
Total	7773	1.02	1.4	0.4781

Chad (7 regions) Dec/Jan 2012-13	N srstate	Mean srstate	Mean c2srstat	Mean pvsrstat
Logone Occidental	830	1.1	1.93	0.165
Logone Oriental	835	0.98	0.06	0.8086
Mandoul	813	1.02	0.06	0.8061
Mayo-Kebbi Est	1023	1.03	0.22	0.6391
Mayo-Kebbi Ouest	673	1.07	0.79	0.3753
Moyen-Chari	720	1.01	0.01	0.9406
Tandjilé	905	1.01	0.03	0.868
Total	5799	1.03	0.43	0.6616

The Gambia 2012	N srstate	Mean srstate	Mean c2srstat	Mean pvsrstat
banjul	745	1.01	0.03	0.8547
basse	1332	1.05	0.68	0.4111
brikama	855	1.11	2.16	0.1414
janjanburay	1013	1.06	0.95	0.3301
kanifing	764	1.03	0.13	0.7175
kerewan	1114	1.06	0.81	0.3687
kuntaur	1112	1.01	0.03	0.8572
mansakonko	844	1.12	2.51	0.1133
Total	7779	1.05	0.89	0.4689

Guinea-Bissau 2008	N srstate	Mean srstate	Mean c2srstat	Mean pvsrstat
Capitale	445	1.12	1.4	0.236
Est (Bafata e Gabu)	836	1.1	1.73	0.1888
Nord (Biombo, Cacheu e Oio)	752	0.96	0.34	0.5596
Sud (Bolama, Quinara e Tombali)	685	1.07	0.77	0.3795
Total	2718	1.05	1.05	0.3472

Guinée Conakay 2012	N srstate	Mean srstate	Mean c2srstat	Mean pvsrstat
boke nord	855	1.01	0.03	0.8642
boke sud	709	1.05	0.41	0.5232
conakry	891	0.96	0.32	0.569
faranah	700	1.03	0.21	0.6501
kankan	843	1.11	2.4	0.1212
kindia	887	1.08	1.23	0.2678
labe	1082	0.91	2.31	0.1285
mamou	1132	1.01	0.06	0.8121
nzerekore	1108	1.01	0.01	0.9043
Total	8207	1.02	0.78	0.5449

Liberia 2010	N srstate	Mean srstate	Mean c2srstat	Mean pvsrstat
bomi	334	0.98	0.05	0.8268
bong	477	0.96	0.17	0.6803

gbarpolu	369	1.15	1.69	0.1931
grand bassa	405	0.93	0.56	0.4561
grand cape mount	500	1	0	1
grand gedeh	443	1.1	1	0.3184
grand kru	450	0.95	0.32	0.5716
lofa	481	0.95	0.35	0.5533
margibi	385	1.25	4.8	0.0284
maryland	373	0.99	0	0.9587
montserrado	350	1.1	0.73	0.3924
nimba	441	1.18	3.1	0.0781
river gee	422	1.15	2.13	0.1442
rivercess	468	1.03	0.14	0.7115
rural montserrado	421	0.9	1.05	0.3061
sinoe	487	0.9	1.28	0.2573
Total	6806	1.03	1.06	0.4715

**Liberia 2011**	**N srstate**	**Mean srstate**	**Mean c2srstat**	**Mean pvsrstat**
North Central	269	1.05	0.18	0.6695
North Western	93	1.07	0.1	0.7557
South Central	1002	0.98	0.14	0.7046
South Eastern	120	1.22	1.2	0.2733
Total	1484	1.02	0.23	0.6666

**Mali 2011**	**N srstate**	**Mean srstate**	**Mean c2srstat**	**Mean pvsrstat**
bamako	1271	0.96	0.42	0.5188
gao	1133	1.14	4.7	0.0301
kayes	1080	1.03	0.24	0.6264
kidal	233	1.01	0	0.9478
koulikoro	899	1.22	8.81	0.003
mopti	1020	1.08	1.57	0.2104
segou	675	0.99	0.04	0.8474
sikasso	1226	1.03	0.33	0.5679
tombouctou	680	0.95	0.48	0.49
Total	8217	1.05	1.99	0.4149

**Mauritania 2006**	**N srstate**	**Mean srstate**	**Mean c2srstat**	**Mean pvsrstat**
Centre	415	1.11	1.06	0.3026
Fleuve	847	0.98	0.1	0.7571
Nord	230	1.09	0.43	0.5097
Nouakchott	1857	1.05	0.91	0.3414
SudEst	528	1	0	1
Total	3877	1.03	0.59	0.5277

**Mauritania March 2008**	**N srstate**	**Mean srstate**	**Mean c2srstat**	**Mean pvsrstat**
Centre	953	1.12	3.17	0.0748
Fleuve Nord	1330	1.15	6.36	0.0116
Fleuve Sud	1012	1	0	0.9499
Nord	493	1.03	0.1	0.7526
Nouakchott	1375	1.23	14.46	0.0001
SudEst	1222	1.15	5.77	0.0163
Total	6385	1.13	6.03	0.2254

**Mauritania Dec 2008**	**N srstate**	**Mean srstate**	**Mean c2srstat**	**Mean pvsrstat**
Centre	959	0.96	0.46	0.4977
Nord	710	1.08	1.1	0.2933
Nouakchott A	804	1	0	1
Nouakchott B	927	1.02	0.13	0.7179
Sud	1072	1.25	13.43	0.0002
SudEst	989	1.17	5.99	0.0143
Trarza	881	0.97	0.19	0.6614
Total	6342	1.07	3.44	0.434

**Mauritania 2009**	**N srstate**	**Mean srstate**	**Mean c2srstat**	**Mean pvsrstat**
Centre	638	1.07	0.76	0.3838
Nord	688	1.2	5.59	0.0181

Nouakchott	679	0.96	0.33	0.5649
Sud	965	0.98	0.08	0.772
Sud-est	699	1.14	2.9	0.0887
Trarza	709	1.03	0.17	0.6795
Total	4378	1.06	1.55	0.4408

**Mauritania July 2010**	**N**	**Mean**	**Mean**	**Mean**
	**srstate**	**srstate**	**c2srstat**	**pvsrstat**
adrar/inchiri tiris	441	0.98	0.06	0.8118
assaba	755	1.03	0.16	0.6889
brakna	576	1.01	0.01	0.9336
gorgol	723	1.06	0.61	0.4348
guidimakha	632	1.01	0.01	0.9366
hodh chargui	485	0.89	1.5	0.2202
hodh gharbi	531	0.92	0.83	0.3621
nouadhibou	505	1.24	5.99	0.0144
nouakchott	505	1.19	3.66	0.0557
tagant	646	0.95	0.5	0.4788
trarza	584	1.02	0.06	0.8039
Total	6383	1.03	1.1	0.5382

**Mauritania Dec 2010**	**N**	**Mean**	**Mean**	**Mean**
	**srstate**	**srstate**	**c2srstat**	**pvsrstat**
adrar/inchiri tiris	408	1.11	1.19	0.2761
assaba	556	1.04	0.26	0.6108
brakna	567	1.05	0.4	0.5287
gorgol	553	1.05	0.31	0.5804
guidimakha	600	0.91	1.31	0.253
hodh chargui	471	1.28	6.9	0.0086
hodh gharbi	551	1.11	1.53	0.2167
nouadhibou	563	1.09	1.11	0.2921
nouakchott	517	0.94	0.56	0.4547
tagant	589	1.16	3.14	0.0764
trarza	476	1.03	0.13	0.7139
Total	5851	1.07	1.49	0.3645

**Mauritania July 2011**	**N**	**Mean**	**Mean**	**Mean**
	**srstate**	**srstate**	**c2srstat**	**pvsrstat**
adrar/inchiri tiris	677	1	0	0.9693
assaba	706	1.08	1.11	0.292
brakna	743	1.09	1.47	0.226
gorgol	680	0.97	0.15	0.7014
guidimakha	769	0.97	0.22	0.6392
hodh chargui	647	1.02	0.08	0.7832
hodh gharbi	777	1.06	0.68	0.4093
nouadhibou	674	0.95	0.38	0.5377
nouakchott	645	1.05	0.45	0.5033
tagant	742	1.04	0.35	0.5569
trarza	719	0.95	0.5	0.4786
Total	7779	1.02	0.5	0.5486

**Mauritania Dec 2011**	**N**	**Mean**	**Mean**	**Mean**
	**srstate**	**srstate**	**c2srstat**	**pvsrstat**
adrar/inchiri tiris	690	1.09	1.3	0.2534
assaba	763	1.14	3.15	0.0761
brakna	701	1.2	6.03	0.0141
gorgol	791	0.89	2.56	0.1096
guidimakha	855	0.94	0.85	0.3558
hodh chargui	628	0.95	0.41	0.5232
hodh gharbi	803	0.92	1.53	0.2168
nouadhibou	716	0.98	0.09	0.765
nouakchott	711	1.1	1.72	0.1893
tagant	789	1.24	9.16	0.0025
trarza	726	1.09	1.41	0.235
Total	8173	1.05	2.61	0.2436

**Mauritania July 2012**	**N**	**Mean**	**Mean**	**Mean**
	**srstate**	**srstate**	**c2srstat**	**pvsrstat**
adrar/inchiri tiris	451	1.23	4.9	0.0269
assaba	640	1.13	2.5	0.1138
brakna	573	1.28	8.8	0.003

gorgol	610	1.05	0.32	0.5708
guidimakha	856	0.85	5.4	0.0201
hodh chargui	670	1	0	1
hodh gharbi	742	1.21	6.99	0.0082
nouadhibou	474	1.02	0.03	0.8542
nouakchott	590	0.93	0.68	0.4103
tagant	755	1.13	2.93	0.0872
trarza	456	0.96	0.22	0.6396
Total	6817	1.07	3.17	0.3128

**Niger 2012**	**N**	**Mean**	**Mean**	**Mean**
	**srstate**	**srstate**	**c2srstat**	**pvsrstat**
agadez	1222	0.89	4.48	0.0343
diffa	1311	0.99	0.04	0.8467
dosso	1123	1.1	2.32	0.128
maradi	1093	1.11	3.18	0.0743
niamey	1173	1.05	0.62	0.4305
tahoua	1469	1.08	2.37	0.1237
tillabéri	895	0.96	0.32	0.5699
zinder	939	1.09	1.62	0.2031
Total	9225	1.03	1.91	0.2997

**Nigeria (Northern States) 2011**	**N**	**Mean**	**Mean**	**Mean**
	**srstate**	**srstate**	**c2srstat**	**pvsrstat**
jigawa	954	0.92	1.68	0.1953
kano	917	1.07	1.19	0.2758
katsina	1003	1.16	5.31	0.0212
kebbi	1021	1.09	1.98	0.159
sokoto	945	1.04	0.31	0.5803
yobe	1074	1	0	0.9513
zamfara	995	0.96	0.44	0.5056
Total	6909	1.03	1.56	0.3902

**Senegal 2012**	**N**	**Mean**	**Mean**	**Mean**
	**srstate**	**srstate**	**c2srstat**	**pvsrstat**
dakar	391	1.21	3.5	0.0613
kolda	551	1	0	0.966
matam	1948	1.09	3.45	0.0632
myf	719	1.04	0.24	0.6278
sedhiou	2060	0.98	0.19	0.6595
tambacounda	2955	1.02	0.18	0.6722
velingara	611	1.15	3.03	0.0819
Total	9235	1.04	1.2	0.4901

**Sierra Leone 2010**	**N**	**Mean**	**Mean**	**Mean**
	**srstate**	**srstate**	**c2srstat**	**pvsrstat**
eastern	2700	1.05	1.61	0.204
northern	5043	0.99	0.17	0.683
southern	3986	0.96	1.86	0.1731
western	2279	0.98	0.32	0.5717
Total	14008	0.99	0.95	0.4275

**Togo June 2012**	**N**	**Mean**	**Mean**	**Mean**
	**srstate**	**srstate**	**c2srstat**	**pvsrstat**
Centrale	416	1.03	0.09	0.7686
Kara	606	1.03	0.17	0.6846
Lomé	328	1.09	0.6	0.4395
Maritime	474	1.03	0.08	0.7829
Plateaux	371	1.05	0.22	0.6403
Savanes	564	0.91	1.2	0.2736
Total	2759	1.02	0.41	0.595

**Togo Dec 2012**	**N**	**Mean**	**Mean**	**Mean**
	**srstate**	**srstate**	**c2srstat**	**pvsrstat**
Kara	649	0.99	0.01	0.9063
Savanes	802	1.06	0.6	0.4372
Total	1451	1.03	0.34	0.647

Sample size, under-5 sex ratio (males per female), chi square value, and p-value, by region MICS

Burkina Faso 2006	N srstate	Mean srstate	Mean c2srstat	Mean pvsrstat
Boucle du Mouhoun	415	0.96	0.2	0.66
Cascade	627	0.93	0.84	0.36
Centre	519	1.02	0.05	0.83
Centre-Est	333	1.01	0	0.96
Centre-Nord	432	0.92	0.75	0.39
Centre-Ouest	531	1.11	1.37	0.24
Centre-Sud	366	1.02	0.04	0.83
Est	481	1.03	0.13	0.72
Hauts-Bassins	336	1.09	0.58	0.45
Nord	484	1.02	0.03	0.86
Plateau-Central	456	1.21	4.25	0.04
Sahel	381	1.1	0.85	0.36
Sud-Ouest	316	1.07	0.32	0.57
Total	5677	1.03	0.76	0.54

Cameroon 2006	N srstate	Mean srstate	Mean c2srstat	Mean pvsrstat
Adamaoua	649	0.93	0.96	0.33
Centre	560	1.12	1.83	0.18
Douala	385	1.03	0.06	0.80
Est	674	0.88	2.87	0.09
Extreme Nord	747	1	0	0.97
Littoral	426	0.9	1.14	0.29
Nord	815	0.96	0.28	0.60
Nord Ouest	383	1.05	0.21	0.65
Ouest	547	1.1	1.33	0.25
Sud	458	0.93	0.56	0.45
Sud Ouest	382	1.03	0.09	0.76
Yaounde	469	0.96	0.17	0.68
Total	6495	0.99	0.85	0.49

Central African Rep 2000	N srstate	Mean srstate	Mean c2srstat	Mean pvsrstat
Bamingui-Bangoran	465	1.07	0.48	0.49
Bangui	1620	1.08	2.53	0.11
Basse-Kotto	1099	1.11	2.96	0.09
Haut-Mbomou	378	0.99	0.01	0.92
Haute-Kotto	790	1.05	0.51	0.48
Kémo	1031	0.97	0.28	0.60
Lobaye	1122	1.12	3.32	0.07
Mambéré-Kadeï	755	0.99	0.03	0.86
Mbomou	899	1.04	0.32	0.57
Nana-Grébizi	1027	1.02	0.12	0.73
Nana-Mambéré	536	0.99	0.03	0.86
Ombella-M'poko	1094	1.04	0.44	0.51
Ouaka	987	1.08	1.39	0.24
Ouham	624	0.99	0.01	0.94
Ouham-Pendé	556	1.03	0.12	0.73
Sangha-Mbaéré	704	0.92	1.28	0.26
Vakaga	613	1.11	1.78	0.18
Total	14300	1.04	1.14	0.45

Central African Rep 2006	N srstate	Mean srstate	Mean c2srstat	Mean pvsrstat
“Baminigui Bangoran”	577	0.91	1.26	0.26
“Bangui”	754	1.08	1.19	0.27
“Basse Kotto”	484	0.83	4.37	0.04
“Haut Mbomou”	364	1.19	2.81	0.09
“Haute Kotto”	1077	1.05	0.68	0.41
“Lobaye”	657	0.98	0.07	0.78
“Mambere Kadei”	528	1.02	0.03	0.86
“Mbomou”	556	1.01	0.01	0.93
“Nana Mambere”	1271	1.06	1.08	0.30

“Ombella Mpoko”	644	0.96	0.22	0.64
“Ouaka”	675	1.09	1.25	0.26
“Ouham Pende”	629	1.16	3.51	0.06
“Ouham”	688	1	0	1.00
“Sangha Mbaere”	916	0.87	4.76	0.03
Total	9820	1.01	1.48	0.41

Central African Rep 2010	N	Mean	Mean	Mean
	srstate	srstate	c2srstat	pvsrstat
Baminigui Bangoran	427	1.01	0.02	0.88
Bangui	717	1.03	0.17	0.68
Basse Kotto	698	1.11	1.86	0.17
Haut Mbomou	373	0.95	0.22	0.64
Haute-Kotto	662	0.86	3.78	0.05
Kémo	846	0.91	1.71	0.19
Lobaye	890	0.96	0.29	0.59
Mambere Kadei	749	0.85	4.97	0.03
Mbomou	612	0.92	0.94	0.33
Nana Grebizi	696	0.99	0.01	0.94
Nana Mambéré	605	0.96	0.28	0.60
Ombella Mpoko	784	0.9	2.25	0.13
Ouaka	571	1.11	1.47	0.22
Ouham	685	1.05	0.42	0.52
Ouham Pende	710	1.13	2.48	0.12
Sangha Mbaere	821	0.91	1.67	0.20
Vakaga	58	1.42	1.72	0.19
Total	10904	0.98	1.5	0.37

Chad 2000	N	Mean	Mean	Mean
	srstate	srstate	c2srstat	pvsrstat
Autres villes	2000	0.95	1.25	0.26
N'Djaména	846	0.99	0.04	0.84
Rural	2538	0.98	0.23	0.63
Total	5384	0.97	0.58	0.53

Chad 2010	N	Mean	Mean	Mean
	srstate	srstate	c2srstat	pvsrstat
Barh El Gazal	753	1.12	2.23	0.14
Bhata	948	1.04	0.34	0.56
Chari Baguirmi	795	0.95	0.45	0.50
Guéra	1462	0.94	1.45	0.23
Hadjer Lamis	837	1.04	0.27	0.60
Kanem	710	1.09	1.44	0.23
Lac	716	0.93	0.94	0.33
Logone Occidental	747	1.05	0.48	0.49
Logone Oriental	934	0.85	5.86	0.02
Mandoul	880	1.04	0.29	0.59
Mayo Kebbi Est	1219	1	0	0.98
Mayo Kebbi Ouest	910	0.99	0.04	0.84
Moyen Chari	736	1.07	0.78	0.38
Ndjaména	983	0.98	0.12	0.73
Ouaddai	922	0.92	1.57	0.21
Salamat	864	1.05	0.46	0.50
Sila	933	1.08	1.47	0.23
Tandjilé	1054	1.08	1.67	0.20
Wad Fira	732	1.13	2.89	0.09
bet	578	1.05	0.34	0.56
Total	17713	1.01	1.16	0.43

Congo DR 2001	N	Mean	Mean	Mean
	srstate	srstate	c2srstat	pvsrstat
Bandundu	1198	0.93	1.77	0.18
Bas-congo	542	1.16	2.95	0.09
Equateur	1160	0.91	2.7	0.10
Kasai Occidental	958	1.13	3.51	0.06
Kasai Oriental	1114	0.98	0.09	0.76
Katanga	1149	0.97	0.31	0.58
Kinshasa	1473	1.01	0.05	0.81
Maniema	262	1.05	0.14	0.71
Nord-Kivu	740	1.04	0.35	0.56
Orientale	1041	1.09	1.78	0.18

Sud-Kivu	617	0.89	2.22	0.14
Total	10254	1	1.39	0.39

Congo DR 2010	N srstate	Mean srstate	Mean c2srstat	Mean pvsrstat
Bandundu	977	0.99	0.03	0.87
Bas congo	769	0.9	1.98	0.16
Equateur	1056	1.19	7.67	0.01
Kasai Occidental	1083	1.03	0.27	0.61
Kasai Oriental	1048	1.02	0.06	0.80
Katanga	1257	1	0.01	0.93
Kinshasa	843	0.92	1.45	0.23
Maniema	1017	1.07	1.07	0.30
Nord Kivu	1169	0.98	0.07	0.79
Province Orientale	894	1.02	0.11	0.74
Sud Kivu	1132	1.05	0.69	0.41
Total	11245	1.02	1.18	0.55

Côte d’Ivoire 2006	N srstate	Mean srstate	Mean c2srstat	Mean pvsrstat
Centre	664	1.08	0.87	0.35
Centre Est	544	1.11	1.44	0.23
Centre Nord	761	1.07	0.96	0.33
Centre Ouest	799	1.07	0.91	0.34
Nord	653	1.26	8.61	0.00
Nord Est	555	0.99	0.02	0.90
Nord Ouest	927	0.93	1.17	0.28
Ouest	706	0.97	0.2	0.65
Sud (sans ville d' Abidjan)	889	1.07	0.95	0.33
Sud Ouest	1127	1.1	2.68	0.10
Ville Abidjan	979	1.01	0.03	0.87
Total	8604	1.06	1.58	0.39

Equatorial Guinea 2000	N srstate	Mean srstate	Mean c2srstat	Mean pvsrstat
Annobon	14	0.56	1.14	0.29
Bioko Norte	609	1	0	0.97
Bioko Sur	35	1.33	0.71	0.40
Centro Sur	267	1.05	0.18	0.67
Kie Ntem	506	1.02	0.03	0.86
Litoral	715	1.14	3.09	0.08
Wele Nzas	311	1.1	0.72	0.40
Total	2457	1.06	1.03	0.57

Gambia 2000	N srstate	Mean srstate	Mean c2srstat	Mean pvsrstat
Banjul	479	1.21	4.23	0.04
Basse	462	0.89	1.46	0.23
Brikama	451	0.93	0.64	0.42
Janjabureh	365	1.04	0.13	0.71
Kanifing	484	1.2	4	0.05
Kerewan	455	0.9	1.37	0.24
Kuntaur	574	1.03	0.11	0.74
Mansakonko	362	1.32	6.91	0.01
Total	3632	1.06	2.25	0.31

Gambia 2005	N srstate	Mean srstate	Mean c2srstat	Mean pvsrstat
Banjul	167	0.99	0.01	0.94
Basse	1161	0.98	0.15	0.70
Brikama	1390	1.05	0.93	0.33
Janjanburay	754	0.97	0.13	0.72
Kanifing	1461	1.07	1.64	0.20
Kerewan	863	1.08	1.42	0.23
Kuntaur	441	1.07	0.51	0.48
Mansakonko	404	1.12	1.2	0.27
Total	6641	1.04	0.89	0.42

Ghana 2006	N srstate	Mean srstate	Mean c2srstat	Mean pvsrstat
Ashanti	426	1.04	0.15	0.70

Brong Ahafo	245	1.08	0.33	0.57
Central	263	1.02	0.03	0.85
Eastern	346	1.18	2.27	0.13
Greater Accra	330	1.17	2.05	0.15
Northern	595	1.07	0.61	0.44
Upper East	399	1.03	0.06	0.80
Upper West	377	0.92	0.6	0.44
Volta	245	1.09	0.49	0.48
Western	319	1.03	0.08	0.78
Total	3545	1.06	0.67	0.53

**Ghana 2011**	**N**	**Mean**	**Mean**	**Mean**
	**srstate**	**srstate**	**c2srstat**	**pvsrstat**
Asante	476	0.88	1.89	0.17
Brong Ahafo	410	1	0	1.00
Central	1009	1.08	1.36	0.24
Eastern	346	1.01	0.01	0.91
Greater Accra	400	0.97	0.09	0.76
Northern	2008	1.09	4.03	0.04
Upper East	997	1	0	0.97
Upper West	1157	1.17	7.16	0.01
Volta	402	1.08	0.64	0.42
Western	421	0.85	2.59	0.11
Total	7626	1.05	2.63	0.35

**Guinea Bissau 2000**	**N**	**Mean**	**Mean**	**Mean**
	**srstate**	**srstate**	**c2srstat**	**pvsrstat**
Bafatá	971	0.97	0.3	0.59
Biombo	301	0.78	4.55	0.03
Bolama/Bijagós	172	0.7	5.23	0.02
Cacheu	773	1.02	0.06	0.80
Gabú	772	1.06	0.63	0.43
Oio	1131	0.93	1.34	0.25
Quinará	239	1.03	0.04	0.85
Tombali	319	0.95	0.25	0.61
sab	1173	0.99	0.01	0.93
Total	5851	0.97	0.81	0.56

**Guinea Bissau 2006**	**N**	**Mean**	**Mean**	**Mean**
	**srstate**	**srstate**	**c2srstat**	**pvsrstat**
EST (Bafata e Gabu)	1519	0.93	2.14	0.14
NORD (Biombo, Cacheu e Oio)	2725	0.94	3.04	0.08
SAB Capital	1069	1.08	1.42	0.23
SUD (Bolama, Quinara e Tombali)	1257	0.92	2.41	0.12
Total	6570	0.95	2.45	0.13

**Mauritania 2007**	**N**	**Mean**	**Mean**	**Mean**
	**srstate**	**srstate**	**c2srstat**	**pvsrstat**
Adrar	335	1.12	1.08	0.30
Assaba	940	0.95	0.72	0.40
Brakna	884	1.01	0.04	0.84
Gorgol	936	0.97	0.15	0.69
Guidimagha	884	1.07	1.02	0.31
Hodh ECharghi	784	1.13	3.07	0.08
Hodh ELGharbi	736	1.07	0.92	0.34
Inchiri	130	1.1	0.28	0.60
Nouadhibou	600	1.11	1.5	0.22
Nouakchott	1507	1.07	1.86	0.17
Tagant	311	0.99	0	0.95
Tiris Zemmour	308	1.14	1.3	0.25
Trarza	626	1.05	0.41	0.52
Total	8981	1.05	1.07	0.40

**Niger 2000**	**N**	**Mean**	**Mean**	**Mean**
	**srstate**	**srstate**	**c2srstat**	**pvsrstat**
Diffa	68	1	0	1.00
Agadez	420	0.94	0.47	0.49
Dosso	616	0.96	0.23	0.63
Maradi	1206	1.11	3.29	0.07
Niamey	729	1.08	1.08	0.30
Tahoua	732	1.11	1.97	0.16

Tillaberi	644	1.11	1.8	0.18
Zinder	659	1.04	0.3	0.59
Total	5074	1.06	1.56	0.31

Nigeria 2007	N	Mean	Mean	Mean
	srstate	srstate	c2srstat	pvsrstat
Abia	340	0.9	0.95	0.33
Abuja FCT	445	0.99	0.02	0.89
Adamawa	411	1.11	1.07	0.30
Akwa-Ibom	487	1.05	0.35	0.56
Anambra	347	1.31	6.37	0.01
Bauchi	788	0.94	0.86	0.35
Bayelsa	510	1.08	0.78	0.38
Benue	526	1.04	0.19	0.66
Borno	472	0.97	0.14	0.71
Cross-Rivers	369	1.21	3.32	0.07
Delta	333	0.8	4.11	0.04
Ebonyi	469	1.04	0.17	0.68
Edo	391	1.15	1.86	0.17
Ekiti	276	0.76	5.23	0.02
Enugu	341	0.91	0.85	0.36
Gombe	433	1.03	0.11	0.74
Imo	270	1.09	0.53	0.47
Jigawa	821	1.24	9.22	0.00
Kaduna	681	0.99	0.01	0.91
Kano	598	1.05	0.43	0.51
Katsina	546	1.08	0.73	0.39
Kebbi	509	0.97	0.1	0.76
Kogi	326	0.94	0.31	0.58
Kwara	330	0.93	0.44	0.51
Lagos	351	0.99	0	0.96
Nasarawa	544	0.92	0.89	0.35
Niger	608	0.99	0.03	0.87
Ogun	299	0.88	1.21	0.27
Ondo	348	1.08	0.56	0.45
Osun	250	0.95	0.14	0.70
Oyo	358	1.12	1.12	0.29
Plataeu	463	0.95	0.37	0.55
Rivers	316	1.21	2.85	0.09
Sokoto	447	1.13	1.63	0.20
Taraba	525	1.13	2.07	0.15
Yobe	733	1.1	1.67	0.20
Zamfara	829	1.11	2.44	0.12
Total	17090	1.04	1.51	0.42

Nigeria 2011	N	Mean	Mean	Mean
	srstate	srstate	c2srstat	pvsrstat
Abia	465	0.95	0.36	0.55
Adamawa	875	1.02	0.09	0.76
Akwa ibom	564	1.01	0.01	0.93
Anambra	562	0.92	0.86	0.35
Bauchi	1001	1.07	1.22	0.27
Bayelsa	552	1.04	0.26	0.61
Benue	633	1	0	0.97
Borno	796	1.18	5.47	0.02
Cross River	593	1.04	0.2	0.65
Delta	552	1.09	1.04	0.31
Ebonyi	685	0.99	0.01	0.91
Edo	519	1.01	0.02	0.90
Ekiti	402	1.04	0.16	0.69
Enugu	352	0.96	0.18	0.67
FCT (Abuja)	646	1.22	6.34	0.01
Gombe	971	1.01	0.05	0.82
Imo	414	1.1	0.97	0.33
Jigawa	1063	0.92	2.08	0.15
Kaduna	962	1.11	2.6	0.11
Kano	956	0.98	0.15	0.70
Katsina	1024	1.06	0.88	0.35
Kebbi	947	1.03	0.18	0.67
Kogi	430	1.07	0.46	0.50
Kwara	564	1.19	4.09	0.04

Lagos	545	1.12	1.76	0.18
Nasarawa	891	0.94	0.82	0.37
Niger	957	1.1	2.12	0.15
Ogun	588	1.08	0.82	0.36
Ondo	427	1.09	0.85	0.36
Osun	456	0.97	0.08	0.78
Oyo	592	0.92	0.97	0.32
Plateau	687	1.16	3.79	0.05
Rivers	467	0.96	0.17	0.68
Sokoto	1020	1	0	0.95
Taraba	792	1.14	3.41	0.06
Yobe	1015	1.08	1.5	0.22
Zamfara	1053	1.09	1.76	0.19
Total	26018	1.05	1.31	0.44

**Sao Tome et Principe 2000**	**N srstate**	**Mean srstate**	**Mean c2srstat**	**Mean pvsrstat**
Centro	1453	0.94	1.52	0.22
Norte	419	0.97	0.12	0.73
Principe	100	0.79	1.44	0.23
Sul	93	0.82	0.87	0.35
Total	2065	0.93	1.2	0.33

**Senegal 2000**	**N srstate**	**Mean srstate**	**Mean c2srstat**	**Mean pvsrstat**
Dakar	1077	1.01	0.02	0.88
Diourbel	759	0.87	3.43	0.06
Fatick	908	1.03	0.16	0.69
Kaolack	968	0.99	0.04	0.85
Kolda	668	1.03	0.15	0.70
Louga	923	0.97	0.24	0.62
Saint louis	893	0.98	0.09	0.76
Tambacounda	897	1.02	0.13	0.71
Thies	1180	1.07	1.22	0.27
Ziguinchor	760	0.98	0.08	0.77
Total	9033	1	0.54	0.63

**Sierra Leone 2000**	**N srstate**	**Mean srstate**	**Mean c2srstat**	**Mean pvsrstat**
East	701	0.98	0.07	0.79
North	1048	0.98	0.1	0.76
South	357	1.04	0.14	0.71
West	598	1.05	0.33	0.57
Total	2704	1	0.15	0.72

**Sierra Leone 2005**	**N srstate**	**Mean srstate**	**Mean c2srstat**	**Mean pvsrstat**
East	1478	0.96	0.53	0.47
North	2273	1.02	0.32	0.57
South	1638	0.98	0.24	0.62
West	515	0.96	0.23	0.63
Total	5904	0.99	0.34	0.56

**Sierra Leone 2010**	**N srstate**	**Mean srstate**	**Mean c2srstat**	**Mean pvsrstat**
East	1942	1.01	0.06	0.80
North	3310	1.03	0.82	0.37
South	2410	0.94	2.03	0.15
West	1136	1	0	0.95
Total	8798	1	0.88	0.48

**Togo 2006**	**N srstate**	**Mean srstate**	**Mean c2srstat**	**Mean pvsrstat**
Centrale	601	1.07	0.6	0.44
Kara	601	0.81	6.6	0.01
Lomé commune	549	0.99	0.02	0.90
Maritime (sans Lomé commune)	831	1.07	1.01	0.31
Plateaux	569	1.14	2.41	0.12
Savanes	1003	1.06	0.96	0.33
Total	4154	1.03	1.81	0.34

Togo 2010	N	Mean	Mean	Mean
	srstate	srstate	c2srstat	pvsrstat
Centrale	778	1.09	1.49	0.22
Kara	863	0.93	1.11	0.29
Lomé	506	0.98	0.03	0.86
Maritime	825	1.08	1.32	0.25
Plateaux	816	1.1	1.96	0.16
Savanes	1120	1.08	1.73	0.19
Total	4908	1.05	1.38	0.29

Appendix K

Number of children aged 6-29 months, 30-59 months and the age ratio (6-29:30-59 months), by region DHS

Benin 2001	Mean	Mean	Mean	Mean
	Rsix29	Rthi59	ageratetot	ageratreg
atacora	282	317	599	0.89
atlantique	411	426	837	0.96
borgou	322	347	669	0.93
mono	255	268	523	0.95
oueme	267	318	585	0.84
zou	333	359	692	0.93
Total	319	347	666	0.92

Benin 2006	Mean	Mean	Mean	Mean
	Rsix29	Rthi59	ageratetot	ageratreg
alibori	458	529	986	0.87
atacora	564	599	1162	0.94
atlantique	711	677	1388	1.05
borgou	608	658	1267	0.92
collines	421	442	863	0.95
couffo	489	542	1031	0.9
donga	344	304	648	1.13
littoral	386	427	812	0.9
mono	381	408	789	0.93
plateau	296	301	596	0.98
quémé	745	679	1424	1.1
zou	634	651	1285	0.97
Total	540	553	1092	0.97

Benin 2011	Mean	Mean	Mean	Mean
	Rsix29	Rthi59	ageratetot	ageratreg
Alibori	321	400	721	0.8
Atacora	475	569	1044	0.84
Atlantique	554	654	1208	0.85
Borgou	410	495	905	0.83
Collines	310	409	719	0.76
Couffo	344	476	819	0.72
Donga	255	324	579	0.79
Littoral	401	428	829	0.94
Mono	266	380	646	0.7
Ou?m?	605	669	1274	0.9
Plateau	320	366	686	0.87
Zou	468	575	1043	0.81
Total	420	505	926	0.83

Burkina Faso 1993	Mean	Mean	Mean	Mean
	Rsix29	Rthi59	ageratetot	ageratreg
central/south	465	565	1030	0.82
east	364	451	815	0.81
north	299	371	670	0.81
ouagadougou	367	464	831	0.79
west	511	599	1110	0.85
Total	414	504	918	0.82

Burkina Faso 1998	Mean	Mean	Mean	Mean
	Rsix29	Rthi59	ageratetot	ageratreg
central/south	524	596	1120	0.88
east	543	699	1242	0.78
north	273	332	605	0.82
ouagadougou	199	227	426	0.88
west	478	579	1057	0.83
Total	452	548	1000	0.83

Burkina Faso 2003	Mean	Mean	Mean	Mean
	Rsix29	Rthi59	ageratetot	ageratreg
boucle de mouhoun	267	327	594	0.81
cascades	253	285	538	0.89

centre (sans ouagadougou)	113	133	246	0.85
centre-est	270	273	543	0.99
centre-nord	303	328	631	0.92
centre-ouest	351	426	777	0.82
centre-sud	248	280	528	0.89
est	263	323	587	0.82
hauts bassins	283	347	630	0.81
nord	291	274	565	1.06
ouagadougou	86	133	220	0.65
plateau central	361	391	752	0.92
sahel	241	254	495	0.95
sud-ouest	318	399	717	0.8
Total	281	321	602	0.88

**Burkina Faso 2010**	**Mean**	**Mean**	**Mean**	**Mean**
	**Rsix29**	**Rthi59**	**ageratetot**	**ageratreg**
boucle de mouhoun	136	147	283	0.92
cascades	84	94	177	0.9
centre	82	98	180	0.84
centre-est	122	128	250	0.95
centre-nord	114	129	243	0.88
centre-ouest	112	145	257	0.77
centre-sud	88	101	189	0.87
est	124	155	279	0.8
hauts basins	126	143	270	0.88
nord	126	132	258	0.96
plateau central	104	109	213	0.96
sahel	110	112	223	0.98
sud-ouest	107	98	205	1.1
Total	112	125	238	0.91

**Cameroon 1991**	**Mean**	**Mean**	**Mean**	**Mean**
	**Rsix29**	**Rthi59**	**ageratetot**	**ageratreg**
adam/nord/ext-nord	338	474	812	0.71
centre/sud/est	222	227	449	0.98
nord-ouest/sud-ouest	165	204	369	0.81
ouest/littoral	190	214	404	0.89
yaoundé/douala	316	379	695	0.83
Total	268	335	603	0.83

**Cameroon 1998**	**Mean**	**Mean**	**Mean**	**Mean**
	**Rsix29**	**Rthi59**	**ageratetot**	**ageratreg**
central, south, & east	408	97	505	
north/ extreme north/ ad	461	88	549	
northwest & southwest	234	43	277	
west & littoral	301	68	369	
Total	376	79	455	

**Cameroon 2011**	**Mean**	**Mean**	**Mean**	**Mean**
	**Rsix29**	**Rthi59**	**ageratetot**	**ageratreg**
adamaoua	102	108	210	0.95
centre	86	69	155	1.24
douala	82	83	165	0.99
est	81	88	169	0.91
extrême-nord	169	172	341	0.99
littoral	68	61	129	1.1
nord	151	155	305	0.98
nord-ouest	126	120	246	1.04
ouest	104	103	207	1
sud	64	53	117	1.21
sud-ouest	71	72	143	0.98
yaoundé	73	57	131	1.28
Total	108	106	215	1.04

**Central African Rep 1994**	**Mean**	**Mean**	**Mean**	**Mean**
	**Rsix29**	**Rthi59**	**ageratetot**	**ageratreg**
bangui	342	96	438	
rs i	287	64	351	
rs ii	274	80	354	
rs iii	320	72	392	
rs iv	235	53	288	

rs v	218	56	274	
Total	286	72	358	

Chad 1996	Mean	Mean	Mean	Mean
	Rsix29	Rthi59	ageratetot	ageratreg
b.e.t.	22	25	47	0.88
batha	98	126	224	0.78
biltine	70	69	139	1.01
chari-baguirmi	258	325	583	0.79
guéra	106	98	204	1.08
kanem	92	117	209	0.79
lac	83	110	193	0.75
logone occidental	166	210	376	0.79
logone oriental	167	194	361	0.86
mayo-kebbi	272	307	579	0.89
moyen chari	291	311	602	0.94
n'djaména	430	496	926	0.87
ouaddaï	227	263	490	0.86
salamat	94	106	200	0.89
tandjilé	167	223	390	0.75
Total	234	273	507	0.86

Chad 2004	Mean	Mean	Mean	Mean
	Rsix29	Rthi59	ageratetot	ageratreg
b. e. t.	202	249	451	0.81
bar azoum	178	212	390	0.84
centre est	174	190	364	0.92
chari baguirmi	203	226	429	0.9
logone occidental	251	285	536	0.88
mayo kebbi	234	272	506	0.86
moyen chari	212	183	395	1.16
n'djaména	426	461	887	0.92
ouaddai est	149	228	377	0.65
Total	249	281	530	0.89

Congo 2005	Mean	Mean	Mean	Mean
	Rsix29	Rthi59	ageratetot	ageratreg
brazzaville	426	411	837	1.04
nord	432	391	823	1.1
pointe noire	353	321	675	1.1
sud	560	555	1115	1.01
Total	455	434	889	1.06

Congo 2012	Mean	Mean	Mean	Mean
	Rsix29	Rthi59	ageratetot	ageratreg
bouenza	100	115	215	0.88
brazzaville	67	76	143	0.88
cuvette	65	83	148	0.79
cuvette - ouest	59	65	125	0.91
kouilou	108	117	225	0.92
lekoumou	77	80	158	0.96
likouala	92	98	190	0.94
niari	89	105	194	0.84
plateaux	80	80	159	1
pointe-noire	110	104	213	1.06
pool	115	122	237	0.95
sangha	63	75	138	0.84
Total	88	96	184	0.92

Congo DR 2007	Mean	Mean	Mean	Mean
	Rsix29	Rthi59	ageratetot	ageratreg
bandundu	83	84	167	0.99
bas-congo	57	64	120	0.89
equateur	77	79	156	0.97
kasaï occident	74	74	148	1.01
kasaï oriental	84	90	174	0.94
katanga	86	83	169	1.04
kinshasa	89	92	181	0.96
maniema	80	77	158	1.04
nord-kivu	81	83	164	0.98
orientale	73	66	139	1.12

sud-kivu	69	72	142	0.95
Total	78	79	158	0.99

Côte d'Ivoire 1994	Mean	Mean	Mean	Mean
	Rsix29	Rthi59	ageratetot	ageratreg
center	230	50	280	
center east	92	18	110	
center north	227	54	281	
center west	387	99	486	
north	170	29	199	
north east	128	28	156	
north west	142	35	177	
south	651	149	800	
south west	153	51	204	
west	227	68	295	
Total	341	82	423	

Côte d'Ivoire 1998	Mean	Mean	Mean	Mean
	Rsix29	Rthi59	ageratetot	ageratreg
capital (abidjan)	210	219	429	0.96
countryside	323	307	630	1.05
small city	248	228	476	1.09
Total	268	258	525	1.04

Côte d'Ivoire 2011	Mean	Mean	Mean	Mean
	Rsix29	Rthi59	ageratetot	ageratreg
Centre	61	57	118	1.07
Centre-Est	56	60	116	0.94
Centre-Nord	74	89	163	0.83
Centre-Ouest	81	72	153	1.11
Nord	73	78	151	0.93
Nord-Ouest	88	102	190	0.86
Nord-est	68	66	134	1.04
Ouest	79	98	177	0.8
Sud sans Abidjan	43	35	78	1.25
Sud-ouest	64	58	122	1.09
Ville d'Abidjan	59	53	112	
Total	70	73	143	0.97

Gabon 2000	Mean	Mean	Mean	Mean
	Rsix29	Rthi59	ageratetot	ageratreg
east (haut-ogooué & ogooué-lolo)	387	418	805	0.93
libreville,port-gentil	421	487	908	0.86
north (ogooué-ivindo & woleu-ntem)	332	315	647	1.05
south (ngounié, nyanga)	343	355	698	0.97
west (estuaire, moyen-ogooué & ogooué-maritime)	260	324	584	0.8
Total	357	390	747	0.92

Gabon 2012	Mean	Mean	Mean	Mean
	Rsix29	Rthi59	ageratetot	ageratreg
estuaire	76	80	156	0.96
haut-ogooué	114	126	241	0.91
libreville-port-gentil	136	145	281	0.93
moyen-ogooué	79	72	151	1.09
ngounié	123	107	229	1.15
nyanga	88	70	159	1.26
ogooué maritime	75	73	148	1.04
ogooué-ivindo	157	140	297	1.12
ogooué-lolo	95	90	185	1.06
woleu-ntem	64	62	125	1.03
Total	108	104	212	1.05

Ghana 1993	Mean	Mean	Mean	Mean
	Rsix29	Rthi59	ageratetot	ageratreg
ashanti	251	60	311	
brong-ahafo	139	35	174	
central	146	31	177	
eastern	157	30	187	
greater accra	127	25	152	
northern	141	34	175	
upper east	83	24	107	

upper west	46	9	55	
volta	149	30	179	
western	115	37	152	
Total	153	36	189	

Ghana 1998	Mean Rsix29	Mean Rthi59	Mean ageratetot	Mean ageratreg
ashanti region	185	188	373	0.98
brong ahafo region	71	105	176	0.68
central region	118	129	247	0.91
eastern region	153	164	317	0.93
greater accra region	123	116	239	1.06
northern region	128	154	282	0.83
upper east region	127	176	303	0.72
upper west region	106	122	228	0.87
volta region	107	131	238	0.82
western region	152	155	307	0.98
Total	132	149	281	0.89

Ghana 2003	Mean Rsix29	Mean Rthi59	Mean ageratetot	Mean ageratreg
ashanti	202	235	436	0.86
brong ahafo	162	187	349	0.87
central	96	104	199	
eastern	131	123	254	1.06
greater accra	124	132	256	0.94
northern	231	253	484	0.92
upper east	111	133	244	0.84
upper west	137	140	277	0.98
volta	112	99	211	1.13
western	123	132	255	0.93
Total	156	169	325	0.93

Ghana 2008	Mean Rsix29	Mean Rthi59	Mean ageratetot	Mean ageratreg
ashanti	162	176	338	
brong ahafo	108	112	221	0.97
central	85	72	157	1.18
eastern	100	93	192	1.07
greater accra	96	122	218	0.78
northern	159	193	352	
upper east	88	97	186	0.91
upper west	121	98	218	1.24
volta	93	96	190	0.97
western	100	98	198	1.02
Total	118	125	243	1.01

Guinea 1999	Mean Rsix29	Mean Rthi59	Mean ageratetot	Mean ageratreg
central guinea	354	396	750	0.89
conakry	310	321	631	0.97
forest guinea	530	618	1148	0.86
lower guinea	408	493	901	0.83
upper guinea	417	449	866	0.93
Total	419	476	896	0.89

Guinea 2005	Mean Rsix29	Mean Rthi59	Mean ageratetot	Mean ageratreg
boké	64	83	147	0.77
conakry	45	51	95	0.88
faranah	97	85	182	1.14
kankan	112	124	236	0.9
kindia	79	87	166	0.91
labé	51	58	109	0.89
mamou	59	65	124	0.91
n'zérékoré	82	89	170	0.92
Total	77	84	162	0.92

Guinea 2012	Mean Rsix29	Mean Rthi59	Mean ageratetot	Mean ageratreg
Bok?	86	79	165	1.09

Conakry	67	56	123	
Faranah	93	108	201	0.87
Kankan	137	143	280	0.96
Kindia	90	96	186	0.93
Lab?	81	86	167	0.95
Mamou	72	85	157	0.84
N'Z?r?kor?	85	102	187	0.83
Total	92	98	189	0.92

**Liberia 2007**	**Mean**	**Mean**	**Mean**	**Mean**
	**Rsix29**	**Rthi59**	**ageratetot**	**ageratreg**
monrovia	343	308	652	1.11
north central	367	426	793	0.86
north western	229	261	490	0.88
south central	294	312	606	0.94
south eastern a	271	293	563	0.93
south eastern b	337	413	749	0.82
Total	314	345	659	0.92

**Mali 1995**	**Mean**	**Mean**	**Mean**	**Mean**
	**Rsix29**	**Rthi59**	**ageratetot**	**ageratreg**
bamako	348	74	422	
gao	175	36	211	
kayes	502	145	647	
koulikoro	644	146	790	
mopti	342	85	427	
sikasso	610	164	774	
ségou	546	148	694	
timbuktu	158	48	206	
Total	487	125	612	

**Mali 2001**	**Mean**	**Mean**	**Mean**	**Mean**
	**Rsix29**	**Rthi59**	**ageratetot**	**ageratreg**
bamako	450	441	892	1.02
gao	180	161	341	1.12
kayes	625	706	1330	0.88
kidal	69	49	118	1.42
koulikoro	688	757	1445	0.91
mopti	506	552	1058	0.91
segou	587	670	1257	0.88
sikasso	873	867	1740	1.01
tombouctou	138	167	305	0.82
Total	599	639	1237	0.95

**Mali 2006**	**Mean**	**Mean**	**Mean**	**Mean**
	**Rsix29**	**Rthi59**	**ageratetot**	**ageratreg**
bamako	519	513	1032	1.01
gao	346	372	719	0.93
kayes	650	636	1286	1.02
kidal	102	123	226	0.83
koulikoro	653	702	1355	0.93
mopti	756	868	1624	0.87
segou	624	600	1225	1.04
sikasso	1032	997	2028	1.04
tombouctou	306	425	731	0.72
Total	668	690	1358	0.96

**Niger 1992**	**Mean**	**Mean**	**Mean**	**Mean**
	**Rsix29**	**Rthi59**	**ageratetot**	**ageratreg**
agadez	98	92	190	1.07
diffa	54	64	118	0.84
dosso	270	285	555	0.95
maradi	393	426	819	0.92
niamey	417	478	895	0.87
tahoua	403	410	813	0.98
tillabéri	342	325	667	1.05
zinder	394	385	779	1.02
Total	358	374	732	0.96

**Niger 1998**	**Mean**	**Mean**	**Mean**	**Mean**
	**Rsix29**	**Rthi59**	**ageratetot**	**ageratreg**

dosso	427	85	512	
maradi	672	146	818	
niamey	269	56	325	
tahoua/agadez	473	95	568	
tillaberi	502	114	616	
zinda/diffa	481	80	561	
Total	501	103	604	

**Niger 2006**	**Mean**	**Mean**	**Mean**	**Mean**
	**Rsix29**	**Rthi59**	**ageratetot**	**ageratreg**
agadez	62	74	135	
diffa	64	72	136	0.9
dosso	134	154	287	0.87
maradi	128	153	281	0.84
niamey	104	97	201	1.06
tahoua	141	150	291	0.94
tillabéri	117	120	238	0.98
zinder	84	99	184	0.85
Total	111	122	233	0.91

**Niger 2012**	**Mean**	**Mean**	**Mean**	**Mean**
	**Rsix29**	**Rthi59**	**ageratetot**	**ageratreg**
Agadez	74	73	147	1.01
Diffa	98	121	218	0.81
Dosso	158	184	342	0.86
Maradi	239	286	525	0.83
Niamey	103	91	193	1.13
Tahoua	166	193	359	0.86
Tillaberi	146	159	304	0.92
Zinder	134	141	276	0.95
Total	157	178	335	0.9

**Nigeria 1990**	**Mean**	**Mean**	**Mean**	**Mean**
	**Rsix29**	**Rthi59**	**ageratetot**	**ageratreg**
northeast	735	718	1453	1.02
northwest	688	665	1353	1.03
southeast	696	893	1589	0.78
southwest	741	872	1613	0.85
Total	716	793	1509	0.91

**Nigeria 2003**	**Mean**	**Mean**	**Mean**	**Mean**
	**Rsix29**	**Rthi59**	**ageratetot**	**ageratreg**
north central	351	370	721	0.95
north east	463	455	918	1.02
north west	623	575	1198	1.08
south east	185	192	376	0.96
south south	180	182	361	0.99
south west	208	248	456	0.84
Total	409	402	812	1

**Nigeria 2008**	**Mean**	**Mean**	**Mean**	**Mean**
	**Rsix29**	**Rthi59**	**ageratetot**	**ageratreg**
north central	1720	1915	3635	0.9
north east	2316	2340	4656	0.99
north west	2865	2804	5669	1.02
south east	865	877	1742	0.99
south south	1150	1191	2341	0.97
south west	1201	1274	2475	0.94
Total	1962	2000	3961	0.97

**Sao Tome et Principe 2008**	**Mean**	**Mean**	**Mean**	**Mean**
	**Rsix29**	**Rthi59**	**ageratetot**	**ageratreg**
região centro	219	258	477	0.85
região do principe	125	126	252	0.99
região norte	214	227	441	0.94
região sul	198	203	401	0.98
Total	197	214	412	0.93

**Senegal 1992**	**Mean**	**Mean**	**Mean**	**Mean**
	**Rsix29**	**Rthi59**	**ageratetot**	**ageratreg**
central	764	916	1680	0.83

north east	355	414	769	0.86
south	233	299	532	0.78
west	679	787	1466	0.86
Total	602	713	1315	0.84

**Senegal 2005**	**Mean**	**Mean**	**Mean**	**Mean**
	**Rsix29**	**Rthi59**	**ageratetot**	**ageratreg**
dakar	23	25	48	0.91
diourbel	52	44	97	1.18
fatick	30	28	57	1.08
kaolack	47	44	92	1.07
kolda	51	49	100	1.03
louga	45	41	85	1.09
matam	37	39	76	0.94
saint-louis	35	37	72	0.95
tambacounda	45	45	89	1
thiès	49	60	108	0.82
zuguinchor	27	26	53	1.03
Total	41	41	83	1.01

**Senegal 2010**	**Mean**	**Mean**	**Mean**	**Mean**
	**Rsix29**	**Rthi59**	**ageratetot**	**ageratreg**
dakar	41	51	91	0.8
diourbel	53	61	113	0.88
fatick	56	61	117	0.92
kaffrine	56	54	111	1.03
kaolack	68	75	143	0.9
kedougou	26	33	58	0.79
kolda	56	59	115	0.94
louga	54	60	113	0.9
matam	48	51	98	0.95
saint-louis	45	50	95	0.89
sedhiou	45	42	88	1.06
tambacounda	51	51	102	1.01
thiès	55	61	115	0.9
ziguinchor	27	23	50	1.17
Total	51	54	105	0.94

**Sierra Leone 2008**	**Mean**	**Mean**	**Mean**	**Mean**
	**Rsix29**	**Rthi59**	**ageratetot**	**ageratreg**
eastern	125	117	242	1.07
northern	205	164	369	1.25
southern	131	119	250	1.09
western	84	73	157	1.15
Total	146	126	272	1.15

**Togo 1998**	**Mean**	**Mean**	**Mean**	**Mean**
	**Rsix29**	**Rthi59**	**ageratetot**	**ageratreg**
centrale	402	82	484	
kara	406	81	487	
lomé	205	60	265	
marities	352	78	430	
plateaux	524	128	652	
savanes	658	181	839	
Total	471	115	586	

Number of children aged 6-29 months, 30-59 months and the age ratio (6-29:30-59 months), by region NNS

Benin 2008	Mean	Mean	Mean	Mean
	Rsix29	Rthi59	ageratetot	ageratreg
alibori	151	202	353	0.75
atacora	159	186	345	0.85
atlantique	143	160	303	0.89
borgou	162	160	322	1.01
collines	129	124	253	1.04
couffo	169	225	394	0.75
donga	121	135	256	0.89
littoral	99	122	221	0.8
mono	103	133	236	0.77
oueme	116	122	238	
plateau	101	128	229	0.78
zou	112	122	234	0.92
Total	135	159	294	0.86

Burkina Faso 2012	Mean	Mean	Mean	Mean
	Rsix29	Rthi59	ageratetot	ageratreg
bales	353	343	696	1.03
bam	410	350	760	1.17
banwa	318	263	581	1.21
banwa ganzourgou	390	364	754	1.07
bazega	286	256	542	1.12
boulgou	279	343	621	0.81
cascades	300	278	578	1.08
centre ouest	288	253	540	1.14
est	411	407	818	1.01
houet	213	224	437	0.95
kadiogo	186	216	402	0.86
kenedougou	372	310	682	1.2
kossi	364	324	688	1.12
koulpelogo	350	415	765	0.84
kouritenga	338	320	658	1.06
kourweogo	309	304	613	1.02
mouhoun	303	284	587	1.07
nahouri	201	246	447	0.82
namentenga	456	331	787	1.38
nayala	292	302	594	0.97
nord	309	354	663	0.87
oubritenga	313	307	619	1.02
sahel	413	358	771	1.15
sanmentenga	421	383	804	1.1
sourou	329	285	613	1.15
sud ouest	265	208	473	1.27
tuy	269	242	511	1.11
zoundweogo	273	305	578	0.9
Total	333	316	649	1.06

Cameroon 2011	Mean	Mean	Mean	Mean
	Rsix29	Rthi59	ageratetot	ageratreg
extrême-nord	275	335	611	0.82
nord	281	327	608	0.86
Total	278	331	609	0.84

Central African Rep 2012	Mean	Mean	Mean	Mean
	Rsix29	Rthi59	ageratetot	ageratreg
bamingui bangoran	393	412	805	0.95
bangui	377	328	706	
basse kotto	397	439	835	0.9
haut kotto	471	374	845	1.26
haut mbomou	446	398	844	1.12
kemo	486	350	836	1.39
lobaye	507	459	966	1.1
mambere kadei	552	599	1151	0.92
mbomou	479	370	849	1.29
nana grebizi	523	404	927	1.29
nana mambere	289	296	585	0.98

ombella mpoko	434	356	790	1.22
ouaka	396	361	757	1.1
ouham	482	416	897	1.16
ouham pende	426	262	688	1.62
sangha mbarere	498	465	963	1.07
vakaga	223	234	457	
Total	448	398	846	1.15

**Chad June 2012**	**Mean**	**Mean**	**Mean**	**Mean**
	**Rsix29**	**Rthi59**	**ageratetot**	**ageratreg**
Barh El Ghazal	336	332	669	1.01
Batha	301	345	646	0.87
Guéra	324	356	680	0.91
Hadjer Lamis	281	282	564	1
Kanem	255	272	527	0.93
Lac	261	226	487	1.16
N'Djamena	349	339	688	1.03
Ouaddai	282	261	542	1.08
Salamat	316	375	691	0.84
Sila	328	321	649	1.02
Wadi Fira	319	343	662	0.93
Total	308	319	627	0.98

**Chad (7 regions) Dec/Jan 2012-13**	**Mean**	**Mean**	**Mean**	**Mean**
	**Rsix29**	**Rthi59**	**ageratetot**	**ageratreg**
Logone Occidental	375	373	747	1.01
Logone Oriental	376	373	748	1.01
Mandoul	345	364	710	0.95
Mayo-Kebbi Est	445	465	910	0.96
Mayo-Kebbi Ouest	301	303	604	0.99
Moyen-Chari	348	324	672	1.07
Tandjilé	419	405	824	1.03
Total	378	379	757	1

**The Gambia 2012**	**Mean**	**Mean**	**Mean**	**Mean**
	**Rsix29**	**Rthi59**	**ageratetot**	**ageratreg**
banjul	341	352	692	0.97
basse	612	599	1210	1.02
brikama	385	400	785	0.96
janjanburay	470	443	913	1.06
kanifing	332	361	692	0.92
kerewan	490	511	1000	0.96
kuntaur	510	485	994	1.05
mansakonko	389	389	778	1
Total	459	458	916	1

**Guinea-Bissau 2008**	**Mean**	**Mean**	**Mean**	**Mean**
	**Rsix29**	**Rthi59**	**ageratetot**	**ageratreg**
Capitale	215	182	397	
Est (Bafata e Gabu)	406	355	760	1.14
Nord (Biombo, Cacheu e Oio)	373	280	654	1.33
Sud (Bolama, Quinara e Tombali)	287	346	633	0.83
Total	336	304	639	1.11

**Guinée Conakay 2012**	**Mean**	**Mean**	**Mean**	**Mean**
	**Rsix29**	**Rthi59**	**ageratetot**	**ageratreg**
boke nord	394	376	770	1.05
boke sud	324	313	637	1.04
conakry	427	358	785	
faranah	308	316	623	0.97
kankan	412	333	745	1.24
kindia	422	363	785	1.16
labe	561	396	957	
mamou	561	445	1006	1.26
nzerekore	497	485	983	1.02
Total	448	385	833	1.11

**Liberia 2010**	**Mean**	**Mean**	**Mean**	**Mean**
	**Rsix29**	**Rthi59**	**ageratetot**	**ageratreg**
bomi	147	168	315	0.87
bong	220	232	451	0.95

gbarpolu	156	186	342	0.84
grand bassa	184	212	396	0.87
grand cape mount	207	284	491	0.73
grand gedeh	208	227	435	0.92
grand kru	233	205	438	1.13
lofa	234	235	469	1
margibi	169	210	379	0.8
maryland	175	181	355	0.97
montserrado	154	188	342	0.82
nimba	214	219	433	0.98
river gee	208	200	407	1.04
rivercess	225	234	459	0.96
rural montserrado	164	239	403	0.69
sinoe	248	219	467	1.13
Total	200	218	417	0.92

**Liberia 2011**	**Mean**	**Mean**	**Mean**	**Mean**
	**Rsix29**	**Rthi59**	**ageratetot**	**ageratreg**
North Central	222	47	269	
North Western	77	16	93	
South Central	824	178	1002	
South Eastern	99	21	120	
Total	609	131	741	

**Mali 2011**	**Mean**	**Mean**	**Mean**	**Mean**
	**Rsix29**	**Rthi59**	**ageratetot**	**ageratreg**
bamako	620	569	1189	1.09
gao	544	513	1057	1.06
kayes	556	462	1018	1.2
kidal	117	96	213	1.22
koulikoro	446	395	841	1.13
mopti	485	423	907	1.15
segou	302	307	609	0.98
sikasso	575	533	1108	1.08
tombouctou	308	326	634	0.94
Total	493	450	942	1.09

**Mauritania 2006**	**Mean**	**Mean**	**Mean**	**Mean**
	**Rsix29**	**Rthi59**	**ageratetot**	**ageratreg**
Centre	214	199	413	1.08
Fleuve	445	400	845	1.11
Nord	116	114	230	1.02
Nouakchott	964	893	1857	1.08
SudEst	260	268	528	0.97
Total	624	579	1203	1.07

**Mauritania March 2008**	**Mean**	**Mean**	**Mean**	**Mean**
	**Rsix29**	**Rthi59**	**ageratetot**	**ageratreg**
Centre	432	521	953	0.83
Fleuve Nord	693	637	1330	1.09
Fleuve Sud	556	456	1012	1.22
Nord	227	266	493	0.85
Nouakchott	673	702	1375	0.96
SudEst	650	572	1222	1.14
Total	584	564	1148	1.03

**Mauritania Dec 2008**	**Mean**	**Mean**	**Mean**	**Mean**
	**Rsix29**	**Rthi59**	**ageratetot**	**ageratreg**
Centre	422	437	859	0.97
Nord	309	350	659	0.88
Nouakchott A	371	346	717	1.07
Nouakchott B	437	397	834	1.1
Sud	512	431	942	
SudEst	467	418	885	1.12
Trarza	407	390	797	1.04
Total	425	399	824	1.03

**Mauritania 2009**	**Mean**	**Mean**	**Mean**	**Mean**
	**Rsix29**	**Rthi59**	**ageratetot**	**ageratreg**
Centre	296	294	590	1.01
Nord	325	305	630	1.07

Nouakchott	354	270	624	1.31
Sud	478	419	897	1.14
Sud-est	357	282	639	1.27
Trarza	346	301	647	1.15
Total	368	319	686	1.16

Mauritania July 2010	Mean	Mean	Mean	Mean
	Rsix29	Rthi59	ageratetot	ageratreg
adrar/inchiri tiris	210	231	441	0.91
assaba	359	396	755	0.91
brakna	299	276	575	1.08
gorgol	331	392	723	0.84
guidimakha	420	212	632	1.98
hodh chargui	256	228	484	1.12
hodh gharbi	297	234	531	1.27
nouadhibou	274	229	503	1.2
nouakchott	269	235	504	1.14
tagant	312	334	646	0.93
trarza	267	317	584	0.84
Total	306	289	596	1.11

Mauritania Dec 2010	Mean	Mean	Mean	Mean
	Rsix29	Rthi59	ageratetot	ageratreg
adrar/inchiri tiris	207	191	398	1.09
assaba	258	298	556	0.87
brakna	330	237	567	1.39
gorgol	280	273	553	1.03
guidimakha	352	248	600	1.42
hodh chargui	250	221	471	1.13
hodh gharbi	261	289	550	0.9
nouadhibou	264	297	561	0.89
nouakchott	290	225	515	1.29
tagant	261	326	587	0.8
trarza	237	233	469	1.02
Total	275	261	536	1.07

Mauritania July 2011	Mean	Mean	Mean	Mean
	Rsix29	Rthi59	ageratetot	ageratreg
adrar/inchiri tiris	334	338	672	0.99
assaba	328	378	706	0.87
brakna	325	410	735	0.79
gorgol	389	291	680	1.34
guidimakha	454	313	767	1.45
hodh chargui	308	198	506	
hodh gharbi	393	384	777	1.02
nouadhibou	299	375	674	0.8
nouakchott	351	294	645	1.19
tagant	400	340	740	
trarza	333	384	717	0.86
Total	358	339	696	1.04

Mauritania Dec 2011	Mean	Mean	Mean	Mean
	Rsix29	Rthi59	ageratetot	ageratreg
adrar/inchiri tiris	351	339	690	1.04
assaba	358	405	763	0.88
brakna	388	305	693	1.27
gorgol	439	348	787	
guidimakha	501	354	855	1.42
hodh chargui	362	266	628	1.36
hodh gharbi	403	396	799	1.02
nouadhibou	337	379	716	0.89
nouakchott	392	317	709	1.24
tagant	409	380	789	1.08
trarza	314	412	726	0.76
Total	390	357	746	1.09

Mauritania July 2012	Mean	Mean	Mean	Mean
	Rsix29	Rthi59	ageratetot	ageratreg
adrar/inchiri tiris	182	217	399	0.84
assaba	307	280	587	1.1
brakna	314	234	548	1.34

gorgol	338	226	564	1.5
guidimakha	383	395	778	0.97
hodh chargui	340	274	614	
hodh gharbi	347	335	682	1.04
nouadhibou	222	207	429	
nouakchott	240	282	521	0.85
tagant	324	340	664	0.95
trarza	188	236	424	0.8
Total	301	286	587	1.05

**Niger 2012**	**Mean Rsix29**	**Mean Rthi59**	**Mean ageratetot**	**Mean ageratreg**
agadez	545	549	1093	0.99
diffa	554	597	1150	0.93
dosso	486	510	995	0.95
maradi	473	497	970	0.95
niamey	519	558	1077	0.93
tahoua	624	680	1304	0.92
tillabéri	416	387	803	1.07
zinder	425	411	836	1.03
Total	515	537	1052	0.97

**Nigeria (Northern States) 2011**	**Mean Rsix29**	**Mean Rthi59**	**Mean ageratetot**	**Mean ageratreg**
jigawa	408	424	832	0.96
kano	380	453	833	0.84
katsina	462	423	884	1.09
kebbi	435	493	928	0.88
sokoto	396	440	836	0.9
yobe	484	483	966	1
zamfara	404	464	868	0.87
Total	426	455	881	0.94

**Senegal 2012**	**Mean Rsix29**	**Mean Rthi59**	**Mean ageratetot**	**Mean ageratreg**
dakar	138	147	285	0.94
kolda	230	273	502	0.84
matam	924	1022	1946	0.9
myf	352	306	658	1.15
sedhiou	830	874	1704	0.95
tambacounda	1312	1283	2595	1.02
velingara	253	255	508	0.99
Total	859	879	1737	0.97

**Sierra Leone 2010**	**Mean Rsix29**	**Mean Rthi59**	**Mean ageratetot**	**Mean ageratreg**
eastern	1272	1094	2366	1.16
northern	2342	2307	4649	1.02
southern	1850	1835	3685	1.01
western	1165	885	2050	1.32
Total	1804	1707	3511	1.09

**Togo June 2012**	**Mean Rsix29**	**Mean Rthi59**	**Mean ageratetot**	**Mean ageratreg**
Centrale	178	181	358	0.98
Kara	273	251	524	1.08
Lomé	140	129	269	1.08
Maritime	190	219	409	0.87
Plateaux	165	148	313	
Savanes	239	250	489	0.96
Total	207	206	413	0.99

**Togo Dec 2012**	**Mean Rsix29**	**Mean Rthi59**	**Mean ageratetot**	**Mean ageratreg**
Kara	308	271	580	1.14
Savanes	362	373	735	0.97
Total	338	328	665	1.04

Number of children aged 6-29 months, 30-59 months and the age ratio (6-29:30-59 months), by region MICS

Burkina Faso 2006	Mean	Mean	Mean	Mean
	Rsix29	Rthi59	ageratetot	ageratreg
Boucle du Mouhoun	152	175	327	0.87
Cascade	242	258	500	0.94
Centre	186	220	406	0.85
Centre-Est	161	119	280	
Centre-Nord	155	145	300	1.07
Centre-Ouest	185	220	406	0.84
Centre-Sud	141	156	297	0.91
Est	174	147	321	1.18
Hauts-Bassins	138	126	264	1.10
Nord	184	194	378	0.95
Plateau-Central	167	186	353	0.90
Sahel	159	158	316	1.01
Sud-Ouest	147	119	267	1.23
Total	173	179	352	0.97

Cameroon 2006	Mean	Mean	Mean	Mean
	Rsix29	Rthi59	ageratetot	ageratreg
Adamaoua	279	287	566	0.97
Centre	219	259	478	0.85
Douala	158	180	339	0.88
Est	279	317	596	0.88
Extreme Nord	319	310	629	1.03
Littoral	186	192	378	0.97
Nord	331	332	664	1.00
Nord Ouest	158	191	349	0.83
Ouest	232	250	483	0.93
Sud	163	197	359	0.83
Sud Ouest	165	179	344	0.92
Yaounde	196	209	405	0.94
Total	240	256	496	0.93

Central African Rep 2000	Mean	Mean	Mean	Mean
	Rsix29	Rthi59	ageratetot	ageratreg
Bamingui-Bangoran	197	194	391	1.02
Bangui	695	729	1423	0.95
Basse-Kotto	477	484	961	0.99
Haut-Mbomou	160	178	338	0.90
Haute-Kotto	326	353	678	0.92
Kémo	456	447	902	1.02
Lobaye	493	472	965	1.04
Mambéré-Kadeï	317	352	669	0.90
Mbomou	399	401	800	1.00
Nana-Grébizi	467	435	902	1.07
Nana-Mambéré	265	197	462	
Ombella-M'poko	440	520	960	0.85
Ouaka	414	444	858	0.93
Ouham	280	264	543	1.06
Ouham-Pendé	242	237	479	1.02
Sangha-Mbaéré	302	320	621	0.94
Vakaga	286	234	519	1.22
Total	412	419	831	0.98

Central African Rep 2006	Mean	Mean	Mean	Mean
	Rsix29	Rthi59	ageratetot	ageratreg
“Baminigui Bangoran”	196	252	447	0.78
“Bangui”	269	280	549	0.96
“Basse Kotto”	194	207	401	0.94
“Haut Mbomou”	123	144	267	
“Haute Kotto”	432	478	910	0.90
“Lobaye”	287	294	581	0.98
“Mambere Kadei”	219	225	444	0.97
“Mbomou”	218	236	454	0.92
“Nana Mambere”	474	510	983	0.93

“Ombella Mpoko”	253	272	525	0.93
“Ouaka”	234	288	523	0.81
“Ouham Pende”	212	256	469	0.83
“Ouham”	278	314	592	0.89
“Sangha Mbaere”	328	430	758	0.76
Total	295	332	626	0.89

**Central African Rep 2010**	**Mean**	**Mean**	**Mean**	**Mean**
	**Rsix29**	**Rthi59**	**ageratetot**	**ageratreg**
Baminigui Bangoran	141	163	304	
Bangui	253	290	542	0.87
Basse Kotto	301	286	587	1.05
Haut Mbomou	150	133	283	
Haute-Kotto	270	257	527	1.05
Kémo	349	366	715	0.95
Lobaye	350	354	704	0.99
Mambere Kadei	279	325	604	0.86
Mbomou	255	251	506	1.02
Nana Grebizi	305	272	577	1.13
Nana Mambéré	229	270	498	0.85
Ombella Mpoko	332	339	671	0.98
Ouaka	240	231	471	1.04
Ouham	278	290	568	0.96
Ouham Pende	264	306	570	0.86
Sangha Mbaere	319	347	666	0.92
Vakaga	21	17	38	1.26
Total	280	291	570	0.97

**Chad 2000**	**Mean**	**Mean**	**Mean**	**Mean**
	**Rsix29**	**Rthi59**	**ageratetot**	**ageratreg**
Autres villes	806	970	1776	0.83
N'Djaména	360	388	748	0.93
Rural	994	1254	2248	0.79
Total	825	1012	1837	0.83

**Chad 2010**	**Mean**	**Mean**	**Mean**	**Mean**
	**Rsix29**	**Rthi59**	**ageratetot**	**ageratreg**
Barh El Gazal	241	414	655	0.58
Bhata	327	449	776	0.73
Chari Baguirmi	239	381	619	0.63
Guéra	479	704	1183	0.68
Hadjer Lamis	272	425	697	0.64
Kanem	206	388	595	0.53
Lac	242	388	630	0.62
Logone Occidental	282	347	630	0.81
Logone Oriental	338	461	798	0.73
Mandoul	325	424	750	0.77
Mayo Kebbi Est	394	620	1015	0.64
Mayo Kebbi Ouest	346	381	727	0.91
Moyen Chari	267	355	622	0.75
Ndjaména	340	360	700	0.95
Ouaddai	319	443	762	0.72
Salamat	273	439	712	0.62
Sila	274	488	762	0.56
Tandjilé	345	501	846	0.69
Wad Fira	222	351	574	0.63
bet	181	307	488	0.59
Total	310	450	759	0.69

**Congo DR 2001**	**Mean**	**Mean**	**Mean**	**Mean**
	**Rsix29**	**Rthi59**	**ageratetot**	**ageratreg**
Bandundu	491	559	1050	0.88
Bas-congo	243	240	482	1.01
Equateur	511	525	1036	0.97
Kasai Occidental	412	426	838	0.97
Kasai Oriental	507	462	968	1.10
Katanga	497	502	1000	0.99
Kinshasa	635	677	1311	0.94
Maniema	113	120	232	0.94
Nord-Kivu	314	339	653	0.93
Orientale	454	462	916	0.98

Sud-Kivu	267	270	538	0.99
Total	456	471	928	0.97

Congo DR 2010	Mean	Mean	Mean	Mean
	Rsix29	Rthi59	ageratetot	ageratreg
Bandundu	391	463	854	0.85
Bas congo	336	350	685	0.96
Equateur	436	451	887	0.97
Kasai Occidental	439	512	951	0.86
Kasai Oriental	445	440	884	1.01
Katanga	527	554	1081	0.95
Kinshasa	359	359	718	1.00
Maniema	404	459	863	0.88
Nord Kivu	470	555	1025	0.85
Province Orientale	344	432	776	0.80
Sud Kivu	472	493	965	0.96
Total	428	469	896	0.92

Côte d'Ivoire 2006	Mean	Mean	Mean	Mean
	Rsix29	Rthi59	ageratetot	ageratreg
Centre	297	295	592	1.01
Centre Est	230	243	473	0.95
Centre Nord	340	353	693	0.96
Centre Ouest	335	385	720	0.87
Nord	272	313	585	0.87
Nord Est	224	256	480	0.88
Nord Ouest	415	411	826	1.01
Ouest	307	320	627	0.96
Sud (sans ville d' Abidjan)	362	422	784	0.86
Sud Ouest	497	508	1005	0.98
Ville Abidjan	436	433	869	1.01
Total	356	375	731	0.94

Equatorial Guinea 2000	Mean	Mean	Mean	Mean
	Rsix29	Rthi59	ageratetot	ageratreg
Annobon	6	6	12	
Bioko Norte	230	297	527	0.77
Bioko Sur	11	20	31	
Centro Sur	106	129	235	0.82
Kie Ntem	204	211	415	0.97
Litoral	297	340	637	0.87
Wele Nzas	115	154	269	0.75
Total	212	250	462	0.84

Gambia 2000	Mean	Mean	Mean	Mean
	Rsix29	Rthi59	ageratetot	ageratreg
Banjul	226	194	419	1.16
Basse	215	207	422	1.04
Brikama	210	197	406	1.07
Janjabureh	186	135	321	
Kanifing	203	216	418	0.94
Kerewan	227	177	404	
Kuntaur	267	242	508	
Mansakonko	147	158	304	0.93
Total	214	195	409	1.03

Gambia 2005	Mean	Mean	Mean	Mean
	Rsix29	Rthi59	ageratetot	ageratreg
Banjul	72	68	140	1.06
Basse	495	483	979	1.02
Brikama	630	537	1166	1.17
Janjanburay	346	310	655	1.12
Kanifing	604	609	1212	0.99
Kerewan	413	326	739	1.26
Kuntaur	205	162	367	1.27
Mansakonko	185	161	346	1.15
Total	471	431	901	1.11

Ghana 2006	Mean	Mean	Mean	Mean
	Rsix29	Rthi59	ageratetot	ageratreg
Ashanti	163	199	361	0.82

Brong Ahafo	78	130	208	0.60
Central	93	137	230	0.67
Eastern	145	153	298	0.95
Greater Accra	129	155	285	0.83
Northern	253	241	493	1.05
Upper East	152	196	348	0.78
Upper West	140	176	316	0.80
Volta	97	109	206	0.89
Western	122	149	270	0.82
Total	150	175	325	0.84

Ghana 2011	Mean	Mean	Mean	Mean
	Rsix29	Rthi59	ageratetot	ageratreg
Asante	183	228	412	0.80
Brong Ahafo	159	198	356	0.80
Central	389	497	886	0.78
Eastern	129	171	299	0.75
Greater Accra	159	190	349	0.84
Northern	788	995	1784	0.79
Upper East	350	524	874	0.67
Upper West	444	566	1010	0.78
Volta	146	213	359	0.69
Western	165	197	362	0.84
Total	423	547	970	0.77

Guinea Bissau 2000	Mean	Mean	Mean	Mean
	Rsix29	Rthi59	ageratetot	ageratreg
Bafatá	404	458	862	0.88
Biombo	126	128	254	0.98
Bolama/Bijagós	76	72	148	1.06
Cacheu	325	332	657	0.98
Gabú	296	383	679	0.77
Oio	449	526	975	0.85
Quinará	91	116	207	0.78
Tombali	133	154	287	0.86
sab	480	551	1031	0.87
Total	352	404	756	0.88

Guinea Bissau 2006	Mean	Mean	Mean	Mean
	Rsix29	Rthi59	ageratetot	ageratreg
EST (Bafata e Gabu)	538	522	1060	1.03
NORD (Biombo, Cacheu e Oio)	987	977	1965	1.01
SAB Capital	347	359	706	0.97
SUD (Bolama, Quinara e Tombali)	432	453	885	0.95
Total	673	671	1344	1.00

Mauritania 2007	Mean	Mean	Mean	Mean
	Rsix29	Rthi59	ageratetot	ageratreg
Adrar	139	141	280	0.99
Assaba	394	414	808	0.95
Brakna	386	373	759	1.03
Gorgol	375	411	786	0.91
Guidimagha	315	399	714	0.79
Hodh ECharghi	312	299	611	1.04
Hodh ELGharbi	319	301	620	1.06
Inchiri	62	50	111	
Nouadhibou	263	254	516	
Nouakchott	606	631	1237	0.96
Tagant	130	124	254	
Tiris Zemmour	143	107	250	
Trarza	245	282	527	0.87
Total	354	369	724	0.95

Niger 2000	Mean	Mean	Mean	Mean
	Rsix29	Rthi59	ageratetot	ageratreg
Diffa	29	34	63	
Agadez	161	210	371	0.77
Dosso	238	305	543	0.78
Maradi	484	585	1069	0.83
Niamey	303	358	661	0.85
Tahoua	289	345	633	0.84

Tillaberi	252	316	567	0.80
Zinder	264	302	566	0.87
Total	309	374	683	0.82

Nigeria 2007	Mean	Mean	Mean	Mean
	Rsix29	Rthi59	ageratetot	ageratreg
Abia	136	154	290	0.88
Abuja FCT	178	183	362	0.97
Adamawa	191	170	361	1.12
Akwa-Ibom	192	219	411	0.88
Anambra	104	147	251	0.71
Bauchi	315	380	695	0.83
Bayelsa	206	218	424	0.95
Benue	178	213	391	0.83
Borno	201	198	399	1.01
Cross-Rivers	138	179	316	0.77
Delta	135	159	294	0.85
Ebonyi	173	217	391	0.80
Edo	145	188	332	0.77
Ekiti	120	127	246	0.95
Enugu	137	150	287	0.92
Gombe	160	164	324	0.98
Imo	104	119	223	0.87
Jigawa	367	345	712	1.06
Kaduna	316	280	596	1.13
Kano	288	223	511	
Katsina	242	258	500	0.94
Kebbi	246	191	438	1.28
Kogi	103	160	263	0.65
Kwara	112	171	283	0.66
Lagos	137	171	309	0.80
Nasarawa	177	182	359	0.98
Niger	204	322	526	0.63
Ogun	118	125	243	0.95
Ondo	116	168	285	0.69
Osun	84	113	197	0.74
Oyo	144	143	286	1.01
Plataeu	198	192	390	1.03
Rivers	118	133	252	0.89
Sokoto	170	223	393	0.76
Taraba	218	226	443	0.97
Yobe	335	290	625	1.15
Zamfara	363	390	752	0.93
Total	210	224	434	0.92

Nigeria 2011	Mean	Mean	Mean	Mean
	Rsix29	Rthi59	ageratetot	ageratreg
Abia	190	225	415	0.85
Adamawa	307	394	700	0.78
Akwa ibom	219	286	505	0.76
Anambra	209	293	502	0.71
Bauchi	366	441	807	0.83
Bayelsa	212	258	471	0.82
Benue	240	303	543	0.79
Borno	249	354	603	0.70
Cross River	246	278	524	0.89
Delta	216	260	476	0.83
Ebonyi	273	285	558	0.96
Edo	194	260	454	0.74
Ekiti	164	191	355	0.86
Enugu	143	164	306	0.87
FCT (Abuja)	265	275	540	0.97
Gombe	345	437	783	0.79
Imo	167	197	363	0.85
Jigawa	374	549	922	0.68
Kaduna	338	450	788	0.75
Kano	341	428	769	0.80
Katsina	380	515	894	0.74
Kebbi	332	428	760	0.77
Kogi	176	205	381	0.86
Kwara	216	262	478	0.83

Lagos	238	224	462	1.06
Nasarawa	300	347	646	0.86
Niger	319	478	796	0.67
Ogun	255	257	512	0.99
Ondo	169	192	361	0.88
Osun	192	210	402	0.91
Oyo	229	278	507	0.82
Plateau	248	305	553	0.81
Rivers	186	225	411	0.83
Sokoto	387	520	907	0.74
Taraba	266	351	617	0.76
Yobe	333	493	826	0.68
Zamfara	377	502	879	0.75
Total	283	362	645	0.80

Sao Tome et Principe 2000	Mean Rsix29	Mean Rthi59	Mean ageratetot	Mean ageratreg
Centro	630	728	1358	0.87
Norte	175	196	371	0.89
Principe	42	53	95	
Sul	43	56	99	
Total	483	558	1041	0.87

Senegal 2000	Mean Rsix29	Mean Rthi59	Mean ageratetot	Mean ageratreg
Dakar	487	508	994	0.96
Diourbel	277	401	678	0.69
Fatick	353	452	805	0.78
Kaolack	376	464	840	0.81
Kolda	250	330	580	0.76
Louga	400	445	845	0.90
Saint louis	355	443	798	0.80
Tambacounda	355	434	789	0.82
Thies	494	553	1047	0.89
Ziguinchor	288	383	671	0.75
Total	376	451	826	0.83

Sierra Leone 2000	Mean Rsix29	Mean Rthi59	Mean ageratetot	Mean ageratreg
East	300	310	610	0.97
North	414	550	964	0.75
South	166	151	317	1.10
West	242	301	543	0.80
Total	314	380	694	0.86

Sierra Leone 2005	Mean Rsix29	Mean Rthi59	Mean ageratetot	Mean ageratreg
East	367	439	806	0.84
North	843	904	1747	0.93
South	569	639	1208	0.89
West	189	285	474	0.66
Total	591	660	1251	0.87

Sierra Leone 2010	Mean Rsix29	Mean Rthi59	Mean ageratetot	Mean ageratreg
East	736	873	1609	0.84
North	1160	1731	2891	0.67
South	905	1193	2098	0.76
West	412	555	967	0.74
Total	900	1242	2142	0.74

Togo 2006	Mean Rsix29	Mean Rthi59	Mean ageratetot	Mean ageratreg
Centrale	264	272	536	0.97
Kara	242	276	518	0.88
Lomé commune	226	237	464	0.95
Maritime (sans Lomé commune)	344	373	718	0.92
Plateaux	218	225	443	0.97
Savanes	443	438	881	1.01
Total	309	322	631	0.96

Togo 2010	Mean	Mean	Mean	Mean
	Rsix29	Rthi59	ageratetot	ageratreg
Centrale	300	331	631	0.91
Kara	312	388	700	0.80
Lomé	186	212	397	0.88
Maritime	286	391	677	0.73
Plateaux	317	363	681	0.87
Savanes	428	532	959	0.80
Total	320	390	710	0.83

Appendix L

Summary of anthropometric data quality indicators and total data quality score, DHS

Benin 2001, regions	Missing/flagged		Sex ratio		Age ratio		Height DPS		Weight DPS		SD WHZ		Skew WHZ		Kurtosis WHZ		Total score
	%	score	p-value	score	value	score	value	score	value	score	SD	score	value	score	value	score	score
atacora	2.9	5.0	0.406	0.0	0.89	10.0	21.0	10.0	5.0	2.0	1.368	2.0	-0.239	3.0	3.865	3.0	35.0
atlantique	2.2	0.0	1	0.0	0.96	2.0	28.6	10.0	3.6	0.0	1.261	2.0	-0.203	3.0	4.24	5.0	22.0
borgou	3.3	5.0	0.268	0.0	0.93	4.0	28.3	10.0	6.1	2.0	1.458	6.0	0.026	0.0	3.857	3.0	30.0
mono	1.8	0.0	0.552	0.0	0.95	4.0	7.1	2.0	4.8	0.0	1.232	2.0	-0.497	5.0	5.303	5.0	18.0
oueme	2.2	0.0	0.135	0.0	0.84	10.0	26.9	10.0	3.0	0.0	1.295	2.0	-0.247	3.0	4.849	5.0	30.0
zou	1.9	0.0	0.298	0.0	0.93	4.0	26.9	10.0	2.1	0.0	1.317	2.0	-0.375	5.0	3.811	3.0	24.0

Benin 2006, regions	Missing/flagged		Sex ratio		Age ratio		Height DPS		Weight DPS		SD WHZ		Skew WHZ		Kurtosis WHZ		Total score
	%	score	p-value	score	value	score	value	score	value	score	SD	score	value	score	value	score	score
alibori	19.3	20.0	0.775	0.0	0.87	10.0	52.5	10.0	12.7	4.0	1.936	15.0	-0.231	3.0	2.67	1.0	63.0
atacora	6.0	10.0	0.61	0.0	0.94	4.0	17.1	4.0	3.3	0.0	1.482	6.0	0.077	0.0	3.894	3.0	27.0
atlantique	4.4	5.0	0.585	0.0	1.05	4.0	37.3	10.0	5.8	2.0	1.403	6.0	-0.019	0.0	3.616	3.0	30.0
borgou	9.3	10.0	0.959	0.0	0.92	4.0	46.0	10.0	22.7	10.0	1.657	15.0	-0.157	1.0	3.437	1.0	51.0
collines	9.2	10.0	0.8	0.0	0.95	2.0	44.3	10.0	5.5	2.0	1.525	6.0	0.192	1.0	3.906	5.0	36.0
couffo	21.2	20.0	0.2	0.0	0.9	4.0	55.6	10.0	15.1	4.0	1.178	0.0	0.067	0.0	4.222	5.0	43.0
donga	11.6	20.0	0.078	2.0	1.13	10.0	46.8	10.0	8.8	2.0	1.716	15.0	-0.05	0.0	3.116	0.0	59.0
littoral	5.8	10.0	0.353	0.0	0.9	4.0	25.7	10.0	2.2	0.0	1.395	2.0	0.137	1.0	3.735	3.0	30.0
mono	13.2	20.0	0.353	0.0	0.93	4.0	31.6	10.0	11.8	4.0	1.489	6.0	0.03	0.0	3.049	0.0	44.0
plateau	9.6	10.0	0.912	0.0	0.98	0.0	38.3	10.0	6.9	2.0	1.579	6.0	-0.098	0.0	3.368	1.0	29.0
quémé	10.0	20.0	0.087	2.0	1.1	4.0	32.4	10.0	5.2	2.0	1.504	6.0	0.138	1.0	4.208	5.0	50.0
zou	13.4	20.0	0.663	0.0	0.97	0.0	23.8	10.0	11.3	4.0	1.799	15.0	0.227	3.0	2.539	1.0	53.0

Benin 2011, regions	Missing/flagged		Sex ratio		Age ratio		Height DPS		Weight DPS		SD WHZ		Skew WHZ		Kurtosis WHZ		Total score
	%	score	p-value	score	value	score	value	score	value	score	SD	score	value	score	value	score	score
Alibori	22.2	20.0	0.974	0.0	0.8	10.0	85.9	10.0	48.9	10.0	2.163	15.0	0.047	0.0	2.323	3.0	68.0
Atacora	17.6	20.0	0.11	0.0	0.84	10.0	60.7	10.0	22.7	10.0	2.103	15.0	0.124	1.0	2.481	1.0	67.0
Atlantique	32.7	20.0	0.604	0.0	0.85	10.0	52.3	10.0	28.7	10.0	2.288	15.0	0.036	0.0	2.333	3.0	68.0
Borgou	33.1	20.0	0.181	0.0	0.83	10.0	58.6	10.0	16.6	4.0	2.221	15.0	-0.087	0.0	2.49	1.0	60.0
Collines	22.6	20.0	0.946	0.0	0.76	10.0	58.3	10.0	13.7	4.0	2.344	15.0	-0.278	3.0	2.453	1.0	63.0
Couffo	5.1	10.0	0.186	0.0	0.72	10.0	31.5	10.0	7.5	2.0	1.727	15.0	-0.007	0.0	2.911	0.0	47.0
Donga	16.0	20.0	0.006	4.0	0.79	10.0	76.9	10.0	30.0	10.0	2.142	15.0	-0.145	1.0	2.295	3.0	73.0
Littoral	39.0	20.0	0.072	2.0	0.94	4.0	63.9	10.0	35.4	10.0	2.115	15.0	-0.085	0.0	2.666	1.0	62.0
Mono	12.7	20.0	0.827	0.0	0.7	10.0	34.2	10.0	16.6	4.0	1.793	15.0	0.136	1.0	3.026	0.0	60.0
Ou?m?	19.9	20.0	0.12	0.0	0.9	4.0	62.3	10.0	13.2	4.0	2.048	15.0	-0.016	0.0	2.681	1.0	54.0
Plateau	34.0	20.0	0.888	0.0	0.87	10.0	58.5	10.0	25.7	10.0	2.535	15.0	-0.259	3.0	2.231	3.0	71.0
Zou	23.7	20.0	0.431	0.0	0.81	10.0	51.6	10.0	22.7	10.0	2.272	15.0	0.094	0.0	2.487	1.0	66.0

Burkina Faso 1993, regions	Missing/flagged		Sex ratio		Age ratio		Height DPS		Weight DPS		SD WHZ		Skew WHZ		Kurtosis WHZ		Total score
	%	score	p-value	score	value	score	value	score	value	score	SD	score	value	score	value	score	score
central/south	9.1	10.0	0.376	0.0	0.82	10.0	9.9	2.0	3.9	0.0	1.486	6.0	-0.178	1.0	3.549	1.0	30.0
east	12.9	20.0	0.672	0.0	0.81	10.0	21.3	10.0	5.8	2.0	1.509	6.0	0.195	1.0	3.705	3.0	52.0
north	7.7	10.0	0.721	0.0	0.81	10.0	14.8	4.0	6.4	2.0	1.349	2.0	0.004	0.0	4.464	5.0	33.0
ouagadougou	6.4	10.0	0.25	0.0	0.79	10.0	24.6	10.0	6.0	2.0	1.295	2.0	-0.072	0.0	4.003	5.0	39.0
west	8.5	10.0	0.385	0.0	0.85	10.0	14.4	4.0	3.1	0.0	1.337	2.0	-0.262	3.0	3.398	1.0	30.0

Burkina Faso 1998, regions	Missing/flagged		Sex ratio		Age ratio		Height DPS		Weight DPS		SD WHZ		Skew WHZ		Kurtosis WHZ		Total score
	%	score	p-value	score	value	score	value	score	value	score	SD	score	value	score	value	score	score
central/south	5.8	10.0	0.467	0.0	0.88	10.0	10.1	4.0	4.1	0.0	1.381	2.0	-0.078	0.0	3.384	1.0	27.0
east	5.5	10.0	0.576	0.0	0.78	10.0	15.1	4.0	2.3	0.0	1.356	2.0	-0.17	1.0	3.747	3.0	30.0
north	8.2	10.0	0.82	0.0	0.82	10.0	11.4	4.0	3.4	0.0	1.417	6.0	-0.092	0.0	4.082	5.0	35.0
ouagadougou	4.1	5.0	0.754	0.0	0.88	10.0	24.2	10.0	4.7	0.0	1.219	2.0	-0.124	1.0	3.412	1.0	29.0
west	4.1	5.0	0.049	4.0	0.83	10.0	19.5	4.0	2.5	0.0	1.324	2.0	-0.322	5.0	3.423	1.0	31.0

Burkina Faso 2003, regions	Missing/flagged		Sex ratio		Age ratio		Height DPS		Weight DPS		SD WHZ		Skew WHZ		Kurtosis WHZ		Total score
	%	score	p-value	score	value	score	value	score	value	score	SD	score	value	score	value	score	score
boucle de mouhoun	7.4	10.0	0.311	0.0	0.81	10.0	20.9	10.0	34.0	10.0	1.577	6.0	-0.034	0.0	2.76	0.0	46.0
cascades	10.7	20.0	0.245	0.0	0.89	10.0	43.4	10.0	79.6	10.0	1.85	15.0	-0.037	0.0	2.686	1.0	66.0
centre (sans ouagadougou)	11.8	20.0	0.025	4.0	0.85	10.0	42.8	10.0	5.7	2.0	1.467	6.0	-0.316	5.0	3.401	1.0	58.0
centre-est	10.7	20.0	0.75	0.0	0.99	0.0	23.4	10.0	44.6	10.0	1.755	15.0	0.036	0.0	3.097	0.0	55.0
centre-nord	6.4	10.0	0.251	0.0	0.92	4.0	27.6	10.0	10.6	4.0	1.806	15.0	0.247	3.0	3.299	0.0	46.0
centre-ouest	12.3	20.0	0.948	0.0	0.82	10.0	33.2	10.0	2.5	0.0	1.448	6.0	0.034	0.0	3.679	3.0	49.0

centre-sud	14.3	20.0	0.238	0.0	0.89	10.0	14.4	4.0	6.5	2.0	1.805	15.0	0.005	0.0	3.061	0.0	51.0
est	16.0	20.0	0.107	0.0	0.82	10.0	55.8	10.0	53.8	10.0	2.193	15.0	-0.179	1.0	2.614	1.0	67.0
hauts bassins	5.5	10.0	0.72	0.0	0.81	10.0	20.0	10.0	4.1	0.0	1.406	6.0	-0.197	1.0	3.82	3.0	40.0
nord	5.5	10.0	0.626	0.0	1.06	4.0	31.1	10.0	3.5	0.0	1.509	6.0	-0.012	0.0	3.291	0.0	30.0
ouagadougou	6.7	10.0	0.81	0.0	0.65	10.0	41.9	10.0	4.1	0.0	1.388	2.0	0.019	0.0	3.7	3.0	35.0
plateau central	11.3	20.0	0.014	4.0	0.92	4.0	14.4	4.0	40.4	10.0	1.789	15.0	0.15	1.0	2.92	0.0	58.0
sahel	9.0	10.0	0.936	0.0	0.95	2.0	16.2	4.0	6.3	2.0	1.591	6.0	-0.225	3.0	2.89	0.0	27.0
sud-ouest	7.5	10.0	0.002	4.0	0.8	10.0	27.3	10.0	3.5	0.0	1.54	6.0	-0.142	1.0	3.145	0.0	41.0

Burkina Faso 2010, regions	Missing/flagged		Sex ratio		Age ratio		Height DPS		Weight DPS		SD WHZ		Skew WHZ		Kurtosis WHZ		Total score
	%	score	p-value	score	value	score	value	score	value	score	SD	score	value	score	value	score	score
boucle de mouhoun	3.2	5.0	1	0.0	0.92	4.0	10.0	4.0	2.6	0.0	1.251	2.0	-0.217	3.0	3.851	3.0	21.0
cascades	4.5	5.0	0.657	0.0	0.9	10.0	20.6	10.0	7.6	2.0	1.541	6.0	-0.109	1.0	3.547	1.0	35.0
centre	8.9	10.0	0.227	0.0	0.84	10.0	14.4	4.0	7.9	2.0	1.583	6.0	0.075	0.0	4.028	5.0	37.0
centre-est	6.9	10.0	0.09	2.0	0.95	4.0	19.2	4.0	5.6	2.0	1.467	6.0	-0.17	1.0	3.49	1.0	30.0
centre-nord	1.9	0.0	0.338	0.0	0.88	10.0	16.5	4.0	10.8	4.0	1.645	15.0	0.231	3.0	3.461	1.0	37.0
centre-ouest	3.0	5.0	0.775	0.0	0.77	10.0	8.4	2.0	1.6	0.0	1.259	2.0	-0.248	3.0	3.537	1.0	23.0
centre-sud	4.2	5.0	0.589	0.0	0.87	10.0	10.5	4.0	7.7	2.0	1.695	15.0	0.053	0.0	3.683	3.0	39.0
est	7.7	10.0	0.569	0.0	0.8	10.0	15.0	4.0	4.7	0.0	1.46	6.0	0.036	0.0	3.829	3.0	33.0
hauts basins	1.2	0.0	0.929	0.0	0.88	10.0	24.3	10.0	4.8	0.0	1.25	2.0	-0.253	3.0	3.385	1.0	26.0
nord	2.3	0.0	0.564	0.0	0.96	2.0	9.2	2.0	4.5	0.0	1.349	2.0	-0.259	3.0	3.934	5.0	14.0
plateau central	3.6	5.0	0.296	0.0	0.96	2.0	15.2	4.0	6.0	2.0	1.373	2.0	0.071	0.0	3.458	1.0	16.0
sahel	4.2	5.0	0.137	0.0	0.98	0.0	11.3	4.0	6.5	2.0	1.319	2.0	-0.061	0.0	3.277	0.0	13.0
sud-ouest	1.5	0.0	0.534	0.0	1.1	4.0	15.5	4.0	3.7	0.0	1.299	2.0	0.003	0.0	3.681	3.0	13.0

Cameroon 1991, regions	Missing/flagged		Sex ratio		Age ratio		Height DPS		Weight DPS		SD WHZ		Skew WHZ		Kurtosis WHZ		Total score
	%	score	p-value	score	value	score	value	score	value	score	SD	score	value	score	value	score	score
adam/nord/ext-nord	7.4	10.0	0.233	0.0	0.71	10.0	8.7	2.0	4.0	0.0	1.222	2.0	-0.156	1.0	3.732	3.0	28.0
centre/sud/est	6.6	10.0	0.127	0.0	0.98	0.0	14.6	4.0	6.5	2.0	1.092	0.0	-0.16	1.0	3.454	1.0	18.0
nord-ouest/sud-ouest	4.5	5.0	0.843	0.0	0.81	10.0	6.4	2.0	6.7	2.0	1.073	0.0	-0.168	1.0	3.73	3.0	23.0
ouest/littoral	8.8	10.0	0.671	0.0	0.89	10.0	7.5	2.0	7.1	2.0	1.079	0.0	-0.332	5.0	4.698	5.0	34.0
yaoundé/douala	10.9	20.0	0.495	0.0	0.83	10.0	18.9	4.0	3.9	0.0	1.051	0.0	-0.121	1.0	3.94	5.0	40.0

Cameroon 1998, regions	Missing/flagged		Sex ratio		Age ratio		Height DPS		Weight DPS		SD WHZ		Skew WHZ		Kurtosis WHZ		Total score
	%	score	p-value	score	value	score	value	score	value	score	SD	score	value	score	value	score	score
central, south, & east	4.5	5.0	0.422	0.0	.	5.0	23.3	10.0	7.4	2.0	1.296	2.0	-0.218	3.0	3.663	3.0	30.0
north/ extreme north/ ad	5.3	10.0	0.91	0.0	.	5.0	24.8	10.0	3.1	0.0	1.402	6.0	0.218	3.0	3.808	3.0	37.0
northwest & southwest	9.3	10.0	0.511	0.0	.	5.0	17.5	4.0	4.7	0.0	1.708	15.0	-0.519	5.0	3.649	3.0	42.0
west & littoral	14.5	20.0	0.665	0.0	.	5.0	26.1	10.0	6.6	2.0	1.387	2.0	-0.097	0.0	3.807	3.0	42.0

Cameroon 2011, regions	Missing/flagged		Sex ratio		Age ratio		Height DPS		Weight DPS		SD WHZ		Skew WHZ		Kurtosis WHZ		Total score
	%	score	p-value	score	value	score	value	score	value	score	SD	score	value	score	value	score	score
adamaoua	2.5	5.0	0.949	0.0	0.95	4.0	7.8	2.0	2.3	0.0	1.312	2.0	0.208	3.0	3.254	0.0	16.0
centre	2.0	0.0	0.974	0.0	1.24	10.0	4.5	0.0	4.6	0.0	1.149	0.0	-0.331	5.0	3.83	3.0	18.0
douala	1.6	0.0	0.742	0.0	0.99	0.0	10.3	4.0	4.7	0.0	1.223	2.0	0.131	1.0	4.283	5.0	12.0
est	3.4	5.0	0.427	0.0	0.91	4.0	10.6	4.0	5.8	2.0	1.341	2.0	-0.314	5.0	3.475	1.0	23.0
extrême-nord	5.8	10.0	0.063	2.0	0.99	0.0	7.9	2.0	2.2	0.0	1.383	2.0	0.18	1.0	3.904	5.0	22.0
littoral	2.9	5.0	0.796	0.0	1.1	10.0	14.2	4.0	6.1	2.0	1.142	0.0	0.077	0.0	3.636	3.0	24.0
nord	4.7	5.0	0.169	0.0	0.98	0.0	10.3	4.0	5.0	2.0	1.267	2.0	-0.221	3.0	3.541	1.0	17.0
nord-ouest	5.7	10.0	0.24	0.0	1.04	2.0	9.1	2.0	5.2	2.0	1.257	2.0	-0.735	5.0	5.39	5.0	28.0
ouest	5.0	5.0	0.374	0.0	1	0.0	12.5	4.0	5.1	2.0	1.092	0.0	-0.011	0.0	3.789	3.0	14.0
sud	1.8	0.0	0.936	0.0	1.21	10.0	10.3	4.0	8.3	2.0	1.268	2.0	-0.459	5.0	4.349	5.0	28.0
sud-ouest	1.6	0.0	0.688	0.0	0.98	0.0	8.4	2.0	6.0	2.0	1.129	0.0	-0.226	3.0	3.569	1.0	8.0
yaoundé	4.5	5.0	0.357	0.0	1.28	10.0	10.6	4.0	7.6	2.0	1.147	0.0	-0.05	0.0	3.965	5.0	26.0

Central African Rep 1994, regions	Missing/flagged		Sex ratio		Age ratio		Height DPS		Weight DPS		SD WHZ		Skew WHZ		Kurtosis WHZ		Total score
	%	score	p-value	score	value	score	value	score	value	score	SD	score	value	score	value	score	score
bangui	5.4	10.0	0.571	0.0	.	5.0	21.4	10.0	5.9	2.0	1.341	2.0	0.221	3.0	4.129	5.0	37.0
rs i	6.2	10.0	0.462	0.0	.	5.0	17.3	4.0	8.3	2.0	1.394	2.0	-0.102	1.0	3.999	5.0	29.0
rs ii	4.9	5.0	0.168	0.0	.	5.0	14.8	4.0	4.7	0.0	1.337	2.0	-0.101	1.0	3.997	5.0	22.0
rs iii	5.0	5.0	0.018	4.0	.	5.0	26.1	10.0	6.3	2.0	1.361	2.0	0.024	0.0	3.829	3.0	31.0
rs iv	4.4	5.0	0.709	0.0	.	5.0	27.3	10.0	3.6	0.0	1.29	2.0	-0.429	5.0	3.585	1.0	28.0
rs v	5.8	10.0	0.019	4.0	.	5.0	19.3	4.0	4.7	0.0	1.395	2.0	-0.098	0.0	3.338	1.0	26.0

Chad 1996, regions	Missing/flagged		Sex ratio		Age ratio		Height DPS		Weight DPS		SD WHZ		Skew WHZ		Kurtosis WHZ		Total score
	%	score	p-value	score	value	score	value	score	value	score	SD	score	value	score	value	score	score
b.e.t.	5.7	10.0	0.181	0.0	0.88	10.0	16.7	4.0	14.9	4.0	1.604	15.0	0.7	5.0	3.722	3.0	51.0
batha	2.4	0.0	0.614	0.0	0.78	10.0	16.7	4.0	7.0	2.0	1.262	2.0	0.536	5.0	5.198	5.0	28.0
biltine	6.4	10.0	0.511	0.0	1.01	0.0	32.5	10.0	11.1	4.0	1.176	0.0	0.373	5.0	3.954	5.0	34.0

chari-baguirmi	7.4	10.0	0.093	2.0	0.79	10.0	17.1	4.0	2.3	0.0	1.366	2.0	0.001	0.0	3.498	1.0	29.0
guéra	7.9	10.0	0.362	0.0	1.08	4.0	14.1	4.0	6.4	2.0	1.438	6.0	0.04	0.0	3.69	3.0	29.0
kanem	4.5	5.0	0.15	0.0	0.79	10.0	13.9	4.0	5.3	2.0	1.21	2.0	0.058	0.0	3.447	1.0	24.0
lac	10.1	20.0	0.641	0.0	0.75	10.0	35.4	10.0	8.2	2.0	1.538	6.0	0.066	0.0	3.407	1.0	49.0
logone occidental	5.1	10.0	0.144	0.0	0.79	10.0	21.6	10.0	3.4	0.0	1.405	6.0	-0.487	5.0	3.919	5.0	46.0
logone oriental	7.6	10.0	0.086	2.0	0.86	10.0	28.1	10.0	6.2	2.0	1.423	6.0	-0.137	1.0	3.755	3.0	44.0
mayo-kebbi	6.1	10.0	0.042	4.0	0.89	10.0	18.1	4.0	4.3	0.0	1.359	2.0	-0.269	3.0	3.655	3.0	36.0
moyen chari	4.7	5.0	0.469	0.0	0.94	4.0	20.3	10.0	4.3	0.0	1.472	6.0	-0.102	1.0	3.861	3.0	29.0
n'djaména	9.5	10.0	0.285	0.0	0.87	10.0	29.9	10.0	2.9	0.0	1.339	2.0	-0.072	0.0	3.948	5.0	37.0
ouaddaï	4.9	5.0	0.967	0.0	0.86	10.0	25.1	10.0	4.7	0.0	1.235	2.0	-0.388	5.0	3.339	1.0	33.0
salamat	1.4	0.0	0.739	0.0	0.89	10.0	23.0	10.0	7.9	2.0	1.429	6.0	0.265	3.0	4.249	5.0	36.0
tandjilé	5.5	10.0	0.925	0.0	0.75	10.0	33.2	10.0	4.6	0.0	1.333	2.0	-0.306	5.0	3.728	3.0	40.0

Chad 2004, regions	Missing/flagged		Sex ratio		Age ratio		Height DPS		Weight DPS		SD WHZ		Skew WHZ		Kurtosis WHZ		Total score
	%	score	p-value	score	value	score	value	score	value	score	SD	score	value	score	value	score	score
b. e. t.	8.3	10.0	0.965	0.0	0.81	10.0	22.9	10.0	7.0	2.0	1.521	6.0	0.151	1.0	3.666	3.0	42.0
bar azoum	8.0	10.0	0.634	0.0	0.84	10.0	27.4	10.0	4.1	0.0	1.57	6.0	0.201	3.0	3.523	1.0	40.0
centre est	7.1	10.0	0.145	0.0	0.92	4.0	41.3	10.0	6.3	2.0	1.491	6.0	0.333	5.0	4.226	5.0	42.0
chari baguirmi	9.9	10.0	0.789	0.0	0.9	10.0	18.2	4.0	3.9	0.0	1.325	2.0	-0.016	0.0	3.411	1.0	27.0
logone occidental	9.5	10.0	0.292	0.0	0.88	10.0	17.0	4.0	4.2	0.0	1.572	6.0	-0.015	0.0	3.419	1.0	31.0
mayo kebbi	6.4	10.0	0.031	4.0	0.86	10.0	22.6	10.0	5.2	2.0	1.554	6.0	-0.442	5.0	3.393	1.0	48.0
moyen chari	2.4	0.0	0.745	0.0	1.16	10.0	15.6	4.0	4.2	0.0	1.457	6.0	-0.237	3.0	3.446	1.0	24.0
n'djaména	10.5	20.0	0.752	0.0	0.92	4.0	29.4	10.0	3.7	0.0	1.617	15.0	0.082	0.0	3.593	1.0	50.0
ouaddai est	7.2	10.0	0.961	0.0	0.65	10.0	36.1	10.0	8.4	2.0	1.489	6.0	0.464	5.0	3.997	5.0	48.0

Congo 2005, regions	Missing/flagged		Sex ratio		Age ratio		Height DPS		Weight DPS		SD WHZ		Skew WHZ		Kurtosis WHZ		Total score
	%	score	p-value	score	value	score	value	score	value	score	SD	score	value	score	value	score	score
brazzaville	5.8	10.0	0.831	0.0	1.04	2.0	46.6	10.0	4.4	0.0	1.482	6.0	-0.153	1.0	3.588	1.0	30.0
nord	5.5	10.0	0.214	0.0	1.1	10.0	28.0	10.0	3.5	0.0	1.434	6.0	-0.018	0.0	3.527	1.0	37.0
pointe noire	6.4	10.0	0.571	0.0	1.1	4.0	55.1	10.0	3.7	0.0	1.499	6.0	-0.242	3.0	3.718	3.0	36.0
sud	3.9	5.0	0.41	0.0	1.01	0.0	47.8	10.0	3.8	0.0	1.474	6.0	-0.211	3.0	4.102	5.0	29.0

Congo 2012, regions	Missing/flagged		Sex ratio		Age ratio		Height DPS		Weight DPS		SD WHZ		Skew WHZ		Kurtosis WHZ		Total score
	%	score	p-value	score	value	score	value	score	value	score	SD	score	value	score	value	score	score
bouenza	0.7	0.0	0.57	0.0	0.88	10.0	11.0	4.0	5.5	2.0	1.203	2.0	-0.134	1.0	3.858	3.0	22.0
brazzaville	4.4	5.0	0.4	0.0	0.88	10.0	20.6	10.0	3.6	0.0	1.253	2.0	0.191	1.0	4.578	5.0	33.0
cuvette	1.2	0.0	0.394	0.0	0.79	10.0	9.1	2.0	3.1	0.0	1.002	0.0	-0.183	1.0	4.345	5.0	18.0
cuvette - ouest	6.0	10.0	0.592	0.0	0.91	4.0	12.2	4.0	6.0	2.0	1.116	0.0	0.059	0.0	3.318	1.0	21.0
kouilou	1.1	0.0	0.16	0.0	0.92	4.0	10.2	4.0	5.4	2.0	1.111	0.0	-0.404	5.0	4.161	5.0	20.0
lekoumou	1.8	0.0	0.22	0.0	0.96	2.0	20.7	10.0	6.6	2.0	1.263	2.0	0.24	3.0	3.832	3.0	22.0
likouala	0.9	0.0	0.508	0.0	0.94	4.0	8.3	2.0	5.1	2.0	1.141	0.0	0.106	1.0	5.52	5.0	14.0
niari	1.0	0.0	0.831	0.0	0.84	10.0	15.8	4.0	5.3	2.0	1.148	0.0	-0.287	3.0	4.525	5.0	24.0
plateaux	4.1	5.0	0.73	0.0	1	0.0	9.6	2.0	4.3	0.0	1.181	0.0	-0.073	0.0	3.77	3.0	10.0
pointe-noire	1.4	0.0	1	0.0	1.06	4.0	4.8	0.0	6.2	2.0	1.095	0.0	-0.01	0.0	3.716	3.0	9.0
pool	1.9	0.0	0.482	0.0	0.95	4.0	11.2	4.0	5.7	2.0	1.274	2.0	0.061	0.0	3.62	3.0	15.0
sangha	3.1	5.0	0.8	0.0	0.84	10.0	12.4	4.0	6.8	2.0	1.271	2.0	0.674	5.0	4.433	5.0	33.0

Congo DR 2007, regions	Missing/flagged		Sex ratio		Age ratio		Height DPS		Weight DPS		SD WHZ		Skew WHZ		Kurtosis WHZ		Total score
	%	score	p-value	score	value	score	value	score	value	score	SD	score	value	score	value	score	score
bandundu	10.8	20.0	0.941	0.0	0.99	0.0	30.9	10.0	5.4	2.0	1.53	6.0	0.436	5.0	4.462	5.0	48.0
bas-congo	19.4	20.0	0.249	0.0	0.89	10.0	34.0	10.0	4.9	0.0	1.643	15.0	0.326	5.0	3.825	3.0	63.0
equateur	18.8	20.0	0.859	0.0	0.97	0.0	46.3	10.0	8.8	2.0	1.648	15.0	-0.186	1.0	3.611	3.0	51.0
kasaï occident	14.2	20.0	0.207	0.0	1.01	0.0	45.4	10.0	3.9	0.0	1.553	6.0	0.247	3.0	3.982	5.0	44.0
kasaï oriental	23.2	20.0	0.486	0.0	0.94	4.0	40.9	10.0	5.4	2.0	1.65	15.0	-0.036	0.0	4.026	5.0	56.0
katanga	14.6	20.0	0.097	2.0	1.04	2.0	42.1	10.0	5.7	2.0	1.617	15.0	0.151	1.0	3.735	3.0	55.0
kinshasa	13.1	20.0	0.732	0.0	0.96	2.0	28.3	10.0	4.7	0.0	1.325	2.0	0.001	0.0	4.605	5.0	39.0
maniema	16.2	20.0	0.507	0.0	1.04	2.0	24.9	10.0	7.7	2.0	1.784	15.0	0.156	1.0	3.225	0.0	50.0
nord-kivu	15.0	20.0	0.304	0.0	0.98	0.0	29.3	10.0	8.4	2.0	1.636	15.0	-0.027	0.0	3.267	0.0	47.0
orientale	14.0	20.0	0.834	0.0	1.12	10.0	37.6	10.0	7.9	2.0	1.493	6.0	0.218	3.0	3.965	5.0	56.0
sud-kivu	15.6	20.0	0.26	0.0	0.95	2.0	41.3	10.0	8.0	2.0	1.693	15.0	0.03	0.0	3.711	3.0	52.0

Côte d'Ivoire 1994, regions	Missing/flagged		Sex ratio		Age ratio		Height DPS		Weight DPS		SD WHZ		Skew WHZ		Kurtosis WHZ		Total score
	%	score	p-value	score	value	score	value	score	value	score	SD	score	value	score	value	score	score
center	1.8	0.0	0.626	0.0	.	5.0	13.2	4.0	5.8	2.0	1.216	2.0	0.057	0.0	3.337	1.0	14.0
center east	4.1	5.0	0.364	0.0	.	5.0	8.7	2.0	9.2	2.0	1.417	6.0	0.14	1.0	3.327	1.0	22.0
center north	2.5	5.0	0.138	0.0	.	5.0	20.9	10.0	5.1	2.0	1.27	2.0	-0.111	1.0	4.376	5.0	30.0
center west	4.0	5.0	0.326	0.0	.	5.0	17.6	4.0	5.1	2.0	1.367	2.0	-0.156	1.0	3.337	1.0	20.0
north	2.4	0.0	0.707	0.0	.	5.0	12.4	4.0	6.0	2.0	1.423	6.0	-0.027	0.0	2.881	0.0	17.0
north east	3.7	5.0	0.03	4.0	.	5.0	37.3	10.0	5.5	2.0	1.437	6.0	-0.191	1.0	3.531	1.0	34.0

north west	2.3	0.0	0.029	4.0	.	5.0	10.8	4.0	6.8	2.0	1.298	2.0	-0.128	1.0	3.456	1.0	19.0
south	1.0	0.0	0.108	0.0	.	5.0	26.6	10.0	2.3	0.0	1.243	2.0	-0.224	3.0	3.414	1.0	21.0
south west	1.6	0.0	0.752	0.0	.	5.0	26.2	10.0	5.2	2.0	1.153	0.0	0.113	1.0	2.735	0.0	18.0
west	3.8	5.0	0.171	0.0	.	5.0	33.9	10.0	5.8	2.0	1.257	2.0	-0.765	5.0	4.014	5.0	34.0

Côte d'Ivoire 1998, regions	Missing/flagged		Sex ratio		Age ratio		Height DPS		Weight DPS		SD WHZ		Skew WHZ		Kurtosis WHZ		Total score
	%	score	p-value	score	value	score	value	score	value	score	SD	score	value	score	value	score	score
capital (abidjan)	2.7	5.0	0.463	0.0	0.96	2.0	25.9	10.0	4.4	0.0	1.298	2.0	0.07	0.0	4.117	5.0	24.0
countryside	2.4	0.0	0.304	0.0	1.05	4.0	16.4	4.0	4.2	0.0	1.246	2.0	-0.005	0.0	3.759	3.0	13.0
small city	5.9	10.0	0.666	0.0	1.09	4.0	40.2	10.0	7.8	2.0	1.502	6.0	-0.168	1.0	4.01	5.0	38.0

Côte d'Ivoire 2011, regions	Missing/flagged		Sex ratio		Age ratio		Height DPS		Weight DPS		SD WHZ		Skew WHZ		Kurtosis WHZ		Total score
	%	score	p-value	score	value	score	value	score	value	score	SD	score	value	score	value	score	score
Centre	4.3	5.0	0.934	0.0	1.07	4.0	8.6	2.0	8.9	2.0	1.196	0.0	-0.168	1.0	3.685	3.0	17.0
Centre-Est	5.2	10.0	0.526	0.0	0.94	4.0	9.7	2.0	4.4	0.0	1.065	0.0	0.231	3.0	3.568	1.0	20.0
Centre-Nord	3.0	5.0	0.552	0.0	0.83	10.0	8.3	2.0	4.0	0.0	1.201	2.0	0.447	5.0	4.907	5.0	29.0
Centre-Ouest	12.7	20.0	0.969	0.0	1.11	10.0	42.3	10.0	6.0	2.0	1.297	2.0	-0.331	5.0	3.644	3.0	52.0
Nord	3.3	5.0	0.297	0.0	0.93	4.0	10.9	4.0	6.8	2.0	1.205	2.0	0.127	1.0	4.351	5.0	23.0
Nord-Ouest	6.2	10.0	0.137	0.0	0.86	10.0	10.8	4.0	3.5	0.0	1.199	0.0	-0.236	3.0	4.149	5.0	32.0
Nord-est	6.9	10.0	0.181	0.0	1.04	2.0	21.0	10.0	6.8	2.0	1.308	2.0	-0.446	5.0	4.018	5.0	36.0
Ouest	3.3	5.0	0.7	0.0	0.8	10.0	16.5	4.0	5.9	2.0	1.188	0.0	-0.086	0.0	3.451	1.0	22.0
Sud sans Abidjan	8.3	10.0	0.785	0.0	1.25	10.0	22.6	10.0	4.6	0.0	1.406	6.0	0.436	5.0	3.737	3.0	44.0
Sud-ouest	11.1	20.0	0.073	2.0	1.09	4.0	12.0	4.0	9.1	2.0	1.312	2.0	-0.425	5.0	3.712	3.0	42.0
Ville d'Abidjan	12.8	20.0	0.805	0.0	.	5.0	24.9	10.0	6.1	2.0	1.315	2.0	0.19	1.0	4.187	5.0	45.0

Gabon 2000, regions	Missing/flagged		Sex ratio		Age ratio		Height DPS		Weight DPS		SD WHZ		Skew WHZ		Kurtosis WHZ		Total score
	%	score	p-value	score	value	score	value	score	value	score	SD	score	value	score	value	score	score
east (haut-ogooué & ogooué-lolo)	3.3	5.0	0.344	0.0	0.93	4.0	11.7	4.0	3.2	0.0	1.228	2.0	-0.149	1.0	4.168	5.0	21.0
libreville,port-gentil	6.3	10.0	0.395	0.0	0.86	10.0	18.3	4.0	1.9	0.0	1.284	2.0	-0.146	1.0	4.022	5.0	32.0
north (ogooué-ivindo & woleu-ntem)	1.6	0.0	0.97	0.0	1.05	4.0	12.9	4.0	3.9	0.0	1.141	0.0	-0.43	5.0	4.582	5.0	18.0
south (ngounié, nyanga)	1.0	0.0	0.614	0.0	0.97	2.0	13.8	4.0	4.3	0.0	1.172	0.0	-0.347	5.0	4.248	5.0	16.0
west (estuaire, moyen-ogooué & ogooué-maritime)	2.4	0.0	0.727	0.0	0.8	10.0	17.9	4.0	3.3	0.0	1.167	0.0	-0.634	5.0	4.665	5.0	24.0

Gabon 2012, regions	Missing/flagged		Sex ratio		Age ratio		Height DPS		Weight DPS		SD WHZ		Skew WHZ		Kurtosis WHZ		Total score
	%	score	p-value	score	value	score	value	score	value	score	SD	score	value	score	value	score	score
estuaire	5.7	10.0	0.091	2.0	0.96	2.0	14.0	4.0	7.4	2.0	1.168	0.0	-0.263	3.0	3.993	5.0	28.0
haut-ogooué	7.0	10.0	0.109	0.0	0.91	4.0	16.1	4.0	6.5	2.0	1.463	6.0	0.36	5.0	4.273	5.0	36.0
libreville-port-gentil	8.0	10.0	0.006	4.0	0.93	4.0	17.4	4.0	5.5	2.0	1.278	2.0	-0.246	3.0	4.547	5.0	34.0
moyen-ogooué	4.4	5.0	0.739	0.0	1.09	4.0	10.7	4.0	6.6	2.0	1.181	0.0	0.058	0.0	4.049	5.0	20.0
ngounié	3.3	5.0	0.415	0.0	1.15	10.0	10.5	4.0	4.0	0.0	1.179	0.0	-0.403	5.0	4.749	5.0	29.0
nyanga	4.2	5.0	0.386	0.0	1.26	10.0	13.4	4.0	4.4	0.0	1.125	0.0	-0.733	5.0	4.418	5.0	29.0
ogooué maritime	8.5	10.0	0.501	0.0	1.04	2.0	10.7	4.0	6.6	2.0	1.495	6.0	0.38	5.0	4.679	5.0	34.0
ogooué-ivindo	3.5	5.0	0.499	0.0	1.12	10.0	13.0	4.0	3.2	0.0	1.321	2.0	-0.318	5.0	4.798	5.0	31.0
ogooué-lolo	15.5	20.0	0.604	0.0	1.06	4.0	18.6	4.0	7.4	2.0	1.24	2.0	-0.479	5.0	4.007	5.0	42.0
woleu-ntem	4.5	5.0	0.803	0.0	1.03	0.0	16.8	4.0	6.6	2.0	1.339	2.0	0.181	1.0	3.636	3.0	17.0

Ghana 1993, regions	Missing/flagged		Sex ratio		Age ratio		Height DPS		Weight DPS		SD WHZ		Skew WHZ		Kurtosis WHZ		Total score
	%	score	p-value	score	value	score	value	score	value	score	SD	score	value	score	value	score	score
ashanti	9.5	10.0	0.111	0.0	.	5.0	19.5	4.0	4.8	0.0	1.374	2.0	-0.541	5.0	3.795	3.0	29.0
brong-ahafo	5.5	10.0	0.671	0.0	.	5.0	17.3	4.0	8.2	2.0	1.444	6.0	-0.101	1.0	3.832	3.0	31.0
central	5.6	10.0	0.373	0.0	.	5.0	19.2	4.0	8.2	2.0	1.518	6.0	-0.434	5.0	2.92	0.0	32.0
eastern	9.0	10.0	0.687	0.0	.	5.0	8.8	2.0	10.5	4.0	1.487	6.0	0.451	5.0	3.036	0.0	32.0
greater accra	3.7	5.0	0.662	0.0	.	5.0	11.4	4.0	8.0	2.0	1.096	0.0	-0.387	5.0	2.852	0.0	21.0
northern	10.6	20.0	0.207	0.0	.	5.0	10.3	4.0	5.1	2.0	1.613	15.0	0.077	0.0	3.221	0.0	46.0
upper east	5.6	10.0	0.452	0.0	.	5.0	17.3	4.0	9.3	2.0	1.279	2.0	-0.454	5.0	3.912	5.0	33.0
upper west	7.0	10.0	0.722	0.0	.	5.0	25.2	10.0	13.5	4.0	1.135	0.0	-0.078	0.0	2.627	1.0	30.0
volta	9.3	10.0	0.008	4.0	.	5.0	14.9	4.0	4.8	0.0	1.325	2.0	-0.218	3.0	4.273	5.0	33.0
western	8.0	10.0	0.048	4.0	.	5.0	17.0	4.0	7.6	2.0	1.332	2.0	-0.185	1.0	2.625	1.0	29.0

Ghana 1998, regions	Missing/flagged		Sex ratio		Age ratio		Height DPS		Weight DPS		SD WHZ		Skew WHZ		Kurtosis WHZ		Total score
	%	score	p-value	score	value	score	value	score	value	score	SD	score	value	score	value	score	score
ashanti region	4.2	5.0	0.626	0.0	0.98	0.0	10.3	4.0	4.5	0.0	1.301	2.0	-0.096	0.0	4.303	5.0	16.0
brong ahafo region	5.6	10.0	1	0.0	0.68	10.0	15.3	4.0	8.3	2.0	1.239	2.0	0.342	5.0	4.734	5.0	38.0
central region	5.4	10.0	0.101	0.0	0.91	4.0	9.9	2.0	9.1	2.0	1.261	2.0	0.175	1.0	4.418	5.0	26.0
eastern region	1.5	0.0	0.555	0.0	0.93	4.0	17.9	4.0	5.5	2.0	1.166	0.0	0.225	3.0	4.053	5.0	18.0
greater accra region	3.9	5.0	0.582	0.0	1.06	4.0	21.5	10.0	7.0	2.0	1.215	2.0	0.436	5.0	5.261	5.0	33.0
northern region	4.1	5.0	0.731	0.0	0.83	10.0	13.3	4.0	5.6	2.0	1.263	2.0	-0.011	0.0	4.251	5.0	28.0
upper east region	2.1	0.0	0.957	0.0	0.72	10.0	8.7	2.0	6.4	2.0	1.215	2.0	-0.394	5.0	5.216	5.0	26.0
upper west region	10.7	20.0	0.073	2.0	0.87	10.0	16.8	4.0	8.4	2.0	1.267	2.0	-0.324	5.0	4.569	5.0	50.0

volta region	4.7	5.0	0.951	0.0	0.82	10.0	22.3	10.0	7.1	2.0	1.338	2.0	0.336	5.0	4.326	5.0	39.0
western region	3.8	5.0	0.869	0.0	0.98	0.0	14.9	4.0	5.6	2.0	1.417	6.0	0.343	5.0	4.202	5.0	27.0

Ghana 2003, regions	Missing/flagged		Sex ratio		Age ratio		Height DPS		Weight DPS		SD WHZ		Skew WHZ		Kurtosis WHZ		Total score
	%	score	p-value	score	value	score	value	score	value	score	SD	score	value	score	value	score	score
ashanti	3.2	5.0	0.632	0.0	0.86	10.0	10.1	4.0	7.2	2.0	1.286	2.0	-0.125	1.0	3.978	5.0	29.0
brong ahafo	4.6	5.0	0.382	0.0	0.87	10.0	14.0	4.0	8.1	2.0	1.207	2.0	0.077	0.0	4.988	5.0	28.0
central	3.7	5.0	0.253	0.0	.	5.0	20.4	10.0	10.8	4.0	1.231	2.0	0.419	5.0	4.18	5.0	36.0
eastern	4.6	5.0	0.414	0.0	1.06	4.0	15.5	4.0	4.3	0.0	1.298	2.0	-0.106	1.0	3.601	3.0	19.0
greater accra	7.5	10.0	0.537	0.0	0.94	4.0	12.5	4.0	5.7	2.0	1.451	6.0	-0.294	3.0	4.078	5.0	34.0
northern	6.6	10.0	0.258	0.0	0.92	4.0	13.1	4.0	5.3	2.0	1.363	2.0	-0.209	3.0	3.89	3.0	28.0
upper east	8.6	10.0	0.169	0.0	0.84	10.0	12.5	4.0	12.5	4.0	1.38	2.0	-0.005	0.0	3.974	5.0	35.0
upper west	7.4	10.0	0.322	0.0	0.98	0.0	8.0	2.0	11.2	4.0	1.691	15.0	0.075	0.0	2.853	0.0	31.0
volta	7.1	10.0	0.799	0.0	1.13	10.0	12.2	4.0	10.6	4.0	1.531	6.0	-0.434	5.0	4.108	5.0	44.0
western	2.7	5.0	0.341	0.0	0.93	4.0	18.6	4.0	8.3	2.0	1.359	2.0	0.441	5.0	4.513	5.0	27.0

Ghana 2008, regions	Missing/flagged		Sex ratio		Age ratio		Height DPS		Weight DPS		SD WHZ		Skew WHZ		Kurtosis WHZ		Total score
	%	score	p-value	score	value	score	value	score	value	score	SD	score	value	score	value	score	score
ashanti	5.1	10.0	0.768	0.0	.	5.0	15.6	4.0	5.3	2.0	1.332	2.0	0.007	0.0	3.945	5.0	28.0
brong ahafo	3.6	5.0	0.451	0.0	0.97	2.0	13.4	4.0	6.7	2.0	1.079	0.0	0.281	3.0	2.913	0.0	16.0
central	15.5	20.0	0.267	0.0	1.18	10.0	10.4	4.0	5.9	2.0	1.664	15.0	0.143	1.0	3.328	1.0	53.0
eastern	14.3	20.0	0.565	0.0	1.07	4.0	16.9	4.0	7.2	2.0	1.583	6.0	0.048	0.0	3.437	1.0	37.0
greater accra	16.7	20.0	0.538	0.0	0.78	10.0	17.7	4.0	6.8	2.0	1.332	2.0	0.524	5.0	4.824	5.0	48.0
northern	10.6	20.0	0.054	2.0	.	5.0	13.9	4.0	5.3	2.0	1.366	2.0	-0.007	0.0	4.018	5.0	40.0
upper east	17.5	20.0	0.734	0.0	0.91	4.0	22.0	10.0	10.0	4.0	1.431	6.0	-0.056	0.0	4.664	5.0	49.0
upper west	10.6	20.0	0.806	0.0	1.24	10.0	12.2	4.0	6.2	2.0	1.442	6.0	0.096	0.0	4.841	5.0	47.0
volta	5.3	10.0	0.604	0.0	0.97	0.0	14.2	4.0	4.4	0.0	1.421	6.0	0.305	5.0	4.914	5.0	30.0
western	12.3	20.0	0.901	0.0	1.02	0.0	11.7	4.0	2.7	0.0	1.42	6.0	-0.286	3.0	4.119	5.0	38.0

Guinea 1999, regions	Missing/flagged		Sex ratio		Age ratio		Height DPS		Weight DPS		SD WHZ		Skew WHZ		Kurtosis WHZ		Total score
	%	score	p-value	score	value	score	value	score	value	score	SD	score	value	score	value	score	score
central guinea	10.4	20.0	0.152	0.0	0.89	10.0	10.1	4.0	3.7	0.0	1.508	6.0	0.087	0.0	4.146	5.0	45.0
conakry	5.8	10.0	0.651	0.0	0.97	2.0	9.2	2.0	5.4	2.0	1.363	2.0	-0.058	0.0	4.375	5.0	23.0
forest guinea	7.6	10.0	0.603	0.0	0.86	10.0	23.1	10.0	2.4	0.0	1.352	2.0	-0.22	3.0	4.039	5.0	40.0
lower guinea	4.9	5.0	0.253	0.0	0.83	10.0	9.6	2.0	1.9	0.0	1.364	2.0	-0.381	5.0	3.749	3.0	27.0
upper guinea	9.3	10.0	0.001	4.0	0.93	4.0	21.0	10.0	4.8	0.0	1.469	6.0	0.113	1.0	4.066	5.0	40.0

Guinea 2005, regions	Missing/flagged		Sex ratio		Age ratio		Height DPS		Weight DPS		SD WHZ		Skew WHZ		Kurtosis WHZ		Total score
	%	score	p-value	score	value	score	value	score	value	score	SD	score	value	score	value	score	score
boké	4.7	5.0	0.766	0.0	0.77	10.0	22.8	10.0	5.6	2.0	1.325	2.0	0.126	1.0	4.591	5.0	35.0
conakry	16.2	20.0	0.514	0.0	0.88	10.0	27.8	10.0	7.8	2.0	1.542	6.0	-0.108	1.0	3.698	3.0	52.0
faranah	3.4	5.0	0.854	0.0	1.14	10.0	28.7	10.0	3.5	0.0	1.422	6.0	-0.21	3.0	3.998	5.0	39.0
kankan	4.2	5.0	0.893	0.0	0.9	4.0	12.3	4.0	6.5	2.0	1.506	6.0	0.167	1.0	3.755	3.0	25.0
kindia	4.0	5.0	0.755	0.0	0.91	4.0	16.6	4.0	3.7	0.0	1.383	2.0	-0.329	5.0	3.96	5.0	25.0
labé	4.9	5.0	0.212	0.0	0.89	10.0	23.1	10.0	6.3	2.0	1.679	15.0	0.291	3.0	3.854	3.0	48.0
mamou	4.0	5.0	0.497	0.0	0.91	4.0	25.9	10.0	5.0	0.0	1.352	2.0	0.525	5.0	4.944	5.0	31.0
n'zérékoré	2.5	0.0	0.127	0.0	0.92	4.0	27.0	10.0	6.9	2.0	1.526	6.0	-0.337	5.0	3.609	3.0	30.0

Guinea 2012, regions	Missing/flagged		Sex ratio		Age ratio		Height DPS		Weight DPS		SD WHZ		Skew WHZ		Kurtosis WHZ		Total score
	%	score	p-value	score	value	score	value	score	value	score	SD	score	value	score	value	score	score
Bok?	3.2	5.0	0.141	0.0	1.09	4.0	9.3	2.0	4.3	0.0	1.406	6.0	0.307	5.0	3.862	3.0	25.0
Conakry	6.7	10.0	0.374	0.0	.	5.0	15.6	4.0	7.2	2.0	1.312	2.0	-0.118	1.0	4.568	5.0	29.0
Faranah	4.2	5.0	0.547	0.0	0.87	10.0	10.3	4.0	2.4	0.0	1.285	2.0	-0.094	0.0	4.065	5.0	26.0
Kankan	4.8	5.0	0.267	0.0	0.96	2.0	9.5	2.0	6.7	2.0	1.534	6.0	-0.116	1.0	3.659	3.0	21.0
Kindia	4.8	5.0	0.442	0.0	0.93	4.0	14.3	4.0	5.2	2.0	1.437	6.0	-0.26	3.0	4.119	5.0	29.0
Lab?	6.7	10.0	0.584	0.0	0.95	4.0	12.7	4.0	5.4	2.0	1.381	2.0	-0.417	5.0	4.448	5.0	32.0
Mamou	4.3	5.0	0.542	0.0	0.84	10.0	30.7	10.0	5.8	2.0	1.391	2.0	-0.314	5.0	3.698	3.0	37.0
N'Z?r?kor?	4.5	5.0	0.58	0.0	0.83	10.0	9.0	2.0	4.5	0.0	1.332	2.0	-0.589	5.0	4.208	5.0	29.0

Liberia 2007, regions	Missing/flagged		Sex ratio		Age ratio		Height DPS		Weight DPS		SD WHZ		Skew WHZ		Kurtosis WHZ		Total score
	%	score	p-value	score	value	score	value	score	value	score	SD	score	value	score	value	score	score
monrovia	10.0	10.0	0.088	2.0	1.11	10.0	10.6	4.0	5.5	2.0	1.464	6.0	-0.168	1.0	3.941	5.0	40.0
north central	8.2	10.0	0.785	0.0	0.86	10.0	9.9	2.0	4.7	0.0	1.274	2.0	-0.381	5.0	4.558	5.0	34.0
north western	5.8	10.0	0.049	4.0	0.88	10.0	9.1	2.0	4.4	0.0	1.194	0.0	0.201	3.0	4.395	5.0	34.0
south central	6.3	10.0	0.89	0.0	0.94	4.0	10.8	4.0	2.7	0.0	1.243	2.0	-0.375	5.0	3.928	5.0	30.0
south eastern a	8.6	10.0	0.39	0.0	0.93	4.0	11.8	4.0	4.7	0.0	1.285	2.0	-0.323	5.0	4.315	5.0	30.0
south eastern b	8.9	10.0	0.95	0.0	0.82	10.0	14.1	4.0	3.7	0.0	1.496	6.0	-0.305	5.0	3.712	3.0	38.0

Mali 1995, regions	Missing/flagged		Sex ratio		Age ratio		Height DPS		Weight DPS		SD WHZ		Skew WHZ		Kurtosis WHZ		Total score

	%	score	p-value	score	value	score	value	score	value	score	SD	score	value	score	value	score	score
bamako	12.5	20.0	0.729	0.0	.	5.0	20.5	10.0	4.3	0.0	1.631	15.0	0.323	5.0	3.452	1.0	56.0
gao	3.4	5.0	0.334	0.0	.	5.0	15.3	4.0	8.3	2.0	1.532	6.0	0.286	3.0	3.522	1.0	26.0
kayes	10.6	20.0	0.916	0.0	.	5.0	14.5	4.0	5.2	2.0	1.327	2.0	-0.212	3.0	3.304	1.0	37.0
koulikoro	5.4	10.0	0.746	0.0	.	5.0	13.7	4.0	5.6	2.0	1.563	6.0	-0.052	0.0	3.042	0.0	27.0
mopti	8.5	10.0	1	0.0	.	5.0	38.7	10.0	9.2	2.0	1.63	15.0	0.134	1.0	2.92	0.0	43.0
sikasso	6.0	10.0	0.01	4.0	.	5.0	17.1	4.0	4.8	0.0	1.536	6.0	0.228	3.0	3.45	1.0	33.0
ségou	7.6	10.0	0.973	0.0	.	5.0	21.5	10.0	6.8	2.0	1.652	15.0	0.004	0.0	2.975	0.0	42.0
timbuktu	8.9	10.0	0.458	0.0	.	5.0	11.6	4.0	6.8	2.0	1.522	6.0	-0.151	1.0	2.422	1.0	29.0

Mali 2001, regions	Missing/flagged		Sex ratio		Age ratio		Height DPS		Weight DPS		SD WHZ		Skew WHZ		Kurtosis WHZ		Total score
	%	score	p-value	score	value	score	value	score	value	score	SD	score	value	score	value	score	score
bamako	5.6	10.0	0.312	0.0	1.02	0.0	17.0	4.0	3.4	0.0	1.232	2.0	-0.234	3.0	4.444	5.0	24.0
gao	3.8	5.0	0.269	0.0	1.12	10.0	19.9	4.0	4.7	0.0	1.329	2.0	-0.081	0.0	3.823	3.0	24.0
kayes	4.7	5.0	0.673	0.0	0.88	10.0	15.8	4.0	4.9	0.0	1.392	2.0	-0.01	0.0	3.969	5.0	26.0
kidal	4.1	5.0	0.937	0.0	1.42	10.0	20.8	10.0	11.0	4.0	1.168	0.0	-0.535	5.0	3.279	0.0	34.0
koulikoro	4.8	5.0	0.168	0.0	0.91	4.0	9.5	2.0	1.9	0.0	1.294	2.0	-0.1	1.0	3.632	3.0	17.0
mopti	6.0	10.0	0.791	0.0	0.91	4.0	13.5	4.0	2.2	0.0	1.337	2.0	-0.105	1.0	3.855	3.0	24.0
segou	9.9	10.0	0.778	0.0	0.88	10.0	7.6	2.0	5.1	2.0	1.603	15.0	0.055	0.0	3.429	1.0	40.0
sikasso	3.8	5.0	0.983	0.0	1.01	0.0	16.7	4.0	1.8	0.0	1.291	2.0	-0.047	0.0	3.778	3.0	14.0
tombouctou	12.3	20.0	0.88	0.0	0.82	10.0	11.6	4.0	5.3	2.0	1.599	6.0	0.273	3.0	3.719	3.0	48.0

Mali 2006, regions	Missing/flagged		Sex ratio		Age ratio		Height DPS		Weight DPS		SD WHZ		Skew WHZ		Kurtosis WHZ		Total score
	%	score	p-value	score	value	score	value	score	value	score	SD	score	value	score	value	score	score
bamako	8.7	10.0	0.545	0.0	1.01	0.0	23.0	10.0	5.3	2.0	1.499	6.0	0.252	3.0	4.195	5.0	36.0
gao	14.4	20.0	0.54	0.0	0.93	4.0	18.3	4.0	5.7	2.0	1.712	15.0	0.295	3.0	3.651	3.0	51.0
kayes	5.4	10.0	0.84	0.0	1.02	0.0	20.6	10.0	4.1	0.0	1.618	15.0	0.329	5.0	3.805	3.0	43.0
kidal	8.8	10.0	0.723	0.0	0.83	10.0	25.0	10.0	8.1	2.0	1.816	15.0	0.088	0.0	2.828	0.0	47.0
koulikoro	5.5	10.0	0.729	0.0	0.93	4.0	19.6	4.0	5.0	0.0	1.581	6.0	-0.076	0.0	3.706	3.0	27.0
mopti	12.2	20.0	0.7	0.0	0.87	10.0	28.6	10.0	6.3	2.0	1.558	6.0	0.068	0.0	3.811	3.0	51.0
segou	6.6	10.0	0.587	0.0	1.04	2.0	24.3	10.0	3.9	0.0	1.477	6.0	0.097	0.0	3.877	3.0	31.0
sikasso	6.6	10.0	0.197	0.0	1.04	2.0	26.3	10.0	2.6	0.0	1.528	6.0	0.019	0.0	3.864	3.0	31.0
tombouctou	7.9	10.0	0.813	0.0	0.72	10.0	41.4	10.0	11.0	4.0	1.72	15.0	0.379	5.0	3.468	1.0	55.0

Niger 1992, regions	Missing/flagged		Sex ratio		Age ratio		Height DPS		Weight DPS		SD WHZ		Skew WHZ		Kurtosis WHZ		Total score
	%	score	p-value	score	value	score	value	score	value	score	SD	score	value	score	value	score	score
agadez	7.4	10.0	0.631	0.0	1.07	4.0	20.4	10.0	8.3	2.0	1.534	6.0	0.191	1.0	3.852	3.0	36.0
diffa	10.2	20.0	0.149	0.0	0.84	10.0	18.6	4.0	7.1	2.0	1.675	15.0	0.679	5.0	4.207	5.0	61.0
dosso	12.3	20.0	0.054	2.0	0.95	4.0	6.3	2.0	4.0	0.0	1.167	0.0	0.075	0.0	5.201	5.0	33.0
maradi	13.6	20.0	0.542	0.0	0.92	4.0	6.9	2.0	6.3	2.0	1.408	6.0	-0.096	0.0	3.283	0.0	34.0
niamey	5.6	10.0	0.546	0.0	0.87	10.0	15.5	4.0	4.4	0.0	1.318	2.0	0.196	1.0	4.161	5.0	32.0
tahoua	13.5	20.0	0.375	0.0	0.98	0.0	11.7	4.0	3.4	0.0	1.335	2.0	0.107	1.0	4.091	5.0	32.0
tillabéri	5.9	10.0	0.135	0.0	1.05	4.0	9.6	2.0	3.4	0.0	1.25	2.0	-0.193	1.0	3.315	1.0	20.0
zinder	8.4	10.0	0.013	4.0	1.02	0.0	10.2	4.0	2.9	0.0	1.498	6.0	-0.012	0.0	3.413	1.0	25.0

Niger 1998, regions	Missing/flagged		Sex ratio		Age ratio		Height DPS		Weight DPS		SD WHZ		Skew WHZ		Kurtosis WHZ		Total score
	%	score	p-value	score	value	score	value	score	value	score	SD	score	value	score	value	score	score
dosso	3.2	5.0	0.24	0.0	.	5.0	8.5	2.0	4.8	0.0	1.217	2.0	0.123	1.0	3.504	1.0	16.0
maradi	5.8	10.0	0.036	4.0	.	5.0	11.6	4.0	2.7	0.0	1.371	2.0	0.129	1.0	3.701	3.0	29.0
niamey	6.5	10.0	0.806	0.0	.	5.0	12.9	4.0	6.6	2.0	1.361	2.0	0.145	1.0	3.648	3.0	27.0
tahoua/agadez	5.6	10.0	0.6	0.0	.	5.0	11.2	4.0	3.3	0.0	1.275	2.0	-0.047	0.0	3.135	0.0	21.0
tillaberi	3.2	5.0	0.262	0.0	.	5.0	10.5	4.0	3.7	0.0	1.312	2.0	-0.005	0.0	3.524	1.0	17.0
zinda/diffa	6.2	10.0	0.451	0.0	.	5.0	14.1	4.0	5.0	2.0	1.458	6.0	0.074	0.0	3.026	0.0	27.0

Niger 2006, regions	Missing/flagged		Sex ratio		Age ratio		Height DPS		Weight DPS		SD WHZ		Skew WHZ		Kurtosis WHZ		Total score
	%	score	p-value	score	value	score	value	score	value	score	SD	score	value	score	value	score	score
agadez	14.4	20.0	0.467	0.0	.	5.0	36.0	10.0	5.0	2.0	1.425	6.0	0.383	5.0	4.849	5.0	53.0
diffa	4.0	5.0	0.97	0.0	0.9	10.0	24.0	10.0	6.1	2.0	1.324	2.0	0.162	1.0	4.67	5.0	35.0
dosso	2.0	0.0	0.955	0.0	0.87	10.0	37.6	10.0	3.1	0.0	1.296	2.0	-0.304	5.0	4.377	5.0	32.0
maradi	6.8	10.0	0.822	0.0	0.84	10.0	30.5	10.0	5.5	2.0	1.408	6.0	-0.106	1.0	3.683	3.0	42.0
niamey	6.0	10.0	0.397	0.0	1.06	4.0	34.3	10.0	4.4	0.0	1.312	2.0	0.305	5.0	4.738	5.0	36.0
tahoua	5.6	10.0	0.243	0.0	0.94	4.0	29.4	10.0	2.8	0.0	1.403	6.0	0.077	0.0	4.376	5.0	35.0
tillabéri	8.5	10.0	0.12	0.0	0.98	0.0	14.1	4.0	4.8	0.0	1.399	2.0	0.375	5.0	4.393	5.0	26.0
zinder	9.5	10.0	0.367	0.0	0.85	10.0	19.1	4.0	5.0	2.0	1.595	6.0	0.351	5.0	3.505	1.0	38.0

Niger 2012, regions	Missing/flagged		Sex ratio		Age ratio		Height DPS		Weight DPS		SD WHZ		Skew WHZ		Kurtosis WHZ		Total score
	%	score	p-value	score	value	score	value	score	value	score	SD	score	value	score	value	score	score
Agadez	16.6	20.0	0.533	0.0	1.01	0.0	57.7	10.0	12.3	4.0	1.841	15.0	0.261	3.0	3.18	0.0	52.0
Diffa	32.2	20.0	0.975	0.0	0.81	10.0	30.3	10.0	30.4	10.0	1.859	15.0	0.375	5.0	3.196	0.0	70.0

Dosso	3.0	5.0	0.481	0.0	0.86	10.0	14.5	4.0	3.5	0.0	1.206	2.0	0.165	1.0	4.318	5.0	27.0
Maradi	12.1	20.0	0.837	0.0	0.83	10.0	37.2	10.0	4.5	0.0	1.597	6.0	0.223	3.0	3.772	3.0	52.0
Niamey	13.6	20.0	0.872	0.0	1.13	10.0	36.0	10.0	9.2	2.0	1.341	2.0	0.269	3.0	4.865	5.0	52.0
Tahoua	13.1	20.0	0.836	0.0	0.86	10.0	26.2	10.0	14.2	4.0	1.517	6.0	0.097	0.0	4.011	5.0	55.0
Tillaberi	3.8	5.0	0.04	4.0	0.92	4.0	10.4	4.0	3.0	0.0	1.152	0.0	0.126	1.0	3.777	3.0	21.0
Zinder	12.9	20.0	0.827	0.0	0.95	2.0	47.4	10.0	11.1	4.0	1.39	2.0	-0.137	1.0	3.478	1.0	40.0

Nigeria 1990, regions	Missing/flagged		Sex ratio		Age ratio		Height DPS		Weight DPS		SD WHZ		Skew WHZ		Kurtosis WHZ		Total score
	%	score	p-value	score	value	score	value	score	value	score	SD	score	value	score	value	score	score
northeast	14.8	20.0	0.658	0.0	1.02	0.0	13.1	4.0	10.4	4.0	1.491	6.0	-0.277	3.0	3.553	1.0	38.0
northwest	10.3	20.0	0.959	0.0	1.03	2.0	13.9	4.0	7.9	2.0	1.479	6.0	-0.231	3.0	3.731	3.0	40.0
southeast	4.1	5.0	0.538	0.0	0.78	10.0	10.4	4.0	7.8	2.0	1.202	2.0	-0.077	0.0	4.267	5.0	28.0
southwest	5.5	10.0	0.496	0.0	0.85	10.0	12.6	4.0	5.7	2.0	1.188	0.0	-0.101	1.0	4.406	5.0	32.0

Nigeria 2003, regions	Missing/flagged		Sex ratio		Age ratio		Height DPS		Weight DPS		SD WHZ		Skew WHZ		Kurtosis WHZ		Total score
	%	score	p-value	score	value	score	value	score	value	score	SD	score	value	score	value	score	score
north central	4.2	5.0	0.327	0.0	0.95	4.0	20.3	10.0	3.5	0.0	1.341	2.0	-0.38	5.0	4.353	5.0	31.0
north east	4.5	5.0	0.577	0.0	1.02	0.0	19.0	4.0	2.0	0.0	1.458	6.0	0.047	0.0	4	5.0	20.0
north west	14.3	20.0	0.775	0.0	1.08	4.0	27.2	10.0	5.4	2.0	1.772	15.0	0.003	0.0	3.276	0.0	51.0
south east	8.6	10.0	0.35	0.0	0.96	2.0	19.9	4.0	5.4	2.0	1.375	2.0	-0.008	0.0	4.049	5.0	25.0
south south	8.6	10.0	0.489	0.0	0.99	0.0	20.1	10.0	8.5	2.0	1.508	6.0	-0.017	0.0	3.923	5.0	33.0
south west	4.5	5.0	0.065	2.0	0.84	10.0	25.2	10.0	5.5	2.0	1.409	6.0	-0.008	0.0	3.428	1.0	36.0

Nigeria 2008, regions	Missing/flagged		Sex ratio		Age ratio		Height DPS		Weight DPS		SD WHZ		Skew WHZ		Kurtosis WHZ		Total score
	%	score	p-value	score	value	score	value	score	value	score	SD	score	value	score	value	score	score
north central	19.4	20.0	0.406	0.0	0.9	10.0	36.0	10.0	6.6	2.0	1.768	15.0	-0.237	3.0	3.603	3.0	63.0
north east	20.9	20.0	0.782	0.0	0.99	0.0	21.8	10.0	6.3	2.0	2.013	15.0	0.005	0.0	2.872	0.0	47.0
north west	26.9	20.0	0.523	0.0	1.02	0.0	29.0	10.0	10.0	2.0	2.006	15.0	0.008	0.0	2.819	0.0	47.0
south east	17.1	20.0	0.506	0.0	0.99	0.0	30.1	10.0	4.8	0.0	1.583	6.0	0.071	0.0	3.993	5.0	41.0
south south	17.2	20.0	0.432	0.0	0.97	2.0	26.2	10.0	3.6	0.0	1.617	15.0	0.101	1.0	3.81	3.0	51.0
south west	11.4	20.0	0.091	2.0	0.94	4.0	22.1	10.0	4.2	0.0	1.614	15.0	-0.141	1.0	3.942	5.0	57.0

Sao Tome et Principe 2008, regions	Missing/flagged		Sex ratio		Age ratio		Height DPS		Weight DPS		SD WHZ		Skew WHZ		Kurtosis WHZ		Total score
	%	score	p-value	score	value	score	value	score	value	score	SD	score	value	score	value	score	score
região centro	30.0	20.0	0.452	0.0	0.85	10.0	56.0	10.0	76.7	10.0	1.705	15.0	0.022	0.0	3.358	1.0	66.0
região do principe	5.9	10.0	0.816	0.0	0.99	0.0	42.2	10.0	71.1	10.0	1.519	6.0	0.26	3.0	3.805	3.0	42.0
região norte	12.3	20.0	0.453	0.0	0.94	4.0	78.8	10.0	81.4	10.0	1.794	15.0	-0.24	3.0	3.237	0.0	62.0
região sul	15.0	20.0	0.08	2.0	0.98	0.0	82.5	10.0	81.2	10.0	1.999	15.0	0.107	1.0	3.079	0.0	58.0

Senegal 1992, regions	Missing/flagged		Sex ratio		Age ratio		Height DPS		Weight DPS		SD WHZ		Skew WHZ		Kurtosis WHZ		Total score
	%	score	p-value	score	value	score	value	score	value	score	SD	score	value	score	value	score	score
central	6.3	10.0	0.176	0.0	0.83	10.0	11.1	4.0	2.9	0.0	1.366	2.0	0.064	0.0	4.024	5.0	31.0
north east	8.9	10.0	0.455	0.0	0.86	10.0	11.9	4.0	2.5	0.0	1.406	6.0	0.06	0.0	4.175	5.0	35.0
south	6.7	10.0	0.686	0.0	0.78	10.0	6.8	2.0	4.6	0.0	1.347	2.0	-0.311	5.0	3.824	3.0	32.0
west	5.7	10.0	0.009	4.0	0.86	10.0	8.2	2.0	3.2	0.0	1.241	2.0	-0.087	0.0	4.118	5.0	33.0

Senegal 2005, regions	Missing/flagged		Sex ratio		Age ratio		Height DPS		Weight DPS		SD WHZ		Skew WHZ		Kurtosis WHZ		Total score
	%	score	p-value	score	value	score	value	score	value	score	SD	score	value	score	value	score	score
dakar	10.0	10.0	0.069	2.0	0.91	4.0	17.2	4.0	5.7	2.0	1.157	0.0	-0.323	5.0	3.179	0.0	27.0
diourbel	6.4	10.0	0.855	0.0	1.18	10.0	12.5	4.0	7.0	2.0	1.212	2.0	0.101	1.0	3.626	3.0	32.0
fatick	5.3	10.0	0.502	0.0	1.08	4.0	12.1	4.0	4.4	0.0	1.139	0.0	-0.245	3.0	3.084	0.0	21.0
kaolack	5.1	10.0	0.148	0.0	1.07	4.0	10.5	4.0	6.2	2.0	1.142	0.0	-0.215	3.0	3.812	3.0	26.0
kolda	1.2	0.0	0.8	0.0	1.03	2.0	19.3	4.0	6.3	2.0	1.207	2.0	-0.145	1.0	4.128	5.0	16.0
louga	12.7	20.0	0.553	0.0	1.09	4.0	13.6	4.0	4.8	0.0	1.289	2.0	0.203	3.0	4.511	5.0	38.0
matam	15.4	20.0	0.106	0.0	0.94	4.0	9.2	2.0	6.5	2.0	1.299	2.0	0.086	0.0	3.271	0.0	30.0
saint-louis	14.2	20.0	0.241	0.0	0.95	4.0	9.2	2.0	5.3	2.0	1.199	0.0	-0.123	1.0	3.332	1.0	30.0
tambacounda	6.3	10.0	0.218	0.0	1	0.0	12.6	4.0	4.4	0.0	1.393	2.0	0.655	5.0	5.093	5.0	26.0
thiès	5.3	10.0	0.016	4.0	0.82	10.0	6.6	2.0	5.5	2.0	1.155	0.0	0.043	0.0	4.749	5.0	33.0
zuguinchor	3.7	5.0	0.041	4.0	1.03	0.0	20.5	10.0	7.4	2.0	1.171	0.0	-0.085	0.0	4.072	5.0	26.0

Senegal 2010, regions	Missing/flagged		Sex ratio		Age ratio		Height DPS		Weight DPS		SD WHZ		Skew WHZ		Kurtosis WHZ		Total score
	%	score	p-value	score	value	score	value	score	value	score	SD	score	value	score	value	score	score
dakar	8.6	10.0	0.586	0.0	0.8	10.0	19.7	4.0	3.6	0.0	1.201	2.0	0.855	5.0	4.66	5.0	36.0
diourbel	18.6	20.0	0.705	0.0	0.88	10.0	27.2	10.0	9.7	2.0	1.242	2.0	0.91	5.0	5.748	5.0	54.0
fatick	7.7	10.0	0.784	0.0	0.92	4.0	32.8	10.0	5.5	2.0	1.214	2.0	-0.189	1.0	4.616	5.0	34.0
kaffrine	4.2	5.0	0.349	0.0	1.03	2.0	30.2	10.0	4.2	0.0	1.38	2.0	0.726	5.0	4.94	5.0	29.0
kaolack	11.4	20.0	0.37	0.0	0.9	10.0	26.5	10.0	7.0	2.0	1.214	2.0	0.289	3.0	5.702	5.0	52.0
kedougou	24.3	20.0	0.96	0.0	0.79	10.0	15.3	4.0	9.2	2.0	1.127	0.0	0.141	1.0	5.391	5.0	42.0
kolda	6.6	10.0	0.367	0.0	0.94	4.0	22.6	10.0	4.4	0.0	1.169	0.0	-0.029	0.0	3.679	3.0	27.0

louga	13.7	20.0	0.428	0.0	0.9	10.0	23.0	10.0	4.1	0.0	1.452	6.0	0.606	5.0	4.804	5.0	56.0
matam	19.2	20.0	0.153	0.0	0.95	4.0	21.3	10.0	4.6	0.0	1.403	6.0	0.548	5.0	4.617	5.0	50.0
saint-louis	18.9	20.0	0.279	0.0	0.89	10.0	33.3	10.0	7.4	2.0	1.199	0.0	-0.207	3.0	3.23	0.0	45.0
sedhiou	7.8	10.0	0.004	4.0	1.06	4.0	26.2	10.0	3.5	0.0	1.267	2.0	0.626	5.0	4.323	5.0	40.0
tambacounda	19.8	20.0	0.741	0.0	1.01	0.0	13.8	4.0	6.0	2.0	1.257	2.0	0.122	1.0	3.975	5.0	34.0
thiès	11.5	20.0	0.566	0.0	0.9	4.0	14.6	4.0	9.4	2.0	1.389	2.0	-0.18	1.0	3.901	5.0	38.0
ziguinchor	3.3	5.0	0.834	0.0	1.17	10.0	26.2	10.0	7.2	2.0	1.169	0.0	0.152	1.0	4.891	5.0	33.0

**Sierra Leone 2008, regions**	**Missing/flagged**		**Sex ratio**		**Age ratio**		**Height DPS**		**Weight DPS**		**SD WHZ**		**Skew WHZ**		**Kurtosis WHZ**		**Total score**
	**%**	**score**	**p-value**	**score**	**value**	**score**	**value**	**score**	**value**	**score**	**SD**	**score**	**value**	**score**	**value**	**score**	**score**
eastern	14.3	20.0	0.192	0.0	1.07	4.0	11.7	4.0	6.4	2.0	1.824	15.0	0.171	1.0	3.446	1.0	47.0
northern	12.5	20.0	0.524	0.0	1.25	10.0	20.1	10.0	4.2	0.0	1.512	6.0	0.144	1.0	4.196	5.0	52.0
southern	17.2	20.0	0.954	0.0	1.09	4.0	24.4	10.0	7.6	2.0	2.021	15.0	0.246	3.0	2.935	0.0	54.0
western	13.6	20.0	0.194	0.0	1.15	10.0	12.4	4.0	7.0	2.0	1.689	15.0	0.313	5.0	3.696	3.0	59.0

**Togo 1998, regions**	**Missing/flagged**		**Sex ratio**		**Age ratio**		**Height DPS**		**Weight DPS**		**SD WHZ**		**Skew WHZ**		**Kurtosis WHZ**		**Total score**
	**%**	**score**	**p-value**	**score**	**value**	**score**	**value**	**score**	**value**	**score**	**SD**	**score**	**value**	**score**	**value**	**score**	**score**
centrale	4.6	5.0	0.57	0.0	.	5.0	9.5	2.0	4.1	0.0	1.364	2.0	-0.105	1.0	3.266	0.0	15.0
kara	3.3	5.0	0.968	0.0	.	5.0	13.6	4.0	3.8	0.0	1.329	2.0	0.025	0.0	3.206	0.0	16.0
lomé	5.9	10.0	1	0.0	.	5.0	30.5	10.0	5.3	2.0	1.353	2.0	-0.163	1.0	4.385	5.0	35.0
marities	3.7	5.0	0.826	0.0	.	5.0	9.4	2.0	6.3	2.0	1.37	2.0	0.345	5.0	4.502	5.0	26.0
plateaux	5.3	10.0	0.274	0.0	.	5.0	14.0	4.0	2.8	0.0	1.344	2.0	-0.15	1.0	3.739	3.0	25.0
savanes	4.2	5.0	0.614	0.0	.	5.0	8.2	2.0	2.5	0.0	1.374	2.0	0.293	3.0	3.566	1.0	18.0

Summary of anthropometric data quality indicators and total data quality score, NNS

Benin 2008, regions	Missing/flagged		Sex ratio		Age ratio		Height DPS		Weight DPS		SD WHZ		Skew WHZ		Kurtosis WHZ		Total score
	%	score	p-value	score	value	score	value	score	value	score	SD	score	value	score	value	score	score
alibori	8.9	10.0	0.455	0.0	0.75	10.0	10.8	4.0	4.8	0.0	1.092	0.0	-0.27	3.0	3.994	5.0	32.0
atacora	6.5	10.0	0.835	0.0	0.85	10.0	21.6	10.0	5.1	2.0	1.157	0.0	0.101	1.0	3.787	3.0	36.0
atlantique	1.3	0.0	0.111	0.0	0.89	10.0	7.3	2.0	6.3	2.0	1.028	0.0	-0.137	1.0	2.819	0.0	15.0
borgou	4.5	5.0	0.411	0.0	1.01	0.0	8.9	2.0	6.3	2.0	1.164	0.0	-0.62	5.0	3.987	5.0	19.0
collines	5.4	10.0	0.457	0.0	1.04	2.0	28.9	10.0	6.5	2.0	1.14	0.0	-0.45	5.0	4.854	5.0	34.0
couffo	1.5	0.0	0.192	0.0	0.75	10.0	6.8	2.0	5.2	2.0	1.074	0.0	-0.201	3.0	3.942	5.0	22.0
donga	3.8	5.0	0.951	0.0	0.89	10.0	21.0	10.0	6.7	2.0	1.171	0.0	-0.215	3.0	5.933	5.0	35.0
littoral	1.8	0.0	0.124	0.0	0.8	10.0	13.1	4.0	12.4	4.0	1.152	0.0	0.394	5.0	4.369	5.0	28.0
mono	3.3	5.0	0.406	0.0	0.77	10.0	6.2	2.0	7.3	2.0	0.953	0.0	-0.176	1.0	3.687	3.0	23.0
oueme	7.4	10.0	0.028	4.0	.	5.0	10.2	4.0	7.1	2.0	1.031	0.0	0.25	3.0	3.647	3.0	31.0
plateau	5.1	10.0	0.215	0.0	0.78	10.0	7.8	2.0	5.2	2.0	0.951	0.0	-0.198	1.0	3.517	1.0	26.0
zou	3.7	5.0	0.247	0.0	0.92	4.0	10.9	4.0	6.5	2.0	0.955	0.0	0.021	0.0	3.351	1.0	16.0

Burkina Faso 2012, regions	Missing/flagged		Sex ratio		Age ratio		Height DPS		Weight DPS		SD WHZ		Skew WHZ		Kurtosis WHZ		Total score
	%	score	p-value	score	value	score	value	score	value	score	SD	score	value	score	value	score	score
bales	0.3	0.0	0.409	0.0	1.03	0.0	1.0	0.0	1.6	0.0	1.039	0.0	-0.052	0.0	2.973	0.0	0.0
bam	2.3	0.0	0.512	0.0	1.17	10.0	3.0	0.0	1.4	0.0	1.146	0.0	-0.003	0.0	3.209	0.0	10.0
banwa	1.9	0.0	0.186	0.0	1.21	10.0	1.7	0.0	1.6	0.0	1.087	0.0	-0.161	1.0	3.663	3.0	14.0
banwa ganzourgou	2.8	5.0	0.584	0.0	1.07	4.0	3.2	0.0	2.5	0.0	0.976	0.0	-0.065	0.0	3.767	3.0	12.0
bazega	1.0	0.0	0.086	2.0	1.12	10.0	3.1	0.0	1.4	0.0	1.039	0.0	-0.298	3.0	3.327	1.0	16.0
boulgou	0.9	0.0	0.675	0.0	0.81	10.0	4.3	0.0	2.7	0.0	1.087	0.0	0.015	0.0	3.172	0.0	10.0
cascades	0.8	0.0	0.937	0.0	1.08	4.0	2.0	0.0	2.4	0.0	1.087	0.0	-0.132	1.0	2.843	0.0	5.0
centre ouest	0.3	0.0	0.562	0.0	1.14	10.0	3.1	0.0	2.6	0.0	1.03	0.0	-0.491	5.0	3.657	3.0	18.0
est	0.3	0.0	0.598	0.0	1.01	0.0	2.0	0.0	1.5	0.0	1.04	0.0	-0.068	0.0	2.767	0.0	0.0
houet	3.5	5.0	0.685	0.0	0.95	2.0	2.9	0.0	1.2	0.0	1.116	0.0	-0.131	1.0	3.307	1.0	9.0
kadiogo	3.2	5.0	0.116	0.0	0.86	10.0	4.6	0.0	4.1	0.0	1.067	0.0	0.066	0.0	2.761	0.0	15.0
kenedougou	0.1	0.0	0.038	4.0	1.2	10.0	3.0	0.0	2.8	0.0	1.066	0.0	-0.206	3.0	3.1	0.0	17.0
kossi	1.9	0.0	0.561	0.0	1.12	10.0	4.6	0.0	1.4	0.0	1.136	0.0	-0.171	1.0	3.084	0.0	11.0
koulpelogo	1.2	0.0	0.835	0.0	0.84	10.0	1.9	0.0	3.4	0.0	1.036	0.0	0.111	1.0	3.926	5.0	16.0
kouritenga	0.3	0.0	0.06	2.0	1.06	4.0	5.3	2.0	3.2	0.0	1.004	0.0	0.108	1.0	3.89	3.0	12.0
kourweogo	0.9	0.0	0.161	0.0	1.02	0.0	2.5	0.0	2.3	0.0	1.051	0.0	-0.004	0.0	2.934	0.0	0.0
mouhoun	0.6	0.0	0.407	0.0	1.07	4.0	3.2	0.0	4.5	0.0	1.052	0.0	-0.108	1.0	2.944	0.0	5.0
nahouri	0.2	0.0	0.788	0.0	0.82	10.0	1.5	0.0	1.6	0.0	0.986	0.0	-0.164	1.0	2.976	0.0	11.0
namentenga	1.0	0.0	0.086	2.0	1.38	10.0	4.6	0.0	1.9	0.0	1.114	0.0	-0.074	0.0	3.411	1.0	13.0
nayala	5.7	10.0	0.3	0.0	0.97	2.0	4.8	0.0	3.8	0.0	1.097	0.0	-0.066	0.0	2.932	0.0	12.0
nord	1.9	0.0	0.283	0.0	0.87	10.0	2.5	0.0	2.1	0.0	1.012	0.0	-0.067	0.0	3.08	0.0	10.0
oubritenga	2.7	5.0	0.44	0.0	1.02	0.0	3.6	0.0	1.7	0.0	1.043	0.0	-0.246	3.0	3.452	1.0	9.0
sahel	2.0	0.0	0.005	4.0	1.15	10.0	1.6	0.0	0.9	0.0	1.138	0.0	-0.16	1.0	3.036	0.0	15.0
sanmentenga	0.6	0.0	0.307	0.0	1.1	4.0	3.4	0.0	1.7	0.0	1.037	0.0	-0.115	1.0	2.839	0.0	5.0
sourou	0.5	0.0	0.815	0.0	1.15	10.0	5.5	2.0	3.5	0.0	1.032	0.0	-0.032	0.0	3.136	0.0	12.0
sud ouest	3.1	5.0	0.895	0.0	1.27	10.0	2.2	0.0	3.4	0.0	1.102	0.0	-0.159	1.0	3.017	0.0	16.0
tuy	0.9	0.0	0.01	4.0	1.11	10.0	0.8	0.0	0.8	0.0	1.079	0.0	-0.091	0.0	3.175	0.0	14.0
zoundweogo	3.5	5.0	0.102	0.0	0.9	10.0	0.6	0.0	0.5	0.0	1.024	0.0	-0.017	0.0	3.883	3.0	18.0

Cameroon 2011, regions	Missing/flagged		Sex ratio		Age ratio		Height DPS		Weight DPS		SD WHZ		Skew WHZ		Kurtosis WHZ		Total score
	%	score	p-value	score	value	score	value	score	value	score	SD	score	value	score	value	score	score
extrême-nord	11.0	20.0	0.969	0.0	0.82	10.0	6.2	2.0	3.5	0.0	1.055	0.0	-0.391	5.0	3.502	1.0	38.0
nord	12.5	20.0	0.878	0.0	0.86	10.0	12.4	4.0	4.1	0.0	1.29	2.0	-0.042	0.0	3.754	3.0	39.0

Central African Rep 2012, regions	Missing/flagged		Sex ratio		Age ratio		Height DPS		Weight DPS		SD WHZ		Skew WHZ		Kurtosis WHZ		Total score
	%	score	p-value	score	value	score	value	score	value	score	SD	score	value	score	value	score	score

bamingui bangoran	4.1	5.0	0.843	0.0	0.95	2.0	10.8	4.0	2.7	0.0	1.126	0.0	-0.071	0.0	3.74	3.0	14.0
bangui	0.6	0.0	0.916	0.0	.	5.0	6.0	2.0	3.6	0.0	1.075	0.0	0.247	3.0	4.084	5.0	15.0
basse kotto	2.7	5.0	0.745	0.0	0.9	4.0	3.5	0.0	2.4	0.0	1.178	0.0	-0.121	1.0	4.213	5.0	15.0
haut kotto	0.3	0.0	0.2	0.0	1.26	10.0	7.9	2.0	3.4	0.0	1.098	0.0	0.011	0.0	3.701	3.0	15.0
haut mbomou	0.5	0.0	0.399	0.0	1.12	10.0	2.9	0.0	2.5	0.0	1.157	0.0	0.055	0.0	4.235	5.0	15.0
kemo	0.9	0.0	0.109	0.0	1.39	10.0	4.5	0.0	2.8	0.0	1.225	2.0	-0.269	3.0	3.726	3.0	18.0
lobaye	0.6	0.0	0.694	0.0	1.1	10.0	8.0	2.0	3.6	0.0	1.079	0.0	0.397	5.0	4.788	5.0	22.0
mambere kadei	2.7	5.0	0.89	0.0	0.92	4.0	5.2	2.0	3.4	0.0	1.123	0.0	-0.169	1.0	3.63	3.0	15.0
mbomou	0.4	0.0	0.191	0.0	1.29	10.0	8.7	2.0	2.6	0.0	1.213	2.0	-0.13	1.0	3.745	3.0	18.0
nana grebizi	0.5	0.0	0.521	0.0	1.29	10.0	5.1	2.0	2.2	0.0	1.143	0.0	-0.171	1.0	3.382	1.0	14.0
nana mambere	2.0	0.0	0.172	0.0	0.98	0.0	6.3	2.0	5.8	2.0	1.165	0.0	-0.117	1.0	3.78	3.0	8.0
ombella mpoko	1.9	0.0	0.17	0.0	1.22	10.0	9.2	2.0	3.1	0.0	1.109	0.0	-0.039	0.0	2.98	0.0	12.0
ouaka	3.4	5.0	0.186	0.0	1.1	4.0	8.1	2.0	3.7	0.0	1.159	0.0	0.11	1.0	3.854	3.0	15.0
ouham	2.6	5.0	0.447	0.0	1.16	10.0	9.3	2.0	3.6	0.0	1.187	0.0	-0.052	0.0	4.08	5.0	22.0
ouham pende	3.0	5.0	0.243	0.0	1.62	10.0	4.3	0.0	3.8	0.0	1.19	0.0	0.243	3.0	4.019	5.0	23.0
sangha mbarere	0.6	0.0	0.649	0.0	1.07	4.0	8.3	2.0	3.2	0.0	1.121	0.0	-0.151	1.0	3.879	3.0	10.0
vakaga	1.6	0.0	0.859	0.0	.	5.0	6.9	2.0	5.1	2.0	1.079	0.0	-0.28	3.0	3.586	1.0	13.0

Chad June 2012, regions	Missing/flagged		Sex ratio		Age ratio		Height DPS		Weight DPS		SD WHZ		Skew WHZ		Kurtosis WHZ		Total score
	%	score	p-value	score	value	score	value	score	value	score	SD	score	value	score	value	score	score
Barh El Ghazal	4.5	5.0	0.913	0.0	1.01	0.0	2.9	0.0	3.1	0.0	1.071	0.0	-0.172	1.0	3.409	1.0	7.0
Batha	8.2	10.0	0.164	0.0	0.87	10.0	4.3	0.0	1.9	0.0	1.009	0.0	0.422	5.0	4.953	5.0	30.0
Guéra	7.0	10.0	0.05	4.0	0.91	4.0	2.2	0.0	1.9	0.0	1.042	0.0	0.168	1.0	4.186	5.0	24.0
Hadjer Lamis	4.6	5.0	0.905	0.0	1	0.0	5.2	2.0	2.5	0.0	1.112	0.0	-0.063	0.0	3.371	1.0	8.0
Kanem	6.5	10.0	0.775	0.0	0.93	4.0	7.5	2.0	6.1	2.0	1.031	0.0	0.283	3.0	3.122	0.0	21.0
Lac	7.5	10.0	0.615	0.0	1.16	10.0	3.0	0.0	4.1	0.0	1.103	0.0	0.241	3.0	3.961	5.0	28.0
N'Djamena	8.0	10.0	0.972	0.0	1.03	2.0	8.2	2.0	2.4	0.0	1.106	0.0	0.069	0.0	3.277	0.0	14.0
Ouaddai	2.2	0.0	0.254	0.0	1.08	4.0	2.8	0.0	4.1	0.0	1.037	0.0	-0.049	0.0	3.641	3.0	7.0
Salamat	8.2	10.0	0.038	4.0	0.84	10.0	8.6	2.0	2.6	0.0	1.136	0.0	0.218	3.0	4.434	5.0	34.0
Sila	11.2	20.0	0.564	0.0	1.02	0.0	3.5	0.0	2.8	0.0	1.096	0.0	0.089	0.0	3.769	3.0	23.0
Wadi Fira	3.5	5.0	0.124	0.0	0.93	4.0	7.4	2.0	3.7	0.0	1.044	0.0	0.036	0.0	3.249	0.0	11.0

Chad (7 regions) Dec/Jan 2012-13, regions	Missing/flagged		Sex ratio		Age ratio		Height DPS		Weight DPS		SD WHZ		Skew WHZ		Kurtosis WHZ		Total score
	%	score	p-value	score	value	score	value	score	value	score	SD	score	value	score	value	score	score
Logone Occidental	4.6	5.0	0.165	0.0	1.01	0.0	3.8	0.0	3.3	0.0	1.193	0.0	-0.425	5.0	3.356	1.0	11.0
Logone Oriental	6.5	10.0	0.809	0.0	1.01	0.0	6.0	2.0	2.3	0.0	1.169	0.0	-0.33	5.0	3.871	3.0	20.0
Mandoul	1.4	0.0	0.806	0.0	0.95	4.0	10.1	4.0	2.1	0.0	1.113	0.0	-0.518	5.0	4.317	5.0	18.0
Mayo-Kebbi Est	4.4	5.0	0.639	0.0	0.96	2.0	5.3	2.0	3.5	0.0	1.093	0.0	-0.242	3.0	3.95	5.0	17.0
Mayo-Kebbi Ouest	8.6	10.0	0.375	0.0	0.99	0.0	6.9	2.0	3.7	0.0	1.152	0.0	-0.399	5.0	4.644	5.0	22.0
Moyen-Chari	3.6	5.0	0.941	0.0	1.07	4.0	5.2	2.0	3.4	0.0	1.125	0.0	-0.261	3.0	4.693	5.0	19.0
Tandjilé	1.3	0.0	0.868	0.0	1.03	2.0	8.4	2.0	3.6	0.0	1.116	0.0	-0.396	5.0	4.247	5.0	14.0

The Gambia 2012, regions	Missing/flagged		Sex ratio		Age ratio		Height DPS		Weight DPS		SD WHZ		Skew WHZ		Kurtosis WHZ		Total score
	%	score	p-value	score	value	score	value	score	value	score	SD	score	value	score	value	score	score
banjul	4.3	5.0	0.855	0.0	0.97	2.0	6.7	2.0	3.5	0.0	1.09	0.0	0.13	1.0	3.989	5.0	15.0
basse	1.4	0.0	0.411	0.0	1.02	0.0	4.9	0.0	2.2	0.0	1.07	0.0	0.095	0.0	3.538	1.0	1.0
brikama	3.3	5.0	0.141	0.0	0.96	2.0	4.9	0.0	4.7	0.0	1.03	0.0	0.187	1.0	3.728	3.0	11.0
janjanburay	1.2	0.0	0.33	0.0	1.06	4.0	6.0	2.0	3.5	0.0	1.103	0.0	-0.12	1.0	4.339	5.0	12.0
kanifing	2.4	0.0	0.718	0.0	0.92	4.0	6.6	2.0	2.6	0.0	1.098	0.0	0.106	1.0	3.921	5.0	12.0
kerewan	1.3	0.0	0.369	0.0	0.96	2.0	5.1	2.0	2.1	0.0	1.075	0.0	0.036	0.0	4.149	5.0	9.0
kuntaur	3.4	5.0	0.857	0.0	1.05	4.0	8.9	2.0	2.8	0.0	1.045	0.0	-0.259	3.0	3.439	1.0	15.0
mansakonko	1.5	0.0	0.113	0.0	1	0.0	7.4	2.0	4.1	0.0	1.079	0.0	0.043	0.0	4.014	5.0	7.0

Guinea-Bissau 2008, regions	Missing/flagged		Sex ratio		Age ratio		Height DPS		Weight DPS		SD WHZ		Skew WHZ		Kurtosis WHZ		Total score
	%	score	p-value	score	value	score	value	score	value	score	SD	score	value	score	value	score	score
Capitale	1.3	0.0	0.236	0.0	.	5.0	8.0	2.0	3.7	0.0	1.077	0.0	0.118	1.0	4.668	5.0	13.0

	Missing/flagged %	score	Sex ratio p-value	score	Age ratio value	score	Height DPS value	score	Weight DPS value	score	SD WHZ SD	score	Skew WHZ value	score	Kurtosis WHZ value	score	Total score score
Est (Bafata e Gabu)	0.8	0.0	0.189	0.0	1.14	10.0	8.2	2.0	3.4	0.0	1.045	0.0	-0.108	1.0	3.588	1.0	14.0
Nord (Biombo, Cacheu e Oio)	2.8	5.0	0.56	0.0	1.33	10.0	7.5	2.0	4.3	0.0	1.085	0.0	-0.027	0.0	3.765	3.0	20.0
Sud (Bolama, Quinara e Tombali)	1.9	0.0	0.38	0.0	0.83	10.0	5.4	2.0	4.6	0.0	1.021	0.0	0.225	3.0	4.489	5.0	20.0

Guinée Conakay 2012, regions	Missing/flagged		Sex ratio		Age ratio		Height DPS		Weight DPS		SD WHZ		Skew WHZ		Kurtosis WHZ		Total score
	%	score	p-value	score	value	score	value	score	value	score	SD	score	value	score	value	score	score
boke nord	0.4	0.0	0.864	0.0	1.05	2.0	8.8	2.0	9.0	2.0	1.218	2.0	0.41	5.0	3.85	3.0	16.0
boke sud	1.0	0.0	0.523	0.0	1.04	2.0	4.4	0.0	4.3	0.0	1.111	0.0	0.115	1.0	4.018	5.0	8.0
conakry	1.3	0.0	0.569	0.0	.	5.0	10.4	4.0	10.3	4.0	1.169	0.0	0.132	1.0	4.67	5.0	19.0
faranah	0.7	0.0	0.65	0.0	0.97	0.0	5.4	2.0	5.5	2.0	1.175	0.0	0.007	0.0	4.675	5.0	9.0
kankan	1.1	0.0	0.121	0.0	1.24	10.0	5.7	2.0	5.3	2.0	1.108	0.0	-0.184	1.0	4.453	5.0	20.0
kindia	1.0	0.0	0.268	0.0	1.16	10.0	6.3	2.0	6.7	2.0	1.193	0.0	-0.117	1.0	4.294	5.0	20.0
labe	0.9	0.0	0.128	0.0	.	5.0	3.9	0.0	3.9	0.0	1.159	0.0	-0.197	1.0	4.18	5.0	11.0
mamou	1.3	0.0	0.812	0.0	1.26	10.0	8.2	2.0	8.3	2.0	1.089	0.0	0.029	0.0	3.863	3.0	17.0
nzerekore	0.9	0.0	0.904	0.0	1.02	0.0	7.6	2.0	7.0	2.0	1.079	0.0	-0.174	1.0	3.923	5.0	10.0

Liberia 2010, regions	Missing/flagged		Sex ratio		Age ratio		Height DPS		Weight DPS		SD WHZ		Skew WHZ		Kurtosis WHZ		Total score
	%	score	p-value	score	value	score	value	score	value	score	SD	score	value	score	value	score	score
bomi	3.0	5.0	0.827	0.0	0.87	10.0	10.1	4.0	4.2	0.0	1.091	0.0	0.011	0.0	3.65	3.0	22.0
bong	3.6	5.0	0.68	0.0	0.95	4.0	12.2	4.0	6.8	2.0	1.085	0.0	-0.3	3.0	3.385	1.0	19.0
gbarpolu	5.1	10.0	0.193	0.0	0.84	10.0	12.1	4.0	9.9	2.0	1.185	0.0	-0.457	5.0	4.325	5.0	36.0
grand bassa	2.0	0.0	0.456	0.0	0.87	10.0	7.6	2.0	5.6	2.0	1.096	0.0	-0.127	1.0	3.659	3.0	18.0
grand cape mount	1.4	0.0	1	0.0	0.73	10.0	11.9	4.0	7.8	2.0	1.222	2.0	-0.273	3.0	5.044	5.0	26.0
grand gedeh	1.6	0.0	0.318	0.0	0.92	4.0	6.5	2.0	3.8	0.0	1.13	0.0	-0.371	5.0	5.094	5.0	16.0
grand kru	2.7	5.0	0.572	0.0	1.13	10.0	7.2	2.0	6.3	2.0	1.025	0.0	-0.217	3.0	4.527	5.0	27.0
lofa	1.7	0.0	0.553	0.0	1	0.0	6.7	2.0	2.8	0.0	1.078	0.0	-0.117	1.0	4.888	5.0	8.0
margibi	1.0	0.0	0.028	4.0	0.8	10.0	15.5	4.0	14.4	4.0	1.117	0.0	0.045	0.0	3.76	3.0	25.0
maryland	4.3	5.0	0.959	0.0	0.97	2.0	10.8	4.0	6.9	2.0	1.26	2.0	-0.355	5.0	3.972	5.0	25.0
montserrado	1.1	0.0	0.392	0.0	0.82	10.0	12.9	4.0	5.0	0.0	1.055	0.0	0.155	1.0	5.089	5.0	20.0
nimba	0.9	0.0	0.078	2.0	0.98	0.0	10.5	4.0	5.3	2.0	1.016	0.0	-0.14	1.0	4.144	5.0	14.0
river gee	5.7	10.0	0.144	0.0	1.04	2.0	8.5	2.0	7.4	2.0	1.089	0.0	0.226	3.0	3.641	3.0	22.0
rivercess	1.7	0.0	0.712	0.0	0.96	2.0	5.5	2.0	3.3	0.0	1.116	0.0	-0.363	5.0	4.517	5.0	14.0
rural montserrado	2.6	5.0	0.306	0.0	0.69	10.0	9.7	2.0	7.9	2.0	1.069	0.0	-0.379	5.0	4.275	5.0	29.0
sinoe	2.3	0.0	0.257	0.0	1.13	10.0	16.2	4.0	5.1	2.0	1.184	0.0	-0.33	5.0	3.732	3.0	24.0

Liberia 2011, regions	Missing/flagged		Sex ratio		Age ratio		Height DPS		Weight DPS		SD WHZ		Skew WHZ		Kurtosis WHZ		Total score
	%	score	p-value	score	value	score	value	score	value	score	SD	score	value	score	value	score	score
North Central	2.2	0.0	0.67	0.0	.	5.0	12.3	4.0	5.6	2.0	1.087	0.0	-0.288	3.0	3.639	3.0	17.0
North Western	3.2	5.0	0.756	0.0	.	5.0	20.2	10.0	10.9	4.0	1.191	0.0	-1.124	5.0	4.88	5.0	34.0
South Central	0.6	0.0	0.705	0.0	.	5.0	10.1	4.0	3.1	0.0	1.272	2.0	0.083	0.0	3.421	1.0	12.0
South Eastern	3.3	5.0	0.273	0.0	.	5.0	12.1	4.0	5.1	2.0	1.191	0.0	-0.391	5.0	2.998	0.0	21.0

Mali 2011, regions	Missing/flagged		Sex ratio		Age ratio		Height DPS		Weight DPS		SD WHZ		Skew WHZ		Kurtosis WHZ		Total score
	%	score	p-value	score	value	score	value	score	value	score	SD	score	value	score	value	score	score
bamako	5.8	10.0	0.519	0.0	1.09	4.0	8.4	2.0	4.4	0.0	1.068	0.0	0.302	5.0	3.862	3.0	24.0
gao	0.4	0.0	0.03	4.0	1.06	4.0	5.3	2.0	2.9	0.0	1.131	0.0	0.009	0.0	3.481	1.0	11.0
kayes	5.7	10.0	0.626	0.0	1.2	10.0	3.7	0.0	4.5	0.0	1.126	0.0	-0.067	0.0	3.122	0.0	20.0
kidal	0.9	0.0	0.948	0.0	1.22	10.0	4.7	0.0	5.5	2.0	1.044	0.0	-0.001	0.0	2.608	1.0	13.0
koulikoro	1.0	0.0	0.003	4.0	1.13	10.0	4.2	0.0	3.6	0.0	1.111	0.0	-0.187	1.0	3.374	1.0	16.0
mopti	1.8	0.0	0.21	0.0	1.15	10.0	10.2	4.0	3.5	0.0	1.112	0.0	-0.138	1.0	3.303	1.0	16.0
segou	1.6	0.0	0.847	0.0	0.98	0.0	3.5	0.0	3.1	0.0	1.027	0.0	-0.347	5.0	3.436	1.0	6.0
sikasso	4.5	5.0	0.568	0.0	1.08	4.0	5.6	2.0	4.6	0.0	1.071	0.0	-0.193	1.0	3.889	3.0	15.0
tombouctou	0.1	0.0	0.49	0.0	0.94	4.0	8.6	2.0	4.1	0.0	1.071	0.0	0.096	0.0	3.771	3.0	9.0

Mauritania 2006, regions	Missing/flagged		Sex ratio		Age ratio		Height DPS		Weight DPS		SD WHZ		Skew WHZ		Kurtosis WHZ		Total score
	%	score	p-value	score	value	score	value	score	value	score	SD	score	value	score	value	score	score

Centre	4.3	5.0	0.303	0.0	1.08	4.0	18.9	4.0	8.3	2.0	1.114	0.0	-0.316	5.0	4.113	5.0	25.0
Fleuve	6.5	10.0	0.757	0.0	1.11	10.0	17.4	4.0	4.2	0.0	1.171	0.0	-0.167	1.0	4.016	5.0	30.0
Nord	3.5	5.0	0.51	0.0	1.02	0.0	14.2	4.0	6.2	2.0	1.124	0.0	-0.242	3.0	3.907	5.0	19.0
Nouakchott	5.9	10.0	0.341	0.0	1.08	4.0	19.6	4.0	3.2	0.0	1.186	0.0	-0.032	0.0	4.214	5.0	23.0
SudEst	5.3	10.0	1	0.0	0.97	0.0	17.2	4.0	5.0	0.0	1.152	0.0	-0.253	3.0	4.25	5.0	22.0

Mauritania March 2008, regions	Missing/flagged		Sex ratio		Age ratio		Height DPS		Weight DPS		SD WHZ		Skew WHZ		Kurtosis WHZ		Total score
	%	score	p-value	score	value	score	value	score	value	score	SD	score	value	score	value	score	score
Centre	1.2	0.0	0.075	2.0	0.83	10.0	39.6	10.0	4.6	0.0	1.151	0.0	0.104	1.0	4.044	5.0	28.0
Fleuve Nord	1.1	0.0	0.012	4.0	1.09	4.0	14.4	4.0	2.9	0.0	1.22	2.0	0.055	0.0	3.754	3.0	17.0
Fleuve Sud	0.9	0.0	0.95	0.0	1.22	10.0	26.6	10.0	3.5	0.0	1.195	0.0	0.048	0.0	3.407	1.0	21.0
Nord	0.4	0.0	0.753	0.0	0.85	10.0	14.6	4.0	7.3	2.0	1.293	2.0	-0.387	5.0	3.962	5.0	28.0
Nouakchott	1.5	0.0	0	10.0	0.96	2.0	8.7	2.0	4.9	0.0	1.286	2.0	0.189	1.0	4.04	5.0	22.0
SudEst	1.6	0.0	0.016	4.0	1.14	10.0	29.8	10.0	4.5	0.0	1.244	2.0	-0.049	0.0	3.509	1.0	27.0

Mauritania Dec 2008, regions	Missing/flagged		Sex ratio		Age ratio		Height DPS		Weight DPS		SD WHZ		Skew WHZ		Kurtosis WHZ		Total score
	%	score	p-value	score	value	score	value	score	value	score	SD	score	value	score	value	score	score
Centre	0.3	0.0	0.498	0.0	0.97	2.0	9.2	2.0	1.7	0.0	1.012	0.0	-0.054	0.0	3.517	1.0	5.0
Nord	0.1	0.0	0.293	0.0	0.88	10.0	7.8	2.0	5.6	2.0	0.962	0.0	-0.166	1.0	3.393	1.0	16.0
Nouakchott A	0.4	0.0	1	0.0	1.07	4.0	3.9	0.0	4.1	0.0	1.069	0.0	-0.264	3.0	3.19	0.0	7.0
Nouakchott B	0.1	0.0	0.718	0.0	1.1	10.0	5.3	2.0	3.2	0.0	0.989	0.0	-0.12	1.0	3.068	0.0	13.0
Sud	0.6	0.0	0	10.0	.	5.0	5.4	2.0	2.1	0.0	1.074	0.0	0.284	3.0	3.768	3.0	23.0
SudEst	0.3	0.0	0.014	4.0	1.12	10.0	4.4	0.0	4.0	0.0	1.05	0.0	-0.11	1.0	3.428	1.0	16.0
Trarza	0.0	0.0	0.661	0.0	1.04	2.0	5.0	2.0	4.6	0.0	1.008	0.0	-0.092	0.0	2.827	0.0	4.0

Mauritania 2009, regions	Missing/flagged		Sex ratio		Age ratio		Height DPS		Weight DPS		SD WHZ		Skew WHZ		Kurtosis WHZ		Total score
	%	score	p-value	score	value	score	value	score	value	score	SD	score	value	score	value	score	score
Centre	0.0	0.0	0.384	0.0	1.01	0.0	7.2	2.0	4.4	0.0	1.102	0.0	0.094	0.0	3.017	0.0	2.0
Nord	0.1	0.0	0.018	4.0	1.07	4.0	5.7	2.0	7.7	2.0	1.024	0.0	-0.146	1.0	3.511	1.0	14.0
Nouakchott	0.1	0.0	0.565	0.0	1.31	10.0	4.1	0.0	4.6	0.0	1.089	0.0	-0.168	1.0	2.984	0.0	11.0
Sud	0.0	0.0	0.772	0.0	1.14	10.0	6.2	2.0	2.9	0.0	1.07	0.0	-0.152	1.0	3.687	3.0	16.0
Sud-est	0.0	0.0	0.089	2.0	1.27	10.0	8.1	2.0	5.3	2.0	1.105	0.0	0.142	1.0	3.405	1.0	18.0
Trarza	0.0	0.0	0.68	0.0	1.15	10.0	5.5	2.0	4.7	0.0	1.108	0.0	-0.252	3.0	3.442	1.0	16.0

Mauritania July 2010, regions	Missing/flagged		Sex ratio		Age ratio		Height DPS		Weight DPS		SD WHZ		Skew WHZ		Kurtosis WHZ		Total score
	%	score	p-value	score	value	score	value	score	value	score	SD	score	value	score	value	score	score
adrar/inchiri tiris	0.0	0.0	0.812	0.0	0.91	4.0	10.1	4.0	3.6	0.0	0.964	0.0	-0.02	0.0	4.087	5.0	13.0
assaba	0.0	0.0	0.689	0.0	0.91	4.0	6.7	2.0	6.1	2.0	0.876	0.0	-0.353	5.0	2.988	0.0	13.0
brakna	0.2	0.0	0.934	0.0	1.08	4.0	10.9	4.0	4.1	0.0	1.031	0.0	0.08	0.0	3.17	0.0	8.0
gorgol	0.0	0.0	0.435	0.0	0.84	10.0	6.2	2.0	3.3	0.0	0.953	0.0	0.059	0.0	3.117	0.0	12.0
guidimakha	0.3	0.0	0.937	0.0	1.98	10.0	14.0	4.0	2.7	0.0	1.058	0.0	-0.093	0.0	3.449	1.0	15.0
hodh chargui	0.4	0.0	0.22	0.0	1.12	10.0	11.0	4.0	6.6	2.0	0.996	0.0	0.105	1.0	3.783	3.0	20.0
hodh gharbi	0.0	0.0	0.362	0.0	1.27	10.0	7.9	2.0	3.9	0.0	0.93	0.0	0.063	0.0	3.051	0.0	12.0
nouadhibou	0.0	0.0	0.014	4.0	1.2	10.0	6.2	2.0	4.6	0.0	1.05	0.0	-0.003	0.0	3.73	3.0	19.0
nouakchott	0.8	0.0	0.056	2.0	1.14	10.0	6.3	2.0	4.8	0.0	1.244	2.0	0.041	0.0	3.613	3.0	19.0
tagant	0.6	0.0	0.479	0.0	0.93	4.0	5.7	2.0	2.4	0.0	0.972	0.0	0.53	5.0	4.493	5.0	16.0
trarza	0.0	0.0	0.804	0.0	0.84	10.0	8.8	2.0	3.6	0.0	1.022	0.0	0.094	0.0	2.974	0.0	12.0

Mauritania Dec 2010, regions	Missing/flagged		Sex ratio		Age ratio		Height DPS		Weight DPS		SD WHZ		Skew WHZ		Kurtosis WHZ		Total score
	%	score	p-value	score	value	score	value	score	value	score	SD	score	value	score	value	score	score
adrar/inchiri tiris	1.2	0.0	0.276	0.0	1.09	4.0	6.0	2.0	3.8	0.0	1.066	0.0	0.031	0.0	2.714	0.0	6.0
assaba	0.0	0.0	0.611	0.0	0.87	10.0	12.0	4.0	4.4	0.0	0.999	0.0	-0.056	0.0	3.642	3.0	17.0
brakna	0.0	0.0	0.529	0.0	1.39	10.0	7.1	2.0	4.1	0.0	1.039	0.0	0.159	1.0	3.229	0.0	13.0
gorgol	0.2	0.0	0.58	0.0	1.03	0.0	6.9	2.0	4.3	0.0	0.963	0.0	0.136	1.0	3.31	1.0	4.0
guidimakha	0.5	0.0	0.253	0.0	1.42	10.0	3.4	0.0	3.6	0.0	0.966	0.0	-0.17	1.0	4.539	5.0	16.0
hodh chargui	0.0	0.0	0.009	4.0	1.13	10.0	8.8	2.0	4.2	0.0	1.049	0.0	0.001	0.0	3.089	0.0	16.0

hodh gharbi	0.2	0.0	0.217	0.0	0.9	4.0	17.2	4.0	4.3	0.0	0.993	0.0	0.042	0.0	3.43	1.0	9.0
nouadhibou	0.4	0.0	0.292	0.0	0.89	10.0	4.2	0.0	3.9	0.0	0.945	0.0	0.17	1.0	3.205	0.0	11.0
nouakchott	0.6	0.0	0.455	0.0	1.29	10.0	5.5	2.0	6.3	2.0	1.071	0.0	-0.154	1.0	3.85	3.0	18.0
tagant	0.2	0.0	0.076	2.0	0.8	10.0	11.1	4.0	4.3	0.0	1.055	0.0	0.107	1.0	3.291	0.0	17.0
trarza	0.8	0.0	0.714	0.0	1.02	0.0	5.9	2.0	3.3	0.0	0.976	0.0	0.117	1.0	3.463	1.0	4.0

Mauritania July 2011, regions	Missing/flagged		Sex ratio		Age ratio		Height DPS		Weight DPS		SD WHZ		Skew WHZ		Kurtosis WHZ		Total score
	%	score	p-value	score	value	score	value	score	value	score	SD	score	value	score	value	score	score
adrar/inchiri tiris	1.5	0.0	0.969	0.0	0.99	0.0	22.8	10.0	5.0	2.0	1.048	0.0	-0.209	3.0	3.429	1.0	16.0
assaba	0.0	0.0	0.292	0.0	0.87	10.0	13.6	4.0	3.7	0.0	0.995	0.0	0.005	0.0	2.953	0.0	14.0
brakna	0.7	0.0	0.226	0.0	0.79	10.0	7.2	2.0	3.1	0.0	1.034	0.0	0.072	0.0	3.088	0.0	12.0
gorgol	0.0	0.0	0.701	0.0	1.34	10.0	5.4	2.0	3.4	0.0	0.917	0.0	0.108	1.0	2.757	0.0	13.0
guidimakha	1.4	0.0	0.639	0.0	1.45	10.0	17.6	4.0	5.8	2.0	1.003	0.0	-0.025	0.0	3.535	1.0	17.0
hodh chargui	12.4	20.0	0.783	0.0	.	5.0	15.5	4.0	4.6	0.0	0.987	0.0	0.052	0.0	3.215	0.0	29.0
hodh gharbi	1.0	0.0	0.409	0.0	1.02	0.0	6.1	2.0	5.8	2.0	1.004	0.0	0.303	5.0	4.489	5.0	14.0
nouadhibou	0.0	0.0	0.538	0.0	0.8	10.0	6.3	2.0	7.9	2.0	1.077	0.0	-0.136	1.0	3.433	1.0	16.0
nouakchott	0.3	0.0	0.503	0.0	1.19	10.0	5.7	2.0	3.4	0.0	0.939	0.0	0.035	0.0	2.878	0.0	12.0
tagant	0.5	0.0	0.557	0.0	.	5.0	3.5	0.0	5.4	2.0	0.978	0.0	0.261	3.0	3.879	3.0	13.0
trarza	2.1	0.0	0.479	0.0	0.86	10.0	5.3	2.0	4.3	0.0	1.084	0.0	0.316	5.0	4.254	5.0	22.0

Mauritania Dec 2011, regions	Missing/flagged		Sex ratio		Age ratio		Height DPS		Weight DPS		SD WHZ		Skew WHZ		Kurtosis WHZ		Total score
	%	score	p-value	score	value	score	value	score	value	score	SD	score	value	score	value	score	score
adrar/inchiri tiris	0.3	0.0	0.253	0.0	1.04	2.0	8.2	2.0	2.9	0.0	0.954	0.0	-0.244	3.0	3.863	3.0	10.0
assaba	0.0	0.0	0.076	2.0	0.88	10.0	5.4	2.0	2.7	0.0	0.956	0.0	0.157	1.0	2.986	0.0	15.0
brakna	0.6	0.0	0.014	4.0	1.27	10.0	7.2	2.0	2.0	0.0	1.022	0.0	-0.085	0.0	3.128	0.0	16.0
gorgol	0.5	0.0	0.11	0.0	.	5.0	14.1	4.0	4.9	0.0	1.096	0.0	-0.173	1.0	3.792	3.0	13.0
guidimakha	0.5	0.0	0.356	0.0	1.42	10.0	11.4	4.0	2.8	0.0	0.995	0.0	0.091	0.0	3.95	5.0	19.0
hodh chargui	0.2	0.0	0.523	0.0	1.36	10.0	7.5	2.0	3.9	0.0	1.031	0.0	-0.182	1.0	3.2	0.0	13.0
hodh gharbi	0.4	0.0	0.217	0.0	1.02	0.0	2.8	0.0	4.2	0.0	0.902	0.0	-0.082	0.0	3.172	0.0	0.0
nouadhibou	0.3	0.0	0.765	0.0	0.89	10.0	1.2	0.0	3.1	0.0	1.059	0.0	-0.129	1.0	3.37	1.0	12.0
nouakchott	0.6	0.0	0.189	0.0	1.24	10.0	8.3	2.0	5.4	2.0	1.021	0.0	0.125	1.0	3.666	3.0	18.0
tagant	0.0	0.0	0.002	4.0	1.08	4.0	6.2	2.0	3.2	0.0	1.014	0.0	-0.075	0.0	3.58	1.0	11.0
trarza	0.6	0.0	0.235	0.0	0.76	10.0	15.7	4.0	5.2	2.0	0.986	0.0	-0.041	0.0	4.178	5.0	21.0

Mauritania July 2012, regions	Missing/flagged		Sex ratio		Age ratio		Height DPS		Weight DPS		SD WHZ		Skew WHZ		Kurtosis WHZ		Total score
	%	score	p-value	score	value	score	value	score	value	score	SD	score	value	score	value	score	score
adrar/inchiri tiris	0.4	0.0	0.027	4.0	0.84	10.0	7.8	2.0	4.9	0.0	1.067	0.0	-0.037	0.0	2.97	0.0	16.0
assaba	0.2	0.0	0.114	0.0	1.1	4.0	11.9	4.0	3.6	0.0	1.09	0.0	0.082	0.0	2.923	0.0	8.0
brakna	0.0	0.0	0.003	4.0	1.34	10.0	10.5	4.0	4.7	0.0	1.156	0.0	0.011	0.0	3.367	1.0	19.0
gorgol	0.0	0.0	0.571	0.0	1.5	10.0	9.1	2.0	4.7	0.0	1.085	0.0	0.186	1.0	3.023	0.0	13.0
guidimakha	0.4	0.0	0.02	4.0	0.97	2.0	10.2	4.0	4.1	0.0	0.968	0.0	-0.249	3.0	3.43	1.0	14.0
hodh chargui	0.3	0.0	1	0.0	.	5.0	7.3	2.0	4.4	0.0	1.038	0.0	-0.027	0.0	3.235	0.0	7.0
hodh gharbi	0.0	0.0	0.008	4.0	1.04	2.0	7.9	2.0	3.5	0.0	1.024	0.0	0.222	3.0	3.178	0.0	11.0
nouadhibou	0.0	0.0	0.854	0.0	.	5.0	10.0	2.0	5.7	2.0	1.085	0.0	-0.138	1.0	3.321	1.0	11.0
nouakchott	1.2	0.0	0.41	0.0	0.85	10.0	8.5	2.0	3.1	0.0	1.116	0.0	0.114	1.0	3.812	3.0	16.0
tagant	0.0	0.0	0.087	2.0	0.95	2.0	5.0	2.0	2.8	0.0	1.007	0.0	0.025	0.0	3.013	0.0	6.0
trarza	0.4	0.0	0.64	0.0	0.8	10.0	4.5	0.0	4.4	0.0	1.113	0.0	0.089	0.0	3.432	1.0	11.0

Niger 2012, regions	Missing/flagged		Sex ratio		Age ratio		Height DPS		Weight DPS		SD WHZ		Skew WHZ		Kurtosis WHZ		Total score
	%	score	p-value	score	value	score	value	score	value	score	SD	score	value	score	value	score	score
agadez	0.3	0.0	0.034	4.0	0.99	0.0	7.4	2.0	1.8	0.0	0.994	0.0	-0.044	0.0	3.009	0.0	6.0
diffa	1.9	0.0	0.847	0.0	0.93	4.0	6.6	2.0	2.5	0.0	1.077	0.0	0.024	0.0	3.553	1.0	7.0
dosso	1.6	0.0	0.128	0.0	0.95	2.0	5.0	0.0	3.4	0.0	1.059	0.0	-0.135	1.0	3.426	1.0	4.0
maradi	0.6	0.0	0.074	2.0	0.95	2.0	5.8	2.0	3.1	0.0	1.134	0.0	0.001	0.0	3.209	0.0	6.0
niamey	1.5	0.0	0.43	0.0	0.93	4.0	3.0	0.0	3.2	0.0	1.064	0.0	0.574	5.0	5.667	5.0	14.0
tahoua	1.1	0.0	0.124	0.0	0.92	4.0	4.1	0.0	4.8	0.0	1.207	2.0	0.204	3.0	4.19	5.0	14.0

tillabéri	0.7	0.0	0.57	0.0	1.07	4.0	5.7	2.0	5.1	2.0	1.1	0.0	-0.014	0.0	3.366	1.0	9.0
zinder	1.8	0.0	0.203	0.0	1.03	2.0	4.1	0.0	2.4	0.0	1.113	0.0	-0.271	3.0	3.426	1.0	6.0

Nigeria (Northern States) 2011, regions	Missing/flagged		Sex ratio		Age ratio		Height DPS		Weight DPS		SD WHZ		Skew WHZ		Kurtosis WHZ		Total score
	%	score	p-value	score	value	score	value	score	value	score	SD	score	value	score	value	score	score
jigawa	8.6	10.0	0.195	0.0	0.96	2.0	6.2	2.0	4.5	0.0	1.251	2.0	0.057	0.0	3.897	3.0	19.0
kano	15.5	20.0	0.276	0.0	0.84	10.0	6.0	2.0	4.0	0.0	1.194	0.0	0.168	1.0	4.358	5.0	38.0
katsina	8.5	10.0	0.021	4.0	1.09	4.0	5.0	0.0	3.4	0.0	1.218	2.0	-0.188	1.0	3.633	3.0	24.0
kebbi	3.9	5.0	0.159	0.0	0.88	10.0	5.7	2.0	2.5	0.0	1.176	0.0	-0.408	5.0	3.483	1.0	23.0
sokoto	7.8	10.0	0.58	0.0	0.9	4.0	5.5	2.0	2.8	0.0	1.151	0.0	-0.311	5.0	3.704	3.0	24.0
yobe	6.1	10.0	0.951	0.0	1	0.0	5.4	2.0	2.4	0.0	1.094	0.0	0.143	1.0	3.55	1.0	14.0
zamfara	6.8	10.0	0.506	0.0	0.87	10.0	4.8	0.0	4.4	0.0	1.226	2.0	-0.559	5.0	3.734	3.0	30.0

Senegal 2012, regions	Missing/flagged		Sex ratio		Age ratio		Height DPS		Weight DPS		SD WHZ		Skew WHZ		Kurtosis WHZ		Total score
	%	score	p-value	score	value	score	value	score	value	score	SD	score	value	score	value	score	score
dakar	19.7	20.0	0.061	2.0	0.94	4.0	7.2	2.0	4.6	0.0	1.076	0.0	0.153	1.0	3.378	1.0	30.0
kolda	0.4	0.0	0.966	0.0	0.84	10.0	10.7	4.0	3.3	0.0	1.021	0.0	-0.296	3.0	3.29	0.0	17.0
matam	0.1	0.0	0.063	2.0	0.9	4.0	5.0	2.0	2.8	0.0	0.981	0.0	-0.03	0.0	3.387	1.0	9.0
myf	1.1	0.0	0.628	0.0	1.15	10.0	4.8	0.0	1.4	0.0	1.031	0.0	0.066	0.0	3.048	0.0	10.0
sedhiou	7.0	10.0	0.659	0.0	0.95	4.0	3.8	0.0	1.9	0.0	0.978	0.0	-0.05	0.0	3.425	1.0	15.0
tambacounda	5.2	10.0	0.672	0.0	1.02	0.0	4.0	0.0	2.0	0.0	1.031	0.0	-0.199	1.0	3.254	0.0	11.0
velingara	10.4	20.0	0.082	2.0	0.99	0.0	8.3	2.0	3.7	0.0	1.039	0.0	0.055	0.0	3.413	1.0	25.0

Sierra Leone 2010, regions	Missing/flagged		Sex ratio		Age ratio		Height DPS		Weight DPS		SD WHZ		Skew WHZ		Kurtosis WHZ		Total score
	%	score	p-value	score	value	score	value	score	value	score	SD	score	value	score	value	score	score
eastern	1.1	0.0	0.204	0.0	1.16	10.0	5.1	2.0	1.1	0.0	1.174	0.0	-0.308	5.0	3.655	3.0	20.0
northern	0.8	0.0	0.683	0.0	1.02	0.0	0.7	0.0	0.4	0.0	1.051	0.0	-0.189	1.0	3.168	0.0	1.0
southern	0.9	0.0	0.173	0.0	1.01	0.0	2.0	0.0	1.1	0.0	1.107	0.0	-0.171	1.0	3.144	0.0	1.0
western	1.6	0.0	0.572	0.0	1.32	10.0	5.3	2.0	2.6	0.0	1.208	2.0	-0.194	1.0	4.079	5.0	20.0

Togo June 2012, regions	Missing/flagged		Sex ratio		Age ratio		Height DPS		Weight DPS		SD WHZ		Skew WHZ		Kurtosis WHZ		Total score
	%	score	p-value	score	value	score	value	score	value	score	SD	score	value	score	value	score	score
Centrale	3.4	5.0	0.769	0.0	0.98	0.0	5.1	2.0	3.7	0.0	1.104	0.0	-0.035	0.0	3.631	3.0	10.0
Kara	4.6	5.0	0.685	0.0	1.08	4.0	2.8	0.0	5.7	2.0	1.066	0.0	0.006	0.0	3.973	5.0	16.0
Lomé	5.7	10.0	0.44	0.0	1.08	4.0	6.1	2.0	5.6	2.0	1.006	0.0	-0.115	1.0	3.447	1.0	20.0
Maritime	5.2	10.0	0.783	0.0	0.87	10.0	5.6	2.0	3.4	0.0	1.037	0.0	0.309	5.0	4.488	5.0	32.0
Plateaux	3.8	5.0	0.64	0.0	.	5.0	6.8	2.0	3.0	0.0	1.02	0.0	0.01	0.0	3.364	1.0	13.0
Savanes	4.4	5.0	0.274	0.0	0.96	2.0	3.4	0.0	3.2	0.0	1.033	0.0	0.058	0.0	3.673	3.0	10.0

Togo Dec 2012, regions	Missing/flagged		Sex ratio		Age ratio		Height DPS		Weight DPS		SD WHZ		Skew WHZ		Kurtosis WHZ		Total score
	%	score	p-value	score	value	score	value	score	value	score	SD	score	value	score	value	score	score
Kara	6.0	10.0	0.906	0.0	1.14	10.0	5.1	2.0	3.0	0.0	1.039	0.0	-0.325	5.0	3.807	3.0	30.0
Savanes	1.9	0.0	0.437	0.0	0.97	0.0	4.7	0.0	2.9	0.0	1.005	0.0	-0.075	0.0	3.886	3.0	3.0

Summary of anthropometric data quality indicators and total data quality score, MICS

Burkina Faso 2006, regions	Missing/flagged		Sex ratio		Age ratio		Height DPS		Weight DPS		SD WHZ		Skew WHZ		Kurtosis WHZ		Total score
	%	score	p-value	score	value	score	value	score	value	score	SD	score	value	score	value	score	score
Boucle du Mouhoun	10.6	20.0	0.659	0.0	0.87	10.0	16.6	4.0	41.7	10.0	1.824	15.0	0.023	0.0	2.586	1.0	60.0
Cascade	21.5	20.0	0.358	0.0	0.94	4.0	17.1	4.0	66.0	10.0	1.925	15.0	0.028	0.0	2.549	1.0	54.0
Centre	17.3	20.0	0.826	0.0	0.85	10.0	23.6	10.0	71.5	10.0	1.781	15.0	-0.025	0.0	2.992	0.0	65.0
Centre-Est	15.9	20.0	0.956	0.0	.	5.0	48.0	10.0	58.2	10.0	2.01	15.0	-0.171	1.0	2.857	0.0	61.0
Centre-Nord	22.2	20.0	0.386	0.0	1.07	4.0	39.4	10.0	81.8	10.0	1.877	15.0	-0.225	3.0	2.618	1.0	63.0
Centre-Ouest	14.3	20.0	0.241	0.0	0.84	10.0	39.5	10.0	34.1	10.0	1.504	6.0	0.053	0.0	3.015	0.0	56.0
Centre-Sud	9.6	10.0	0.834	0.0	0.91	4.0	42.3	10.0	64.9	10.0	1.694	15.0	-0.088	0.0	2.841	0.0	49.0
Est	26.6	20.0	0.715	0.0	1.18	10.0	37.9	10.0	65.4	10.0	1.986	15.0	0.173	1.0	2.712	0.0	66.0
Hauts-Bassins	12.2	20.0	0.445	0.0	1.1	4.0	20.5	10.0	42.8	10.0	1.792	15.0	0.175	1.0	2.947	0.0	60.0
Nord	18.2	20.0	0.856	0.0	0.95	2.0	47.3	10.0	70.9	10.0	2.016	15.0	0.08	0.0	2.612	1.0	58.0
Plateau-Central	16.2	20.0	0.039	4.0	0.9	10.0	35.1	10.0	68.6	10.0	1.894	15.0	0.023	0.0	2.748	0.0	69.0
Sahel	23.1	20.0	0.356	0.0	1.01	0.0	54.3	10.0	88.7	10.0	2.125	15.0	0.221	3.0	2.723	0.0	58.0
Sud-Ouest	16.8	20.0	0.574	0.0	1.23	10.0	29.3	10.0	58.6	10.0	1.844	15.0	-0.16	1.0	2.512	1.0	67.0

Cameroon 2006, regions	Missing/flagged		Sex ratio		Age ratio		Height DPS		Weight DPS		SD WHZ		Skew WHZ		Kurtosis WHZ		Total score
	%	score	p-value	score	value	score	value	score	value	score	SD	score	value	score	value	score	score
Adamaoua	5.2	10.0	0.326	0.0	0.97	0.0	9.9	2.0	4.3	0.0	1.264	2.0	-0.252	3.0	3.974	5.0	22.0
Centre	10.0	20.0	0.176	0.0	0.85	10.0	37.4	10.0	3.9	0.0	1.367	2.0	0.077	0.0	3.816	3.0	45.0
Douala	12.2	20.0	0.799	0.0	0.88	10.0	33.8	10.0	9.9	2.0	1.508	6.0	-0.336	5.0	3.809	3.0	56.0
Est	6.5	10.0	0.09	2.0	0.88	10.0	16.5	4.0	3.6	0.0	1.353	2.0	-0.435	5.0	3.798	3.0	36.0
Extreme Nord	6.4	10.0	0.971	0.0	1.03	0.0	12.4	4.0	3.8	0.0	1.276	2.0	-0.104	1.0	4.033	5.0	22.0
Littoral	3.8	5.0	0.286	0.0	0.97	2.0	12.6	4.0	4.4	0.0	1.389	2.0	-0.288	3.0	4.409	5.0	21.0
Nord	15.0	20.0	0.599	0.0	1	0.0	26.4	10.0	11.7	4.0	1.842	15.0	0.274	3.0	3.018	0.0	52.0
Nord Ouest	16.7	20.0	0.646	0.0	0.83	10.0	13.2	4.0	6.9	2.0	1.569	6.0	-0.497	5.0	3.597	1.0	48.0
Ouest	6.6	10.0	0.248	0.0	0.93	4.0	13.3	4.0	6.3	2.0	1.197	0.0	0.107	1.0	4.082	5.0	26.0
Sud	14.0	20.0	0.455	0.0	0.83	10.0	29.5	10.0	7.4	2.0	1.414	6.0	-0.079	0.0	3.66	3.0	51.0
Sud Ouest	6.0	10.0	0.759	0.0	0.92	4.0	25.0	10.0	3.6	0.0	1.341	2.0	-0.015	0.0	3.368	1.0	27.0
Yaounde	17.9	20.0	0.678	0.0	0.94	4.0	38.0	10.0	6.1	2.0	1.367	2.0	-0.238	3.0	4.154	5.0	46.0

Central African Rep 2000, regions	Missing/flagged		Sex ratio		Age ratio		Height DPS		Weight DPS		SD WHZ		Skew WHZ		Kurtosis WHZ		Total score
	%	score	p-value	score	value	score	value	score	value	score	SD	score	value	score	value	score	score
Bamingui-Bangoran	8.0	10.0	0.487	0.0	1.02	0.0	71.4	10.0	15.9	4.0	1.512	6.0	0.109	1.0	3.315	1.0	32.0
Bangui	12.4	20.0	0.112	0.0	0.95	2.0	83.8	10.0	22.4	10.0	1.727	15.0	-0.109	1.0	3.363	1.0	59.0
Basse-Kotto	16.6	20.0	0.086	2.0	0.99	0.0	82.8	10.0	23.4	10.0	1.96	15.0	-0.047	0.0	2.869	0.0	57.0
Haut-Mbomou	13.2	20.0	0.918	0.0	0.9	10.0	65.8	10.0	23.8	10.0	1.62	15.0	-0.131	1.0	3.487	1.0	67.0
Haute-Kotto	7.7	10.0	0.477	0.0	0.92	4.0	86.9	10.0	24.7	10.0	1.449	6.0	-0.189	1.0	4.303	5.0	46.0
Kémo	12.0	20.0	0.596	0.0	1.02	0.0	92.2	10.0	16.8	4.0	1.661	15.0	-0.241	3.0	3.646	3.0	55.0
Lobaye	13.4	20.0	0.068	2.0	1.04	2.0	82.1	10.0	16.9	4.0	1.718	15.0	-0.168	1.0	3.038	0.0	54.0
Mambéré-Kadeï	9.0	10.0	0.856	0.0	0.9	4.0	63.8	10.0	17.8	4.0	1.37	2.0	0.015	0.0	3.789	3.0	33.0
Mbomou	20.7	20.0	0.571	0.0	1	0.0	91.5	10.0	29.5	10.0	1.766	15.0	-0.152	1.0	3.155	0.0	56.0
Nana-Grébizi	5.8	10.0	0.731	0.0	1.07	4.0	85.4	10.0	19.5	4.0	1.338	2.0	-0.379	5.0	3.802	3.0	38.0
Nana-Mambéré	10.3	20.0	0.863	0.0	.	5.0	45.0	10.0	27.3	10.0	1.759	15.0	-0.096	0.0	3.16	0.0	60.0
Ombella-M'poko	10.5	20.0	0.506	0.0	0.85	10.0	87.9	10.0	21.8	10.0	1.618	15.0	-0.165	1.0	3.693	3.0	69.0
Ouaka	11.1	20.0	0.239	0.0	0.93	4.0	78.2	10.0	29.6	10.0	1.806	15.0	-0.221	3.0	2.968	0.0	62.0
Ouham	8.3	10.0	0.936	0.0	1.06	4.0	73.6	10.0	18.1	4.0	1.546	6.0	-0.01	0.0	3.621	3.0	37.0
Ouham-Pendé	14.7	20.0	0.734	0.0	1.02	0.0	70.9	10.0	26.4	10.0	1.691	15.0	-0.468	5.0	3.431	1.0	61.0
Sangha-Mbaéré	7.2	10.0	0.258	0.0	0.94	4.0	88.4	10.0	19.6	4.0	1.444	6.0	0.005	0.0	4.185	5.0	39.0
Vakaga	10.9	20.0	0.183	0.0	1.22	10.0	78.4	10.0	17.1	4.0	1.614	15.0	0.116	1.0	3.659	3.0	63.0

Central African Rep 2006, regions	Missing/flagged		Sex ratio		Age ratio		Height DPS		Weight DPS		SD WHZ		Skew WHZ		Kurtosis WHZ		Total score
	%	score	p-value	score	value	score	value	score	value	score	SD	score	value	score	value	score	score
"Baminigui Bangoran"	16.5	20.0	0.261	0.0	0.78	10.0	42.4	10.0	7.7	2.0	1.798	15.0	-0.004	0.0	3.088	0.0	57.0
"Bangui"	22.8	20.0	0.275	0.0	0.96	2.0	55.6	10.0	4.3	0.0	1.617	15.0	0.059	0.0	3.762	3.0	50.0
"Basse Kotto"	15.1	20.0	0.037	4.0	0.94	4.0	34.4	10.0	3.9	0.0	1.78	15.0	-0.148	1.0	3.332	1.0	55.0
"Haut Mbomou"	21.2	20.0	0.093	2.0	.	5.0	29.3	10.0	8.4	2.0	1.625	15.0	-0.072	0.0	4.017	5.0	59.0
"Haute Kotto"	8.1	10.0	0.411	0.0	0.9	4.0	38.1	10.0	4.3	0.0	1.663	15.0	0.105	1.0	3.391	1.0	41.0
"Lobaye"	5.9	10.0	0.785	0.0	0.98	0.0	83.9	10.0	9.5	2.0	1.603	15.0	-0.223	3.0	3.452	1.0	41.0
"Mambere Kadei"	9.7	10.0	0.862	0.0	0.97	2.0	57.2	10.0	4.7	0.0	1.568	6.0	-0.175	1.0	4.113	5.0	34.0
"Mbomou"	12.6	20.0	0.932	0.0	0.92	4.0	76.6	10.0	7.9	2.0	1.623	15.0	-0.389	5.0	3.969	5.0	61.0
"Nana Mambere"	17.1	20.0	0.299	0.0	0.93	4.0	63.7	10.0	7.5	2.0	1.774	15.0	-0.03	0.0	3.222	0.0	51.0
"Ombella Mpoko"	18.9	20.0	0.636	0.0	0.93	4.0	53.0	10.0	4.7	0.0	1.539	6.0	0.274	3.0	4.444	5.0	48.0

“Ouaka”	11.7	20.0	0.264	0.0	0.81	10.0	50.7	10.0	3.6	0.0	1.605	15.0	0.087	0.0	3.682	3.0	58.0
“Ouham Pende”	15.4	20.0	0.061	2.0	0.83	10.0	62.8	10.0	13.5	4.0	1.571	6.0	-0.063	0.0	3.226	0.0	52.0
“Ouham”	7.6	10.0	1	0.0	0.89	10.0	55.7	10.0	3.3	0.0	1.502	6.0	0.188	1.0	3.847	3.0	40.0
“Sangha Mbaere”	6.7	10.0	0.029	4.0	0.76	10.0	80.6	10.0	2.8	0.0	1.327	2.0	-0.208	3.0	3.747	3.0	42.0

Central African Rep 2010, regions	Missing/flagged		Sex ratio		Age ratio		Height DPS		Weight DPS		SD WHZ		Skew WHZ		Kurtosis WHZ		Total score
	%	score	p-value	score	value	score	value	score	value	score	SD	score	value	score	value	score	score
Baminigui Bangoran	11.7	20.0	0.885	0.0	.	5.0	10.1	4.0	5.5	2.0	1.124	0.0	-0.016	0.0	3.691	3.0	34.0
Bangui	11.9	20.0	0.681	0.0	0.87	10.0	26.3	10.0	8.8	2.0	1.215	2.0	0.014	0.0	3.918	5.0	49.0
Basse Kotto	6.2	10.0	0.173	0.0	1.05	4.0	4.6	0.0	3.8	0.0	1.02	0.0	-0.165	1.0	3.687	3.0	18.0
Haut Mbomou	9.1	10.0	0.641	0.0	.	5.0	7.3	2.0	5.8	2.0	1.026	0.0	-0.161	1.0	3.037	0.0	20.0
Haute-Kotto	4.2	5.0	0.052	2.0	1.05	4.0	10.7	4.0	3.2	0.0	1.057	0.0	0.154	1.0	3.646	3.0	19.0
Kémo	2.7	5.0	0.191	0.0	0.95	2.0	7.8	2.0	2.7	0.0	1.059	0.0	-0.058	0.0	3.582	1.0	10.0
Lobaye	7.8	10.0	0.592	0.0	0.99	0.0	6.0	2.0	4.7	0.0	1.251	2.0	-0.076	0.0	3.863	3.0	17.0
Mambere Kadei	6.8	10.0	0.026	4.0	0.86	10.0	4.1	0.0	6.0	2.0	1.155	0.0	-0.406	5.0	3.661	3.0	34.0
Mbomou	8.3	10.0	0.332	0.0	1.02	0.0	4.9	0.0	5.2	2.0	1.014	0.0	-0.198	1.0	3.55	1.0	14.0
Nana Grebizi	6.9	10.0	0.94	0.0	1.13	10.0	8.1	2.0	3.2	0.0	1.219	2.0	-0.06	0.0	3.562	1.0	25.0
Nana Mambéré	3.5	5.0	0.597	0.0	0.85	10.0	6.2	2.0	5.6	2.0	1.166	0.0	-0.176	1.0	3.541	1.0	21.0
Ombella Mpoko	2.9	5.0	0.134	0.0	0.98	0.0	7.3	2.0	3.7	0.0	1.147	0.0	-0.12	1.0	3.455	1.0	9.0
Ouaka	4.4	5.0	0.225	0.0	1.04	2.0	5.2	2.0	5.0	2.0	1.005	0.0	-0.249	3.0	3.392	1.0	15.0
Ouham	5.5	10.0	0.516	0.0	0.96	2.0	11.3	4.0	2.8	0.0	1.144	0.0	-0.3	3.0	3.096	0.0	19.0
Ouham Pende	5.6	10.0	0.115	0.0	0.86	10.0	8.8	2.0	6.2	2.0	1.148	0.0	-0.219	3.0	3.436	1.0	28.0
Sangha Mbaere	6.2	10.0	0.197	0.0	0.92	4.0	7.1	2.0	4.1	0.0	1.146	0.0	0.021	0.0	3.435	1.0	17.0
Vakaga	17.2	20.0	0.189	0.0	1.26	10.0	18.3	4.0	15.8	4.0	1.018	0.0	-0.388	5.0	3.261	0.0	43.0

Chad 2000, regions	Missing/flagged		Sex ratio		Age ratio		Height DPS		Weight DPS		SD WHZ		Skew WHZ		Kurtosis WHZ		Total score
	%	score	p-value	score	value	score	value	score	value	score	SD	score	value	score	value	score	score
Autres villes	4.7	5.0	0.264	0.0	0.83	10.0	13.0	4.0	1.5	0.0	1.406	6.0	-0.026	0.0	3.726	3.0	28.0
N'Djaména	2.0	0.0	0.837	0.0	0.93	4.0	13.8	4.0	4.1	0.0	1.242	2.0	-0.265	3.0	3.781	3.0	16.0
Rural	3.7	5.0	0.634	0.0	0.79	10.0	12.7	4.0	2.9	0.0	1.361	2.0	-0.067	0.0	3.515	1.0	22.0

Chad 2010, regions	Missing/flagged		Sex ratio		Age ratio		Height DPS		Weight DPS		SD WHZ		Skew WHZ		Kurtosis WHZ		Total score
	%	score	p-value	score	value	score	value	score	value	score	SD	score	value	score	value	score	score
Barh El Gazal	16.7	20.0	0.135	0.0	0.58	10.0	22.1	10.0	3.7	0.0	1.423	6.0	0.058	0.0	3.389	1.0	47.0
Bhata	16.0	20.0	0.559	0.0	0.73	10.0	10.6	4.0	3.7	0.0	1.363	2.0	0.082	0.0	3.522	1.0	37.0
Chari Baguirmi	13.3	20.0	0.5	0.0	0.63	10.0	22.8	10.0	5.3	2.0	1.42	6.0	0.017	0.0	3.483	1.0	49.0
Guéra	15.0	20.0	0.229	0.0	0.68	10.0	23.2	10.0	6.3	2.0	1.274	2.0	-0.249	3.0	3.567	1.0	48.0
Hadjer Lamis	18.0	20.0	0.604	0.0	0.64	10.0	18.4	4.0	4.3	0.0	1.494	6.0	-0.232	3.0	2.956	0.0	43.0
Kanem	23.7	20.0	0.23	0.0	0.53	10.0	18.1	4.0	3.0	0.0	1.334	2.0	0.011	0.0	4.043	5.0	41.0
Lac	17.6	20.0	0.331	0.0	0.62	10.0	17.0	4.0	4.9	0.0	1.254	2.0	-0.168	1.0	3.575	1.0	38.0
Logone Occidental	17.7	20.0	0.487	0.0	0.81	10.0	28.9	10.0	6.8	2.0	1.428	6.0	-0.089	0.0	3.9	5.0	53.0
Logone Oriental	10.8	20.0	0.015	4.0	0.73	10.0	12.6	4.0	7.8	2.0	1.497	6.0	-0.518	5.0	3.246	0.0	51.0
Mandoul	10.0	20.0	0.59	0.0	0.77	10.0	11.8	4.0	1.7	0.0	1.285	2.0	-0.36	5.0	3.784	3.0	44.0
Mayo Kebbi Est	16.6	20.0	0.977	0.0	0.64	10.0	8.4	2.0	3.8	0.0	1.377	2.0	-0.465	5.0	3.386	1.0	40.0
Mayo Kebbi Ouest	13.2	20.0	0.842	0.0	0.91	4.0	39.9	10.0	3.7	0.0	1.328	2.0	-0.528	5.0	3.407	1.0	42.0
Moyen Chari	10.7	20.0	0.376	0.0	0.75	10.0	23.9	10.0	8.4	2.0	1.251	2.0	-0.579	5.0	3.683	3.0	52.0
Ndjaména	20.4	20.0	0.726	0.0	0.95	4.0	26.1	10.0	5.4	2.0	1.331	2.0	0.051	0.0	3.988	5.0	43.0
Ouaddai	18.1	20.0	0.211	0.0	0.72	10.0	25.1	10.0	10.8	4.0	1.433	6.0	-0.002	0.0	3.634	3.0	53.0
Salamat	21.8	20.0	0.496	0.0	0.62	10.0	56.0	10.0	9.3	2.0	1.45	6.0	0.064	0.0	3.72	3.0	51.0
Sila	20.6	20.0	0.226	0.0	0.56	10.0	14.1	4.0	7.7	2.0	1.704	15.0	0.113	1.0	3.303	1.0	53.0
Tandjilé	20.8	20.0	0.196	0.0	0.69	10.0	43.7	10.0	4.1	0.0	1.475	6.0	-0.367	5.0	3.804	3.0	54.0
Wad Fira	16.7	20.0	0.089	2.0	0.63	10.0	10.1	4.0	4.5	0.0	1.272	2.0	0.061	0.0	4.091	5.0	43.0
bet	25.3	20.0	0.56	0.0	0.59	10.0	10.8	4.0	5.4	2.0	1.41	6.0	0.334	5.0	3.474	1.0	48.0

Congo DR 2001, regions	Missing/flagged		Sex ratio		Age ratio		Height DPS		Weight DPS		SD WHZ		Skew WHZ		Kurtosis WHZ		Total score
	%	score	p-value	score	value	score	value	score	value	score	SD	score	value	score	value	score	score
Bandundu	10.9	20.0	0.184	0.0	0.88	10.0	37.9	10.0	5.3	2.0	1.629	15.0	0.214	3.0	3.443	1.0	61.0
Bas-congo	6.8	10.0	0.086	2.0	1.01	0.0	42.5	10.0	2.2	0.0	1.432	6.0	-0.158	1.0	3.723	3.0	32.0
Equateur	8.4	10.0	0.1	0.0	0.97	0.0	37.2	10.0	6.5	2.0	1.694	15.0	-0.052	0.0	3.203	0.0	37.0
Kasai Occidental	6.8	10.0	0.061	2.0	0.97	2.0	39.0	10.0	4.3	0.0	1.678	15.0	0.052	0.0	2.962	0.0	39.0
Kasai Oriental	13.5	20.0	0.764	0.0	1.1	4.0	54.3	10.0	4.1	0.0	1.755	15.0	0.14	1.0	3.265	0.0	50.0
Katanga	9.2	10.0	0.575	0.0	0.99	0.0	35.1	10.0	4.2	0.0	1.62	15.0	0.046	0.0	3.214	0.0	35.0
Kinshasa	8.2	10.0	0.815	0.0	0.94	4.0	30.4	10.0	2.7	0.0	1.523	6.0	0.089	0.0	3.768	3.0	33.0
Maniema	14.5	20.0	0.711	0.0	0.94	4.0	45.4	10.0	8.9	2.0	1.856	15.0	0.002	0.0	3.138	0.0	51.0
Nord-Kivu	10.0	20.0	0.556	0.0	0.93	4.0	17.1	4.0	6.1	2.0	1.594	6.0	-0.093	0.0	3.211	0.0	36.0
Orientale	10.6	20.0	0.183	0.0	0.98	0.0	46.4	10.0	7.7	2.0	1.54	6.0	0.084	0.0	3.387	1.0	39.0
Sud-Kivu	11.8	20.0	0.136	0.0	0.99	0.0	48.2	10.0	14.0	4.0	1.696	15.0	-0.185	1.0	3.129	0.0	50.0

Congo DR 2010, regions	Missing/flagged		Sex ratio		Age ratio		Height DPS		Weight DPS		SD WHZ		Skew WHZ		Kurtosis WHZ		Total score
	%	score	p-value	score	value	score	value	score	value	score	SD	score	value	score	value	score	score
Bandundu	6.4	10.0	0.873	0.0	0.85	10.0	10.5	4.0	4.1	0.0	1.442	6.0	0.253	3.0	4.194	5.0	38.0
Bas congo	4.3	5.0	0.16	0.0	0.96	2.0	25.4	10.0	3.7	0.0	1.189	0.0	-0.269	3.0	3.367	1.0	21.0
Equateur	8.4	10.0	0.006	4.0	0.97	2.0	29.2	10.0	5.8	2.0	1.344	2.0	-0.124	1.0	3.846	3.0	34.0
Kasai Occidental	3.3	5.0	0.605	0.0	0.86	10.0	14.4	4.0	4.3	0.0	1.208	2.0	-0.119	1.0	3.851	3.0	25.0
Kasai Oriental	7.6	10.0	0.805	0.0	1.01	0.0	14.4	4.0	2.8	0.0	1.316	2.0	-0.289	3.0	4.418	5.0	24.0
Katanga	5.4	10.0	0.933	0.0	0.95	2.0	21.3	10.0	2.8	0.0	1.524	6.0	0.093	0.0	4.045	5.0	33.0
Kinshasa	5.6	10.0	0.228	0.0	1	0.0	20.3	10.0	5.0	2.0	1.192	0.0	-0.161	1.0	4.079	5.0	28.0
Maniema	8.1	10.0	0.301	0.0	0.88	10.0	14.1	4.0	1.4	0.0	1.484	6.0	0.131	1.0	3.789	3.0	34.0
Nord Kivu	9.8	10.0	0.792	0.0	0.85	10.0	17.9	4.0	4.5	0.0	1.403	6.0	-0.204	3.0	4.052	5.0	38.0
Province Orientale	7.5	10.0	0.738	0.0	0.8	10.0	14.2	4.0	2.5	0.0	1.392	2.0	-0.114	1.0	3.683	3.0	30.0
Sud Kivu	6.4	10.0	0.405	0.0	0.96	2.0	18.3	4.0	3.0	0.0	1.223	2.0	-0.13	1.0	3.811	3.0	22.0

Côte d'Ivoire 2006, regions	Missing/flagged		Sex ratio		Age ratio		Height DPS		Weight DPS		SD WHZ		Skew WHZ		Kurtosis WHZ		Total score
	%	score	p-value	score	value	score	value	score	value	score	SD	score	value	score	value	score	score
Centre	2.6	5.0	0.352	0.0	1.01	0.0	70.0	10.0	3.6	0.0	1.368	2.0	0.004	0.0	3.927	5.0	22.0
Centre Est	0.6	0.0	0.23	0.0	0.95	4.0	62.9	10.0	5.2	2.0	1.263	2.0	-0.388	5.0	4.016	5.0	28.0
Centre Nord	2.6	5.0	0.328	0.0	0.96	2.0	10.7	4.0	5.0	0.0	1.629	15.0	-0.034	0.0	2.923	0.0	26.0
Centre Ouest	3.0	5.0	0.339	0.0	0.87	10.0	39.2	10.0	5.2	2.0	1.501	6.0	-0.236	3.0	3.593	1.0	37.0
Nord	2.6	5.0	0.003	4.0	0.87	10.0	18.3	4.0	3.9	0.0	1.48	6.0	-0.226	3.0	3.414	1.0	33.0
Nord Est	5.8	10.0	0.899	0.0	0.88	10.0	70.9	10.0	13.8	4.0	1.555	6.0	0.131	1.0	3.451	1.0	42.0
Nord Ouest	10.0	20.0	0.278	0.0	1.01	0.0	27.5	10.0	6.7	2.0	1.717	15.0	0.288	3.0	3.472	1.0	51.0
Ouest	5.7	10.0	0.652	0.0	0.96	2.0	26.6	10.0	4.8	0.0	1.499	6.0	-0.009	0.0	3.716	3.0	31.0
Sud (sans ville d' Abidjan)	4.2	5.0	0.331	0.0	0.86	10.0	63.6	10.0	5.6	2.0	1.491	6.0	-0.056	0.0	3.618	3.0	36.0
Sud Ouest	4.7	5.0	0.101	0.0	0.98	0.0	49.4	10.0	3.9	0.0	1.474	6.0	-0.336	5.0	4.061	5.0	31.0
Ville Abidjan	3.8	5.0	0.873	0.0	1.01	0.0	57.4	10.0	9.4	2.0	1.442	6.0	0.009	0.0	3.911	5.0	28.0

Equatorial Guinea 2000, regions	Missing/flagged		Sex ratio		Age ratio		Height DPS		Weight DPS		SD WHZ		Skew WHZ		Kurtosis WHZ		Total score
	%	score	p-value	score	value	score	value	score	value	score	SD	score	value	score	value	score	score
Annobon	21.4	20.0	0.285	0.0	.	5.0	17.8	4.0	12.6	4.0	1.763	15.0	-1.22	5.0	3.307	1.0	54.0
Bioko Norte	28.1	20.0	0.968	0.0	0.77	10.0	18.7	4.0	7.2	2.0	1.71	15.0	-0.385	5.0	3.794	3.0	59.0
Bioko Sur	11.4	20.0	0.398	0.0	.	5.0	24.9	10.0	17.3	4.0	1.089	0.0	-0.179	1.0	2.246	3.0	43.0
Centro Sur	21.3	20.0	0.668	0.0	0.82	10.0	31.2	10.0	12.6	4.0	1.75	15.0	-0.129	1.0	2.991	0.0	60.0
Kie Ntem	14.4	20.0	0.859	0.0	0.97	2.0	33.0	10.0	5.5	2.0	1.54	6.0	-0.353	5.0	3.567	1.0	46.0
Litoral	17.8	20.0	0.079	2.0	0.87	10.0	22.2	10.0	10.6	4.0	1.827	15.0	-0.25	3.0	3.246	0.0	64.0
Wele Nzas	13.2	20.0	0.395	0.0	0.75	10.0	17.2	4.0	4.3	0.0	1.52	6.0	-0.1	0.0	3.992	5.0	45.0

Gambia 2000, regions	Missing/flagged		Sex ratio		Age ratio		Height DPS		Weight DPS		SD WHZ		Skew WHZ		Kurtosis WHZ		Total score
	%	score	p-value	score	value	score	value	score	value	score	SD	score	value	score	value	score	score
Banjul	30.3	20.0	0.04	4.0	1.16	10.0	33.8	10.0	14.2	4.0	1.32	2.0	0.156	1.0	3.88	3.0	54.0
Basse	22.1	20.0	0.226	0.0	1.04	2.0	10.6	4.0	6.8	2.0	1.163	0.0	-0.155	1.0	3.403	1.0	30.0
Brikama	25.5	20.0	0.423	0.0	1.07	4.0	22.9	10.0	15.5	4.0	1.129	0.0	-0.201	3.0	3.231	0.0	41.0
Janjabureh	22.2	20.0	0.714	0.0	.	5.0	31.2	10.0	14.3	4.0	1.585	6.0	0.136	1.0	3.166	0.0	46.0
Kanifing	34.5	20.0	0.046	4.0	0.94	4.0	32.8	10.0	18.2	4.0	1.304	2.0	-0.344	5.0	4.193	5.0	54.0
Kerewan	12.7	20.0	0.241	0.0	.	5.0	19.2	4.0	9.5	2.0	1.367	2.0	-0.158	1.0	4.115	5.0	39.0
Kuntaur	32.9	20.0	0.738	0.0	.	5.0	12.5	4.0	5.2	2.0	1.218	2.0	-0.213	3.0	3.544	1.0	37.0
Mansakonko	16.9	20.0	0.009	4.0	0.93	4.0	44.7	10.0	25.8	10.0	1.38	2.0	0.154	1.0	3.896	3.0	54.0

Gambia 2005, regions	Missing/flagged		Sex ratio		Age ratio		Height DPS		Weight DPS		SD WHZ		Skew WHZ		Kurtosis WHZ		Total score
	%	score	p-value	score	value	score	value	score	value	score	SD	score	value	score	value	score	score
Banjul	4.2	5.0	0.938	0.0	1.06	4.0	14.4	4.0	10.0	2.0	0.992	0.0	0.058	0.0	3.396	1.0	16.0
Basse	9.6	10.0	0.703	0.0	1.02	0.0	4.9	0.0	2.7	0.0	1.109	0.0	-0.016	0.0	4.196	5.0	15.0
Brikama	2.4	0.0	0.334	0.0	1.17	10.0	25.7	10.0	3.6	0.0	1.317	2.0	0.067	0.0	3.917	5.0	27.0
Janjanburay	0.4	0.0	0.716	0.0	1.12	10.0	6.0	2.0	2.6	0.0	1.042	0.0	-0.027	0.0	3.622	3.0	15.0
Kanifing	3.3	5.0	0.2	0.0	0.99	0.0	15.9	4.0	5.7	2.0	1.163	0.0	-0.064	0.0	3.295	0.0	11.0
Kerewan	2.9	5.0	0.233	0.0	1.26	10.0	59.2	10.0	5.0	2.0	1.392	2.0	0.093	0.0	3.862	3.0	32.0
Kuntaur	10.7	20.0	0.475	0.0	1.27	10.0	8.5	2.0	5.0	0.0	1.302	2.0	-0.19	1.0	3.598	1.0	36.0
Mansakonko	2.2	0.0	0.274	0.0	1.15	10.0	11.3	4.0	3.3	0.0	1.192	0.0	-0.103	1.0	4.227	5.0	20.0

Ghana 2006, regions	Missing/flagged		Sex ratio		Age ratio		Height DPS		Weight DPS		SD WHZ		Skew WHZ		Kurtosis WHZ		Total score
	%	score	p-value	score	value	score	value	score	value	score	SD	score	value	score	value	score	score
Ashanti	7.0	10.0	0.698	0.0	0.82	10.0	23.9	10.0	3.3	0.0	1.228	2.0	0.046	0.0	4.315	5.0	37.0
Brong Ahafo	2.9	5.0	0.565	0.0	0.6	10.0	13.8	4.0	6.7	2.0	1.126	0.0	-0.081	0.0	3.337	1.0	22.0
Central	3.8	5.0	0.853	0.0	0.67	10.0	8.9	2.0	5.9	2.0	1.018	0.0	-0.085	0.0	3.243	0.0	19.0
Eastern	5.5	10.0	0.132	0.0	0.95	4.0	10.9	4.0	4.4	0.0	1.131	0.0	0.039	0.0	5.964	5.0	23.0

Greater Accra	9.1	10.0	0.152	0.0	0.83	10.0	7.4	2.0	6.2	2.0	1.141	0.0	-0.433	5.0	4.498	5.0	34.0
Northern	5.9	10.0	0.436	0.0	1.05	2.0	10.8	4.0	3.7	0.0	1.222	2.0	-0.229	3.0	3.996	5.0	26.0
Upper East	10.3	20.0	0.802	0.0	0.78	10.0	9.5	2.0	5.4	2.0	1.354	2.0	-0.114	1.0	3.857	3.0	40.0
Upper West	5.3	10.0	0.44	0.0	0.8	10.0	5.5	2.0	4.0	0.0	1.203	2.0	-0.263	3.0	3.645	3.0	30.0
Volta	10.2	20.0	0.482	0.0	0.89	10.0	15.1	4.0	10.3	4.0	1.163	0.0	-0.45	5.0	3.418	1.0	44.0
Western	2.8	5.0	0.78	0.0	0.82	10.0	7.3	2.0	3.7	0.0	1.122	0.0	-0.075	0.0	4.116	5.0	22.0

Ghana 2011, regions	Missing/flagged		Sex ratio		Age ratio		Height DPS		Weight DPS		SD WHZ		Skew WHZ		Kurtosis WHZ		Total score
	%	score	p-value	score	value	score	value	score	value	score	SD	score	value	score	value	score	score
Asante	4.0	5.0	0.169	0.0	0.8	10.0	17.0	4.0	3.9	0.0	1.243	2.0	0.087	0.0	3.768	3.0	24.0
Brong Ahafo	3.2	5.0	1	0.0	0.8	10.0	14.9	4.0	4.7	0.0	1.054	0.0	-0.007	0.0	3.66	3.0	22.0
Central	3.1	5.0	0.244	0.0	0.78	10.0	13.9	4.0	2.5	0.0	1.091	0.0	-0.052	0.0	4.063	5.0	24.0
Eastern	2.3	0.0	0.914	0.0	0.75	10.0	16.5	4.0	5.2	2.0	1.176	0.0	0.092	0.0	4.209	5.0	21.0
Greater Accra	4.0	5.0	0.764	0.0	0.84	10.0	29.3	10.0	4.9	0.0	1.128	0.0	0.727	5.0	5.02	5.0	35.0
Northern	2.8	5.0	0.045	4.0	0.79	10.0	21.3	10.0	2.0	0.0	1.137	0.0	-0.187	1.0	3.558	1.0	31.0
Upper East	3.5	5.0	0.975	0.0	0.67	10.0	10.1	4.0	3.7	0.0	1.101	0.0	0.062	0.0	5.544	5.0	24.0
Upper West	5.6	10.0	0.007	4.0	0.78	10.0	19.1	4.0	2.4	0.0	1.166	0.0	0.072	0.0	4.301	5.0	33.0
Volta	1.5	0.0	0.425	0.0	0.69	10.0	64.6	10.0	9.2	2.0	1.175	0.0	0.179	1.0	4.325	5.0	28.0
Western	7.8	10.0	0.108	0.0	0.84	10.0	8.5	2.0	5.9	2.0	1.214	2.0	0.146	1.0	4.281	5.0	32.0

Guinea Bissau 2000, regions	Missing/flagged		Sex ratio		Age ratio		Height DPS		Weight DPS		SD WHZ		Skew WHZ		Kurtosis WHZ		Total score
	%	score	p-value	score	value	score	value	score	value	score	SD	score	value	score	value	score	score
Bafatá	11.4	20.0	0.585	0.0	0.88	10.0	57.5	10.0	23.9	10.0	1.439	6.0	0.091	0.0	3.88	3.0	59.0
Biombo	3.0	5.0	0.033	4.0	0.98	0.0	57.4	10.0	14.0	4.0	1.574	6.0	0.053	0.0	3.39	1.0	30.0
Bolama/Bijagós	6.4	10.0	0.022	4.0	1.06	4.0	65.6	10.0	7.1	2.0	1.301	2.0	-0.355	5.0	3.76	3.0	40.0
Cacheu	3.6	5.0	0.801	0.0	0.98	0.0	58.1	10.0	6.8	2.0	1.445	6.0	-0.146	1.0	3.866	3.0	27.0
Gabú	14.8	20.0	0.428	0.0	0.77	10.0	88.8	10.0	30.3	10.0	1.584	6.0	0.02	0.0	3.165	0.0	56.0
Oio	4.9	5.0	0.246	0.0	0.85	10.0	82.1	10.0	4.9	0.0	1.517	6.0	-0.167	1.0	3.465	1.0	33.0
Quinará	8.8	10.0	0.846	0.0	0.78	10.0	55.3	10.0	3.6	0.0	1.386	2.0	0.055	0.0	3.424	1.0	33.0
Tombali	8.5	10.0	0.614	0.0	0.86	10.0	41.3	10.0	5.1	2.0	1.402	6.0	-0.206	3.0	3.911	5.0	46.0
sab	10.0	10.0	0.93	0.0	0.87	10.0	28.9	10.0	3.7	0.0	1.356	2.0	-0.087	0.0	3.577	1.0	33.0

Guinea Bissau 2006, regions	Missing/flagged		Sex ratio		Age ratio		Height DPS		Weight DPS		SD WHZ		Skew WHZ		Kurtosis WHZ		Total score
	%	score	p-value	score	value	score	value	score	value	score	SD	score	value	score	value	score	score
EST (Bafata e Gabu)	28.0	20.0	0.144	0.0	1.03	0.0	77.7	10.0	12.7	4.0	1.844	15.0	0.085	0.0	3.111	0.0	49.0
NORD (Biombo, Cacheu e Oio)	24.1	20.0	0.081	2.0	1.01	0.0	63.5	10.0	12.7	4.0	1.773	15.0	-0.101	1.0	3.516	1.0	53.0
SAB Capital	33.8	20.0	0.233	0.0	0.97	2.0	74.9	10.0	47.3	10.0	1.643	15.0	-0.433	5.0	4.213	5.0	67.0
SUD (Bolama, Quinara e Tombali)	45.6	20.0	0.121	0.0	0.95	2.0	72.9	10.0	21.0	10.0	2.016	15.0	-0.259	3.0	3.101	0.0	60.0

Mauritania 2007, regions	Missing/flagged		Sex ratio		Age ratio		Height DPS		Weight DPS		SD WHZ		Skew WHZ		Kurtosis WHZ		Total score
	%	score	p-value	score	value	score	value	score	value	score	SD	score	value	score	value	score	score
Adrar	14.3	20.0	0.299	0.0	0.99	0.0	36.0	10.0	6.5	2.0	1.193	0.0	0.244	3.0	4.229	5.0	40.0
Assaba	11.9	20.0	0.396	0.0	0.95	2.0	32.8	10.0	3.3	0.0	1.273	2.0	0.38	5.0	4.426	5.0	44.0
Brakna	11.9	20.0	0.84	0.0	1.03	2.0	63.2	10.0	5.4	2.0	1.324	2.0	0.053	0.0	3.946	5.0	41.0
Gorgol	13.2	20.0	0.695	0.0	0.91	4.0	59.2	10.0	4.8	0.0	1.306	2.0	0.164	1.0	4.141	5.0	42.0
Guidimagha	13.9	20.0	0.313	0.0	0.79	10.0	17.5	4.0	3.2	0.0	1.278	2.0	0.294	3.0	4.456	5.0	44.0
Hodh ECharghi	14.7	20.0	0.08	2.0	1.04	2.0	48.0	10.0	3.3	0.0	1.343	2.0	0.002	0.0	4.463	5.0	41.0
Hodh ELGharbi	8.8	10.0	0.338	0.0	1.06	4.0	17.7	4.0	3.8	0.0	1.225	2.0	0.077	0.0	4.671	5.0	25.0
Inchiri	9.2	10.0	0.599	0.0	.	5.0	36.9	10.0	10.6	4.0	1.227	2.0	-0.462	5.0	3.908	5.0	41.0
Nouadhibou	8.0	10.0	0.221	0.0	.	5.0	44.4	10.0	2.9	0.0	1.227	2.0	-0.13	1.0	3.724	3.0	31.0
Nouakchott	16.3	20.0	0.172	0.0	0.96	2.0	36.3	10.0	3.2	0.0	1.255	2.0	0.195	1.0	4.441	5.0	40.0
Tagant	10.9	20.0	0.955	0.0	.	5.0	44.1	10.0	8.1	2.0	1.458	6.0	0.573	5.0	4.894	5.0	53.0
Tiris Zemmour	6.8	10.0	0.254	0.0	.	5.0	76.9	10.0	5.6	2.0	1.235	2.0	0.288	3.0	3.775	3.0	35.0
Trarza	7.8	10.0	0.523	0.0	0.87	10.0	18.8	4.0	4.2	0.0	1.188	0.0	0.207	3.0	3.972	5.0	32.0

Niger 2000, regions	Missing/flagged		Sex ratio		Age ratio		Height DPS		Weight DPS		SD WHZ		Skew WHZ		Kurtosis WHZ		Total score
	%	score	p-value	score	value	score	value	score	value	score	SD	score	value	score	value	score	score
Diffa	7.4	10.0	1	0.0	.	5.0	15.4	4.0	12.7	4.0	1.371	2.0	0.162	1.0	2.925	0.0	26.0
Agadez	3.3	5.0	0.495	0.0	0.77	10.0	10.9	4.0	5.3	2.0	1.049	0.0	0.266	3.0	4.791	5.0	29.0
Dosso	7.4	10.0	0.629	0.0	0.78	10.0	7.5	2.0	3.2	0.0	1.335	2.0	-0.413	5.0	3.886	3.0	32.0
Maradi	4.0	5.0	0.07	2.0	0.83	10.0	11.3	4.0	2.7	0.0	1.267	2.0	-0.225	3.0	3.685	3.0	29.0
Niamey	5.2	10.0	0.299	0.0	0.85	10.0	9.1	2.0	4.5	0.0	1.18	0.0	-0.147	1.0	3.578	1.0	24.0
Tahoua	10.4	20.0	0.16	0.0	0.84	10.0	7.1	2.0	4.9	0.0	1.408	6.0	0.148	1.0	4.366	5.0	44.0
Tillaberi	4.2	5.0	0.18	0.0	0.8	10.0	8.3	2.0	3.8	0.0	1.281	2.0	-0.149	1.0	4.216	5.0	25.0
Zinder	9.4	10.0	0.585	0.0	0.87	10.0	6.5	2.0	4.6	0.0	1.37	2.0	-0.134	1.0	3.384	1.0	26.0

Nigeria 2007, regions	Missing/flagged		Sex ratio		Age ratio		Height DPS		Weight DPS		SD WHZ		Skew WHZ		Kurtosis WHZ		Total score

	%	score	p-value	score	value	score	value	score	value	score	SD	score	value	score	value	score	score
Abia	8.2	10.0	0.329	0.0	0.88	10.0	41.7	10.0	11.0	4.0	1.47	6.0	0.081	0.0	3.992	5.0	45.0
Abuja FCT	14.6	20.0	0.887	0.0	0.97	0.0	37.9	10.0	14.1	4.0	1.647	15.0	-0.07	0.0	3.901	5.0	54.0
Adamawa	22.6	20.0	0.3	0.0	1.12	10.0	64.3	10.0	28.8	10.0	2.075	15.0	-0.587	5.0	2.508	1.0	71.0
Akwa-Ibom	10.3	20.0	0.556	0.0	0.88	10.0	53.1	10.0	6.6	2.0	1.44	6.0	-0.005	0.0	4.171	5.0	53.0
Anambra	20.5	20.0	0.012	4.0	0.71	10.0	56.2	10.0	15.5	4.0	1.499	6.0	-0.159	1.0	4.713	5.0	60.0
Bauchi	21.0	20.0	0.354	0.0	0.83	10.0	54.0	10.0	19.9	4.0	2.068	15.0	-0.484	5.0	2.525	1.0	65.0
Bayelsa	10.8	20.0	0.376	0.0	0.95	4.0	56.9	10.0	5.0	0.0	1.432	6.0	0.029	0.0	4.7	5.0	45.0
Benue	12.7	20.0	0.663	0.0	0.83	10.0	61.9	10.0	5.3	2.0	1.461	6.0	-0.095	0.0	4.48	5.0	53.0
Borno	22.2	20.0	0.713	0.0	1.01	0.0	45.6	10.0	29.1	10.0	2.112	15.0	-0.095	0.0	2.659	1.0	56.0
Cross-Rivers	10.6	20.0	0.068	2.0	0.77	10.0	29.4	10.0	4.4	0.0	1.304	2.0	-0.19	1.0	3.307	1.0	46.0
Delta	5.4	10.0	0.043	4.0	0.85	10.0	69.7	10.0	7.9	2.0	1.434	6.0	0.054	0.0	3.993	5.0	47.0
Ebonyi	18.3	20.0	0.678	0.0	0.8	10.0	22.2	10.0	7.2	2.0	1.667	15.0	0.049	0.0	3.626	3.0	60.0
Edo	9.2	10.0	0.172	0.0	0.77	10.0	68.3	10.0	5.7	2.0	1.451	6.0	-0.009	0.0	4.579	5.0	43.0
Ekiti	8.0	10.0	0.022	4.0	0.95	4.0	48.2	10.0	8.5	2.0	1.585	6.0	-0.368	5.0	3.72	3.0	44.0
Enugu	10.9	20.0	0.357	0.0	0.92	4.0	32.5	10.0	6.3	2.0	1.44	6.0	0.193	1.0	4.673	5.0	48.0
Gombe	26.5	20.0	0.737	0.0	0.98	0.0	51.6	10.0	56.3	10.0	2.454	15.0	-0.225	3.0	2.033	5.0	63.0
Imo	23.7	20.0	0.465	0.0	0.87	10.0	66.4	10.0	11.1	4.0	1.799	15.0	-0.191	1.0	3.389	1.0	61.0
Jigawa	16.3	20.0	0.002	4.0	1.06	4.0	55.9	10.0	6.6	2.0	1.901	15.0	-0.031	0.0	2.981	0.0	55.0
Kaduna	11.7	20.0	0.908	0.0	1.13	10.0	67.4	10.0	30.0	10.0	2.069	15.0	-0.174	1.0	2.797	0.0	66.0
Kano	24.6	20.0	0.513	0.0	.	5.0	47.4	10.0	18.8	4.0	2.061	15.0	-0.187	1.0	2.876	0.0	55.0
Katsina	12.3	20.0	0.392	0.0	0.94	4.0	48.6	10.0	18.6	4.0	2.033	15.0	0.175	1.0	2.778	0.0	54.0
Kebbi	24.8	20.0	0.756	0.0	1.28	10.0	69.1	10.0	31.3	10.0	2.141	15.0	-0.005	0.0	2.555	1.0	66.0
Kogi	9.5	10.0	0.58	0.0	0.65	10.0	24.4	10.0	6.8	2.0	1.538	6.0	-0.059	0.0	3.598	1.0	39.0
Kwara	10.9	20.0	0.509	0.0	0.66	10.0	28.3	10.0	7.7	2.0	1.683	15.0	0.028	0.0	3.607	3.0	60.0
Lagos	18.5	20.0	0.957	0.0	0.8	10.0	78.2	10.0	8.5	2.0	1.76	15.0	-0.178	1.0	3.736	3.0	61.0
Nasarawa	23.2	20.0	0.346	0.0	0.98	0.0	17.8	4.0	10.0	2.0	1.956	15.0	-0.19	1.0	2.934	0.0	42.0
Niger	10.4	20.0	0.871	0.0	0.63	10.0	44.4	10.0	18.5	4.0	1.95	15.0	-0.075	0.0	2.744	0.0	59.0
Ogun	11.7	20.0	0.272	0.0	0.95	4.0	39.0	10.0	6.2	2.0	1.73	15.0	0.001	0.0	3.337	1.0	52.0
Ondo	14.1	20.0	0.453	0.0	0.69	10.0	15.2	4.0	9.2	2.0	1.704	15.0	0.026	0.0	4.22	5.0	56.0
Osun	11.2	20.0	0.704	0.0	0.74	10.0	16.5	4.0	5.1	2.0	1.403	6.0	0.108	1.0	4.262	5.0	48.0
Oyo	11.2	20.0	0.29	0.0	1.01	0.0	11.8	4.0	8.0	2.0	1.566	6.0	0.127	1.0	3.541	1.0	34.0
Plataeu	14.3	20.0	0.546	0.0	1.03	2.0	46.0	10.0	9.5	2.0	1.89	15.0	-0.307	5.0	2.834	0.0	54.0
Rivers	18.4	20.0	0.091	2.0	0.89	10.0	47.7	10.0	29.6	10.0	1.699	15.0	-0.082	0.0	3.933	5.0	72.0
Sokoto	25.7	20.0	0.202	0.0	0.76	10.0	76.6	10.0	23.4	10.0	2.073	15.0	-0.375	5.0	2.879	0.0	70.0
Taraba	21.1	20.0	0.15	0.0	0.97	2.0	50.6	10.0	18.0	4.0	1.946	15.0	-0.326	5.0	2.88	0.0	56.0
Yobe	21.4	20.0	0.196	0.0	1.15	10.0	50.7	10.0	22.8	10.0	2.102	15.0	-0.15	1.0	2.298	3.0	69.0
Zamfara	21.7	20.0	0.118	0.0	0.93	4.0	71.1	10.0	24.5	10.0	1.797	15.0	-0.033	0.0	3.385	1.0	60.0

Nigeria 2011, regions	Missing/flagged		Sex ratio		Age ratio		Height DPS		Weight DPS		SD WHZ		Skew WHZ		Kurtosis WHZ		Total score
	%	score	p-value	score	value	score	value	score	value	score	SD	score	value	score	value	score	score
Abia	1.7	0.0	0.547	0.0	0.85	10.0	12.3	4.0	7.3	2.0	1.193	0.0	-0.129	1.0	3.54	1.0	18.0
Adamawa	11.3	20.0	0.761	0.0	0.78	10.0	5.5	2.0	3.2	0.0	1.131	0.0	-0.204	3.0	3.572	1.0	36.0
Akwa ibom	3.5	5.0	0.933	0.0	0.76	10.0	26.1	10.0	5.3	2.0	1.089	0.0	0.286	3.0	4.423	5.0	35.0
Anambra	3.4	5.0	0.353	0.0	0.71	10.0	11.5	4.0	5.7	2.0	1.087	0.0	-0.212	3.0	4.266	5.0	29.0
Bauchi	8.4	10.0	0.269	0.0	0.83	10.0	18.9	4.0	6.9	2.0	1.245	2.0	-0.262	3.0	3.347	1.0	32.0
Bayelsa	6.5	10.0	0.61	0.0	0.82	10.0	7.4	2.0	4.6	0.0	1.105	0.0	-0.014	0.0	3.531	1.0	23.0
Benue	6.0	10.0	0.968	0.0	0.79	10.0	52.5	10.0	5.7	2.0	1.286	2.0	0.05	0.0	4.61	5.0	39.0
Borno	16.8	20.0	0.019	4.0	0.7	10.0	5.3	2.0	5.2	2.0	1.36	2.0	-0.099	0.0	3.821	3.0	43.0
Cross River	7.3	10.0	0.651	0.0	0.89	10.0	8.5	2.0	4.4	0.0	1.154	0.0	0.251	3.0	4.387	5.0	30.0
Delta	5.3	10.0	0.307	0.0	0.83	10.0	9.8	2.0	4.6	0.0	1.26	2.0	-0.048	0.0	3.757	3.0	27.0
Ebonyi	5.8	10.0	0.909	0.0	0.96	2.0	9.3	2.0	2.5	0.0	1.101	0.0	-0.238	3.0	4.617	5.0	22.0
Edo	3.3	5.0	0.895	0.0	0.74	10.0	18.4	4.0	4.5	0.0	1.125	0.0	-0.019	0.0	4.234	5.0	24.0
Ekiti	7.5	10.0	0.69	0.0	0.86	10.0	12.7	4.0	5.5	2.0	1.163	0.0	-0.131	1.0	3.693	3.0	30.0
Enugu	2.3	0.0	0.67	0.0	0.87	10.0	33.0	10.0	4.5	0.0	1.187	0.0	-0.034	0.0	3.908	5.0	25.0
FCT (Abuja)	7.3	10.0	0.012	4.0	0.97	2.0	5.9	2.0	6.3	2.0	1.153	0.0	-0.116	1.0	3.712	3.0	24.0
Gombe	8.2	10.0	0.822	0.0	0.79	10.0	8.1	2.0	4.8	0.0	1.353	2.0	-0.227	3.0	3.7	3.0	30.0
Imo	2.2	0.0	0.326	0.0	0.85	10.0	15.5	4.0	3.2	0.0	1.061	0.0	-0.2	1.0	3.496	1.0	16.0
Jigawa	7.5	10.0	0.149	0.0	0.68	10.0	12.2	4.0	3.6	0.0	1.467	6.0	-0.081	0.0	3.662	3.0	33.0
Kaduna	6.3	10.0	0.107	0.0	0.75	10.0	6.3	2.0	5.1	2.0	1.317	2.0	-0.381	5.0	3.75	3.0	34.0
Kano	7.4	10.0	0.698	0.0	0.8	10.0	10.9	4.0	2.7	0.0	1.318	2.0	-0.056	0.0	3.594	1.0	27.0
Katsina	8.0	10.0	0.349	0.0	0.74	10.0	11.7	4.0	4.9	0.0	1.459	6.0	0.034	0.0	3.683	3.0	33.0
Kebbi	10.2	20.0	0.673	0.0	0.77	10.0	11.4	4.0	5.9	2.0	1.395	2.0	-0.002	0.0	3.538	1.0	39.0
Kogi	2.3	0.0	0.5	0.0	0.86	10.0	36.0	10.0	5.2	2.0	1.354	2.0	0.042	0.0	4.062	5.0	29.0
Kwara	3.9	5.0	0.043	4.0	0.83	10.0	6.1	2.0	5.8	2.0	1.245	2.0	-0.041	0.0	3.906	5.0	30.0
Lagos	6.4	10.0	0.184	0.0	1.06	4.0	8.6	2.0	6.1	2.0	1.204	2.0	0.347	5.0	5.065	5.0	30.0
Nasarawa	16.8	20.0	0.366	0.0	0.86	10.0	9.8	2.0	2.9	0.0	1.229	2.0	0.175	1.0	3.783	3.0	38.0

Niger	7.7	10.0	0.146	0.0	0.67	10.0	34.4	10.0	6.2	2.0	1.507	6.0	-0.1	0.0	3.409	1.0	39.0
Ogun	9.5	10.0	0.364	0.0	0.99	0.0	13.5	4.0	4.7	0.0	1.156	0.0	-0.187	1.0	4.13	5.0	20.0
Ondo	6.8	10.0	0.358	0.0	0.88	10.0	40.4	10.0	11.7	4.0	1.417	6.0	0.002	0.0	3.85	3.0	43.0
Osun	2.9	5.0	0.779	0.0	0.91	4.0	15.2	4.0	4.3	0.0	1.031	0.0	-0.372	5.0	4.241	5.0	23.0
Oyo	10.3	20.0	0.324	0.0	0.82	10.0	21.1	10.0	3.8	0.0	1.327	2.0	0.334	5.0	4.467	5.0	52.0
Plateau	8.0	10.0	0.052	2.0	0.81	10.0	12.4	4.0	5.3	2.0	1.173	0.0	-0.167	1.0	3.135	0.0	29.0
Rivers	5.8	10.0	0.677	0.0	0.83	10.0	12.7	4.0	4.8	0.0	1.347	2.0	0.291	3.0	4.338	5.0	34.0
Sokoto	3.5	5.0	0.95	0.0	0.74	10.0	12.3	4.0	6.6	2.0	1.533	6.0	-0.139	1.0	2.93	0.0	28.0
Taraba	17.4	20.0	0.065	2.0	0.76	10.0	8.6	2.0	4.2	0.0	1.238	2.0	-0.374	5.0	4.333	5.0	46.0
Yobe	9.5	10.0	0.221	0.0	0.68	10.0	13.6	4.0	4.4	0.0	1.322	2.0	-0.193	1.0	3.674	3.0	30.0
Zamfara	9.8	10.0	0.185	0.0	0.75	10.0	15.5	4.0	6.4	2.0	1.477	6.0	-0.193	1.0	2.951	0.0	33.0

Sao Tome et Principe 2000, regions	Missing/flagged		Sex ratio		Age ratio		Height DPS		Weight DPS		SD WHZ		Skew WHZ		Kurtosis WHZ		Total score
	%	score	p-value	score	value	score	value	score	value	score	SD	score	value	score	value	score	score
Centro	25.1	20.0	0.218	0.0	0.87	10.0	84.3	10.0	47.1	10.0	1.376	2.0	-0.117	1.0	4.032	5.0	58.0
Norte	22.9	20.0	0.732	0.0	0.89	10.0	79.3	10.0	46.9	10.0	1.402	6.0	0.282	3.0	3.871	3.0	62.0
Principe	17.3	20.0	0.23	0.0	.	5.0	55.3	10.0	38.5	10.0	0.991	0.0	-0.264	3.0	3.948	5.0	53.0
Sul	23.8	20.0	0.351	0.0	.	5.0	46.9	10.0	42.4	10.0	1.192	0.0	-0.408	5.0	4.929	5.0	55.0

Senegal 2000, regions	Missing/flagged		Sex ratio		Age ratio		Height DPS		Weight DPS		SD WHZ		Skew WHZ		Kurtosis WHZ		Total score
	%	score	p-value	score	value	score	value	score	value	score	SD	score	value	score	value	score	score
Dakar	11.6	20.0	0.879	0.0	0.96	2.0	21.7	10.0	18.9	4.0	1.411	6.0	0.071	0.0	3.958	5.0	47.0
Diourbel	5.8	10.0	0.064	2.0	0.69	10.0	18.2	4.0	21.4	10.0	1.315	2.0	0.191	1.0	3.562	1.0	40.0
Fatick	5.9	10.0	0.69	0.0	0.78	10.0	23.9	10.0	9.2	2.0	1.215	2.0	-0.16	1.0	3.984	5.0	40.0
Kaolack	6.8	10.0	0.847	0.0	0.81	10.0	22.6	10.0	19.7	4.0	1.302	2.0	-0.257	3.0	3.523	1.0	40.0
Kolda	7.2	10.0	0.699	0.0	0.76	10.0	37.6	10.0	4.6	0.0	1.167	0.0	-0.325	5.0	3.609	3.0	38.0
Louga	5.9	10.0	0.621	0.0	0.9	10.0	6.0	2.0	3.0	0.0	1.171	0.0	0.047	0.0	4.443	5.0	27.0
Saint louis	5.5	10.0	0.763	0.0	0.8	10.0	5.8	2.0	3.6	0.0	1.169	0.0	-0.142	1.0	3.3	0.0	23.0
Tambacounda	9.0	10.0	0.713	0.0	0.82	10.0	18.2	4.0	4.9	0.0	1.302	2.0	-0.042	0.0	3.934	5.0	31.0
Thies	4.6	5.0	0.269	0.0	0.89	10.0	15.7	4.0	6.5	2.0	1.235	2.0	-0.009	0.0	4.286	5.0	28.0
Ziguinchor	7.6	10.0	0.772	0.0	0.75	10.0	33.1	10.0	4.3	0.0	1.17	0.0	0.056	0.0	4.923	5.0	35.0

Sierra Leone 2000, regions	Missing/flagged		Sex ratio		Age ratio		Height DPS		Weight DPS		SD WHZ		Skew WHZ		Kurtosis WHZ		Total score
	%	score	p-value	score	value	score	value	score	value	score	SD	score	value	score	value	score	score
East	22.1	20.0	0.791	0.0	0.97	2.0	24.7	10.0	5.5	2.0	1.407	6.0	-0.175	1.0	3.728	3.0	44.0
North	22.7	20.0	0.757	0.0	0.75	10.0	19.1	4.0	5.3	2.0	1.411	6.0	-0.036	0.0	4.165	5.0	47.0
South	16.2	20.0	0.711	0.0	1.1	4.0	18.5	4.0	5.7	2.0	1.77	15.0	-0.131	1.0	3.424	1.0	47.0
West	5.9	10.0	0.567	0.0	0.8	10.0	33.5	10.0	8.9	2.0	1.401	6.0	-0.174	1.0	3.965	5.0	44.0

Sierra Leone 2005, regions	Missing/flagged		Sex ratio		Age ratio		Height DPS		Weight DPS		SD WHZ		Skew WHZ		Kurtosis WHZ		Total score
	%	score	p-value	score	value	score	value	score	value	score	SD	score	value	score	value	score	score
East	30.9	20.0	0.466	0.0	0.84	10.0	46.7	10.0	6.3	2.0	1.478	6.0	-0.155	1.0	3.673	3.0	52.0
North	17.9	20.0	0.571	0.0	0.93	4.0	26.6	10.0	4.2	0.0	1.468	6.0	-0.184	1.0	3.928	5.0	46.0
South	15.3	20.0	0.621	0.0	0.89	10.0	13.2	4.0	2.3	0.0	1.527	6.0	-0.131	1.0	3.733	3.0	44.0
West	6.0	10.0	0.628	0.0	0.66	10.0	22.5	10.0	3.7	0.0	1.468	6.0	-0.003	0.0	3.648	3.0	39.0

Sierra Leone 2010, regions	Missing/flagged		Sex ratio		Age ratio		Height DPS		Weight DPS		SD WHZ		Skew WHZ		Kurtosis WHZ		Total score
	%	score	p-value	score	value	score	value	score	value	score	SD	score	value	score	value	score	score
East	14.5	20.0	0.803	0.0	0.84	10.0	28.3	10.0	3.2	0.0	1.51	6.0	-0.014	0.0	4.29	5.0	51.0
North	9.6	10.0	0.366	0.0	0.67	10.0	18.8	4.0	4.9	0.0	1.543	6.0	-0.004	0.0	3.551	1.0	31.0
South	12.1	20.0	0.154	0.0	0.76	10.0	26.5	10.0	4.1	0.0	1.578	6.0	0.122	1.0	3.467	1.0	48.0
West	18.1	20.0	0.953	0.0	0.74	10.0	28.7	10.0	10.1	4.0	1.763	15.0	0.155	1.0	3.16	0.0	60.0

Togo 2006, regions	Missing/flagged		Sex ratio		Age ratio		Height DPS		Weight DPS		SD WHZ		Skew WHZ		Kurtosis WHZ		Total score
	%	score	p-value	score	value	score	value	score	value	score	SD	score	value	score	value	score	score
Centrale	3.7	5.0	0.438	0.0	0.97	0.0	28.9	10.0	13.5	4.0	1.425	6.0	-0.198	1.0	4.298	5.0	31.0
Kara	9.5	10.0	0.01	4.0	0.88	10.0	53.9	10.0	57.6	10.0	1.873	15.0	0.066	0.0	2.781	0.0	59.0
Lomé commune	9.8	10.0	0.898	0.0	0.95	2.0	18.7	4.0	5.8	2.0	1.284	2.0	-0.115	1.0	4.487	5.0	26.0
Maritime (sans Lomé commune)	6.5	10.0	0.314	0.0	0.92	4.0	36.8	10.0	2.9	0.0	1.443	6.0	-0.088	0.0	3.79	3.0	33.0
Plateaux	10.7	20.0	0.121	0.0	0.97	0.0	38.6	10.0	11.4	4.0	1.532	6.0	-0.058	0.0	3.622	3.0	43.0
Savanes	8.4	10.0	0.328	0.0	1.01	0.0	30.5	10.0	45.6	10.0	1.43	6.0	-0.06	0.0	2.969	0.0	36.0

Togo 2010, regions	Missing/flagged		Sex ratio		Age ratio		Height DPS		Weight DPS		SD WHZ		Skew WHZ		Kurtosis WHZ		Total score
	%	score	p-value	score	value	score	value	score	value	score	SD	score	value	score	value	score	score
Centrale	5.3	10.0	0.223	0.0	0.91	4.0	4.3	0.0	2.3	0.0	1.039	0.0	-0.152	1.0	3.38	1.0	16.0
Kara	5.0	5.0	0.291	0.0	0.8	10.0	5.4	2.0	2.8	0.0	1.077	0.0	-0.282	3.0	3.825	3.0	23.0
Lomé	8.1	10.0	0.859	0.0	0.88	10.0	8.9	2.0	4.1	0.0	1.057	0.0	-0.046	0.0	3.554	1.0	23.0

Maritime	6.7	10.0	0.251	0.0	0.73	10.0	4.3	0.0	4.1	0.0	1.016	0.0	-0.115	1.0	4.083	5.0	26.0
Plateaux	3.8	5.0	0.161	0.0	0.87	10.0	8.2	2.0	3.0	0.0	1.006	0.0	-0.114	1.0	3.281	0.0	18.0
Savanes	2.4	0.0	0.189	0.0	0.8	10.0	3.8	0.0	2.9	0.0	1	0.0	-0.163	1.0	3.719	3.0	14.0
