# Supplementary material for: Child anthropometry data quality from Demographic and Health Surveys, Multiple Indicator Cluster Surveys, and National Nutrition Surveys in the West Central Africa region: are we comparing apples and oranges?
Source: Glob Health Action. 2017 Jun 22;10(1):1328185. doi: 10.1080/16549716.2017.1328185 (PMC5496063; doi:10.1080/16549716.2017.1328185)
Supplement: Supplementary Material [file zgha_a_1328185_sm3408.docx]

Supplemental Table 1. List of countries where Multiple Indicator Cluster Surveys (MICS), Demographic and Health Surveys (DHS), and National Nutrition Surveys (NNS) were conducted, separately, in West/Central Africa, which included anthropometric data from children aged 059 months.

| Country | MICS | DHS | | NNS | |
| --- | --- | --- | --- | --- | --- |
| Benin |  | X | | X | |
| Burkina Faso | X | X | | X | |
| Cameroon | X | X | |  | |
| Central African Republic | X | X | | X | |
| Chad | X | X | | X | |
| Congo |  | X | |  | |
| Côte d'Ivoire | X | X | |  | |
| Democratic Republic of Congo | X | X | |  | |
| Equatorial Guinea | | X |  | |  |
| Gabon |  | X | |  | |
| Gambia | X |  | |  | |
| Ghana | X | X | |  | |
| Guinea |  | X | | X | |
| GuineaBissau | | X | X | |  |
| Liberia |  | X | |  | |
| Mali |  | X | | X | |
| Mauritania |  |  | | X | |
| Niger | X | X | | X | |
| Nigeria | X | X | | X | |
| Sao Tome and Principe | X | X | |  | |
| Senegal | X | X | | X | |
| Sierra Leone | X | X | | X | |
| Togo | X | X | | X | |

Supplemental Table 2. Number of children aged 6-29 months, 30-59 months and the age ratio (6-29:30-59 months), and the number of males and female children and the sex ratio in MICS.

| Country, survey year | N of children aged 6-29 months | N of children aged 30-59 months | Age ratio | N of males | N of females | Sex ratio | Chi-square | p-value |
| --- | --- | --- | --- | --- | --- | --- | --- | --- |
| Burkina Faso 2006 | 2354 | 2386 | 0.99 | 2880 | 2795 | 1.03 | 1.27 | 0.2592 |
| Cameroon 2006 | 2741 | 2963 | 0.93 | 3222 | 3273 | 0.98 | 0.4 | 0.5269 |
| Central African Rep 2000 | 6218 | 6263 | 0.99 | 7292 | 7007 | 1.04 | 5.68 | 0.0172 |
| Central African Rep 2006 | 3855 | 4341 | 0.89 | 4935 | 4885 | 1.01 | 0.25 | 0.6139 |
| Central African Rep 2010 | 4512 | 4679 | 0.96 | 5380 | 5524 | 0.97 | 1.9 | 0.1679 |
| Chad 2000 | 2160 | 2612 | 0.83 | 2652 | 2732 | 0.97 | 1.19 | 0.2756 |
| Chad 2010 | 6160 | 8979 | 0.69 | 8909 | 8804 | 1.01 | 0.62 | 0.4301 |
| Congo DR 2001 | 4477 | 4614 | 0.97 | 5129 | 5125 | 1 | 0 | 0.9685 |
| Congo DR 2010 | 4688 | 5134 | 0.91 | 5673 | 5572 | 1.02 | 0.91 | 0.3409 |
| Côte d’Ivoire 2006 | 3715 | 3939 | 0.94 | 4413 | 4191 | 1.05 | 5.73 | 0.0167 |
| Equatorial Guinea 2000 | 1154 | 1358 | 0.85 | 1480 | 1469 | 1.01 | 0.04 | 0.8395 |
| Gambia 2000 | 1683 | 1527 | 1.1 | 1859 | 1773 | 1.05 | 2.04 | 0.1536 |
| Gambia 2005 | 2992 | 2696 | 1.11 | 3387 | 3254 | 1.04 | 2.66 | 0.1027 |
| Ghana 2006 | 1403 | 1681 | 0.83 | 1822 | 1723 | 1.06 | 2.76 | 0.0964 |
| Ghana 2011 | 2942 | 3816 | 0.77 | 3896 | 3730 | 1.04 | 3.61 | 0.0573 |
| Guinea Bissau 2000 | 2380 | 2721 | 0.87 | 2876 | 2977 | 0.97 | 1.74 | 0.1868 |
| Guinea Bissau 2006 | 2589 | 2598 | 1 | 3203 | 3367 | 0.95 | 4.09 | 0.043 |
| Mauritania 2007 | 3821 | 3926 | 0.97 | 4598 | 4382 | 1.05 | 5.2 | 0.0226 |
| Niger 2000 | 2024 | 2459 | 0.82 | 2611 | 2460 | 1.06 | 4.5 | 0.034 |
| Nigeria 2007 | 7084 | 7736 | 0.92 | 8687 | 8403 | 1.03 | 4.72 | 0.0298 |
| Nigeria 2011 | 9969 | 12509 | 0.8 | 13284 | 12734 | 1.04 | 11.63 | 0.0007 |
| Sao Tome et Principe 2000 | 890 | 1034 | 0.86 | 995 | 1070 | 0.93 | 2.72 | 0.0989 |
| Senegal 2000 | 3644 | 4431 | 0.82 | 4510 | 4554 | 0.99 | 0.21 | 0.644 |
| Sierra Leone 2000 | 1122 | 1312 | 0.86 | 1354 | 1350 | 1 | 0.01 | 0.9387 |
| Sierra Leone 2005 | 2203 | 2535 | 0.87 | 2936 | 2968 | 0.99 | 0.17 | 0.6771 |
| Sierra Leone 2010 | 3294 | 4459 | 0.74 | 4395 | 4400 | 1 | 0 | 0.9575 |
| Togo 2006 | 1771 | 1856 | 0.95 | 2102 | 2052 | 1.02 | 0.6 | 0.4379 |
| Togo 2010 | 1891 | 2292 | 0.83 | 2512 | 2396 | 1.05 | 2.74 | 0.0978 |

Supplemental Table 3. Number of children aged 6-29 months, 30-59 months and the age ratio (6-29:30-59 months), and the number of males and female children and the sex ratio in DHS.

| Country, survey year | N of children aged 6-29 months | N of children aged 30-59 months | Age ratio | N of males | N of females | Sex ratio | Chi-square | p-value |
| --- | --- | --- | --- | --- | --- | --- | --- | --- |
| Benin 2001 | 1933 | 2106 | 0.92 | 2372 | 2357 | 1.01 | 0.05 | 0.8273 |
| Benin 2006 | 6233 | 6418 | 0.97 | 7380 | 7302 | 1.01 | 0.41 | 0.5198 |
| Benin 2011 | 4950 | 6012 | 0.82 | 6491 | 6188 | 1.05 | 7.24 | 0.0071 |
| Burkina Faso 1993 | 2006 | 2450 | 0.82 | 2567 | 2508 | 1.02 | 0.69 | 0.4076 |
| Burkina Faso 1998 | 2017 | 2433 | 0.83 | 2567 | 2505 | 1.02 | 0.76 | 0.384 |
| Burkina Faso 2003 | 3742 | 4280 | 0.87 | 4804 | 4548 | 1.06 | 7.01 | 0.0081 |
| Burkina Faso 2010 | 2877 | 3191 | 0.9 | 6932 | 6784 | 1.02 | 1.6 | 0.2063 |
| Cameroon 1991 | 1231 | 1498 | 0.82 | 1525 | 1513 | 1.01 | 0.05 | 0.8277 |
| Cameroon 1998 | 1404 | 296 |  | 1053 | 1033 | 1.02 | 0.19 | 0.6615 |
| Cameroon 2011 | 2385 | 2308 | 1.03 | 5296 | 5438 | 0.97 | 1.88 | 0.1705 |
| Central African Rep 1994 | 1676 | 421 |  | 1286 | 1271 | 1.01 | 0.09 | 0.7667 |
| Chad 1996 | 2543 | 2980 | 0.85 | 3162 | 3176 | 1 | 0.03 | 0.8604 |
| Chad 2004 | 2029 | 2306 | 0.88 | 2466 | 2460 | 1 | 0.01 | 0.9319 |
| Congo 2005 | 1875 | 1776 | 1.06 | 2265 | 2170 | 1.04 | 2.03 | 0.1537 |
| Congo 2012 | 1960 | 2141 | 0.92 | 4465 | 4392 | 1.02 | 0.6 | 0.4379 |
| Congo DR 2007 | 1725 | 1744 | 0.99 | 3956 | 4031 | 0.98 | 0.7 | 0.4014 |
| Côte d'Ivoire 1994 | 2407 | 581 |  | 1834 | 1824 | 1.01 | 0.03 | 0.8687 |
| Côte d'Ivoire 1998 | 781 | 754 | 1.04 | 835 | 868 | 0.96 | 0.64 | 0.4239 |
| Côte d'Ivoire 2011 | 1514 | 1555 | 0.97 | 3503 | 3590 | 0.98 | 1.07 | 0.3016 |
| Gabon 2000 | 1743 | 1899 | 0.92 | 2052 | 2048 | 1 | 0 | 0.9502 |
| Gabon 2012 | 1610 | 1539 | 1.05 | 2857 | 2890 | 0.99 | 0.19 | 0.6633 |
| Ghana 1993 | 1354 | 315 |  | 1050 | 1006 | 1.04 | 0.94 | 0.3319 |
| Ghana 1998 | 1270 | 1440 | 0.88 | 1475 | 1540 | 0.96 | 1.4 | 0.2365 |
| Ghana 2003 | 1483 | 1596 | 0.93 | 1787 | 1743 | 1.03 | 0.55 | 0.459 |
| Ghana 2008 | 1158 | 1203 | 0.96 | 1420 | 1374 | 1.03 | 0.76 | 0.3842 |
| Guinea 1999 | 2019 | 2277 | 0.89 | 2589 | 2377 | 1.09 | 9.05 | 0.0026 |
| Guinea 2005 | 1168 | 1277 | 0.91 | 2856 | 2760 | 1.03 | 1.64 | 0.2002 |
| Guinea 2012 | 1391 | 1473 | 0.94 | 3304 | 3120 | 1.06 | 5.27 | 0.0217 |
| Liberia 2007 | 2041 | 2231 | 0.91 | 2721 | 2584 | 1.05 | 3.54 | 0.06 |
| Mali 1995 | 3325 | 846 |  | 2569 | 2649 | 0.97 | 1.23 | 0.2681 |
| Mali 2001 | 4287 | 4550 | 0.94 | 5318 | 5301 | 1 | 0.03 | 0.869 |
| Mali 2006 | 5160 | 5417 | 0.95 | 6247 | 6190 | 1.01 | 0.26 | 0.6093 |
| Niger 1992 | 2371 | 2465 | 0.96 | 2896 | 2656 | 1.09 | 10.37 | 0.0013 |
| Niger 1998 | 2824 | 576 |  | 2196 | 2030 | 1.08 | 6.52 | 0.0107 |
| Niger 2006 | 1692 | 1867 | 0.91 | 4173 | 4036 | 1.03 | 2.29 | 0.1305 |
| Niger 2012 | 2320 | 2588 | 0.9 | 5842 | 5760 | 1.01 | 0.58 | 0.4465 |
| Nigeria 1990 | 3151 | 3739 | 0.84 | 3387 | 3400 | 1 | 0.02 | 0.8746 |
| Nigeria 2003 | 2095 | 2109 | 0.99 | 2518 | 2490 | 1.01 | 0.16 | 0.6924 |
| Nigeria 2008 | 10568 | 10866 | 0.97 | 12872 | 12574 | 1.02 | 3.49 | 0.0617 |
| Sao Tome et Principe 2008 | 774 | 834 | 0.93 | 926 | 925 | 1 | 0 | 0.9815 |
| Senegal 1992 | 2031 | 2416 | 0.84 | 2445 | 2623 | 0.93 | 6.25 | 0.0124 |
| Senegal 2005 | 1386 | 1377 | 1.01 | 5141 | 4885 | 1.05 | 6.54 | 0.0106 |
| Senegal 2010 | 1825 | 1959 | 0.93 | 5948 | 5685 | 1.05 | 5.95 | 0.0148 |
| Sierra Leone 2008 | 1130 | 986 | 1.15 | 2552 | 2491 | 1.02 | 0.74 | 0.3903 |
| Togo 1998 | 2547 | 610 |  | 1899 | 1964 | 0.97 | 1.09 | 0.2957 |

Supplemental Table 4. Number of children aged 6-29 months, 30-59 months and the age ratio (6-29:30-59 months), and the number of males and female children and the sex ratio in NNS.

| Country, survey year | N of children aged 6-29 months | N of children aged 30-59 months | Age ratio | N of males | N of females | Sex ratio | Chi-square | p-value |
| --- | --- | --- | --- | --- | --- | --- | --- | --- |
| Benin 2008 | 1600 | 1864 | 0.86 | 1827 | 1719 | 1.06 | 3.29 | 0.07 |
| Burkina Faso 2012 | 9027 | 8960 | 1.01 | 9938 | 9413 | 1.06 | 14.24 | 0 |
| Cameroon 2011 | 613 | 730 | 0.84 | 674 | 669 | 1.01 | 0.02 | 0.892 |
| Central African Rep 2012 | 7387 | 6754 | 1.09 | 7912 | 7826 | 1.01 | 0.47 | 0.493 |
| Chad (7 regions) Dec/Jan 2012-13 | 2616 | 2662 | 0.98 | 3927 | 3846 | 1.05 | 5.19 | 0.023 |
| Chad June 2012 | 3410 | 3523 | 0.97 | 2942 | 2857 | 1.05 | 4.53 | 0.033 |
| Guinea-Bissau 2008 | 1293 | 1211 | 1.07 | 3990 | 3789 | 0.99 | 0.53 | 0.468 |
| Guinée Conakay 2012 | 3508 | 3161 | 1.11 | 1394 | 1324 | 1.04 | 3.39 | 0.066 |
| Liberia 2010 | 3197 | 3603 | 0.89 | 4132 | 4075 | 1.05 | 1.8 | 0.179 |
| Liberia 2011 | 1222 | 301 |  | 3443 | 3363 | 1.01 | 0.4 | 0.529 |
| Mali 2011 | 3965 | 3684 | 1.08 | 747 | 737 | 1.02 | 0.94 | 0.332 |
| Mauritania 2006 | 2001 | 1876 | 1.07 | 4205 | 4012 | 1.01 | 0.07 | 0.795 |
| Mauritania 2009 | 2157 | 1904 | 1.13 | 1970 | 1907 | 1.02 | 0.84 | 0.358 |
| Mauritania Dec 2011 | 4259 | 3964 | 1.07 | 3383 | 3002 | 1.03 | 1.25 | 0.264 |
| Mauritania Dec 2008 | 2925 | 2805 | 1.04 | 3272 | 3070 | 1.03 | 1.02 | 0.312 |
| Mauritania Dec 2010 | 2995 | 2842 | 1.05 | 2247 | 2131 | 1.13 | 22.73 | 0 |
| Mauritania July 2010 | 3294 | 3084 | 1.07 | 3226 | 3157 | 1.07 | 6.43 | 0.011 |
| Mauritania July 2011 | 3959 | 3782 | 1.05 | 3015 | 2836 | 1.05 | 3.07 | 0.08 |
| Mauritania July 2012 | 3185 | 3026 | 1.05 | 3923 | 3856 | 1.02 | 0.75 | 0.388 |
| Mauritania March 2008 | 3231 | 3154 | 1.02 | 4169 | 4004 | 1.06 | 5.48 | 0.019 |
| Niger 2012 | 4045 | 4263 | 0.95 | 3507 | 3310 | 1.02 | 0.58 | 0.448 |
| Nigeria (Northern States) 2011 | 2974 | 3190 | 0.93 | 4681 | 4544 | 1.04 | 3.33 | 0.068 |
| Senegal 2012 | 4235 | 4476 | 0.95 | 3507 | 3402 | 1.06 | 5.69 | 0.017 |
| Sierra Leone 2010 | 6652 | 6345 | 1.05 | 4706 | 4529 | 1.03 | 2.03 | 0.154 |
| The Gambia 2012 | 3533 | 3672 | 0.96 | 6966 | 7052 | 1.03 | 1.6 | 0.207 |
| Togo Dec 2012 | 673 | 671 | 1 | 1389 | 1370 | 1.01 | 0.13 | 0.718 |
| Togo June 2012 | 1220 | 1243 | 0.98 | 735 | 716 | 1.03 | 0.25 | 0.618 |

Supplemental Table 5. The prevalence of valid, missing, and implausible anthropometric data among children aged 059 months in 28 Multiple Indicator Cluster Surveys (MICS) in West Central Africa.

|  |  | The percentage of anthropometric data (height or weight) that was… | | | |
| --- | --- | --- | --- | --- | --- |
| Country, survey year | Total N of Children | Valid | Implausible | Missing | Implausible/Missing |
| Ghana 2011 | 7626 | 96.3 | 1.2 | 2.5 | 3.7 |
| Chad 2000 | 5384 | 96.2 | 2.0 | 1.8 | 3.8 |
| Côte d’Ivoire 2006 | 8604 | 95.7 | 3.7 | 0.6 | 4.3 |
| Gambia 2005 | 6641 | 95.7 | 0.5 | 3.8 | 4.3 |
| Togo 2010 | 4908 | 95.2 | 0.4 | 4.5 | 4.8 |
| Niger 2000 | 5080 | 93.8 | 2.3 | 3.9 | 6.2 |
| Central African Rep 2010 | 10904 | 93.7 | 0.6 | 5.7 | 6.3 |
| Ghana 2006 | 3545 | 93.6 | 1.8 | 4.5 | 6.4 |
| Congo DR 2010 | 11245 | 93.3 | 2.6 | 4.1 | 6.7 |
| Senegal 2000 | 9064 | 92.9 | 2.1 | 5.0 | 7.1 |
| Nigeria 2011 | 26018 | 92.2 | 1.1 | 6.8 | 7.8 |
| Togo 2006 | 4154 | 92.0 | 3.9 | 4.1 | 8.0 |
| Guinea Bissau 2000 | 5853 | 91.6 | 4.6 | 3.8 | 8.4 |
| Cameroon 2006 | 6495 | 90.2 | 3.2 | 6.6 | 9.8 |
| Congo DR 2001 | 10254 | 90.2 | 5.9 | 3.9 | 9.8 |
| Central African Rep 2000 | 14300 | 88.5 | 5.2 | 6.4 | 11.5 |
| Mauritania 2007 | 8981 | 87.7 | 2.9 | 9.4 | 12.3 |
| Sierra Leone 2010 | 8798 | 87.5 | 5.9 | 6.5 | 12.5 |
| Central African Rep 2006 | 9820 | 86.8 | 5.9 | 7.3 | 13.2 |
| Nigeria 2007 | 17093 | 83.4 | 11.1 | 5.5 | 16.6 |
| Chad 2010 | 17713 | 83.0 | 3.9 | 13.0 | 17.0 |
| Burkina Faso 2006 | 5677 | 82.4 | 7.2 | 10.4 | 17.6 |
| Sierra Leone 2000 | 2704 | 82.0 | 6.9 | 11.1 | 18.0 |
| Sierra Leone 2005 | 5904 | 80.6 | 5.8 | 13.6 | 19.4 |
| Sao Tome et Principe 2000 | 2208 | 75.8 | 3.1 | 21.1 | 24.2 |
| Gambia 2000 | 3632 | 74.7 | 9.1 | 16.2 | 25.3 |
| Guinea Bissau 2006 | 6570 | 69.3 | 12.1 | 18.6 | 30.7 |
| Equatorial Guinea 2000 | 2949 | 68.5 | 10.0 | 21.5 | 31.5 |

Supplemental Table 6. The prevalence of valid, missing, and implausible anthropometric data among children aged 059 months in 45 Demographic and Health Surveys (DHS) in West Central Africa.

|  |  | The percentage of anthropometric data (height or weight) that was… | | | |
| --- | --- | --- | --- | --- | --- |
| Country, survey year | Total N of Children | Valid | Implausible | Missing | Implausible/Missing |
| Congo 2012 | 8857 | 97.9 | 0.9 | 1.2 | 2.1 |
| Benin 2001 | 4729 | 97.6 | 1.8 | 0.6 | 2.4 |
| Côte d'Ivoire 1994 | 3658 | 97.5 | 1.8 | 0.7 | 2.5 |
| Gabon 2000 | 4100 | 96.9 | 1.3 | 1.8 | 3.1 |
| Côte d'Ivoire 1998 | 1703 | 96.4 | 2.1 | 1.5 | 3.6 |
| Cameroon 2011 | 10734 | 96.2 | 2.0 | 1.8 | 3.8 |
| Burkina Faso 2010 | 13716 | 96.0 | 2.2 | 1.8 | 4.0 |
| Ghana 1998 | 3015 | 95.6 | 2.0 | 2.3 | 4.4 |
| Togo 1998 | 3863 | 95.6 | 2.6 | 1.9 | 4.4 |
| Guinea 2005 | 5616 | 95.2 | 3.1 | 1.7 | 4.8 |
| Guinea 2012 | 6424 | 95.1 | 3.0 | 1.8 | 4.9 |
| Niger 1998 | 4226 | 95.0 | 2.7 | 2.3 | 5.0 |
| Congo 2005 | 4435 | 94.8 | 3.0 | 2.3 | 5.2 |
| Central African Rep 1994 | 2557 | 94.7 | 2.7 | 2.6 | 5.3 |
| Burkina Faso 1998 | 5072 | 94.5 | 3.2 | 2.3 | 5.5 |
| Ghana 2003 | 3530 | 94.5 | 2.9 | 2.5 | 5.5 |
| Mali 2001 | 10619 | 94.2 | 4.1 | 1.6 | 5.8 |
| Chad 1996 | 6338 | 93.6 | 2.6 | 3.8 | 6.4 |
| Gabon 2012 | 5747 | 93.6 | 3.0 | 3.4 | 6.4 |
| Niger 2006 | 8209 | 93.4 | 3.4 | 3.2 | 6.6 |
| Senegal 1992 | 5068 | 93.4 | 2.7 | 3.9 | 6.6 |
| Côte d'Ivoire 2011 | 7093 | 93.2 | 2.3 | 4.5 | 6.8 |
| Senegal 2005 | 10026 | 92.5 | 1.4 | 6.1 | 7.5 |
| Cameroon 1998 | 2086 | 92.4 | 2.3 | 5.3 | 7.6 |
| Guinea 1999 | 4966 | 92.4 | 4.6 | 3.1 | 7.6 |
| Ghana 1993 | 2056 | 92.3 | 2.8 | 4.9 | 7.7 |
| Mali 1995 | 5218 | 92.2 | 5.3 | 2.5 | 7.8 |
| Cameroon 1991 | 3038 | 92.0 | 1.0 | 7.0 | 8.0 |
| Chad 2004 | 4926 | 91.9 | 4.6 | 3.5 | 8.1 |
| Liberia 2007 | 5305 | 91.9 | 3.7 | 4.4 | 8.1 |
| Mali 2006 | 12437 | 91.9 | 5.3 | 2.8 | 8.1 |
| Nigeria 2003 | 5008 | 91.9 | 6.8 | 1.3 | 8.1 |
| Nigeria 1990 | 6787 | 91.5 | 3.6 | 4.9 | 8.5 |
| Burkina Faso 1993 | 5075 | 91.1 | 2.8 | 6.2 | 8.9 |
| Burkina Faso 2003 | 9352 | 90.4 | 6.8 | 2.8 | 9.6 |
| Niger 1992 | 5552 | 90.2 | 3.1 | 6.7 | 9.8 |
| Ghana 2008 | 2794 | 89.3 | 3.6 | 7.1 | 10.7 |
| Benin 2006 | 14682 | 89.0 | 6.1 | 4.9 | 11.0 |
| Niger 2012 | 11602 | 87.8 | 5.3 | 6.9 | 12.2 |
| Senegal 2010 | 11633 | 87.7 | 3.9 | 8.4 | 12.3 |
| Sierra Leone 2008 | 5043 | 85.7 | 8.7 | 5.6 | 14.3 |
| Congo DR 2007 | 7987 | 84.1 | 7.2 | 8.7 | 15.9 |
| Sao Tome et Principe 2008 | 1851 | 82.7 | 11.8 | 5.5 | 17.3 |
| Nigeria 2008 | 25446 | 79.6 | 12.7 | 7.7 | 20.4 |
| Benin 2011 | 12679 | 76.3 | 17.2 | 6.5 | 23.7 |

Supplemental Table 7. The prevalence of valid, missing, and implausible anthropometric data among children aged 059 months in 27 National Nutrition Surveys (NNS) in West Central Africa.

|  |  | The percentage of anthropometric data (height or weight) that was… | | | |
| --- | --- | --- | --- | --- | --- |
| Country, survey year | Total N of Children | Valid | Implausible | Missing | Implausible/Missing |
| Mauritania 2009 | 4378 | 100.0 | 0.0 | 0.0 | 0.0 |
| Mauritania July 2010 | 6383 | 99.8 | 0.2 | 0.0 | 0.2 |
| Mauritania July 2012 | 6817 | 99.8 | 0.1 | 0.1 | 0.2 |
| Mauritania Dec 2011 | 8173 | 99.7 | 0.2 | 0.1 | 0.3 |
| Mauritania Dec 2008 | 6342 | 99.7 | 0.1 | 0.1 | 0.3 |
| Mauritania Dec 2010 | 5851 | 99.7 | 0.2 | 0.2 | 0.3 |
| Sierra Leone 2010 | 14027 | 99.0 | 0.3 | 0.7 | 1.0 |
| Mauritania March 2008 | 6385 | 98.8 | 1.1 | 0.1 | 1.2 |
| Niger 2012 | 9226 | 98.8 | 0.3 | 0.9 | 1.2 |
| Liberia 2011 | 1484 | 98.7 | 0.8 | 0.5 | 1.3 |
| Burkina Faso 2012 | 19354 | 98.5 | 0.1 | 1.4 | 1.5 |
| Central African Rep 2012 | 15741 | 98.4 | 0.5 | 1.1 | 1.6 |
| GuineaBissau 2008 | 2718 | 98.3 | 0.7 | 1.0 | 1.7 |
| Mauritania July 2011 | 7780 | 98.3 | 0.5 | 1.2 | 1.7 |
| The Gambia 2012 | 7779 | 97.8 | 0.2 | 2.0 | 2.2 |
| Liberia 2010 | 6806 | 97.5 | 0.7 | 1.8 | 2.5 |
| Mali 2011 | 8231 | 97.1 | 0.2 | 2.7 | 2.9 |
| Togo Dec 2012 | 1451 | 96.3 | 0.1 | 3.6 | 3.7 |
| Chad (7 regions) Dec/Jan 201213 | 5799 | 95.8 | 0.3 | 3.9 | 4.2 |
| Togo June 2012 | 2780 | 95.5 | 0.2 | 4.3 | 4.5 |
| Benin 2008 | 3552 | 95.4 | 0.5 | 4.1 | 4.6 |
| Senegal 2012 | 9715 | 94.9 | 0.1 | 5.0 | 5.1 |
| Mauritania 2006 | 3881 | 94.4 | 4.1 | 1.5 | 5.6 |
| Chad June 2012 | 7777 | 93.4 | 0.3 | 6.3 | 6.6 |
| Nigeria (Northern States) 2011 | 6909 | 91.9 | 0.4 | 7.6 | 8.1 |
| Guinée Conakay 2012 | 8209 | 88.9 | 0.2 | 10.9 | 11.1 |
| Cameroon 2011 | 1481 | 88.3 | 0.7 | 11.1 | 11.7 |

Supplemental Table 8. Descriptive statistics on the terminal digit preference score for height (or length) and weight of children aged 059 months in 28 Multiple Indicator Cluster Surveys (MICS) in West Central Africa.

|  |  | Height | | |  | Weight | | | |
| --- | --- | --- | --- | --- | --- | --- | --- | --- | --- |
| Country, survey year | n | DPS | Chi-square | p-value |  | n | DPS | Chi-square | p-value |
| Burkina Faso 2006 | 5101 | 31.7 | 4613.67 | 0 |  | 5147 | 71.18 | 23467.08 | 0 |
| Cameroon 2006 | 6099 | 19.6 | 2109.29 | 0 |  | 6142 | 2.72 | 40.99 | 0 |
| Central African Rep 2000 | 13611 | 92.4 | 104596.54 | 0 |  | 13566 | 21.04 | 5407.3 | 0 |
| Central African Rep 2006 | 9256 | 57 | 27068.68 | 0 |  | 9322 | 3.71 | 115.21 | 0 |
| Central African Rep 2010 | 10283 | 6.02 | 335.86 | 0 |  | 10304 | 1.2 | 13.44 | 0.14 |
| Chad 2000 | 5266 | 12.8 | 776.79 | 0 |  | 5384 | 2.08 | 21.03 | 0.01 |
| Chad 2010 | 15361 | 20.7 | 5924.58 | 0 |  | 15600 | 3.37 | 159.33 | 0 |
| Congo DR 2001 | 9975 | 38.42 | 13253.63 | 0 |  | 10084 | 4.22 | 161.75 | 0 |
| Congo DR 2010 | 10797 | 17.47 | 2964.08 | 0 |  | 10903 | 1.87 | 34.4 | 0 |
| Côte d’Ivoire 2006 | 8558 | 43.91 | 14847.37 | 0 |  | 8588 | 4.44 | 152.14 | 0 |
| Equatorial Guinea 2000 | 2381 | 22.69 | 1103.11 | 0 |  | 2429 | 6.22 | 84.45 | 0 |
| Gambia 2000 | 3067 | 24.01 | 1591.54 | 0 |  | 3624 | 11.83 | 456.83 | 0 |
| Gambia 2005 | 6388 | 18.56 | 1979.71 | 0 |  | 6425 | 2.64 | 40.41 | 0 |
| Ghana 2006 | 3395 | 9.2 | 258.76 | 0 |  | 3404 | 1.92 | 11.28 | 0.26 |
| Ghana 2011 | 7452 | 18.12 | 2201.51 | 0 |  | 7462 | 1.31 | 11.45 | 0.25 |
| Guinea Bissau 2000 | 5670 | 67.05 | 22944.4 | 0 |  | 5736 | 10.2 | 537.41 | 0 |
| Guinea Bissau 2006 | 5539 | 70.29 | 24626.48 | 0 |  | 5655 | 18.86 | 1810.08 | 0 |
| Mauritania 2007 | 8211 | 38.56 | 10988.81 | 0 |  | 8369 | 1.21 | 10.96 | 0.28 |
| Niger 2000 | 4921 | 7.48 | 247.69 | 0 |  | 5080 | 2.7 | 33.29 | 0 |
| Nigeria 2007 | 16182 | 48.79 | 34669.68 | 0 |  | 16493 | 12.6 | 2356.04 | 0 |
| Nigeria 2011 | 24242 | 10.73 | 2509.99 | 0 |  | 24352 | 1.77 | 68.65 | 0 |
| Sao Tome et Principe 2000 | 1949 | 84.47 | 12515.51 | 0 |  | 2207 | 46.06 | 4213.15 | 0 |
| Senegal 2000 | 8621 | 18.46 | 2644.78 | 0 |  | 8688 | 7.2 | 405.05 | 0 |
| Sierra Leone 2000 | 2458 | 22.38 | 1107.86 | 0 |  | 2462 | 4.1 | 37.16 | 0 |
| Sierra Leone 2005 | 5183 | 25.22 | 2966.43 | 0 |  | 5207 | 3.17 | 46.96 | 0 |
| Sierra Leone 2010 | 8335 | 23.88 | 4276.14 | 0 |  | 8385 | 4.38 | 145.08 | 0 |
| Togo 2006 | 3987 | 32 | 3674.27 | 0 |  | 4004 | 21.98 | 1740.78 | 0 |
| Togo 2010 | 4691 | 4.24 | 75.83 | 0 |  | 4695 | 1.34 | 7.57 | 0.58 |

Supplemental Table 9. Descriptive statistics on the terminal digit preference score for height (or length) and weight of children aged 059 months in 28 Demographic and Health Surveys (DHS) in West Central Africa.

|  |  | Height | | |  | Weight | | | |
| --- | --- | --- | --- | --- | --- | --- | --- | --- | --- |
| Country, survey year | N | DPS | Chi-square | p-value |  | n | DPS | Chi-square | p-value |
| Benin 2001 | 4524 | 22.96 | 2143.45 | 0 |  | 4488 | 2.3 | 21.39 | 0.01 |
| Benin 2006 | 13443 | 35.54 | 15245.24 | 0 |  | 13344 | 6.91 | 573.07 | 0 |
| Benin 2011 | 11441 | 57.2 | 33250.66 | 0 |  | 11038 | 21.18 | 4457.51 | 0 |
| Burkina Faso 1993 | 4590 | 16.14 | 1071.76 | 0 |  | 4679 | 3.73 | 58.71 | 0 |
| Burkina Faso 1998 | 4782 | 14.89 | 949.18 | 0 |  | 4768 | 1.34 | 7.76 | 0.56 |
| Burkina Faso 2003 | 8800 | 27.13 | 5812.69 | 0 |  | 8752 | 20.43 | 3288.64 | 0 |
| Burkina Faso 2010 | 6730 | 12.67 | 969.46 | 0 |  | 6673 | 2.74 | 45.19 | 0 |
| Cameroon 1991 | 2687 | 10.23 | 252.33 | 0 |  | 2653 | 3.84 | 35.13 | 0 |
| Cameroon 1998 | 1876 | 22.66 | 863.47 | 0 |  | 1926 | 2.87 | 14.32 | 0.11 |
| Cameroon 2011 | 5190 | 6.72 | 210.37 | 0 |  | 5149 | 1.48 | 10.17 | 0.34 |
| Central African Rep 1994 | 2453 | 20.11 | 885.48 | 0 |  | 2429 | 3.02 | 19.89 | 0.02 |
| Chad 1996 | 5856 | 22.69 | 2705.58 | 0 |  | 5896 | 1.13 | 6.72 | 0.67 |
| Chad 2004 | 4656 | 24.7 | 2539.97 | 0 |  | 4635 | 2.08 | 17.99 | 0.04 |
| Congo 2005 | 4063 | 43.55 | 6889.53 | 0 |  | 4055 | 2.43 | 21.55 | 0.01 |
| Congo 2012 | 4531 | 8.93 | 325 | 0 |  | 4504 | 1.39 | 7.78 | 0.56 |
| Congo DR 2007 | 3671 | 36.07 | 4251.11 | 0 |  | 3665 | 3.49 | 40.21 | 0 |
| Côte d'Ivoire 1994 | 3521 | 20.77 | 1361.19 | 0 |  | 3464 | 0.67 | 1.39 | 1 |
| Côte d'Ivoire 1998 | 1598 | 26.18 | 980.41 | 0 |  | 1590 | 4 | 22.89 | 0.01 |
| Côte d'Ivoire 2011 | 3301 | 13.38 | 530.29 | 0 |  | 3280 | 2.32 | 15.85 | 0.07 |
| Gabon 2000 | 3572 | 14.17 | 642.96 | 0 |  | 3532 | 1.26 | 5.08 | 0.83 |
| Gabon 2012 | 3491 | 11.69 | 426.76 | 0 |  | 3449 | 1.93 | 11.54 | 0.24 |
| Ghana 1993 | 1967 | 12.98 | 297.72 | 0 |  | 1968 | 4.04 | 28.85 | 0 |
| Ghana 1998 | 2859 | 13.14 | 439.59 | 0 |  | 2843 | 1.65 | 6.96 | 0.64 |
| Ghana 2003 | 3201 | 10.61 | 323.94 | 0 |  | 3247 | 3 | 26.38 | 0 |
| Ghana 2008 | 2539 | 12.34 | 343.62 | 0 |  | 2530 | 2.79 | 17.74 | 0.04 |
| Guinea 1999 | 4679 | 14.03 | 813.64 | 0 |  | 4608 | 1.23 | 6.27 | 0.71 |
| Guinea 2005 | 2754 | 20.92 | 1079.08 | 0 |  | 2738 | 2.57 | 16.22 | 0.06 |
| Guinea 2012 | 3221 | 10.49 | 316.21 | 0 |  | 3183 | 2.12 | 12.82 | 0.17 |
| Liberia 2007 | 4594 | 10.17 | 423.29 | 0 |  | 4541 | 2.36 | 22.78 | 0.01 |
| Mali 1995 | 5009 | 18.28 | 1504.33 | 0 |  | 4951 | 3.96 | 70.01 | 0 |
| Mali 2001 | 9903 | 12.98 | 1492.17 | 0 |  | 9775 | 1.53 | 20.47 | 0.02 |
| Mali 2006 | 11650 | 24.52 | 6263.98 | 0 |  | 11441 | 3.96 | 161.43 | 0 |
| Niger 1992 | 4891 | 9.77 | 419.09 | 0 |  | 4986 | 2.72 | 33.23 | 0 |
| Niger 1998 | 4035 | 10.31 | 385.82 | 0 |  | 4026 | 2.13 | 16.49 | 0.06 |
| Niger 2006 | 3870 | 27.57 | 2642.79 | 0 |  | 3858 | 2.38 | 19.65 | 0.02 |
| Niger 2012 | 5154 | 29.13 | 3922.63 | 0 |  | 5127 | 7.66 | 271.02 | 0 |
| Nigeria 1990 | 6154 | 12 | 796.26 | 0 |  | 6110 | 7.05 | 273.13 | 0 |
| Nigeria 2003 | 4803 | 22.02 | 2080.6 | 0 |  | 4737 | 3.09 | 40.73 | 0 |
| Nigeria 2008 | 23259 | 27.25 | 15245.1 | 0 |  | 22972 | 6.08 | 763.27 | 0 |
| Sao Tome et Principe 2008 | 1712 | 91.76 | 12875.91 | 0 |  | 1632 | 80.31 | 9473.43 | 0 |
| Senegal 1992 | 4694 | 8.78 | 322.89 | 0 |  | 4678 | 1.64 | 11.33 | 0.25 |
| Senegal 2005 | 2940 | 10.81 | 308.18 | 0 |  | 2932 | 2.09 | 11.56 | 0.24 |
| Senegal 2010 | 3929 | 22.94 | 1856.55 | 0 |  | 3905 | 2.5 | 22.01 | 0.01 |
| Sierra Leone 2008 | 2285 | 16.86 | 580.42 | 0 |  | 2229 | 3.58 | 25.67 | 0 |
| Togo 1998 | 3788 | 10.46 | 371.49 | 0 |  | 3782 | 2.15 | 15.66 | 0.07 |

Supplemental Table 10. Descriptive statistics on the terminal digit preference score for height (or length) and weight of children aged 059 months in 28 National Nutrition Surveys (NNS) in West Central Africa.

|  |  | Height | | |  | Weight | | | |
| --- | --- | --- | --- | --- | --- | --- | --- | --- | --- |
| Country, survey year | n | DPS | Chi-square | p-value |  | n | DPS | Chi-square | p-value |
| Benin 2008 | 3423 | 8.22 | 208.37 | 0 |  | 3437 | 1.85 | 10.58 | 0.31 |
| Burkina Faso 2012 | 19075 | 1.96 | 66.25 | 0 |  | 19099 | 0.68 | 8.04 | 0.53 |
| Cameroon 2011 | 1317 | 8.39 | 83.4 | 0 |  | 1317 | 2.74 | 8.91 | 0.45 |
| Central African Rep 2012 | 15582 | 5.22 | 382.6 | 0 |  | 15653 | 0.85 | 10.28 | 0.33 |
| Chad June 2012 | 7303 | 3.43 | 77.15 | 0 |  | 7338 | 0.78 | 4.03 | 0.91 |
| Chad (7 regions) Dec/Jan 2012-13 | 5583 | 4.54 | 103.52 | 0 |  | 5671 | 1.03 | 5.4 | 0.8 |
| The Gambia 2012 | 7626 | 4.32 | 127.92 | 0 |  | 7636 | 1.27 | 11.13 | 0.27 |
| Guinea-Bissau 2008 | 2716 | 5.83 | 83.08 | 0 |  | 2717 | 2.58 | 16.33 | 0.06 |
| Guinée Conakay 2012 | 8162 | 3.93 | 113.25 | 0 |  | 7510 | 3.82 | 98.79 | 0 |
| Liberia 2010 | 6796 | 4.93 | 148.38 | 0 |  | 6802 | 2.46 | 37.01 | 0 |
| Liberia 2011 | 1479 | 10.26 | 140.25 | 0 |  | 1478 | 2.8 | 10.44 | 0.32 |
| Mali 2011 | 8011 | 4.58 | 151.33 | 0 |  | 8032 | 1.84 | 24.49 | 0 |
| Mauritania 2006 | 3843 | 18.12 | 1136 | 0 |  | 3881 | 3.23 | 36.48 | 0 |
| Mauritania March 2008 | 6371 | 19.69 | 2224.07 | 0 |  | 6385 | 1.84 | 19.55 | 0.02 |
| Mauritania Dec 2008 | 6335 | 4.33 | 107.1 | 0 |  | 6341 | 1.86 | 19.77 | 0.02 |
| Mauritania 2009 | 4378 | 4.92 | 95.26 | 0 |  | 4378 | 1.87 | 13.83 | 0.13 |
| Mauritania July 2010 | 6383 | 5.07 | 147.49 | 0 |  | 6383 | 1.96 | 22.06 | 0.01 |
| Mauritania Dec 2010 | 5850 | 5.07 | 135.23 | 0 |  | 5851 | 1.39 | 10.17 | 0.34 |
| Mauritania July 2011 | 7773 | 4.84 | 163.66 | 0 |  | 7778 | 1.22 | 10.49 | 0.31 |
| Mauritania Dec 2011 | 8172 | 5.02 | 185.1 | 0 |  | 8173 | 1.03 | 7.85 | 0.55 |
| Mauritania July 2012 | 6816 | 5.68 | 197.92 | 0 |  | 6817 | 0.92 | 5.15 | 0.82 |
| Niger 2012 | 9152 | 3.97 | 129.79 | 0 |  | 9168 | 2.04 | 34.49 | 0 |
| Nigeria (Northern States) 2011 | 6603 | 4.38 | 114.21 | 0 |  | 6627 | 1.08 | 6.94 | 0.64 |
| Senegal 2012 | 9216 | 3.24 | 86.87 | 0 |  | 9229 | 1.01 | 8.44 | 0.49 |
| Sierra Leone 2010 | 13950 | 2.51 | 78.79 | 0 |  | 13955 | 0.56 | 3.9 | 0.92 |
| Togo June 2012 | 2663 | 2.43 | 14.17 | 0.12 |  | 2669 | 1.52 | 5.55 | 0.78 |
| Togo Dec 2012 | 1400 | 4.3 | 23.29 | 0.01 |  | 1400 | 2.56 | 8.27 | 0.51 |

Supplemental Table 11. Mean and standard deviation (SD) for height-for-age (HAZ), weight-for-age (WTZ), and weight-for-height (WHZ), as well as skewness and kurtosis, based on the WHO 2006 reference standard among children aged 0-59 months in 28 MICS.

|  | HAZ | | | |  | WAZ | | | |  | WHZ | | | |
| --- | --- | --- | --- | --- | --- | --- | --- | --- | --- | --- | --- | --- | --- | --- |
| Country, survey year | Mean | SD | Skewness | Kurtosis |  | Mean | SD | Skewness | Kurtosis |  | Mean | SD | Skewness | Kurtosis |
| Burkina Faso 2006 | 1.629 | 1.891 | 0.417 | 3.484 |  | 1.568 | 1.665 | 0.004 | 3.001 |  | 0.692 | 1.888 | 0.039 | 2.749 |
| Cameroon 2006 | 1.383 | 1.768 | 0.45 | 3.882 |  | 0.644 | 1.421 | 0.138 | 3.551 |  | 0.214 | 1.485 | 0.121 | 3.597 |
| Central African Rep 2000 | 1.636 | 1.977 | 0.576 | 3.812 |  | 0.969 | 1.419 | 0.037 | 3.764 |  | 0.057 | 1.661 | 0.148 | 3.488 |
| Central African Rep 2006 | 1.57 | 2.081 | 0.514 | 3.523 |  | 1.058 | 1.569 | 0.125 | 3.625 |  | 0.133 | 1.636 | 0.007 | 3.561 |
| Central African Rep 2010 | 1.589 | 1.557 | 0.296 | 3.522 |  | 1.152 | 1.242 | 0.165 | 3.526 |  | 0.311 | 1.135 | 0.142 | 3.61 |
| Chad 2000 | 1.448 | 1.787 | 0.246 | 3.372 |  | 1.247 | 1.454 | 0.092 | 3.438 |  | 0.572 | 1.359 | 0.073 | 3.656 |
| Chad 2010 | 1.406 | 2.088 | 0.502 | 3.456 |  | 1.417 | 1.513 | 0.079 | 3.376 |  | 0.732 | 1.44 | 0.162 | 3.387 |
| Congo DR 2001 | 1.554 | 2.071 | 0.502 | 3.495 |  | 1.297 | 1.462 | 0.087 | 3.535 |  | 0.431 | 1.638 | 0.041 | 3.315 |
| Congo DR 2010 | 1.613 | 1.872 | 0.596 | 4.051 |  | 1.086 | 1.397 | 0.154 | 3.859 |  | 0.218 | 1.365 | 0.021 | 4.01 |
| Côte d’Ivoire 2006 | 1.555 | 1.833 | 0.452 | 3.916 |  | 0.875 | 1.32 | 0.033 | 4.274 |  | 0.043 | 1.526 | 0.077 | 3.583 |
| Equatorial Guinea 2000 | 1.564 | 2.082 | 0.514 | 3.664 |  | 0.626 | 1.515 | 0.092 | 4.02 |  | 0.39 | 1.681 | 0.276 | 3.546 |
| Gambia 2000 | 1.036 | 1.775 | 0.477 | 4.616 |  | 0.872 | 1.317 | 0.152 | 4.036 |  | 0.333 | 1.342 | 0.043 | 3.756 |
| Gambia 2005 | 1.176 | 1.552 | 0.184 | 3.92 |  | 0.892 | 1.204 | 0.046 | 3.918 |  | 0.309 | 1.221 | 0.021 | 3.96 |
| Ghana 2006 | 1.26 | 1.522 | 0.292 | 4.088 |  | 0.912 | 1.181 | 0.177 | 4.41 |  | 0.261 | 1.198 | 0.2 | 4.125 |
| Ghana 2011 | 1.26 | 1.367 | 0.15 | 4.165 |  | 1 | 1.123 | 0.079 | 4.161 |  | 0.401 | 1.147 | 0.036 | 4.209 |
| Guinea Bissau 2000 | 1.346 | 1.794 | 0.389 | 3.996 |  | 1.036 | 1.407 | 0.037 | 4.083 |  | 0.335 | 1.467 | 0.079 | 3.615 |
| Guinea Bissau 2006 | 1.8 | 2.054 | 0.632 | 4.046 |  | 0.881 | 1.366 | 0.151 | 4.341 |  | 0.365 | 1.818 | 0.12 | 3.377 |
| Mauritania 2007 | 1.074 | 1.76 | 0.362 | 3.613 |  | 1.132 | 1.318 | 0.034 | 3.589 |  | 0.703 | 1.309 | 0.14 | 4.141 |
| Niger 2000 | 1.911 | 1.619 | 0.254 | 3.557 |  | 1.7 | 1.33 | 0.182 | 3.443 |  | 0.796 | 1.292 | 0.111 | 3.974 |
| Nigeria 2007 | 1.459 | 2.369 | 0.495 | 3.173 |  | 0.961 | 1.876 | 0.134 | 3.349 |  | 0.092 | 1.84 | 0.118 | 3.155 |
| Nigeria 2011 | 1.494 | 1.881 | 0.275 | 3.451 |  | 1.16 | 1.405 | 0.007 | 3.644 |  | 0.403 | 1.31 | 0.116 | 3.843 |
| Sao Tome et Principe 2000 | 1.442 | 1.611 | 0.285 | 4.258 |  | 0.624 | 1.21 | 0.107 | 4.73 |  | 0.315 | 1.356 | 0.052 | 4.073 |
| Senegal 2000 | 1.201 | 1.689 | 0.279 | 3.877 |  | 1.014 | 1.347 | 0.067 | 3.722 |  | 0.434 | 1.275 | 0.034 | 4.003 |
| Sierra Leone 2000 | 1.296 | 2.099 | 0.482 | 3.471 |  | 1.051 | 1.54 | 0.059 | 3.662 |  | 0.342 | 1.468 | 0.065 | 3.939 |
| Sierra Leone 2005 | 1.759 | 1.973 | 0.504 | 3.558 |  | 1.231 | 1.499 | 0.01 | 3.748 |  | 0.178 | 1.492 | 0.14 | 3.79 |
| Sierra Leone 2010 | 1.731 | 1.881 | 0.434 | 3.726 |  | 0.99 | 1.423 | 0.057 | 3.961 |  | 0.119 | 1.576 | 0.072 | 3.643 |
| Togo 2006 | 1.341 | 1.783 | 0.219 | 3.655 |  | 1.284 | 1.481 | 0.074 | 3.52 |  | 0.654 | 1.595 | 0.072 | 3.369 |
| Togo 2010 | 1.457 | 1.335 | 0.237 | 4.357 |  | 1.082 | 1.138 | 0.18 | 3.969 |  | 0.346 | 1.046 | 0.144 | 3.619 |

Supplemental Table 12. Mean and standard deviation (SD) for height-for-age (HAZ), weight-for-age (WTZ), and weight-for-height (WHZ), as well as skewness and kurtosis, based on the WHO 2006 reference standard among children aged 0-59 months in 45 DHS.

|  | HAZ | | | |  | WAZ | | | |  | WHZ | | | |
| --- | --- | --- | --- | --- | --- | --- | --- | --- | --- | --- | --- | --- | --- | --- |
| Country, survey year | Mean | SD | Skewness | Kurtosis |  | Mean | SD | Skewness | Kurtosis |  | Mean | SD | Skewness | Kurtosis |
| Benin 2001 | -1.51 | 1.61 | 0.43 | 4.01 |  | -1.11 | 1.23 | -0.14 | 3.88 |  | -0.30 | 1.21 | -0.25 | 4.19 |
| Benin 2006 | -1.71 | 1.87 | 0.37 | 3.80 |  | -1.01 | 1.39 | 0.09 | 4.26 |  | 0.08 | 1.47 | 0.13 | 3.51 |
| Benin 2011 | -1.77 | 1.72 | 0.52 | 2.99 |  | -0.83 | 1.39 | 0.26 | 3.50 |  | 0.12 | 1.34 | -0.01 | 2.54 |
| Burkina Faso 1993 | -1.40 | 1.88 | 0.27 | 3.41 |  | -1.23 | 1.23 | -0.06 | 3.75 |  | -0.59 | 1.49 | -0.08 | 3.83 |
| Burkina Faso 1998 | -1.70 | 1.64 | 0.39 | 3.65 |  | -1.50 | 1.26 | -0.17 | 3.28 |  | -0.67 | 1.43 | -0.18 | 3.61 |
| Burkina Faso 2003 | -1.59 | 1.81 | 0.43 | 3.48 |  | -1.57 | 1.40 | 0.02 | 3.50 |  | -0.77 | 1.35 | 0.09 | 3.28 |
| Burkina Faso 2010 | -1.37 | 1.91 | 0.36 | 3.99 |  | -1.26 | 1.44 | -0.05 | 3.91 |  | -0.64 | 1.42 | -0.05 | 3.77 |
| Cameroon 1991 | -1.40 | 2.22 | 0.00 | 3.72 |  | -0.70 | 1.71 | -0.33 | 3.59 |  | 0.17 | 1.86 | -0.29 | 3.72 |
| Cameroon 1998 | -1.29 | 1.64 | 0.34 | 3.68 |  | -0.66 | 1.36 | -0.02 | 3.26 |  | 0.04 | 1.34 | -0.09 | 3.50 |
| Cameroon 2011 | -1.24 | 1.51 | 0.43 | 4.14 |  | -0.62 | 1.20 | -0.21 | 3.55 |  | 0.14 | 1.23 | -0.21 | 3.64 |
| Central African Rep 1994 | -1.53 | 1.60 | 0.26 | 3.59 |  | -1.10 | 1.25 | -0.21 | 3.39 |  | -0.31 | 1.29 | -0.05 | 3.86 |
| Chad 1996 | -1.61 | 1.61 | 0.40 | 3.40 |  | -1.43 | 1.36 | 0.03 | 3.34 |  | -0.68 | 1.21 | -0.07 | 3.57 |
| Chad 2004 | -1.54 | 1.79 | 0.42 | 3.28 |  | -1.41 | 1.44 | 0.04 | 3.45 |  | -0.67 | 1.48 | 0.05 | 3.55 |
| Congo 2005 | -1.05 | 2.20 | 0.39 | 3.79 |  | -0.57 | 1.70 | -0.11 | 4.22 |  | 0.04 | 1.76 | -0.16 | 3.77 |
| Congo 2012 | -1.14 | 1.84 | 0.29 | 4.15 |  | -0.79 | 1.39 | 0.03 | 3.83 |  | -0.19 | 1.40 | 0.03 | 4.27 |
| Congo DR 2007 | -1.60 | 1.59 | 0.61 | 3.77 |  | -1.06 | 1.28 | 0.17 | 3.93 |  | -0.15 | 1.39 | 0.14 | 3.80 |
| Côte d'Ivoire 1994 | -1.27 | 1.88 | 0.22 | 3.64 |  | -1.01 | 1.46 | -0.24 | 3.71 |  | -0.41 | 1.42 | -0.18 | 3.47 |
| Côte d'Ivoire 1998 | -1.21 | 1.77 | 0.13 | 3.98 |  | -0.79 | 1.38 | -0.14 | 3.88 |  | -0.11 | 1.49 | 0.00 | 4.01 |
| Côte d'Ivoire 2011 | -1.25 | 1.88 | 0.36 | 4.19 |  | -0.88 | 1.45 | -0.18 | 4.34 |  | -0.19 | 1.37 | -0.05 | 4.05 |
| Gabon 2000 | -1.19 | 1.66 | 0.50 | 4.78 |  | -0.59 | 1.29 | 0.05 | 4.54 |  | 0.12 | 1.29 | -0.29 | 4.28 |
| Gabon 2012 | -1.00 | 1.68 | 0.34 | 4.71 |  | -0.42 | 1.34 | 0.05 | 4.57 |  | 0.16 | 1.37 | -0.08 | 4.56 |
| Ghana 1993 | -1.30 | 1.60 | 0.25 | 3.88 |  | -1.16 | 1.35 | -0.21 | 3.67 |  | -0.56 | 1.30 | -0.15 | 3.57 |
| Ghana 1998 | -1.36 | 1.58 | 0.28 | 3.67 |  | -1.12 | 1.25 | 0.08 | 4.06 |  | -0.47 | 1.35 | 0.09 | 4.54 |
| Ghana 2003 | -1.44 | 1.89 | 0.36 | 3.94 |  | -1.00 | 1.38 | -0.01 | 4.34 |  | -0.23 | 1.41 | -0.08 | 4.04 |
| Ghana 2008 | -1.07 | 1.85 | 0.39 | 4.04 |  | -0.78 | 1.45 | 0.41 | 4.63 |  | -0.30 | 1.34 | 0.11 | 4.21 |
| Guinea 1999 | -1.22 | 1.86 | 0.48 | 3.81 |  | -0.95 | 1.54 | 0.04 | 3.98 |  | -0.29 | 1.56 | -0.11 | 4.01 |
| Guinea 2005 | -1.42 | 1.71 | 0.54 | 3.93 |  | -1.02 | 1.32 | 0.12 | 3.88 |  | -0.26 | 1.42 | -0.01 | 4.04 |
| Guinea 2012 | -1.06 | 2.01 | 0.43 | 3.68 |  | -0.87 | 1.52 | 0.15 | 4.00 |  | -0.36 | 1.58 | -0.22 | 4.04 |
| Liberia 2007 | -1.48 | 1.83 | 0.46 | 3.83 |  | -0.95 | 1.35 | -0.10 | 3.88 |  | -0.12 | 1.34 | -0.28 | 4.17 |
| Mali 1995 | -1.36 | 1.86 | 0.19 | 3.19 |  | -1.67 | 1.31 | -0.11 | 3.10 |  | -1.14 | 1.62 | 0.06 | 3.18 |
| Mali 2001 | -1.60 | 1.71 | 0.40 | 3.62 |  | -1.34 | 1.33 | -0.01 | 3.55 |  | -0.53 | 1.36 | -0.06 | 3.85 |
| Mali 2006 | -1.40 | 2.10 | 0.42 | 3.52 |  | -1.24 | 1.55 | 0.13 | 3.73 |  | -0.58 | 1.55 | 0.14 | 3.80 |
| Niger 1992 | -1.70 | 1.69 | 0.40 | 3.73 |  | -1.59 | 1.39 | 0.09 | 3.82 |  | -0.83 | 1.40 | 0.06 | 3.92 |
| Niger 1998 | -1.76 | 1.65 | 0.36 | 3.62 |  | -1.82 | 1.25 | 0.06 | 3.43 |  | -1.11 | 1.33 | 0.07 | 3.47 |
| Niger 2006 | -1.92 | 1.95 | 0.45 | 3.60 |  | -1.53 | 1.54 | 0.06 | 3.48 |  | -0.53 | 1.72 | 0.14 | 4.26 |
| Niger 2012 | -1.63 | 2.47 | 0.42 | 3.99 |  | -1.61 | 1.85 | 0.08 | 3.63 |  | -0.82 | 2.15 | 0.17 | 4.04 |
| Nigeria 1990 | -1.83 | 1.71 | 0.39 | 3.61 |  | -1.38 | 1.34 | -0.09 | 3.53 |  | -0.37 | 1.33 | -0.23 | 4.08 |
| Nigeria 2003 | -1.60 | 1.51 | 0.42 | 3.63 |  | -1.19 | 1.13 | -0.02 | 3.75 |  | -0.27 | 1.19 | -0.04 | 3.80 |
| Nigeria 2008 | -1.49 | 1.57 | 0.51 | 3.39 |  | -1.12 | 1.21 | 0.03 | 3.48 |  | -0.15 | 1.24 | -0.10 | 3.24 |
| Sao Tome et Principe 2008 | -1.04 | 1.92 | 0.64 | 4.37 |  | -0.67 | 1.41 | -0.02 | 4.02 |  | -0.08 | 1.80 | 0.02 | 3.24 |
| Senegal 1992 | -1.38 | 1.64 | 0.11 | 3.49 |  | -1.06 | 1.24 | -0.11 | 3.80 |  | -0.34 | 1.27 | -0.05 | 4.06 |
| Senegal 2005 | -0.91 | 1.80 | 0.19 | 4.24 |  | -0.85 | 1.40 | -0.10 | 3.86 |  | -0.46 | 1.37 | 0.04 | 4.04 |
| Senegal 2010 | -1.25 | 1.66 | 0.30 | 4.24 |  | -1.08 | 1.44 | 0.13 | 4.45 |  | -0.51 | 1.42 | 0.31 | 4.64 |
| Sierra Leone 2008 | -1.25 | 1.97 | 0.48 | 3.41 |  | -0.71 | 1.50 | 0.23 | 3.52 |  | -0.07 | 1.53 | 0.24 | 3.58 |
| Togo 1998 | -1.24 | 2.08 | 0.33 | 3.88 |  | -1.17 | 1.52 | -0.06 | 3.83 |  | -0.64 | 1.62 | 0.07 | 3.58 |

Supplemental Table 13. Mean and standard deviation (SD) for height-for-age (HAZ), weight-for-age (WTZ), and weight-for-height (WHZ), as well as skewness and kurtosis, based on the WHO 2006 reference standard among children aged 0-59 months in 27 NNS.

|  | HAZ | | | |  | WAZ | | | |  | WHZ | | | |
| --- | --- | --- | --- | --- | --- | --- | --- | --- | --- | --- | --- | --- | --- | --- |
| Country, survey year | Mean | SD | Skewness | Kurtosis |  | Mean | SD | Skewness | Kurtosis |  | Mean | SD | Skewness | Kurtosis |
| Benin 2008 | -1.54 | 1.36 | 0.19 | 4.18 |  | -1.03 | 1.10 | -0.20 | 4.00 |  | -0.22 | 1.09 | -0.11 | 4.09 |
| Burkina Faso 2012 | -1.51 | 1.21 | 0.09 | 3.52 |  | -1.33 | 1.06 | -0.17 | 3.31 |  | -0.68 | 1.07 | -0.09 | 3.22 |
| Cameroon 2011 | -1.73 | 1.53 | 0.36 | 3.39 |  | -1.37 | 1.25 | -0.06 | 3.28 |  | -0.56 | 1.20 | -0.06 | 3.85 |
| Central African Rep 2012 | -1.59 | 1.48 | 0.27 | 3.99 |  | -1.21 | 1.23 | -0.19 | 3.44 |  | -0.39 | 1.15 | -0.03 | 3.84 |
| The Gambia 2012 | -1.18 | 1.21 | 0.10 | 3.96 |  | -1.15 | 1.08 | -0.07 | 3.55 |  | -0.70 | 1.08 | 0.02 | 3.92 |
| Mali 2011 | -1.10 | 1.42 | 0.06 | 3.12 |  | -1.10 | 1.12 | -0.17 | 3.31 |  | -0.69 | 1.10 | -0.06 | 3.45 |
| Sierra Leone 2010 | -1.44 | 1.31 | 0.44 | 4.57 |  | -1.09 | 1.11 | -0.16 | 3.78 |  | -0.36 | 1.12 | -0.23 | 3.51 |
| Senegal 2012 | -1.08 | 1.30 | 0.16 | 4.05 |  | -1.18 | 1.08 | -0.11 | 3.46 |  | -0.83 | 1.03 | -0.07 | 3.34 |
| Guinea-Bissau 2008 | -1.31 | 1.35 | 0.38 | 4.86 |  | -0.95 | 1.19 | 0.07 | 4.33 |  | -0.31 | 1.07 | 0.03 | 4.06 |
| Guinée Conakay 2012 | -1.52 | 1.35 | 0.20 | 4.12 |  | -0.97 | 1.14 | -0.19 | 4.02 |  | -0.13 | 1.16 | 0.02 | 4.14 |
| Liberia 2010 | -1.67 | 1.46 | 0.56 | 4.65 |  | -0.93 | 1.16 | -0.15 | 3.76 |  | 0.01 | 1.12 | -0.21 | 4.30 |
| Liberia 2011 | -1.50 | 1.44 | 0.50 | 5.06 |  | -1.17 | 1.29 | 0.02 | 3.98 |  | -0.50 | 1.24 | -0.10 | 3.46 |
| Chad June 2012 | -1.39 | 1.44 | 0.11 | 3.61 |  | -1.54 | 1.14 | -0.11 | 3.55 |  | -1.08 | 1.08 | 0.12 | 3.75 |
| Chad (7 regions) Dec/Jan 2012-13 | -1.41 | 1.43 | 0.04 | 3.47 |  | -0.82 | 1.19 | -0.36 | 3.86 |  | -0.03 | 1.14 | -0.36 | 4.06 |
| Mauritania 2006 | -1.33 | 1.71 | 0.60 | 4.84 |  | -1.09 | 1.40 | 0.19 | 4.87 |  | -0.48 | 1.17 | -0.12 | 4.16 |
| Mauritania March 2008 | -1.29 | 1.63 | 0.41 | 4.02 |  | -1.16 | 1.28 | -0.03 | 3.48 |  | -0.61 | 1.26 | 0.08 | 3.73 |
| Mauritania Dec 2008 | -1.05 | 1.25 | 0.03 | 3.03 |  | -0.92 | 1.04 | -0.16 | 3.18 |  | -0.47 | 1.07 | -0.08 | 3.19 |
| Mauritania 2009 | -1.06 | 1.27 | 0.09 | 2.82 |  | -1.12 | 1.01 | -0.09 | 2.98 |  | -0.75 | 1.11 | -0.08 | 3.26 |
| Mauritania July 2010 | -1.17 | 1.31 | 0.28 | 4.30 |  | -1.21 | 1.03 | 0.01 | 3.65 |  | -0.82 | 1.05 | 0.12 | 3.54 |
| Mauritania Dec 2010 | -1.07 | 1.30 | 0.26 | 4.39 |  | -0.95 | 1.04 | -0.06 | 3.69 |  | -0.53 | 1.03 | 0.03 | 3.41 |
| Mauritania July 2011 | -1.09 | 1.26 | 0.22 | 4.06 |  | -1.13 | 1.05 | 0.02 | 3.63 |  | -0.75 | 1.06 | 0.12 | 3.44 |
| Mauritania Dec 2011 | -1.25 | 1.24 | 0.06 | 3.40 |  | -1.05 | 1.03 | -0.18 | 3.52 |  | -0.51 | 1.03 | -0.04 | 3.53 |
| Mauritania July 2012 | -1.03 | 1.36 | 0.30 | 3.73 |  | -1.14 | 1.13 | 0.16 | 3.25 |  | -0.81 | 1.10 | 0.10 | 3.26 |
| Niger 2012 | -1.56 | 1.41 | 0.22 | 3.78 |  | -1.46 | 1.18 | -0.08 | 3.47 |  | -0.81 | 1.10 | 0.08 | 3.89 |
| Nigeria (Northern States) 2011 | -1.75 | 1.38 | 0.17 | 3.79 |  | -1.43 | 1.12 | -0.40 | 4.06 |  | -0.60 | 1.19 | -0.17 | 3.80 |
| Togo June 2012 | -1.31 | 1.24 | 0.17 | 3.80 |  | -1.00 | 1.11 | -0.10 | 3.49 |  | -0.36 | 1.06 | 0.06 | 3.77 |
| Togo Dec 2012 | -1.53 | 1.19 | 0.05 | 3.47 |  | -1.27 | 1.02 | -0.30 | 3.41 |  | -0.58 | 1.03 | -0.17 | 3.76 |

Supplemental Table 14. Overall child anthropometric data quality scores as calculated using the SMART methodology* among 28 MICS in West Central Africa countries

| Country, survey year | Overall Anthropometric Data Quality | | |
| --- | --- | --- | --- |
|  | Total score at the country level | Minimum score at the regional level | Maximum score at the regional level |
| Burkina Faso 2006 | 55 | 49 | 69 |
| Cameroon 2006 | 26 | 21 | 56 |
| Central African Rep 2000 | 61 | 32 | 69 |
| Central African Rep 2006 | 56 | 34 | 61 |
| Central African Rep 2010 | 18 | 9 | 49 |
| Chad 2000 | 24 | 16 | 28 |
| Chad 2010 | 48 | 37 | 54 |
| Congo DR 2001 | 36 | 32 | 61 |
| Congo DR 2010 | 25 | 21 | 38 |
| Côte d’Ivoire 2006 | 30 | 22 | 51 |
| Equatorial Guinea 2000 | 61 | 43 | 64 |
| Gambia 2000 | 49 | 30 | 54 |
| Gambia 2005 | 26 | 11 | 36 |
| Ghana 2006 | 30 | 19 | 44 |
| Ghana 2011 | 26 | 21 | 35 |
| Guinea Bissau 2000 | 43 | 27 | 59 |
| Guinea Bissau 2006 | 55 | 49 | 67 |
| Mauritania 2007 | 42 | 25 | 53 |
| Niger 2000 | 34 | 25 | 44 |
| Nigeria 2007 | 58 | 34 | 72 |
| Nigeria 2011 | 40 | 16 | 52 |
| Sao Tome et Principe 2000 | 59 | 53 | 62 |
| Senegal 2000 | 33 | 23 | 47 |
| Sierra Leone 2000 | 51 | 44 | 47 |
| Sierra Leone 2005 | 50 | 39 | 52 |
| Sierra Leone 2010 | 49 | 31 | 60 |
| Togo 2006 | 39 | 26 | 59 |
| Togo 2010 | 21 | 14 | 26 |

* Note: The data quality score represents a weighted combination of the level of missing and/or implausible data, overall sex ratio, overall age ratio, digit preference score for weight, digit preference score for height, standard deviation of WHZ, skewness of WHS, and kurtosis of WHZ.

Supplemental Table 15. Overall child anthropometric data quality scores as calculated using the SMART methodology* among 45 DHS in West Central Africa countries

| Country, survey year | Overall Anthropometric Data Quality | | |
| --- | --- | --- | --- |
|  | Total score at the country level | Minimum score at the regional level | Maximum score at the regional level |
| Benin 2001 | 24 | 18 | 35 |
| Benin 2006 | 49 | 27 | 63 |
| Benin 2011 | 70 | 47 | 73 |
| Burkina Faso 1993 | 33 | 30 | 52 |
| Burkina Faso 1998 | 30 | 27 | 35 |
| Burkina Faso 2003 | 59 | 27 | 67 |
| Burkina Faso 2010 | 22 | 13 | 39 |
| Cameroon 1991 | 32 | 18 | 40 |
| Cameroon 1998 | 32 | 30 | 42 |
| Cameroon 2011 | 17 | 8 | 28 |
| Central African Rep 1994 | 30 | 22 | 37 |
| Chad 1996 | 37 | 28 | 51 |
| Chad 2004 | 37 | 24 | 50 |
| Congo 2005 | 34 | 29 | 37 |
| Congo 2012 | 11 | 9 | 33 |
| Congo DR 2007 | 49 | 39 | 63 |
| Côte d'Ivoire 1994 | 19 | 14 | 34 |
| Côte d'Ivoire 1998 | 24 | 13 | 38 |
| Côte d'Ivoire 2011 | 21 | 17 | 52 |
| Gabon 2000 | 23 | 16 | 32 |
| Gabon 2012 | 23 | 17 | 42 |
| Ghana 1993 | 27 | 21 | 46 |
| Ghana 1998 | 26 | 16 | 50 |
| Ghana 2003 | 25 | 19 | 44 |
| Ghana 2008 | 38 | 16 | 53 |
| Guinea 1999 | 40 | 23 | 45 |
| Guinea 2005 | 30 | 25 | 52 |
| Guinea 2012 | 31 | 21 | 37 |
| Liberia 2007 | 30 | 30 | 40 |
| Mali 1995 | 25 | 26 | 56 |
| Mali 2001 | 23 | 14 | 48 |
| Mali 2006 | 32 | 27 | 55 |
| Niger 1992 | 25 | 20 | 61 |
| Niger 1998 | 26 | 16 | 29 |
| Niger 2006 | 32 | 26 | 53 |
| Niger 2012 | 54 | 21 | 70 |
| Nigeria 1990 | 30 | 28 | 40 |
| Nigeria 2003 | 29 | 20 | 51 |
| Nigeria 2008 | 49 | 41 | 63 |
| Sao Tome et Principe 2008 | 59 | 42 | 66 |
| Senegal 1992 | 33 | 31 | 35 |
| Senegal 2005 | 25 | 16 | 38 |
| Senegal 2010 | 50 | 27 | 56 |
| Sierra Leone 2008 | 53 | 47 | 59 |
| Togo 1998 | 17 | 15 | 35 |

Note: The data quality score represents a weighted combination of the level of missing and/or implausible data, overall sex ratio, overall age ratio, digit preference score for weight, digit preference score for height, standard deviation of WHZ, skewness of WHS, and kurtosis of WHZ.

Supplemental Table 16. Overall child anthropometric data quality scores as calculated using the SMART methodology* among 27 NNS in West Central Africa countries

| Country, survey year | Overall Anthropometric Data Quality | | |
| --- | --- | --- | --- |
|  | Total score at the country level | Minimum score at the regional level | Maximum score at the regional level |
| Benin 2008 | 25 | 15 | 36 |
| Burkina Faso 2012 | 10 | 0 | 18 |
| Cameroon 2011 | 35 | 38 | 39 |
| Central African Rep 2012 | 9 | 8 | 23 |
| The Gambia 2012 | 11 | 1 | 15 |
| Mali 2011 | 14 | 6 | 24 |
| Sierra Leone 2010 | 6 | 1 | 20 |
| Senegal 2012 | 17 | 9 | 30 |
| Guinea-Bissau 2008 | 11 | 13 | 20 |
| Guinée Conakay 2012 | 35 | 8 | 20 |
| Liberia 2010 | 18 | 8 | 36 |
| Liberia 2011 | 12 | 12 | 34 |
| Chad June 2012 | 16 | 7 | 34 |
| Chad (7 regions) Dec/Jan 2012-13 | 15 | 11 | 22 |
| Mauritania 2006 | 24 | 19 | 30 |
| Mauritania March 2008 | 19 | 17 | 28 |
| Mauritania Dec 2008 | 6 | 4 | 23 |
| Mauritania 2009 | 12 | 2 | 18 |
| Mauritania July 2010 | 8 | 8 | 20 |
| Mauritania Dec 2010 | 11 | 4 | 18 |
| Mauritania July 2011 | 4 | 12 | 29 |
| Mauritania Dec 2011 | 9 | 0 | 21 |
| Mauritania July 2012 | 10 | 6 | 19 |
| Niger 2012 | 7 | 6 | 14 |
| Nigeria (Northern States) 2011 | 18 | 14 | 38 |
| Togo June 2012 | 8 | 10 | 32 |
| Togo Dec 2012 | 9 | 3 | 30 |

* Note: The data quality score represents a weighted combination of the level of missing and/or implausible data, overall sex ratio, overall age ratio, digit preference score for weight, digit preference score for height, standard deviation of WHZ, skewness of WHS, and kurtosis of WHZ.

Supplemental Figure 1. Empirical age distributions of children 0-59 months in DHS surveys irrespective of survival status along with expected values.

| 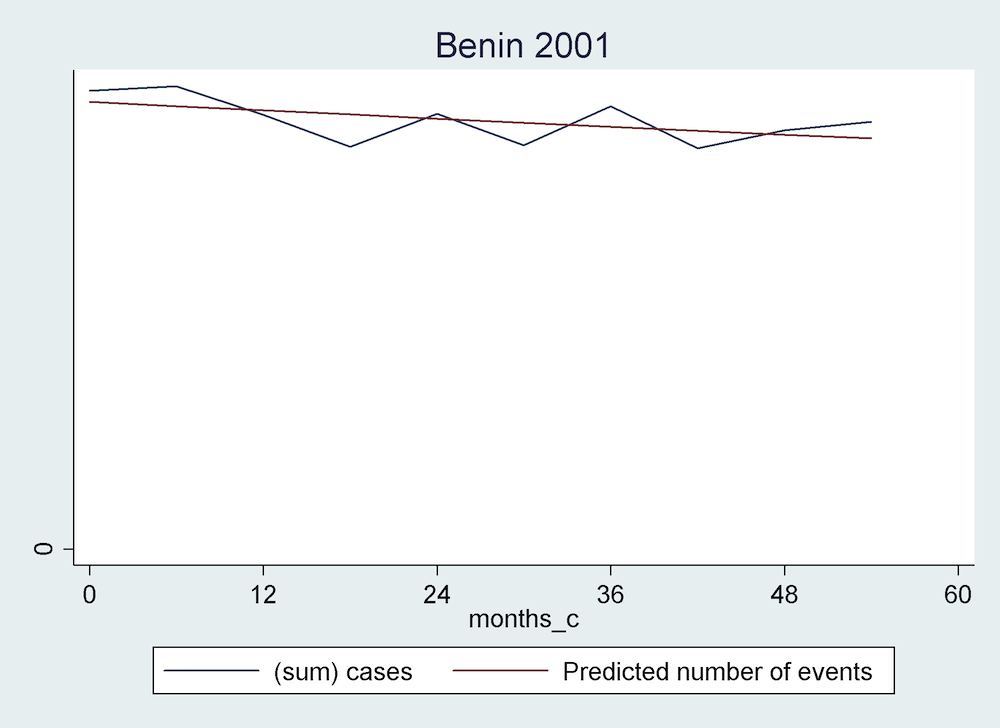 | 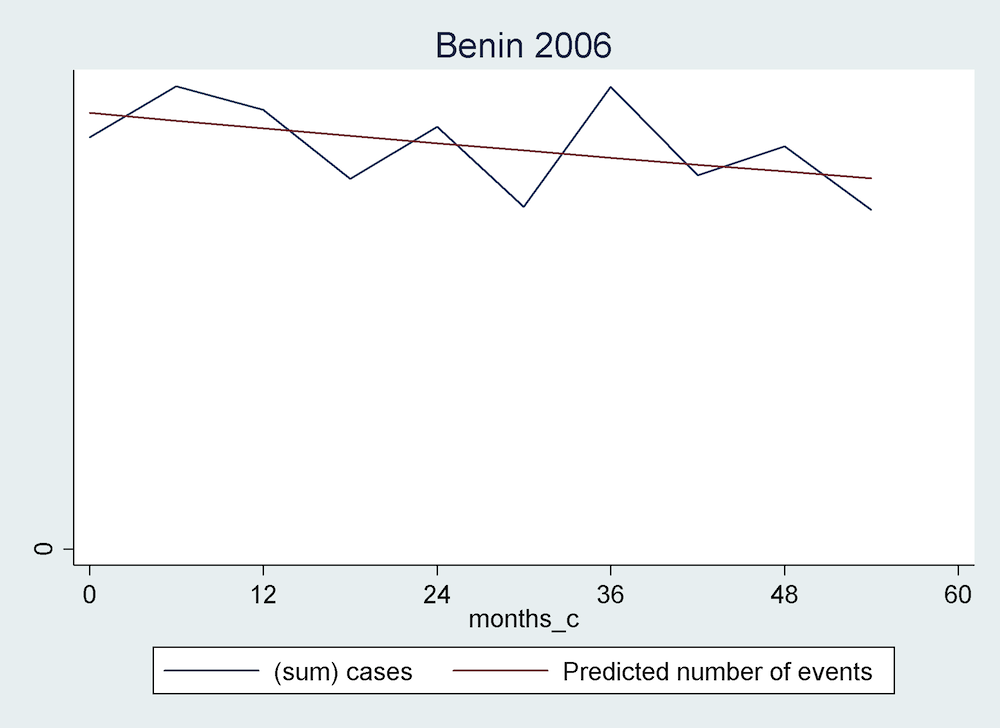 |
| --- | --- |
| 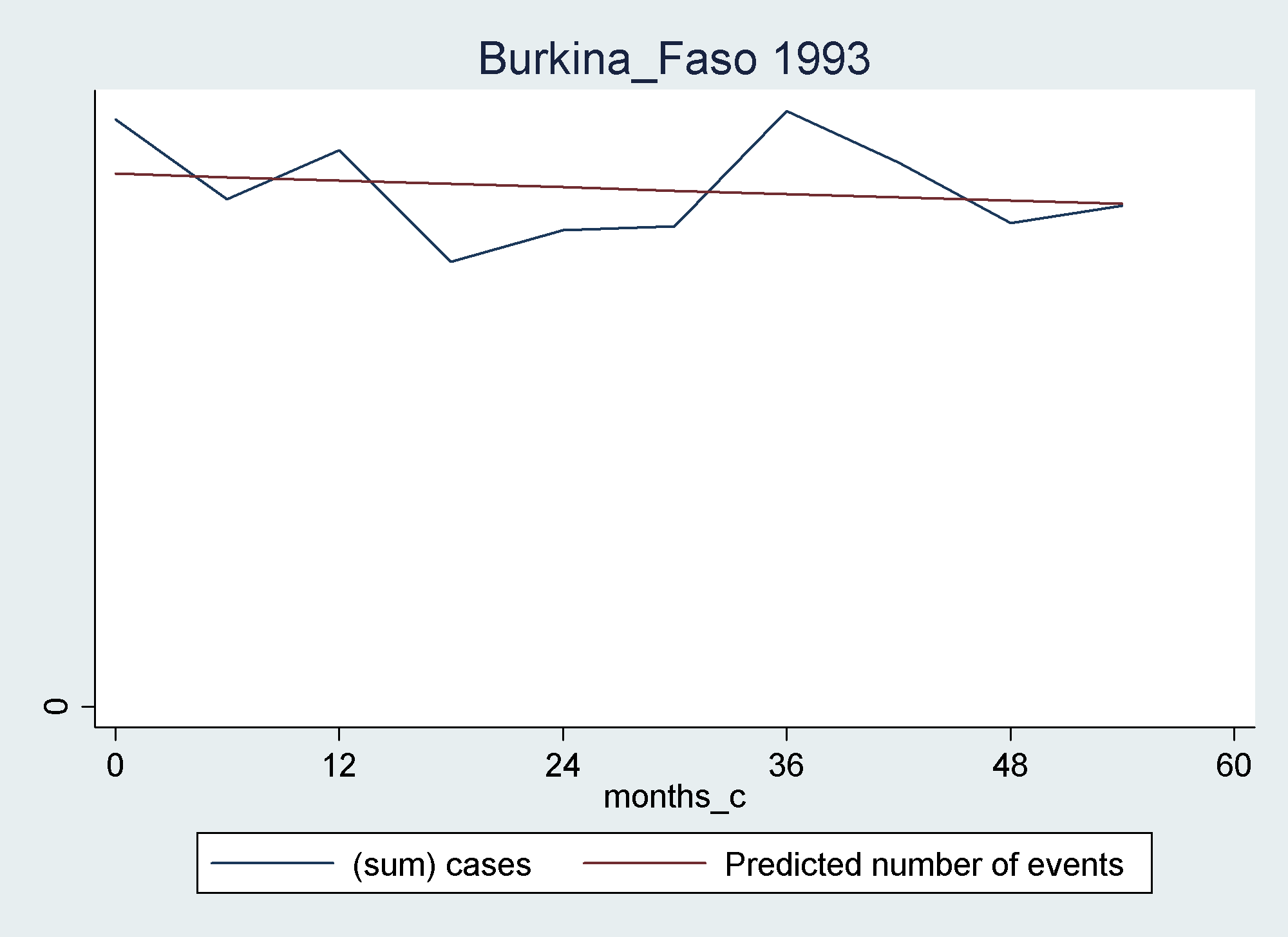 | 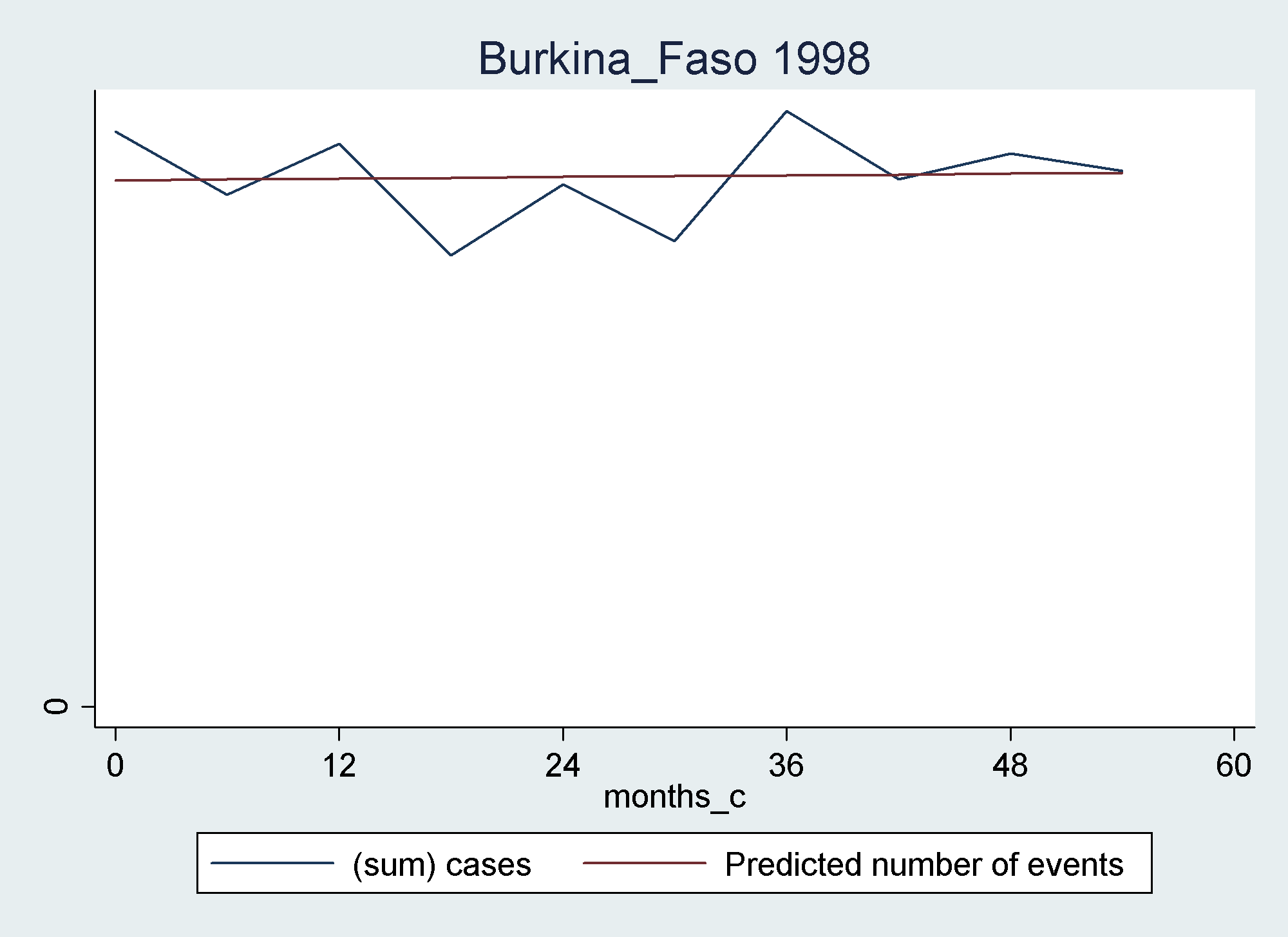 |
| 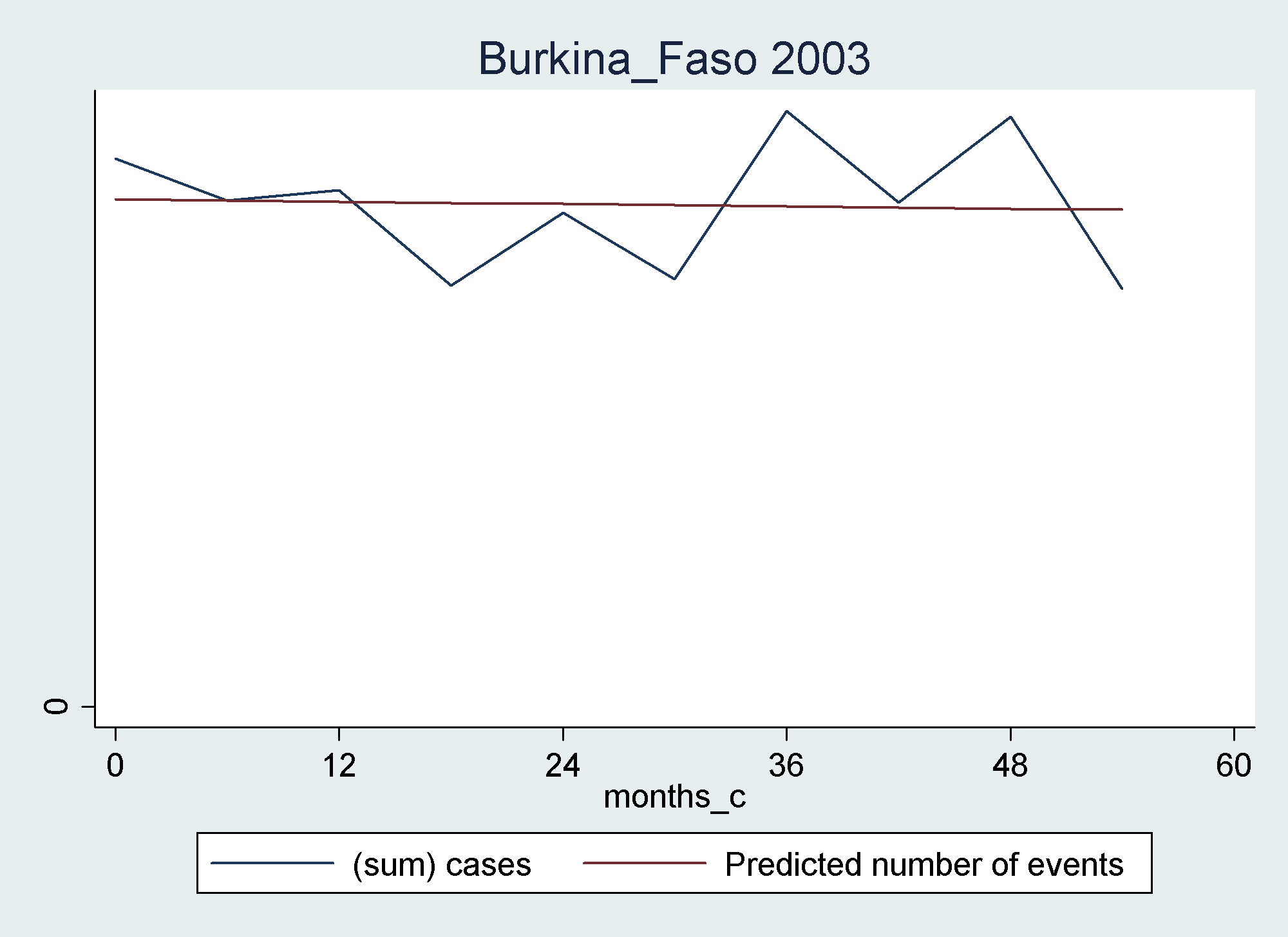 | 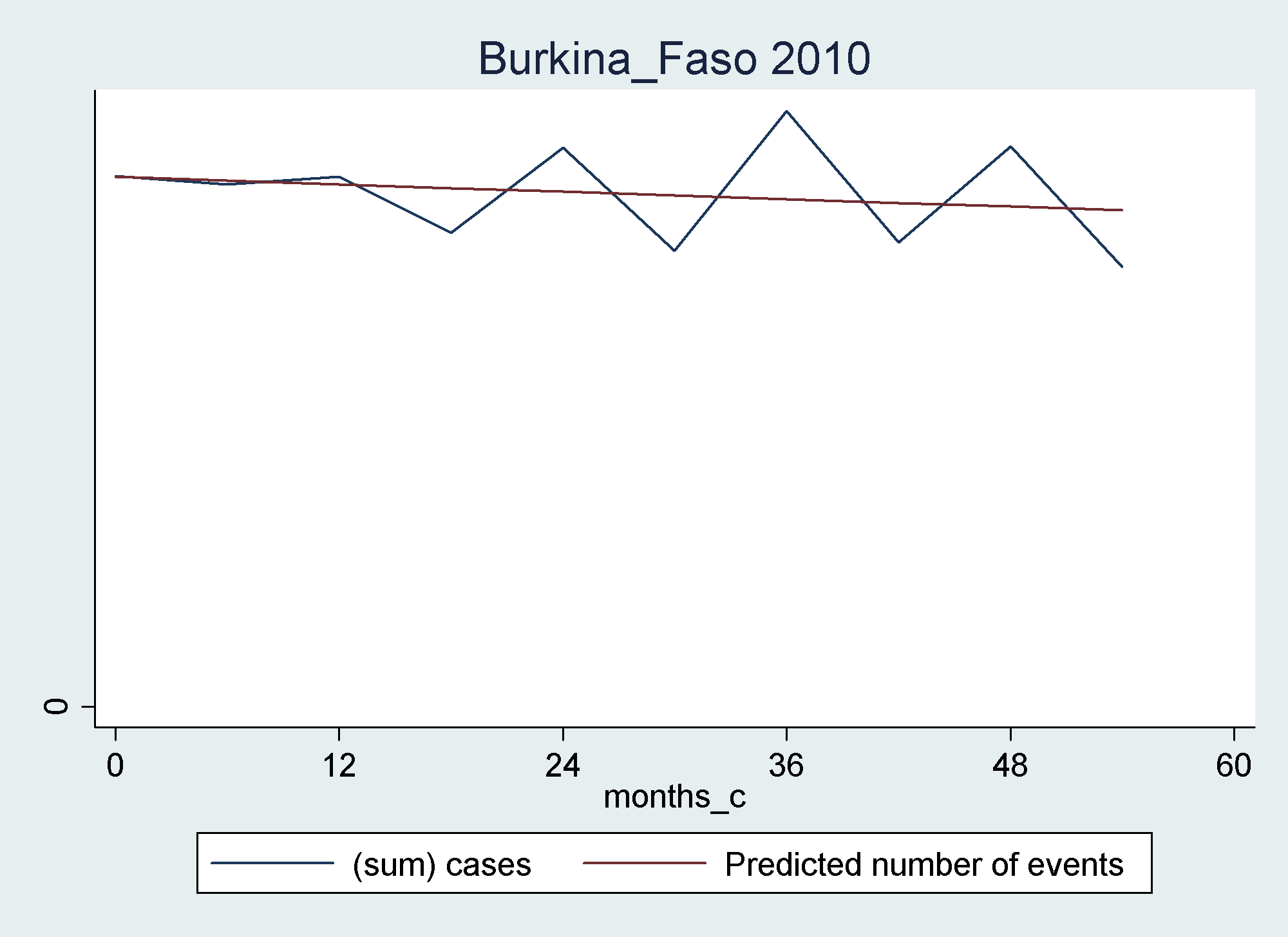 |
| 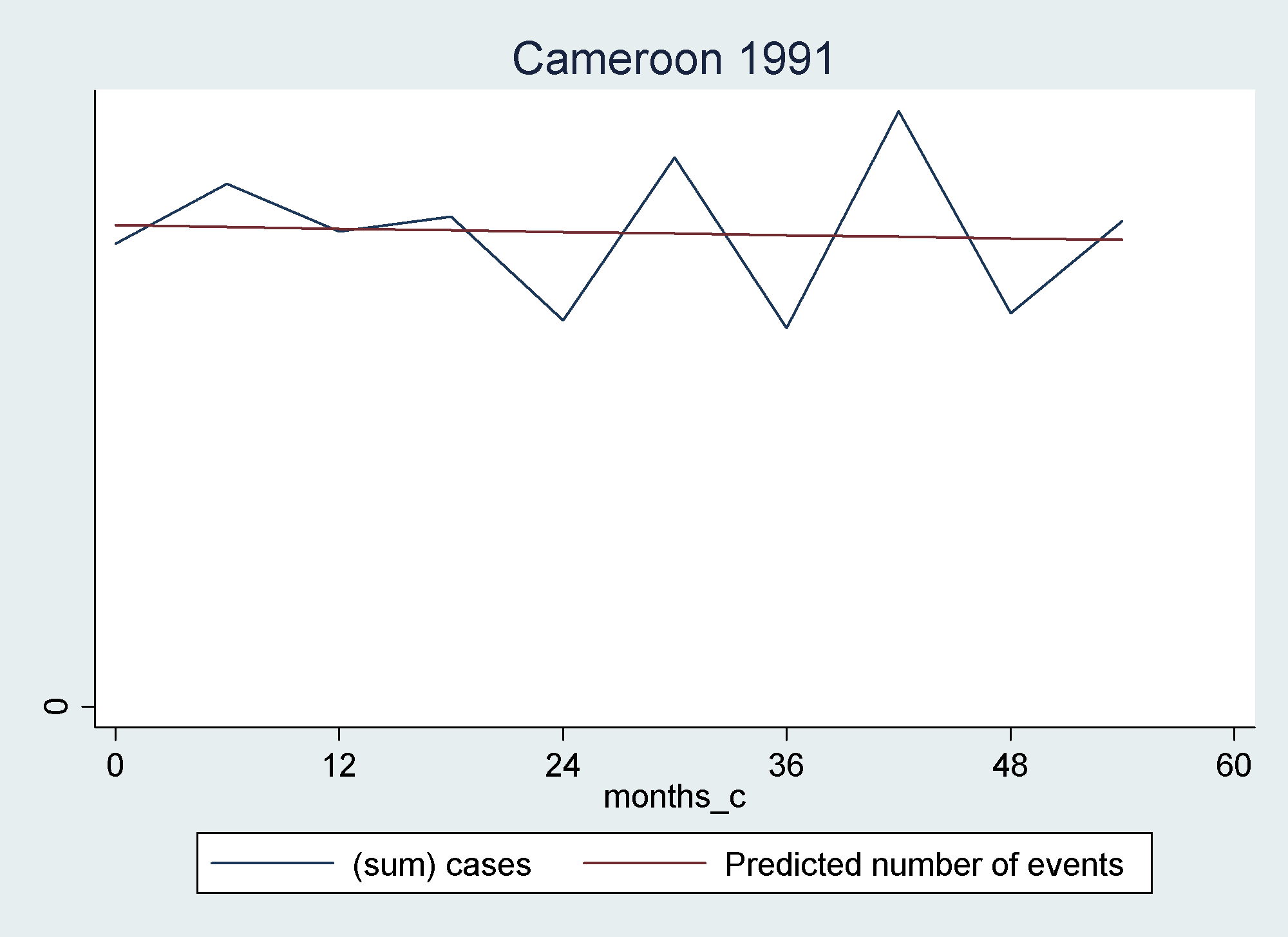 | 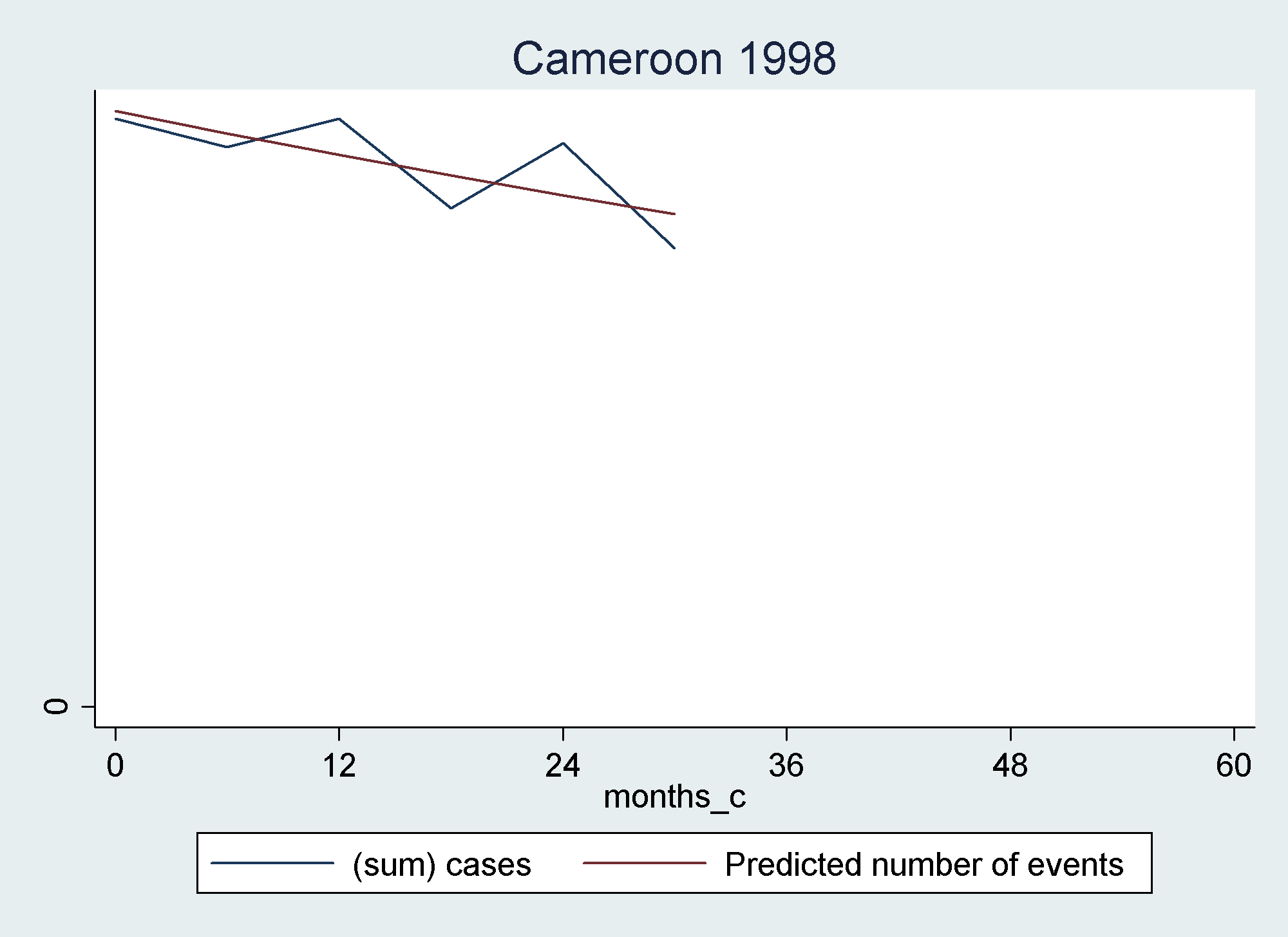 |
| 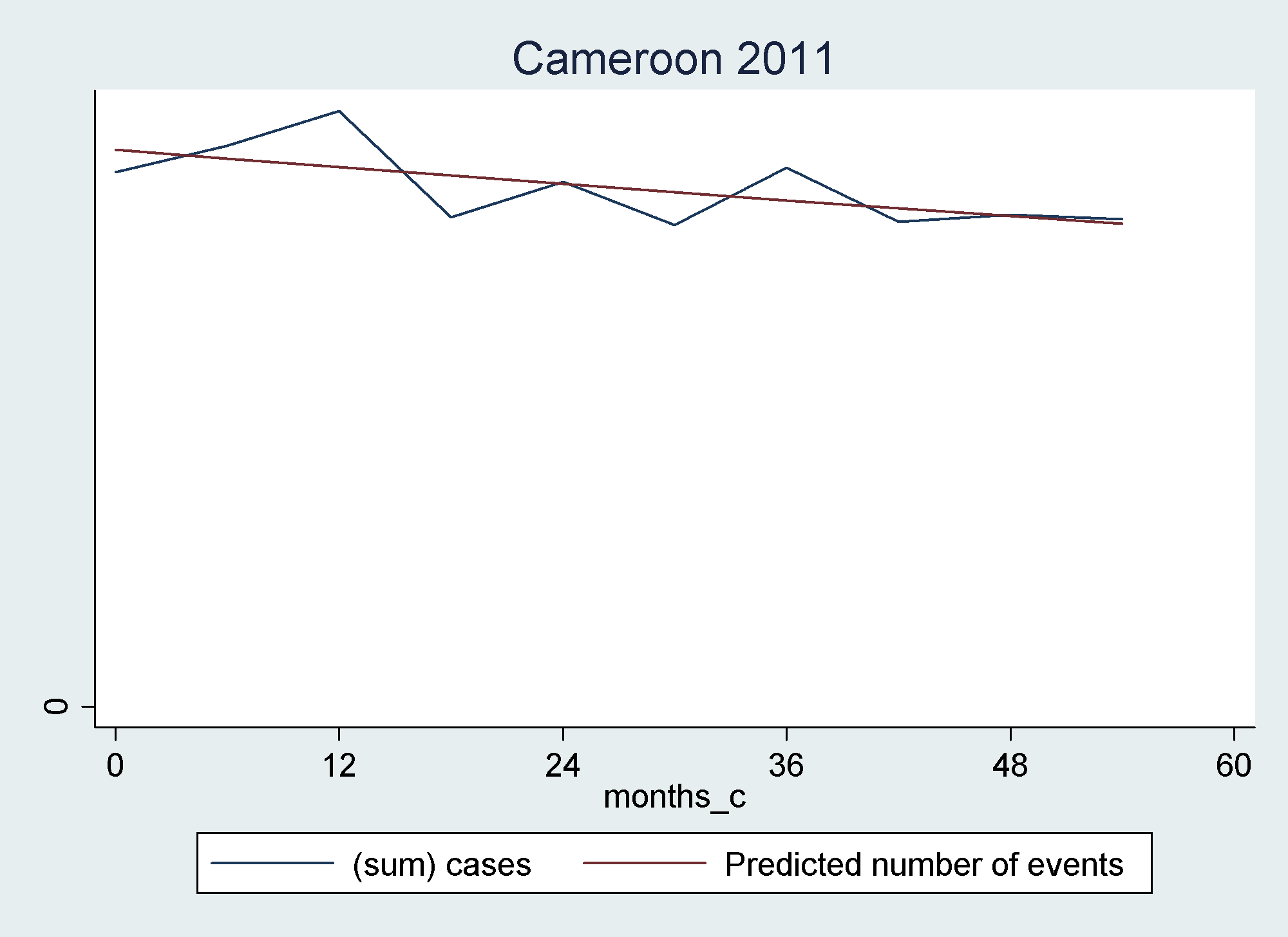 | 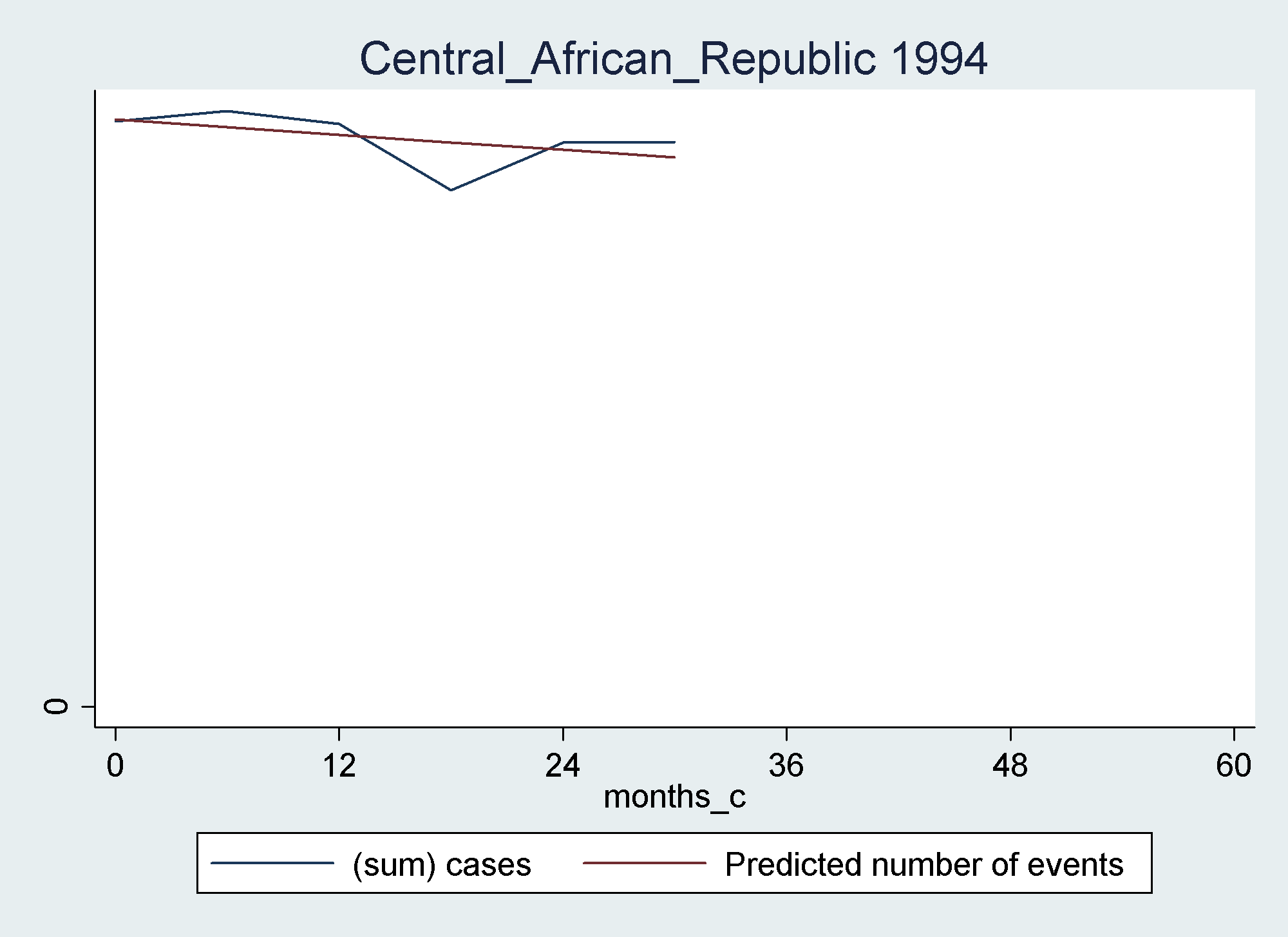 |
| 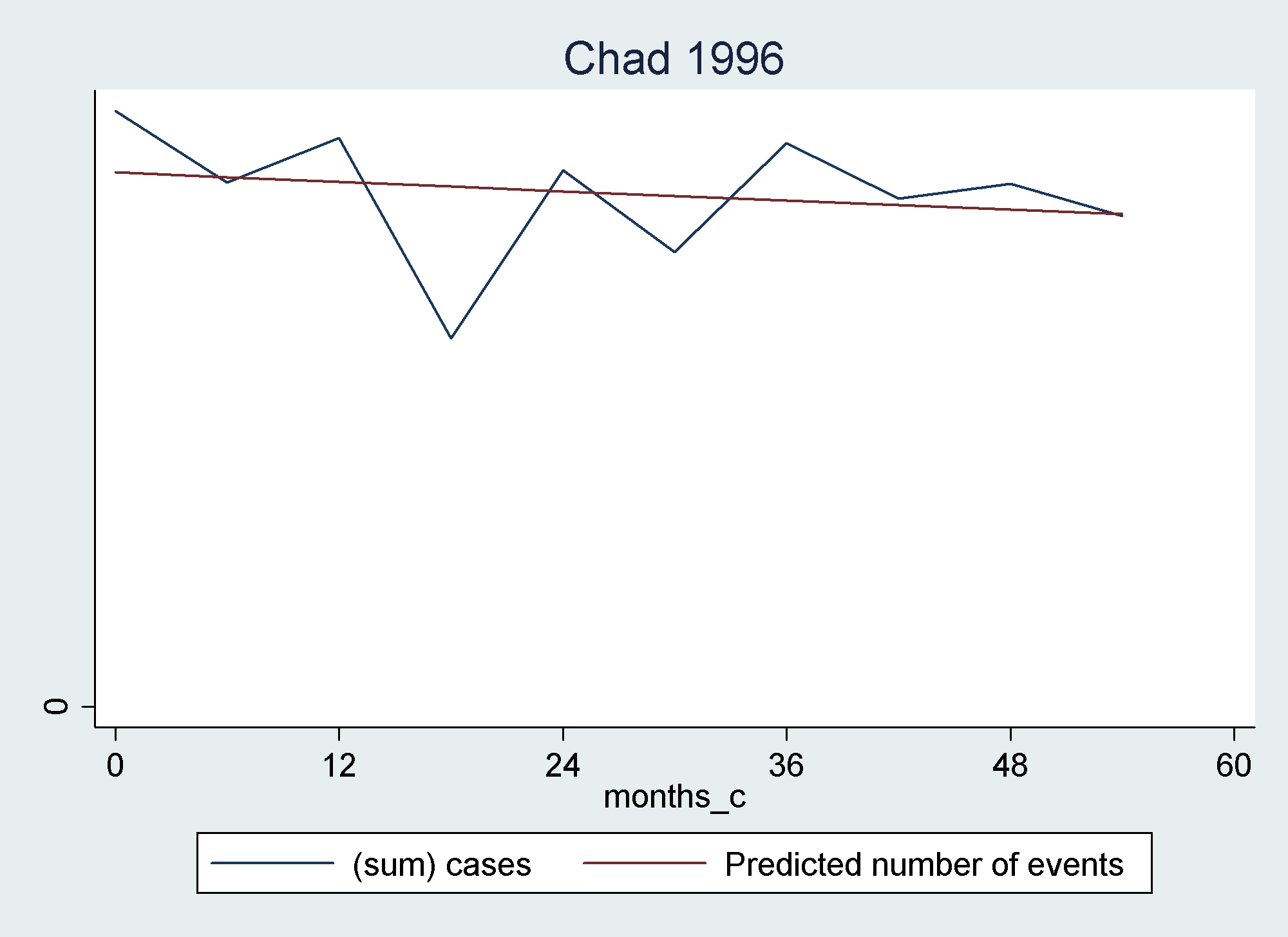 | 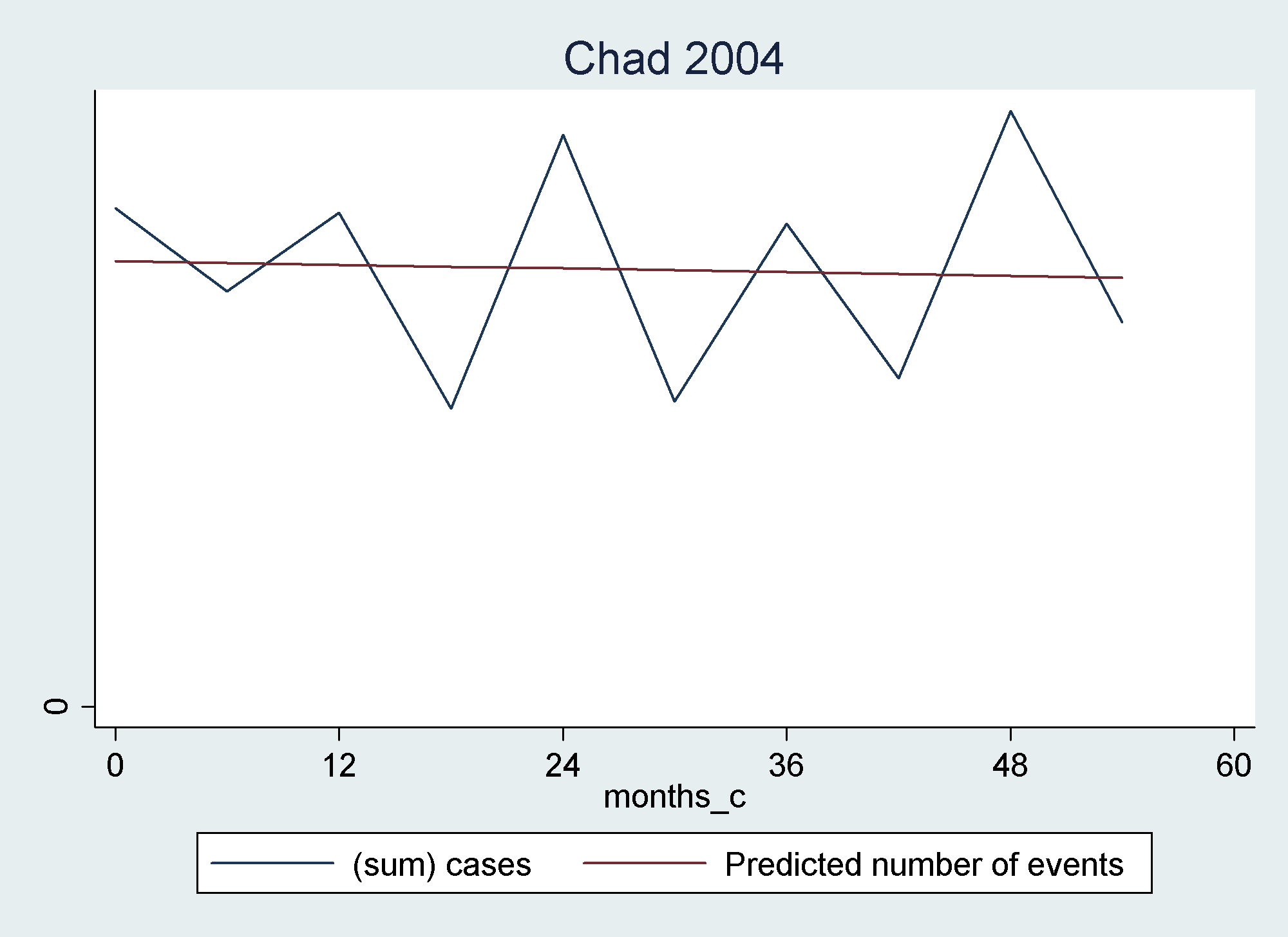 |
| 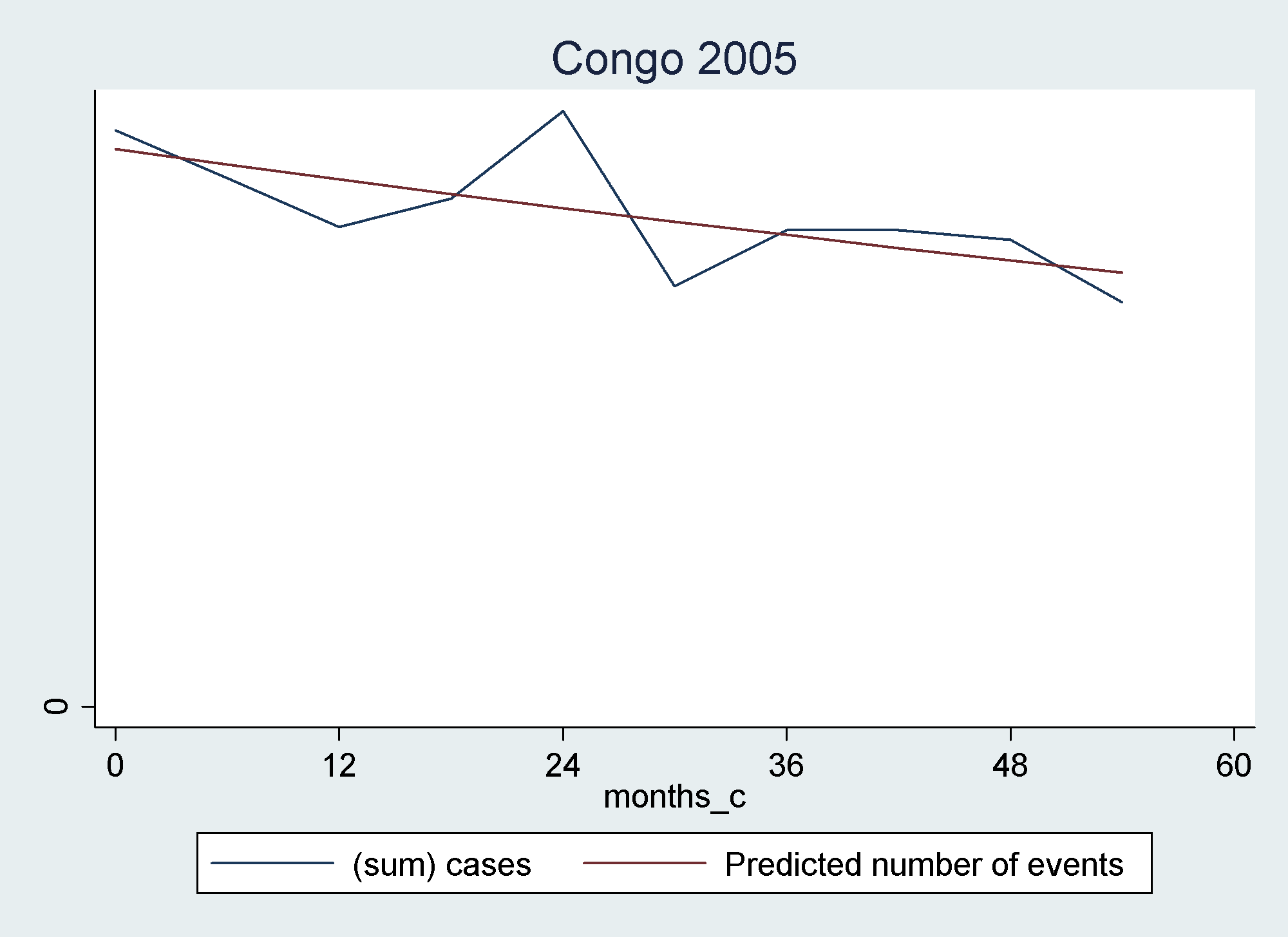 | 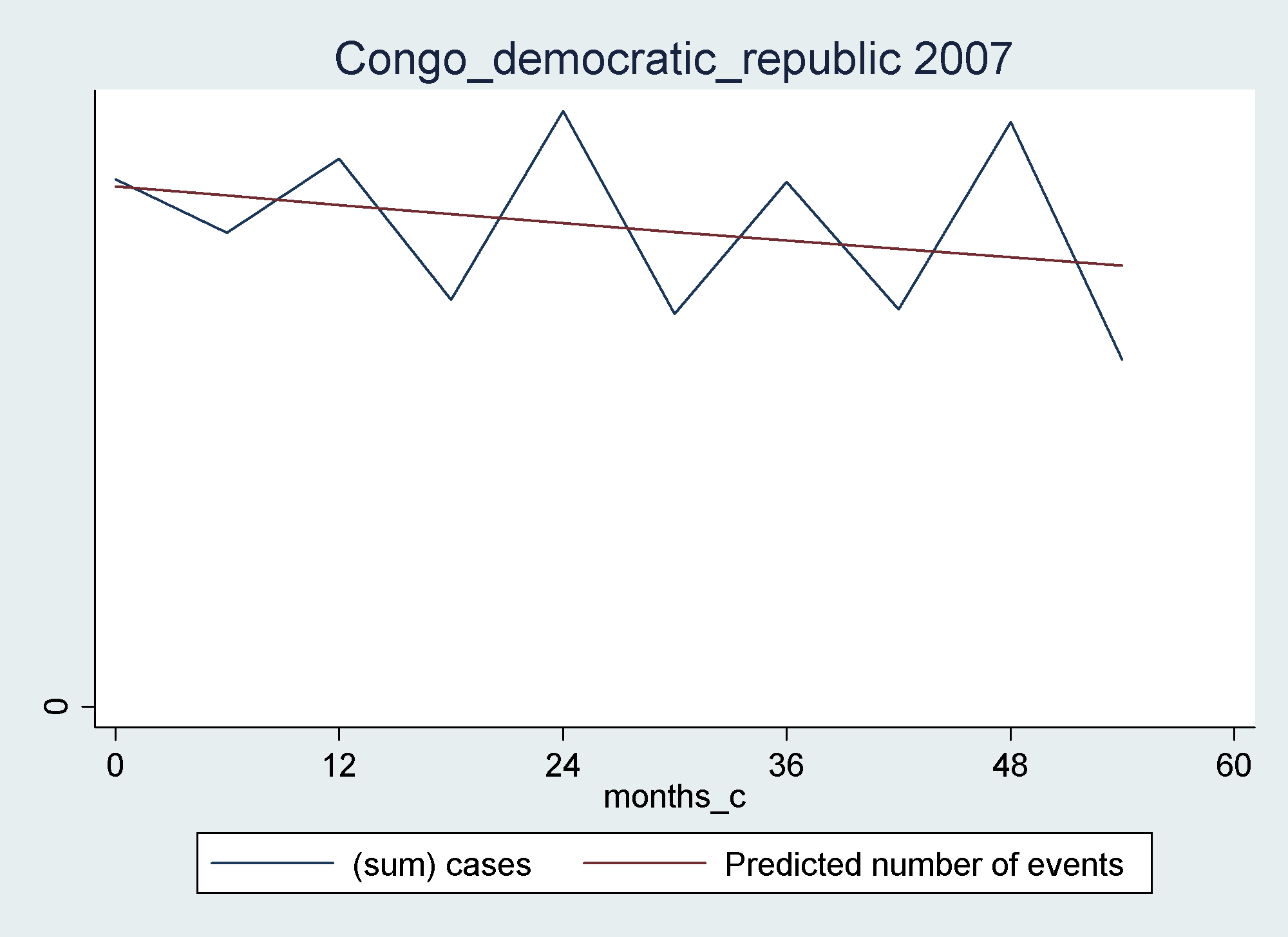 |
| 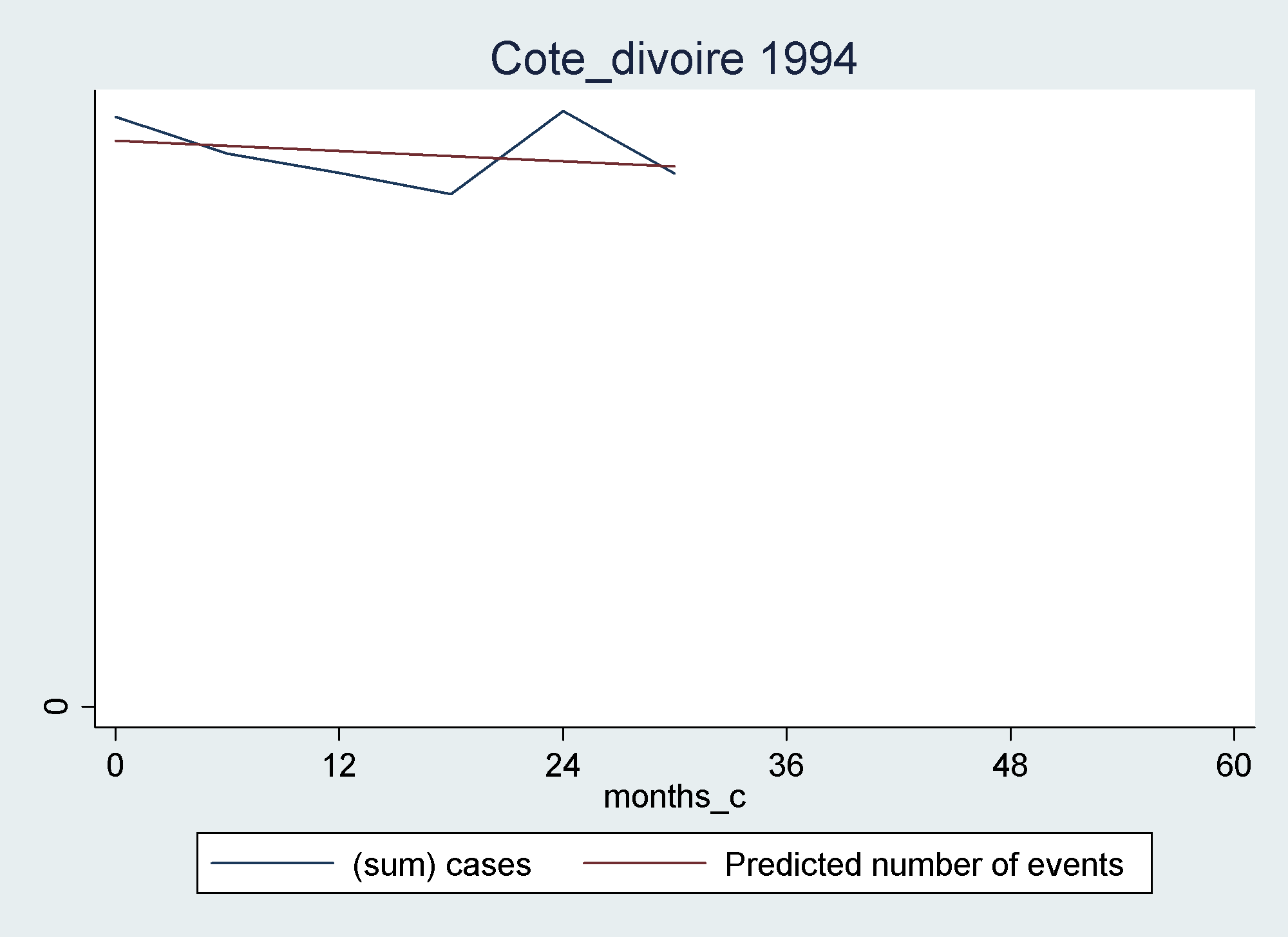 | 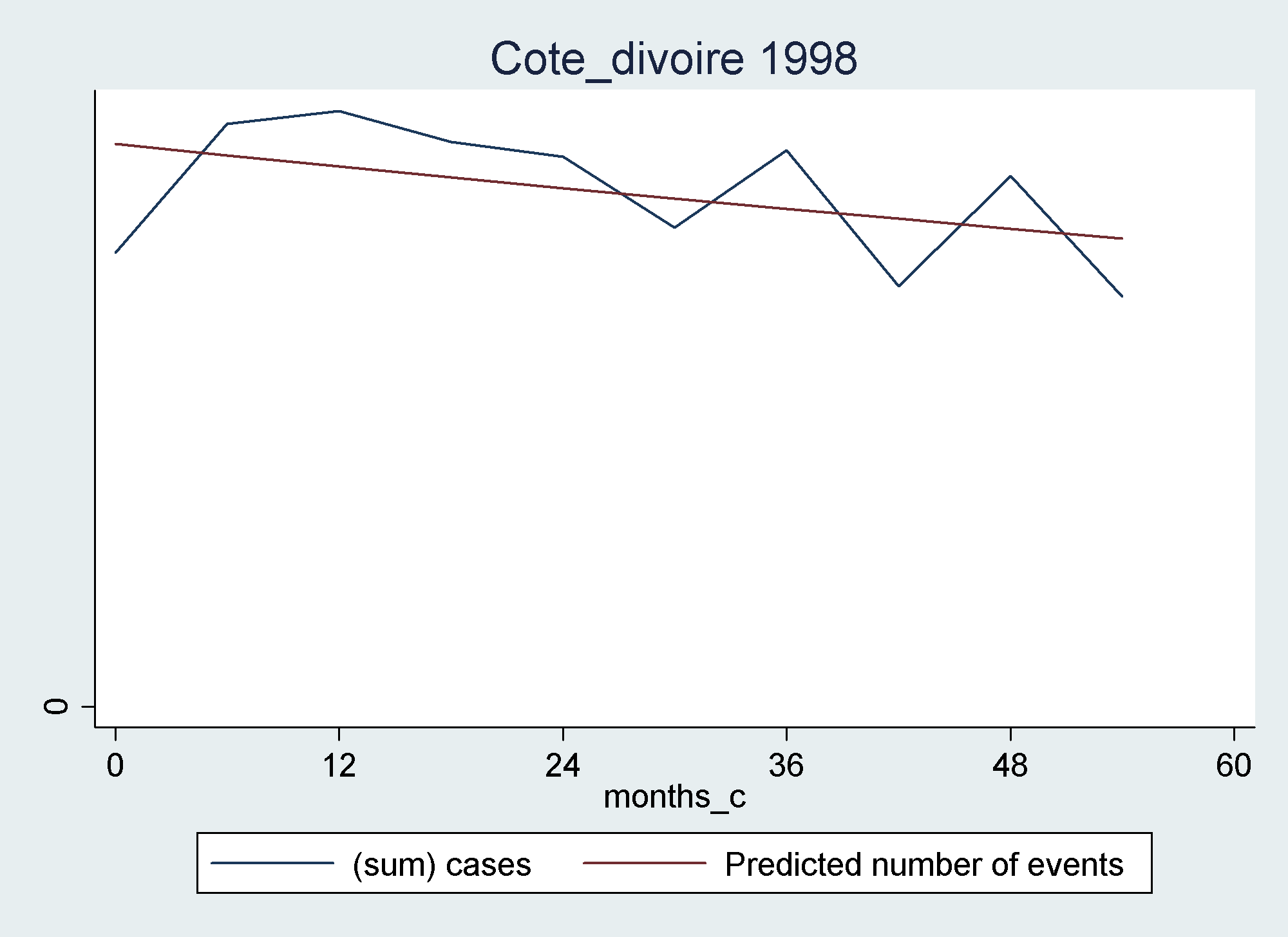 |
| 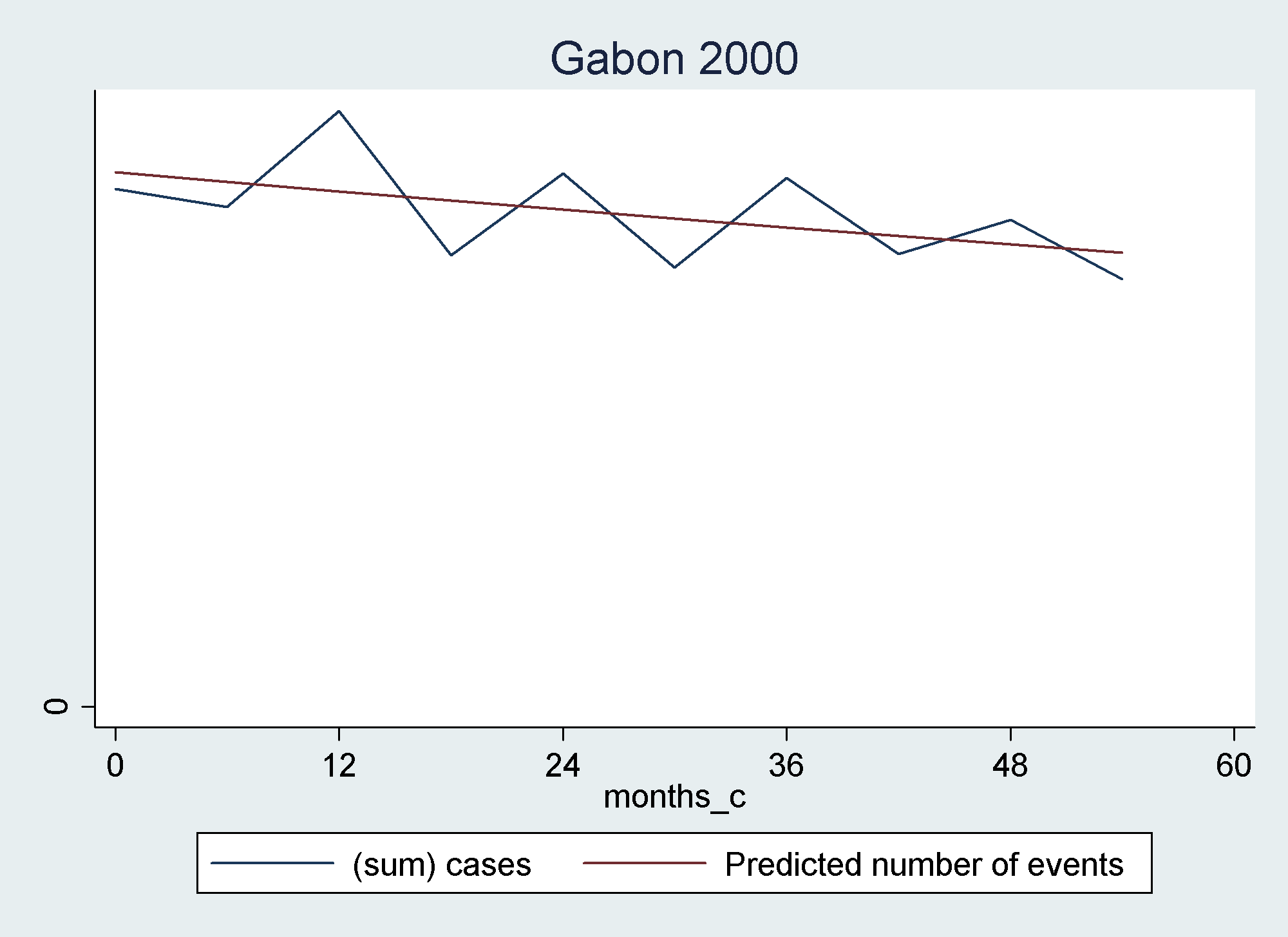 | 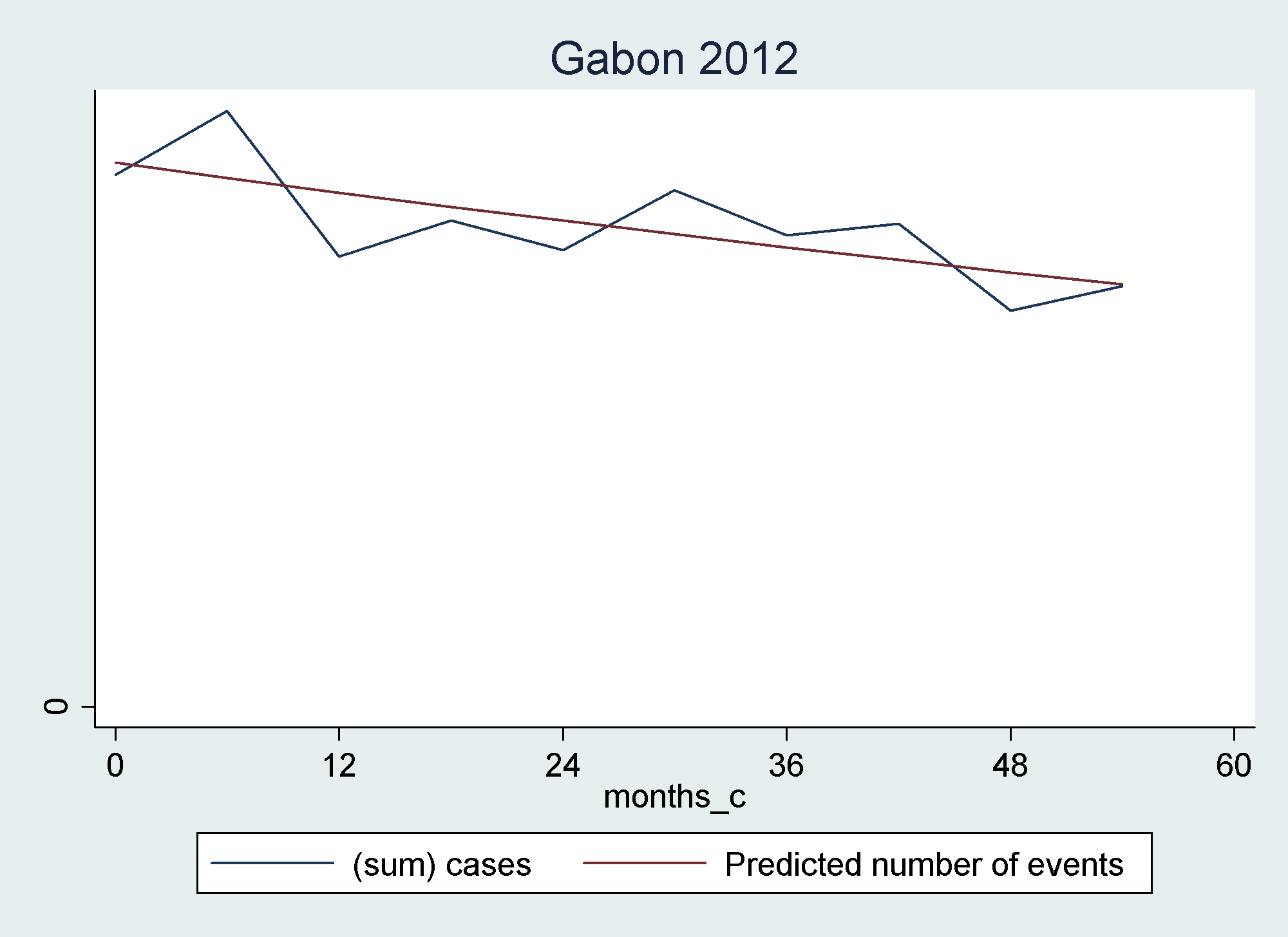 |
| 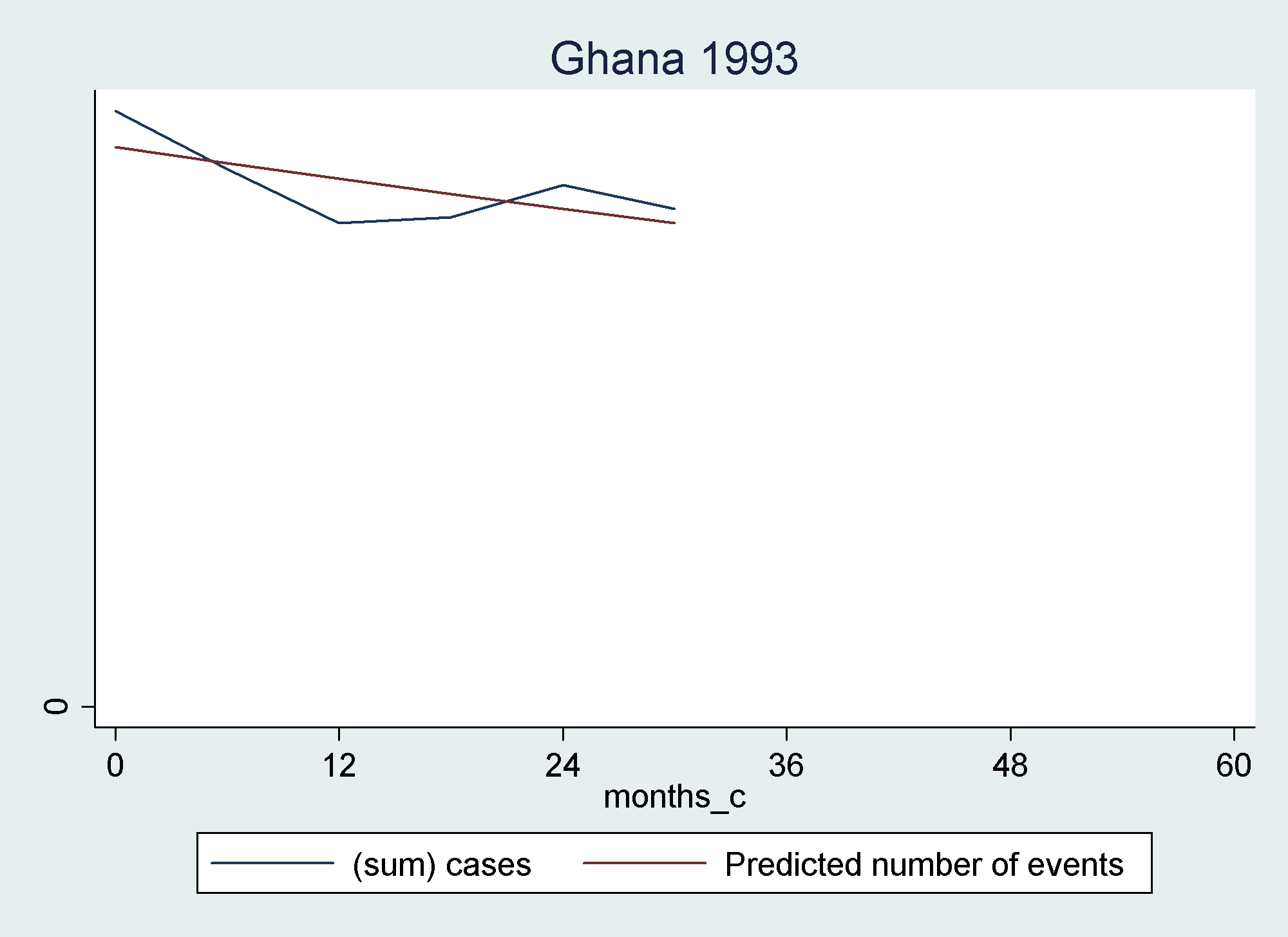 | 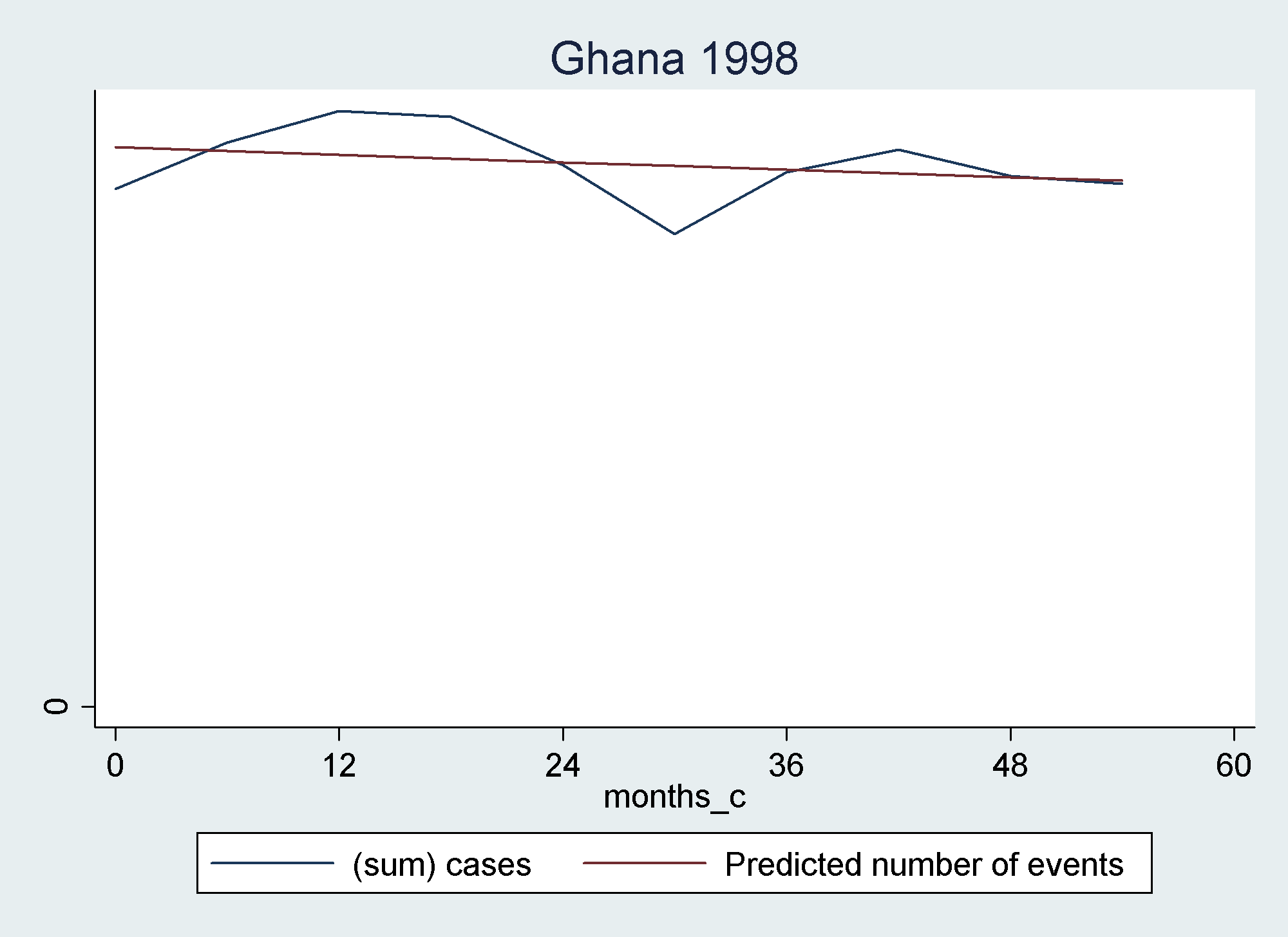 |
| 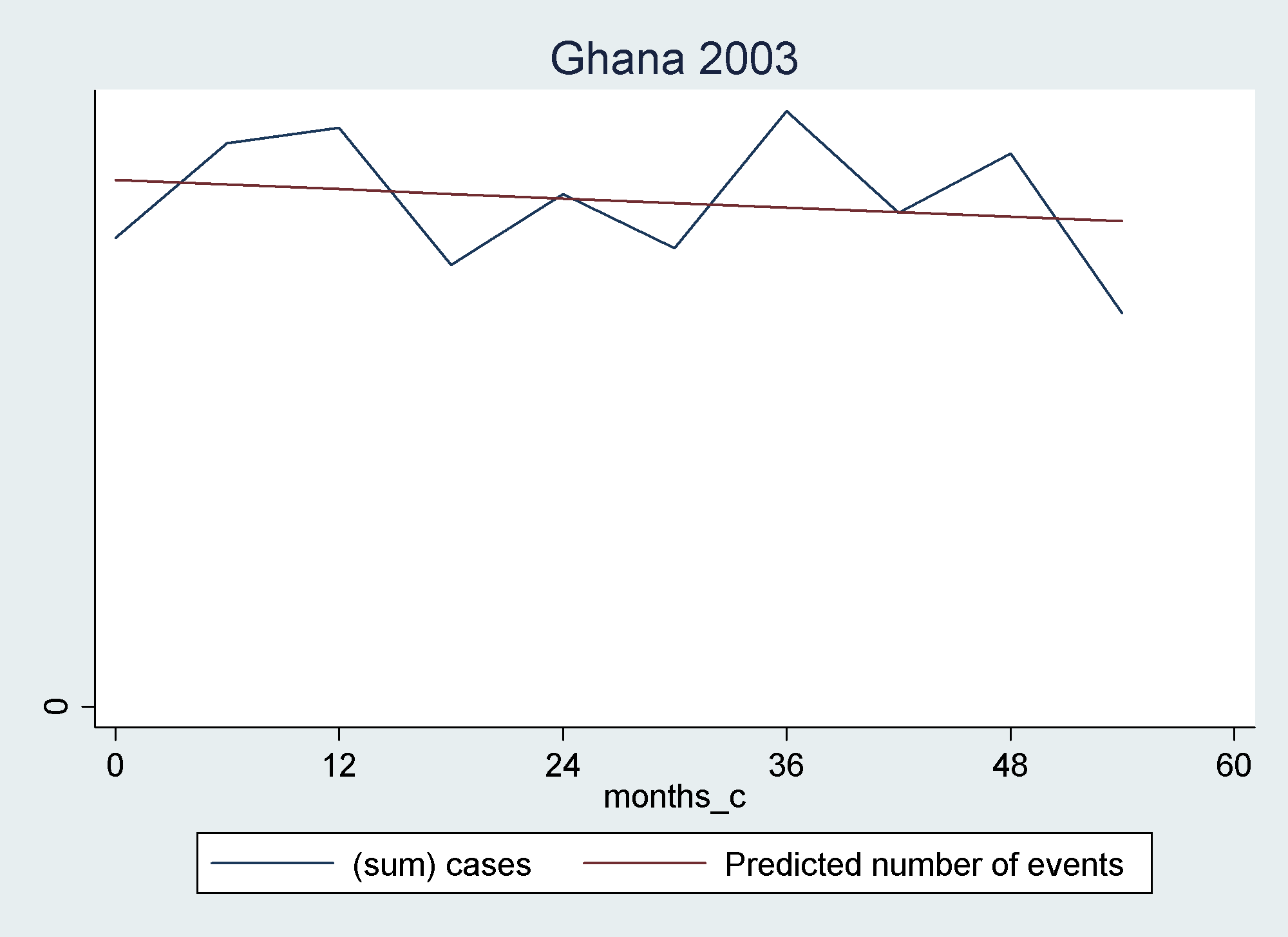 | 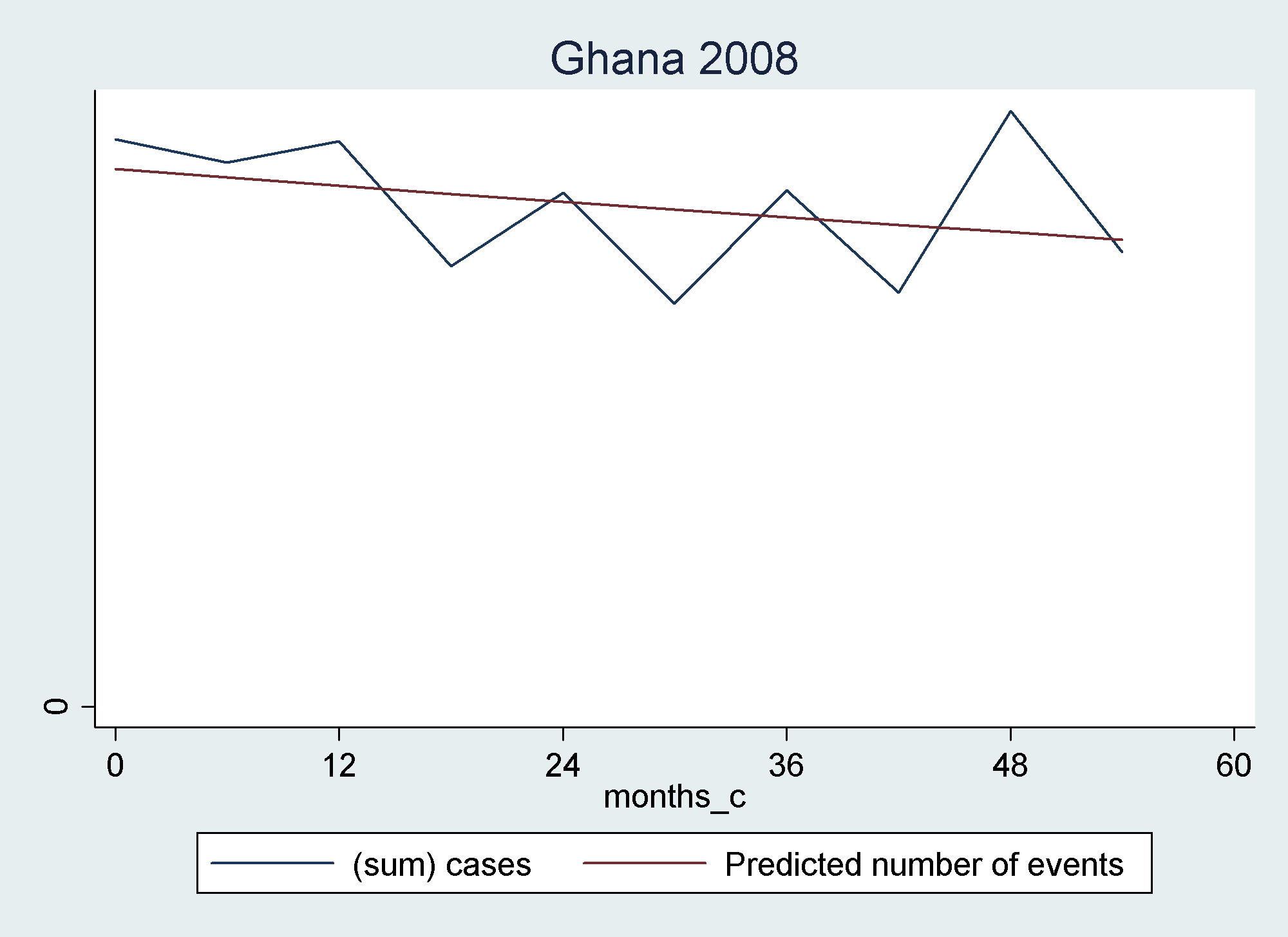 |
| 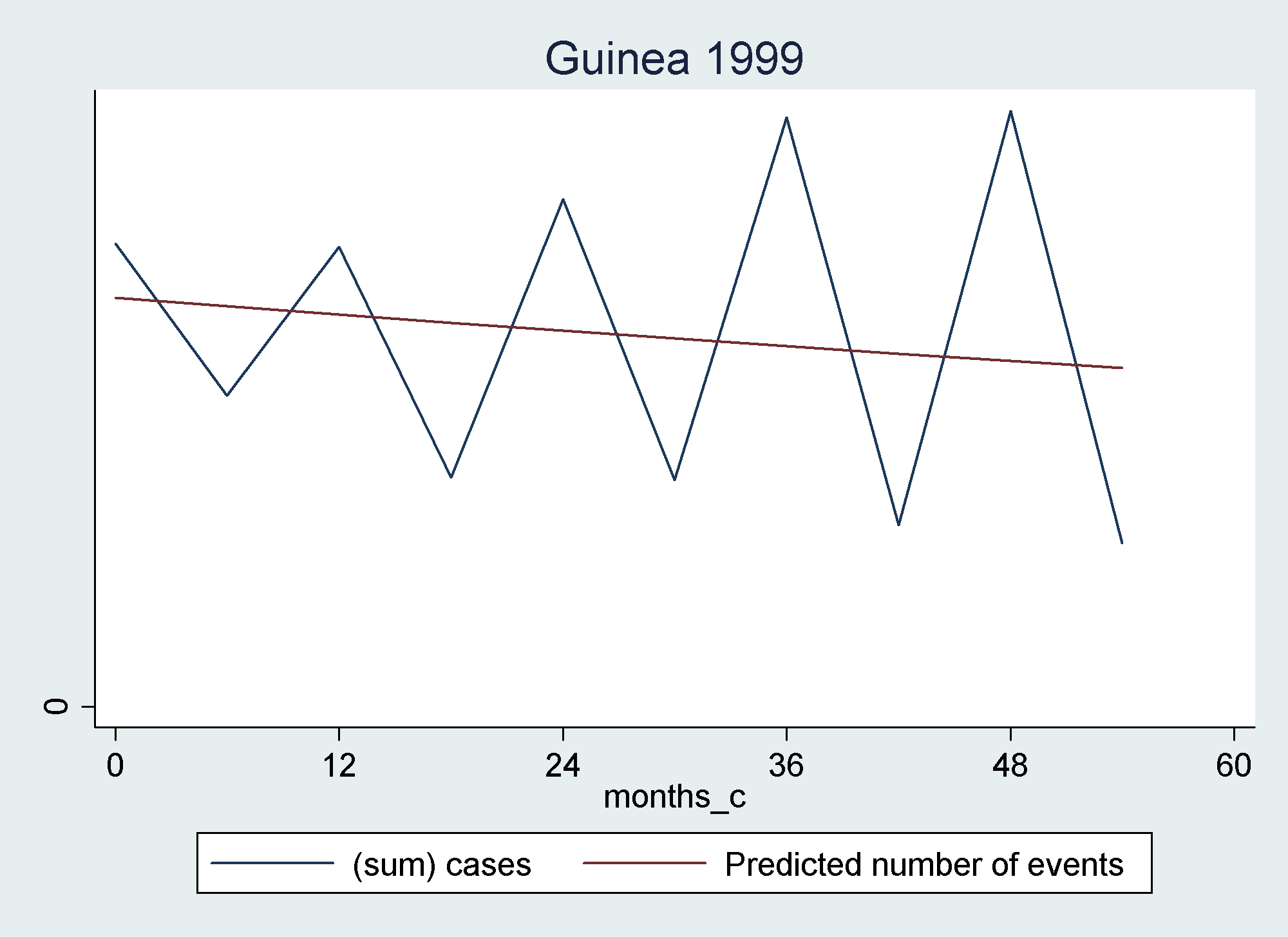 | 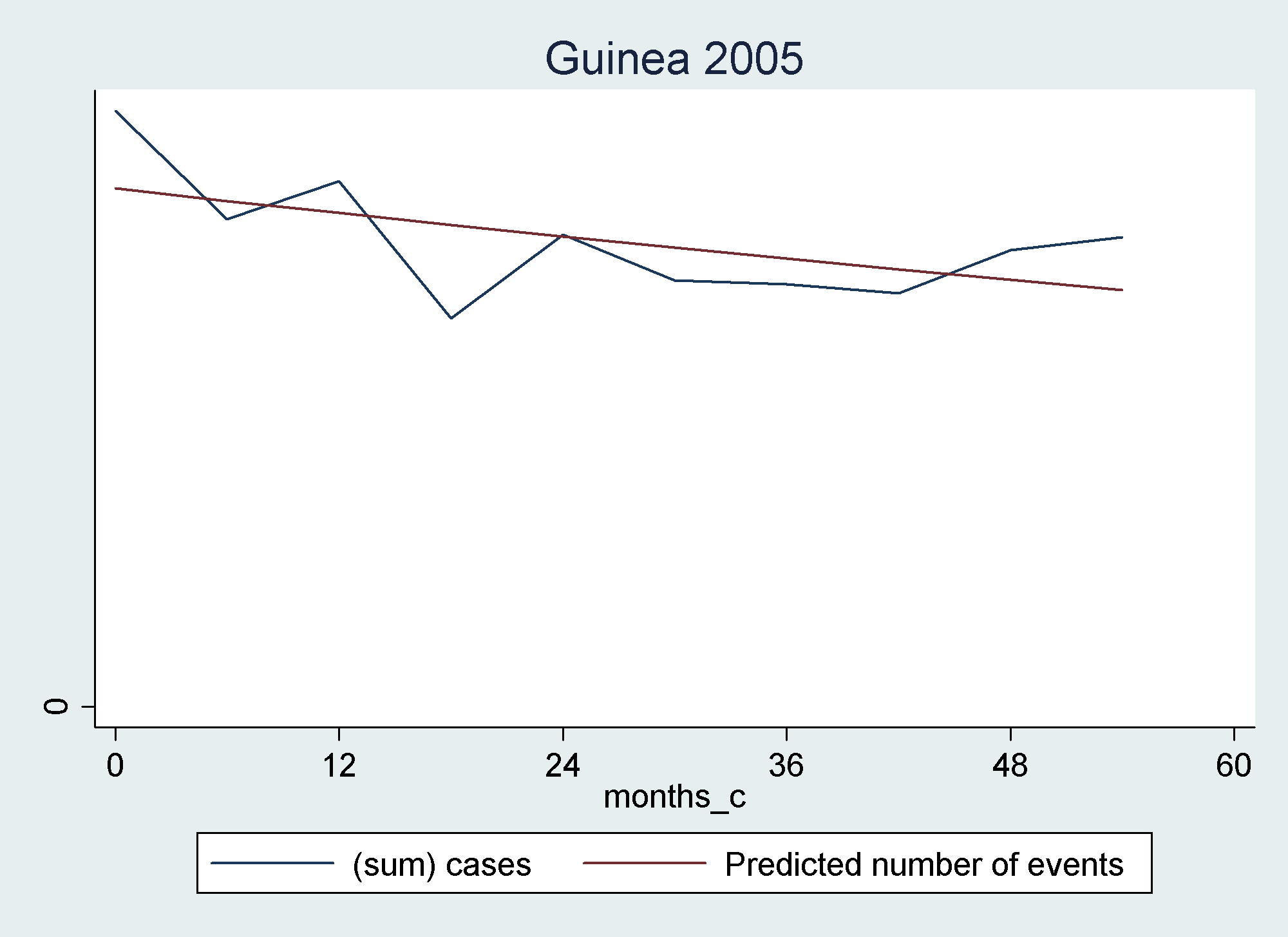 |
| 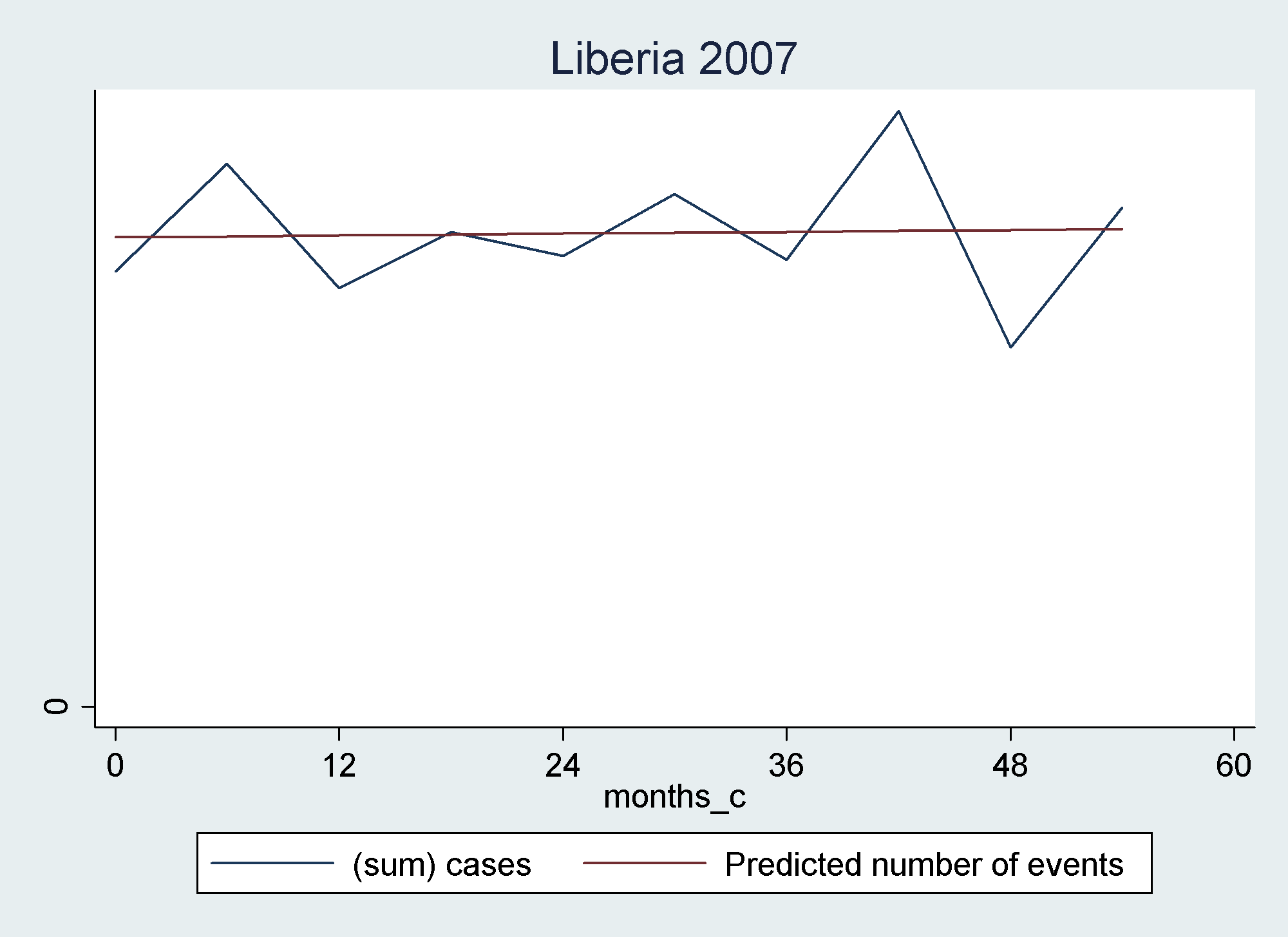 | 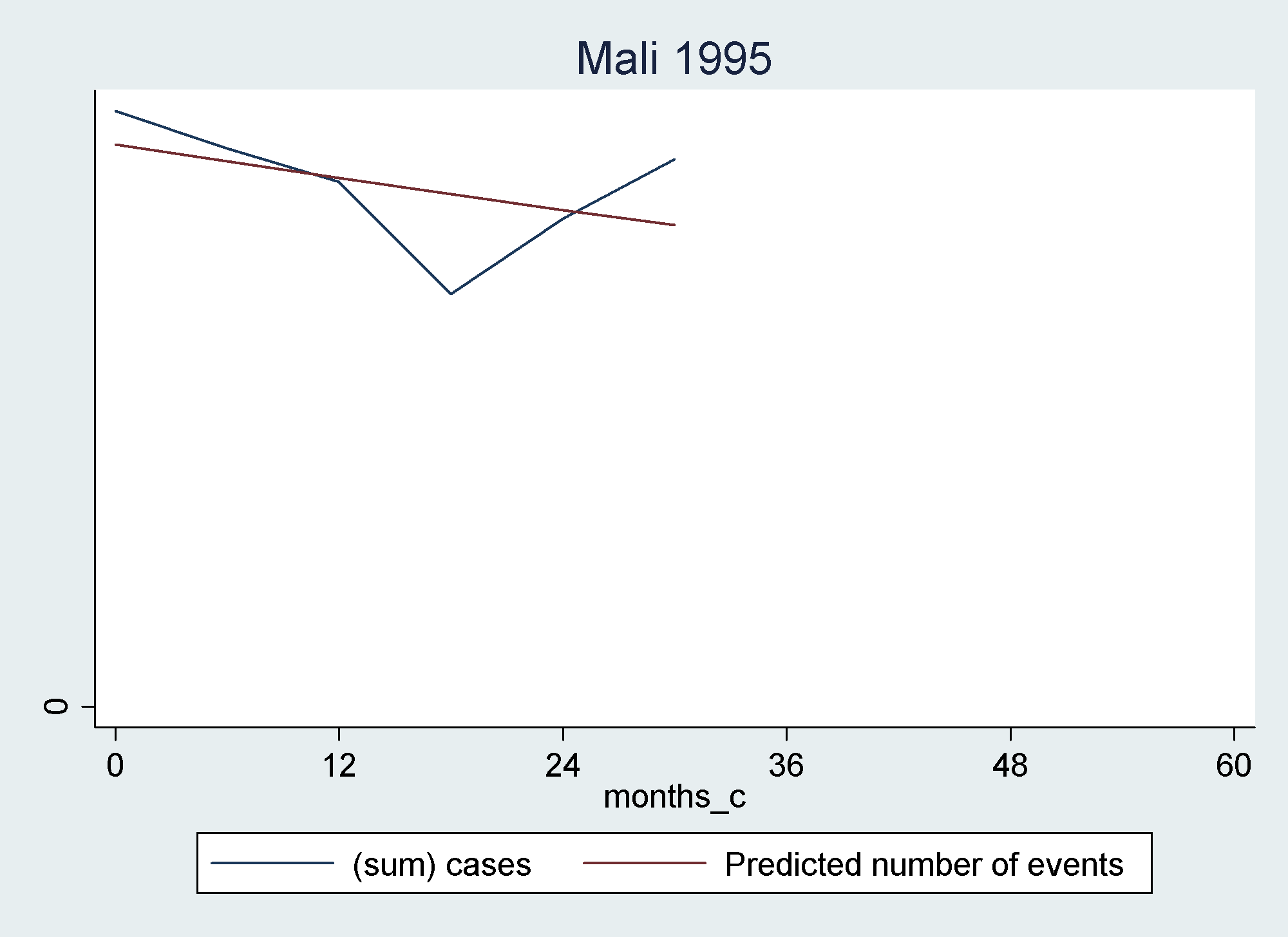 |
| 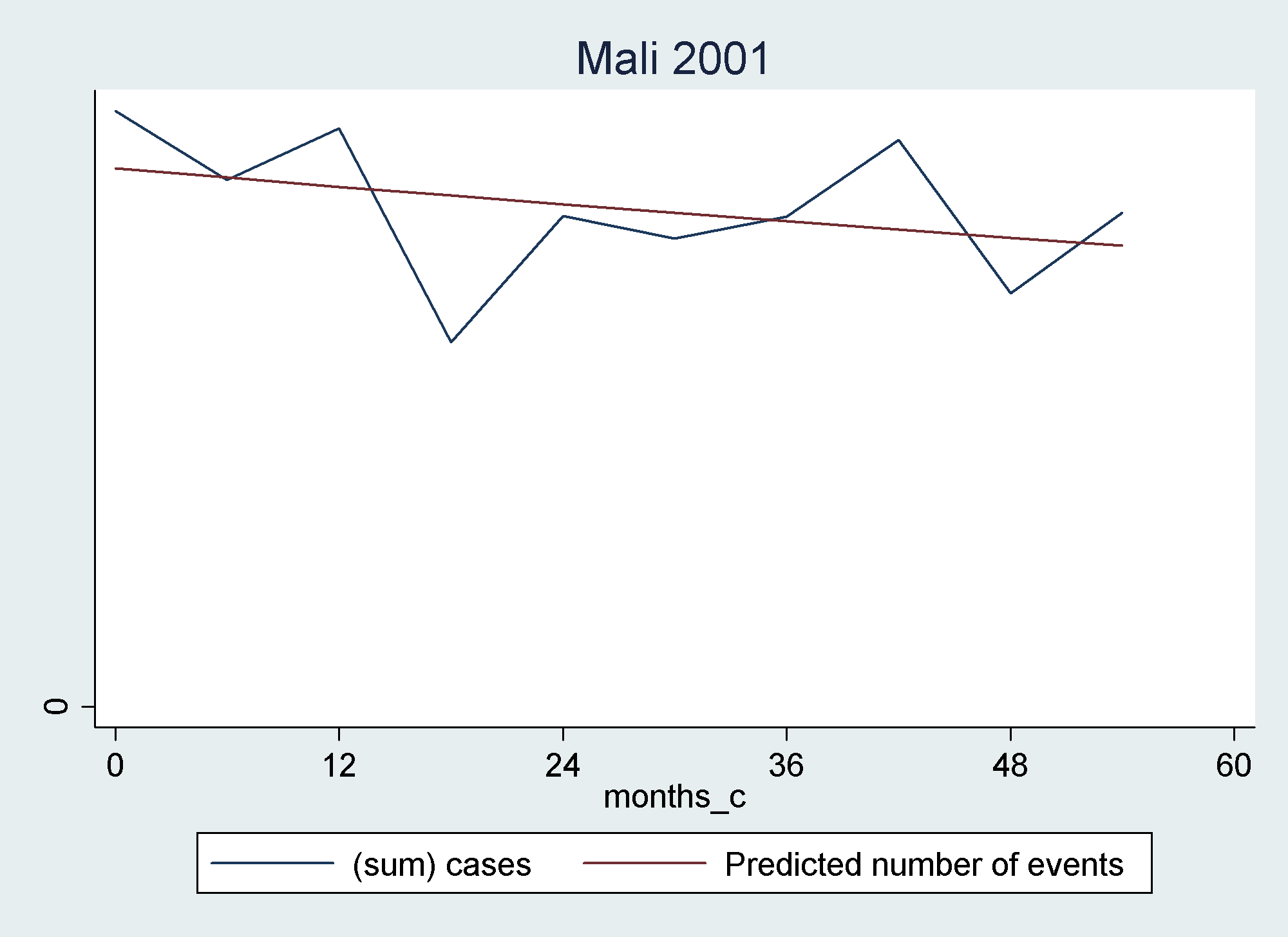 | 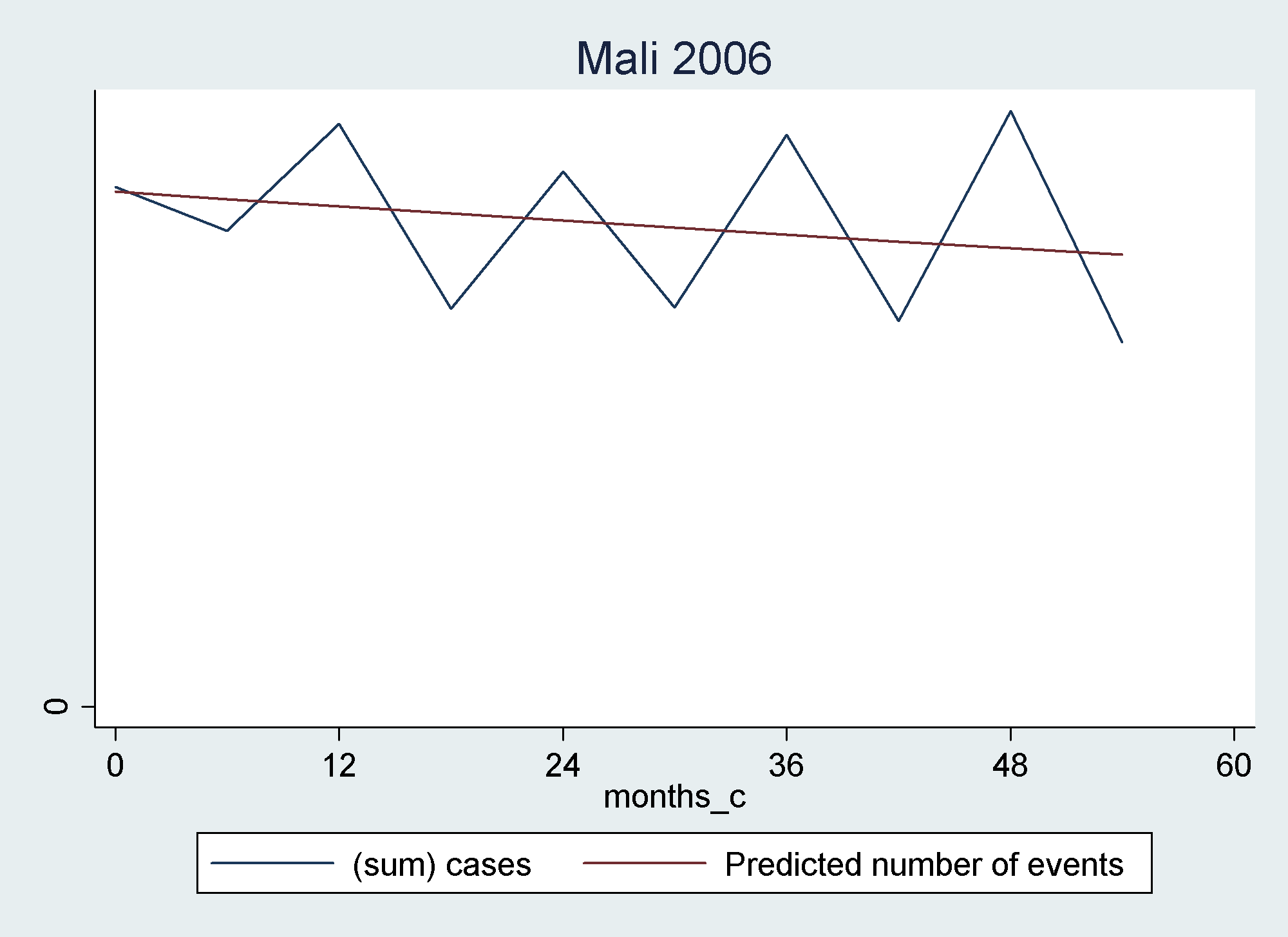 |
| 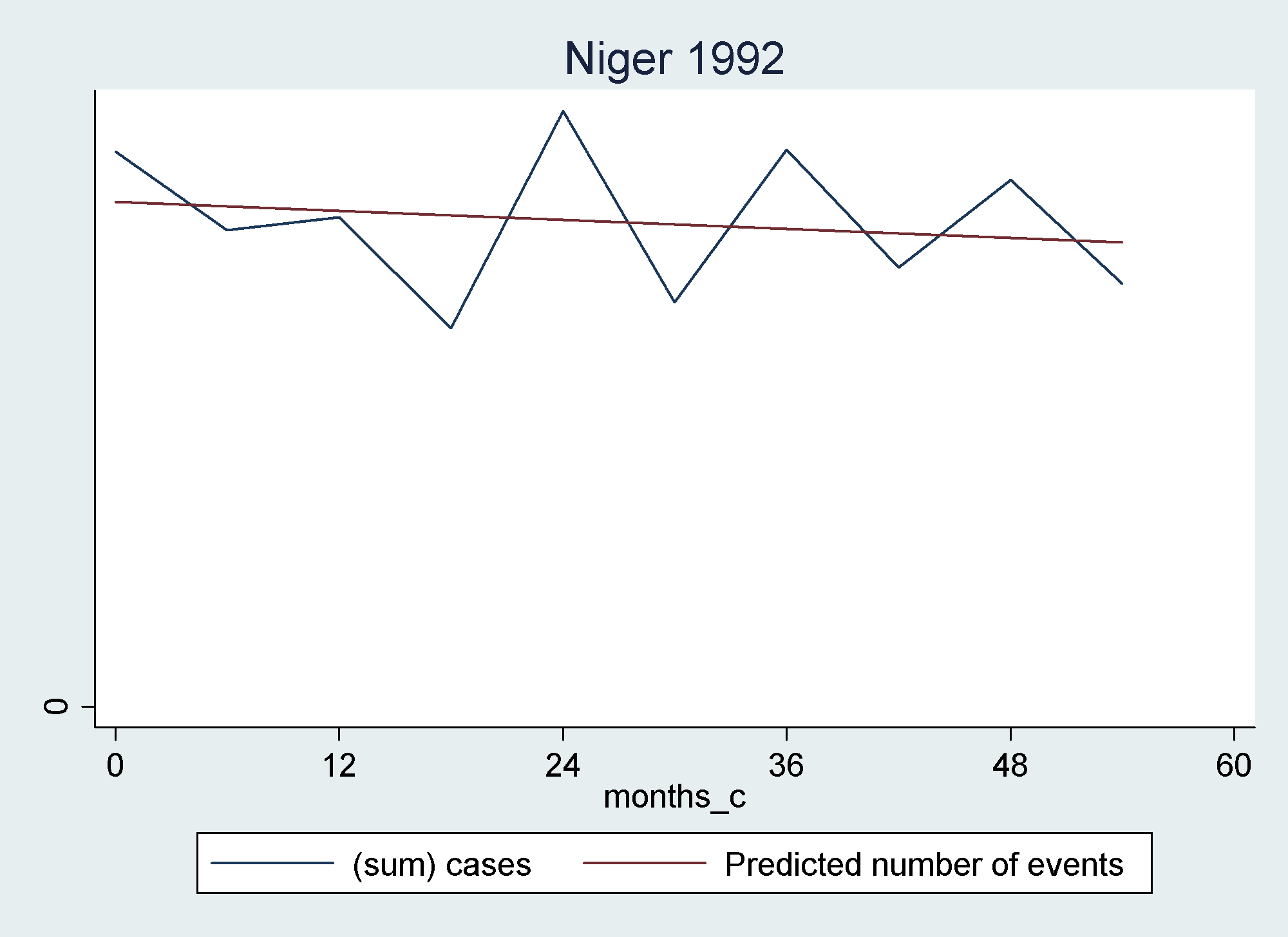 | 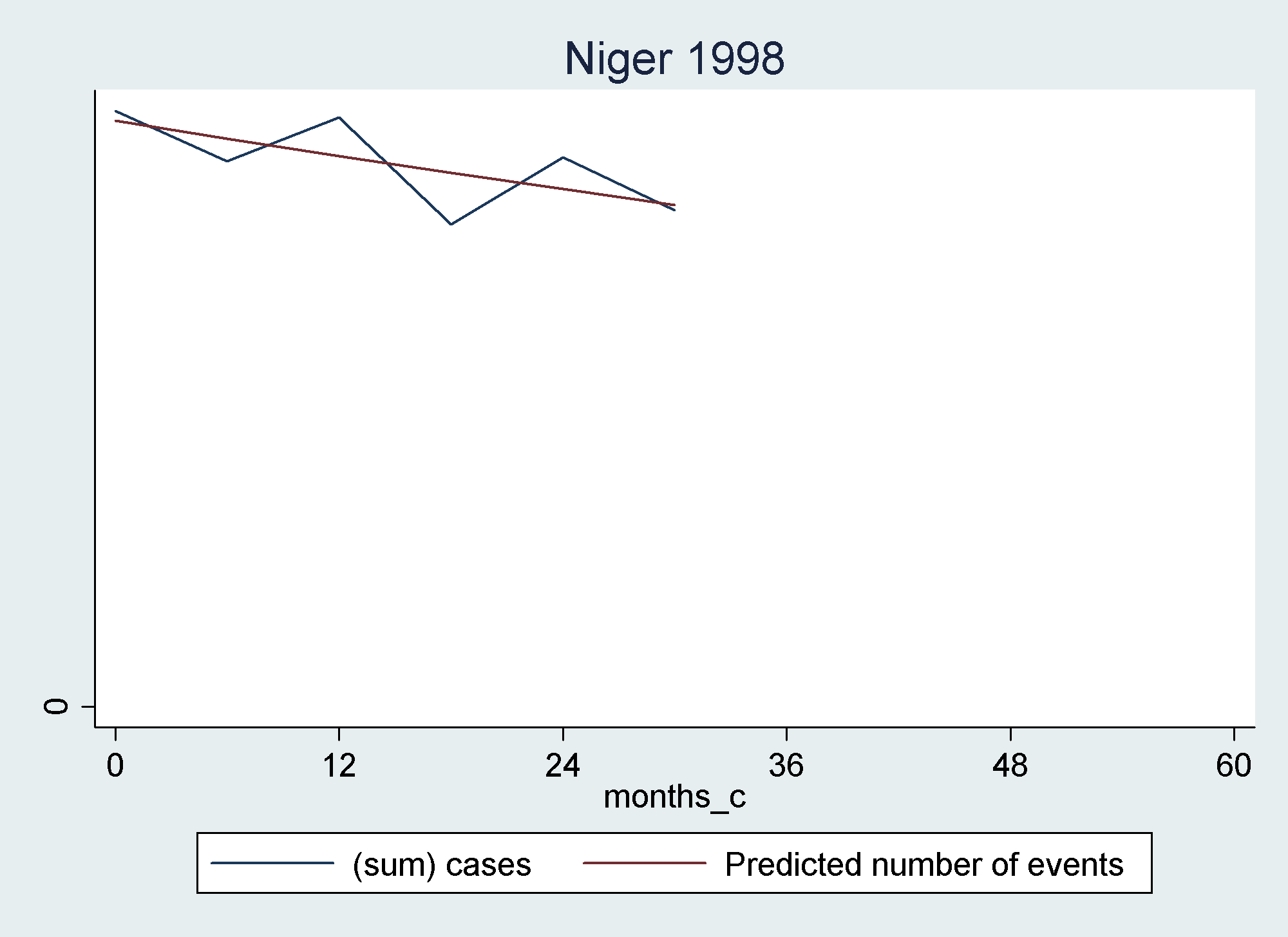 |
| 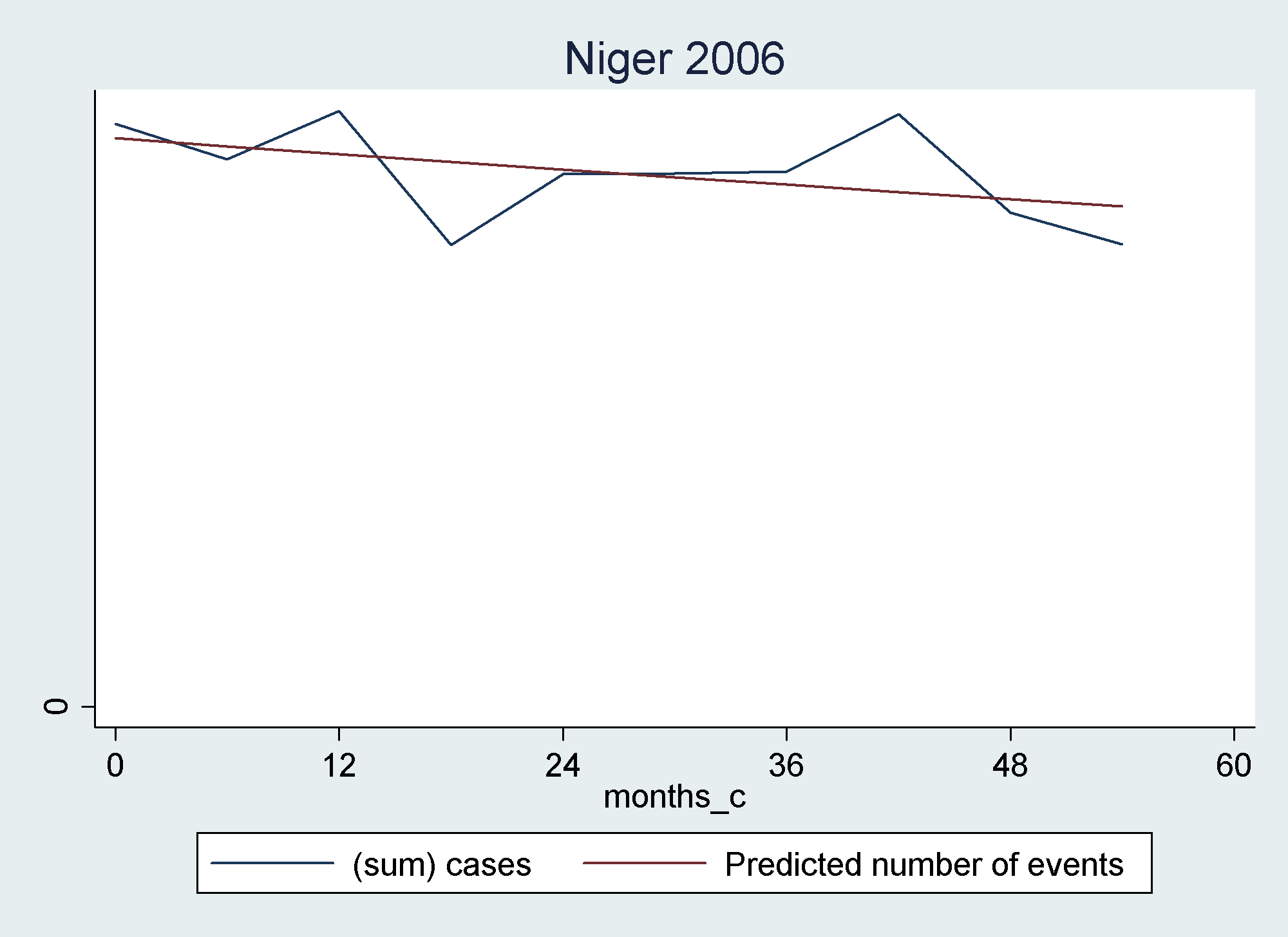 | 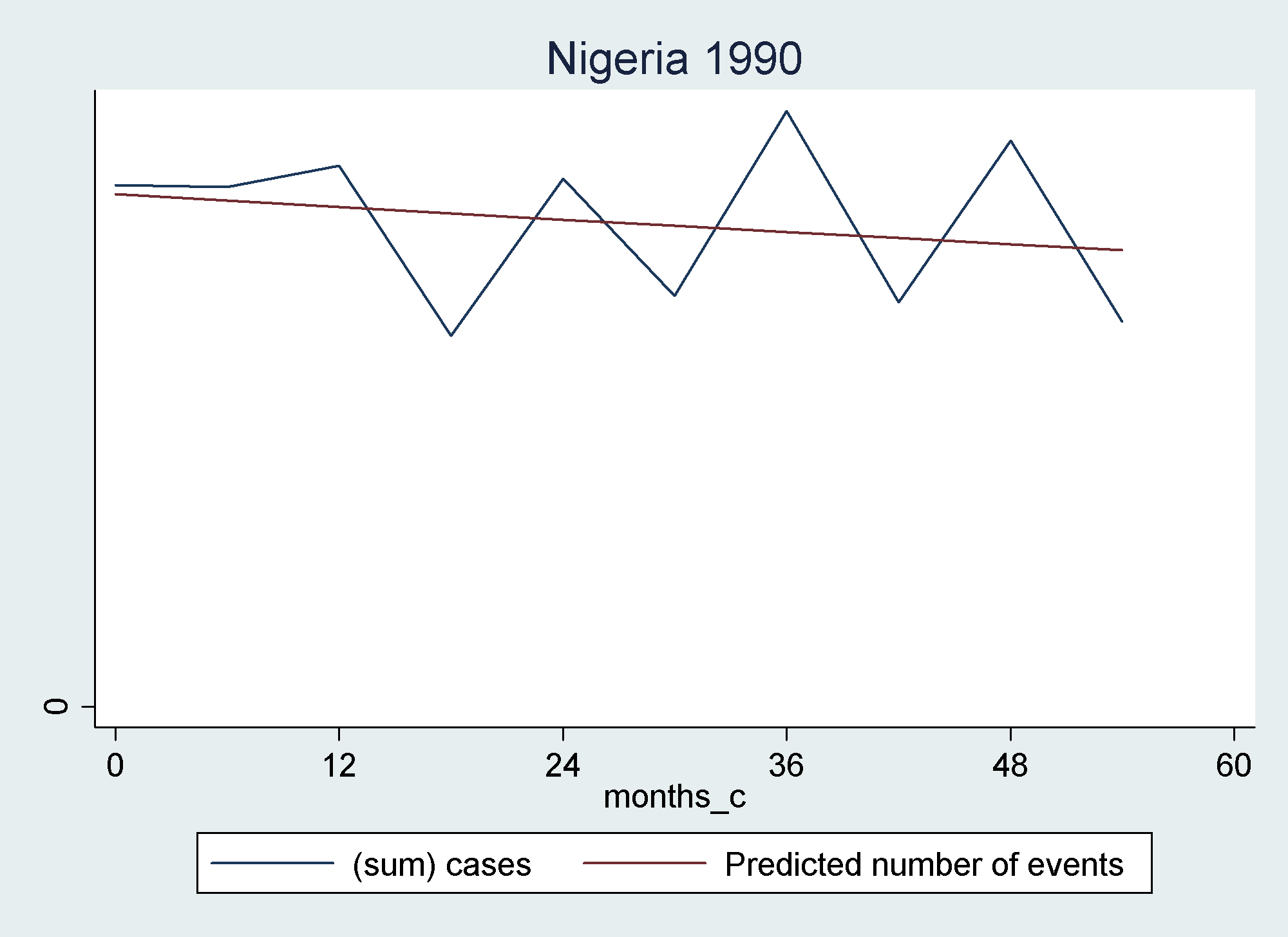 |
| 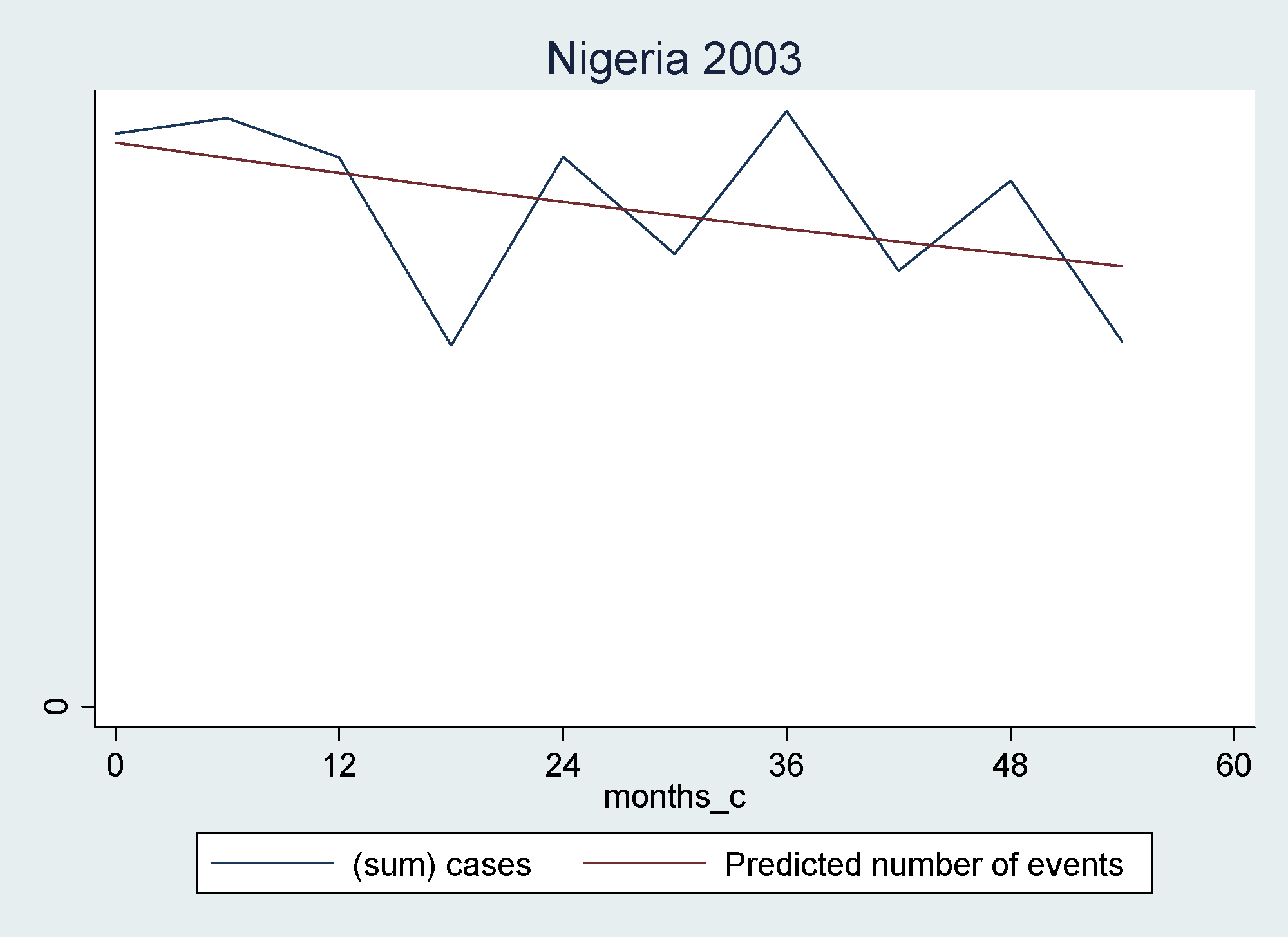 | 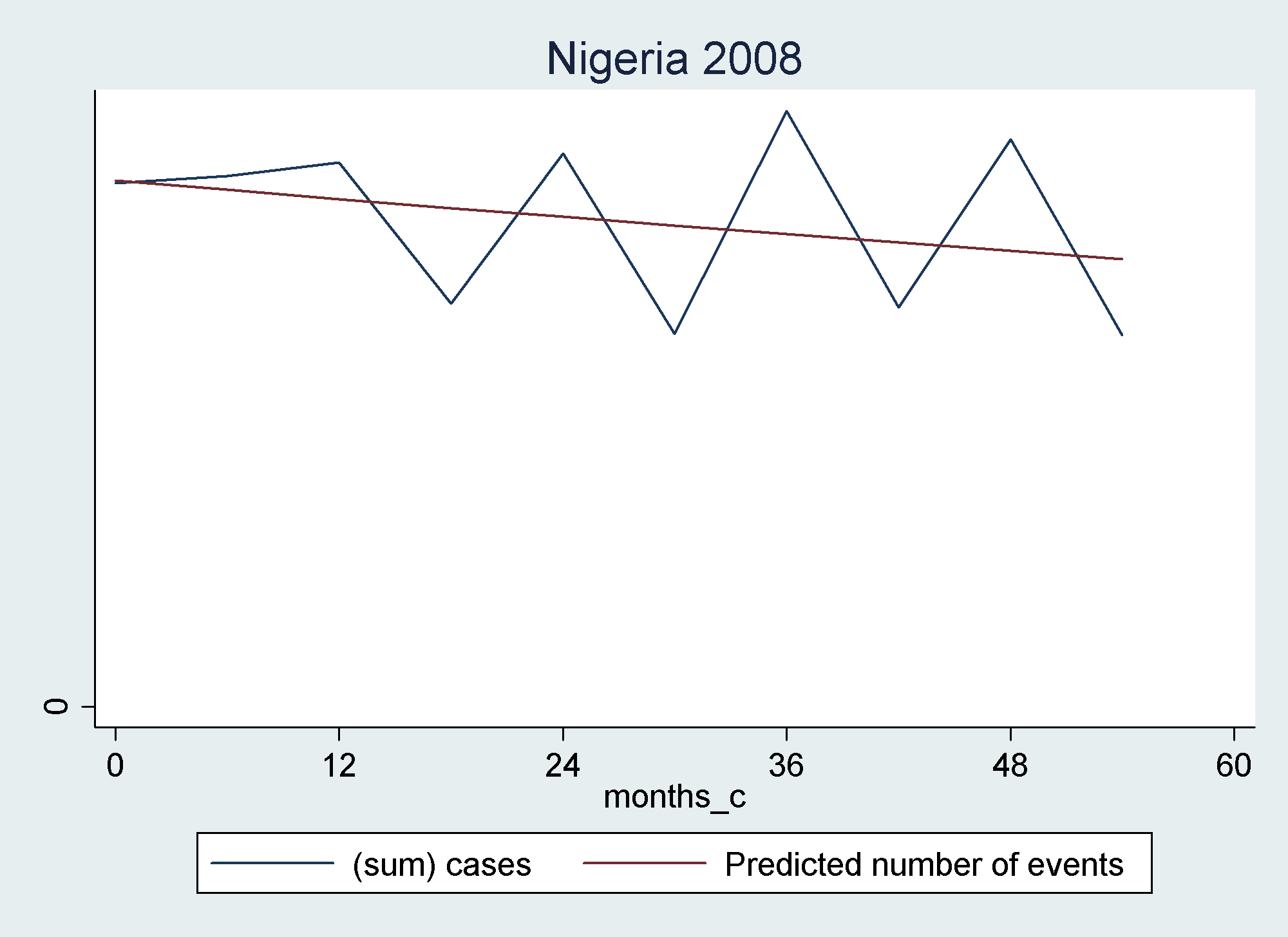 |
| 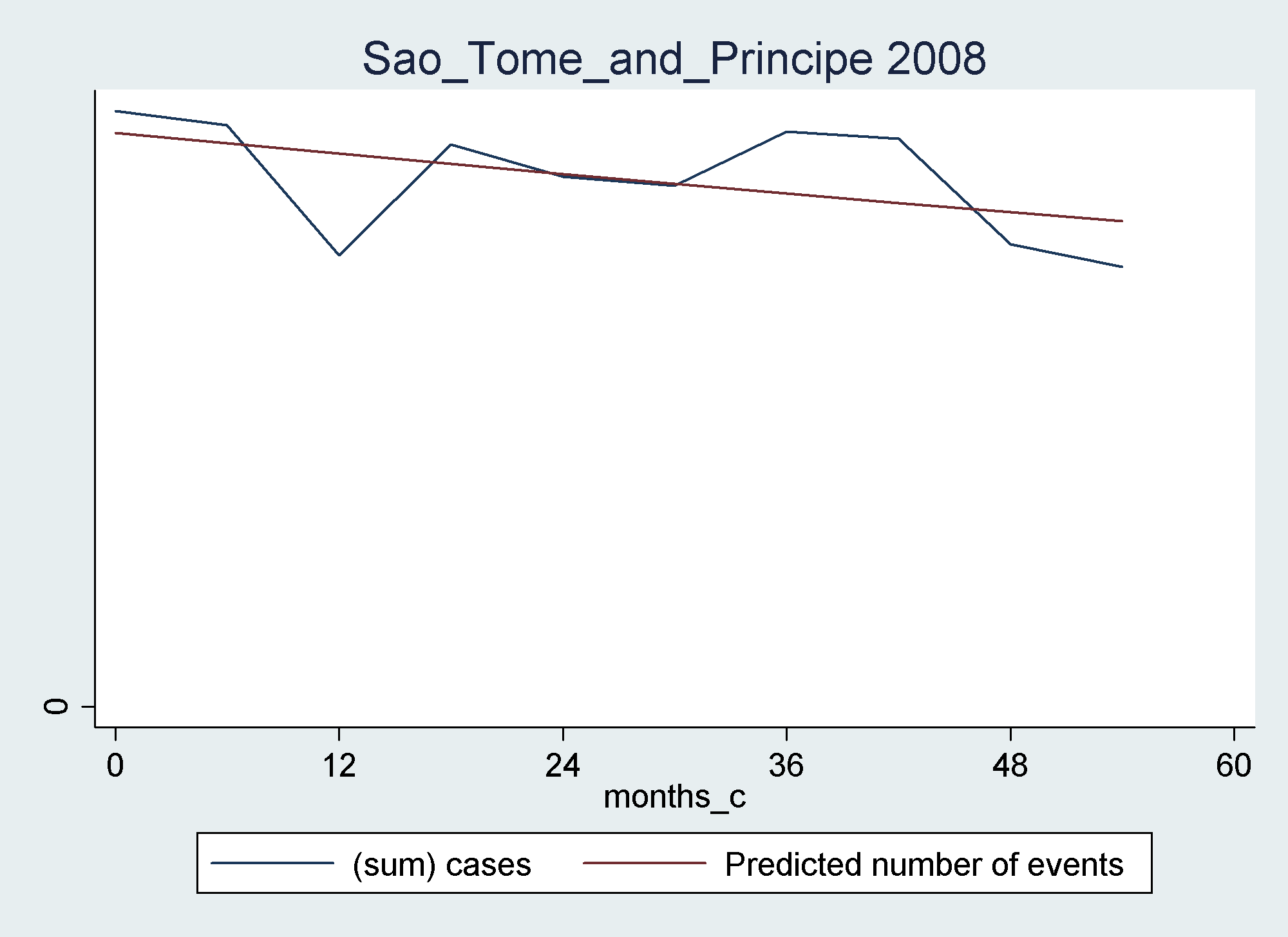 | 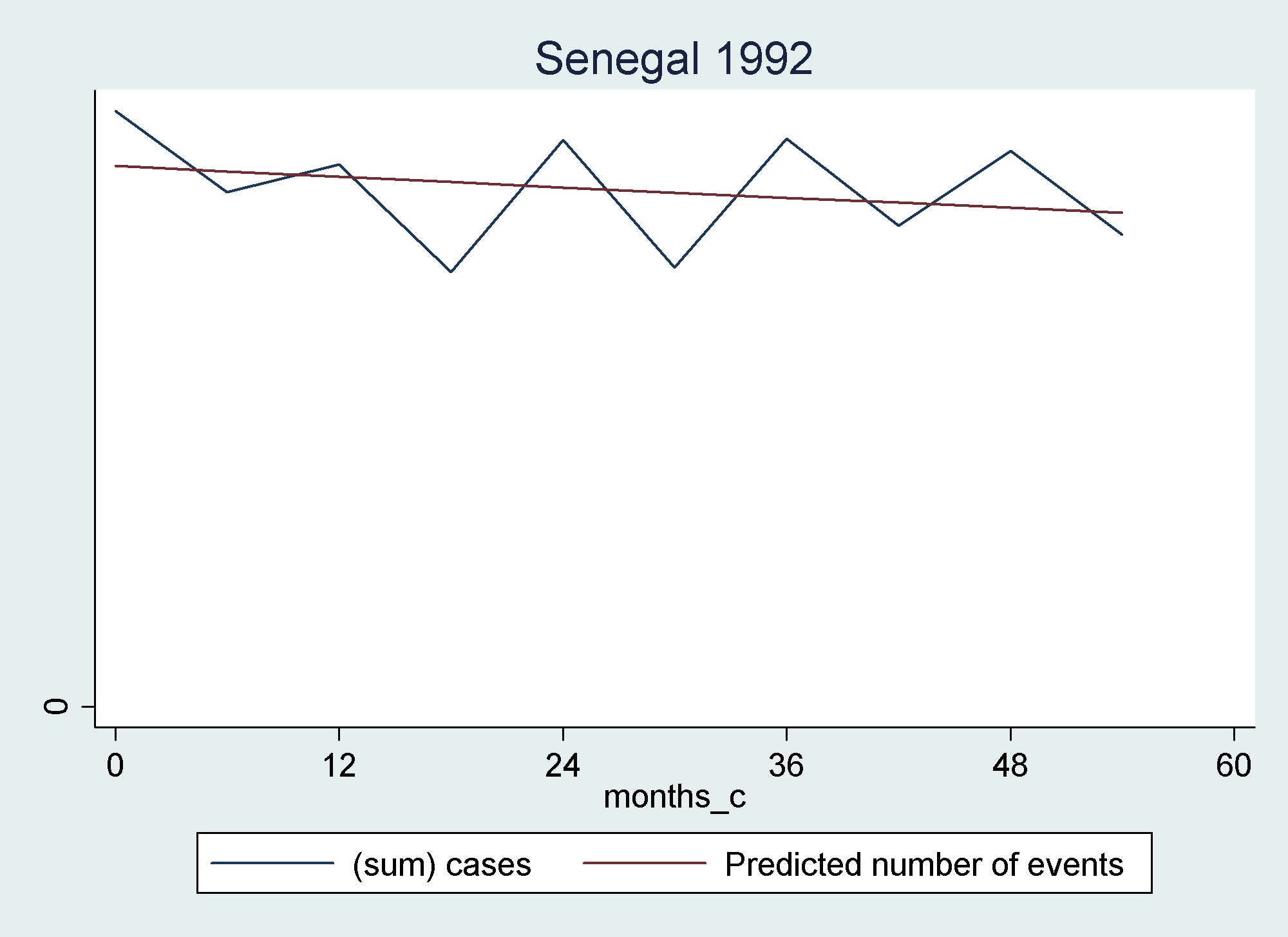 |
| 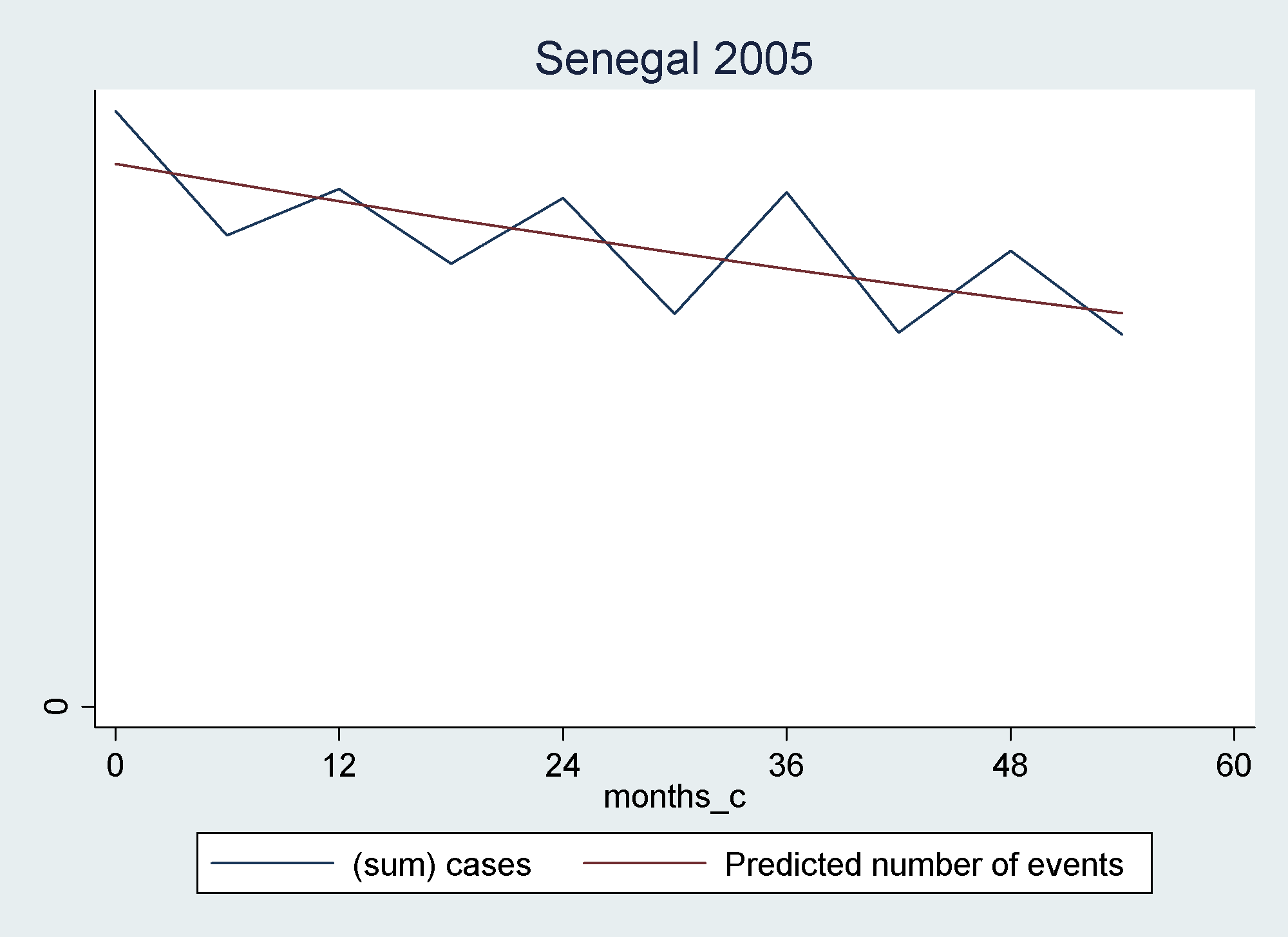 | 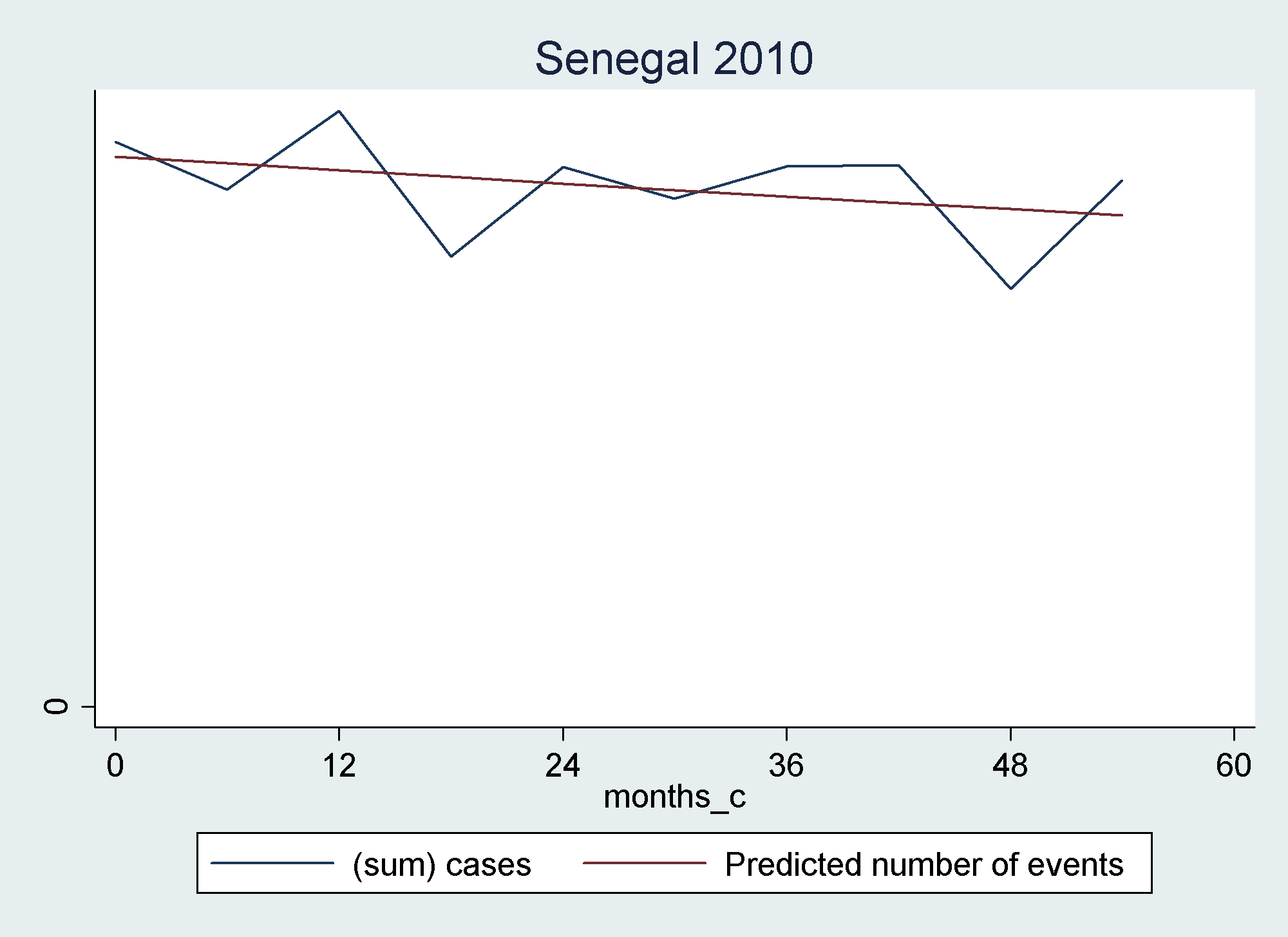 |
| 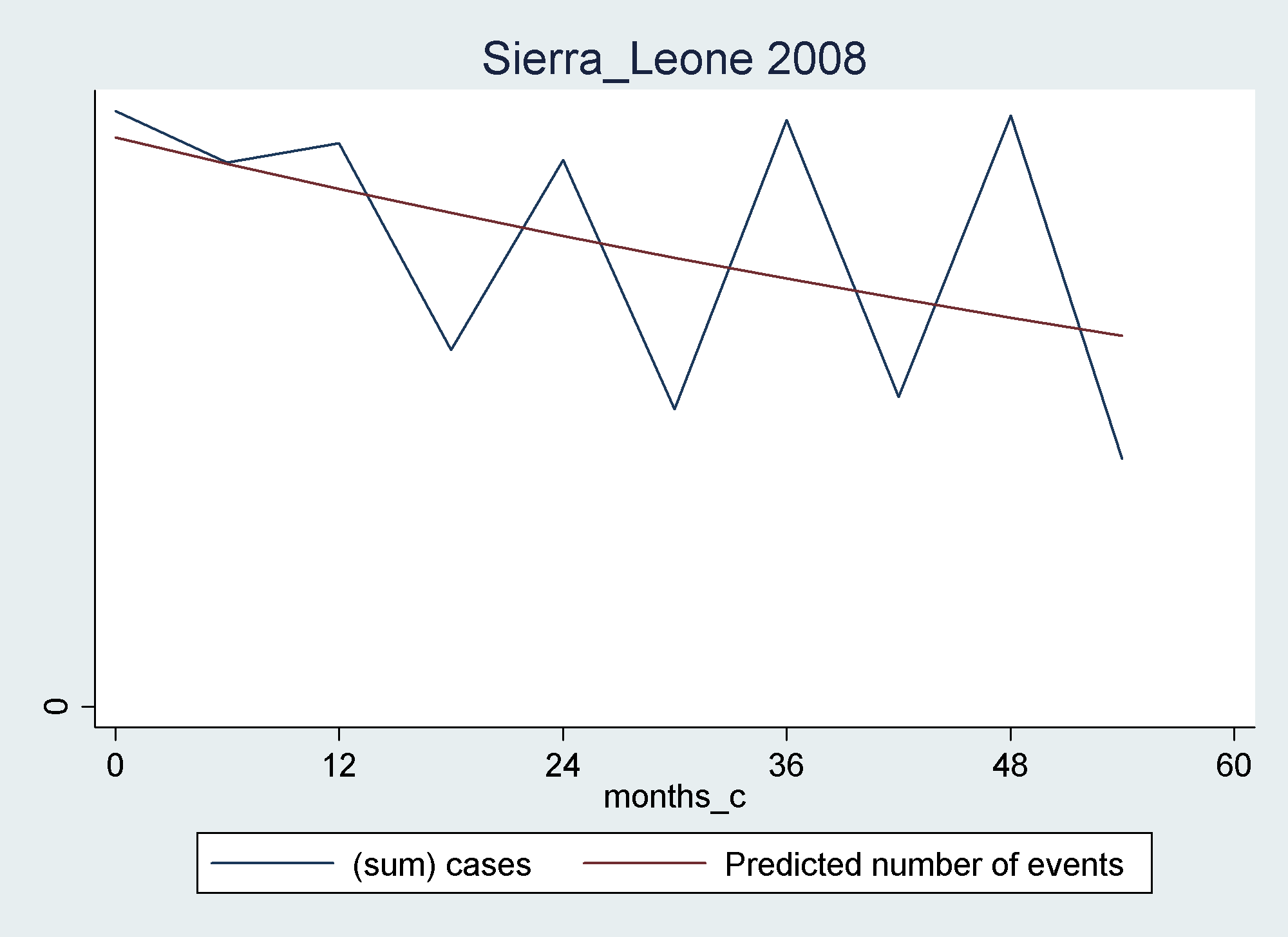 | 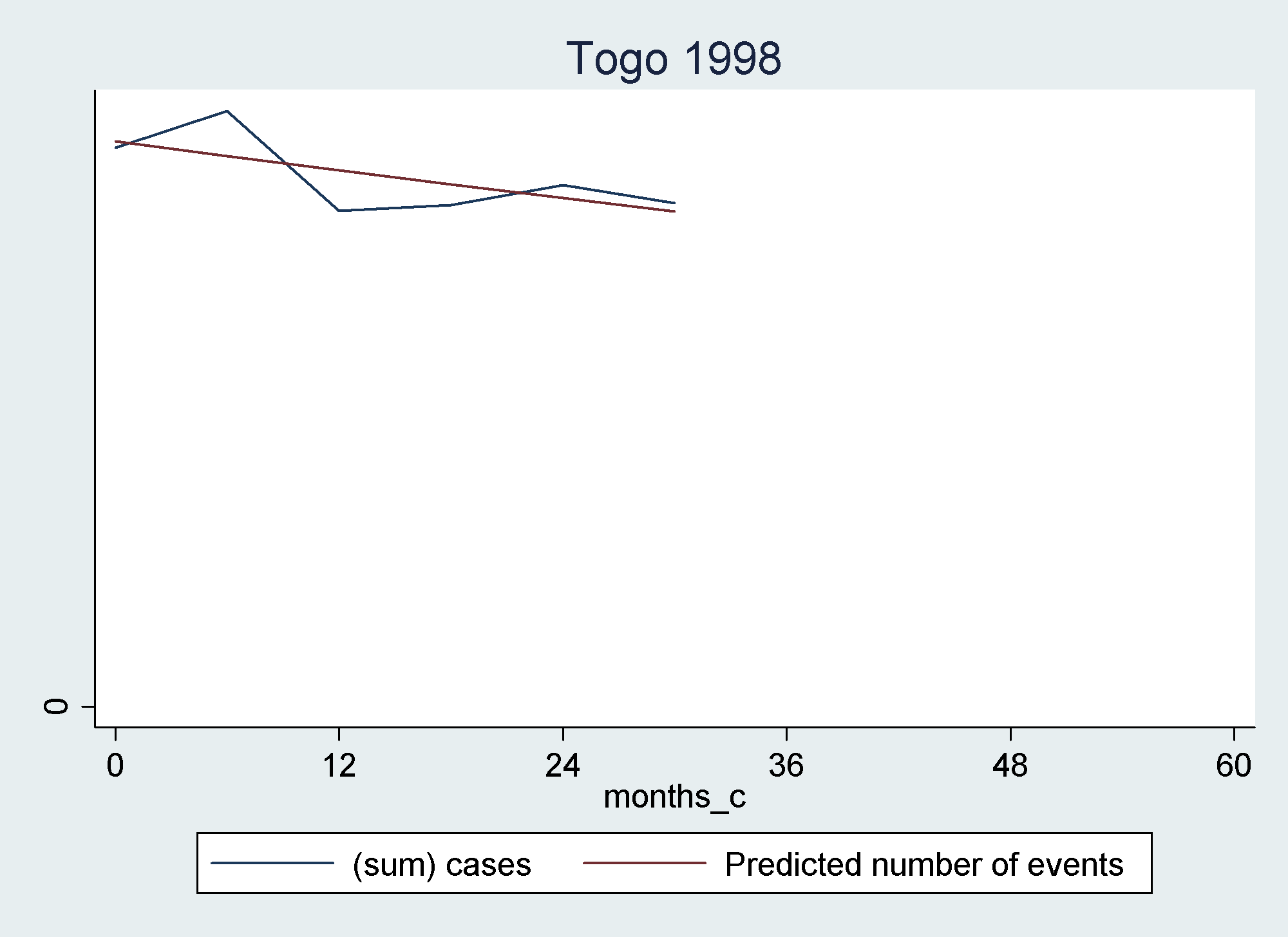 |
